# Evidence for Dicot Plants as Alternative Hosts of Banana Bunchy Top Virus and Its Alphasatellites in South-East Asia

**DOI:** 10.3390/pathogens12111289

**Published:** 2023-10-28

**Authors:** Valentin Guyot, Ngoc-Sam Ly, Tien-Dung Trieu, Oudomphone Insisiengmay, Ting Zhang, Marie-Line Iskra-Caruana, BforBB Consortium, Mikhail M. Pooggin

**Affiliations:** 1 PHIM Plant Health Institute, University of Montpellier, INRAE, CIRAD, IRD, Institute Agro, 34398 Montpellier, France; 2 Institute of Tropical Biology, Vietnam Academy of Science and Technology, Ho Chi Minh City 721400, Vietnam; 3 Northern Mountainous Agriculture and Forestry Science Institute, Phu Tho City 290000, Vietnam; 4 Life Science Research Centre, Science and Innovation Research Institute, Ministry of Education and Sports, Dontiew Road, Xaythany District, Vientiane 99241, Laos; 5 Kunming Institute of Botany, Chinese Academy of Sciences, Kunming 650201, China; 6 CIRAD, DGD-RS, 34398 Montpellier, France

**Keywords:** banana bunchy top virus, alphasatellite, host range, *Musa*, Commelina, Chromolaena, *Bidens*

## Abstract

Banana bunchy top virus is a multicomponent circular ssDNA virus (family *Nanoviridae*) that causes one of the most devastating diseases of cultivated bananas and plantains (family Musaceae). It is transmitted by the aphids *Pentalonia nigronervosa* and *P. caladii* among host plants of Musaceae and some other families of monocots. Our Illumina sequencing reconstruction of virome components of BBTV-infected banana plants and their neighbor non-banana plants sampled in Vietnam and Laos revealed the monocot *Commelina* sp. (Commelinaceae) and the dicots *Bidens pilosa* and *Chromolaena odorata* (both Asteraceae) as hosts of BBTV and circular ssDNA alphasatellites (family *Alphasatellitidae*). Counting the proportions and relative abundances of Illumina reads representing BBTV genome components and alphasatellites suggested that Chromolaena and Commelina are poor hosts for BBTV and one to three alphasatellite species, whereas Bidens is a permissive host for BBTV and four alphasatellite species representing two genera of *Alphasatellitidae*. Our findings provide evidence for the dicot plants of family Asteraceae as alternative hosts of BBTV and its alphasatellites, which warrants further investigation of these and other dicots as a potential refuge and source of BBTV and multiple alphasatellites that become associated with this virus and likely affect its replication, transmission, and host range.

## 1. Introduction

Banana bunchy top virus (BBTV, genus *Babuvirus*, family *Nanoviridae*) causes severe disease in cultivated bananas and plantains (*Musa* sp.) and represents a serious threat to global food security. Since the beginning of the 19th century, the disease has resulted in devastating epidemics, reducing banana production by up to 95% in several countries in South-East Asia, Oceania, and Africa [1,2]. BBTV is transmitted by the banana aphid *Pentalonia nigronervosa* and its relative *P. caladii* [3]. The BBTV genome is composed of six circular single-stranded (ss)DNA components of ca. 1.0 to 1.1 kilobases (Kb), each encoding one protein and each encapsidated individually in isometric 18–20 nm virions. DNA-S encodes the capsid protein, while DNA-R encodes a master replication protein (Rep) that recruits the host DNA polymerase machinery for replication of DNA-R itself and trans-replication of other BBTV components. DNA-C encodes a cell-cycle link protein that facilitates replication, while DNA-M and DNA-N encode movement and nuclear shuttle proteins. DNA-U3 encodes a small protein of unknown function [4,5,6]. Sequenced isolates of BBTV are classified into two phylogenetic groups with a distinct geographic delineation—Pacific and Indian Oceans (PIO) and South-East Asia (SEA). In South-East Asia, BBTV is frequently associated with one or more circular ssDNA alphasatellites (family *Alphasatellitidae*) that are similar in size to BBTV genome components and encode a Rep-like protein that mediates replication of alphasatellite DNA. Alphasatellites depend on their helper viruses for movement, encapsidation, and transmission [7,8,9].

The host range of BBTV has so far been reported to be restricted to plant species of the monocot families Musaceae (*Musa acuminata*, *M. balbisiana*, *M. coccinea*, *M. jackeyi*, *M. ornata*, *M. textilis*, *M. velutina*, and *Ensete ventricosum*, as well as *M. acuminata* × *balbisiana* hybrids), Zingiberaceae (*Alpinia zerumbet*, *Zingiber officinale*, *Curcuma longa*, and *Kaempferia galanga*), Araceae (*Colocasia esculenta*), Cannaceae (*Canna indica*), and Heliconiaceae (*Heliconia aurantiaca*) [10,11,12,13,14]. Studies of alternative hosts for BBTV were mainly conducted on plant species co-cultivated with or grown near banana plants and those hosting *Pentalonia* aphids. The host range of *P. nigronervosa* and *P. caladii* is also restricted to the monocot families, including Araceae, Cannaceae, Commelinaceae, Heliconiaceae, Musaceae, Strelitzeaceae, and Zingiberaceae [15,16] and thus overlaps with the BBTV host range.

## 2. Results and Discussion

During our surveys in South-East Asia (Vietnam, Laos, and China) in 2018 and 2019, leaf samples of wild and cultivated banana plants displaying BBTV symptoms were collected together with leaf samples of non-banana plants grown in close vicinity of the banana plants (Table 1; Supplementary Figure S1).

The non-banana species included the monocot *Commelina* sp. (Commelinaceae) and the dicots *Arachis hypogaea* (Fabaceae), *Bidens pilosa* (Asteraceae), *Chromolaena odorata* (Asteraceae), *Ipomoea aquatica* (Convolvulaceae), and *Phyllanthus* sp. (Phyllanthaceae). Notably, *B. pilosa* plants exhibited leaf chlorosis (Supplementary Figure S2). The total DNA extracted from the leaf samples (dried over silica gel after sampling) was used for the enrichment of circular viral DNA by rolling circle amplification (RCA) and Illumina sequencing of the resulting RCA products, followed by (i) de novo assembly of 125 nt paired-end Illumina reads to reconstruct complete genomes of circular DNA virome components and (ii) the verification of consensus sequences of the reconstructed genomes by read mapping and analysis (see Section 3). The virome components reconstructed from the banana and non-banana samples are listed in Table 1, and their sequences with annotations are provided in Supplementary Dataset S1 and deposited in the NCBI GenBank.

In the samples of *A. hypogaea* (ALYU-31), *I. aquatica* (ALYU-28), *Phyllanthus* sp. (ALYU-30), and 2 of the 26 banana plants (ALYU-46 and ALYU-52), very low numbers of viral (BBTV and alphasatellite) reads were detected; no complete consensus viral component could be reconstructed from these reads. We assumed that these reads represent cross-contamination from other samples multiplexed and sequenced in one flow cell of Illumina HiSeq2500. This assumption was supported by the inspection of read coverage profiles with and without mismatches (Supplementary Figure S3). PCR analysis of the RCA products using primers specific for a conserved region of BBTV DNA-R (see Section 3) showed that the banana samples ALYU-46 and ALYU-52, were negative, while all the other samples, including ALYU-28, ALYU-30, and ALYU-31, were PCR-positive (Table 1). The latter three samples were taken to establish the cross-contamination threshold (0.0006% of total reads; Supplementary Figure S4). Based on this threshold and the read coverage profiles (Supplementary Figure S3), *Commelina* sp. (ALYU-27; hereafter Commelina), *B. pilosa* (ALYU-38; hereafter Bidens), and *C. odorata* (ALYU-41; hereafter Chromolaena) were considered to be infected with BBTV and coinfected with one or more alphasatellites (Table 1; Supplementary Dataset S1; Supplementary Figure S3).

**Figure 1:**
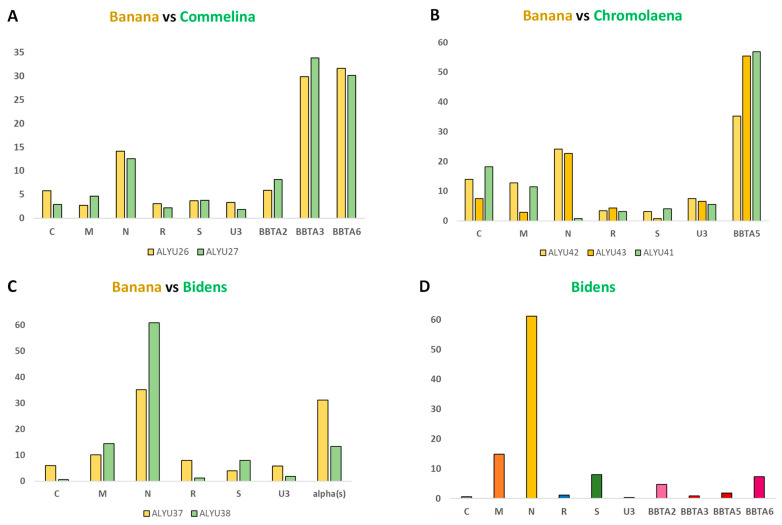
Relative abundance (formula) of virome components in Commelina, Chromolaena, and Bidens and their neighbor banana plants. Illumina DNA-seq reads were mapped to the genome sequences of BBTV genome components (C, M, N, R, S, and U3) and alphasatellites (BBTA2, BBTA3, BBTA5, and BBTA6) reconstructed from each plant sample (ALYU) and their relative abundance was calculated and plotted in percentages of total viral (BBTV + alphasatellite) reads. Panels show comparisons of the virome components’ formulas in Commelina (**A**), Chromolaena (**B**) and Bidens (**C**,**D**) versus their banana neighbors.

Both Commelina and its neighbor banana (ALYU-26) sampled in Vietnam shared 100% identical consensus sequences of all the virome components, including six components of BBTV genome and three species of BBTV alphasatellites (BBTA2, BBTA3, and BBTA6) (Supplementary Dataset S1), although single-nucleotide polymorphism (SNP) profiles of their virome quasispecies population (calculated using MISIS-2; [17]) differed substantially. Furthermore, relative abundances of their virome components (virome DNA formulas) were found to be similar but not identical (Figure 1A). The percentage of viral reads in the total (plant + viral) reads was found to be much lower in Commelina (0.004%) than in its neighbor banana (0.64%) (Supplementary Figure S4). These findings suggest that Commelina is a poor host for BBTV and its alphasatellites, allowing very low accumulation of viral DNA compared to bananas.

The viromes of Chromolaena (ALYU-41) and its two banana neighbors (ALYU-42 and ALYU-43) sampled in Laos all contained BBTV and a single alphasatellite (BBTA5). However, both BBTV and BBTA5 were represented with distinct genetic variants in Chromolaena and each banana neighbor (Supplementary Dataset S1). Most notably, DNA-N, one of the most abundant BBTV components in banana plants, was at the cross-contamination threshold level in Chromolaena, DNA-U3 in Chromolaena differed at multiple nucleotide positions (ca. 92% identity to banana neighbors), while BBTA5 differed at single distinct nucleotide positions in all three samples (Supplementary Dataset S1). The virome DNA formulas differed substantially not only between Chromolaena and its banana neighbors (due to the absence of DNA-N in the former) but also between the two banana neighbors (Figure 1B). The percentage of viral reads in total reads was found to be much lower in Chromolaena (0.002%) than in its banana neighbors (0.13% in ALYU-42 and 0.22% in ALYU-43) (Supplementary Figure S4). Thus, similar to Commelina, Chromolaena appeared to be a poor host for BBTV and alphasatellite BBTA5. Previously, we have shown that DNA-N could be lost upon aphid transmission without affecting BBTV disease symptoms in recipient banana plants, but the virus lacking DNA-N was not transmissible by *P. nigronervosa* [9]. Likewise, the DNA-N of faba been necrotic yellows virus (genus *Nanovirus*, family *Nanoviridae*) is essential for aphid transmission but not for disease symptom development [18,19]. Based on these findings, BBTV-infected *C. odorata* lacking DNA-N is unlikely to serve as a source for virus transmission, while the self-replicating alphasatellite BBTA5 as the most abundant virome component (Figure 1B) likely encapsidated by BBTV capsid protein (expressed from DNA-S) can potentially be transmissible to other plants by viruliferous aphids carrying complete BBTV.

The viromes of Bidens (ALYU-38) and its banana neighbor (ALYU-37) sampled in Vietnam shared six-component BBTV and one alphasatellite (BBTA2), with all these seven components having 100% identity in their consensus sequences. In addition, the virome of Bidens contained three more alphasatellites (BBTA3, BBTA5, and BBTA6) and two Rep-encoding circular ssDNA viruses, both classified as novel species in the non-plant families *Microviridae* and *Circoviridae* (Supplementary Dataset S1); all these additional components were below the detection threshold in the banana neighbor. Remarkably, the alphasatellites BBTA3 and BBTA6 identified in Bidens share 100% sequence identity with the respective alphasatellites identified in Commelina and one of its banana neighbors (ALYU-26) sampled at a far-away location (Supplementary Figure S1). Likewise, the alphasatellite BBTA5 from Bidens shares 100% sequence identity with BBTA5 identified in the banana ALYU-40 at another far-away location in Vietnam (Supplementary Figure S1). Note that the genome sequence of helper BBTV in Bidens differs from the genome sequences of helper BBTV in the ALYU-26 and ALYU-40 bananas (respectively, 98.1 and 98.2% pairwise identity in DNA-C; 95.3 and 97.9% in DNA-M; 98.3 and 98.5% in DNA-N; 98.4 and 98.9% in DNA-R; 98.2 and 99.1% in DNA-S; and 97.9 and 98.3% in DNA-U3; Supplementary Dataset S1), thus excluding cross-contamination between the samples. These findings highlight the genetic stability of BBTV alphasatellites, consistent with our analysis of all 26 isolates representing the four alphasatellite species in Vietnam (Table 1; Valentin Guyot, Marie-Line Iskra-Caruana, and Mikhail Pooggin; unpublished data). The virome DNA formulas in Bidens and its banana neighbor were found to be different, although in both cases, DNA-N and (combined) alphasatellite DNA accumulated at high levels (Figure 1C; Supplementary Figure S3). Most notably, the combined BBTV and alphasatellite reads constituted 0.24% of the total (plant + viral) reads in Bidens, which is more than 50 times higher than in Commelina and Chromolaena and only ~4 times lower than in its banana neighbor (0.90%) (Supplementary Figure S4). These findings indicate that *B. pilosa* is a permissive host for BBTV and all four BBTV alphasatellite species currently circulating in South-East Asia (Table 1).

Collectively, our findings suggest that viruliferous banana aphids could have transmitted BBTV and alphasatellites from the respective banana neighbors not only to *Commelina* sp., which represents the monocot family Commelinaceae and falls within the known host range of *Pentalonia* aphids and BBTV, but also to *C. odorata* and *B. pilosa*. Both *C. odorata* and *B. pilosa* represent the dicot family Asteraceae, which was unexpected to host BBTV or banana aphids. In the latter case, even a short-term probing and salivation event would be sufficient for the release of viral particles circulating in the aphid body and accumulating in the salivary glands [3,20]. It remains to be investigated if *Pentalonia* aphids can feed on BBTV- and alphasatellite-infected Bidens and Chromolaena plants for a prolonged time to acquire BBTV and its alphasatellites and transmit them among these and other Asteraceae plants or back to Musaceae hosts. Previous studies have established a minimal acquisition access period of 4 h for banana aphids on BBTV-infected banana plants to transmit the virus to new plants with a minimal inoculation access period of 15 min [21,22].

Phylogenetic analysis of the alphasatellites associated with our BBTV isolates from Vietnam, Laos, and China (Table 1) revealed that, besides new genetic variants of BBTA2 and BBTA3 species previously found to be associated with other BBTV isolates from South-East Asia and classified in the genus *Muscarsatellite* of the subfamily *Petromoalphasatellitinae* (comprising alphasatellites of several genera that infect monocots), other alphasatellites represent two new species, BBTA5 and BBTA6, which belong to a tentative genus *Banaphisatellite* within the subfamily *Nanoalphasatellitinae* ([9], Valentin Guyot, Marie-Line Iskra-Caruana, and Mikhail Pooggin; unpublished data). Until recently, the latter subfamily was known to comprise several genera of alphasatellites that infect dicots. The first species classified in the genus *Banaphisatellite* was banana bunchy top alphasatellite 4 (BBTA4), recently discovered to be associated with BBTV isolates from the banana aphid and plant samples collected in Africa (Democratic Republic of the Congo) [9]. A hypothetical dicot origin of the banaphisatellite BBTA4 was proposed based on its phylogeny and the ability of its clone to infect the model dicot *Nicotiana benthamiana* (Solanaceae) [9]. This hypothesis is now supported by the above-described findings that the banaphisatellites BBTA5 and BBTA6 can naturally infect the dicot plants *Bidens pilosa* and *Chromolaena odorata*.

## 3. Materials and Methods

### 3.1. Surveys in Vietnam, Laos, and China

Leaf samples of banana plants displaying characteristic BBTV disease symptoms and non-banana plants grown in close vicinity of the BBTV-infected banana plants (see Supplementary Figure S2) were collected during surveys in Vietnam (2018) and in Laos and China (2019) and were locally dried using silica gel. Non-banana plants did not display any strong symptoms except for leaf chlorosis in the case of *B. pilosa* (Supplementary Figure S2). Each ALYU sample in Table 1 represents a single leaf (or a pool of leaves from a single plant) per plant species per location.

### 3.2. Total DNA Extraction from Banana Leaf Samples

Dried leaf tissue (100 mg) was ground in liquid nitrogen, and 500 µL extraction buffer (100 mM Tris-HCl pH 8.0, 1.4 M NaCl, 20 mM EDTA, 2% alkyltrimethylamonium bromide, 1% polyethyleneglycol 6000, and 0.5% sodium sulfite) pre-heated at 74 °C and supplemented with 0.4 µL RNase (100 mg/mL) was added to the frozen powder. The mixture was vortexed for 20 s, incubated at 74 °C for 20 min, and then mixed vigorously with one volume of chloroform–isoamyl alcohol (24:1 *v*/*v*) (CIAA), followed by centrifugation at 13,000 rpm for 30 min at 4 °C. The supernatant was taken for a second round of extraction with CIAA, followed by centrifugation as described above. The supernatant was mixed with one volume of isopropanol pre-cooled at −20 °C. The mixture was shaken until appearance of a hank and was then spun at 13,000 rpm and 4 °C for 30 min. The pellet was washed twice with 500 µL of 70% ethanol, air-dried, and dissolved in 100 µL of milli-Q water.

### 3.3. Rolling Circle Amplification (RCA)

Circular viral DNA components were enriched in total DNA by RCA using a TempliPhi RCA kit (GE Healthcare, Chicago, IL, USA) following the manufacturer’s protocol. Briefly, 5 μL sample buffer and 1 μL total DNA extracted from leaf tissues were mixed and heated at 95 °C for 3 min. The samples were cooled in ice, and 5 μL reaction buffer and 0.2 μL enzyme mix were added, followed by incubation at 30 °C for 18 h. The enzyme was inactivated by heating at 65 °C for 10 min. RCA products were purified using NucleoSpin Gel and PCR Clean-up kit (Macherey-Nagel, Allentown, PA, USA) following the manufacturer’s protocol. DNA concentration was measured by Qubit fluorimeter using Qubit dsDNA HS Assay Kit (Thermo Fischer Scientific, Waltham, MA, USA).

### 3.4. Polymerase Chain Reaction (PCR) Analysis

The RCA products were analyzed by PCR using a pair of primers (5′-GGCGCGATATGTGGTATGCTGG and 5′-CCAAACTCGAAGGGACCTTCG) specific for a conserved region of DNA-R, yielding a 285 bp product. The PCR reaction was performed in a volume of 25 µL containing 1 µL of the RCA-treated total DNA, 2 µL primer mix (10 µM each primer), 1 µL of 2.5 mM dNTPs, 5 µL of 5× GoTaq buffer, and 1U GoTaq DNA polymerase (Promega, Madison, WI, USA). After denaturation at 94 °C for 5 min, DNA was amplified for 35 cycles of 30 s at 94 °C, 30 s at 56 °C, and 30 s at 72 °C, followed by a final extension at 72 °C for 10 min. The results of the PCR analysis are shown in Table 1.

### 3.5. Illumina Sequencing of RCA Products and De Novo Reconstruction of Viral Genomes

Fifty ng of the cleaned RCA products were taken for Illumina sequencing at Fasteris AG (www.fasteris.com; accessed on 23 October 2023). Libraries were prepared using Nextera XT standard DNA protocol, and all libraries were multiplexed and sequenced in one flow cell of HiSeq2500 with a 2× 125-nt paired-end run. Viral genomes were de novo reconstructed from the sequencing reads of each library by selecting unique inserts sequenced ≥5, ≥10, ≥20, ≥30, ≥40, or ≥50 times and assembling them using Velvet v. 1.2.10 [23] with k-mers 77, 79, 83, 87, 91, 95, 99, 103, 107, 111, 113, and 117. All the resulting Velvet contigs were scaffolded using SeqMan Pro v. 7.1.0 (DNASTAR Lasergene). SeqMan contigs of viral origin were identified by BLASTn analysis. The consensus viral genome sequences were verified using SeqMan scaffolds and validated by mapping back the Illumina reads using Burrow–Wheeler Aligner (BWA) 0.7.12 [24] and visualization using MISIS-2 [17].

## Figures and Tables

**Table 1:** Virome components identified in wild and cultivated banana (*Musa*) and non-banana plants sampled in South-East Asia.

Sample/Isolate	Country	Plant Species/Genome/Cultivar	BBTV DNA-R PCR	BBTV Genome Illumina	No. of Alpha-Satellites	BBTA2	BBTA3	BBTA5	BBTA6	Defective (d) Molecules	Other Viruses
ALYU-25	Vietnam	*Musa itinerans*	(+)	full	2	1		1			Badnavirus
ALYU-26	Vietnam	*Musa* sp.	(+)	full	3	1	1		1		
ALYU-27	Vietnam	*Commelina* sp.	(+)	full	3	1	1		1		
ALYU-28	Vietnam	*Ipomoea aquatica*	(+)	no *							
ALYU-29	Vietnam	*Musa* AA Pisang mas?	(+)	full	3	1	1	1		dA5, dA2, dR	
ALYU-30	Vietnam	*Phyllanthus* sp.	(+)	no *							
ALYU-31	Vietnam	*Arachis hypogaea*	(+)	no*							
ALYU-32	Vietnam	*Musa* sp. sweet banana	(+)	full	2	1		1		dA2	
ALYU-33	Vietnam	*Musa* AAB Chuoi Ngop	(+)	full	2	1		1			Badnavirus
ALYU-34	Vietnam	*Musa* sp.	(+)	full	2	1		1			
ALYU-35	Vietnam	*Musa* sp.	(+)	full	1				1		
ALYU-36	Vietnam	*Musa* sp.	(+)	full	1	1					
ALYU-37	Vietnam	*Musa* sp.	(+)	full	1	1					
ALYU-38	Vietnam	*Bidens pilosa*	(+)	full	4	1	1	1	1		Circo-, Microvirus
ALYU-39	Vietnam	*Musa* sp.	(+)	full	2	1			1		
ALYU-40	Vietnam	*Musa* AAA red banana	(+)	full	1			1			
ALYU-41	Laos	*Chromolaena odorata*	(+)	no N *	1			1			
ALYU-42	Laos	*Musa* AAA Cavendish	(+)	full	1			1			
ALYU-43	Laos	*Musa* AAA Cavendish	(+)	full	1			1			
ALYU-44	Laos	*Musa ornata*	(+)	full							
ALYU-45	Laos	*Musa* sp.	(+)	full							
ALYU-46	Laos	*Musa* ABB Klue Tiparot	(−)	no **							
ALYU-47	Laos	*Musa yunnenensis*	(+)	full							
ALYU-48	Laos	*Musa* sp.	(+)	full							
ALYU-49	Laos	*Musa* sp.	(+)	full							
ALYU-50	Laos	*Musa* AA Kouay niew mung	(+)	full							
ALYU-51	Laos	*Musa* ABB Pisang Awak?	(+)	full							
ALYU-52	China	*Musa acuminata* wild	(−)	no **							
ALYU-53	China	*Musa yunnanensis*	(+)	full							
ALYU-54	China	*Musa* AAA Cavendish	(+)	full	2	1			1	dA6	
ALYU-55	China	*Musa* AAA Cavendish	(+)	full	3	1	1		1	A5-U3 chimera	
ALYU-56	China	*Musa* AAA Cavendish	(+)	full	1	1					

* at (or close to) the cross-contamination threshold level. ** below the cross-contamination threshold level.

## Data Availability

Viral genome sequences obtained in this study were deposited in the NCBI Genbank under the accession numbers ON959832–ON959993 (BBTV isolates ALYU-25–56), ON959994–ON960032 (BBTV alphasatellites), ON960033–ON960034 (Badnaviruses BSVNV and BSMIV), ON960035 (Bidens microvirus), and ON960036 (Bidens circovirus).

## Supplementary Materials

The following supporting information can be downloaded at https://www.mdpi.com/article/10.3390/pathogens12111289/s1. Figure S1: Locations of banana and non-banana plant leaf sampling places in Vietnam, Laos, and China in 2018 and 2019; Figure S2: *Bidens pilosa* and its neighbor banana plants; Figure S3: Maps of Illumina sequencing reads of viral DNA enriched by rolling circle amplification of total DNA from BBTV- and alphasatellite-infected banana and their neighbor non-banana plants; Figure S4: Counts of Illumina reads representing BBTV and alphasatellite DNA from banana and non-banana neighbor plants; Dataset S1: Supplementary sequence information.
