# Supplementary material for: Evidence for Dicot Plants as Alternative Hosts of Banana Bunchy Top Virus and Its Alphasatellites in South-East Asia
Source: Pathogens. 2023 Oct 28;12(11):1289. doi: 10.3390/pathogens12111289 (PMC10675457; doi:10.3390/pathogens12111289)
Supplement: Supplementary file 1 [file pathogens-12-01289-s001.zip › Suppementary Figure S3.pdf]

Figure S3. **Maps of Illumina sequencing reads of viral DNA enriched by rolling circle amplification of total DNA from BBTV- and alphasatellite-infected banana and their neighbor non-banana plants.** The Illumina 125 nt paired-end reads were mapped without or with mismatches on the concatenated reference sequences of BBTV genome components (C, M, N, R, S, U3), alphasatellites (BBTA2, BBTA3, BBTA5, BBTA6) and their defective (d) derivatives (d-BBTA2, d-BBTA5, d-R) identified and reconstructed from each plant sample (ALYU) and the mapped reads were visualized using MISIS-2 [17]. In each panel, histograms plot the numbers of viral 125 nt forward and reverse reads at each nucleotide position of the concatenated virome genome: blue bars above the axis represent forward reads ending at each respective position red bars below the axis represent reverse reads ending at each respective position. (**A**) Musa (ALYU-26) vs Commelina (ALYU-27). (**B**) Musa (ALYU-26) vs Ipomoea (ALYU-28). (**C**) Musa (ALYU-29) vs Phyllanthus (ALYU-30). (**D**) Musa (ALYU-29) vs Arachis (ALYU-31). (**E**) Musa (ALYU-37) vs Bidens (ALYU-38). (**F**) Musa (ALYU-42) vs Chromolaena (ALYU-41). (**G**) Musa (ALYU-43) vs Chromolaena (ALYU-41). (**H**) Musa (ALYU-46) vs Musa (ALYU-52).

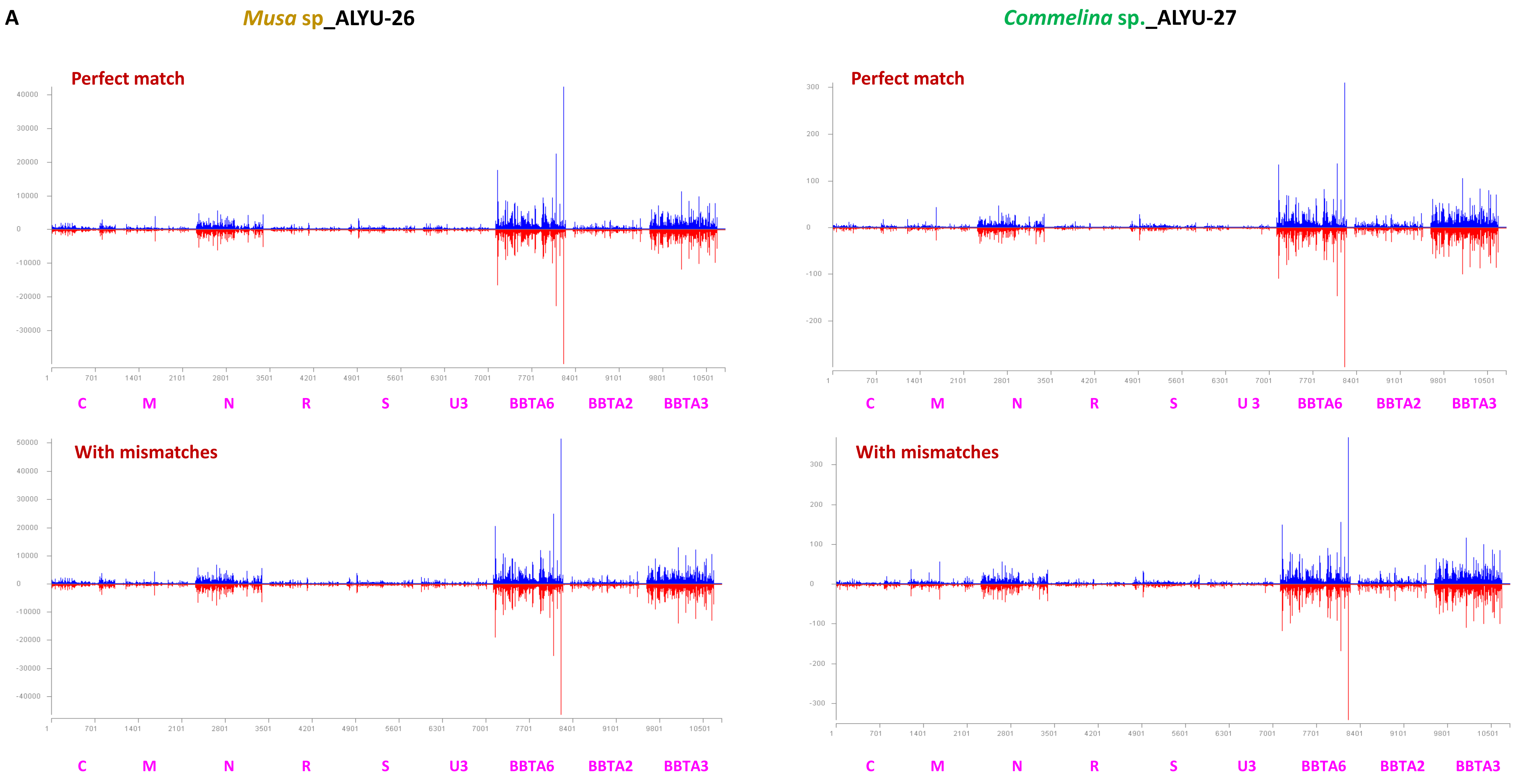
A
Musa sp_ALYU-26
Commelina sp._ALYU-27
Perfect match
With mismatches
C
M
N
R
S
U3
BBTA6
BBTA2
BBTA3
U 3

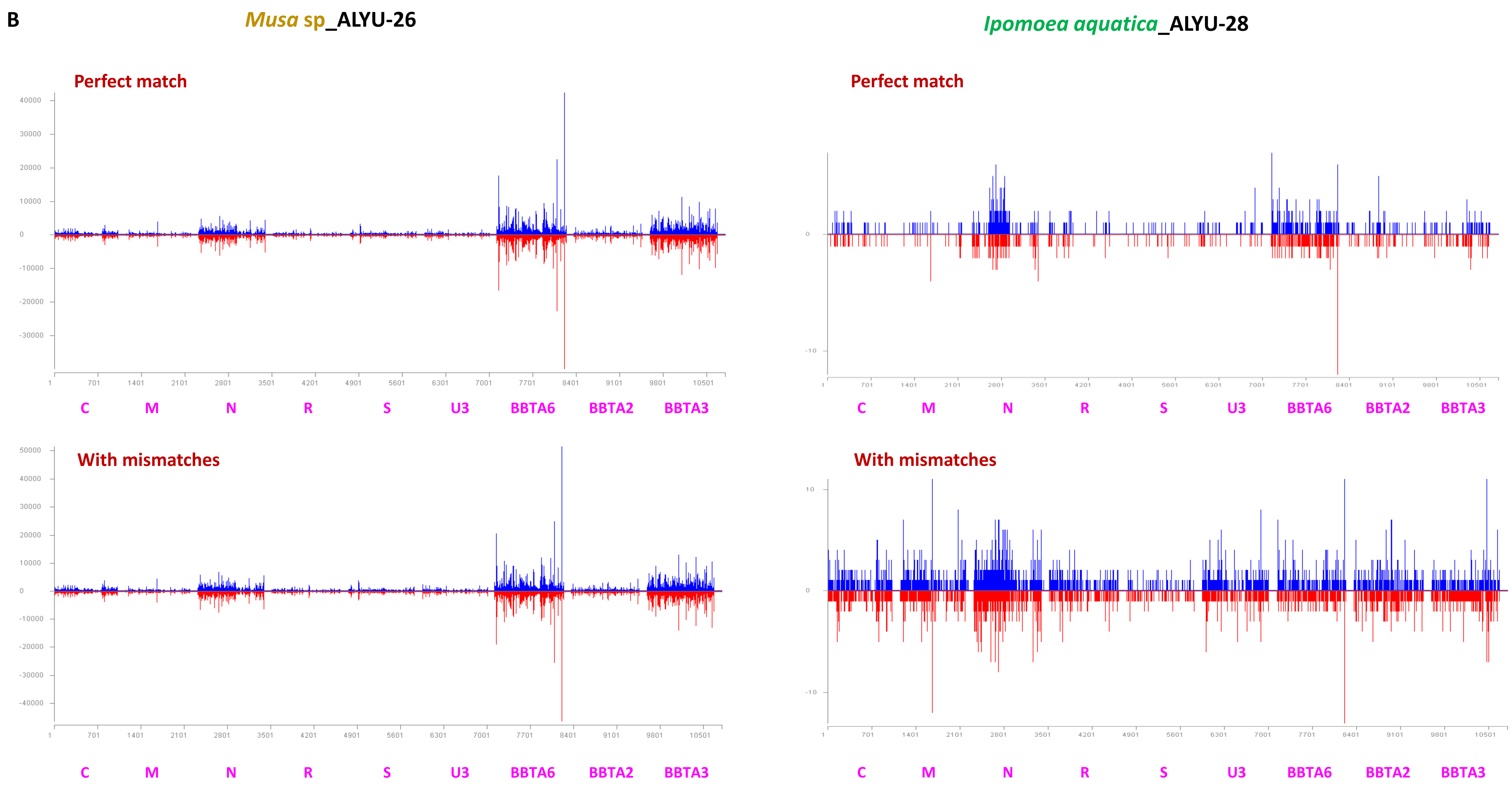
B
Musa sp_ALYU-26
Ipomoea aquatica_ALYU-28
Perfect match
40000
30000
20000
10000
0
-10000
-20000
-30000
1
701
1401
2101
2801
3501
4201
4901
5601
6301
7001
7701
8401
9101
9801
10501
C
M
N
R
S
U3
BBTA6
BBTA2
BBTA3
Perfect match
0
-10
With mismatches
50000
40000
30000
20000
10000
0
-10000
-20000
-30000
-40000
With mismatches
10
0
-10

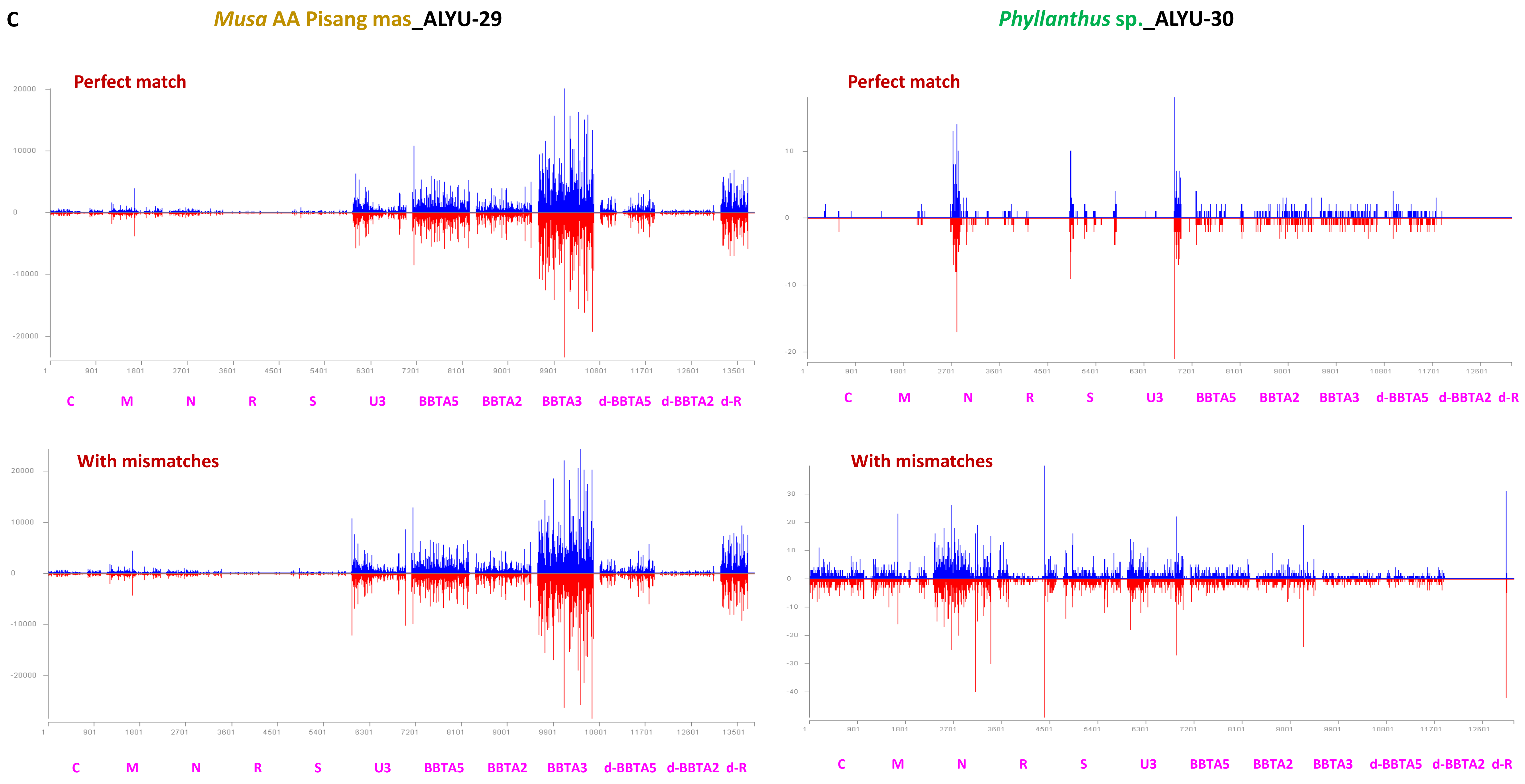
C
Musa AA Pisang mas_ALYU-29
Phyllanthus sp._ALYU-30
Perfect match
20000
10000
0
-10000
-20000
1
901
1801
2701
3601
4501
5401
6301
7201
8101
9001
9901
10801
11701
12601
13501
C M N R S U3 BBTA5 BBTA2 BBTA3 d-BBTA5 d-BBTA2 d-R
With mismatches
Perfect match
10
0
-10
-20
With mismatches
30
20
10
0
-10
-20
-30
-40

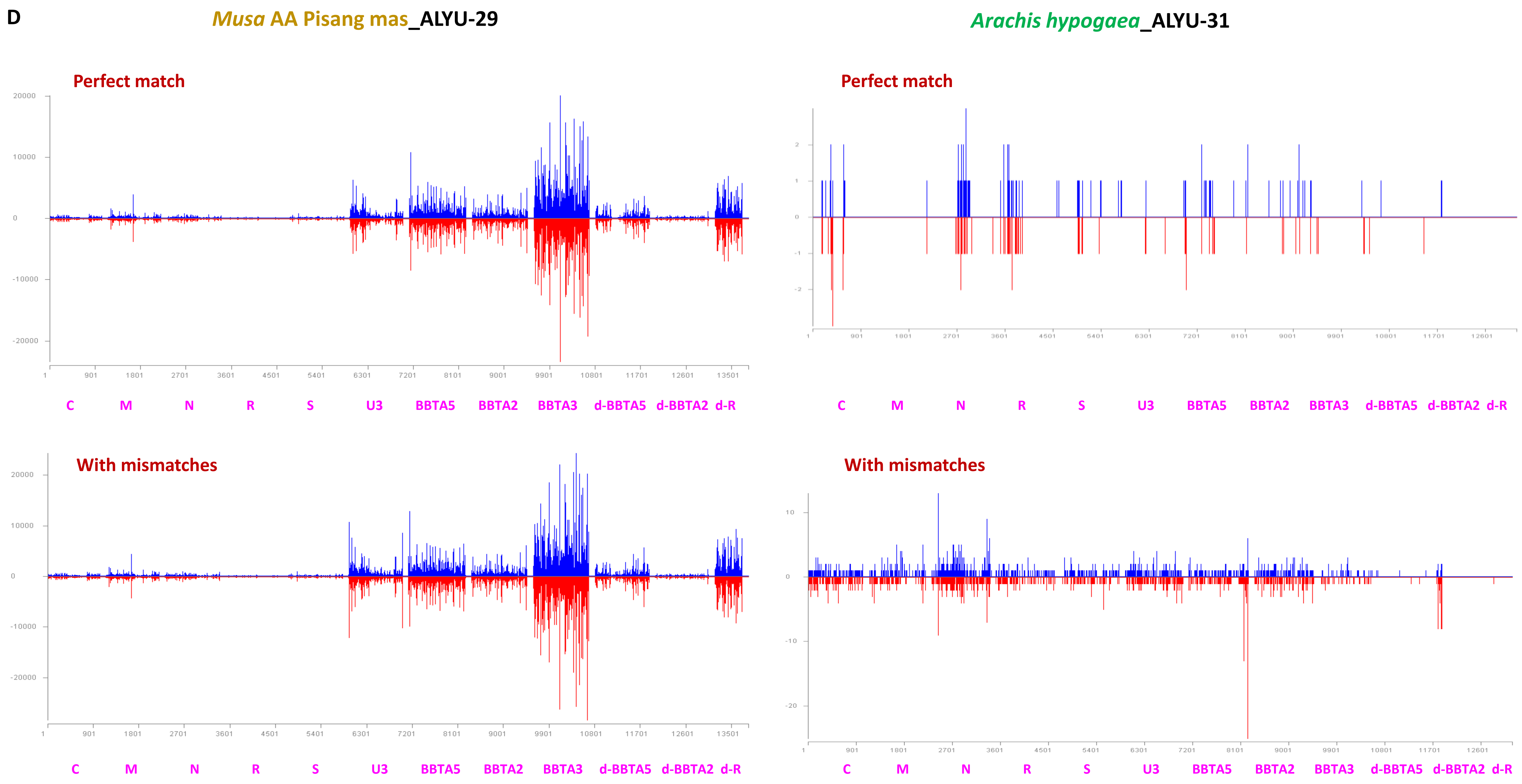
D
Musa AA Pisang mas_ALYU-29
Arachis hypogaea_ALYU-31
Perfect match
With mismatches
20000
10000
0
-10000
-20000
1
901
1801
2701
3601
4501
5401
6301
7201
8101
9001
9901
10801
11701
12601
13501
C
M
N
R
S
U3
BBTA5
BBTA2
BBTA3
d-BBTA5
d-BBTA2
d-R

E

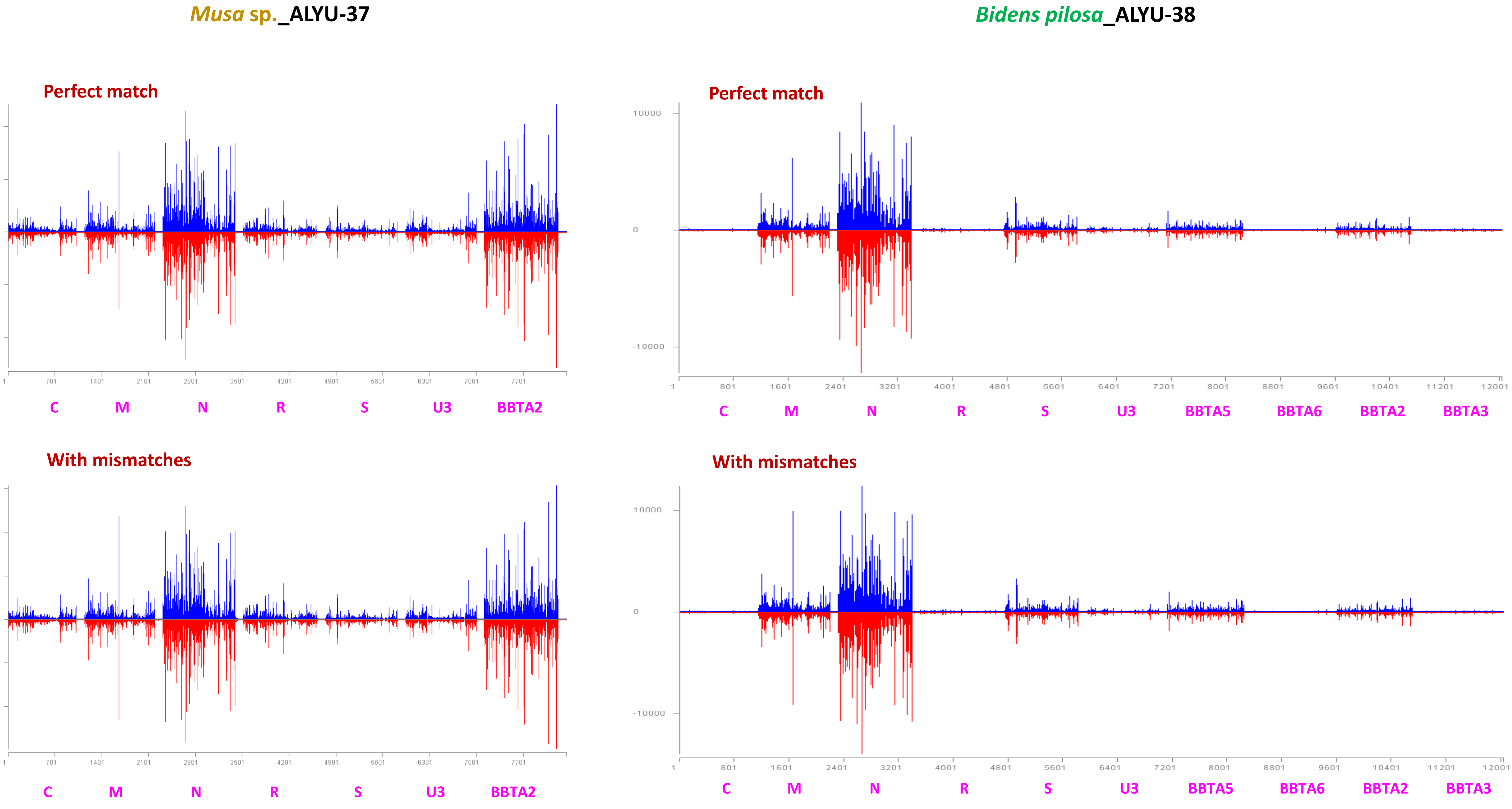
Musa sp._ALYU-37
Bidens pilosa_ALYU-38
Perfect match
20000
10000
0
-10000
-20000
1 701 1401 2101 2801 3501 4201 4901 5601 6301 7001 7701
C M N R S U3 BBTA2
Perfect match
10000
0
-10000
1 801 1601 2401 3201 4001 4801 5601 6401 7201 8001 8801 9601 10401 11201 12001
C M N R S U3 BBTA5 BBTA6 BBTA2 BBTA3
With mismatches
30000
20000
10000
0
-10000
-20000
1 701 1401 2101 2801 3501 4201 4901 5601 6301 7001 7701
C M N R S U3 BBTA2
With mismatches
10000
0
-10000
1 801 1601 2401 3201 4001 4801 5601 6401 7201 8001 8801 9601 10401 11201 12001
C M N R S U3 BBTA5 BBTA6 BBTA2 BBTA3

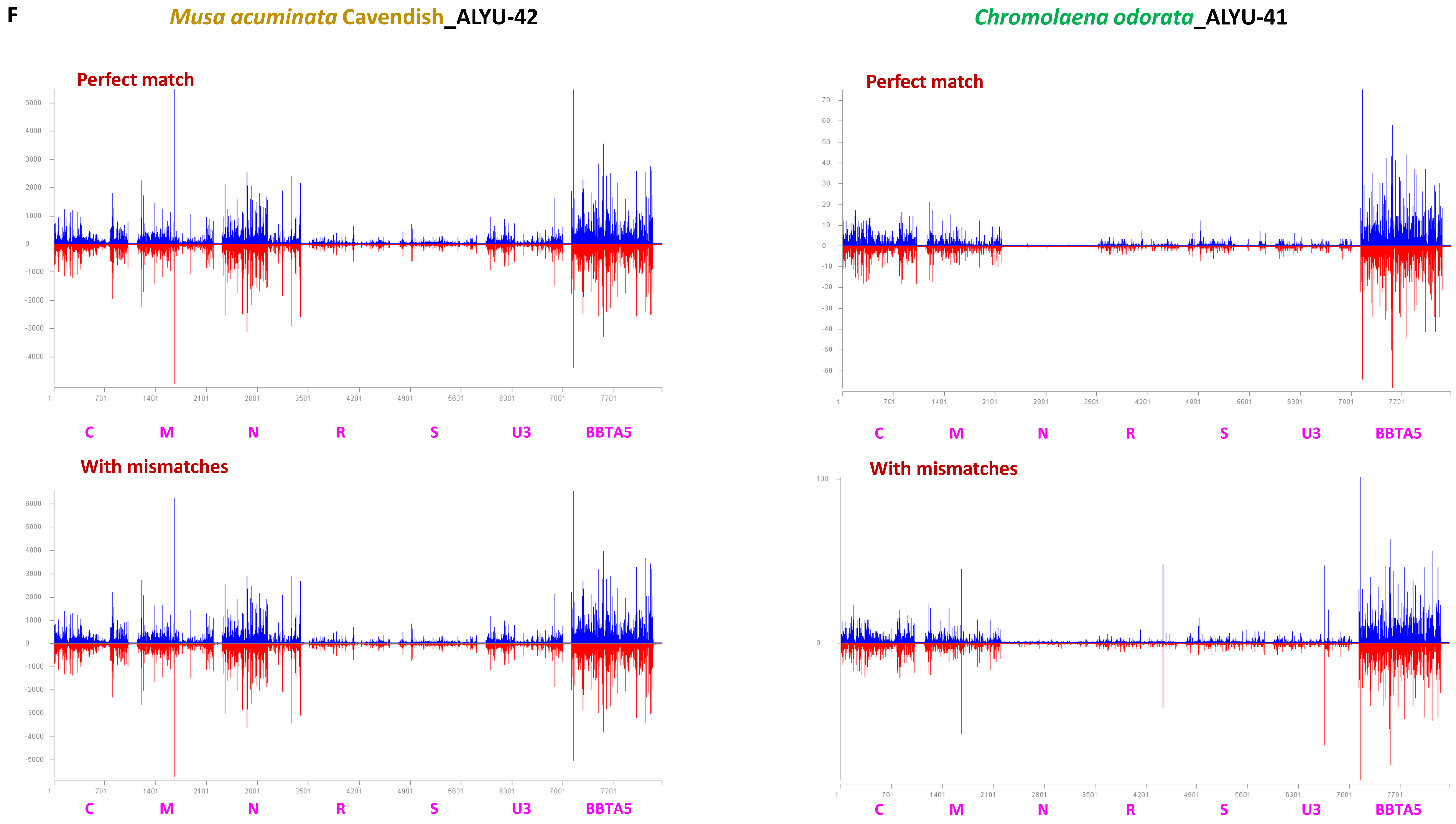
F
Musa acuminata Cavendish_ALYU-42
Chromolaena odorata_ALYU-41
Perfect match
With mismatches
Perfect match
With mismatches
5000
4000
3000
2000
1000
0
-1000
-2000
-3000
-4000
6000
-5000
70
60
50
40
30
20
10
-10
-20
-30
-40
-50
-60
100
1
701
1401
2101
2801
3501
4201
4901
5601
6301
7001
7701
C
M
N
R
S
U3
BBTA5

G

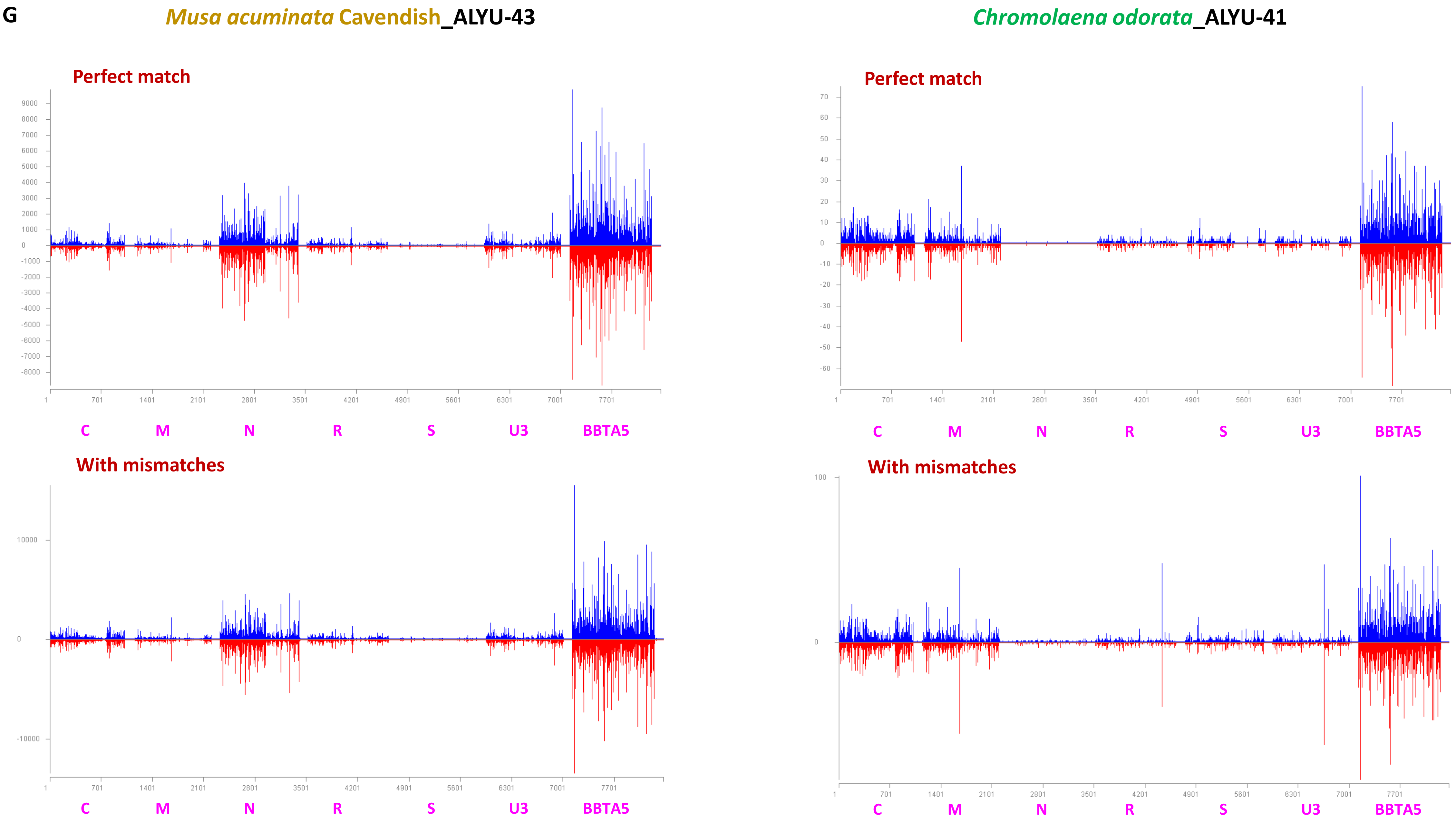

Musa acuminata Cavendish_ALYU-43
*Chromolaena odorata*_ALYU-41
Perfect match
9000
8000
7000
6000
5000
4000
3000
2000
1000
0
-1000
-2000
-3000
-4000
-5000
-6000
-7000
-8000
1
701
1401
2101
2801
3501
4201
4901
5601
6301
7001
7701
C
M
N
R
S
U3
BBTA5
With mismatches
10000
0
-10000
Perfect match
70
60
50
40
30
20
10
0
-10
-20
-30
-40
-50
-60
With mismatches
100

H

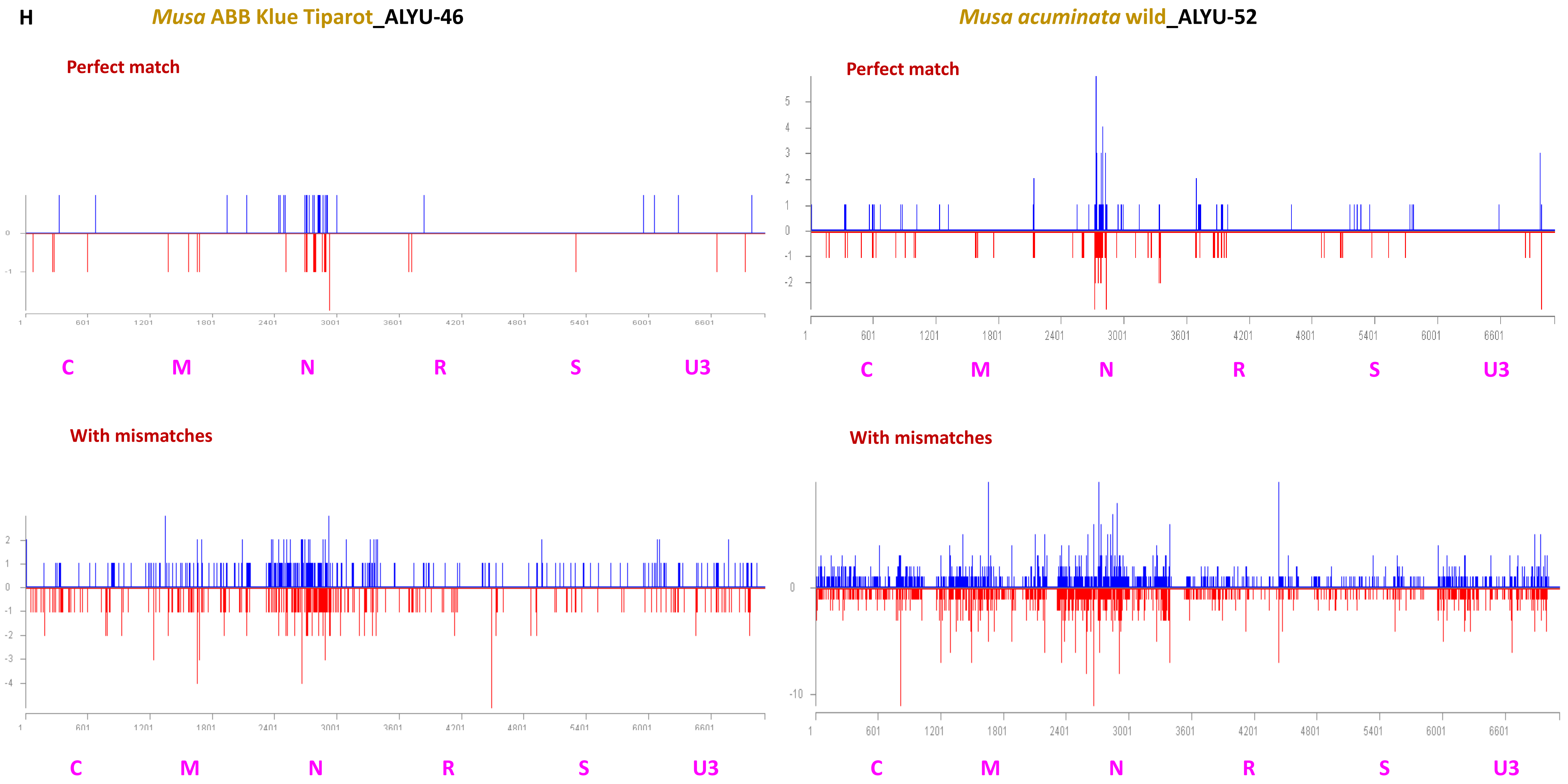
Musa ABB Klue Tiparot_ALYU-46
Perfect match
1 601 1201 1801 2401 3001 3601 4201 4801 5401 6001 6601
C M N R S U3
With mismatches
1 601 1201 1801 2401 3001 3601 4201 4801 5401 6001 6601
C M N R S U3
Musa acuminata wild_ALYU-52
Perfect match
1 601 1201 1801 2401 3001 3601 4201 4801 5401 6001 6601
C M N R S U3
With mismatches
1 601 1201 1801 2401 3001 3601 4201 4801 5401 6001 6601
C M N R S U3
