# Supplementary material for: Evidence for Dicot Plants as Alternative Hosts of Banana Bunchy Top Virus and Its Alphasatellites in South-East Asia
Source: Pathogens. 2023 Oct 28;12(11):1289. doi: 10.3390/pathogens12111289 (PMC10675457; doi:10.3390/pathogens12111289)
Supplement: Supplementary file 1 [file pathogens-12-01289-s001.zip › Supplementary Dataset S1.pdf]

Dataset S1. Supplementary sequence information. Virome components of banana and non-banana leaf samples from SEA (ALYU-25 to ALYU-56).

ALYU-25 - Musa itinerans - Vietnam

Virome
1. **BBTV** six components
2. **BBTA5** - new alphasatellite named here Banana bunchy top alphasatellite 5, genus *Banaphisatellite*
3. **BBTA2** - Banana bunchy top alphasatellite 2, genus *Muscarsatellite*
4. **Badnavirus** new species – named here Banana streak Musa itinerans virus

BBTA5
Best matches to MF510475 MF510474 73% coverage 66.6% identity Faba bean necrotic yellows virus associated alphasatellite 2; AJ132187 AJ005966 74% coverage 66.6-66.4% identity Faba bean necrotic yellows C9 alphasatellite; MK291270 72% coverage 67.1% identity Sophora yellow stunt alphasatellite 3

Rep protein best matches to QBF29185 99% coverage 63% identities 80% positives Sophora yellow stunt alphasatellite 3; ATU31571 99% coverage 62% identities 80% positive Faba bean necrotic yellows virus associated alphasatellite 2; NP_619574 100% coverage 62% identities 77% positives Faba bean necrotic yellows C9 alphasatellite

>BBTA5_alyu25
TATTACCCGACCTTGGCTCACTGGCTCACTATATAAGGCGGAGATGTCGCAGCAGCGAAATTGGGTATTCACGCGTAACTTCTCCGGCGACCCGCCGAATTTAAAGTTCGCCGGAGAAAC
ACAATACGCGTGTTGGCAACATGAGAAGAAGAATCACGACCATCTTCAGGGAGTGATTCAATTGAAGAAGAAGCTCCGGATGAACGCAGTGAAGACGTTAATTGGGGAAATCCACACCT
CGAAGCCATGAGAGGAACAATCGACGAAGCAATAAGGTACGTGACGAAAGAAGAGACACGAGTAGCAGGTCCGTGGGAATTCGGAGAATTACTACGAAAAGGATCTCATAAGAGGAAATT
GATGGAATTGCTCGACGATCCAGACAACGAAATAATGGAACCCCAAAAATATCGACGCGCCATTACTAAACAAGCAATGGACGCGTCAAAGAAGAAGGCGGAATTAGGGTTTCCTTACGA
CTTAAAGGAGTGGCAAAAGATGGTTATTGAGTTAATCGAAGAACAACCCGACAACAGAACTATTATCTGGGTCTATGGACCTAATGGTGGAGAAGGTAAAACGCAGTTCGCAAAGCATTA
TGGATTACTCAAAGGATGGACATATCTACCTGGTGGTGAACTGAAAGACATGATGTATCTGTTGGCAAAAGAAATTAATAATAACGTTATTATTGATTTCCCCCGATGTACAAAGGATTT
CATATCCTATAAGTTTATTGAGATGGTAAAGAACCGATGCATATTTAGTTATAAATATGAACCAATCGGGGCTATTGTAAGTAATGAAGTCCATGTAATTGTAATGTCGAATGAGCTTCC
AGATTATTCAAAAATTTCGGAAGACAGAATAAAATTAATATTTACATAACGCACACTATGACAAAAGGGCAAAAATGCAAAATCGGGGGTTGATTTGTCTATATTTACGAATAAGGGCCG
CAGGCCCGTACACAATGCGCGGGAAATTCAAAATCGGaCGCCAAATATTATTAAATACAATACACCGCCTTTTAGAAATCCGTGGACGAGAAGATGCCACGTAGCAAAACACTGTACTCC
TTGGCTCACTATAAATACCTGAGCCAAGGTCGGGCATAG

BBTA2
Best matches to EU430730 100% coverage 98% identity; MG545616 100% coverage 96% identity; NC_038953 100% coverage 96% identity

Rep protein best matches to AZL93963 100% coverage 99% identities (1 aa) 100% positives; ACB86656 100% coverage 99% identities (2 aa) 99% positives; YP_009508281 100% coverage 94% identities (11 gaps in middle) 94% positives

>BBTA2_alyu25
TATTACCCACCTCCGCGCACCACCTCCGCGCACCTATAAAATGTCTGCCTCCCGATGGACATTTACGCTTCATTATTCCGACGCAACGGAGCGAGGCAAATTCCTCGCGACTTTGAAGGA
GGAAGATGTGCACTACGCCGTCGTCGGCGACGAAACTGCTCCGAATACTGGTCGAAAACATCTTCAAGGATATCTCTCCTTGAAGAAACGTTTTCGTATTAGCGGAATAAAGAAGAAATA
TTCGTCGAGAGCGCATTGGGAGAAAGCTCGAGGATCAGACTACGACAACAAGGCGTACTGTTCCAAAGAAGCCCTAATTCTTGAATTAGGGGTTCCCTGTCAAACAGGTTCGAATAAGCG
TAAATTAGCAGATATGGTTACAAGATCTCCCGAACGAATGAAAATTGAACAGCCAGAGATCTTTCACCGATACGAATCGGTGAAGAAGATGAAAGAATTCAAAGAAAGGTATGTCTATCC
TATCCTCGATAGGCCATGGCAGGTACAATTAACGGAGTTAATTGAAGCAGAACCTGATGATCGAACGATCATCTGGGTATTTGGACCAAAAGGGAATGAAGGCAAATCAACGTATGCGAA
GTCATTAATCCAAAAGGATTGGTTCTACACAAGGGGAGGAAAGAAGGAGAACATATTGTTCGCCTACGTAGATGAAGGTTCGACAAAAAACGTTGTATTTGATCTTCCGCGTACTGTACA
AGAATTTATTAATTATGATGTAATCGAAGCACTGAAGGATAGAGTAATCGAGAGTACAAAATACAAGCCTGTGAAGTATTTAGAATTGAATACTGTACATGTAATAGTTATGGCTAATTT
TCTTCCTGATATGTGTAAAATATCTGAAGATCGAATAAAAATAGTTGCTTGCTGAACACGCTATGACAAACGCACGCTATGACAAAAGGGGAAAAAAGAAAAATCGGGGGTTGATTGGGC
TATCCTAACGATTAAGGGCCGCAGGCCCGTCAAGATGGATCCAATAACCCGATAAGAAGTTAAACGGGTCTAAAATACTTTCTTCGCCCGCAAGCAACCACTTTAACCTCTGCGCACCTA
TATATAGCGGAGGTGGGCTAG

DNA-C
41 SNPs to MF039864 KY427060 (**Thailand**). **44 SNPs** to MF039876 (**Thailand**). **49 SNPs** to MF039870 (**Thailand**) KM607098 (**China**)

C protein (161 aa): 4 aa to KM607098 KM607099 (**China**) MF039864 KY427060 (**Thailand**)

>C_alyu25
TATTACCCCCAGCGCTCAGGACGGGACATCACGTGCATCTAACAAATGCACGTGATATAGCAGTAGCTTGTAGCGAAAGATAACCATCACTATCAAAAAAGTAAGAGGAATATTCGTTGC
TTAGGGACTAAGCAACGGGTATAGATATTTGTTCGAGAAGCGACATTGGAGGCTATTTAAACCTGTTGGTTTTGTGATTTCCGGAATCACTCATCGGAGCAGAAATGGAGTTCTGGGAAT
CGTCTGCAATGCCAGACGATGTCAAGAGAGTGATCAAGGAAATATATTGGGAGCATCGAAACAAACTTCTGTTTTGTCAGAAGTTGAAAGGCTGTGTAAGAAGGATTCTGGAGCATGGAG
ATCAAGATGACGCTCTAAACGCAGTGAAGGAGATGAAGACTTCTATAATTCGCTATAGCGAATATTTGAAGAAACCTTGTGTGGTATTGTGTTTTAGTACTAATAAATCTATTGCATATA
GGTTAAACACAATGGTGTTCTTTTATCATGAATATATTGAAGACCTAGGTGGTGACTACTCGGTATATCAAGAGTTGTTCTGTGATGAGGAACTTCCTTCTTCGTCGACAGAAGAAGAAG
ATGAAGAAGTAATATACAGGAATGTTATCATGGCATCGACAGAAGAGAAGATCTCTTGGAGTGAATGTCAAAAGATAGTTATATCAGATTATGATGTAACATTACTGTAATGTAATAACC
ATTATCTTACATAAAATAATGGAATGATGATTATGTATTCTAGCATAAATACATAATGGTATACGTATAGCATAAAATACATATACCTACATACAACACGCTATGACAAACAGGGAAAAA
TGAATAATCGGGGGTTGATTGGGCTATCTTAACGATTAAGGGCCGAAGGCCCGTTTAAATATGTGGTGGACGAAGTCCACAACACATAAAAAGTCATCAGAACAATGGAATATAATGAGC
TGGCAACGTAGGGTCCATGTCCCGAGTTAGTGCGCCACGTGAGCGCTGGGGCTTAT

DNA-M
23-27 SNPs to MF039871 KY427061 MF039865 MF039876 (**Thailand**). **56 SNPs** to KM607239 (**China**)

M protein (117 aa): 0 aa to KC581796 (**Thailand**) **1 aa** to MF039871 MF039865 MF039877 (**Thailand**)

>M_alyu25
TATTACCCCCAGCGCTCAGGACGGGACATCACGTGCAACTAAGAAATGCACGTGACTGATATATTCTACATAACGGTTTATTGAACCGTTATATATTGTTGTAACGTAAAGTCACGTGTG
ATAGCGAAAGGCACGTGACTAAGTCAAATGTATTGAATAAACATTTGACGTCCGGTTGCTTCCTACGGAAGTCATGGTTGCTTCGTGGCGAAGCAAAACATTTATATATTGGCTTGGACT
GCTGCCTATAAATAGGCATGCAGGGAAATGGCATTAACAACAGAGCGAGTGAAACTATTCTTTGAATGGTTTCTGTTTATTGGTGCAATATTTATTGCGATAACAATATTATACATATTG
TTGGCGTTGCTCTTTGAGGTCCCCAAGTATATTAAGGATGTTGTGAGGTATCTTGTAGAATACCTGACCAGACGACGTGTATGGATGCAGAGAACGCAGTTGACGGAGGCAACAGGAGAT
GTTGAACTCGGCAGAGGTAGTGTGGAAGACAGACGGGATCAACAACCGGCTGTCATACCACATGCAACACAGGTTATCCCTTCGCATCAACCTAGAAGGGATGAGCAAGGAAGACGAGGA
AACGCCGGACCAATGTTTTAATACACGGTATTGTAATATACGAAATATAAATGGATAATGATATGTATTGTGAAACAAAATATATGTGAAACATAATATATATATGTTTGATAATTTACATA
TTGTAATATGTGAATTGTATACGAGTGGTGTATTTATAGAACATACAACACGCTATGACAAACAGGGAAAAATGAAGAATCGGGGGTTGATTGGTCTATCGTATCGCTTAAGGGCCGCAG
GCCCGTTGAAATGATTCTTTATAAAACAAATATACATGATACGGATTGTTGAAAATATAATCAACGATGTATAAATACAACAGAATGTTGTATACTAATTAAAATAATGAGAAGATAAGT
ATATTTGTGAAGGATAAGGATCACAACCACCACTTTAGTGGTGGGTCATATGTCCCGAGTTAGTGCGCCACGTAAGCGCTGGGGCTTAT

DNA-N
19-20 SNPs to MF039872 MF039878 (**Thailand**). **22 SNPs** to KM607387 KM607385 (**China**). **23 SNPs** to MF039866 KY427062 (**Thailand**). **32 SNPs** to KM607386 (**China**)

N protein (154 aa): 0 aa to MF039872 MF039878 KY427062 (**Thailand**) KM607387 KM607385 (**China**) **1 aa** to MF039866 (**Thailand**)

>N_alyu25
TATTACCCCCCGTGCTTGGGACGGGACATGACGTCAGCATAGATTATAATGGGCCAACTAAAGGCCCATTTAATAGAATAGGCGGGCTTTTGACATATTTCAAAGGCCCAGCCTGGAAGT
GGATAATGTCACGTGCCTAATAAGAGGTTGCTTCGCCTCGAAGCAATCCGAATAAATGTTGCGTATTCAATACGCAACTGAAGTCTATTAATATGGATGTCTCTGCCGAATAAATCAGAG
CGTAAGCGAAGCAGAAGCGATGGATTGGGCAGAATCACAATTCAAGACATGTACTCATGGGTGCGATTGGAAGACGATATCATCGGATTCATCCGAGAATCGACAATATGTACCTTGCGT
CGACGCTGGCAGCGGAAGAAAGTCACCTCGCAAGGTACTGCTAAGATCTATTGAAGCTGTGTTTAATGGAAGCTTCAAAGGAAATAACAGGAATGTTCGTGGGTTTATATACGTATCAAT

ACGAGACGATGATGGAGAAATGCGTCCAGTACTTATAATACCATTCGGAGGATATGGATATCATAATGATTTCTATTATTTCGAAGGGAAGGGGAAAGTTGAATGTGATATATCATCAGA
TTATGTAGCGCCAGGAGTCGATTGGAGCAGAGACATGGAAGTTAGTATTAGTAACAGCAACAACTGTAATGAATTATGTGATCTGAAGTGTTATGTTGTTTGTTCGTTAAGAATAAAGGA
ATAACAGATGTGCTGTAATGATTATTAATAAAACATATTTTCATGTAATTGATAGTTGTATAAAACATACAACACGCTATGACAAACAGGGAAAAATGAAAAATCGGGGGTTGATTGGTC
TATCGTATCGCTTAAGGGCCGCAGGCCCGTTGAAAAATAATAATCGAATTATAAACGTTTGATAATAATCAGAGATAGATGATAAGCATCCAAAAACATAGACGAAGTATATGGCTGTAT
AATATAAAAGAAGCATATAAATAAAATATGTAAACTAATCTCTGATTGGTTCAGAATGAAACCCCACCAACTTTATGTTGGTGGAAATGTCCCGATGACGTAAGCACGGGGGACTAT

DNA-R
16 SNPs to AF416475 (**Viet Nam: Hue region**). **17-21 SNPs** to KM607677 KM607676 KM607678 KM607679 (**China**). **19-23 SNPs** to MF039879 MF039873 MF039867 (**Thailand**)

R protein (286 aa): 0 aa to KM607676 KM607677 KM607679 (**China**) **1 aa** to AF416464 AB113660 AF416474 (**Vietnam**) MF039879 (**Thailand**) …

>R_alyu25
TATTACCCCCAGCGCTCGGGACGGGACATTTGCATCTATAAATAGACCTCCCCCCCCTCCACTACCAGATCATCATCGTCGACAGAAATGGCGCGATATGTGGTATGCTGGATGTTCACC
ATCAACAATCCCGCTTCACTACCAGTGATGCGGGATGAGTTCAAATATATGGTATATCAAGTGGAGAGGGGACAGGAGGGTACTCGTCATGTGCAGGGATACGTTGAGATGAAGAGACGA
AGTTCTCTGAAGCAGATGAGAGGCTTCTTCCCTGGCGCACACCTTGAGAAACGAAAGGGAAGCCAAGAAGAAGCACGGGCATACTGTATGAAGGAAGACACAAGAATCGAAGGTCCCTTC
GAGTTTGGTGCCTTTAAATTGTCATGTAATGATAATTTATTTGATGTCATACAGGATATGCGTGAAACGCACAAACGGCCTTTGGAGTATTTATATGAGTGTCCAAATACCTTCGATAGA
AGTAAGGATACATTATACAGAGTTCAAGCAGAGTTGAATAAAACGAAGGCGATGAATAGCTGGAAAACATCCTTCAGTTCGTGGACATCGGAAGTTGAAAATATAATGGCGGAGCCATGT
CACCGAAGGATAATTTGGGTCTATGGCCCAAATGGAGGAGAAGGAAAGACAACCTATGCAAAATATTTAATGAAGACGAAGAATGCGTTTTATTCGCCAGGAGGAAAATCATTGGATATA
TGTCGATTGTATAATTACGAGGAAATAGTTATATTTGATATTCCCAGATGCAAAGAGGAATATTTAAACTACGGTTTATTAGAAGAATTTAAAAATGGAATTATTCAAAGCGGGAAATAT
GAACCCGTTTTGAAAATTGTAGAATATGTGGAAGTCATTGTAATGGCTAACTTCCTTCCGAAGGAAGGAATCTTTTCTGAAGATCGAATAAAGCTAGTTGCTTGCTGAACACGCTATGAC
AATCGTACGCTATGACAAAAGGGGAAAAGCAAAGAATCGGGGGTTGATTGGTCTATCCTACCGACAAAGGGCCGCAGGCCCGTCAAGATGGACGGCTTGATCAGATGTCCCGAGTTAGTG
CGCCACGTAAGCGCTGGGGCTTAT

DNA-S
12/16 SNPs to AB113661/AB113662 (**Viet Nam**). **12 SNPs** to MF039874 (**Thailand**). **22-23 SNPs** to KM607535 KM607534 (**China**). **26 SNPs** to KY427064 (**Thailand**)

S protein (170 aa): 0 aa to AY337715 AY494786 HQ378191 (**China**) FJ787433 FJ787434 (**Philippines**) **1 aa** to several isolates

>S_alyu25
TATTACCCCCAGCGCTCGGGACGGGACATGGGCTAATGGATCATGGAATAAGGGCCCATCGGGCCCGTTAAGATGGGTTTTGGGCTTATGGGCTTTATCCAGAAGACCAAAAACAGGCGG
GAACCGTCCCAATTTCAAATTTAGATAGCTTGCCCCGCAAGCAAACTAAAAGTCTATATATACCAGTGTAGATATATTGTTCAGAGAACAAATGGCGAGGTTTCCGAAGAAATCAATCAA
GAAGAGGCGGGTTGGGCGACGGAAGTATGGCAGCAAGGCGGCAACCAGTCACGACTACTCGTCGTTAGGTTCAATATTGGTTCCTGAAAATACCGTCAAGGTATTTAGGATTGAGCCTAC
TGATAAAACATTACCCAGATATTTTATCTGGAAAATGTTTATGCTTTTGGTGTGTAAGGTGAAGCCCGGAAGAATACTTCACTGGGCTATGATTAAAAGTTCATGGGAAATCAACCAGCC
GACTACATGTCTGGAAGCACCAGGTTTATTTATAAAACCTGAACATAGCCATCTGGTGAAACTGGTATGCAGTGGGGAACTTGAAGCCGGAGTCGCAACAGGGACATCAGATGTTGAATG
TCTTCTAAGGAAGACAACCGTGTTGAGGAAGAATGTAACAGAGGTGGATTACTTGTATTTGGCATTTTATTGTAGTGCTGGAGTTAGTATTAACTACCAGAACAGAATTACATATCATGT
ATGATATGGTTATGTAAACATAAACCTTTGTATGGAATAATGACCAAATAACATACAAGACGCTATGAAATACAACACGCTATGACAAACAGGGAAAAATGAAGAATCGGGGGTTGATTG
GTCTATCGTATCGCTTAAGGGCCGCAGGCCCGTTGAAATGATTCTTTATAAAACAAATATACATGATATGGATTGTTGAAAATATAATCAACGATGTATAAATACAACAGAATGTTGTAT
ACTAATTAAAAAAATGAGAAGAAAAGAATATTTGTGAAGGATAAGCATCAGAACCACCACTTTAGTGGTGGGTCATATGTCCCGAGTTAGTGCGCCACGTAAGCGCTGGGGCTTAT

DNA-U3
41-43 SNPs to KY427065 MF039869 (**Thailand**)

U3 protein (39 aa): an identical protein encoded by KY427065 (**Thailand**); N and C termini similar to parts of a putative protein encoded by two BBTV isolates AF102781 (**Egypt**) and U18078 (**Hawaii**); C-terminal amino acids 19-38 similar to a motif in DNA-directed RNA polymerase I subunit RPA12 isoform X2 [Bombyx mandarina] (120 aa) and transcription-associated zinc ribbon protein [Bombyx mori] (120 aa)

>U3_alyu25
TATTACCCCCAGCCCCCAGGACGGGACATGGGCTTTTTAA**ATG**GGCCGAGAGAGTTTGAACAGTTCAGTATCTTCGTTATTGGGCCATCTGGCCCAATAATTAAGAGAACGTGTTCAAAA
TCGGGGTTTGACCGAAGGTCAAGG**TAG**ACGGTCAACAATATTCTGGCTTGCGGAGCAAGCAACACGAATTAAATATTTAATTCGTAAGACACGTGGACGGACCGAAATACTCCTGTATCT
CTATAAATAGCTTAAATCTGGCTTGGATAATTGCTCTCGCTCTTCTGTCAAAGGTGTTGTGTTGAGGCGGAAGATCGCCATCGGCGATCATCGGACGAAAAGCTGCAAGAGAGACGGAGA
ACCATGCTACGAAGCGTATATCGGGTATTTATAGACTTCTAGCGCAGCTAGAAGTATCCTTGTACTTGATATTGTATTTTGTAAATTACGAAGAAATTCGTATATTGATAATAATAAAAC
ATCTGGGACTGTTAATGTTTACATTAACCAGTATTAATAATGTATAATAACAAATATTGTATGAGGAACGAATACAATAATATTAAAATATGTGGAGCGTAGCGTGATAAACAGGTGTTT
AAGGTATAATTAAATTAATTATGCAATGTCATGATAATACGGTATAAGTTGAAGTATGAGGTGAAAGAGGAGATATTAGAATATTAAAAACCCAATTATATTATTTATAATACATACAAC
ACGCTATGACAAACGGGGAAAAAAGAAAAATCGGGGGTTGATTGGTCTATCCTAACGATTAAGGGCCGCAGGCCCGTCAAGATGGATGAACGGTTAGATTTGATTGCTTAGCCACGAAGA
AAGGAATCGTTTTGGGACCACTGACAAGACAGCTGTCACTACTTTTAAAAAATAATATAATAACTAATTGACGATAGTACCCCTCCCAAAGATGACGTATAGGGGTGTCCCGAGGTAATT
TAACATAGCTCTGACAAGAGATAGTGGACGTTGGATGCCACGATCGGACGATCGTGGTTTGATGAACTAATGATGACGTAGGGGCTGGGGCTTAT

Badnavirus a novel species, named **Banana streak Musa itinerans virus**
similar to KJ013510 Banana streak VN virus 100% coverage, 76.9% Identity, Gaps: 560/8084 (6.9%), AY750155 Banana streak VN virus Banana streak virus Acuminata Vietnam 100% coverage 76.9% Identity 7.1% Gaps: 574/8093

ORF I protein (CTG start codon) similar to AHM92958 *Banana streak VN virus* 98% identity (4 aa) 100% positives; YP_605809 Banana streak virus Acuminata Yunnan 93% identity (4 aa), 97% positives

ORF II protein similar to AHM92958 YP_233109 Banana streak virus Acuminata Vietnam 76% identity 87% positives

ORF III polyprotein similar to AHM92960 81% Identity 89% Positives Gaps: 40/1934 - 26 aa insertion at position 1010, 8 aa insertion at 1067; YP_605811 Banana streak virus Acuminata Yunnan 82% Identity 89% Positives 2% Gaps: 39/1935

SNPs: **ins-C485AC** (1589/165) **G623A** (1374/617) **C661T** (953/1226) **G958A** (1327/477) **G1134A** (1248/645) **A2177G** (1661/437) **C2219T** (1295/692) **G2296A** (1474/533) **G2546A** (1100/589) **C2575A** (1486/367) **A2756G** (709/1174) **G2897A** (1264/485) **G3107A** (1694/196) **G4250A** (1715/889) **A4534G** (1335/867) **A4601G** (502/1680) **A4622G** (1287/833) **G4646A** (519/1649) **C5855T** (1206/520) **G6197A** (859/307) **G6311A** (1304/512) **T6809C** (1919/458) **G7110A** (866/1279) **G7293A** (1327/835) **T7730C** (1134/432)

T661C G2756A G4601A A4646G - all silent substitutions; **A7110G** - amino acid substitution (T1836A) in ORF III

>Badna_alyu25
TGGTATCAGAGCAAGGTTCTCTGTAAGTAATTTATGGCTTTT**ATG**GGG**TAA**AACCCTTAGATAGGAGCCTGAGGGCTCTGCTATGCTTTATTTTGGAAAATAGCTGTGTAAGTAGTGAAT
GCATGATAAATCGAATGAGTTCTTCTGAAAGTCCCGAACTTATGAAATCATCCTTTTATAACAAGAGGGAAACCTTTGGTGTTTCCAAGTTCTACTGAAAAATTGTTTGGCCTAGAGAAA
CTGGTAGTACTATCAAAGGGAAGAAAAAGAAATTCAAGAGTTGGGTTTTAGGGAATCATCTAGCTAAGAGGCAAAAGAACCGGAACCTTACCTGGCAAGTAAGACCCGCGGAAGGGCAAT
CAGCTGGAGAACCCCGGTGTGAGAATCTGGCTGGATGAACGCCTATATCCACTTGAGAAGGAATGAAGTAATCCCTGAAAATAGTACTATTAGCTATTGAAGCTGGCTTTTAATCACAAA
AAAACTTGAGTTGTGCAAGGCAGAACTCTGGTATAACTGGATGACTAATAATTAGGGATCTCTATCTAGAACTCAGTAAGACGTAAGTGCATAAGCTACCTCTGGAATTGGCTACAATCC
TTGATAAGCATAAGTATCCCGAGGGTTACTCCTATCTGTCTTAAGACCATAAGAAAGCCCcTTTAAACT**CTG**GATTCCTACTGGGATCTCGAATTCGAAAAATATAAAAACTCTCATTCC
AAGTCTGTTTCTGATCTTTCCTACTTAGATCTAGCCACTGCTGAAAAGGTCTCTAATAAGGACCTCGCATTTAATTTGCATATAAACACCTACCGTAGTGATCTCGGTTTTAAAGTTGCA
ATTCACTCTTTATCAAAGAATCGTGAGCTGCTAATCCAGAACAGGAAGCTCTTGGAAGAGCAAAAGCAGCAGCTTTCTGAAATTAACAACCTATCTAAGGTTGTACGTCTCCAACGTGCT
GATTTAAAGGAGACCCTGAGGAAGCAGGACGTCCTTGCGAAGGAGCTTCAAGCTCTTCGTAAAGACTATCTGGAGAGGCGCCCTCTCAGCAAGGAGGACGTGGAGGAACTAGTTGTTCGC
ATCTCGGAACAACCCAAGTTCATTGAGAAACAAACTGAAGCTCTTACTGAGGAGCTTACGAAGGAAGTCCAGGCACTAAAGGTGATCATCCATAACTTCGAGCAGAAGTTACTGGGATGA
GCCTAGCTGGAAGCAAGGCCACAGCAATCTATCAAGAGGCTTTGCAATCTACAACTGCTGGCTGGGAAGACACCGGCATAGGATTTACTGATAGAGGAGTCATCAGTACAACGACTCTGT
CAAGACAGCTAAACTCGCTGTTATTTCTGGTGGTAAAACAAAATCAACAAATCACCACCCTCTCTGAAGAATTGGCCCAAGTCCACAATCGGGTCAAAATTATTGAAGGAGGAATAGGGG
AACCATCCACCCCCTTAATCAGAAGTGAAGTAGAGGCTCTCAACACCAAAATCCAAAATATTCAGAGCATCCAAAGAAGTCAACCTTCTAAGGAAACTTCTTCTGGGAAGACCAAGGTGT
TCGAGGATCCTTACAAAATCCTCCGACAACTCTAAATGGCGACAAGACCCCGAGTTTCGGGAGGCACTACCAGAACTCTGGTAGCTGAGCCAGGGACACCCCTGGTAGATGATCAGATCC
GGGAATACCGGACGGCGGCCCGAGCCGCATATGAAGCCCAAAGAATGGCCAGACGAACTGGCAATATTATTGGGAGAATAGTTGGACGACAGCCAAGGGAGCATACGCTGTCATTATTGG
TGGATCCAAACAGTGAGCTGGAGCGCTCACTAGCTCACCGGGCGAGAACAATTCCTGCTGAGGTGTTATACATGACCCAGCGCGGTGAGCCTACAAATCGGGTCTACAGAAACCGTACTG
AAGAAAGAATGCTGGTCACACAAGGGCAGCAAGACAGAACTATGATCATGCCCGAATCATATGATGCATTAAGAGAAGCTGGATACGAGTACATCCATCTCGGAGTTCTACAAGTCCGAA

TCCAGATTATGCATCGGACTTTCGCTGGTACAATGGCGCTGGTGGTCTTCCGTGACACCAGATGGACGCAAGAAAACAACCAGGACAGATCAATTATTGCGGCAATGGAAGCTGACCTGT
CACAGGGACATCAGCTAATATATGTGATCCCGGATATCATGATGACAATAAGGGATTTCTACCAGCATATACAGATTAGTATCCTGACCAAGGGATACGAGGGATTCCAAGGAGAAGCCA
ATCTCCTTGTTACAAGAAGCTGTAGATGCAGACTCAGCAATATACCAAATGTCGGATTTCAATACAATATCCAAAACGTGGTGGAGTTTCTAAAGTCCAAAGGTGTTAAAGCCTTAAATG
CCACGAAGCTGAGCACTAGGAGGTTCCAGGGAGGAAGCTGGAACATCCGACCATCGGAGGTGGTGGTCCCTATGCAGCCCACTCGGATGATAACCCGAGTAAATTATGATTCCTCCAGAA
GCATCTGCTTTGGAGACTATGAGGCGAGCACTTCAACGGCCCCCCCCAAATATTCTACTGATGGAGATGAGGACGAGTCTCTGGGGGAGATTCATCAAGTTAATATGTTCACATTCGTTG
AAGACAACTCTGAAGATGACTACCCACAGTTATCAGCTTTAGAGCGAATAATCGCTCCAGAAAGCATGGTGGGAGGAGAAGATGTGATTGCCGAGTTTTTAGGCAATCTATCTTTaGACA
CATCGGATGAAGACGAGGAATTTTATGACACTTTTGATAGACCTCAGTCCGATGAAGAATACAACGGGGACGGAGAATCTGAAGCAAGTACCCCAAGGAGCAAGTATGATATATTTACGT
TTGAAGACGATTACCCGAAGTTGCAACAATTAGAAAGTCTGGTTCTATCTACAAATGAATCAGCGATCAGCAACTACAGGCCAGCCGACACTGACATGATCGGAGTGGCACCTGGATATG
CACCAGCCACTGGAGCAACAACAAGTGGTGCAGGACCTTCTGATTTCCCTTATCCAAGAAGGCCAAGGAAATGGGAAAGCAATTCTGAATGGTTCAACCTCCCAACGGCAAACGCAAGGC
AAGCCTCTATCTTTGTCATGCCACAAGATTTTGACACAAAGGTATTCGAAAGATGGGAGAGTTCCGTGATACTACATCTGACGGATAAAATATTTGATGATCCGCAAGACAAGCTGACGT
ATATTGAAAATCTTCTTGGAGAATCTGAGAAGAAAATGTTTATTACATGGCGGATGAAGTACGCAGATGAATATGAAGTCATGAAGACGCAAGCCCTAGGATCAAATGGGACACAGAACA
TCCTGAACCAGATCAGGATGATATTCTATTTGGAAAATCCGCAAGTCGGGACCACTGACAGCCAGGACGCAGCATACAAAACTTTGAAACAACTGGTATGCACTGAGATGTCCGGCCAAG
CTATTTATCGTTATTTAAATGATTACTTCCATCTATCTGCAAAATCTGGAAGAGCTTGGGCGTCAGAGGAATTATCTAAGGAATTCTTCACTAAATTACCACGAGGATTAGGTGATAGAG
TGGAGAAGAAATTTAAGGAAAGGTACCCCCACAACACAATAGGGGTAGCCCCGAGAATCACCTTCACGAGAAACTATATGAAGGAAATATGCCAAGAAGCGGTGTTTCAGAGCCAACTGA
AGAAATTGGACTTCTGCAGAAGTACTCCAGTTCATGGGCTCTATGGCAAGGACAAGGCATATGGGAGAAAATATGGGGTCAGGAAGAGCACCTCATACAAAGGAAAACCTCACAAGTCTC
ACGTGAGGATAGACAAAAAGAAGCATTTAACAATGAAAAGGAAAGACTGCAAATGCTTCGCATGTGGAGACATTGGGCACTTTGCATCAGAATGCCCCAACCCAAAGAAACTTATGCACC
GAGTGCAAATCTTGCAATCTTTGGAGTTAGAAGATGGAATAGATGTGATTTCCGTTGGATTTGACGAATCGGACATATCAGACATCTATTCAGTATCTGAAGGAGAGGATAACTACCGGT
TCAACAATGAAGACTTTGAGGTAGTCAACCATGATCTCTTTATGTTCACTATTGAGGAAAGAAATCGCTTGGTTACAACTTCAGCACCATGGAGATGCGCTATGAGGGTAACTGCAAAAG
AAGCACAGTGCAAGCATGACTGGAGCTTCACAGAAAGCAAACAGGAAAGGTGCAGAGCCTGTAACAATGAAGCCCTGAAGGAAAGTAGGGCAGACTGCAAAGAATGTGGCTTAGTCATCT
GTGCCATATGCAAGCCATATTATTACAAAGGAGACGCACCAATCCCTGCACAAAAGAAAAAGGATATAACAAAGGAGCATGATTGGTTTGAGACAGCTCAGCTATTTATGATAAGAAAGC
AAAAGGCAGAAGCCAAAAGCAGAGAGCTGACTGAAGAGCTGGAAACAGCTTTGGAGCAGATTGCCTTATACAAAGAAAAAGAGCAAAAGGAAAAGGAAAATGCAACAACTACAGCTGAAG
AATATCAAAAAAGAATCAGATCATTGGAAGTAGAGAAAGAaCTCTACAAAGGATTAGAAAAAGAGACTGACAAAGAGTACATCCTgTACAGAATCAAGGCAAAAAAGGAAAAGGAAGCCT
TGAGACAAGAAATGGCGAGCGCAGCACAAGAAGAAATAATAGCGCTGAAACAAAGGATGACAGATGAAAGAGAAGAAATGATGAGAAAAATACAGTCTCTGGAAGAAGAAAAACAAGCCT
TAAAGGAGGAAAATGAGAAGCTTGGAACTACAAACAGCACCCTCCTAGAAGAGATGGCAGCCCTCACAAAGGAAAATGAGGATATGAAAGCAGCAAGGGTTATAGAAGAACCCAAGGAAG
TAGAAATGGTAAATAATATTGAGGAGCACTTGGTTCTAACTGGGCAGCAAAAGAACAATCTCCTAAACATCAAAGTTACCATTGAAATCAAGGAAAAGAAGATCACAATGAATGCGATCT
TGGACACAGGAGCAGCTATCTGTGTCTGCGACGGCCAGATGATCAATGAATACTTTAGGCGGCCATCACTGATGAACGCCTTCATCAAGGGCGTTAATGGAATTACTAATGTCAGTGAGA
TCCTTGAAGAAGGAAAGATCTGGATTGGGAACCAATGGTTCAGGATTCCCAGAACATACGTCATGCCTCAATTATCCGAAGGTCTTCACTTCATCATAGGCATGAATTTTATAAGAGCCA
TGGAAGGAGGCATCAGGATTGAACAAGGGCTGGTGACTTTCTACAAAATGGTAACGCAAACCCCAGCACCCCCTATAACTCATGAAATTTCATATATTGAAGAACTGGAATTAGAATTGC
CGCTTTATTATGATATATGTGCAACTAATCCAGTAAATGGAATGATCAATAATGAGCTGATCTCCCCCAAAGAAATACAGCAATTGAAGGATTTAGGCTATATTGGGGAAGAGCCATTAA
AACATTGGGCTAAAAACAGGGTCAAATGCAAAATTGAAGTGAAGAACCCTGACTTGGTCATTGAAGATCGGCCCCTGAAACACGTGACGCCTGCAATGAAGGAATCTATGAAGAGGCATG
TGGACAAGTTGCTGGAACTGAAAGTGATTAGACCATCAACCAGCAAACACAGAACCACCGCTATAATAGTTCAATCTGGAACAGAGGTGGATCCATTAACTGGAAAGGAGAAAAGAGGCA
AGGAAAGATTAGTATTCAACTATAAACGCCTCAACGACAATACAGAAAAAGACCAGTACTCACTGCCAGGGATAAATACGATTATCAGCAGAATCGGCAAGTCAAAGATTTACAGCAAGT
TTGACTTGAAATCTGGCTTTCACCAGGTAGCCATGGACCAGGAAAGCATCCCATGGACGGCCTTTTGGGCCATAGATGGACTTTATGAATGGCTTGTCATGCCATTCGGGCTAAAGAATG
CGCCGGCCATATTTCAGAGAAAAATGGACAACTGCTTCCGAGGAACAGAGGAGTATATAGCAGTTTATATTGATGATATTCTGATATTCTCTGACAATATTTCTGATCACAGGAAACATT
TAGCTAAATTCCTGAAAATTTGCAAGGAAAATGGGCTGGTTTTGAGCCCAACCAAAATGAAGATAGGCGCTAAAGAGATAGATTTTCTGGGAGCTACGATTGGAAACTCGAGAATCAGGC
TCCAGCCCCACATAATCAAGAAGATTATTGAAACAAAGGATGAAGAGTTAAAGGAGACAAAGGGACTCAGGAAATGGCTAGGAGTCCTTAATTATGCAAGGGCATACATCCCGAATTTGG
GCAAGACGCTTGGCCCACTGTACTCCAAGACATCAATTAATGGAGAGAAGAGGATGAACAACCAGGACTGGAAGGTTGTTCAAACAATCAAAAGTCAGGTACAAAACTTACCTGATCTTG
ACATACCCCCTGCAGAAGCTACTATGATTCTAGAAACTGACGGGTGTATGGAAGGATGGGGAGGAGTCTGTAAGTGGAAAGCTCACCCCTCAGATACAAGACTGGCGGAAAAGGTCTGTG
CATACGCCAGTGGGAGGTATAATCCTATCAAAAGCACAATCGATGCAGAAGTACACGCAGTAATCAACAGTCTGGAGAAGTTCAAGATTTACTACCTAGACAAGAAAGAACTGATCATCA
GGACGGATAGCCAGGCAATTGTTGCGTTCTACAAGAAACAGGCTGATCATAAGCCCTCAAGGACAAGGTGGCTTATGCTGATAGATTATATCACCGGCCTTGGAATTAACGTCAAATTTG
AGCATATTGACGGGAAGGAGAATGTACTAGCAGATACTCTATCAAGACTGGTACAAGTTCTGATCACAAAAATACACCACCCAGCGGAGACCCAACTGGTGGACGCCGTCATGGAGGTCA
TGAGCAATCCCAAGAAGGAGGCCTTAGCCAAGGTGAATCATTTTATCATGCTAACCCAACAATGGATTGCAGGACACCAAGAGGAACAAGGAGTGAACTCACTGCTCCATCTGGAAGAAC
CACAACTATGCTGCGGCTGTAAAGATCCTgCGACAGGAGGAAGAAGGAACGCAATCCTTCTGCAAAGCCACACGTCAGCCAATCCCTACCGATGGTTCTACAAGTGTGCGAAGGACAAAT
GTCACACTTGGATTTGGAAAGATATCCTGGACCAGTATGCTGAAGACTATGCAGCGTATACCAGGATTGGGCTCGAAGCCCTCAGCTTAAGAGACTGGTTTGAGGAACCAGCCCCAGATC
CACCTGACCCAGTGGACCGCCAGAGAATAGAAGATATCCTGGACTTACAAGACGTCAGCAATGACGATTGAAAGATTCCCGGAGACATCGGCGGAAGTGGTGGACCCAGTCTAGGTGCGA
TGCTTAGTCACGCACGATGACTTTGTCGGGAGCATCTTTGCTTTCGGCTAGCTTTACTAATACTTTAAGAAAAGTATTGTACAAGTTAGGTGCAGAATCATGTTGCGCACCCAGCTTTAG
TCTTGTCTACTGATTAGTGTGTCGGTTGCATTATTGGATGCCTGCGTGCACCCTAAGCAATCTCAGACCCTCTTCGC**TATAAG**AGGAGTCCTTGTATTCAGTTGCAAG**CA**TGCAAGTTTC
CCACTGCAAGCTTACTTCTGAGAAAGATTTAAGCAATAAAATTCCAATTTCCCCTTACATTAGTGTTCTTGAAAGGTTCGATTGAATCGAGCGAGTAGAGAGCAAGATCTTTTGGGATTT
CCGCACCTACA

ALYU-26 - Musa sp. - Vietnam

Virome

1. **BBTV** six components
2. **BBTA6** alyu26=27=38v2 – new alphasatellite named here Banana bunchy top alphasatellite 6, genus *Banaphisatellite*
3. **BBTA2** alyu26=27 – Banana bunchy top alphasatellite 2, genus *Muscarsatellite*
4. **BBTA3** alyu26=27=38 – Banana bunchy top alphasatellite 3, genus *Muscarsatellite*

BBTA6

Best matches to MF510475 MF510474 75% coverage 66.6% identity Faba bean necrotic yellows virus associated alphasatellite 2; AJ005966 AJ132187 74% coverage 66.6-66.3% identity Faba bean necrotic yellows C9 alphasatellite; KX534406 75% coverage 63.9% identity Sophora alopecuroides yellow stunt alphasatellite 3 isolate

Rep protein best matches to ARI50297 100% coverage 61% identities 77% positives Sophora alopecuroides yellow stunt alphasatellite 3; P0CK61 100% coverage 60% identities 77% positives Faba bean necrotic yellows C9 alphasatellite; ATU31571 100% coverage 59% identities 77% positives Faba bean necrotic yellows virus associated alphasatellite 2

>BBTA6_alyu26
TATTACCCGACTTTGGCGCAGGGCGCACTATAAAAGATGTCTGTGAGAAATTGGGTCTTCACACGCAATTTCGAAGGCGAAAGACCAGAGCTCCTGTTCGGCGGCGAAACTCAATACGCC
GCTTGGCAACATGAAAGAAAGAATCACGACCACCTACAGGGTGTGATTCAGTTGAAGAAGAAGACCCGACTGAACGCAGTGAAGAGCATAATCGGTGGAAATCCACATGTCGAACCCATG
CGAGCGTCTTTCGACGATGCCCTAGCATACGTCATGAAAGACGAAACCCGAATCGATGGTCCATGGGAATTCGGTATTCGAATCCGTCAGGGTTCGAATAAACGAAAGCTGTTAGAAATA
CTGGAGGATTCGGACAACGAAGTCCTTCAACCCCAAAAATACAGACGAGCCATGGCCAAACAGGCCATGAGTGAGTCTAAGAAGAAAGCTGCAGAAGAAGGATTTAGTTATGAACTGCGA
GAGTGGCAAGTGCAATTGACGAAGTTGTTGGAAGAAGAACCCGATAACAGAACAATAATTTGGGTATATGGACCAAAGGGTGGAGAAGGTAAAACAGAGTTCGCTAAACATTTAGGGTTA
AAGGATGGTTGGACATATTTGCCCGGTGGTGAACTCAAAGATATGATGTACCTGTTATCAAAGGACATGAAAAATAATGTTGTAATTGATTTCCCCCGTTGTACCAAGGAATTTATTTCA
TATAAATTCCTTGAGATGGTTAAGAATAGAACTATATATAGTTATAAGTATGAACCAGTTGGTTCAATCGTATGTAATAAGGTTCATGTAGTGGTCTTCAGTAATGAGGAGCCAGATTAT
GTAAAATTATCAGTGGATCGAATAAAGTTGATTTCTTGCTGAACACGCTATGAAATACAACACACTATGAAATACTGTACGGGTATCTGTATGGTTTATCTTAACGCTATTGGGCCGAAG
GCCCAAGCCCAAGCCCATGCCCCCAAAATGAACGGTCAGAATTGATTGCTTCGCCACGAAGCAAACAAAAAAATGCGTGGTGGAGAATTCGCCACGTCATTGGGTACTGTTATCCTTGGCG
CACTATATATATAGGTGCGCCAAAGTCGGCCATAG

BBTA2

Best match to EU430730 100% coverage 98% identity; MG545616 100% coverage 97% identity; NC_038953 100% coverage 96% identity

Rep protein best matches to AZL93963 100% coverage 99% identities (1 aa) 100% positives; ACB86656 100% coverage 99% identities (2 aa) 99% positives; YP_009508281 100% coverage 94% identities (11 gaps in middle) 94% positives Banana bunchy top alphasatellite 2

>BBTA2_alyu26
TATTACCCACCTCCGCGCACCACCTCCGCGCACCTATAAAATGTCTGCCTCTCGATGGACATTTACGCTTCATTATTCCGACGCAACGGAGCGAGGCAAATTCCTCGCGACTTTGAAGGA
GGAAGATGTGCACTACGCCGTCGTCGGCGACGAAACTGCTCCGAATACTGGTCGAAAACATCTTCAAGGATATCTTTCCTTGAAGAAACGTTTTCGTATTAGCGGGATAAAGAAGAAATA
TTCGTCGAGAGCGCATTGGGAGAAAGCTCGAGGATCAGATTACGACAACAAGGCGTATTGTTCCAAAGAAGCCCTAATTCTCGAATTAGGGGTTCCTTGCCAAACAGGTTCGAATAAGCG
TAAATTAGCAGATATGGTTACAAGATCTCCGGAACGAATGAAAATTGAACAGCCAGAGATATTTCACCGATACGAATCGGTGAAGAAGATGAAAGAATTCAAAGAAAGGTATGTCTATCC
TATCCTCGATAGGCCATGGCAGGTACAATTAACGGAGTTAATTGAAGCAGAACCTGATGATCGAACGATCATCTGGGTATTCGGACCAAAAGGGAATGAAGGCAAATCAACGTATGCGAA
GTCATTAATCCAAAAGGATTGGTTCTACACCAGGGGAGGAAAGAAGGAGAACATATTGTTCGCCTACGTAGATGAAGGTTCGACCAAAAACGTTGTATTTGATCTTCCGCGTACAGTACA

AGAATTTATTAATTATGATGTAATCGAGGCACTGAAGGATAGAGTAATCGAGAGTACAAAATACAAGCCTGTGAAGTATTTAGAATTGAATACTGTACATGTAATAGTTATGGCTAATTT
TCTTCCTGATATGTGTAAAATATCTGAAGATCGAATAAAAATAGTTGCTTGCTGAACACGCTATGACAAACGCACGCTATGACAAAAGGGGAAAAAAGAAAAATCGGGGGTTGATTGGGC
TATCCTAACGATTAAGGGCCGCAGGCCCGTCAAGATGGATCCAATAACCCGATAAGAAGGTAAACGGGTCTAAAACGATTTCTTCGCCCGCAAGCAACCACTTTAACCTCTGCGCACCTA
TATATAGCGGAGGTGGGCTAG

BBTA3
Best match to AF216221 ca 100% coverage 80% identity; U12586 ca 100% coverage ca. 83% identity; GU074392 ca 100% coverage 82% identity; NC_038955 ca 100% coverage 82% identity

Rep protein best matches to ADV18492 100% coverage 87% identities 96% positives; YP_009508283 100% coverage 86% identities 96% positives; ACJ36781 100% coverage 85% identities 94% positives

>BBTA3_alyu26
TATTACCCGCTTCCTCGCCCCTCTTCCTCGCCCCAGTGCAAAGCACGTGATGTCTTCCTTTAAATGGTGCTTCACGCTGAATTACTCCAACGCAACAGAGAGAGAAGACTTTCTCTCTCT
TTTGAAGGAGGAGGACTTGCGCTACGCAGTAGTGGGAGACGAAGTCGCCCCGTCAACTGGTCAGAAGCACCTACAGGGATATATATCCCTAAGGAAGAGTATGAAACTGGGTGGTTTGAA
GAAAAAATACTCGGCGAAGGCACACTGGGAGAAGGCGAAAGGATCAGATGAAGATAATACAAAGTATTGTTCCAAGGAAACCCTAATTCTTGAATTAGGGGTTCCTACATCCCAGGGTTC
AAACAAGCGTAAGCTTTCAGAGATGGTTACCAGATCACCGGAACGCATGAGGATAGAACAGCCAGAGATATATCACAGATATCAATCTGTGAATAAGTTAAAACTATTCAAAGAGGAATT
CGTACATCCTTGCCTCGATAGACCATGGCAGATACAATTGACGGAGGCAATTGAAGAGGAACCAGATGATCGAAGTATCATCTGGGTCTATGGACCGAATGGCAATGAGGGGAAATCAAC
ATATGCGAAGTCTCTGATAAAACATGATTGGTTCTATACAAGGGGAGGCAAGAAGGAGAACATACTGTTCTCCTACGTAGACGAAGGGTCTACGAAGCATGTTGTGTTCGACATACCAAG
ATGTCATCAAGAATATCTAAATTATGATGTAATCGAGGCATTGAAGGATAGGGTGATCGAGAGCACTAAGTATAAGCCGATTAAAATAATCGAGTTGAATAGGGTACATGTAATTGTAAT
GGCTAACTTCATGCCAGAGTACTGTAAGATCTCTGAGGATCGAATAAAATTAATATTTGCATAGCGCACGCTATGACAAAAGGGCAAAAATGCAAAATCGGGGGTTGATTGGGCTATCCT
AACGAATAAGGGCCGCAGGCCCGTCAAGATGGATCCGTTAACCCGGTATCAGATTACTTCGGGACGAAGGAATCACTTTAAGTTCCTCGCCCCTATATATAGTTTTGCCCGAGGAAGCGG
CCTAG

DNA-C
20-24 SNPs to KY427060 MF039864 MF039876 (**Thailand**). **24/26 SNPs** to KM607099 KM607098 (**China**). **26 SNPs** to MF039870 (**Thailand**)

C protein (161 aa): 2 aa to KY427060 (**Thailand**) **3 aa** to KM607098 KM607099 (**China**) MF039864 (**Thailand**)

>C_alyu26
TATTACCCCCCCTGCTCGGGACGGGACATCACGTGCATCAAACAAATGCACGTGACAAAGTAGTAGCTTGTAGCGAAAGATAACCATCACTATCACAAAAGTGAGAGGAATATTCGTTGC
TTAGGGACTAAGCAACGAGGACAGATAATTGTTCGAGAAGCGACAATGGAGGCTATTTAAACCTGATGGTTTTGTGATTTCCGAAATCACTCATCGGAGCAGAAATGGAGTTCTGGGAAT
CGTCTGCCATGCCAGACGATGTCAAGAGAGTGATCAAGGAAATATATTGGGAGCATCGAAACAAACTTCTGTTCTGTCAGAAGTTGAAAGGCTGTGTAAGAAGGATTCTGGAGCATGGAA
ATCAAGATGATGCTCTAGACGCAGTGAAGGAGATGAAGACTTCTATTATTCGCTATAGCGAATATTTGAAGAAACCTTGTGTGGTATTGTGTTTTAGTAATAATAAATCTATTGCATATA
GGTTAAACACAATGGTGTTCTTTTATCATGAATATATTGAAGACCTAGGTGGTGACTACTCGGTATATCAAGATCTGTTTTGTGATGAGGAACTTCCTTATTCGTCGACAGAAGAAGAAG
ATGAAGAAGTAATATACAGGAATGTTATCATGGCATCGACAGAAGAGAAGATCTCTTGGAGTGAATGTCAAAAGATAGTTATATCAGATTATGATGTAACATTACTGTAATGAATATCCA
TTATCATAAATAAAATAATGGAATGATGATTATGTATTCTAGTATAAATACATAATGGTATACGTATAGCATAAAATACATATACCTACATACAACACACTATGACAAACAGGGAAAAAT
GAAGAATCGGGGGTTGATTGGGCTATCTTAACGATTAAGGGCCGAAGGCCCGTTTAAATATGTGGTGGACGAAGTCCACAACACATAAAAAGTGAGCAGAACAATGGAATATAATGAGCT
GGCAATGTAGGGACCATGTCCCGAGTTAGTGCGCCACGTGAGCAGGGGGGCTTAT

DNA-M
22-27 SNPs to KY427061 MF039871 MF039865 MF039876 (**Thailand**). **55 SNPs** to KM607239 (**China**)

M protein (117 aa): 2 aa to KC581796 (**Thailand**) **3 aa** to MF039871 MF039865 MF039877 KY427061 (**Thailand**)

>M_alyu26
TATTACCCCCAGCGCTCAGGACGGGACATCACGTGCAACTAACAAATGCACGTGACTGATATATACTACATAACGGTTTATTGAACCGTTATATATTCTTGTAACGTAAAGTCACGTGTG
ATAATGAAATGCACGTGACCAAGTCAAATGTATTGAATAAACATTTGACGTCCGGATGCTTCCTACGGAAGCCATGATTGCTTCGTGGCGAAGCAAAACATTTATATATTGGCTTGGACT
GCTGCCTATAAATAGGCACGCAGGGAAATGGCATTAACAACAGAGCGAGTGAAACTATTCTTTGAATGGTTTCTGTTTATTGGCGCAATATTTATTGCAATAACAATATTATACATATTG
TTGGCGTTGCTCTTTGAGGTCCCCAAGTATATTAAGGATGTTGTGAGGTATCTTGTAGAATACCTGACCAGACGACGTGTATGGATGCAGAGAACGCAGTTGACGGAGGCAACAGGAGAT
GCAGAACTCGGCAGAGGTATTGTGGAAGACAGACGGGATCAACAACCGGCTGTCATACCACATGCAACACAGGTTATCCCTTCGCATCAACCTAGAAGGGATGAGCAAGGAAGACGAGGA
AACGCCGGACCAATGTTTTAATACACGGTATTGTAATATACGAAATATAAATGGGTAATGATAAGTATTGTGAAACAAAATATATGTGAAACATAATATATGTTTGATAATTTACATATT
ATAATATGTGAATTGTATACGAGTGTTGTATTTATAGAACATACAACACGCTATGACAAACAGGGAAAAATGAAGAATCGGGGGTTGATTGGTCTATCGTATCGCTTAAGGGCCGCAGGC
CCGTTGAAATGATTCTTTATAAAACAAATATACATGATACGGATTGTTGAAAATATAATCAACGATGTATAAATACAACAGAATGTTGTATACTAATTAAAATAATGAGAAGATAAGTAT
ATTTGTGAAGGATAAGGATCACAACCACCACTTTAGTGGTGGGTCATATGTCCCGAGTTAGTGCGCCACGTAAGCGCTGGGGCTTAT

DNA-N
19-23 SNPs to MF039872 MF039878 MF039866 KY427062 (**Thailand**). **24 SNPs** to KM607387 KM607385 (**China**). **34 SNPs** to KM607386 (**China**)

N protein (154 aa): 0 aa to MF039872 MF039878 KY427062 (**Thailand**) KM607387 KM607385 (**China**) **1 aa** to MF039866 (**Thailand**)

>N_alyu26
TATTACCCCCCGTGCTTGGGACGGGACATGACGTCAGCATAGATTATAATGGGCCAACTAAAGGCCCATTTAATAGAATAGGCGGGCTTTTGACATATTTCAAAGGCCCAGCCTGGAAGT
GGATAATGTCACGTGCCTAATAAGAGGTTGCTTCGCCTCGAAGCAATCCGAATAAATGTTGCGTATTCAATACGCAACTGAAGTCTATTAATATGGATGTCTCTGCCGAATAAATCAGAG
CGTAAGCGAAGCtGAAGCGATGGATTGGGCAGAATCACAATTCAAGACATGTACTCATGGGTGCGATTGGAAGACGATATCATCGGATTCATCCGAGAATCGACAATATGTACCTTGCGT
CGACGCTGGCAGCGGAAGAAAGTCACCTCGCAAGGTACTGCTAAGATCTATTGAAGCTGTGTTTAATGGAAGCTTCAAAGGAAAtAACAGGAATGTTCGTGGGTTTATATACGTATCAAT
ACGAGACGATGATGGAGAAATGCGTCCAGTACTCATAATACCATTCGGAGGATATGGATATCATAATGATTTCTATTATTTCGAAGGGAAGGGGAAAGTTGAATGTGATATATCATCAGA
TTATGTAGCGCCAGGAGTCGATTGGAGCAGAGACATGGAAGTTAGTATTAGTAACAGCAACAACTGTAATGAATTATGTGATCTGAAGTGTTATGTTGTTTGTTCGTTAAGAATAAAGGA
ATAACAGATGTGCTGTAATGATTATTAATAAAACATATTTTCATGTAATTGATAGTTGTATAAAACATACAACACGCTATGACAAACAGGGAAAAATGAAAAATCGGGGGTTGATTGGTC
TATCGTATCGCTTAAGGGCCGCAGGCCCGTTGAAAAATAATAATCGAATTATAAACGTTTGATAATAATCAGAGATAGATGATAAGCATCCTAAAACATAGACGAAGTATATGCCTGTAT
AATATAAAAGAAGCATATAAATAAAATATGTAAACTAATCTCTGATTGGTTTAGAATGTAACCCCACCAACTTTATGTTGGTGGAAATGTCCCGATGACGTAAGCACGGGGGACTAT

DNA-R
10 SNPs to AB113659 (**Viet Nam**). **11-14 SNPs** to MF039879 MF039873 MF039867 (**Thailand**) KM607677 (**China**)

N protein (286 aa): 1 aa to KM607676 KM607677 KM607679 (**China**) **2 aa** to AB113660 (**Vietnam**) …

>R_alyu26
TATTACCCCCAGCGCTCGGGACGGGACATTTGCATCTATAAATAGACCTCCCCCCCCCTCCACTACAAGATCATCATCGTCGACAGAAATGGCGCGATATGTGGTATGCTGGATGTTCAC
CATCAACAATCCCGCTTCACTACCAGTGATGCGGGATGAGTTTAAATATATGGTATATCAAGTGGAGAGGGGACAGGAGGGTACTCGTCATGTGCAGGGATACGTCGAGATGAAGAGACG
AAGTTCTCTGAAGCAGATGAGAGTCTTCTTCCCTGGCGCACACCTTGAGAAACGAAAGGGAAGCCAAGAAGAAGCACGGGCATACTGTATGAAGGAAGACACAAGAATCGAAGGTCCCTT
CGAGTTTGGTGCCTTTAAATTGTCATGTAATGATAATTTATTTGATGTCATACAGGATATGCGTGAAACGCACAAACGGCCTTTGGAGTATTTATATGAGTGTCCAAATACCTTCGATAG
AAGTAAGGATACATTATACAGAGTTCAAGCAGAGTTGAATAAAACGAAGGCGATGAATAGCTGGAAAACATCCTTCAGTTCGTGGACATCGGAAGTTGAAAATATTATGGCGGAGCCATG
TCACCGAAGGATAATTTGGGTCTATGGCCCAAATGGAGGAGAAGGAAAGACAACTTATGCAAAATATTTAATGAAGACGAAGAATGCGTTTTATTCGCCTGGAGGAAAATCATTGGATAT
ATGTAGATTGTATAATTATGAGGAAATAGTTATATTTGATATTCCCAGATGCAAAGAGGAATATTTAAACTATGGTTTATTAGAAGAATTTAAAAATGGAATTATTCAAAGCGGGAAATA
TGAACCCGTTTTGAAAATTGTAGAATATGTGGAAGTCATTGTAATGGCTAACTTCCTTCCGAAGGAAGGAATCTTTTCAGAAGATCGAATAAAGCTAGTTGCTTGCTGAACACGCTATGA
CAATCGTACGCTATGACAAAAGGGGAAAAGCAAAGAATCGGGGGTTGATTGGGCTATCCTAACGATTAAGGGCCGCAGGCCCGTCAAGATGGACGGTTTGATCAGATGTCCCGAGTTAGT
GCGCCACGTAAGCGCTGGGGCTTAT

DNA-S
10/13 SNPs to AB113661/AB113662 (**Viet Nam**). **17 SNPs** to MF039874 (**Thailand**). **19-20 SNPs** to KM607535 KM607534 (**China**). **23 SNPs** to KY427064 (**Thailand**)

S protein (170 aa): 0 aa to AY337715 AY494786 HQ378191 (**China**) FJ787433 FJ787434 (**Philippines**) **1 aa** to several isolates

>S_alyu26
TATTACCCCCAGCGCTCGGGACGGGACATGGGCTAATGGATCATGGAATAAGGGCCCATCGGGCCCGTTTAGATGGGTTTTGGGCTTATGGGCTCTATCCAGAAGACCAAAAACAGGCGG
GAACCGTCCCAATTTCAAATTTAGATAGCTTGCCCCGCAAGCAAACTAAAAGTCTATATATACCAGTGTAGACATATTGTTCAGAGAACAAATGGCGAGGTTTCCGAAGAAATCGATCAA
GAAGAGGCGGGTTGGGCGACGGAAGTATGGAAGCAAGGCGGCAACCAGTCACGACTACTCGTCGTTAGGTTCAATATTGGTTCCTGAAAATACCGTCAAGGTATTTAGGATTGAGCCTAC
TGATAAAACATTACCCAGATATTTTATCTGGAAAATGTTTATGCTTTTGGTGTGCAAGGTGAAGCCCGGAAGAATACTTCACTGGGCTATGATTAAAAGTTCATGGGAAATCAACCAGCC
GACTACATGTCTGGAAGCACCAGGTTTATTTATAAAACCTGAACATAGCCATCTGGTGAAACTGGTATGCAGTGGGGAACTTGAAGCCGGAGTCGCAACAGGGACATCAGATGTTGAATG
TCTTCTAAGGAAGACAACCGTGTTGAGGAAGAATGTAACAGAGGTGGATTACTTGTATTTGGCATTTTATTGTAGTGCTGGAGTTAGTATTAACTACCAGAACAGAATTACATATCATGT
ATGATATGTTTATGTAAACATAAACCTTTGTATGGAATAATGACCAAATAACATACAAGACGCTATGAAATACAACACGCTATGACAAACAGGGAAAAATGAAGAATCGGGGGTTGATTG
GTCTATCGTATCGCTTAAGGGCCGCAGGCCCGTTGAAATGATTCTTTATAAAACAAATATACATGATACGGATTGTTGAAAATATAATCAACGATGTATAAATACAACAGAATGTTGTAA
ACTAATTAAAAAAATGAGAAGAAAAGAATATTTGTGAAGGATAAGCATCAGAACCACCACTTTAGTGGTGGGTCATATGTCCCGAGTTAGTGCGCCACGTAAGCGCTGGGGCTTAT

DNA-U3
46-48 SNPs to KY427065 MF039869 (**Thailand**)

U3 protein (39 aa): 0 aa to KY427065 (**Thailand**)

>U3_alyu26
TATTACCCCCAGCCCCCAGGACGGGACATGGGCTTTTTAAATGGGCCGAGAGAGTTTGAACAGTTCAGTATCTTCGTTATTGGGCCATCTGGCCCAATAATTAAGAGAACGTGTTCAAAA
TCTGGGTTTGACCGAAGGTCAAGGTAGACGGTCAACAATATTCTGGCTTGCGGAGCAAGCAACAAGAATTAAATATTTAATTCGTATGACACGTGGACGGACCGAAATACTCCTGCATCT
CTATAAATAGCCTAAAACTGGCATGGATAATTGCTCTCGCTCTTCTGTCAAAGCTATTGTGTTGAGGCGGAAGATCGCCATCGGCGATCATCGGACGAAAAGCTGCAAGAGAGACGGATA
ACCATGCTGCGAAGCGTATATCGGGTATTTATAGACTTCTAGCGCAGCTAGAAGTTTCCTTGTATTTGATATTGTATTTTGTAAATTACGAAGAAATTCGTACATTGATAATAATAAAAC
ATCTGGGATTGTTAATGTTTACATTAACCAGTATTAATAATGTACAATAACAAATATTGTATGAGGAACGAATACAATAATATTAAAATATGAGGAGCGTAGCGTGATAAACAGGTGTTT
AAGGTATAATTAAATTAATTATGCAATGTAATGATAATACGGTATAAGTTGAAGTATGAGGTGAAAGAGGAGATATTAGAATATTAAAAACCCAATTATATTATTTATAATACATACAAC
ACGCTATGACAAACGGGGAAAAAAGAAAAATCGGGGGTTGATTGGTCTATCCTAACGATTAAGGGCCGCAGGCCCGTCAAGATGGATGAACGGTTAGATTTGATTGCTTAGCGACGAAGA
AAGGAATCGTTTTGGGACCACAGACAAGACAGCTGTCACTACTTTTAAAAAATAATATAATAACCAATTGACGATAGTACCCCTCCCAAAGATGACGTATAGGGGTGTCCCGAGGTAATT
TAACATAGCTCTGACAAGAGATAGTGGACGTTGGATGCCACGATCGGACGATCGTGGTTTGATGAACCAATGATGACGTAGGGGCTGGGGCTTAT

ALYU-27 - **Commelina sp.** - **Vietnam**

Neighbour of Musa ALYU-26

Virome
1. **BBTV** six components - consensus alyu27 = consensus alyu26. However, many SNPs distinguish alyu27 from alyu26, while only some SNPs are shared between alyu26 and alyu27
2. **BBTA6** alyu27=26=38v2 - new alphasatellite named here Banana bunchy top alphasatellite 6, genus *Banaphisatellite*
3. **BBTA2** alyu27=26 - Banana bunchy top alphasatellite 2, genus *Muscarsatellite*
4. **BBTA3** alyu27=26=38 - Banana bunchy top alphasatellite 3, genus *Muscarsatellite*

BBTA6 (alyu27 = alyu26)

>BBTA6_alyu27
TATTACCCGACTTTGGCGCAGGGCGCACTATAAAAGATGTCTGTGAGAAATTGGGTCTTCACACGCAATTTCGAAGGCGAAAGACCAGAGCTCCTGTTCGGCGGCGAAACTCAATACGCC
GCTTGGCAACATGAAAGAAAGAATCACGACCACCTACAGGGTGTGATTCAGTTGAAGAAGAAGACCCGACTGAACGCAGTGAAGAGCATAATCGGTGGAAATCCACATGTCGAACCCATG
CGAGCGTCTTTCGACGATGCCCTAGCATACGTCATGAAAGACGAAACCCGAATCGATGGTCCATGGGAATTCGGTATTCGAATCCGTCAGGGTTCGAATAAACGAAAGCTGTTAGAAATA
CTGGAGGATTCGGACAACGAAGTCCTTCAACCCCAAAAATACAGACGAGCCATGGCCAAACAGGCCATGAGTGAGTCTAAGAAGAAAGCTGCAGAAGAAGGATTTAGTTATGAACTGCGA
GAGTGGCAAGTGCAATTGACGAAGTTGTTGGAAGAAGAACCCGATAACAGAACAATAATTTGGGTATATGGACCAAAGGGTGGAGAAGGTAAAACAGAGTTCGCTAAACATTTAGGGTTA
AAGGATGGTTGGACATATTTGCCCGGTGGTGAACTCAAAGATATGATGTACCTGTTATCAAAGGACATGAAAAATAATGTTGTAATTGATTTCCCCCGTTGTACCAAGGAATTTATTTCA
TATAAATTCCTTGAGATGGTTAAGAATAGAACTATATATAGTTATAAGTATGAACCAGTTGGTTCAATCGTATGTAATAAGGTTCATGTAGTGGTCTTCAGTAATGAGGAGCCAGATTAT
GTAAAATTATCAGTGGATCGAATAAAGTTGATTTCTTGCTGAACACGCTATGAAATACAACACACTATGAAATACTGTACGGGTATCTGTATGGTTTATCTTAACGCTATTGGGCCGAAG
GCCCAAGCCCAAGCCCATGCCCCCAAAATGAACGGTCAGAATTGATTGCTTCGCCACGAAGCAAACAAAAAATGCGTGGTGGAGAATTCGCCACGTCATTGGGTACTGTTATCCTTGGCG
CACTATATATAGGTGCGCCAAAGTCGGCCATAG

BBTA2 (alyu27 = alyu26)

>BBTA2_alyu27
TATTACCCACCTCCGCGCACCACCTCCGCGCACCTATAAAATGTCTGCCTCTCGATGGACATTTACGCTTCATTATTCCGACGCAACGGAGCGAGGCAAATTCCTCGCGACTTTGAAGGA
GGAAGATGTGCACTACGCCGTCGTCGGCGACGAAACTGCTCCGAATACTGGTCGAAAACATCTTCAAGGATATCTTTCCTTGAAGAAACGTTTTCGTATTAGCGGGATAAAGAAGAAATA
TTCGTCGAGAGCGCATTGGGAGAAAGCTCGAGGATCAGATTACGACAACAAGGCGTATTGTTCCAAAGAAGCCCTAATTCTCGAATTAGGGGTTCCTTGCCAAACAGGTTCGAATAAGCG
TAAATTAGCAGATATGGTTACAAGATCTCCGGAACGAATGAAAATTGAACAGCCAGAGATATTTCACCGATACGAATCGGTGAAGAAGATGAAAGAATTCAAAGAAAGGTATGTCTATCC
TATCCTCGATAGGCCATGGCAGGTACAATTAACGGAGTTAATTGAAGCAGAACCTGATGATCGAACGATCATCTGGGTATTCGGACCAAAAGGGAATGAAGGCAAATCAACGTATGCGAA
GTCATTAATCCAAAAGGATTGGTTCTACACCAGGGGAGGAAAGAAGGAGAACATATTGTTCGCCTACGTAGATGAAGGTTCGACCAAAAACGTTGTATTTGATCTTCCGCGTACAGTACA
AGAATTTATTAATTATGATGTAATCGAGGCACTGAAGGATAGAGTAATCGAGAGTACAAAATACAAGCCTGTGAAGTATTTAGAATTGAATACTGTACATGTAATAGTTATGGCTAATTT
TCTTCCTGATATGTGTAAAATATCTGAAGATCGAATAAAAATAGTTGCTTGCTGAACACGCTATGACAAACGCACGCTATGACAAAAGGGGAAAAAAGAAAAATCGGGGGTTGATTGGGC
TATCCTAACGATTAAGGGCCGCAGGCCCGTCAAGATGGATCCAATAACCCGATAAGAAGGTAAACGGGTCTAAAACGATTTCTTCGCCCGCAAGCAACCACTTTAACCTCTGCGCACCTA
TATATAGCGGAGGTGGGCTAG

BBTA3 (alyu27 = alyu26)

>BBTA3_alyu27
TATTACCCGCTTCCTCGCCCCTCTTCCTCGCCCCAGTGCAAAGCACGTGATGTCTTCCTTTAAATGGTGCTTCACGCTGAATTACTCCAACGCAACAGAGAGAGAAGACTTTCTCTCTCT
TTTGAAGGAGGAGGACTTGCGCTACGCAGTAGTGGGAGACGAAGTCGCCCCGTCAACTGGTCAGAAGCACCTACAGGGATATATATCCCTAAGGAAGAGTATGAAACTGGGTGGTTTGAA
GAAAAAATACTCGGCGAAGGCACACTGGGAGAAGGCGAAAGGATCAGATGAAGATAATACAAAGTATTGTTCCAAGGAAACCCTAATTCTTGAATTAGGGGTTCCTACATCCCAGGGTTC
AAACAAGCGTAAGCTTTCAGAGATGGTTACCAGATCACCGGAACGCATGAGGATAGAACAGCCAGAGATATATCACAGATATCAATCTGTGAATAAGTTAAAACTATTCAAAGAGGAATT
CGTACATCCTTGCCTCGATAGACCATGGCAGATACAATTGACGGAGGCAATTGAAGAGGAACCAGATGATCGAAGTATCATCTGGGTCTATGGACCGAATGGCAATGAGGGGAAATCAAC
ATATGCGAAGTCTCTGATAAAACATGATTGGTTCTATACAAGGGGAGGCAAGAAGGAGAACATACTGTTCTCCTACGTAGACGAAGGGTCTACGAAGCATGTTGTGTTCGACATACCAAG
ATGTCATCAAGAATATCTAAATTATGATGTAATCGAGGCATTGAAGGATAGGGTGATCGAGAGCACTAAGTATAAGCCGATTAAAATAATCGAGTTGAATAGGGTACATGTAATTGTAAT
GGCTAACTTCATGCCAGAGTACTGTAAGATCTCTGAGGATCGAATAAAATTAATATTTGCATAGCGCACGCTATGACAAAAGGGCAAAAATGCAAAATCGGGGGTTGATTGGGCTATCCT
AACGAATAAGGGCCGCAGGCCCGTCAAGATGGATCCGTTAACCCGGTATCAGATTACTTCGGGACGAAGGAATCACTTTAAGTTCCTCGCCCCTATATATAGTTTTGCCCGAGGAAGCGG
CCTAG

DNA-C

>C_alyu27
TATTACCCCCCCTGCTCGGGACGGGACATCACGTGCATCAAACAAATGCACGTGACAAAGTAGTAGCTTGTAGCGAAAGATAACCATCACTATCACAAAAGTGAGAGGAATATTCGTTGC
TTAGGGACTAAGCAACGAGGACAGATAATTGTTCGAGAAGCGACAATGGAGGCTATTTAAACCTGATGGTTTTGTGATTTCCGAAATCACTCATCGGAGCAGAAATGGAGTTCTGGGAAT
CGTCTGCCATGCCAGACGATGTCAAGAGAGTGATCAAGGAAATATATTGGGAGCATCGAAACAAACTTCTGTTCTGTCAGAAGTTGAAAGGCTGTGTAAGAAGGATTCTGGAGCATGGAA
ATCAAGATGATGCTCTAGACGCAGTGAAGGAGATGAAGACTTCTATTATTCGCTATAGCGAATATTTGAAGAAACCTTGTGTGGTATTGTGTTTTAGTAATAATAAATCTATTGCATATA
GGTTAAACACAATGGTGTTCTTTTATCATGAATATATTGAAGACCTAGGTGGTGACTACTCGGTATATCAAGATCTGTTTTGTGATGAGGAACTTCCTTATTCGTCGACAGAAGAAGAAG
ATGAAGAAGTAATATACAGGAATGTTATCATGGCATCGACAGAAGAGAAGATCTCTTGGAGTGAATGTCAAAAGATAGTTATATCAGATTATGATGTAACATTACTGTAATGAATATCCA
TTATCATAAATAAAATAATGGAATGATGATTATGTATTCTAGTATAAATACATAATGGTATACGTATAGCATAAAATACATATACCTACATACAACACACTATGACAAACAGGGAAAAAT
GAAGAATCGGGGGTTGATTGGGCTATCTTAACGATTAAGGGCCGAAGGCCCGTTTAAATATGTGGTGGACGAAGTCCACAACACATAAAAAGTGAGCAGAACAATGGAATATAATGAGCT
GGCAATGTAGGGACCATGTCCCGAGTTAGTGCGCCACGTGAGCAGGGGGGCTTAT

DNA-M

>M_alyu27
TATTACCCCCAGCGCTCAGGACGGGACATCACGTGCAACTAACAAATGCACGTGACTGATATATACTACATAACGGTTTATTGAACCGTTATATATTCTTGTAACGTAAAGTCACGTGTG
ATAATGAAATGCACGTGACCAAGTCAAATGTATTGAATAAACATTTGACGTCCGGATGCTTCCTACGGAAGCCATGATTGCTTCGTGGCGAAGCAAAACATTTATATATTGGCTTGGACT
GCTGCCTATAAATAGGCACGCAGGGAAATGGCATTAACAACAGAGCGAGTGAAACTATTCTTTGAATGGTTTCTGTTTATTGGCGCAATATTTATTGCAATAACAATATTATACATATTG
TTGGCGTTGCTCTTTGAGGTCCCCAAGTATATTAAGGATGTTGTGAGGTATCTTGTAGAATACCTGACCAGACGACGTGTATGGATGCAGAGAACGCAGTTGACGGAGGCAACAGGAGAT
GCAGAACTCGGCAGAGGTATTGTGGAAGACAGACGGGATCAACAACCGGCTGTCATACCACATGCAACACAGGTTATCCCTTCGCATCAACCTAGAAGGGATGAGCAAGGAAGACGAGGA
AACGCCGGACCAATGTTTTAATACACGGTATTGTAATATACGAAATATAAATGGGTAATGATAAGTATTGTGAAACAAAATATATGTGAAACATAATATATGTTTGATAATTTACATATT
ATAATATGTGAATTGTATACGAGTGTTGTATTTATAGAACATACAACACGCTATGACAAACAGGGAAAAATGAAGAATCGGGGGTTGATTGGTCTATCGTATCGCTTAAGGGCCGCAGGC
CCGTTGAAATGATTCTTTATAAAACAAATATACATGATACGGATTGTTGAAAATATAATCAACGATGTATAAATACAACAGAATGTTGTATACTAATTAAAATAATGAGAAGATAAGTAT
ATTTGTGAAGGATAAGGATCACAACCACCACTTTAGTGGTGGGTCATATGTCCCGAGTTAGTGCGCCACGTAAGCGCTGGGGCTTAT

DNA-N

>N_alyu27
TATTACCCCCCGTGCTTGGGACGGGACATGACGTCAGCATAGATTATAATGGGCCAACTAAAGGCCCATTTAATAGAATAGGCGGGCTTTTGACATATTTCAAAGGCCCAGCCTGGAAGT
GGATAATGTCACGTGCCTAATAAGAGGTTGCTTCGCCTCGAAGCAATCCGAATAAATGTTGCGTATTCAATACGCAACTGAAGTCTATTAATATGGATGTCTCTGCCGAATAAATCAGAG
CGTAAGCGAAGCtGAAGCGATGGATTGGGCAGAATCACAATTCAAGACATGTACTCATGGGTGCGATTGGAAGACGATATCATCGGATTCATCCGAGAATCGACAATATGTACCTTGCGT
CGACGCTGGCAGCGGAAGAAAGTCACCTCGCAAGGTACTGCTAAGATCTATTGAAGCTGTGTTTAATGGAAGCTTCAAAGGAAAtAACAGGAATGTTCGTGGGTTTATATACGTATCAAT
ACGAGACGATGATGGAGAAATGCGTCCAGTACTCATAATACCATTCGGAGGATATGGATATCATAATGATTTCTATTATTTCGAAGGGAAGGGGAAAGTTGAATGTGATATATCATCAGA
TTATGTAGCGCCAGGAGTCGATTGGAGCAGAGACATGGAAGTTAGTATTAGTAACAGCAACAACTGTAATGAATTATGTGATCTGAAGTGTTATGTTGTTTGTTCGTTAAGAATAAAGGA
ATAACAGATGTGCTGTAATGATTATTAATAAAACATATTTTCATGTAATTGATAGTTGTATAAAACATACAACACGCTATGACAAACAGGGAAAAATGAAAAATCGGGGGTTGATTGGTC
TATCGTATCGCTTAAGGGCCGCAGGCCCGTTGAAAAATAATAATCGAATTATAAACGTTTGATAATAATCAGAGATAGATGATAAGCATCCTAAAACATAGACGAAGTATATGCCTGTAT
AATATAAAAGAAGCATATAAATAAAATATGTAAACTAATCTCTGATTGGTTTAGAATGTAACCCCACCAACTTTATGTTGGTGGAAATGTCCCGATGACGTAAGCACGGGGGACTAT

DNA-R

>R_alyu27
TATTACCCCCAGCGCTCGGGACGGGACATTTGCATCTATAAATAGACCTCCCCCCCCCTCCACTACAAGATCATCATCGTCGACAGAAATGGCGCGATATGTGGTATGCTGGATGTTCAC
CATCAACAATCCCGCTTCACTACCAGTGATGCGGGATGAGTTTAAATATATGGTATATCAAGTGGAGAGGGGACAGGAGGGTACTCGTCATGTGCAGGGATACGTCGAGATGAAGAGACG
AAGTTCTCTGAAGCAGATGAGAGTCTTCTTCCCTGGCGCACACCTTGAGAAACGAAAGGGAAGCCAAGAAGAAGCACGGGCATACTGTATGAAGGAAGACACAAGAATCGAAGGTCCCTT
CGAGTTTGGTGCCTTTAAATTGTCATGTAATGATAATTTATTTGATGTCATACAGGATATGCGTGAAACGCACAAACGGCCTTTGGAGTATTTATATGAGTGTCCAAATACCTTCGATAG
AAGTAAGGATACATTATACAGAGTTCAAGCAGAGTTGAATAAAACGAAGGCGATGAATAGCTGGAAAACATCCTTCAGTTCGTGGACATCGGAAGTTGAAAATATTATGGCGGAGCCATG
TCACCGAAGGATAATTTGGGTCTATGGCCCAAATGGAGGAGAAGGAAAGACAACTTATGCAAAATATTTAATGAAGACGAAGAATGCGTTTTATTCGCCTGGAGGAAAATCATTGGATAT
ATGTAGATTGTATAATTATGAGGAAATAGTTATATTTGATATTCCCAGATGCAAAGAGGAATATTTAAACTATGGTTTATTAGAAGAATTTAAAAATGGAATTATTCAAAGCGGAAATA
TGAACCCGTTTTGAAAATTGTAGAATATGTGGAAGTCATTGTAATGGCTAACTTCCTTCCGAAGGAAGGAATCTTTTCAGAAGATCGAATAAAGCTAGTTGCTTGCTGAACACGCTATGA
CAATCGTACGCTATGACAAAAGGGGAAAAGCAAAGAATCGGGGGTTGATTGGGCTATCCTAACGATTAAGGGCCGCAGGCCCGTCAAGATGGACGGTTTGATCAGATGTCCCGAGTTAGT
GCGCCACGTAAGCGCTGGGGCTTAT

DNA-S

>S_alyu27
TATTACCCCCAGCGCTCGGGACGGGACATGGGCTAATGGATCATGGAATAAGGGCCCATCGGGCCCGTTTAGATGGGTTTTGGGCTTATGGGCTCTATCCAGAAGACCAAAAACAGGCGG
GAACCGTCCCAATTTCAAATTTAGATAGCTTGCCCCGCAAGCAAACTAAAAGTCTATATATACCAGTGTAGACATATTGTTCAGAGAACAAATGGCGAGGTTTCCGAAGAAATCGATCAA
GAAGAGGCGGGTTGGGCGACGGAAGTATGGAAGCAAGGCGGCAACCAGTCACGACTACTCGTCGTTAGGTTCAATATTGGTTCCTGAAAATACCGTCAAGGTATTTAGGATTGAGCCTAC
TGATAAAACATTACCCAGATATTTTATCTGGAAAATGTTTATGCTTTTGGTGTGCAAGGTGAAGCCCGGAAGAATACTTCACTGGGCTATGATTAAAAGTTCATGGGAAATCAACCAGCC
GACTACATGTCTGGAAGCACCAGGTTTATTTATAAAACCTGAACATAGCCATCTGGTGAAACTGGTATGCAGTGGGGAACTTGAAGCCGGAGTCGCAACAGGGACATCAGATGTTGAATG
TCTTCTAAGGAAGACAACCGTGTTGAGGAAGAATGTAACAGAGGTGGATTACTTGTATTTGGCATTTTATTGTAGTGCTGGAGTTAGTATTAACTACCAGAACAGAATTACATATCATGT
ATGATATGTTTATGTAAACATAAACCTTTGTATGGAATAATGACCAAATAACATACAAGACGCTATGAAATACAACACGCTATGACAAACAGGGAAAAATGAAGAATCGGGGGTTGATTG
GTCTATCGTATCGCTTAAGGGCCGCAGGCCCGTTGAAATGATTCTTTATAAAACAAATATACATGATACGGATTGTTGAAAATATAATCAACGATGTATAAATACAACAGAATGTTGTAA
ACTAATTAAAAAAATGAGAAGAAAAGAATATTTGTGAAGGATAAGCATCAGAACCACCACTTTAGTGGTGGGTCATATGTCCCGAGTTAGTGCGCCACGTAAGCGCTGGGGCTTAT

DNA-U3

>U3_alyu27
TATTACCCCCAGCCCCCAGGACGGGACATGGGCTTTTTAAATGGGCCGAGAGAGTTTGAACAGTTCAGTATCTTCGTTATTGGGCCATCTGGCCCAATAATTAAGAGAACGTGTTCAAAA
TCTGGGTTTGACCGAAGGTCAAGGTAGACGGTCAACAATATTCTGGCTTGCGGAGCAAGCAACAAGAATTAAATATTTAATTCGTATGACACGTGGACGGACCGAAATACTCCTGCATCT
CTATAAATAGCCTAAAACTGGCATGGATAATTGCTCTCGCTCTTCTGTCAAAGCTATTGTGTTGAGGCGGAAGATCGCCATCGGCGATCATCGGACGAAAAGCTGCAAGAGAGACGGATA
ACCATGCTGCGAAGCGTATATCGGGTATTTATAGACTTCTAGCGCAGCTAGAAGTTTCCTTGTATTTGATATTGTATTTTGTAAATTACGAAGAAATTCGTACATTGATAATAATAAAAC
ATCTGGGATTGTTAATGTTTACATTAACCAGTATTAATAATGTACAATAACAAATATTGTATGAGGAACGAATACAATAATATTAAAATATGAGGAGCGTAGCGTGATAAACAGGTGTTT
AAGGTATAATTAAATTAATTATGCAATGTAATGATAATACGGTATAAGTTGAAGTATGAGGTGAAAGAGGAGATATTAGAATATTAAAAACCCAATTATATTATTTATAATACATACAAC
ACGCTATGACAAACGGGGAAAAAAGAAAAATCGGGGGTTGATTGGTCTATCCTAACGATTAAGGGCCGCAGGCCCGTCAAGATGGATGAACGGTTAGATTTGATTGCTTAGCGACGAAGA
AAGGAATCGTTTTGGGACCACAGACAAGACAGCTGTCACTACTTTTAAAAAATAATATAATAACCAATTGACGATAGTACCCCTCCCAAAGATGACGTATAGGGGTGTCCCGAGGTAATT
TAACATAGCTCTGACAAGAGATAGTGGACGTTGGATGCCACGATCGGACGATCGTGGTTTGATGAACCAATGATGACGTAGGGGCTGGGGCTTAT

ALYU-28 - Ipomoea aquatica - Vietnam

Neighbour of Musa ALYU-26

Virome
"Virus-free": Reads representing BBTV genome components and alphasatellites are at a cross-contamination level (see Supplementary Figure S3B).

ALYU-29 - Musa AA sucrier Pisang mas ? - Vietnam

Virome
1. **BBTV** six components
2. **BBTA5** alyu29 – new alphasatellite named here Banana bunchy top alphasatellite 5, genus *Banaphisatellite*
3. **BBTA2** alyu29 – Banana bunchy top alphasatellite 2, genus *Muscarsatellite*
4. **BBTA3** alyu29v2 – Banana bunchy top alphasatellite 3, genus *Muscarsatellite*
5. **BBTA5 defective** alyu29
6. **BBTA2 defective** alyu29
7. **BBTV DNA-R defective** alyu29 532 nt circular (deletion within Rep ORF)

BBTA5
Best matches to MK291270 77% coverage 67.3% identity Sophora yellow stunt alphasatellite 3; MF510475 MF510474 78% coverage 66.1% identity Faba bean necrotic yellows virus associated alphasatellite 2, MF510474 78% coverage 65.9% identity Faba bean necrotic yellows C9 alphasatellite

Rep protein best matches to QBF29185 ARI50297 98% coverage 63-62% identities 80-81% positives Sophora yellow stunt alphasatellite 3; ATU31571 98% coverage 62% identities 80% positives Faba bean necrotic yellows virus associated

alphasatellite 2; NP_619574 98% coverage 62% identities 80% positives Faba bean necrotic yellows C9 alphasatellite; QBO55993 98% coverage 61% identities 79% positives Faba bean necrotic yellows alphasatellite 2

>BBTA5_alyu29
TATTACCCGACCTTGGCTCACTGGCTCACTATAAAAGGgGGAGATGTCgCaGCAGCGAAATTGGGTATTCACGCGTAACtTCTCCGGCGACCTGCCGATTTTAAAGTTCGCCGGAGAAAC
TCAATACGCGTGTTGGCAACATGAGAGGAAGAATCACGACCATCTTCAGGGAGTGATTCAATTGAAGAAGAAGCTCCGGATGAACGCAGTGAAGACGTTAATTGGGGAAATCCACACCT
CGAAGCCATGAGAGGAACAATCGACGAAGCAATAAGGTACGTGACGAAAGAAGAGACACGAGTAGCAGGTCCGTGGGAATTCGGAGAATTACTACGAAAAGGATCTCATAAGAGGAAATT
GATGGAATTGCTCGACGATCCAGATAACGAAATAATGGAACCTCAAAAATATCGACGCGCCATTACTAAACAAGCAATGGACGCGTCAAAGAAGAAGGCGGAATTAGGGTTTCCTTACGA
CTTAAAGGAGTGGCAAAAGATGGTTATTGAGTTAATCGAAGAACAACCCGACAACAGAACTATTATCTGGGTGTATGGACCTAATGGTGGAGAAGGTAAAACGCAGTTCGCAAAGCATTA
TGGATTACTCAAAGGATGGACATATCTACCAGGTGGTGAACTGAAAGACATGATGTATCTGTTGGCAAAAGAAATTAACAATAACGTAATTATTGATTTCCCCCGATGTACAAAGGATTT
CATATCCTATAAGTTTATTGAGATGGTAAAGAACCGATGCATATTTAGTTATAAATATGAACCAATCGGGGCGATTGTAAGTAATGAAGTCCATGTAATTGTAATGTCGAATGAGCTTCC
AGATTATTCAAAAATATCGGAAGACAGAATAAAATTAATATTTGCATAGCGCACACTATGACAAAAGGGCAAAAATGCAAAATCGGGGGTTGATTTGTCTATATTTACGATTAAGGGCCG
AAGGCCCGTACACATTGCGCGGGAAATTCAAAATCGGACGCCAAATATTATTAAATACAATACACCGCCTTTTAGAAATCCGTGGACGAGAAGATGCCACGTAGCAAAACACTGTACTCC
TTGGCTCACTATAAATACCTGAGCCAAGGTCGGGCATAG

BBTA5def
Defective derivative of BBTA5_alyu29 (87% non-gap nt identity), **non-autonomous satellite**
Two big regions are derived from BBTA5_alyu29: 886-1119/1-265 and 617-876 (with several SNPs)
Two short regions (140 and 80 nt) share 80% identity with BBTV DNA-N

no Rep protein coding capacity, only short ORFs but two of them matching to alphasatellite Rep (due to BBTA5-derived regions)

>BBTA5def_alyu29
TATTACCCGACCTTGGCTCACTGGCTCACTATAAAAGGCGGAGATGTCTCCGCAGCGAAATTGGGTATTCACGCGTAACCTCTCCGGCGACCAGGCGATTTAAAGTTCGCCGGAGAAACT
TAATACGCGTGTTGGTAATATGAGATGAAGAATCACGACCATCTTCAGGGAGTGATTCAATTGAAGAAGAAGCTCCGGATGAACGCAGTGAAGACGGAAATTGGGGAAATCCACTCCTC
GAAGCCATGAGAGGAAGAATCACGACCATGTTCAGGGAGTGATTCAATTGAAGAAGAAGCTCCGAATGAACGCAGTGAAGGTGTTAATTGGGGATTATCCACAATAATTAAATTATAAAT
GATAGATAATAATCAGAGATAGATGATTAGCATCTAAAAACATAGACGAAGTATATGCTGTATAATATAAAAGATATACATAAACAAAAAAAAAAAAAAATGTGAACTAATCTCTGATTG
GACATATCTACCAGTGGATGAACTGAAAGCCTGATGTATCTGTTGGTAAAAGAAATTAATAATAATGTAATCATTGATTTCCCCCGATGTACAGAGGATTTCATATCCTATAAGTTTATT
GGGATGGTAAAGAACCGATGCATATTTAGTTATAAATATGAATCAATCTCGGCGATCGTAAGTAATGAAATCCATGTAATTGTAATGTTCAACGAGCTTCCAGATTAATCAAAAAATTTC
GGAAGACATAATAAAATTTTAGTAACATAATGCACACTATGACAAAAGGCCACTAGTTGCGTGCTGAACACGCTATGTCAATCGTACGGTACGACAGAATGGCAAAAATGTAAAATCGCA
CACTATGACAAAAGGGCAAAAATGCAAAATCGGGGGTTGATTTGTCTATATTTACGATTAAGGGCCGAAGGCCCGTACACATTGCGCGGGAAATTCAAAATCGGACGCCAAAAATTATTA
AATACAATACACCGCCTTTTAGAAATCCGTGGACGAGAAGATGCCACGTAGCAAAACACTGTACTCCTTGGCTCACTATAAATACCTGAGCCAAGGTCGGGCATAG

BBTA2
Best match to EU430730 100% coverage 98% identity; MG545616 100% coverage 97% identity; NC_038953 100% coverage 96% identity

Rep protein best matches to AZL93963 100% coverage, 99% identities (1 aa) 100% positives; YP_009508281 100% coverage 94% identities (11 gaps in middle), 94% positives

>BBTA2_alyu29
TATTACCCACCTCCGCGCACCACCTCCGCGCACCTATAAAATGTCTGCCTCTCGCTGGACATTTACGCTTCATTATTCCGACGCAACGGAGCGAGGCAAATTCCTCGCGACTTTGAAGGA
GGAAGATGTGCACTACGCCGTCGTCGGCGACGAAACTGCTCCGAATACTGGTCGAAAACATCTTCAAGGATATCTTTCCTTGAAGAAACGTTTTCGTATTAGCGGAATAAAGAAGAAATA
TTCGTCGAGAGCGCATTGGGAGAAAGCTCGAGGATCAGACTACGACAACAAGGCGTACTGTTCCAAAGAAGCCCTAATTCTTGAATTAGGGGTTCCTTGCCAAACAGGTTCGAATAAGCG
TAAATTAGCAGATATGGTTACAAGATCGCCGGAACGAATGAAAATTGAACAGCCAGAGATATTTCACCGATACGAATCGGTGAAGAAGATGAAAGAATTCAAAGAAAGGTATGTCTATCC
TATCCTCGATAGGCCATGGCAGGTACAATTAACGGAGTTAATTGAAGCAGAACCTGATGATCGAACGATCATCTGGGTATTCGGACCAAAAGGGAATGAAGGCAAATCAACGTACGCGAA
GTCATTAATCCAAAAGGATTGGTTCTACACCAGGGGAGGAAAGAAGGAGAACATACTGTTCGCCTACGTAGATGAAGGTTCGACAAAAAACGTTGTATTTGATCTTCCGCGTACAGTACA
AGAATTTATTAATTATGATGTTATCGAAGCACTGAAGGATAGAGTAATCGAGAGTACAAAATACAAGCCTGTGAAGTATTTAGAATTGAATACTGTACATGTAATAGTTATGGCTAATTT
TCTTCCTGATATGTGTAAAATATCTGAAGATCGAATAAAAATAGTTGCTTGCTGAACACGCTATGAAATACAACACGCTATGACAAACGGGGAAAAATGAAGAATCGGGGGTTGATTGGG
CTATCCTAACGATTAAGGGCCGCAGGCCCGTCAAGATGGATCCAATAACCCGATAAGAAGGTAAACGGGTCTAAAACGATTTCTTCGCCCGCAAGCAACACCTTTAACCTCTGCGCACCT
ATATATAGCGGAGGTGGGCTAG

BBTA2def
Defective no-Rep variant of BBTA2, best match to EU430730 89% identity; MG545616 89% identity; NC_038953 89% identity

Defective muscarsatellite BBTA2 – **no Rep protein** (Rep ORF is lost through several short and long indels and multiple SNPs; CRM and CR-SL are highly similar to those of BBTA2_alyu29)

>BBTA2def_alyu29
TATTACCCACCTCCGCGCACCACCTCTGCGCACCTATAAAAGGATTTGTCTCGATGAAAATTTGCGCTTCATCATTCCGACGCAACGGAGCGAGGCATATTCCTCGCGACTTCGAAGGAG
GAAGATGTGGACGACGCCGTCGTCGGCGACGAATCTGCTCCGACCACTGGTCGGAAACATCTTCAAGGATATCTTCCTTGAAGACCGGATCAGATTACGACAACAAGGCGTTCTGCTCCA
AAGAAGCCCTAATTCTTGAATTAGGGCTTTTATGCCAAACAGGATCGAAAAGCGTAAATTATCAGATATGGATACAAGATCGCCGGAACGAATGAAAATTGAACAGCCAGAGATTATTCA
CCGATGCAATCGGTGAAGAAGATGAAAGTATTCAAAGAAAGGTATGTCTATCCTATCCTCGATAGGTCATGGCAGGTACAATTAACGGAGTTAATTGAAGCAGAACCTGATGATCGAACG
ATCATCTGGGTATTCGGATCAAAAGGGAATGAAGGCAAATCAACGTATGGAAGGCAATAATCCAAAAGGATTGGATCTACACCAGGGGAGGAAAGAAGGAGAACATATTGTTCGCCTACG
AAGATGAAGGATCAATCAAAAACGTTGTATTTGATCTTCCGCGTATTGTACACGAATTAATTAATAATGATATTATCGAGGCATTGAAGGATAGTGTAATCGAGAATATAAAATACAAAC
CTGTGAAGTATTTGGAGATGAATACTGTACATGTAATAGTTATGGCCAAATTTCTTCTTGATATGTGTAAAATATCTGAAGATCGAATAACATGTTGTTGCTTGCAGAACACGCTATGAA
ATACAACGCGCTATGACAAAAGGGGAAAAATGAAGAATCGGGGGTTGATTGGGCTATCCTAACGATTAAGGGCCGCAGGCCCGTTAAGATGGATCCGATAACCCGATAAGAGGATCCGAT
AACCTAATAAGCGTGTGAACCGGGTCTAAAACGATTGCTTCGCCCGCAAGCAACAACTTTAACCTCTGCGCACTATATATAGCGGAGGTGGGCTAG

BBTA3
Best match to BBU12586 ca 100% coverage ca. 83% identity; NC_038955 ca 100% coverage 82% identity; GU074392 ca 100% coverage 82% identity; AF216221 ca 100% coverage 79% identity

Rep protein best matches to ADV18492 100% coverage 87% identities 96% positives; YP_009508283 100% coverage 86% identities 96% positives, ACJ36781 100% coverage 85% identities 94% positives

>BBTA3_alyu29
TATTACCCGCTTCCTCGCCCCTCTTCCTCGCCCCAGTGCAAAGCACGTGATGTCTTCCTTTAAATGGTGCTTCACGCTGAATTACTCAAACGCAACAGAGAGAGAAGACTTTCTCTCTCT
TTTGAAGGAGGAGGACCTGCGCTACGCAGTAGTGGGAGACGAAGTCGCCCCGTCAACTGGACAGAAGCACCTACAGGGATATATATCCCTAAGGAAGAGTATGAAACTGGGTGGTTTGAA
GAAAAAATACTCAGCGAAGGCACACTGGGAGAAGGCGAAAGGATCAGATGAAGATAATACAAAGTATTGTTCTAAGGAAACCCTAATTCTAGAATTAGGGGTTCCTACATCCCAGGGTTC
AAACAAGCGTAAGCTTTCAGAGATGGTTACTAGATCACCGGAACGCATGAGGATAGAACAGCCAGAGATATATCACAGATATCAATCTGTGAATAAGTTAAAACTATTCAAAGAGGAATT
CGTTCATCCTTGCCTCGATAGACCTTGGCAGATACAATTGACGGAGGCAATTGAAGAGGAACCAGATGATCGAAGCATCATCTGGGTCTATGGACCGAATGGCAATGAGGGGAAATCAAC
ATATGCGAAGTCTCTGATAAAACATGATTGGTTCTATACAAGGGGAGGCAAGAAGGAGAACATACTGTTCTCCTACGTAGACGAAGGGACTACGAAGCATGTTGTGTTCGACATACCAAG
ATGTCATCAAGAATATCTAAATTATGATGTAATCGAGGCATTGAAGGATAGGGTGATCGAGAGCACTAAGTATAAGCCGATTAAAATAATCGAGTTGAATAGGGTACATGTAATTGTAAT
GGCTAACTTCATGCCAGAGTACTGTAAGATCTCTGAGGATCGAATAAAATTAATATTTGCATAGCGCACACTATGACAAAAGGGCAAAAATGCAAAATCGGGGGTTGATTGGGCTATCCT
ACCGAtTAAGGGCCGCAGGCCCGTTAAGATGAATAAATCCAAACCCGTTAACCCGGTATCCGATTACTTCGGGACGAAGGAATCACTTTAAGTTCCTCGCCCCTATATATAGTTTTGCCC
GAGGAAGCGGCCTAG

DNA-C
21-25 SNPs to KY427060 MF039864 MF039876 MF039870 (**Thailand**). **27 SNPs** to KM607098 KM607099 (**China**)

C protein (161 aa): 1 aa to KY427060 (**Thailand**) **2 aa** to KM607098 KM607099 (**China**) MF039864 (**Thailand**)

>C_alyu29

TATTACCCCCCCTGCTCGGGACGGGACATCACGTGCATCTAACAAATGCACGTGACAAAGCAGTAGCTTGTAGCGAAAGATAACCATCACTATCACAAAAGTGAGAGGAATATTCGTTGC
TTAGGGACTAAGCAACGTGGACAGATATTTGTTCGAGAAGCGACAATGGAGGCTATTTAAACCTGATGGTTTTGTGATTTCCGAAATCACTCATCGGAGCAGAAATGGAGTTCTGGGAAT
CGTCTGCCATGCCAGACGATGTCAAGAGAGTGATCAAGGAAATATATTGGGAGCATCGAAACAAACTTCTGTTCTGTCAGAAGTTGAAAGGCTGTGTAAGAAGGATTCTGGAGCATGGAA
ATCAAGATGATGCTCTAGACGCAGTGAAGGAGATGAAGACTTCTATTATTCGCTATAGCGAATATTTGAAGAAACCTTGTGTGGTATTGTGTTTTAGTACTAATAAATCTATTGCATATA
GGTTAAACACAATGGTATTCTTTTATCATGAATATATTGAGGACCTAGGAGGAGACTACTCGGTATATCAAGATCTGTTCTGTGATGAGGAACTTCCTTATTCGTCGACAGAAGAAGAAG
ATGAAGAAGTAATATACAGGAATGTTATCATGGCATCGACAGAAGAGAAGATCTCTTGGAGTGAATGTCAAAAGATAGTTATATCAGATTATGATGTAACATTACTGTAATGAAAATCCA
TTATCATAAATAAAATAATGGAATGATGATTATGTATTCTAGTATAAATACATAATGGTATACGTATAGCATAAAATACATATACCAACATACAACACGCTATGACAAACAGGGAAAAAT
GAAGAATCGGGGGTTGATTGGGCTATCTTAACGATTAAGGGCCGAAGGCCCGTTTAAATATGTGGTGGACGAAGTCCACAACACATAAAAAGTGATCAGAACAATGGAAAATAATGAGCT
GGCAAGGTAGGGACCATGTCCCGAGTTAGTGCGCCACGTGAGCAGGGGGGCTTAT

DNA-M
14-18 SNPs to MF039865 KY427061 MF039871 MF039876 (**Thailand**). **53 SNPs** to KM607239 (**China**)

M protein (117 aa): 1 aa to KC581796 (**Thailand**) **2 aa** to MF039871 MF039865 MF039877 KY427061 (**Thailand**)

>M_alyu29
TATTACCCCCAGCGCTCAGGACGGGACATCACGTGCAACTAACAAATGCACGTGACTGATATATACTACATAACGGTTTATTGAACCGTTATATATTGTTGTAACGCAAAGTCACGTGTG
ATAGCGAAATGCACGTGACTAAGTCAAATGTATTGAATAAACATTTGACGTCCGGATGCTTCCTACGGAAGCCATGGTTGCTTCGTGGCGAAGCAAAACATTTATATATTGGCATGGACT
GCTGCCTATAAATAGGCACGCAGGGAAATGGCATTAACAACAGAGCGAGTGAAACTATTCTTTGAATGGTTTCTGTTTATTGGTGCAATATTTATTGCGATAACAATATTATACATATTG
TTGGTGTTGCTCTTTGAGGTCCCCAAGTATATTAAGGATGTTGTGAGGTATCTCGTAGAATACCTGACCAGACGACGTGTATGGATGCAGAGAACGCAGTTGACGGAGGCAACAGGAGAT
GTAGAACTCGGCAGAGGTAGTGTGGAAGACAGACGGGATCAACAACCGGCTGTCATACCACATGCAACACAGGTTATCCCTTCGCATCAACCTAGAAGGGATGAGCAAGGAAGACGAGGA
AACGCCGGACCGATGTTTTAATACACGGTATTGTAATATACGAAATATAAATGGATAATGATAAGTATTGTGAAACATAATATATGTGAAACATAATATATGTTTGATAATTTACATATT
GTAATATGTGAATTGTATACGAGTGTTGTATTTATAAAACATACAACACGCTATGACAAACAGGGAAAAATGCAGAATCGGGGGTTGATTGGTCTATCGTATCGCTTAAGGGCCGCAGGC
CCGTTGAAATGATTCTGTATAAAACAAATATACATGATACGGATTGTTGAAAATATAATCAACTATGTATAAATACAACAGAATGTTGTATACTAATTAAAATAATGAGAAGATAAGTAT
ATTTGTGAAGGATAAGGATCACAACCACCACTTTAGTGGTGGGTCATATGTCCCGAGTTAGTGCGCCACGTAAGCGCTGGGGCTTAT

DNA-N
22-26 SNPs to MF039872 MF039878 MF039866 KY427062 (**Thailand**). **27 SNPs** to KM607387 KM607385 (**China**)

N protein (154 aa): 0 aa to MF039872 MF039878 KY427062 (**Thailand**) KM607387 KM607385 (**China**) **1 aa** to MF039866 (**Thailand:**)

>N_alyu29
TATTACCCCCGTGCTTGGGACGGGACATGACGTCAGCATAGATTATAATGGGCCAACTAAAGGCCCATTTAATAGAATAGGCGGGCTTTTGACATATTTCAAAGGCCCAGACTGGATGT
GGATAATGTCACGTGCCTAATAAGAGGTTGCTTCGCCTCGAAGCAATCCGATTAAATGTTGCGTATTCAATACGCAACTGAAGTCTATTAATATGGATGTCTCTGCCGAATAAATCAGAG
CGAAAGCGAAGCAGAAGCGATGGATTGGGCAGAATCACAATTCAAGACATGTACTCATGGGTGCGATTGGAAGACGATATCATCGGATTCATCCGAGAATCGACAATATGTACCGTGCGT
CGACGCTGGCAGCGGAAGAAAGTCACCTCGCAAGGTACTGCTAAGATCTATTGAAGCTGTGTTTAATGGAAGCTTCAAAGGAAATAACAGGAATGTTCGTGGGTTTATATACGTATCAAT
ACGAGACGATGATGGAGAAATGCGTCCAGTACTCATAATACCATTCGGAGGATATGGATATCATAATGATTTCTATTATTTCGAAGGGAAGGGGAAAGTTGAATGTGATATATCATCAGA
TTATGTAGCGCCAGGAGTCGATTGGAGCAGAGACATGGAAGTTAGTATTAGTAACAGCAACAACTGTAATGAATTATGTGATCTGAAGTGTTATGTTGTTTGTTCGTTAAGAATAAAGGA
ATAACAGATGTGCTGTAATGATTATTAATAAAACATATTTTCATGTAATTGATAGTTGTATAAAACATACAACACGCTATGACAAACAGGGAAAAATGAAAAATCGGGGGTTGATTGGTC
TATCGTATCGCTTAAGGGCCGCAGGCCCGTTGAAAAATAATAATCGAATTATAAACGTTTGATAATAATCAGAGATAGATGATAAGCATCCAAAAACATAGACGAAGTATATGGCTGTAT
AATATAAAAGAAGCATATAAATAAAATATGTAAACTAATCTCTGATTGGTTTAGAATGAAACCCCACCAACTTTATGTTGGTGGAAATGTCCCGATGACGTAAGCACGGGGGACTAT

DNA-R
10 SNPs to MF039879 (**Thailand**). **12 SNPs** to MF039873 (**Thailand**) KM607678 (**China**). **13 SNPs** to MF039867 (**Thailand**) KM607677 (**China**)

R protein (286 aa): 0 aa to KM607676 KM607677 KM607679 (**China**) **1 aa** to MF039879 (**Thailand**) AF416464 AB113660 (**Vietnam**)…

>R_alyu29
TATTACCCCCAGCGCTCGGGACGGGACATTTGCATCTATAAATAGACCTCCCCCCCCTCCACTACAAGATCATCATCGTCGACAGAAATGGCGCGATATGTGGTATGCTGGATGTTCACC
ATCAACAATCCCGCTTCACTACCAGTGATGCGGGATGAGTTCAAATATATGGTATATCAAGTGGAGAGGGGACAGGAGGGTACTCGTCATGTGCAGGGATACGTCGAGATGAAGAGACGA
AGTTCTCTGAAGCAGATGAGAGGCTTCTTCCCTGGCGCACACCTTGAGAAACGAAAGGGAAGCCAAGAAGAAGCACGGGCATACTGTATGAAGGAAGACACAAGAATCGAAGGTCCCTTC
GAGTTTGGTGCCTTTAAATTGTCATGTAATGATAATTTATTTGATGTCATACAGGATATGCGTGAAACGCACAAACGGCCTTTGGAGTATTTATATGAGTGTCCAAATACCTTCGATAGA
AGTAAGGATACATTATACAGAGTTCAAGCAGAGTTGAATAAAACGAAGGCGATGAATAGCTGGAAAACATCCTTCAGTTCCTGGACATCGGAAGTTGAAAATATTATGGCGGAGCCATGT
CATCGAAGGATAATTTGGGTCTATGGCCCAAATGGAGGAGAAGGAAAGACAACTTATGCAAAATATTTAATGAAGACGAAGAATGCGTTTTATTCGCCAGGAGGAAAATCATTGGATATA
TGTAGATTGTATAATTATGAGGAAATAGTTATATTTGATATTCCTAGATGCAAAGAGGAATATTTAAACTATGGTTTATTAGAAGAATTTAAAAATGGAATTATTCAAAGCGGGAAATAT
GAACCCGTTTTGAAAATTGTAGAATATGTTGAAGTCATTGTAATGGCAAACTTCCTTCCGAAGGAAGGAATCTTTTCTGAAGATCGAATAAAGCTAGTTGCTTGCTGAACACGCTATGAC
AATCGTACGCTATGACAAAAGGGGAAAAGCAAAGAATCGGGGGTTGATTGGGCTATCCTAACGATTAAGGGCCGCAGGCCCGTCAAGATGGACGGTTTGATCAGATGTCCCGAGTTAGTG
CGCCACGTAAGCGCTGGGGCTTAT

DNA-Rdef
Defective derivative of DNA-R: deletion in Rep ORF (nts ca. 250-750), followed by SNP mutations.

Protein 57 aa: a truncated Rep/mutated N-terminal part of Rep protein

>Rdef_alyu29
TATTACCCCCAGCGCTTGGGACGGGACATTTGGGTCTATAAATAGACATCCCCCCTCTCCACTACAAGATCAACATCGTCGACAGAAATGGCGCGATGGGCGGAATGCTGGATCGTCAAC
ATCAACAATCCCGCTTCTCTACCAGTGATGCGGGATGAGATCAAGGATATGGTATATCAAGAGGAGAGGGGAAAGGATGGAACTTGTCATGTCCAAGGATACATCGAGATGAAGAGACGA
AGTTCTCTGAAATCTCTGTAATGCCTAACTTCCTTCCGAAGGAAGACGAAGTTCTCTGACGTCATTGTACTGGATTACTTCCTTCCGAAGGAAGGAATCTTTTCTGAAAAAACGAATAAA
GCTAGTTGCTTGCTGAACACGCTATGACAATCGTACGCTATGACAAAAGGGGAAAAGCAAAGATTCGGGGGTTGATTGGTCTATCCTAACGATTAAGGGCCGCAGGCCCGTCAAGATGGA
CGGCTTGATCAAATGTCCCGAGTTAGTGCGCCACGTAAGCGCTGGGGCTTAT

DNA-S
16 SNPs to AB113662, AB113661 (**Viet Nam**). **17 SNPs** to MF039874 (**Thailand**). **19-20 SNPs** to KM607535 KM607534 (**China**). **23 SNPs** to KY427064 (**Thailand**)

S protein (170 aa): 0 aa to AY337715 AY494786 HQ378191 (**China**) FJ787433 FJ787434 (**Philippines**) **1 aa** to several isolates

>S_alyu29
TATTACCCCCAGCGCTCGGGACGGGACATGGGCTAATGGATCATGGAATAAGGGCCCATCGGGCCCGTTAAGATGGGTTTTGGGCTTATGGGCTCTATCCAGAAGACCAAAAACAGGCGG
GAAACGTCCCAAATTCAAATTTAGATAGCTTGCCCCGCAAGCAAACTAAAAGTCTATATATACCAGTGTAGACATATTGTTCAGATAACAAATGGCGAGGTTTCCGAAGAAATCGATCAA
GAAGAGGCGGGTTGGGCGACGGAAGTATGGAAGCAAGGCGGCAACCAGTCACGACTACTCGTCGTTAGGTTCAATATTGGTTCCTGAAAATACCGTCAAGGTATTTAGGATTGAGCCTAC
TGATAAAACATTACCCAGATATTTTATCTGGAAAATGTTTATGCTTTTGGTGTGCAAGGTGAAGCCCGGAAGAATACTTCATTGGGCTATGATTAAAAGTTCATGGGAAATCAACCAGCC
GACTACATGTCTGGAAGCACCAGGTTTATTTATAAAACCTGAACATAGCCATCTGGTGAAACTGGTATGCAGTGGGGAACTTGAAGCCGGAGTCGCAACAGGGACATCAGATGTTGAATG
TCTTCTAAGAAAGACAACCGTGTTGAGGAAGAATGTAACAGAGGTGGATTACTTGTATTTGGCATTTTATTGTAGTGCTGGAGTTAGTATTAACTACCAGAACAGAATTACATATCATGT
ATGATATGTTTATGTAAACATAAACCTTTGTATGGAATAATGACCAAATAACATACAACACGCTATGAAATACAACACGCTATGACAAACAGGGAAAAATGAAGAATCGGGGGTTGATTG
GTCTATCGTATCGCTTAAGGGCCGCAGGCCCGTTGAAATGATTCTTTATTAAACAAATATACATGATACGGATTGTTGAAAATATAATCAACGATGTATAAATACAACAGAATGTTGTAT
ACTAATTAAAATAATGAGAAGAAAAGAATATTTGTGAAGGATAAGCATCAGAACCACCACTTTAGTGGTGGGTCATATGTCCCGAGTTAGTGCGCCACGTAAGCGCTGGGGCTTAT

DNA-U3
34-42 SNPs to MF039881 MF039875 KY427065 MF039869 (**Thailand**)

U3 protein (39 aa): 1 aa to KY427065 (**Thailand)**

>U3_alyu29

TATTACCCCCAGCCCCCAGGACGGGACATGGGCTTTTTAAATGGGCCGAGAGAGTTTGAACAGTTCAGTATCTTCGTTATTGGGCCATCTGGCCCAATAATTAAGAGAACGTGTTCAAAA
TCTGGGTGTGACCGAAGGTCAAGGTAGACGGTCAACAATATTCTGGCTTGCGGAGCAAGCAACACGAATTAAATATCTAATTCGTAAGACACGTGGACGGACCGAAATACTCCTGTATCT
CTATAAATAGCTTAAATCTGGCTTGGATAATTACTCTCGCTCTTCTGTCAAAGCTGTTGTGTTGAGGCGGAAGATCGCCATCGGCGATCATCAGACGAAAAGCTGCAAGAGAGACGGAGA
ACCATGCTGCGAAGCGTATATCGGGTATTTATAGACTTCTAGCGCAGCTAGAAGTTTCCTTGTACTTGATATTGTATTTTGTAAATcACGAAGAAATTCGTACATTGATAATAATAAAAC
ATCTGGGATTGTTAATGTTTACATTAACCAGTATTAAAAATGTACAATAACAAATATTGTATGAGGAACGAATACAATAATATTAAAATATGAGGAGCGTAGCGTGATAAACAGGTGTTT
AAGGTATAATTAAATTAATTATGCAATGTCATGATAATACGGTATAAGTTGAAGTATGAGGTGAAAGAGGAGATATTAGAATATTAAAAACCCAATTATATTATTTATAATACATACAAC
ACGCTATGACAAAAGGGGAAAAAAGAAAAATCGGGGGTTGATTGGTCTATCCTAACGATTAAGGGCCGCAGGCCCGTCAAGATGGATGAACGGTTAGATTTGATTGCTTAGCCACGAAGA
AAGGAATCTTTTGTGGGACCACAGACAAGACAGCTGTCACTACTTTTTAAAATAATATAATAACCAATGGACGATACTACCCCTCCCAAAGATGACGTATAGGGGTGTCCCGAGGTAATT
TAACATAGCTCTGACAAGAGATAGTGGACGTTGGATGCCACGATCGGACGATCGTGGTTTGATGAACTAATGATGACGTAGGGGCTGGGGCTTAT

ALYU-30 - Phyllanthus sp. - Vietnam

Neighbor to Musa ALYU-29

Virome
"**Virus free**": however, low titre infection with BBTV and alphasatellites cannot be ruled out 100%, because of somewhat higher coverage with viral reads of a concatenated reference virome genome, compared to other "virus free" samples (see Supplementary Figure S3C)

ALYU-31 - Arachis hypogaea - Vietnam

Neighbor to ALYU-29

Virome
"**Virus free**": Reads representing BBTV genome components and alphasatellites are at a cross-contamination level (see Supplementary Figure S3D).

ALYU-32 - Musa sweet banana - Vietnam

Virome
1. **BBTV** six components
2. **BBTA5** alyu32 – new alphasatellite named here Banana bunchy top alphasatellite 5, genus *Banaphisatellite*
3. **BBTA2** alyu32 – Banana bunchy top alphasatellite 2, genus *Muscarsatellite*
4. **Babu2def** alyu32 164 nt circle (CR-SL)

BBTA5
Best matches to MF510475 MF510474 73% coverage 66.5% identity Faba bean necrotic yellows virus associated alphasatellite 2; MK291270 72% coverage 67.2% identity Sophora yellow stunt alphasatellite 3; AJ132187 AJ005966 74% coverage, 66.3-65.8% identity Faba bean necrotic yellows C9 alphasatellite

Rep protein best matches to QBF29185 100% coverage 63% identities 80% positives Sophora yellow stunt alphasatellite 3; ATU31571 100% coverage 62% identities 80% positives Faba bean necrotic yellows virus associated alphasatellite 2 ARI50297 100% coverage 62% identities 80% positives Sophora alopecuroides yellow stunt alphasatellite 3; NP_619574 100% coverage 62% identities 80% positives Faba bean necrotic yellows C9 alphasatellite

>BBTA5_alyu32
TATTACCCGACCTTGGCTCACTGGCTCACTATAAAAGGGGGAAATGTCGCAGCAGCGAAATTGGGTATTCACGCGTAACTTCTCCGGCGACCCGCCGATTTTAAAGTTCGCCGGAGAAAC
TCAATACGCGTGCTGGCAACATGAGAAGAAGAATCACGACCATCTTCAGGGAGTGATTCAATTGAAGAAGAAGCTCCGGATGAACGCAGTGAAGACGTTAATTGGGGAAATCCACACCT
CGAAGCCATGAGAGGAACAATCGACGAAGCAATAAGGTACGTGACGAAAGAAGAGACACGAGTAGCAGGTCCGTGGGAATTCGGAGAATTACTACGAAAAGGATCTCATAAGAGGAAATT
GATGGAATTGCTCGACGATCCAGATAACGAAATAATGGAACCTCAAAAATATCGACGCGCCATTACTAAACAAGCAATGGACGCGTCAAAGAAGAAGGCGGAATTAGGGTTTCCTTACGA
CTTAAAGGAGTGGCAAAAGATGGTTATTGAGTTAATCGAAGAACAACCAGACAACAGAACTATTATCTGGGTCTATGGACCTAATGGTGGAGAAGGTAAAACGCAGTTCGCAAAGCATTA
TGGATTACTCAAAGGATGGACATATCTACCAGGTGGTGAACTGAAAGACATGATGTATCTGTTGGCAAAAGAAATTAATAATAACGTTATTATTGATTTCCCCCGATGTACAAAGGATTT
CATATCCTATAAGTTTATTGAGATGGTAAAGAACCGATGCATATTTAGTTATAAATATGAACCAATCGGGGCGATTGTAAGTAATGAAGTCCATGTAATTGTAATGTCGAATGAGCTTCC
AGATTATTCAAAAATTTCGGAAGACAGAATAAAATTAATATTTGCATAACGCACACTATGACAAAAGGGCAAAAATGCAAAATCGGGGGTTGATTTGTCTATATTTACGAATAAGGGCCG
CAGGCCCGTACAAATTGCGCGGGAAATTCAAATTCGGACGCCAAATATTATTAAATACAATACACCGCCTTTTAGAAATCCGTGGACGAGAAGATGCCACGTAGCAAAACACTGTACTCC
TTGGCTCACTATAAATACCTGAGCCAAGGTCGGGCATAG

BBTA2
Best match to EU430730 100% coverage 97% identity; MG545616 100% coverage 97% identity; NC_038953 100% coverage 95% identity

Rep protein best matches to AZL93963 100% coverage 99% identities (2 aa) 99% positives; ACB86656 100% coverage 99% identities (4 aa) 99% positives; YP_009508281 100% coverage 93% identities (11 gaps in middle) 94% positives *Banana bunchy top alphasatellite 2*

>BBTA2_alyu32
TATTACCCACCTCCGCGCACCACCTCCGCGCACCTATAAAATGTCTGCCTCTCGCTGGACATTTACGCTTCATTATTCCGACGCAACGGAGCGAGGCAAATTCCTCGCGACTTTGAAGGA
GGAAGATGTGCACTACGCCGTCGTCGGCGACGAAACTGCTCCGAATACAGGTCGAAAACATCTTCAAGGATATCTTTCCTTGAAGAAACGTTTTCGTATTAGCGGAATAAAGAAGAAATA
TTCGTCGAGAGCGCATTGGGAGAAAGCTCGAGGATCAGATTACGACAACAAGGCGTACTGTTCCAAAGAAGCCCTAATTCTTGAATTAGGGGTTCCTTGCCAAACAGGTTCGAATAAGCG
TAAATTAGCAGATATGGTTACAAGATCTCCGGAACGAATGAAAATTGAACAGCCAGAGATATTTCACCGATACCAATCGGTGAAGAAGATGAAAGAATTCAAAGAAAGGTATGTCTATCC
TAACCTCGATAGGCCATGGCAGGTACAATTAACGGAGTTAATTGAAGCAGAACCTGATGATCGAACGATCATCTGGGTATTCGGACCAAAAGGGAATGAAGGCAAATCAACGTATGCGAA
GTCATTAATCCAAAAGGATTGGTTCTACACAAGGGGAGGAAAGAAGGAGAACATACTGTTCGCCTACGTAGATGAAGGTTCGACCAAAAACGTTGTATTTGATCTTCCGCGTACAGTACA
AGAATTTATTAATTATGATGTTATCGAAGCATTGAAGGATAGAGTAATCGAGAGTACAAAATACAAGCCTGTGAAGTATTTAGAATTGAATTCTGTACATGTAATAGTTATGGCTAATTT
TCTTCCTGATATGTGTAAAATATCTGAAGATCGAATAAAAATAGTTGCTTGCTGAACACGCTATGAAATACAACACGCTATGACAAACGGGGAAAAATGAAGAATCGGGGGTTGATTGGG
CTATCCTAACGATTAAGGGCCGCAGGCCCGTCAAGATGGATCCAATAACCCGATAAGAAGTTAAACGGGTTTAAAATGATTCCTTCGCCCGCAAGCAACACCTTTAACCTCTGCGCACCT
ATATATAGCGGAGGTGGGCTAG

Babu2def
Defective derivative of BBTA2_alyu32 CR-SL + non-viral DNA

>BBTA2def_alyu32
TATTACCCACCTCCGCGCACAACCTCTGCGCACCTGACGTCACACACACATACAACACACTATGACAAAAGGGCAAAAAAATCATTTCATTATAGGACCCACCAAAAACACTTTAACCTT
CGCGCACTCCTCTCACAAACAGCGACGCGCGGAGGGTGGGCTAG

DNA-C
30-36 SNPs to KY427060 MF039864 MF039876 MF039870 (**Thailand**). **35 SNPs** to KM607098 (**China**)

C protein (161 aa): 2 aa to KY427060 (**Thailand**) **3 aa** to KM607098 KM607099 (**China**) MF039864 (**Thailand**)

>C_alyu32

TATTACCCCCCCTGCTCGGGACGGGACATCACGTGCATCAAACAAATGCACGTGACAAAGCAGTAGCTTGTAGCGAAAGATAACCATCACTATCACAAAAGTGAGAGGAATATTCGTTGC
TTAGGGACTAAGCAACGTGCTCAGATAATTGTTCGAGAAGCGACAATGGAGGCTATTTAAACCTGATGGTTTTGTGATTTCCGAAATCACTCATCGGTGCAGAAATGGAGTTCTGGGAAT
CGTCTGCCATGCCAGACGATGTAAAGAGAGTGATCAAGGAAATATATTGGGAGCATCGAAACAAACTTCTGTTCTGTCAGAAGTTGAAAGGCTGTGTAAGAAGGATTCTGGAGCATGGAA
ATCAAGATGATGCTCTAGACGCAGTGAAGGAGATGAAGACTTCTATTATTCGCTATAGCGAATATTTGAAGAAACCATGTGTGGTATTGTGTTTTAGTTCTAATAAATCTATTGCATATA
GGTTAAACACAATGGTGTTCTTTTATCATGAATATATTGAGGATCTAGGAGGAGACTACTCGGTATATCAAGATCTGTTCTGTGATGAGGAACTTCCTTATTCGTCGACAGAAGAAGAAG
ATGAAGAAGTAATATACAGGAATGTTATCATGGCATCGACAGAAGAGAAGATCTCTTGGAGTGAATGTCAAAAGATAGTTATATCAGATTATGATGTAACATTACTGTAATGAATATCCA
TTATCATAAATAAAATAATGGAATGATGATTATGTATTCTAGTATAAATACATAATGGTATACGTATAGCATAAAATACATATATCTACATACAACACGCTATGACAAACAGGGAAAAAT
GAAGAATCGGGGGTTGATTGGGCTATCTTAACGATTAAGGGCCGAAGGCCCGTTTAAATATGTGGTGGACGAAGTCCACAACACATAAAAAGTGAGCAGAACAATGGAATATAATGAGCT
GGCAACGTAGGGTCCATGTCCCGAGTTAGTGCGCCACGTGAGCAGGGGGGACTAT

DNA-M
29-34 SNPs to KM607239 KM607240 (**China**). **48-51 SNPs** to KY427061 MF039865 MF039876 MF039871 (**Thailand**)

M protein (117 aa): 0 aa to KC581796 (**Thailand**) **1 aa** to MF039871 MF039865 MF039877 KY427061 (**Thailand**)

>M_alyu32
TATTACCCCCAGCGCTCAGGACGGGACATCACGTGCAACTAACAAATGCACGTGACTGATATATACTACATAACGGTTTAACGAACCGTTATATAATGTTATAACGATAAGTCACGTGTG
ATAGAGACATGCACGTGACATAGTCAAATGTATTGAATAAACATTTGACGTCCGGATGCTTCCGACGGAAGCTTAGGTTACTTCGTGGCGAAGCAAAACATTTATAAAATTGCCTGGATT
GCTGCCTATAAATAGGCAAGCAAGGAAATGGCATTAACAACAGAGCGAGTGAAACTATTCTTTGAATGGTTTCTGTTCATCGGTGCAATATTTATTGCGATAACAATATTATATATATTG
TTGGCGTTGCTCTTTGAGGTCCCCAAGTATATTAAGGATGTTGTGAGGTATCTCGTAGAATACCTGACCAGAAGACGTGTATGGATGCAGAGAACGCAGTTGACGGAGGCAACAGGAGAT
GTAGAGCTCGGCAGAGGTAGTGTGGAAGACAGACGGGATCAACAACCGGCTGTCATACCACATGCAACACAGGTTATCCCTTCGCATCAACCTAGAAGGGATGAGCAAGGAAGACGAGGA
AACGCCGGACCGATGTTTTAATACACGGTATTGTAATATACGAAATATAAATGGATAATGATATGTATTGTGAAACATAATATATGTGAAACATAATATATGTTTGATAATTTACATATT
GTAATATGTAAAATGTATACGAGTGTTGTATTTATAGAACATACAACACGCTATGACAAACAGGGAAAAATGAAGAATCGGGGGTTGATTGGTCTATCGTATCGCTTAAGGGCCGCAGGC
CCGTTGAAATGATTCTTAATAAGACAAATATACATGATACGGATAATTGAATATATAAACTACGAAGTATAAATACAACAGAATGTTGTATACTAATTAAAATAATGAGAAGATAAGTAT
ATTTGTGAGGGATGATGATCACAACCACCACTTTAGTGGTGGGTCATATGTCCCGAGTTAGTGCGCCACGTAAGCGCTGGGGCTTAT

DNA-N
17-21 SNPs to MF039872 MF039878 MF039866 (KY427062 (**Thailand**). **22 SNPs** to KM607387 KM607385 (**China**). **33 SNPs** to KM607386 (**China**)

N protein (154 aa): 0 aa to MF039872 MF039878 KY427062 (**Thailand**) KM607387 KM607385 (**China**) **1 aa** to MF039866 (**Thailand**)

>N_alyu32
TATTACCCCCCGTGCTTGGGACGGGACATGACGTCAGCATAGATTATAATGGGCCAACTAAAGGCCCATTTAATAGAATAGGCGGGCTTTTGACATATTTCAAAGGCCCAGCCTGGAAGT
GGATAATGTCACGTGCCTAATAAGAGGTTGCTTCGCCTCGAAGCAATCCGAATAAATGTTGCGTATTCAATACGCAACTGAAGTCTATTAATATGGATGTCTCTGCCGAATAAATCAGAG
CGTAAGCGAAGCAGAAGCGATGGATTGGGCAGAATCACAATTCAAGACATGTACTCATGGGTGCGATTGGAAGACGATATCATCGGATTCATCCGAGAATCGACAATATGTACCTTGCGT
CGACGCTGGCAGCGGAAGAAAGTCACCTCGCAAGGTACTGCTAAGATCTATTGAAGCTGTGTTTAATGGAAGCTTCAAAGGAAATAACAGGAATGTTCGTGGGTTTATATACGTATCAAT
ACGAGACGATGATGGAGAAATGCGTCCAGTACTCATAATACCATTCGGAGGATATGGATATCATAATGATTTCTATTATTTCGAAGGGAAGGGGAAAGTTGAATGTGATATATCATCAGA
TTATGTAGCGCCAGGAGTCGATTGGAGCAGAGACATGGAAGTTAGTATTAGTAACAGCAACAACTGTAATGAATTATGTGATCTGAAGTGTTATGTTGTTTGTTCGTTAAGAATAAAGGA
ATAACAGATGTGCTGTAATGATTATTAATAAAACATATTTTCATGTAATTGATAGTTGTATAAAACATACAACACGCTATGACAAACAGGGAAAAATGAAAAATTGGGGGTTGATTGGTC
TATCGTATCGCTTAAGGGCCGCAGGCCCGTTGAAAAATAATAATCGAATTATAAACGTTTGATAATAATCAGAGATAGATGATAAGCATCCAAAAACATAGACGAAGTATATGGCTGTAT
AATATAAAAGAAGCATATAAATAAAATATGTAAACTAATCTCTGATTGGTTTAGAATGTAACCCCACCAACTTTATGTTGGTGGAAATGTCCCGATGACGTAAGCACGGGGGACTAT

DNA-R
15 SNPs to AF416475 (**Viet Nam: Hue region**). **16 SNPs** to MF039879 (**Thailand**). **16-18 SNPs** to KM60767 KM607678 KM607676 KM607679 (**China**). **20-1 SNPs** to MF039873 MF039867 (**Thailand**)

R protein (286 aa): 0 aa to KM607676 KM607677 KM607679 (**China**) **1 aa** to MF039879 (**Thailand**) AF416464 AB113660 (**Vietnam**)…

>R_alyu32
TATTACCCCCAGCGCTCGGGACGGGACATTTGCATCTATAAATAGACCTCCCCCCTCTCCACTACAAGATCATCATCGTCGACAGAAATGGCGCGATATGTGGTATGCTGGATGTTCACC
ATCAACAATCCCGCTTCACTACCAGTGATGCGGGATGAGTTCAAATATATGGTATATCAAGTGGAGAGGGGCCAGGAGGGTACTCGTCATGTGCAGGGATACGTCGAGATGAAGAGACGA
AGTTCTCTGAAGCAGATGAGAGGCTTCTTCCCTGGCGCACACCTTGAGAAACGAAAGGGAAGCCAAGAAGAAGCACGGGCATACTGTATGAAGGAAGACACAAGAATCGAAGGTCCCTTC
GAGTTTGGTGCCTTTAAATTGTCATGTAATGATAATTTATTTGATGTCATACAGGATATGCGTGAAACGCACAAACGGCCTTTGGAGTATTTATATGAGTGTCCAAATACCTTCGACAGA
AGTAAGGATACATTATACAGAGTTCAAGCAGAGTTGAATAAAACGAAGGCGATGAATAGCTGGAAAACATCCTTCAGTTCTTGGACATCGGAAGTGGAAAATATCATGGCGGAGCCATGT
CACCGAAGGATAATTTGGGTCTATGGCCCAAATGGAGGAGAAGGAAAGACAACCTATGCAAAATATTTAATGAAGACGAAGAATGCGTTTTATTCGCCAGGAGGAAAATCATTGGATATA
TGTAGATTGTATAATTATGAGGAAATAGTTATATTTGATATTCCCAGATGCAAAGAGGAATATTTAAACTACGGTTTATTAGAAGAATTTAAAAATGGAATTATTCAAAGCGGGAAATAT
GAACCCGTTTTGAAAATTGTAGAATATGTGGAAGTCATTGTAATGGCTAACTTCCTTCCGAAGGAAGGAATCTTTTCAGAAGATCGAATAAAGCTAGTTGCTTGCTGAACACGCTATGAC
AATCGTACGCTATGACAAAAGGGGAAAAGCAAAGATTCGGGGGTTGATTGTGCTATCCTAACGATTAAGGGCCGCAGGCCCGTCAAGATGGACGGCTTGATCAGATGTCCCGAGTTAGTG
CGCCACGTAAGCGCTGGGGCTTAT

DNA-S
11/12 SNPs to AB113661 AB113662 (**Viet Nam**). **15 SNPs** to MF039874 (**Thailand**). **18/19 SNPs** to KM607535 KM607534 (**China**). **21 SNPs** to KY427064 (**Thailand**)

S protein (170 aa): 0 aa to AY337715 AY494786 HQ378191 (**China**) FJ787433 FJ787434 (**Philippines**) **1 aa** to several isolates

>S_alyu32
TATTACCCCCAGCGCTCGGGACGGGACATGGGCTAATGGATCATGGAATAAGGGCCCATCGGGCCCGTTAAGATGGGTTTTGGGCTTATGGGCTTTATCCAGAAGACCAAAAACAGGCGG
GAACCGTCCCAATTTCAAATTAGATAGCTTGCCCCGCAAGCAAACTAAAAGTCTATATATACCAGTGTAGACATATTGTTCAGAGAACAAATGGCGAGGTTTCCGAAGAAATCGATCAAG
AAGAGGCGGGTTGGGCGACGGAAGTATGGAAGCAAGGCGGCAACCAGTCACGACTACTCGTCGTTAGGTTCAATATTGGTTCCTGAAAATACCGTCAAGGTATTTAGGATTGAGCCTACT
GATAAAACATTACCCAGATATTTTATCTGGAAAATGTTTATGCTTTTGGTGTGCAAGGTGAAGCCCGGAAGAATACTTCACTGGGCTATGATTAAAAGTTCATGGGAAATCAACCAGCCG
ACTACATGTCTGGAAGCACCAGGTTTATTTATAAAACCTGAACATAGCCATCTGGTGAAACTGGTATGCAGTGGGGAACTTGAAGCCGGAGTCGCAACAGGGACATCAGATGTTGAATGT
CTTCTAAGGAAGACAACCGTGTTGAGGAAGAATGTAACAGAGGTGGATTACTTGTATTTGGCATTTTATTGTAGTGCTGGAGTTAGTATTAACTACCAGAACAGAATTACATATCATGTA
TGATATGTTTATGTAAACATAAACCTTTGTATGGAATAATGACCAAATAACATACAAGACGCTATGAAATACAACACGCTATGACAAACGGGGAAAAATGAAGAATCGGGGGTTGATTGG
TCTATCGTATCGCTTAAGGGCCGCAGGCCCGTTGAAATGATTCTTTATAAAACAAATATACATGATACGGATTGTTGAAAATATAATCAACGATGTATAAATACAACAGAATGTTGTATA
CTAATAAAAATAATGAGAAGAAAAGAATATTTGTGAAGGATAAGCATCAGAACCACCACTTTAGTGGTGGGTCATATGTCCCGAGTTAGTGCGCCACGTAAGCGCTGGGGCTTAT

DNA-U3
27-34 SNPs to MF039881 MF039875 KY427065 MF039869 (**Thailand**)

U3 protein (39 aa): 0 aa to KY427065 (**Thailand)**

>U3_alyu32
TATTACCCCCAGCCCCCAGGACGGGACATGGGCTTTTTAAATGGGCCGAGAGAGTTTGAACAGTTCAGTATCTTCGTTATTGGGCCATCTGGCCCAATAATTAAGAGAACGTGTTCAAAA
TCTGGGTTTGACCGAAGGTCAAGGTAGACGGTCAACAATATTCTGGCTTGCGGAGCAAGCAACACGAATTAAATATTTAATTCGTATGACACGTGGACGGACCGAAATACTCCTGCATCT
CTATAAATAGCTTAAATCTGGCTTGGATAATTGCTCTCGCTCTTCTGTCAAAGATGTTGTGTTGAGGCGGAAGATCGCCATCGGCGATCATCAGACGAAAAGCTGCAAGAGAGACGGAGA
ACCATGCTGCGAAGCGTATATCGGGTATTTATAGACTTCTAGCGCAGCTAGAAGTTTCCTTGTACTTGATTTTGTATTTTGTAAATTACGAAGAAATTCGTATATTGATAATAATAAAAC
ATCTGGGATTGTTAATGTTTACATTAACCAGTATTAATAATGTACAATAACAAATATTGTATGAGGAACGAATACAATAATATTAAAATATGAGGAGCGTAGCGTGATAAACAGGTGTTT
AAGGTATAATTAAATTAATTATGCAATGTCATGATAATACGGTATAAGTTGAAGTATGAGGTGAAAGAGGAGATATTAGAATATTAAAAACCCAATTATATTATTTATAATACATACAAC
ACGCTATGACAAAAGGGGAAAAAAGAAAAATCGGGGGTTGATTGGTCTATCGTATCGCTTAAGGGCCGCAGGCCCGTCAAGATGGATGAACGGTTAGATTTGATTGCTTAGCCACGAAGA
AAGGAATCTTTTTGGGACCACAGATAAGACAGCTGTCATTACTTTTTAAAATAATATAATAACCAAAGGACGATAGTACCCCTCCCAAAGATGACGTATAGGGGTGTCCCGAGGTAATTT
AACATAGCTCTGACAAGAGATAGTGGACGTTGGATGCCACGATCGGACGATCGTGGTTTGATGAACCAATGATGACGTAGGGGCTGGGGCTTAT

ALYU-33 - **Musa ABB Chuoi Ngop** - **Vietnam**

Virome

1. **BBTV** six components
2. **BBTA5** alyu33- new alphasatellite named here Banana bunchy top alphasatellite 5, genus *Banaphisatellite*
3. **BBTA2** alyu33 – Banana bunchy top alphasatellite 2, genus *Muscarsatellite*
4. **BSVNV** alyu33 new isolate of Banana streak VN virus (*Badnavirus*, *Caulimoviridae*)

BBTA5

Best matches to MK291270 72% coverage 67.2% identity Sophora yellow stunt alphasatellite 3; MF510475 MF510474 73% coverage 66.2% identity Faba bean necrotic yellows virus associated alphasatellite 2; AJ132187 AJ005966 73% coverage 66.1-65.6% identity Faba bean necrotic yellows C9 alphasatellite

Rep protein best matches to QBF29185 98% coverage, 63% identities 80% positives Sophora yellow stunt alphasatellite 3; ATU31571 99% coverage 62% identities 80% positives Faba bean necrotic yellows virus associated alphasatellite 2; ARI50297 98% coverage 61% identities 81% positives Sophora alopecuroides yellow stunt alphasatellite 3

```
>BBTA5_alyu33
TATTACCCGACCTTGGCTCACTGGCTCAATATAAAAGGGGGAGATGTCGCAGCAGCGAAATTGGGTATTCACGCGTAACTTCTCCGGCGACCTGCCGATTTTAAATTTCGCCGGAGAAAC
TCAATACGCGTGTTGGCAACATGAGAGGAAGAATCACGACCATCTTCAGGGAGTGATTCAATTGAAGAAGAAGCTTCGGATGAACGCAGTGAAGACGTTAATTGGGGGAAATCCACACCT
CGAAGCCATGAGAGGAACAATCGACGAAGCAAAAAGGTACGTGACGAAAGAAGAGACACGAGTAGCAGGTCCGTGGGAATTCGGAGAATTACTACGAAAAGGATCTCATAAGAGGAAATT
GATGGAATTGCTCGACGATTCAGATAACGAAATAATGGAACCTCAAAAATATCGACGCGCCATTACTAAACAAGCAATGGACGCGTCAAAGAAGAAGGCGGAATTAGGGTTTCCTTACGA
CTTAAAGGAGTGGCAAAAGATGATTGTTGAGTTAATCGAAGAACAACCCGACAACAGAACTATTATCTGGGTGTATGGACCTAATGGTGGAGAAGGTAAAACGCAGTTCGCAAAGCATTA
TGGATTACTCAAAGGATGGACATATCTACCAGGTGGTGAACTGAAAGACATGATGTATCTGTTGGCAAAAGAAATTAACAATAACGTAATTATTGATTTCCCCCGATGTACAAAGGATTT
CATATCCTATAAGTTTATTGAGATGGTAAAGAACCGATGCATATTTAGTTATAAATATGAACCAATCGGGGCGATTGTAAGTAATGAAGTCCATGTAATTGTAATGTCGAATGAGCTTCC
AGATTATTCAAAAATTTCGGAAGACAGAATAAAATTAATATTTGCATAACGCACACTATGACAAAAGGGCAAAAATGCAAAATCGGGGGTTGATTGGTCTATATTTACGAATAAGGGCCG
AAGGCCCGTACACATTGCGCGGGAAATTCAAAATCCGGACGCCAAATATTATTAAATACAATACACCGCCTTTTAGAAATCCGTGGACGAGAAGATGCCACGTAGCAAAACACTGTACTC
CTTGGCTCACTATAAATACCTGAGCCAAGGTCGGGCATAG
```

BBTA2

Best match to EU430730 100% coverage 97% identity; MG545616 100% coverage 97% identity; NC_038953 100% coverage 96% identity

Rep protein best matches to ACB86656 100% coverage 99% identities (4 aa) 99% positives; YP_009508281 100% coverage 93% identities (11 gaps in middle) 94% positives Banana bunchy top alphasatellite 2

```
>BBTA2_alyu33
TATTACCCACCTCCGCGCACCACCTCCGCGCACCTATAAAATGTCTGCCTCTCGCTGGACATTTACGCTTCATTATTCCGACGCAACGGAGCGAGGCAAATTCCTCGCGACTTTGAAGGA
GGAAGATGTGCACTACGCCGTCGTCGGCGACGAAACTGCTCCGAATACAGGTCGAAAACATCTTCAAGGATATCTTTCCTTGAAGAAACGTTTTCGTATTAGCGGAATAAAGAAGAAATA
TTCGTCGAGAGCGCATTGGGAGAAAGCTCGAGGATCAGATTACGACAACAAGGCGTACTGTTCCAAAGAAGCCCTAATTCTTGAATTAGGGGTTCCTTGCCAAACAGGTTCGAATAAGCG
TAAATTAGCAGATATGGTTACAAGATCGCCGGAACGAATGAAAATTGAACAGCCAGAGATATTTCACCGATATCAATCGGTGAAGAAGATGAAAGAATTTAAAGAAAGGTATGTCTATCC
TAACCTCGATAGGCCATGGCAGGTACAATTAACGGAGTTAATTGAAGCAGAACCTGATGATCGAACGATCATCTGGGTATTCGGACCAAAAGGGAATGAAGGCAAATCAACGTATGCGAA
GTCATTAATCCAAAAGGATTGGTTCTACACAAGGGGAGGAAAGAAGGAGAACATATTGTTCGCCTACGTAGATGAAGGTTCGACCAAAAACGTTGTATTTGATCTTCCGCGTACAGTACA
AGAATTTATTAATTATGATGTTATCGAAGCACTGAAGGATAGAGTAATCGAGAGTACAAAATACAAGCCTGTGAAGTATTTAGAATTGAATTCTGTACATGTAATAGTTATGGCTAATTT
TCTTCCTGATATGTGTAAAATATCTGAAGATCGAATAAAAATAGTTGCTTGCTGAACACGCTATGAAATACAACACGCTATGACAAACGGGGAAAAATGAAGAATCGGGGGTTGATTGGG
CTATCCTAACGATTAAGGGCCGCAGGCCCGTCAAGATGGATCCAATAACCCGATAAGAAGCTAAACGGGTCTAAAACGATTCCTTCGCCCGCAAGCAACACCTTTAACCTCTGCGCACCT
ATATATAGCGGAGGTGGGCTAG
```

DNA-C

18-22 SNPs to KM607098 KM607099 (**China**). **24-30 SNPs** to KY427060 MF039864 MF039876 MF039870 (**Thailand**)

C protein (161 aa): 1 aa to KY427060 (**Thailand**) **2 aa** to KM607098 KM607099 (**China**) MF039864 (**Thailand**)

```
>C_alyu33
TATTACCCCCCTGCTCGGGACGGGACATCACGTGCATCAAACAAATGCACGTGACAAAGCAGTAGCTTGTAGCGAAAGATAACCATCACTATCACAAAAGTGAGAGGAATATTCGTTGC
TTAGGGACTAAGCAACGAGGACAGATATTTGTTCGAGAAGCGACAATGGAGGCTATTTAAACCTGATGGTTTTGTGATTTCCGAAATCACTCATCGGAGCAGAAATGGAGTTCTGGGAAT
CGTCTGCCATGCCAGACGATGTCAAGAGAGTGATCAAGGAAATATATTGGGAGCATCGAAATAAACTTCTGTTCTGTCAGAAGTTGAAAGGCTGTGTAAGAAGGATTCTGGAGCATGGAA
ATCAAGATGATGCTCTAGACGCAGTGAAGGAGATGAAGACTTCTATTATTCGCTATAGCGAATATTTGAAGAAACCATGTGTGGTATTGTGTTTTAGTACTAATAAATCTATTGCATATA
GGTTAAACACAATGGTGTTCTTTTATCATGAATATATTGAGGACCTAGGAGGTGACTACTCGGTATATCAAGATCTGTTCTGTGATGAGGAACTTCCTTATTCGTCGACAGAAGAAGAAG
ATGAAGAAGTAATATACAGGAATGTTATCATGGCATCGACAGAAGAGAAGATCTCTTGGAGTGAATGTCAAAAGATAGTTATATCAGATTATGATGTAACATTACTGTAATGAATATCCA
TTATCATAAATAAAATAATGGAATGATGATTATGTATTCTAGTATAAATACATAATGGTATACGTATAGCATAAAATACATAAACCAACATACAACACGCTATGACAAACGGGGAAAAAT
GAAGAATCGGGGGTTGATTGGGCTATCTTAACGATTAAGGGCCGAAGGCCCGTTTAAATATGTGGTGGACGAAGTCCACAACACAAAAAAGTGATCAGAACAATGGAATATACTGAGCTG
GCAACGTAGGGACCATGTCCCGAGTTAGTGCGCCACGTGAGCAGGGGGGCTTAT
```

DNA-M

19-22 SNPs to KY427061 MF039865 MF039871 MF039876 (**Thailand**). **55 SNPs** to KM607239 (**China**)

M protein (117 aa): 0 aa to KC581796 (**Thailand**) **1 aa** to MF039871 MF039865 MF039877 KY427061 (**Thailand**)

```
>M_alyu33
TATTACCCCCAGCGCTCAGGACGGGACATCACGTGCAACTAACAAATGCACGTGACTGATATATACTATATAACGGTTTAATGAACCGTTATATATTGTTGTAACGCAAAGTCACGTGTG
AAAGCGAAATGCACGTGAATAAGTCAAATGTATTGAATAAACATTTGACGTCCGGATGCTTCCTACGGAAGCCATGGTTGCTTCGTGGCGAAGCAAAACATTTATATATTGGCCTGGACT
GCTGCCTATAAATAGGCACGCAGGGAAATGGCATTAACAACAGAGCGAGTGAAACTATTCTTTGAATGGTTTCTGTTTATTGGTGCAATATTTATTGCGATAACAATATTATACATATTG
TTGGCGTTGCTCTTTGAGGTCCCCAAGTATATTAAGGATGTTGTGAGGTATCTCGTAGAATACCTGACTAGACGACGTGTATGGATGCAGAGAACGCAGTTGACGGAGGCAACAGGAGAT
GTAGAACTCGGCAGAGGTAGTGTGGAAGACAGACGGGATCAACAACCGGCTGTCATACCACATGCAACACAGGTTATCCCTTCGCATCAACCTAGAAGGGATGAGCAAGGAAGACGAGGA
AACGCCGGACCGATGTTTTAATACACGGTATTGTAATATACGAAATATAAATGGATAATGATAAGTATTGTGAAAAAAATATATGTGAAACATAATATATGTTTGATAATTTACATATTG
CAATATGTGAATTGTATACGAGTGTTGTATTTATAAAACATACAACACGCTATGACAAACAGGGAAAAATGAAGAATCGGGGGTTGATTGGTCTATCGTATCGCTTAAGGGCCGCAGGCC
CGTTGAAATGATTCTTTATAAAACAAATATACATGATACGGAATGTTGAAAATATAATCAACGATGTATAAATACAACAGAATGTTGTATACTAATTAAAATAATGAGAAGATAAGTATA
TTTGTGAAGGATAAGGATCACAACCAACACTTTAGTGGTGGGTCATATGTCCCGAGTTAGTGCGCCACGTAAGCGCTGGGGCTTAT
```

DNA-N

20-24 SNPs to MF039872 MF039878 MF039866 KY427062 (**Thailand**). **25 SNPs** to KM607387 KM607385 (**China**)

N protein (154 aa): 0 aa to MF039872 MF039878 KY427062 (**Thailand**) KM607387 KM607385 (**China**) **1 aa** to MF039866 (**Thailand**)

```
>N_alyu33
TATTACCCCCCGTGCTTGGGACGGGACATGACGTCAGCATAGATTATAATGGGCCAACTAAAGGCCCATTTAATAGAATAGGCGGGCTTTTGACATATTTCAAAGGCCCAGCCTGGAAGG
GGATAATGTCACGTGCCTAATAAGAGGTTGCTTCGCCTCGAAGCAATCCGAATAAATGTTGCGTATTCAATACGCAACTGAAGTCTATTAATATGGATGTCTCTGCCGAATAAATCAGAG
CGTAAGCGAAGCAGAAGCGATGGATTGGGCAGAATCACAATTCAAGACATGTACTCATGGCTGCGATTGGAAGACGATATCATCGGATTCaTCCGAGAATCGACAATATGTACCTTGCGT
CGACGCTGGCAGCGGAAGAAAGTCACCTCGCAAGGTACTGCTAAGATCTATTGAAGCTGTGTTTAATGGAAGCTTCAAAGGAAATAACAGGAATGTTCGTGGGTTTATATACGTATCAAT
ACGAGACGACGATGGAGAAATGCGTCCAGTACTCATAATACCCTTCGGAGGATATGGATATCATAATGATTTCTATTATTTCGAAGGGAAGGGGAAAGTTGAATGTGATATATCATCAGA
TTATGTAGCGCCAGGAGTCGATTGGAGCAGAGACATGGAAGTTAGTATTAGTAACAGCAACAACTGTAATGAATTATGTGATCTGAAGTGTTATGTTTGTTTGTTCGTTAAGAATAAAGGA
ATAACAGATGTGCTGTAATGATTATTAATAAAACATATTTTCATGTAATTGATAGTTGTATAAAACATACAACACGCTATGACAAACAGGGAAAAATGAAAAATCGGGGGTTGATTGGTC
TATCGTATCGCTTAAGGGCCGCAGGCCCGTTGAAAAATAATAATCGAATTATAAACGTTTGATAATAATCAGAGATAGATGATAAGCATCCAAAAACATAGACGAAGTATATGGCTGTAT
AATATAAAAGAAGCATATAAATAAAATATGTAAACTAATCTCTGATTGGTTTAGAATGTAACCCCACCAACTTTATGTTGGTGGAAATGTCCCGATGACGTAAGCACGGGGGACTAT
```

DNA-R
11-15 SNPs to MF039879 MF039873 MF039867 KY427063 (**Thailand**). **15 SNPs** to AB113659 (**Viet Nam: Hanoi**). **17-19 SNPs** to KM607678 KM60767 KM607676 KM607679 (**China**)

R protein (286 aa): 0 aa to KM607676 KM607677 KM607679 (**China**) **1 aa** to MF039879 (**Thailand**) AF416464 AB113660 (**Vietnam**)

>R_alyu33
TATTACCCCCAGCGCTCGGGACGGGACATTTGCATCTATAAATAGACCTCCCCCCCCCTCCACTACAAGATCATCATCGTCGACAGAAATGGCGCGATATGTGGTATGCTGGATGTTCAC
CATCAACAATCCCGCTTCACTACCAGTGATGCGGGATGAGTTTAAATATATGGTATATCAAGTGGAGAGGGGACAGGAGGGTACTCGTCATGTGCAGGGATACGTCGAGATGAAGAGACG
AAGTTCTCTGAAGCAGATGAGAGGCTTCTTCCCTGGCGCACACCTTGAGAAACGAAAGGGAAGCCAAGAAGAAGCACGGGCATACTGTATGAAGGAAGACACAAGAATCGAAGGTCCCTT
CGAGTTTGGTGCCTTTAAATTGTCATGTAATGATAATTTATTTGATGTCATACAGGATATGCGTGAAACGCACAAACGGCCTTTGGAGTATTTATATGAGTGTCCAAATACCTTCGATAG
AAGTAAGGATACATTATACAGAGTTCAAGCAGAGTTGAATAAAACGAAGGCGATGAATAGCTGGAAAACATCCTTCAGTTCATGGACATCGGAAGTTGAAAATATTATGGCGGAGCCATG
TCACCGAAGGATAATTTGGGTCTATGGCCCAAATGGAGGAGAAGGAAAGACAACTTATGCAAAATATTTAATGAAGACGAAGAATGCGTTTTATTCGCCAGGAGGAAAATCATTGGATAT
ATGTAGATTGTATAATTATGAGGAAATAGTTATATTTGATATTCCTAGATGCAAAGAGGAATATTTAAACTATGGTCTATTAGAAGAATTTAAAAATGGAATTATTCAAAGCGGGAAATA
TGAACCCGTTTTGAAAATTGTAGAATATGTAGAAGTCATTGTAATGGCTAACTTCCTTCCGAAGGAAGGAATCTTCTCTGAAGATCGAATAAAGCTAGTTGCTTGCTGAACACGCTATGA
CAATCGTACGCTATGACAAAAGGGGAAAAGCAAAGAATCGGGGGTTGATTGGGCTATCCTAACGATTAAGGGCCGTAGGCCCGTCAAGATGGACGGTTTGATCAGATGTCCCGAGTTAGT
GCGCCACGTAAGCGCTGGGGCTTAT

DNA-S
15/16 SNPs to AB113661 AB113662 (**Viet Nam**). **16 SNPs** to MF039874 (**Thailand**). **17-20 SNPs** to KM607535 KM607534 KM607536 KM607533 (**China**). **22 SNPs** to KY427064 (**Thailand**)

S protein (170 aa): 0 aa to AY337715 AY494786 HQ378191 (**China**) FJ787433 FJ787434 (**Philippines**) **1 aa** to several isolates

>S_alyu33
TATTACCCCCAGCGCTCGGGACGGGACATGGGCTAATGGATCATGGAATAAGGGCCCATCGGGCCCGTTAAGATGGGTTTTGGGCTTCTGGGCTTTATCCAGAAGACCAAAAACAGGCGG
GAACCGTCCCAATTTCAAATTTAGATAGCTTGCCCCGCAAGCAAACTAAAAGTCTATATATACCAGTGTAGACATATTGTTCAGATAACAAATGGCGAGGTTTCCGAAGAAATCGATCAA
GAAGAGGCGGGTTGGGCGACGGAAGTATGGAAGCAAGGCGGCAACCAGTCACGACTACTCGTCGTTAGGTTCAATATTGGTTCCTGAAAATACCGTCAAGGTATTTAGGATTGAGCCTAC
TGATAAAACATTACCCAGATATTTTATCTGGAAAATGTTTATGCTTTTGGTGTGCAAGGTGAAGCCCGGAAGAATACTTCATTGGGCTATGATTAAAAGTTCATGGGAAATCAACCAGCC
GACTACATGTCTGGAAGCACCAGGTTTATTTATAAAACCTGAACATAGCCATCTGGTGAAACTGGTATGCAGTGGGGAACTTGAAGCCGGAGTAGCAACAGGGACATCAGATGTTGAATG
TCTTCTAAGGAAGACAACCGTGTTGAGGAAGAATGTAACAGAGGTGGATTACTTGTATTTGGCATTTTATTGTAGTGCTGGAGTTAGTATTAACTACCAGAACAGAATTACATATCATGT
ATGATATGTTTATGTAAACATAAACCTTTGTATGGAATAATGCCCAAATAACATACAACACTCTATGAAATACAACACGCTATGACAAACAGGGAAAAATGAAGAATCGGGGGTTGATTG
GTCTATCGTATCGCTTAAGGGCCGCAGGCCCGTTGAAATGATTCTTTATAAAACAAATATACATGATACGGATTGTTGAAAATATAATCAACGATGTATAAATACAACAGAATGTTGTAT
ACTAATTAAAATAATGAGAAGAAAAGAATATTTGTGAAGGATAAGCATCAGAACCACCACTTTAGTGGTGGGTCATATGTCCCGAGTTAGTGCGCCACGTAAGCGCTGGGGCTTAT

DNA-U3
25-32 SNPs to KY427065 MF039869 MF039881 MF039875 (**Thailand**)

U3 protein (39 aa): 0 aa to KY427065 (**Thailand)**

>U3_alyu33
TATTACCCCCAGCCCCCAGGACGGGACATGGGCTTTTTAAATGGGCCGAGAGAGTTTGAACAGTTCAGTATCTTCGTTATTGGGCCATCTGGCCCAATAATTAAGAGAACGTGTTCAAAA
TCGGGGTTTGACCGAAGGTCAAGGTAGACGGTCAACAATATTCTGGCTTGCGGAGCAAGCAACACGAATTAAATATTTAATTCGTAGACACGTGGACGGACCGAAATACTCCTGTATCTC
TATAAATAGCTTAAATCTGGCTTGGATATTTGCTCTCGCTCTTCTGTCAAAGCTGTTGTGTTGAGGCGGAAGATCGCCATCGGCGATCATCAGACGAAAAGCTGCAAGAGAGACGGAGAA
CCATGCTGCGAAGCGTATATCCGGTATTTATAGACTTCTAGCGCAGCTAGAAGTTTCCCTGTACTTGATATTGTATTTTGTAAATTACGAAGAAATTCGTACATTGATAATAATAAAACA
TCTGGGTTTGTTAATGTTTACATTAACCAGTATCAATAATGTACAATAACAAATATTGTATGAGGAACGAATACAATAATATTAAAATATGAGGAGCGTAGCGTGATAAACAGGTGTTTA
AGGTATAATTAAATTAATTATGCAATGTCATGATAATACGGTATAAGTTGAAGTATGAGGTGAAAGAGGAGATATTAGAATATTAAAAAACCCAATTATATTATTTATAATACATACAAC
ACGCTATGACAAAAGGGGAAAAATGAAGAATCGGGGGTTGATTGGTCTATCCTAACGATTAAGGGCCGCAGGCCCGTCAAGATGGATGAACGGTTAGATTTGATTGCTTAGCCAAGAAGA
AAGGAATCTTTTTGGGACCACAGATAAGACAGCTGTTACTACCTTTAAAAATAATATAATATCCAATTGACGATAGTACCCCTCCCAAAGATGACGTATAGGGGTGTCCCGAGGTAATTT
AACATAGCTCTGACAAGAGATAGTGGACGTTGGATGCCACGATCGGACGATCGTGGTTTGATGAACTAATGATGACGTAGGGGCTGGGGCTTAT

BSVNV
Best match to KJ013510 **Banana streak VN virus** Banana streak virus Acuminata Yunnan 100% coverage 90.4% Identity Gaps: 46/7738; AY750155 Banana streak virus Acuminata Vietnam 100% coverage 88% Identity 1% Gaps: 74/7516

ORF I protein (**CTG** start codon) best match to YP_605809 Banana streak virus Acuminata Yunnan 96% identity 99% positives

ORF II protein best match to YP_233109 Banana streak virus Acuminata Vietnam 89% identity, 94% positives

ORF III polyprotein best match to YP_605811 Banana streak virus Acuminata Yunnan 96% Identity 98% Positives

>BSVNV_alyu33
TGGTATCAGAGCTAGATTTGTAAGTAATTTATGGCTTTTATGGGGTAAAACCCTTAGATAGGAGCCTAAGGGCTCTGCTATGTTCTGCTTATGAAAATAGTTGTTCAAGTAGAAAATGCA
TGATAAATCGAGTAGGTTCTTACAAATGGTCCTGAAATTATGAAATTATCCTTTTATACTTTGAGGGAAACTTCTGTGTCTCCAAGTTCTACTGAGATGATACCTATCCAAAGAAGGTAG
TAGCCTGGTGAAGGGGAAGAAAAATGAGTTGAGTTGGGAGTAGAATTCTCTAAGGGCTAGGGGCAAAAGAACCGGAACCTTACCCAGTAAGTAAGACCCGCGGAAGGGCAGTAAGCTGGA
GAACCCCGGTGTGAGCTTCTTTACCTTAGAAAAATTATACTCTAGTATCCTCAAAACTGCGTGAAGTAATTCCCAAAACCATGTTACTAATTTTTAGGATATTTATCAGATCACAATCAA
ACTTGAGTTGTACAGGGTAGAACTCTGGTATGACTGGATAACTAATGATTAAGGACCTTCACTAAGAACTTAGTAAGAAGTAAGTGCATAGACTACCTCTGGAACTGACTACAATCATGA
GCAAACATATGCATCCCGAAGGTTACTCCTATCTGTCCTAAGACCATAAGAAACTTCTCCTTAAAAACT**CTG**GAATCCTATTGGGATCTTGAATTTGAAAAATATAAAAATTCTCACTCC
AAATCTGTCTCTGATCTTTCTTATCTAGACTTAGCTACTGCTGAAAAGGTCTCTAATAAAGACCTAGCACATAATTTGCATATAAACACCTATCGTAGTGATCTTGGCTTTAAAGTTGCA
ATTCATTCTTTATCAAAAAATCGTGAGCTACTAATCCAGAACAGAAAGCTCTTGGAGGAGCAAAAGCAACAGCTTTCTGAAATCAGCAACCTATCTAAGGTTGTGCGTCTCCAACGCGCT
GACTTAAAGGAGACCCTAAAGCAGCAGGACGTCCTTGCGAAGGAGCTTCAAGCTCTTCGTAAAGACTATCTGGAGAGGCGTCCCCTCAGCAAAAAGGACGTAGAAGAACTGGTTGTCCGA
ATCTCAGAACAACCGAAGTTCATTGAAAAACAAACTGAGGCTCTCACTATAGAGCTCACAAAAGAAGTCAGTACATTAAAGTCCATCATCAAAGACTTTGAGCAAAGGCTCCTGGGATGA
GCCTAGCTGGAAGCAAGGCAACAGCTGCATATCAAGAAGCCTTGCAATCCACTACTGCTGGCTGGGAAGATCTCGGCATCGGATTCACTGACAAGGGAATTATCAGCACAACAACCTTGT
CAAGGCAGCTTAACTCGCTGTTATTTTTGGTGGTTAAGCAAAACCAGCAAATTACCACCATCACTGAAGAGCTGACCCAACTCCACAACCGGGTCAAAAACCTTGAAGGGAAGACAGGGG
TATCCGCAAGTCCCCTGAACAAATCTGAAATTGAATCCATCAACGACAAGCTTAGAAGGATTCAAGAAATCCAGGGGAGTCACCCACCAAAAGAGACCCCCTCCGGAGCTATCAAAGTTT
TTGAGGATCCCTATAACATCCTCAGAAAATTATAATGGCATCAAGGCCGAGGGTTTCCGGAAGCACCACCCGAACCATGGTGGCAGAACCAGGGGTTCCCCTGGTTGATGATCAGATCCG
GGAATACCGGAATGCGGCCCGAGCCGCATATGAAGCGCAAAGGATTGCTCGACGCACGGGCAATATTTTTGGAAGAATTGTTGGGCGACAACCAAGGGAACATACCCTTGCAATGGTCGT
AGACCCCAACAGTGAGCTGGAACGCTCACTAGCTCACCGTGCACGAACGATACCCGCTGAAGTCCTCTACATGACCCAGCGCGGTGAGCCTGCAAATCGGGTCTACCGGAACCGAACTGA
AGAAAGGATGCTCGTGACTCATGAGCAACAAGACAGGACCTTCATCCTGCCTGAGTCATATGAAGAACTCAGAGAAGCTGGATTCGAGTACATCCATCTAGGAGTACTCCAGGTGCGCAT
CCAAATTATGCACCGTTCATACGCTGGTACAATGGCGCTAATAGTTTTCAGAGACACCAGGTGGACGCAAGAGAACCACCAGGACAGATCCATTATAGCAACCATGGAGGCTGACCTTTC
ACAAGGGCATCAGCTTATTTATGTCATCCCAGATATAATGATGACAATCAGGGATTTCTACCAGCACATCCAGATCAGCATCCTAACAAAAGGATATGAGGGCTTTCAAGGAGAAGCAAA
TCTCCTTATTACAAGAAGCTGCCGGTGCAGATTAAGCAACGTTCCTAACGTTGGCTTCCAGTATAATATTCAAAACGTCGTGGAATTCCTAAAATCAAAAGGTGTAAAAGCCCTAAACGC
GACGAAATTAAGCACCAGGAGGTTCCAAGGGAGTGAATGGAACATCCGACCATCAGAAGTGGTGGTCCCTATGCAGCCAACAACAATGATAGTACGAGTTAATTATGACTCGTCAAGAAG
CATCAGATTTGGGGATTATGAAGCCAGCACATCATCATCAGCCCCAAAGTACGAACAAGACGGTGATGAAAATGAAGCACTTGGTGAAATCCACCAAGTAAATATGATCACCATTATTGA
AGATGATGCAGAAGATGACTACCCACGGTTATCAGCTTTAGAGCGAATAATCGCTCCAGAAAGCATGGTGGGAGAGGAGGACACAATTGCCGAATTTTTAGGCAATCTGTCCTTAGATTC
CTCCACTGATGAGGAATTCTATGACGCTGACAATTATCTATTCGAGGAAGAAGAATATGATGGAGATAATGATTCTGAAGTCAGCACACCAAGGAGCAAATACAACATATTTGCACTAGA
GGATGAGTACCCAAAGCTTCAACAGCTGGAAAGTCTAGTACTCTCCACAACAGAGTCTGCCATCAGCCGCTTTAGACCTGCAGACACAGACATGACTGGCATAGGCCCCGCCTATGCACC
AGCAACCGGAACAGCTGGCTACACTGGAGCCAGTTCATCTGATTTCCCTTACCCAAGAAGGCCAAGAAAGTGGGACAACAACTCAGAATGGTTTAACCTGCCCACTGCTAATGCCAGGCA
AGCATCAATATTCGTCATGCCTCAGGACTTCGACACAAAGGTCTTTGAAAGATGGGAAAGCTCTGTTCTCCTCCATATGTCTGATAAGGTTTTCGATGATCCACAAGATAAGCTTACCTA
TGTGGAAAATCTGCTAGGAGAATCAGAGAAAAAGATGTTTATCACTTGGAGGATGATGTTCACAGCCGAATATGAAGAAATGAAGAACAACGCCCTCGGCTCAAATGGAACACAGAACAT
CCTGAACCAGATCAGGATGATATTCTTTCTGGAGAATCCCCAAGTCGGAACAACTAATACTCAGGACGCTGCATACAAAACGCTCAAACAACTGGTGTGCACAGAAATGTCCGGACCTGC
AATCTACAGGTACCTCAACGATTACTTCCATTTAGCTGCTAAATCTGGAAGAGCATGGGCATCTGATGAGCTGTCCAAAGAGTTCTTTACTAAATTACCAAAAGGATTGGGGGATAGAGT
GGAAAAGGAATTCAAGAAAAGATACCCCAACAACACTATTGGAGTGGCACCCAGAATTACCTTCACAAGGAATTATATGAAGGAAATATGCCAGGAAGCCGTATTCCAAAGCCAACTGAA
GAAGCTGGACTTCTGCAAAGGAACACCTGTCCATGGCTTATACGGAAAAGATAAGGTATATGGGAGGAAATACGGAGTCAGAAAGAGTACTTCGTACAAAGGAAAGCCTCACAAGTCACA

TGTGAGGATAGACAAAAAGAAGCACCTCCTGATGAAAAGAAAAGACTGCAAGTGCTTTGCTTGCGGTGATGTCGGCCACTTCGCATCAGAGTGTCCGAATCCCAAGAAACTGATGCACAG
AGTTCAGATCCTACAATCCCTGGAGCTTGATGACGGAATCGATGTGATCTCCGTAGGATTTGATGAGTCAGATGTCTCAGACATCTACTCAGTCTCTGAAGGCGAGGACAACTACCAGTT
CAATAATGAAGACTTTGACGTTATAGGACATGACGTGTTCATGTTCACTATTGAAGAACAGAGGAACTGTCTGGTAGAAACCACATCAGCCTGGAGGAGTGCAATGAAAGTCACTTCAGA
AGAAAAGAACTGCTCACACACATGGAGCTTTGAGGAAAAGACATCAGACTACTGCAGGGCGTGCAAAAATCGTGCCCTACAAGGAAGCAGAGCTGACTGCACTCAGTGTAAGATGATTAT
CTGCTCTTTATGCAAACCATACTATTTCCAGGACGGTTCACCGATCCCAGCACAAGGAACAACCCCCTCAGGACTCTCCCACGATGACTGGATGGGATCAGCAAATAGGTGGAAAGCACA
CTATGAGTTCTCTCAGGCAAGAAGAAAAAGCCTGAAGGCAGACCTGGAAAAGGCAGAAGAAGAGCTGAAGTTCTACAAACAAAAGGAAAAGGAAAGAGCCAAATTGAAGAATCAAATTCC
AGAGGCAATACAGGCAAAGCTCGATGATCTGGAAAAAGAAAAGGAGCTCAACAATATTCTAAGGATAGAAGCAGAAGCAGAGCTAAAAGCACTTAAGAAAGATTTTAAGGAAAGGGAAGA
AGCCCTGATGGGAGAAATCACAGCCTTAGAAGAGGAAGTAAGGCTGCACAAGGAAGAATCTGAAGAACTTCAAGAGGAAAATCAAAGGCTCAAAGAAAAGATCCTAGCCTTGGAAGAAAA
AGAAACAATACAAGAACCAGAGGAAGTGATCGAATTAGTCAATAATGTGGAGGAACATCTGGTGCTAACAGGACAACAGAAGAACAATCTCCTCAACATTAAGATTACTCTGGAAATCAA
GGAAAAAAAGATCACCATGAACGCAATACTAGACACTGGAGCAGCAATCTGTGTCTGCGATGGAAAGATGGTTGACGAATACTTCAGAAGACCATCCATGATGAACGCGTTCATAAAAGG
AGTCAACGGGATTACCAATGTCAAAGAAATTCTGGAAGAAGGAAAAATATGGATTGGTAATCAATGGTTCAGAATCCCAAGGACATACATCATGCCCCAGCTATCGGAAGGCCTTCACTT
CATCATCGGAATGAATTTCATCAGGGCGATGGAAGGAGGGATTCGAATTGAACAAGGGATGGTAACCTTTTACAAGATGGTTACACAAGCACAAGCACCCCCTATGGTACATGACATTTC
TTACCTTGAGGAATTAGAACTTGAATTGCCTATCTATTATGATATTTGTGCAACTAACCCCTCAGGAGGAGAAATCAACAGTGACCTAATACCCCCTTCAGAAATCCAGAAATTGAAGGA
CTTAGGTTACATTGGGGAAGAGCCCTTGAAACATTGGGCCAAGAATCAGGTCAAATGCAAAATTGAAATTAAAAATCCTGATCTGGTAATTGAAGACAGGCCACTGAAGCATGTGACCCC
AGCAATGAAAGAGTCCATGAAAAGGCATGTGGATAAGCTGCTGGAACTCAAAGTAATTAGGCCTTCAACAAGTAAACACCGGACAACTGCTATTATTGTCCAATCCGGTACAGAAATTGA
CCCCCTCACCGGAAAAGAGAAAAGAGGAAAGGAGAGGCTGGTATTCAACTACAAACGCCTCAATGACAACACTGAAAAAGATCAGTATTCACTACCCGGTATCAATACAATCATCAGCAG
AATCGGCAAGTCAAAAATCTACAGCAAATTCGACTTAAAATCGGGTTTTCATCAAGTGGCCATGGATCCAGAAAGTATCCCATGGACAGCCTTTTGGGCCATAGATGGACTATACGAATG
GCTTGTTATGCCATTTGGATTGAAGAATGCGCCTGCCATATTTCAGAGAAAGATGGACAACTGCTTCCGAGGAACGGAAGATTTCATAGCAGTATACATCGACGACATTTTAATATTCTC
TGACAATATCTCTGATCACAGGAAACATCTGTCAAAATTCCTGGAAATTTGCAAGGCGAATGGGCTGGTCCTAAGCCCAACGAAAATGAAGATTGGCGCAAGGGAAATTGATTTTCTTGG
AGCAACTATCGGAAATTCCAAAATCAAGCTCCAACCACACATAATCAAGAAGATTATTGAGACAAAGGATGAAGAGCTCAAGGAGACAAAAGGACTAAGAAAATGGCTGGGAGTCCTTAA
CTATGCACGGGCATATATTCCAAACTTAGGGAAAACTCTAGGCCCGCTCTACTCCAAGACATCTGTTAATGGAGAAAAGCGAATGAACAGCCAAGACTGGAAGGTTGTTCAGCTGATTAA
GGAGCAGGTACAAAATCTACCTGACCTTGACATACCCCCTACAGAAGCAATTATTGTCTTGGAAACCGATGGGTGTATGGAAGGATGGGGAGGAGTGTGCAAATGGAAACTCCGTCCCTC
TGACACAAGGCTGGCAGAAAAGGTCTGTGCATACGCTAGTGGAAGATACCACCCCATCAAGAGCACCATTGATGCGGAGGTTCATGCAGTGATCAACAGCCTGGAAAAATTCAAAATTTA
TTACCTGGATAAAAGGGAGTTGATCATAAGAACAGACAGCCAGGCTATTGTGGCCTTCTACAAAAAGCAAGCAGATCATAAGCCTTCAAGAACAAGGTGGCTTATGTTGATTGACTACAT
CACTGGGCTTGGAATCAACGTCAAATTCGAGCACATTGACGGGAAAGAAAATGTCCTAGCTGACACTTTATCAAGATTGGTCCAAGTCCTAATTACAAGGGTTCATCACCCAGCAGAGAC
TCAGCTAGTTGACGCCGTCATAGAAGTCATGGGCAATCCAAAAAAGGAAGCCTTGGACAAGGTAAATCATTTTATCTTTCTAACCCAACGGTGGATTGCAGAACATGAAGAAGAGCATAC
GGTGAACACACTGCTCCGATTGGAGGAACCGCAACTACACTGCGGATGTAGAAATCACGCAACGGGAGAAAGAAGAAATGCCATTCTCCTTCAAAGCCACACTTCAGCCAACCCGTACAG
ATGGTTCTATAAGTGTGCACAAGACAAGTGCCACATTTGGATCTGGAAGGATATCCTAGATCAATATGCTGAAGAATACGCTGCCTACACCAGGATAGGACTCGAGGCACTCAATCTTGA
AGACTGGTTTGAAGAACCAGAACCCGATCCACCCAACCTAGTGGACCGCCAGAAGATCGAAGACATCCTGGACCTGCAAGACGTCAGCAATGACGATTAAAAGATTCCCAGGATAGCCGG
CGGACGTGGTGGACCCAGTCTAGGTGCGATGCTTAGTCACGCACGATGACTTTGTCGGAAGGCATCTTTACTTTCGGCAAACTTTAATAATACTTTAGGAAAAGTATTTACAAGTCAGGT
GCAGAATCAATAATGCACCCAACTTTAGTCTTGTCTACTGAATTATTGTGTCGGTTGCATTATTGGATGCCTGCGTGCACCCTAAGCAATCTCCGGCCCTCATCTCTATAAGAGGAGCCT
TTGTATTCAGTTGCAAGCATGCAAGTTTCCAACTGCAAGCTTACTTCTGAGCAATCTTTGAGAAATATATTCCAAGTTTTCTTTACTTACATGTTCTTGAAAGTTTCTATCTGATAGAGC
GAGTAGAGAGCAAGATCAAATTTGGGAAATTCCGCTTTCATTTCTTCGA

ALYU-34 - Musa sp. - Vietnam

Virome
1. **BBTV** six components (each with two genetic variants)
2. **BBTA5** alyu34- new alphasatellite named here Banana bunchy top alphasatellite 5, genus *Banaphisatellite*
3. **BBTA2** alyu34 – Banana bunchy top alphasatellite 2, genus *Muscarsatellite*

BBTA5
Best matches to MF510475 MF510474 (3% coverage 66.5% identity Faba bean necrotic yellows virus associated alphasatellite 2; AJ132187 AJ005966 73% coverage 66.3-65.8% identity Faba bean necrotic yellows C9 alphasatellite; MK291270 72% coverage 67.0% identity Sophora yellow stunt alphasatellite 3

Rep protein best matches to QBF29185 98% coverage 63% identities,80% positives Sophora yellow stunt alphasatellite 3; ATU31571 98% coverage 62% identities 80% positives Faba bean necrotic yellows virus associated alphasatellite 2; ARI50297 98% coverage 62% identities 80% positives Sophora alopecuroides yellow stunt alphasatellite 3

>BBTA5_alyu34
TATTACCCGACCTTGGCTCACTGGCTCACTATAAAAGGCGGAGATGTCGCAGCAGCGAAATTGGGTATTCACGCGTAACTTCTCCGGCGACCCGCCGATTTTAAAGTTCGCCGGAGAAAC
TCAATACGCGTGTTGGCAACATGAGAAGAAGAATCACGACCATCTTCAGGGAGTGATTCAATTGAAGAAGAAGCTCCGGATGAACGCAGTGAAGACGTTAATTGGGGGAAATCCACACCT
CGAAGCCATGAGAGGAACAATCGACGAAGCAATAAGGTACGTGACGAAAGAAGAGACACGAGTAGCAGGTCCGTGGGAATTCGGAGAATTACTACGAAAAGGATCTCATAAGAGGAAATT
GATGGAATTGCTCGACGATCCAGATAACGAAATAATGGAACCCCAAAAATATCGACGCGCCATTACTAAACAAGCAATGGACGCGTCAAAGAAGAAGGCGGAATTAGGGTTTCCGTACGA
CTTAAAGGAGTGGCAAAAGATGGTTATTGAGTTACTCGAAGAACAACCCGACAACAGAACTATTATCTGGGTGTATGGACCTAATGGTGGAGAAGGTAAAACGCAGTTCGCAAAGCATTA
TGGATTACTCAAAGGATGGACATATCTACCAGGTGGTGAACTGAAAGACATGATGTATCTGTTGGCAAAAGAAATTAATAATAACGTTATTATTGATTTCCCCCGATGTACAAAGGATTT
CATATCCTATAAGTTTATTGAGATGGTAAAGAACCGATGCATATTTAGTTATAAATATGAACCAATCGGGGCGATTGTAAGTAATGAAGTCCATGTAATTGTAATGTCGAATGAGCTTCC
AGATTATTCAAAAATTTCGGAAGACAGAATAAAATTAATATTTGCATAACGCACACTATGACAAAAGGGGAAAAATGCAAAATCGGGGGTTGATTTGTCTATATTTACGAATAAGGGCCG
CAGGCCCGTACACAATGCGCGGGAAATTCAAAATCGGACGCCAAATATTATTAAATACAATACACCGCCTTTTAGAAATCCGTGGACGAGAAGATGCCACGTAGCAAAACACTGTACTCC
TTGGCTCACTATAAATACCTGAGCCAAGGTCGGGCATAG

BBTA2
Best match to EU430730 100% coverage 98% identity; MG545616 100% coverage 97% identity; NC_038953 100% coverage, 96% identity

Rep protein best matches to AZL93963 100% coverage 100% identities (4 aa) 100% positive; ACB86656 100% coverage 99% identities (3 aa) 99% positives; YP_009508281 100% coverage 94% identities (11 gaps in middle) 94% positives

>BBTA2_alyu34
TATTACCCACCTCCGCGCACCACCTCCGCGCACCTATAAAATGTCTGCCTCTCGATGGACATTTACGCTTCATTATTCCGACGCAACGGAGCGAGGCAAATTCCTCGCGACTTTGAAGGA
GGAAGATGTGCACTACGCCGTCGTCGGCGACGAAACTGCTCCGAATACTGGTCGAAAACATCTTCAAGGATATCTTTCCTTGAAGAAACGTTTTCGTATTAGCGGAATAAAGAAGAAATA
TTCGTCGAGAGCGCATTGGGAGAAAGCTCGAGGATCAGACTACGACAACAAGGCGTACTGTTCCAAAGAAGCCCTAATTCTTGAATTAGGGGTTCCCTGTCAAACAGGTTCGAATAAGCG
TAAATTAGCAGATATGGTTACAAGATCTCCCGAACGAATGAAAATTGAACAGCCAGAGATATTTCACCGATACGAATCGGTGAAGAAGATGAAAGAATTCAAAGAAAGGTATGTCTATCC
TATCCTCGATAGGCCATGGCAGGTACAATTAACGGAGTTAATTGAAGCAGAACCTGATGATCGAACGATCATCTGGGTATTCGGACCAAAAGGGAATGAAGGCAAATCAACGTATGCGAA
GTCATTAATCCAAAAGGATTGGTTCTACACAAGGGGAGGAAAGAAGGAGAACATATTGTTCGCCTACGTAGATGAAGGTTCGACAAAAAACGTTGTATTTGATCTTCCGCGTACAGTACA
AGAATTTATTAATTATGATGTAATCGAAGCACTGAAGGATAGAGTAATCGAGAGTACAAAATACAAGCCTGTGAAGTATTTAGAATTGAATTCTGTACATGTAATAGTTATGGCTAATTT
TCTTCCTGATATGTGTAAAATATCTGAAGATCGAATAAAAATAGTTGCTTGCTGAACACGCTATGAAATACAACACGCTATGACAAACGGGGAAAAATGAAGAATCGGGGGTTGATTGGG
CTATCCTAACGATTAAGGGCCGCAGGCCCGTCAAGATGGATCCAATAACCCGATAAGAAGTTAAACGGGTCTAAAACGATTTCTTCGCCCGCAAGCAACCACTTTAACCTCTGCGCACCT
ATATATAGCGGAGGTGGGCTAG

DNA-C
22 SNPs to KY427060 (**Thailand**). **22-25 SNPs** to KM607098 KM607099 (**China**). **24-28 SNPs** to MF039864 MF039876 MF039870 (**Thailand**)

C protein (161 aa): 2 aa to KY427060 (**Thailand**) **3 aa** to KM607098 KM607099 (**China**) MF039864 (**Thailand**)

>C_alyu34_v1
TATTACCCCCCCTGCTCGGGACGGGACATCACGTGCATCAACCAAATGCACGTGACAAAGCAGTAGCTTGTAGCGAAAGATAACCATCACTATCACAAAAGTGAGAGGAATATTCGTTGC
TTAGGGACTAAGCAACGAGGACAGATATTTGTTCGAGAAGCGACAATGGAGGCTATTTAAACCTGATGGTTTTGTGATTTCCGAAATCACTCATCGGAGCAGAAATGGAGTTCTGGGAAT
CGTCTGCCATGCCAGACGATGTCAAGAGAGTGATCAAGGAAATATATTGGGAGCATCGAAATAAACTTCTGTTCTGTCAGAAGTTGAAAGGCTGTGTAAGAAGGATTCTGGAGCATGGAA
ATCAAGATGATGCTCTAGACGCAGTGAAGGAGATGAAGACTTCTATTATTCGCTATAGCGAATATTTGAAGAAACCTTGTGTGGTATTGTGTTTTAGTACGAATAAATCTATTGCATATA
GGTTAAACACAATGGTGTTCTTTTATCATGAATATGTTGAAGACCTAGGTGGTGACTACTCGGTATATCAAGATCTGTTCTGTGATGAGGAACTTCCTTATTCGTCGACAGAAGAAGAAG
ATGAAGAAGTAATATACAGGAATGTTATCATGGCATCGACAGAAGAGAAGATCTCTTGGAGTGAATGTCAAAAGATAGTTATATCAGATTATGATGTAACATTACTGTAATGAATATCCA

TTATCATAAATAAAATAATGGAATGATGATTATGTATTCTAGTATAAATACATAATGGTATACGTATAGCATAAAATACATAAACCAACATACAACACGCTATGACAAACAGGGAAAAAT
GAAGAATCGGGGGTTGATTGGGCTATCTTAACGATTAAGGGCCGAAGGCCCGTTTAAATATGTGGTGGACGAAGTCCACAACACATAAAAAGTAATCAGAACAATGGAATATAATGAGCT
GGCAACGTAGGGACCATGTCCCGAGTTAGTGCGCCACGTGAGCAGGGGGGCTTAT

SNPs in C (all minor – ca. 15% reads): C42A T81C GA105AC G140T C164A T190G G461T G516A G577A G584A A807T G819A AA934GT

DNA-M
16-21 SNPs to KM607239, KM607240 (**China**). **49-50 SNPs** to KY427061 MF039871 MF039865 (**Thailand: Chonburi**)

M protein (117 aa): v1 **1 aa** to KC581796 (**Thailand**) **2 aa** to MF039871 (**Thailand**) KM607240 (**China**)
v2 **0 aa** to KC581796 (**Thailand**) **1 aa** to MF039865 (**Thailand**) **4 aa** to KM607240 (**China**)

>M_alyu34_v1
TATTACCCCCAGCGCTCAGGACGGGACATCACGTGCAACTAACAAATGCACGTGACTGATATATACTACATAACGGTTTAACGAACCGTTATATAATGTTATAACGAcAAGTCACGTGTG
ATAGAGACATGCACGTGACATAGTCAAATGTATTGAATAAACATTTGACGTCCGGATGCTTCCGACGGAAGCTTAGGTTACTTCGTGGCGAAGCAAAACATTTATAtAtTGGCCTGGAcT
GCTGCCTATAAATAGGCAAGCAAGGAAATGGCATTAACAACAGAGCGAGTGAAAcTATTCTTTGAATGGTTTCTGTTCATCGGTGCAATATTTATTGCGATAACAATATTATATATATTG
TTGGCGTTGCTCTTTGAGGTCCCCAAGTATATTAAGGATGTTGTGAGGTAtCTCGTAGAATACCTGACCAGAAGACGTGTATGGATGCAGAGAACGCAGTTGACGGAGGCAACAGGAGAT
GTAGAGCTCGGCAGAGGTAGTGTGGAAGACAGACGGGATCAACAACCGGCTGTCATACCACATGCAACACAGGTTATCCCTTCGCATCAACCTAGAAGGGATGATCAAGGAAGACGAGGA
AACGCAGGACCAATGTTTTAATACACGGTATTGTAATATACGAAATATAAATGGATAATGATATGTATTGTGTAACATAATATACGTGAAACATAATATATGTTTGATAATTTACATATT
ATAATATGTAAATTGTATACGAGTGTTGTATTTATAGAACATACAACACGCTATGACAAACAGGGAAAAATGAAGAATCGGGGGTTGATTGGTCTATCGTATCGCTTAAGGGCCGCAGGC
CCGTTGAAATGATTCTTAATAAAACAAATATACATGATACGGATAGTTGAATATATAAACAACGAAGTATAAATACAACAGAATGGTGTATACTAATTAAAATAATGAGAAGATAAGTAT
ATTTGTGAGGGATGATGATCACAACCACCACTTTAGTGGTGGGTCATATGTCCCGAGTTAGTGCGCCACGTAAGCGCTGGGGCTTAT

T/C (16364/81303) **A/T** (16346/81967) **A/T** (16783/80754) **C/T** (100085/21127) **C/T** (81691/20307) **C/T** (80026/17034) **T/A** (97135/20874) **A/C** (90266/19352)
A/G (88699/19010) **T/A** (32213/7550) **C/T** (23115/7191) **A/G** (23455/5601) **T/A** (27080/7060) **G/A** (34811/6758) **A/T** (33006/6267) **A/T** (45174/9845)

>M_alyu34_v2
TATTACCCCCAGCGCTCAGGACGGGACATCACGTGCAACTAACAAATGCACGTGACTGATATATACTACATAACGGTTTAACGAACCGTTATATAATGTTATAACGAtAAGTCACGTGTG
ATAGAGACATGCACGTGACATAGTCAAATGTATTGAATAAACATTTGACGTCCGGATGCTTCCGACGGAAGCTTAGGTTACTTCGTGGCGAAGCAAAACATTTATAaAaTGGCCTGGAtT
GCTGCCTATAAATAGGCAAGCAAGGAAATGGCATTAACAACAGAGCGAGTGAAAtTATTCTTTGAATGGTTTCTGTTCATCGGTGCAATATTTATTGCGATAACAATATTATATATATTG
TTGGCGTTGCTtTTTGAGGTCCCCAAGTATATTAAGGATGTTGTGAGGTAtCTCGTAGAATACCTGACCAGAAGACGTGTATGGATGCAGAGAACGCAGTTGACGGAGGCAACAGGAGAT
GTAGAGCTCGGCAGAGGTAGTGTGGAAGACAGACGGGATCAACAACCGGCTGTCATACCACATGCAACACAGGTTATCCCTTCGCATCAACCTAGAAGGGATGAaCAAGGAAGACGAGGA
AACGCcGGACCgATGTTTTAATACACGGTATTGTAATATACGAAATATAAATGGATAATGATATGTATTGTGaAACATAATATAtGTGAAACATAATATATGTTTGATAATTTACATATT
gTAATATGTAAAaTGTATACGAGTGTTGTATTTATAGAACATACAACACGCTATGACAAACAGGGAAAAATGAAGAATCGGGGGTTGATTGGTCTATCGTATCGCTTAAGGGCCGCAGGC
CCGTTGAAATGATTCTTAATAAAACAAATATACATGATACGGATAaTTGAATATATAAACtACGAAGTATAAATACAACAGAATGGTGTATACTAATTtAAATAATGAGAAGATAAGTAT
ATTTGTGAGGGATGATGATCACAACCACCACTTTAGTGGTGGGTCATATGTCCCGAGTTAGTGCGCCACGTAAGCGCTGGGGCTTAT

DNA-N
23 SNPs to KM607387 KM607385 (**China**). **29-31 SNPs** to MF039872 MF039878 MF039866 (**Thailand**). **32 SNPs** to KM607386 (**China**)

N protein (154 aa): 0 aa to MF039872 MF039878 KY427062 (**Thailand**) KM607387 KM607385 (**China**) **1 aa** to MF039866 (**Thailand**)

>N_alyu34_v1
TATTACCCCCCGTGCTCGGGACGGGACATGACGTCAGCATAGATTATAATGGGCCAACTAAAGGCCCATTTAATAGAATAGGCGGGCTTTTGACATATTTCAAAGGCCCAGCCTGGAAGT
GGATAATGTCACGTGCCGAATAAGAGGTTGCTTCGCCTCGAAGCAATCCGAATAAATGTTGCGTATTCAATACGCAACTGAAGTCTATTAATATGGATGTCTCTGCCGAATAAATCAGAG
CGTAaGCGAAGCAGAAGCGATGGATTGGGCAGAATCACAATTCAAGACATGTACTCATGGTTGCGATTGGAAGACGATATCATCgGATTCATCCGAGAATCGACAATATGTACCTTGCGT
CGACGCtGGCAGcGGAAGAAAGTCTCCTCGCAAGGTACTGCTTAGATCTATTGAAGCTGTGTTTAATGGAAGCTTCAAAGGAAACAACAGGAATGTTCGTGGGTTTATATACGTATCAAT
ACGAGACGATGATGGAGAAATGCGTCCAGTACTCATAATACCATTCGGAGGATATGGATATCATAATGATTTCTATTATTTCGAAGGGAAGGGGAAAGTTGAATGTGATATATCATCAGA
TTATGTAGCGCCAGGAGTCGATTGGAGCAGAGACATGGAAGTTAGTATTAGCAACAGCAACAACTGTAATGAATTATGTGATCTGAAGTGTTATGTTGTTTGTTCGTTAAGAATAAAGGA
ATAACAGATGTGCTGTAATGATCATTAATAAAACTTATTTTCATGAAATTGATAGTTGTATAAAACATACAACACGCTATGACAAACAGGGAAAAATGAAAAATCAGGGGTTGATTGGTC
TATCGTATCGCTTAAGGGCCGCAGGCCCGTTGAAAAATAATAATCGAATTATAAACGTATGATAATAATCAGAGATAGATGATAAGCATCCAAAAACATAAACGAAGTATATGGCTGTAT
AATATAAAAGAAGCATATAAATAAAAAACTGTGAACTAATCTCTGATTGGTTCAGAACGTAACCCCACCAACTTTATGTTGGTGGAAATGTCCCGATGACGTAAGCACGGGGGACTAT

G/A (54977/218448) **C/G** (59319/220772) **C/T** (61780/233524) **T/C** (62781/236903), **+ several other SNPs**

DNA-R
5/6/7 SNPs to KM607677 KM607676 KM607679 KM607678 (**China**). **8 SNPs** to AB113660 (**Viet Nam**). **14-19 SNPs** to MF039879 MF039873 MF039867 (**Thailand**)

R protein (286 aa): v1=v2 **0 aa** to KM607676 KM607677 KM607679 (**China**) **1 aa** to MF039879 (**Thailand**) AF416464 AB113660 (**Vietnam**)

>R_alyu34_v1
TATTACCCCCAGCGCTCGGGACGGGACATTTGCATCTATAAATAGACCTCCCCCCCCTCCACTACAAGATCATCATCGTCGACAGAAATGGCGCGATATGTGGTATGCTGGATGTTCACC
ATCAACAATCCCGCTTCACTACCAGTGATGCGGGATGAGTTCAAATATATGGTATATCAAGTGGAGAGGGGACAGGAGGGTACTCGTCATGTGCAGGGATAcGTCGAGATGAAGAGACGA
AGTTCTCTGAAGCAGATGAGAGGCTTCTTCCCTGGCGCACACCTTGAGAAACGAAAGGGAAGCCAAGAAGAAGCACGGGCATACTGTATGAAGGAAGACACAAGAATCGAAGGTCCCTTC
GAGTTTGGTGCCTTTAAATTGTCATGTAATGATAATTTATTTGATGTCATACAGGATATGCGTGAAACGCACAAACGGCCTCTGGAGTATTTATATGAGTGTCCAAATACCTTCGATAGA
AGTAAGGATACATTATACAGAGTTCAAGCAGAGTTGAATAAAACGAAGGCGATGAATAGCTGGAAAACATCCTTCAGTTCATGGACATCGGAAGTTGAAAATATTATGGCGGAGCCATGT
CATCGAAGGATAATTTGGGTCTATGGCCCAAATGGAGGAGAAGGAAAGACAACTTATGCAAAATATTTAATGAAGACGAAGAATGCGTTTTATTCACCAGGAGGAAAATCATTGGATATA
TGTAGATTGTATAATTACGAGGAAATAGTTATATTTGATATTCCCAGATGCAAAGAGGAATATTTAAACTATGGTTTATTAGAAGAATTTAAAAATGGAATTATTCAAAGCGGGAAATAT
GAACCCGTTTTGAAAATTGTAGAATATGTGGAAGTCATTGTAATGGCTAACTTCCTTCCGAAGGAAGGAATCTTTTCAGAAGATCGAATAAAGCTAGTTGCTTGCTGAACACGCTATGAC
AATCGTACGCTATGACAAAAGGGGAAAAGCAAAGATTCGGGGGTTGATTGGgCTATCCTAACGATTAAGGGCCGCAGGCCCGTCAAGATGGACGGCTaGATCAGATGTCCCGAGTTAGTG
CGCCACGTAAGCGCTGGGGCTTAT

SNPs close to 50% reads supporting R_v2

>R_alyu34_v2
TATTACCCCCAGCGCTCGGGACGGGACATTTGCATCTATAAATAGACCTCCCCCCTCTCCACTACAAGATCATCATCGTCGACAGAAATGGCGCGATATGTGGTATGCTGGATGTTCACC
ATCAACAATCCCGCTTCACTACCAGTGATGCGGGATGAGTTCAAATATATGGTATATCAAGTGGAGAGGGGACAGGAGGGTACTCGTCATGTGCAGGGATAtGTCGAGATGAAGAGACGA
AGTTCTCTGAAGCAGATGAGAGGCTTCTTCCCTGGCGCACACCTTGAGAAACGAAAGGGAAGCCAAGAAGAAGCACGGGCATACTGTATGAAGGAAGACACAAGAATCGAAGGTCCCTTC
GAGTTTGGTGCCTTTAAATTGTCATGTAATGATAATTTATTTGATGTcATACAGGATATGCGTGAAACGCACAAACGGCCTCTGGAGTATTTATATGAGTGTCCAAATACCTTCGATAGA
AGTAAGGATACATTATACAGAGTTCAAGCAGAGTTGAATAAAACGAAGGCGATGAATAGCTGGAAAACATCCTTCAGTTCtTGGACATCGGAAGTTGAAAATATcATGGCGGAGCCATGT
CATCGAAGGATAATTTGGGTCTATGGCCCAAATGGAGGAGAAGGAAAGACAACTTATGCAAAATATTTAATGAAGACGAAGAATGCGTTTTATTCtCCAGGAGGAAAATCAcTGGATATA
TGTAGATTGTATAATTACGAGGAAATAGTTATATTTGATATTCCCAGATGCAAAGAGGAATATTTAAACTATGGTTTATTAGAAGAATTTAAAAATGGAATTATTCAAAGCGGGAAATAT
GAACCCGTTTTGAAAATTGTAGAATATGTGGAAGTCATTGTAATGGCTAACTTCCTTCCGAAGGAAGGAATCTTTTCAGAAGATCGAATAAAGCTAGTTGCTTGCTGAACACGCTATGAC
AATCGTACGCTATGACAAAAGGGGAAAAGCAAAGATTCGGGGGTTGATTGGtCTATCCTAACGATTAAGGGCCGCAGGCCCGTCAAGATGGACGGCTtGATCAGATGTCCCGAGTTAGTG
CGCCACGTAAGCGCTGGGGCTTAT

DNA-S
6/16 SNPs to AB113662, AB113661 (**Viet Nam**). **16-19 SNPs** to KM607535 KM607534 KM607536 KM607533 (**China**). **20-26 SNPs** to MF039874 KY427064 (**Thailand**)

S protein (170 aa): 0 aa to U97526 AF246122 FJ463044 KX779467 (**China**) **1 aa** to several isolates

>S_alyu34_v1

TATTACCCCCAGCGCTCGGGACGGGACATGGGCTAATGGATCATGGAATAAGGGCCCATCGGGCCCGTTAAGATGGGTTTTGGGCTTATGGGCTTTATCCAGAAGACCAAAAACAGGCGG
GAACCGTCCCAATTTCAAATTTAGATAGCTTGCCCCGCAAGCAAACTAAAAGTCTATATATACCAGTGTAGACATATTGTTCAGAGAACAAATGGCGAGGTATCCGAAGaaATCGATCAA
GAAGAGGCGGGTTGGGCGAAGGAAGTATGGAAGCAAGGCGGCAACCAGTCACGACTACTCGTCGTTAGGTTCAATATTGGTTCCTGAAAATACCGTCAAGGTATTTAGGATTGAGCCTAC
TGATAAAACATTACCCAGATATTTTATCTGGAAAATGTTTATGCTTTTGGTGTGCAAGGTGAAGCCCGGAAGAATACTTCACTGGGCTATGATTAAAAGTTCATGGGAAATCAACCAGCC
GACTACATGTCTGGAAGCACCAGGTTTATTTATAAAACCTGAACATAGCCATCTGGTGAAACTGGTATGCAGTGGGGAACTTGAAGCAGGAGTCGCAACAGGGACATCAGATGTTGAATG
TCTTCTAAGGAAGACAACCGTGTTGAGGAAGAATGTAACAGAGGTGGATTACTTGTATTTGGCATTTTATTGTAGTGCTGGAGTTAGTATTAACTACCAGAACAGAATTACATATCATGT
ATGATATGTTTATGTAAACATAAACCTTTGTATGGAATAATGACCAAATAACATACAAGACGCTATGAAATACAACACGCTATGACAAACAGGGAAAAATGAAGAATCGGGGGTTGATTG
GTCTATCGTATCGCTTAAGGGCCGCAGGCCCGTTGAAATGATTCTTAATAAAACAAATATACATGATACGGATAGTTGAATATATAAACAACGATGTATAAATACAACAGAATGTTGTAT
ACTAATTAAAATAATGAGAAGAAAAGAATATTTGTGAAGGATAAGCATCAGAACCACCACTTTAGTGGTGGGTCATATGTCCCGAGTTAGTGCGCCACGTAAGCGCTGGGGCTTAT

SNPs (all minor – ca. 15-20% reads)

DNA-U3
37-44 SNPs to KY427065 MF039869 MF039875 MF039881 (**Thailand)**

U3 protein (39 aa): v1=v2 **0 aa** to KY427065 (**Thailand)**

>U3_alyu34_v1
TATTACCCCCAGCCCCCAGGACGGGACATGGGCTTTTTAAATGGGCCGAGAGAGTTTGAACAGTTCAGTATCTTCGTTATTGGGCCATCTGGCCCAATAATTAAGAGAACGTGTTCAAAA
TCGGGGTTTGACCGAAGGTCAAGGTAGACGGTCAACAATATTCTGGCTTGCGGAGCAAGCAACAcGAATTAAATATTTAATTCgTATGACACGTGGACGGACCGAAATACTCCTGTATCT
CTATAAATAGCcTAAATCTGGCTTGGATAATTGCTCTCGCTCTTCTGTCAAAGcTGTTGTGTTGAGGCGGAAGATCGCCATCGGCGATCATCGGACGAAAAGCTGCAAGAGAGACGGAGA
ACCATGCTaCGAAGCGTATATCGGGTATTTATAGACTTCTAGCGCAGCTAGAAGTTTCCTTGTACTTGtTATTGTATTTTGTAAATTACGAAGAAATTCGTATATTGATAATAATAAAAC
ATCTGGGATTGTTAATGTTTACATTAACcAGTATTAATAATGTACAATAACAAATATTGTATGAGGAACGAATACAAGAATATTAAAATATGAGGAGCGTAGCGTGATAAACAGGTGTTT
AAGGTATAATTAAAaTAATTATGCAAaGTaATGATAATACGGTATAAGTTGAAGTATGAGGTGAAAGAGGAGATATTAGAATATTAAAAACCCAATTATATTATTTATAATACATACAAC
ACGCTATGACAAACGGGGAAAAAAGAAAAATCGGGGGTTGATTGGTCTATCCTAACGATTAAGGGCCGCAGGCCCGTCAAGATGGATGAACGGTTAGATTTGATTGCTTAGCCACGAAGA
AAGGAATCGTTTTGGGACCACAGACAAGACAGCTGTCACTACTTTTAAAAAATAATATAATAACCAATTGACGATAGTACCCCTCCCAAAGATGACGTATAGGGGTGTCCCGAGGTAATT
TAACATAGCTCTGACAAGAGATAGTGGACGTTGGATGCCACGATCGGACGATCGTGGTTTGATGAACcAATGATGACGTAGGGGCTGGGGCTTAT

A6125C (59313/127758) **T/G** (50157/109485) **T/C** (45228/94445) **G/C** (54292/77699) **G/A** (46608/101167) **A/T** (31236/63372) **T/C** (20182/42722) **T/A** (26100/4720) **T/A** (27311/49528) **T/A** (27243/47228) **C/A** (26625/48191) **A/T** (77680/18847) **T/A** (61736/33152) **G/C** (152398/44365) **T/C** (69631/143453)

>U3_alyu34_v2
TATTACCCCCAGCCCCCAGGACGGGACATGGGCTTTTTAAATGGGCCGAGAGAGTTTGAACAGTTCAGTATCTTCGTTATTGGGCCATCTGGCCCAATAATTAAGAGAACGTGTTCAAAA
TCGGGGTTTGACCGAAGGTCAAGGTAGACGGTCAACAATATTCTGGCTTGCGGAGCAAGCAACAaGAATTAAATATTTAATTCtTATGACACGTGGACGGACCGAAATACTCCTGTATCT
CTATAAATAGCtTAAATCTGGCTTGGATAATTGCTCTCGCTCTTCTGTCAAAGgTGTTGTGTTGAGGCGGAAGATCGCCATCGGCGATCATCGGACGAAAAGCTGCAAGAGAGACGGAGA
ACCATGCTgCGAAGCGTATATCGGGTATTTATAGACTTCTAGCGCAGCTAGAAGTTTCCTTGTACTTGaTATTGTATTTTGTAAATTACGAAGAAATTCGTATATTGATAATAATAAAAC
ATCTGGGATTGTTAATGTTTACATTAACtAGTATTAATAATGTACAATAACAAATATTGTATGAGGAACGAATACAAGAATATTAAAAATGAGGAGCGTAGCGTGATAAACAGGTGTTTA
AGGTATAATTAAAtTAATTATGCAAtGTcATGATAATACGGTATAAGTTGAAGTATGAGGTGAAAGAGGAGATATTAGAATATTAAAAACCCAATTATATTATTTATAATACATACAACA
CGCTATGACAAACGGGGAAAAAAGAAAAATCGGGGGTTGATTGGTCTATCCTAACGATTAAGGGCCGCAGGCCCGTCAAGATGGATGAACGGTTAGATTTGATTGCTTAGCCACGAAGAA
AGGtATCGTTTTGGGACCACAGACAAGACAGCTGTCACTACTTTTAAAAAATAATATAATAACCAAaTGACGATAGTACCCCTCCCAAAGATGACGTATAGGGGTGTCCCGAGGTAATTT
AACATAGCTCTGACAAGAGATAGTGGACGTTGGATcCCACGATCGGACGATCGTGGTTTGATGAACtAATGATGACGTAGGGGCTGGGGCTTAT

ALYU-35 - **Musa sp.** - **Vietnam**

Virome
1. **BBTV** six components
2. **BBTA6** alyu35 - new alphasatellite named here Banana bunchy top alphasatellite 6, genus *Banaphisatellite*

BBTA6
Best matches to MF510475 MF510474 75% coverage 66.6% identity Faba bean necrotic yellows virus associated alphasatellite 2; AJ132187 AJ005966 74% coverage 66.3-66.6% identity Faba bean necrotic yellows C9 alphasatellite; MK291270 68% coverage 64.6% identity Sophora yellow stunt alphasatellite 3

Rep protein best matches to ARI50297 100% coverage 61% identities 77% positives Sophora alopecuroides yellow stunt alphasatellite; P0CK6 100% coverage 60% identities 77% positives Faba bean necrotic yellows C9 alphasatellite; ATU31571 100% coverage 60% identities 77% positives Faba bean necrotic yellows virus associated alphasatellite 2

>BBTA6_alyu35
TATTACCCGACTTTGGCGCAGGGCGCACTATAAAAGATGTCTGTGAGAAATTGGGTCTTCACACGCAATTTCGAAGGCGAAAGACCAGAGCTCCTGTTCGGCGGCGAAACTCAATACGCC
GCTTGGCAACATGAAAGAAAGAATCACGACCACCTACAGGGCGTGATTCAGTTGAAGAAGAAGACCCGACTGAACGCAGTGAAGAGCATAATCGGTGGAAATCCACATGTCGAACCCATG
CGAGCGTCTTTCGACGATGCCCTAGCATACGTCATGAAAGACGAAACCCGAATCGATGGTCCATGGGAATTCGGTATTCGAATCCGTAAGGGTTCGAATAAACGAAAGCTGTTAGAAATA
CTGGAGGATTCGGACAACGAAGTCCTTCAACCCCAAAAATACAGACGAGCCATGGCCAAACAGGCCATGAGTGAGTCTAAGAAGAAAGCTGCTGAAGAAGGATTTAGTTATGAACTGCGA
GAGTGGCAAGTGCAATTGACGACGTTGTTGGAAGAAGAACCCGATAACAGAACAATAATTTGGGTATATGGACCAAAGGGTGGAGAAGGTAAAACAGAGTTCGCTAAACATTTAGGTTTA
AAGGATGGTTGGACATATTTGCCCGGTGGTGAACTCAAAGATATGATGTACCTGTTATCAAAGGACATGAAAAATAATGTTGTAATTGATTTCCCCCGTTGTACCAAGGAATTTATTTCA
TATAAATTCCTTGAGATGGTTAAGAATAGAACTATATATAGTTATAAGTATGAACCAGTTGGTTCAATCGTATGTAATAAGGTTCATGTAGTGGTCTTCAGTAATGAGGAGCCAGATTAT
GTAAAATTATCATTGGATCGAATAAAGTTGATTTCTTGCTGAACACGCTATGAAATACAACACACTATGAAATACTACACGGGTAAGATTGTGTATATTAACGATATTGGGCCGAAGGCC
CAAGCCCAAGCCCATGCCCCCAAAATGAACGGTCAGAATTGATTGCTTCCCCACGAAGCAAACAAAAAATGCGTGGTGAAGAATTCGCCACGTCATTGGTTACTGTTATCCTTGGCGCAC
TATATATAGGTGCGCCAAAGTCGGCCATAG

DNA-C
24-29 SNPs to KM607098 KM607099 (**China**). **26-32 SNPs** to KY427060 MF039864 MF039876 MF039870 (**Thailand**)

C protein (161 aa): **1 aa** to KY427060 (**Thailand**) **2 aa** to KM607098 KM607099 (**China**) MF039864 (**Thailand**)

>C_alyu35
TATTACCCCCCCTGCTCGGGACGGGACATCACGTGCATCAAACAAATGCACGTGACAAAGCAGTAGCTTGTAGCGAAAGACAACCATCACTATCACAAAAGTGAACGGAATATTCGTTGC
TTAGGGACTAAGCAACGAGTACAGATATTTGTTCGAGAAGCGAAAATGGAGGCTATTTAAACCTGATGGTATTGTGATTTCCGAAATCACTCATCGGAGCAGAAATGGAGTTCTGGGAAT
CGTCTGCCATGCCAGACGATGTCAAGAGAGTGATCAAGGAAATATATTGGGAGCATCGAAATAAACTTCTGTTCTGTCAGAAGTTGAAAGGCTGTGTAAGAAGGATTCTGGAGCATGGAA
ATCAAGATGATGCCCTAGACGCAGTGAAGGAGATGAAGACTTCTATTATTCGCTATAGCGAATATTTGAAGAAACCTTGTGTGGTATTGTGTTTTAGTACTAATAAATCTATTGCATATA
GGTTAAACACAATGGTGTTCTTTTATCATGAATATATTGAAGACCTAGGTGGTGACTACTCGGTATATCAAGATCTATTCTGTGATGAGGAACTTCCTTATTCATCGACAGAAGAAGAAG
ATGAAGAAGTAATATACAGGAATGTTATCATGGCATCGACAGAAGAGAAGATCTCTTGGAGTGAATGTCAAAAGATAGTTATATCAGATTATGATGTAACATTACTGTAATGAATATCCA
TTATCATAAATAAAATAATGGAATGATGATTATGTATTCTAGTATATATACATAATGGTATACGTATAGCATAAAATACATAAACCAACATACAACACACTATGACAAACGGGGAAAAAT
GAAGAATCGGGGGTTGATTGGGCTATCTTAACGATTAAGGGCCGCAGGCCCGTTTAAATATGTGGTGGACGAAGTCCACAACACAAAAAAAGTGATCAGAACAATGGAATATAATGAGCT
GGCAACGTAGGGACCATGTCCCGAGTTAGTGCGCCACGTGAGCAGGGGGGCTTAT

DNA-M
26-31 SNPs to KM607239 KM607240 (**China**). **49-52 SNPs** to KY427061 MF039865 MF039876 MF039871 (**Thailand**)

M protein (117 aa): **1 aa** to KC581796 (**Thailand**) **2 aa** to MF039871 MF039865 MF039877 KY427061 (**Thailand**)

>M_alyu35
TATTACCCCCAGCGCTCAGGACGGGACATCACGTGCAACTAACAAATGCACGTGACTGATATATACTACATAACGGTTTAACGAACCGTTATATAATGTTATAACGATAAGTCACGTGTG
ATAGAGACATGCACGTGACATAGTCAAATGTATTGAATAAACATTTGACGTCCGGATGCTTCCGACGGAAGCTTAGGTTACTTCGTGGCGAAGCAAAACATTTATAAAATGGCCTGGATT
GCTGCCTATAAATAGGCAAGCAAGGAAATGGCATTAACAACAGAGCGAGTGAAACTATTCTTTGAATGGTTTCTGTTCATCGGTGCAATATTTATTGCGATAACAATATTATATATATTG

TTGGCGTTGCTCTTTGAGGTCCCCAAGTATATTAAGGATGTTGTGAGGTATCTCGTAGAATACCTGACCAGAAGACGTGTATGGATGCAGAGAACGCAGTTGACGGAGGCAACAGGAGAT
GTAGAGCTCGGCAGAGGTAGTGTGGAAGACAGACGGGATCAACAACCGGCTGTCATTCCACATGCAACACAGGTTATCCCTTCGCATCAACCTAGAAGGGATGATCAAGGAAGACGAGGA
AACGCCGGACCGATGTTTTAATACACGGTATTGTAATATACGAAATATAAATGGATAATGATATGTATTGTGAAACATAATATACGTGAAACATAATATATGTTTGATAATTTACATATA
GTAATATGTAAAATGTATACGAGTGTTGTATTTATAGAACATACAACACGCTATGACAAACAGGGAAAAATGAAGAATCGGGGGTTGATTGGTCTATCGTATCGCTTAAGGGCCGCAGGC
CCGTTGAAATGATTCTTAATAAAACAAATATACATGATACGGATAATTGAATATATAAACAACGAAGTATAAATACAACAGAATGTTGTATACTAATTAAAATAATGAGAAGATAAGTAT
ATTTGTGAGGGATGATGATCACAACCACCACTTTAGTGGTGGGTCATATGTCCCGAGTTAGTGCGCCACGTAAGCGCTGGGGCTTAT

DNA-N
34 SNPs to KM607387 KM607385 (**China**). **37-41 SNPs** to MF039872 MF039878 KY427062 MF039866 (**Thailand**)

N protein (154 aa): 0 aa to MF039872 MF039878 KY427062 (**Thailand**) KM607387 KM607385 (**China**) **1 aa** to MF039866 (**Thailand**)

>N_alyu35
TATTACCCCCCGTGCTCGGGACGGGACATGACGTCAGCATAGATTATAATGGGCCAACTAAAGGCCCATTTAATAGAATAGGCGGGCTTTTGACATATTTCAAAGGCCCAGCCTGGAAGT
GGATAATGTCACGTGCCGAATAAGAGGTTGCTTCGCCTCGAAGCAATCCGAATAAATGTTGCGTATTCAATACGCAACTGAAGTCTATTAATATGGATGTCTCTGCCGAATAAATCAGAG
CGTAAGCGAAGCAGAAGCG**ATG**GATTGGGCAGAATCACAATTCAAGACATGTACTCATGGTTGCGATTGGAAGACGATATCATCGGATTCATCCGAGAATCGACAATATGTACCTTGCGT
CGACGCTGGCAGTGGAAGAAAGTCTCCTCGCAAGGTACTGCTTAGATCTATTGAAGCTGTGTTTAATGGAAGCTTCAAAGGAAATAACAGGAATGTTCGTGGGTTTATATACGTATCAAT
ACGAGACGATGATGGAGAAATGCGTCCAGTACTCATAATACCATTCGGAGGATATGGATATCATAATGATTTCTATTATTTCGAAGGGAAGGGGAAAGTTGAATGTGATATATCATCAGA
TTATGTAGCGCCAGGAGTCGATTGGAGCAGAGACATGGAAGTTAGTATTAGCAACAGCAACAACTGTAATGAATTATGTGATCTGAAGTGTTATGTTGTTTGTTCGTTAAGAATAAAGGA
ATAACAGATGTGCTGTAATGATTATTAATAAAACATATTTTCATGTAATTGATAGTTGTATAAAACATACAACACACTATGAAATACAACACGCTATGACAAACAGGGAAAAATGAAAAA
TCGGGGGTTGATTGGTCTATCGTATCGCTTAAGGGCCGCAGGCCCGTTGAAAAATAATAATCGAATTATAAACGTTTGATAATAATCAGAGAAAGATGATAAGCATCCAAAAACATAGAC
GAAGTATATGGCTGTATAATATAAAAGAAGCATATAAATAAAATATGTGAACTAATCTCTGATTGGTTCAGAACGTAACCCCACCAACTTTATGTTGGTGGAAATGTCCCGATGACGTAA
GCACGGGGGACTAT

17 nt insert downstream of ORF identical to that in alyu23_Gabon

DNA-R
12 SNPs to AF416475 AB113660 (**Viet Nam**). **14 SNPs** to MF039879 (**Thailand**). **14-16 SNPs** to KM607677 KM607678 KM607676 KM607679 (**China**). **15 SNPs** to AB113659 (**Viet Nam**). **16-17 SNPs** to MF039873 MF039867 (**Thailand**)

R protein (286 aa): 1 aa to AF416464 (**Vietnam**) **2 aa** to KM607676 KM607677 KM607679 (**China**) **3 aa** to AB113660 (**Vietnam**) MF039879 (**Thailand**)…

>R_alyu35
TATTACCCCCAGCGCTCGGGACGGGACATTTGCATCTATAAATAGACCTCCCCCCCCTCCACTCCAAGATCATCATCGTCGACAGAAATGGCGCGATATGTGGTATGCTGGATGTTCACC
ATCAACAATCCCGCTTCACTACCAGTGATGCGGGAAGAGTATAAATATATGGTATATCAAGTGGAGAGGGGACAGGAGGGTACTCGTCATGTGCAGGGATACGTCGAGATGAAGAGACGA
AGTTCTCTGAAGCAGATGAGAGGCTTCTTCCCTGGCGCACACCTTGAGAAACGAAAGGGAAGCCAAGAAGAAGCACGGGCATACTGTATGAAGGAAGACACAAGAATCGAAGGTCCCTTC
GAGTTTGGTGCCTTTAAATTGTCATGTAATGATAATTTATTTGATGTCATACAGGATATGCGTGAAACGCACAAACGGCCTTTGGAGTATTTATATGAGTGTCCAAATACCTTCGACAGA
AGTAAGGATACATTATACAGAGTTCAAGCAGAGTTAAATAAAACGAAGGCGATGAATAGCTGGAAAACATCCTTCAGTTCTTGGACATCGGAAGTTGAAAATATTATGGCGGAGCCATGT
CATCGAAGGATAATTTGGGTCTATGGCCCAAATGGAGGAGAAGGAAAGACAACTTATGCAAAATATTTAATGAAGACGAAGAATGCGTTTTATTCGCCAGGAGGAAAATCATTGGATATA
TGTAGATTGTATAATTATGAGGAAATAGTTATATTTGATATTCCCAGATGCAAAGAGGAATATTTAAACTATGGTTTATTAGAAGAATTTAAAAATGGAATTATTCAAAGCGGGAAATAT
GAACCCGTTTTGAAAATTGTAGAATATGTGGAAGTCATTGTAATGGCTAACTTCCTTCCGAAGGAAGGAATCTTTTCAGAAGATCGAATAAAGCTAGTTGCTTGCTGAACACGCTATGAC
AATCGTACGCTATGACAAAAGGGGAAAAGCAAAGATTCGGGGGTTGATTGGGCTATCCTAACGATTAAGGGCCGCAGGCCCGTCAAGATGGACGGCTAGATCAGATGTCCCGAGTTAGTG
CGCCACGTAAGCGCTGGGGCTTAT

DNA-S
15 SNPs to AF148945 (**Viet Nam**). **16-18 SNPs** to KM607534 KM607535 KM607536 KM607533 (**China**). **17-20 SNPs** to AB113662 AB113661 (**Viet Nam**). **21-27 SNPs** to MF039874 KY427064 (**Thailand**)

S protein (170 aa): 0 aa to AY337715 AY494786 HQ378191 (**China**) FJ787433 FJ787434 (**Philippines**). **1 aa** to several isolates

>S_alyu35
TATTACCCCCAGCGCTCGGGACGGGACATGGGCTAATGGATCATGGAATAAGGGCCCATCGGGCCCGTTAAGATGGGTTTTGGGCTTATGGGCTTTATCCATAAGACCAAAAACAGGCGG
GAACCGTCCCAATTTCAAATTTAGATAGCTTGCCTCGCAAGCAAACTAAAAGTCTATATATACCAGTGTAGACATATTGTTCAGATAACAAATGGCGAGGTTTCCGAAGAAATCGATCAA
GAAGAGGCGGGTTGGGCGACGGAAGTATGGAAGCAAGGCGGCAACCAGTCACGACTACTCGTCGTTAGGTTCAATATTGGTTCCTGAAAATACCGTCAAGGTATTTAGGATTGAGCCTAC
CGATAAAACATTACCCAGATATTTTATCTGGAAAATGTTTATGCTTTTGGTGTGCAAGGTAAAGCCCGGAAGAATACTTCACTGGGCTATGATTAAAAGTTCATGGGAAATCAACCAGCC
GACTACATGTCTGGAAGCACCAGGTTTATTTATAAAACCTGAACATAGCCATCTGGTGAAACTGGTATGCAGTGGGGAACTTGAAGCCGGAGTCGCAACAGGGACATCAGATGTTGAATG
TCTTCTAAGGAAGACAACCGTGTTGAGGAAGAATGTAACAGAGGTGGATTACTTGTATTTGGCATTTTATTGTAGTGCTGGAGTTAGTATTAACTACCAGAACAGAATTACATATCATGT
ATGATATGTTTATGTAAACATAAACCTTTGTATGGAATAATGACCAAATAACATACAACACACTATGAAATACAACACGCTATGACAAACGGGGAAAAATGAAGAATCGGGGGTTGATTG
GTCTATCGTATCGCTTAAGGGCCGCAGGCCCGTTGAAATGATTCTTTATAAAACAAATATACAGGATACGGATAGTTGAATATATAAACAACGATGTATAAATACAACAGAATGTTGTAT
ACTAATTAAAAAAATGAGAAGAAAAGGATATTTGTGAAGGATAAGCATCAGAACCACCACTTTAGTGGTGGGTCATATGTCCCGAGTTAGTGCGCCACGTAAGCGCTGGGGCTTAT

DNA-U3
37-45 SNPs to KY427065 MF039881 MF039875 MF039869 (**Thailand**)

U3 protein (39 aa): 0 aa to KY427065 (**Thailand)**

>U3_alyu35
TATTACCCCCAGCCCCCAGGACGGGACATGGGCTTTTTAAATGGGCCGAGAGAGTTTGAACAGTTCAGTATCTTCGTTATTGGGCCATCTGGCCCAATAATTAAGAGAACGTGTTCAAAA
TCGGGGTTTGACCGAAGGTCAAGGTAGACGGTCAACAATATTCTGGCTTGCGGAGCAAGCAACACGAATTAAATATTTAATTCGTATGACACGTGGACGGACCGAAATACTCCTGTATCT
CTATAAATAGCTTAAATCTGGCTTGGATAATTGCTCTCGCTCTTCTGTCAAAGCTGTTGTGTTGAGGCGGAAGATCGCCATCGGCGATCATCGGACGAAAAGCTGCAAGAGAGACGGAGA
ACCATGCCACGAAGCGTATATCGGGTATTTATAGACTTCTAGCGCAGCTAGAAGTTTCCTTGTACTTGATATTGTATTTTGTAAATTACGAAGAAATTCGTATATTGATAATAATAAAAC
ATCTGGGATTGTTAATGTTTACATTAACCAGTATTAATAATGTACAATAACAAATATTGTATGAGGAACGAATACAAAAATATTAAAATATGAGGAGCGTAGCATGATAAACAGGTGTTT
AAGGTATAATTAAATTAATTATGCAATGTCATGATAATACGGTATAAGTTGAAGTATGAGGTGAAAGAGGAGATATAAGAATATTAAAAACCCAATTATATTATTTATAGGACATACAAC
ACGCTATGACAAAAGGGGAAAAAAGAAAAATCGGGGGTTGATTGGTCTATCCTAACGATTAAGGGCCGCAGGCCCGTCAAGATGGATGAACGGTTAGATTTGATTGCTTAGCTACGAAGA
AAGGAATCCTTTTGGGACCACAGACAAGACAGCTGTCACTACTTTTAAAAAATAATATAATAACCAAATGACGATAGTACCCCTCCCAAAGATGACGTATAGGGGTGTCCCGAGGTAATT
TAACATAGCTCTGACAAGAGATAGTGGACGTTGGATGCCACGATCGGACGATCGTGGTTTGATGAACTAATGATGACGTAGGGGCTGGGGCTTAT

ALYU-36 - **Musa sp.** - **Vietnam**

Virome
1. **BBTV** six components
3. **BBTA2** alyu36 isolate of Banana bunchy top alphasatellite 2, genus *Muscarsatellite*

BBTA2
best match to EU430730 100% coverage 99% identity; NC_038953 100% coverage 97% identity; MG545616 (100% coverage, 97% identity

Rep protein best matches to ACB86656 100% coverage 99% identities (1 aa), 100% positives; AZL93963 100% coverage 99% identities (2 aa) 99% positives; YP_009508281 100% coverage, 94% identities (11 gaps in middle) 95% positives

>BBTA2_alyu36
TATTACCCACCTCCGCGCACTACCTCCGCGCACCTATAAAATGTCTGCCTCTCGTTGGACATTTACGCTTCACTATTCCGACGCAACGGAGCGAGGCAAATTCCTCGCGACTTTGAAGGA
GGAAGATGTGCACTACGCCGTCGTCGGCGACGAAACTGCTCCGAATACTGGTCGGAAACATCTTCAAGGATATCTTTCCTTGAAGAAACGTTTTCGTATTAGCGGAATAAAGAAGAAATA

TTCGTCGAGAGCGCATTGGGAGAAAGCTCGAGGATCAGACTACGACAACAAGGCGTACTGTTCCAAAGAAGCCCTAATTCTTGAATTAGGGGTTCCTTGCCAAACAGGTTCGAATAAGCG
TAAATTAGCAGATATGGTTACAAGATCACCGGAACGAATGAAAATTGAACAGCCAGAGATATTTCACCGATACGCATCGGTGAAGAAGATGAAAGAATTCAAAGAAAGGTATGTCTATCC
TATCCTCGATAGGCCATGGCAGGTACAATTAACGGAGTTAATTGAAGCAGAACCAGATGATCGAACGATCATCTGGGTATTCGGACCAAAAGGGAATGAAGGCAAATCAACGTATGCGAA
GTCATTAATCCAAAAGGATTGGTTCTACACAAGGGGAGGAAAGAAGGAGAACATATTGTTCGCCTACGTAGATGAAGGTTCGACCAAAAACGTTGTATTTGATCTTCCGCGTACAGTACA
AGAATTTATTAATTATGATGTTATCGAAGCACTGAAGGATAGAGTAATCGAGAGTACAAAATACAAGCCTGTGAAGTATTTAGAATTGAATACTGTACATGTAATAGTTATGGCTAATTT
TCTTCCTGATATGTGTAAAATATCTGAAGATCGAATAAAAATAGTTGCTTGCTGAACACGCTATGATAAACGCACGCTATGACAAAGGGGAAAAAGAAAAATCGGGGGTTGATTGGGC
TATCCTAACGATTAAGGGCCGCAGGCCCGTCAAGATGGATCCAATAACCCGATAAGAAGTTAAACGGGTCTAAAACACTTTCTTCGCCCGCAAGCAACCACTTTAACCTCTGCGCACCTA
TATATAGCGGAGGTGGGCTAG

DNA-C
21 SNPs to KY427060 (**Thailand**). **23-25 SNPs** to KM607098 KM607099 (**China**). **24-27 SNPs** to MF039864 MF039876 MF039870 (**Thailand**)

C protein (161 aa): 2 aa to KY427060 (**Thailand**) **3 aa** to KM607098 KM607099 (**China**) MF039864 (**Thailand**)

>C_alyu36
TATTACCCCCCCTGCTCGGGACGGGACATCACGTGCATCAAACAAATGCACGTGACAAAGCAGTAGCTTGTAGCGAAAGATAACCATCACTATCACAAAAGTGAGAGGAATATTCGTTGC
TTAGGGACTAAGCAACGAGGACAGATAATTGTTCGAGAAGCGACAATGGAGGCTATTTAAACCTGATGGTTTTGTGATTTCCGAAATCACTCATCGGAGCAGAAATGGAGTTCTGGGAAT
CGTCTGCCATGCCAGACGATGTCAAGAGAGTGATCAAGGAAATATATTGGGAGCATCGAAACAAACTTCTGTTCTGTCAGAAGTTGAAAGGCTGTGTAAGAAGGATTCTGGAGCATGGAA
ATCAAGATGATGCTCTAGACGCAGTGAAGGAGATGAAGACTTCTATTATTCGCTATAGCGAATATTTGAAGAAACCTTGTGTGGTATTGTGTTTTAGTAATAATAAATCTATTGCATATA
GGTTAAACACAATGGTGTTCTTTTATCATGAATATATTGAAGACCTAGGTGGTGACTACTCGGTATATCAAGATCTGTTTTGTGATGAGGAACTTCCTTATTCTTCGACAGAAGAAGAAG
ATGAAGAAGTAATATACAGGAATGTTATCATGGCATCGACAGAAGAGAAGATCTCTTGGAGTGAATGTCAAAAGATAGTTATATCAGATTATGATGTAACATTACTGTAATGAATATCCA
TTATCATAAATAAAATAATGGAATGATGATTATGTATTCTAGTATAAATACATAATGGTATACGTATAGCATAAAATACATATACCTACATACAACACGCTATGACAAACAGGGAAAAAT
GAAGAATCGGGGGTTGATTGGGCTATCTTAACGATTAAGGGCCGAAGGCCCGTTTAAATATGTGGTGGACGAAGTCCACAACACATAAAAAGTGAGCAGAACAATGGAATATAATGAGCT
GGCAACGTAGGGTCCATGTCCCGAGTTAGTGCGCCACGTGAGCAGGGGGGCTTAT

DNA-M
34-38 SNPs to MF039865 KY427061 MF039876 MF039871 (**Thailand**)

M protein (117 aa): 2 aa MF039865 MF039877 KY427061 (**Thailand**) **3 aa** to KC581796 (**Thailand**)

>M_alyu36
TATTACCCCCAGCGCTCAGGACGGGACATCACGTGCACATAACAAATGCACGTGACTGATATATACTACATAACGGTTTATTGAACCGGTATGTAGTGTTGTAACGTAAAGTCACGTGTG
ATAGTGATATGCACGTGACTATGTCAAATGTATTGAATAAACATTTGACGTCCGGATGCTTCCTACGGAAGCCATGGTTGCTTCGTGGCGAAGCAAAACATTTATATATTGGCATTGGAC
TGCCGCCTATAAATAGGCAGGCAGGGAAATGGCATTAACAACAGAGCGAGTGAAACTATTCTTTGAATGGTTTCTGTTCATTGGTGCAATATTTATTGCGATAACAATATTATATATATT
GTTGGTGTTGCTCTTTGAGGTCCCCAAGTATATGAAGGATGTTGTGAGGTATCTCGTAGAATACCTGACCAGACGACGTGTATGGATGCAGAGAACGCAGTTGACGGAGGCAACAGGAGA
TGTAGAGCTCGGCAGAGGTAGTGTGGAAGACAGACGGGATCAACAACCGGCTGTCATACCACATGCAACACAGGTTATCCCTTCGCATCAACCTAGAAGGGATGAGCAAGGAAGACGAGG
AAACGTTGGACCGATGTTTTAATACACAGTATCTTAATATACGAAATATAAATGGATAATGATAAGTATTGTGAAACATATATATGTGAAACATAATATATGTTTGATAATTTACATATT
GTAATATGTGAATTGTATACGAGTGTTGTATTTATAAAACATACAACACGCTATGACAAACAGGGAAAAATGAAGAATCGGGGGTTGATTGGTCTATCGTATCGCTTAAGGGCCGCAGGC
CCGTTGAAATGATTCTTTATAAAACAAATATACATGATACGGATTGTTGAAATTATAAACAACGATGTATAAATACAACAGAATGTTGTATACTAAATAAAATAATGAGAAGATAAGTAT
ATTTGTGAAGGATATGGATCACAACCACCACTTTAGTGGTGGGTCATATGTCCCGAGTTAGTGCGCCACGTAAGCGCTGGGGCTTAT

DNA-N
16-23 SNPs to MF039872 MF039878 MF039866 KY427062 (**Thailand**). **23 SNPs** to KM607387 KM607385 (**China**)

N protein (154 aa): 0 aa to MF039872 MF039878 KY427062 (**Thailand**) KM607387 KM607385 (**China**) **1 aa** to MF039866 (**Thailand**)

>N_alyu36
TATTACCCCCGTGCTTGGGACGGGACATGACGTCAGCATAGATTATAATGGGCCAACTAAAGGCCCATTTAATAGAATAGGCGGGCTTTTGACATATTTCAAAGGCCCAGCCTGGAAGT
GGATAATGTCACGTGCCTAATAAGAGGTTGCTTCGCCTCGAAGCAACCCGAATAAATGTTGCGTATTCAATACGCAACTGAAGTCTATTAATATGGATGTCTCTGCCGAATAAATCAGAG
CGTAAGCGAAGCAGAAGCGATGGATTGGGCAGAATCACAATTCAAGACATGTACTCATGGGTGCGATTGGAAGACGATATCATCGGATTCATCCGAGAATCGACAATATGTACCTTGCGT
CGACGCTGGCAGCGGAAGAAAGTCACCTCGCAAGGTACTGCTAAGATCTATTGAAGCTGTGTTTAATGGAAGCTTCAAAGGAAATAACAGGAATGTTCGTGGGTTTATATACGTATCAAT
ACGAGACGATGATGGAGAAATGCGTCCAGTACTCATAATACCATTCGGAGGATATGGATATCATAATGATTTCTATTATTTCGAAGGGAAGGGGAAAGTTGAATGTGATATATCATCAGA
TTATGTAGCGCCAGGAGTCGATTGGAGCAGAGACATGGAAGTTAGTATTAGTAACAGCAACAACTGTAATGAATTATGTGATTTGAAGTGTTATGTTGTTTGTTCGTTAAGAATAAAGGA
ATAACAGATGTGCTGTAATGATTATTAATAAAACATATTTTCATGTAATTGATAGTTGTATAAAACATACAACACGCTATGACAAACAGGGAAAAATGAAAAATCGGGGGTTGATTGGTC
TATCGTATCGCTTAAGGGCCGCAGGCCCGTTGAAAAATAATAATCGAATTATAAACGTTTGATAATAATCAGAGATAGATGATAAGCATCCAAAAACATAGACGAAGTATATGGCTGTAT
AATATAAAAGAAGCATATAAATAAAATATGTAAACTAATCTCTGATTGGTTTAGAATGTAACCCCACCAACTTTATGTTGGTGGAAATGTCCCGATGACGTAAGCACGGGGGACTAT

DNA-R
10-12 SNPs to MF039879 MF039873 (**Thailand**). **12-14 SNPs** to KM607677 KM607678 KM607676 KM607679 (**China**). **13-14 SNPs** to MF039867 KY427063 (**Thailand**). **20 SNPs** to AF416475 AB113660 (**Viet Nam**)

R protein (286 aa): 0 aa to KM607676 KM607677 KM607679 (**China**) **1 aa** to MF039879 (**Thailand**) AF41646 AB113660 (**Vietnam**)…

>R_alyu36
TATTACCCCCAGCGCTCGGGACGGGACATTTGCATCTATAAATAGACCTCCCCCCCCTCCACTACAAGATCATCATCGTCGACAGAAATGGCGCGATATGTGGTATGCTGGATGTTCACC
ATCAACAATCCCGCTTCACTACCAGTGATGCGGGATGAGTTTAAATATATGGTATATCAAGTGGAGAGGGGACAGGAGGGTACTCGTCATGTGCAGGGATACGTCGAGATGAAGAGACGA
AGTTCTCTGAAGCAGATGAGAGGCTTCTTCCCTGGCGCACACCTTGAGAAACGAAAGGGAAGCCAAGAAGAAGCACGGGCATACTGTATGAAGGAAGACACAAGAATCGAAGGTCCCTTC
GAGTTTGGTGCCTTTAAATTGTCATGTAATGATAATTTATTTGATGTCATACAGGATATGCGTGAAACGCACAAACGGCCTTTGGAGTATTTATATGAGTGTCCAAATACCTTCGATAGA
AGTAAGGATACATTATACAGAGTTCAAGCAGAGTTGAATAAAACGAAGGCGATGAATAGCTGGAAAACATCCTTCAGTTCTTGGACATCGGAAGTTGAAAATATTATGGCGGAGCCATGT
CATCGAAGGATAATTTGGGTCTATGGCCCAAATGGAGGAGAAGGAAAGACAACCTATGCAAAATATTTAATGAAGACGAAGAATGCGTTTTATTCGCCAGGAGGAAAATCATTGGATATA
TGTAGATTGTATAATTATGAGGAAATAGTTATATTTGATATTCCTAGATGCAAAGAGGAATATTTAAACTATGGTTTATTAGAAGAATTTAAAAATGGAATTATTCAAAGCGGGAAATAT
GAACCCGTTTTGAAAATTGTAGAATATGTGGAAGTCATTGTAATGGCTAACTTCCTTCCGAAGGAAGGAATCTTTTCAGAAGATCGAATAAAGCTAGTTGCTTGCTGAACACGCTATGAC
AATCGTACGCTATGACAAAAGGGGAAAAGCAAAGATTCGGGGGTTGATTGTGCTATCCTAACGATTAAGGGCCGCAGGCCCGTCAAGATGGACGGTTTGATCAGATGTCCCGAGTTAGTG
CGCCACGTAAGCGCTGGGGCTTAT

DNA-S
9/12 SNPs to AB113661 AB113662 (**Viet Nam**). **16 SNPs** to MF039874 (**Thailand**). **18-22 SNPs** to KM607535 KM607534 KM607536 KM607533 KY427064 (**China**). **22 SNPs** to AF148945 (**Viet Nam**)

S protein (170 aa): 0 aa to AY33771 AY494786 HQ378191 (**China**) FJ787433 FJ787434 (**Philippines**) **1 aa** to several isolates

>S_alyu36
TATTACCCCCAGCGCTCGGGACGGGACATGGGCTAATGGATCATGGAATAAGGGCCCATCGGGCCCGTTAAGATGGGTTTTGGGCTTATGGGCTTTATCCAGAAGACCAAAAACAGGCGG
GAACCGTCCCAATTTCAAATTTAGATAGCTTGCCCCGCAAGCAAACTAAAAGTCTATATATACCAGTGTAGACATATTGTTCAGAGAACAAATGGCGAGGTTTCCGAAGAAATCGATCAA
GAAGAGGCGGGTTGGGCGACGGAAGTATGGAAGCAAGGCGGCAACCAGTCACGACTACTCGTCGTTAGGTTCAATATTGGTACCTGAAAATACCGTCAAGGTATTTAGGATTGAGCCTAC
TGATAAAACATTACCCAGATATTTTATCTGGAAAATGTTTATGCTTTTGGTGTGCAAGGTGAAGCCCGGAAGAATACTTCACTGGGCTATGATTAAAAGTTCATGGGAAATCAACCAGCC
GACTACATGTCTGGAAGCACCAGGTTTATTTATAAAACCTGAACATAGCCATCTGGTGAAACTGGTATGCAGTGGGGAACTTGAAGCCGGAGTCGCAACAGGGACATCAGATGTTGAATG
TCTTCTAAGGAAGACAACCGTGTTGAGGAAGAATGTAACAGAGGTGGATTACTTGTATTTGGCATTCTATTGTAGTGCTGGAGTTAGTATTAACTACCAGAACAGAATTACATATCATGT
ATGATATGTTTATGTAAACATAAACCTTTGTATGGAATAATGACCAAATAACATACAAGACGCTATGAAATACAACACGCTATGACAAACAGGGAAAAATGAAGAATCGGGGGTTGATTG
GTCTATCGTATCGCTTAAGGGCCGCAGGCCCGTTGAAATGATTCTTTATAAAACAAATATACATGATACGGATTGTTGAAAATATAATCAACGATGTATAAATACAACAGAATGTTGTAT
ACTAATTAAAAAAATGAGAAGAAAAGAATATTTGTGAAGGATAAGCATCAGAACCACCACTTTAGTGGTGGGTCATATGTCCCGAGTTAGTGCGCCACGTAAGCGCTGGGGCTTAT

DNA-U3
37-43 SNPs to MF039881 MF039875 KY427065 MF039869 (**Thailand**)

U3 protein (39 aa): 0 aa to KY427065 (**Thailand)**

```
>U3_alyu36
TATTACCCCCAGCCCCCAGGACGGGACATGGGCTTTTTAAATGGGCCGAGAGAGTTTGAACAGTTCAGTATCTTCGTTATTGGGCCATCTGGCCCAATAATTAAGAGAACGTGTTCAAAA
TCGGGGTTTGACCGAAGGTCAAGGTAGACGGTCAACAATATTCTGGCTTGCGGAGCAAGCAACACGAATTAAATATTTAATTCGTAAGACACGTGGACGGACCGAAATACTCCTGAATCT
CTATAAATAGCTTAAATCTGGCTTGGATAATTGCTCTCGCTCTTCTGTCAAAGCTGTTGTGTTGAGGCGGAAGATCGCCATCGGCGATCATCGGACGAAAAGCTGCAAGAGAGACGGAGA
ACCATGCTACGAAGCGTATATCGGGTATTTATAGACTTCTAGCGCAGCTAGAAGTTTCCTTGTACTTGATATTGTATTTTGTAAATTACGAAGAAATTCGTATATTGATAATAATAAAAC
ATCTGGGACTGTTAATGTTTACATTAACCAGTATTAATAATGTATAATAACAAATATTGTATGAGGAACGAATACAAGAATATTAAAATATGAGGAGCGTAGCGTGATAAACAGGTGTTT
AAGGTATAATTAAATTAATTATGCAATGTCATGATAATACGGTATAAGTTGAAGTATGAGGTGAAAGAGGAGATATTAGAATATTAAAAACCCAATTATATTATTTATAATACATACAAC
ACGCTATGACAAACGGGGAAAAAAGAAAAATCGGGGGTTGATTGGTCTATCCTAACGATTAAGGGCCGCAGGCCCGTCAAGATGGATGAACGGTTAGATTTGATTGCTTAGCCACGAAGA
AAGGAATCGTTTTGGGACCACTGACAAGACAGCTGTCACTACTTTTAAAAAATAATATAATAACCAATTGACGATAGTACCCCTCCCAAAGATGACGTATAGGGGTGTCCCGAGGTAATT
TAACATAGCTCTGACAAGAGATAGTGGACGTTGGATGCCACGATCGGACGATCGTGGTTTGATGAACTAATGATGACGTAGGGGCTGGGGCTTAT
```

ALYU-37 - Musa ABB bluggoe type rosé - Vietnam

Virome

1. **BBTV** six components
2. **BBTA2** alyu37 isolate of Banana bunchy top alphasatellite 2, genus *Muscarsatellite*
3. **BBTA5** partial: 275 nt contig to alyu32_38_40; above cross-contamination threshold -> low titre infection?

Minor genetic variants in M, N, U and Babu2

BBTA2

Best match to EU430730 100% coverage 98% identity; MG545616 100% coverage 97.5% identity; NC_038953 100% coverage 96% identity

Rep protein best matches to AZL93963 100% coverage 99% identities (2 aa) 100% positives; ACB86656 100% coverage 99% identities (1 aa) 99% positives; YP_009508281 100% coverage 94% identities (11 gaps in middle) 95% positives

```
>Babu2_alyu37
TATTACCCACCTCCGCGCACTACCTCCGCGCACCTATAAAATGTCTGCCTCTCGATGGACATTTACGCTTCACTATTCCGACGCAACGGAGCGAGGCAAATTCCTCGCGACTTTGAAGGA
GGAAGATGTGCACTACGCCGTCGTCGGCGACGAAACTGCTCCGAATACTGGTCGAAAACATCTTCAAGGATATCTTTCCTTGAAGAAACGTTTTCGTATTAGCGGAATAAAGAAGAAATA
TTCGTCGAGAGCGCATTGGGAGAAAGCTCGAGGATCAGACTACGACAACAAGGCGTACTGTTCCAAAGAAGCCCTAATTCTTGAATTAGGGGTTCCTTGCCAAACAGGTTCGAATAAGCG
TAAATTAGCAGATATGGTTACAAGATCGCCGGAACGAATGAAAATTGAACAGCCAGAGATATTTCACCGATACGAATCGGTGAAGAAGATGAAAGAATTCAAAGAAAGGTATGTCTATCC
TATCCTCGATAGGCCATGGCAGGTACAATTAACGGAGTTAATTGAAGCAGAACCTGATGATCGAACGATCATCTGGGTATTCGGACCAAAAGGGAATGAAGGCAAATCAACGTATGCGAA
GTCATTAATCCAAAAGGATTGGTTCTACACAAGGGGAGGAAAGAAGGAGAACATATTGTTCGCCTACGTAGATGAAGGTTCGACCAAAAACGTTGTATTTGATCTTCCGCGTACAGTACA
AGAATTTATTAATTATGATGTTATCGAAGCATTGAAGGATAGAGTAATCGAGAGTACAAAATACAAGCCTGTGAAGTATTTAGAATTGAATACTGTACATGTACTAGTTATGGCTAATTT
TCTTCCTGATATGTGTAAAATATCTGAAGATCGAATAAAAATAGTTGCTTGCTGAACACGCTATGAAATACAACACGCTATGACAAACGGGGAAAAATGAAGAATCGGGGGTTGATTGGG
CTATCCTAACGATTAAGGGCCGCAGGCCCGTCAAGATGGATCCAATAACCCGTTAAGAAGTTAAACGGGTCTAAAACGATAACTTCGCCCGCAAGCAACACCTTTAACCTCTGCGCACCT
ATATATAGCGGAGGTGGGCTAG
```

BBTA5 partial

```
>BBTA5_partial
ACTTAAAGGAGTGGCAAAAGATGGTTATTGAGTTAATCGAAGAACAACCAGACAACAGAACTATTATCTGGGTCTATGGACCTAATGGTGGAGAAGGTAAAACGCAGTTCGCAAAGCATT
ATGGATTACTCAAAGGATGGACATATCTACCAGGTGGTGAACTGAAAGACATGATGTATCTGTTGGCAAAAGAAATTAATAATAACGTTATTATTGATTTCCCCCGATGTACAAAGGATT
TCATATCCTATAAGTTTATTGAGATGGTAAAGAAC
```

DNA-C

23/26 SNPs to KM607098 KM607099 (**China**). **29-35 SNPs** to KY427060 MF039876 MF039864 to MF039870 (**Thailand**)

C protein (161 aa): 1 aa to KY427060 (**Thailand**) **2 aa** to KM607098 KM607099 (**China**) MF039864 (**Thailand**)

```
>C_alyu37
TATTACCCCCCCTGCTCGGGACGGGACATCACGTGCATCAAACAAATGCACGTGACAAAGAGGTAGCTTGTAGCGAAAGATAACCATCACTATCACAAAAGTGAGAGGAATATTCGTTGC
TTAGGGACTAAGCAACGAGGACAGATATATGTTCGAGAAGCGACAATGGAGGCTATTTAAACCTGATGGTTTTGTGATTTCCGAAATCACTCATCGGAGCAGAAATGGAGTTTTGGGAAT
CGTCTGCCATGCCAGACGATGTCAAGAGAGTGATCAAGGAAATATATTGGGAGCATCGAAATAAACTTCTGTTCTGTCAGAAGTTGAAAGGCTGTGTAAGAAGGATTCTGGAGCATGGAA
ATCAAGATGATGCTCTAGACGCAGTGAAGGAGATGAAGACTTCTATTATTCGCTATAGCGAATATTTGAAGAAACCTTGTGTGGTATTGTGTTTTAGTACTAATAAATCTATTGCTTATA
GGTTAAACACAATGGTGTTCTTTTATCATGAATATATTGAAGACCTAGGTGGTGACTACTCGGTATATCAAGATCTGTTTTGTGATGAGGAACTTCCTTATTCGTCGACAGAAGAAGAAG
ATGAAGAAGTAATATACAGGAATGTTATCATGGCATCGACAGAAGAGAAGATCTCTTGGAGTGAATGTCAAAAGATAGTTATATCAGATTATGATGTAACATTACTGTAATGAATATCCA
TTATCATAAATAAAATAATGGAATGATGATTATGTATTCTAGTATAAATACATAATGGTATACGTATAGCATAAAATACATAAACCAACATACAACACGCTATGACAAACGGGGAAAAAT
GAATAATCGGGGGTTGATTGGGCTATCTTAACGATTAAGGGCCGAAGGCCCGTTTAAATATGTGGTGGACGAAGTCCACAACACAAAAAAAGTGATCAGAACAATGGAATATAATGAGCT
GGCAACGTAGGGACCATGTCCCGAGTTAGTGCGCCACGTGAGCAGGGGGGACTAT
```

DNA-M

28-33 SNPs to KM607239 KM607240 (**China**). **51-52 SNPs** to MF039871 KY427061 MF039865 MF039876 (**Thailand**)

M protein (117 aa): 1 aa to KC581796 (**Thailand**) **2 aa** to MF039871 MF039865 MF039877 KY427061 (**Thailand**)

```
>M_alyu37
TATTACCCCCAGCGCTCAGGACGGGACATCACGTGCAACTAACATATGCACGTGACTGATATATACTACATAACGGTTTAATGAACCGTTATATAATGTTATAACGAAAAGTCACGTGTG
ATAGAGACCTGCACGTGACATAGTCAAATGTATTGAATAAACATTTGACGTCCGGATGCTTCCGACGGAAGCTTATGTTACTTCGTGGCGAAGCAAAACATTTATATATTGCCCTGGACT
GCTGCCTATAAATAGGCAAGCAAGGAAATGGCATTAACAACAGAGCGAGTGAAACTATTCTTTGAATGGTTTCTGTTCATCGGTGCAATATTTATTGCGATAACAATATTATATATATTG
TTGGCGTTGCTCTTTGAGGTCCCCAAGTATATTAAGGATGTTGTGAGGTATCTCGTAGAATACCTGACCAGAAGACGTGTATGGATGCAGAGAACGCAGTTTACGGAGGCAACAGGAGAT
GTAGAGCTCGGCAGAGGTAGTGTGGAAGACAGACGGGATCAACAACCGGCTGTCATACCACATGCAACACAGGTTATCCCTTCGCATCAACCTAGAAGGGATGAGCAAGGAAGACGAGGA
AACGCCGGACCAATGTTTTAATACACGGTATTGTAATATACGAAATATAAATTGGTAATGATATCTATTGTGAAACATAATATACGTGAAACATAATATATGTTTGATAATTTACATATT
GTAATATGTAATATGTATACGAGTGTTGTATTTATAGAACATACAACACGCTATGACAAACAGGGAAAAATGAAGAATCGGGGGTTGATTGGTCTATCGTATCGCTTAAGGGCCGCAGGC
CCGTTGAAATGATTCTTAATAAAACAAATATACATGATACGGATAGTTGAATATATAAACAACGAAGTATAAATACAACAAAATGTTGTATACTAATTAAAATAATGAGAAGATAAGTAT
ATTTGTGAGGGATGATGATCACAACCACCACTTTAGTGGTGGGTCATATGTCCCGAGTTAGTGCGCCACGTAAGCGCTGGGGCTTAT
```

DNA-N

11-13 SNPs to MF039872 MF039878 MF039866 (**Thailand**). **14 SNPs** to KM607387 KM607385 (**China**). **15 SNPs** to KY427062 (**Thailand**)

N protein (154 aa): 0 aa to MF039872 MF039878 KY427062 (**Thailand**) KM607387 KM607385 (**China**) **1 aa** to MF039866 (**Thailand**)

```
>N_alyu37
TATTACCCCCCGTGCTTGGGACGGGACATGACGTCAGCATAGATTATAATGGGCCAACTAAAGGCCCATTTAATAGAATAGGCGGGCTTTTGACATATTTCAAAGGCCCAGCCTGGAAGT
GGATAATGTCACGTGCCTAATAAGAGGTTGCTTCGCCTCGAAGCAATCCGAATAAATGTTGCGTATTCAATACGCAACTGAAGTCTATTAATATGGATGTCTCTGCCGAATAAATCAGAG
CGTAAGCGAAGCAGAAGCGATGGATTGGGCAGAATCACAATTCAAGACATGTACTCATGGGTGCGATTGGAAGACGATATCATCGGATTCATCCGAGAATCGACAATATGTACCTTGCGT
CGACGCTGGCAGCGGAAGAAAGTCACCTCGCAAGGTACTGCTAAGATCTATTGAAGCTGTGTTTAATGGAAGCTTCAAAGGAAATAACAGGAATGTTCGTGGGTTTATATACGTATCAAT
ACGAGACGATGATGGAGAAATGCGTCCAGTACTCATAATACCATTCGGAGGATATGGATATCATAATGATTTCTATTATTTCGAAGGGAAGGGGAAAGTTGAATGTGATATATCATCAGA
TTATGTAGCGCCAGGAGTCGATTGGAGCAGAGACATGGAAGTTAGTATTAGTAACAGCAACAACTGTAATGAATTATGTGATCTGAAGTGTTATGTTGTTTGTTCGTTAAGAATAAAGGA
ATAACAGATGTGCTGTAATGATTATTAATAAATCTTATTTTCATGTAATTGATAGTTGTATAAAACATACAACACGCTATGACAAACAGGGAAAAATGAAAAATCAGGGGTTGATTGGTC
```

TATCGTATCGCTTAAGGGCCGCAGGCCCGTTGAAAAATAATAATCGAATTATAAACGTTTGATAATAATCAGAGATAGATGATAAGCATCCAAAAACATAGACGAAGTATATGGATGTAT
AATATAAAACAAGCATATAAATAAAATATGTGAACTAATCTCTGATTGGTGCAGAACGTAGCCCCACTAACTTTAAGTTAGTGGAAATGTCCCGATGACGTAAGCACGGGGGACTAT

DNA-R

10 SNPs to AF416475 AB113660 (**Viet Nam**). **10-12 SNPs** to KM607678 KM607677 KM607676 KM607679 (**China**). **18 SNPs** to AB113659 (**Viet Nam**). **18-21 SNPs** to MF039873 MF039879 KY427063 MF039867 (**Thailand: Chonburi**)

R protein (286 aa): 1 aa to AB113660 (**Vietnam**) KM607676 KM607677 KM607679 (**China**) **2 aa** to several isolates …

>R_alyu37
TATTACCCCCAGCGCTCGGGACGGGACATTTGCATCTATAAATAGACCTCCCCCCTCTCCACTACAAGATCATCATCGTCGACAGAAATGGCGCGATATGTGGTATGCTGGATGTTCACC
ATCAACAATCCCGCTTCACTACCAGTGATGCGGGATGAGTTCAAATATATGGTATATCAAGTGGAGAGGGGACAGGAGGGTACTCGTCATGTGCAGGGATACGTCGAGATGAAGAGACGA
AGTTCTCTGAAGCAGATGAGAGGCTTCTTCCCAGGCGCACACCTTGAGAAACGAAAGGGAAGCCAAGAAGAAGCACGGGCATACTGTATGAAGGAAGACACAAGAATCGAAGGTCCCTTC
GAGTTTGGTGCCTTTAAATTGTCATGTAATGATAATTTATTTGATGTCATACAGGATATGCGTGAAACGCACAAACGGCCTTTGGAGTATTTATATGAGTGTCCAAATACCTTCGATAGA
AGTAAGGATACATTATACAGAGTTCAAGCAGAGTTGAATAAAACGAAGGCGATGAATAGCTGGAAGACATCCTTCAGTGCATGGACATCGGAAGTTGAAAATATTATGGCGGAGCCATGT
CATCGAAGGATAATTTGGGTCTATGGCCCAAATGGAGGAGAAGGAAAGACAACCTATGCAAAGTATTTAATGAAGACGAAGAATGCGTTTTATTCTCCAGGAGGAAAATCATTGGATATA
TGTAGATTGTATAATTACGAGGAAATAGTTATATTTGATATTCCCAGATGCAAAGAGGAATATTTAAACTATGGTTTATTAGAAGAATTTAAAAATGGAATTATTCAAAGCGGGAAATAT
GAACCCGTTTTGAAAATTGTAGAATATGTCGAAGTCATTGTAATGGCTAACTTCCTTCCGAAGGAAGGAATCTTTTCTGAAGATCGAATAAAGCTAGTTGCTTGCTGAACACGCTATGAC
AATCGTACGCTATGACAAAAGGGGAAAAGCAAAGATTCGGGGGTTGATTGGGCTATCCTAACGATTAAGGGCCGCAGGCCCGTCAAGATGGACGGCTTGATCAGATGTCCCGAGTTAGTG
CGCCACGTAAGCGCTGGGGCTTAT

DNA-S

14/15 SNPs to AB113661 AB113662 (**Viet Nam**). **19 SNPs** to MF039874 (**Thailand**). **19-22 SNPs** to KM607535 KM607534 KM607536 KM607533 (**China**). **25 SNPs** to KY427064 (**Thailand**). **25 SNPs** to AF148945 (**Viet Nam**)

S protein (170 aa): 0 aa to AF148068 (**Philippines**) **1-2 aa** to several isolates

>S_alyu37
TATTACCCCCAGCGCTCGGGACGGGACATGGGCTAATGGATCATGGAATAAGGGCCCATCGGGCCCGTTAAGATGGGTTTTGGGCTTATGGGCTTTATCCAGAAGACCAAAAACAGGCGG
GAACCGTCCCAATTTCAAATTTAGATAGCTTGCCCCGCAAGCAAACTAAAAGTCTATATATACCAGTGTAGACATATTGTTCAGAGAACAAATGGCGAGGTTTCCGAAGAAATCGATCAA
GAAGAGGCGGGTTGGGCGACGGAAGTATGCAAGCAAGGCGGCAACTAGTCACGACTACTCGTCGTTAGGTTCAATATTGGTTCCTGAAAATACCGTCAAGGTATTTAGGATTGAGCCTAC
TGATAAAACATTACCCAGATATTTTATCTGGAAAATGTTTATGCTTTTGGTGTGCAAGGTGAAGCCCGGAAGAATACTTCACTGGGCTATGATTAAAAGTTCATGGGAAATCAACCAGCC
GACTACATGTCTGGAAGCACCAGGTTTATTTATTAAACCTGAACATAGCCATCTGGTGAAACTGGTATGCAGTGGGGAACTTGAAGCCGGAGTCGCAACAGGGACATCAGATGTTGAATG
TCTTCTAAGGAAGACAACCGTGTTGAGGAAGAATGTAACAGAGGTGGATTACTTGTATTTGGCATTTTATTGTAGTGCTGGAGTTAGTATTAACTACCAGAACAGAATTACATATCATGT
ATGATATGTTTATGTAAACATAAACCATTGTATGGAATAATGACCAAATAACATACAACACGCTATGAAATACAACACGCTATGACAAACAGGGAAAAATGAAGAATCGGGGGTTGATTG
GTCTATCGTATCGCTTAAGGGCCGCAGGCCCGTTGAAATGATTCTTTATAAAACAAATATACATGATACGGATTGTTGAAAATATAATCAACGATGTATAAATACAACAGAATGATGTAT
ACTAATTAAAATAATGAGAAGAAAAGAATATTTGTGAAGGATAAGCATCAGAACCACCACTTTAGTGGTGGGTCATATGTCCCGAGTTAGTGCGCCACGTAAGCGCTGGGGCTTAT

DNA-U3

36-43 SNPs to MF039881 MF039875 KY427065 MF039869 (**Thailand**)

U3 protein (39 aa): 0 aa to KY427065 (**Thailand)**

>U3_alyu37
TATTACCCCCAGCCCCCAGGACGGGACATGGGCTTTTTAAATGGGCCGAGAGAGTTTGAACAGTTCAGTATCTTCGTTATTGGGCCATCTGGCCCAATAATTAAGAGAACGTGTTCAAAA
TCGGGGTTTGACCGAAGGTCAAGGTAGACGGTCAACAATATTCTGGCTTGCGAAGCAAGCAACACGAATTAAATATTTAATTCGTATGACACGTGGACGGACCGAAATACTCCTGTATCT
CTATAAATAGCTTAAATCTGGCTTGGATAATTGCTCTCGCTCTTCTGTCAAAGCTGTTGTGTTGAGGCGGAAGATCGCCATCGGCGATCATCGGACGAAAAGCTGCAAGAGAGACGGAGA
ACCATGCTACGAAGCGTATATCGGGTATTTATAGACTTCTAGCGCAGCTTGAAGTTTCCTTGTACTTGATATTGTATTTTGTAAATTACGAAGAAATTCGTATATTGATAATAATAAAAC
ATCTGGGATTGTTAATGTTTACATTAACCAGTATTAATAATGTACAATAACAAATATTGTATGAGGAACGAATACAAGAATATTAAAATATGAGGAGCGTAGCGTGATAAACAGGTGTTT
AAGGTATAATTAAATTAATTATGCAATGTCATGATAATACGGTATAAGTTGAAGTATGAGGTGAAAGAGGAGATATTAGAATATTAAAAACCCAATTATATTATTTATAATACATACAAC
ACGCTATGACAAACGGGGAAAAAAGAAAAATCGGGGGTTGATTGGTCTATCCTAACGATTAAGGGCCGCAGGCCCGTCAAGATGGATGAACGGTTAGATTTGATTGCTTAGCCACGAAGA
AAGGAATCGTTTTGGGACCACAGACAAGACAGCTGTCATTACTTTTAAAAAATAATATAATAACCAATTGACGATAGTACCCCTCCCAAAGATGACGTATAGGGGTGTCCCGAGGTAATT
TAACATAGCTCTGACAAGAGATAGTGGACGTTGGATGGCACGATCGGACGATCGTGGTTTGATGAACCAATGATGACGTAGGGGCTGGGGCTTAT

ALYU-38 – Bidens pilosa - Vietnam

Neighbour to Musa ALYU-37 (Musa ABB infected with BBTV + one satellite BBTA2)

Virome

1. **BBTV** six components – identical to BBTV components in alyu37 (the Musa neighbour)
2. **BBTA5** alyu38=40 – identical to BBTA5_alyu40 (Musa ALYU-40 at another location)
3. **BBTA6** alyu38=26=27 - identical to BBTA6_alyu26 (Musa ALYU-26 at another location) and its Commelina neighbour alyu27
4. **BBTA2** alyu38=37 – identical to Babu2_alyu37 in the Musa neighbour
5. **BBTA3** alyu38=26=27 – identical to BBTA2_alyu26 (Musa ALYU-26 at another location) and its Commelina neighbour alyu27
6. **Bidens microvirus** – a new Rep-encoding ssDNA virus, 5051 nt circle
7. **Bidens circovirus** – a new Rep-encoding ssDNA virus, 2164 nt circle

BBTA5 = alyu40 (Musa)

>BBTA5_alyu38
TATTACCCGACCTTGGCTCACTGGCTCACTATAAAAGGGGGAAATGTCGCAGCAGCGAAATTGGGTATTCACGCGTAACTTCTCCGGCGACCCGCCGATTTTAAAGTTCGCCGGAGAAAC
TCAATACGCGTGCTGGCAACATGAGAAGAAGAATCACGACCATCTTCAGGGAGTGATTCAATTGAAGAAGAAGCTCCGGATGAACGCAGTGAAGACGTTAATTGGGGGAAATCCACACCT
CGAAGCCATGAGAGGAACAATCGACGAAGCAATAAGGTACGTGACGAAAGAAGAAACACGAGTAGCAGGTCCGTGGGAATTCGGAGAATTACTACGAAAAGGATCTCATAAGAGGAAATT
GATGGAATTGCTCGACGATCCAGATAACGAAATAATGGAACCTCAAAAATATCGACGCGCCATTACTAAACAAGCAATGGACGCGTCAAAGAAGAAGGCGGAATTAGGGTTTCCTTACGA
CTTAAAGGAGTGGCAAAAGATGGTTATTGAGTTAATCGAAGAACAACCAGACAACAGAACTATTATCTGGGTCTATGGACCTAATGGTGGAGAAGGTAAAACGCAGTTCGCAAAGCATTA
TGGATTACTCAAAGGATGGACATATCTACCAGGTGGTGAACTGAAAGACATGATGTATCTGTTGGCAAAAGAAATTAATAATAACGTTATTATTGATTTCCCCCGATGTACAAAGGATTT
CATATCCTATAAGTTTATTGAGATGGTAAAGAACCGATGCATATTTAGCTATAAATATGAACCAATCGGGGCGATTGTAAGTAATGAAGTCCATGTAATTGTAATGTCGAATGAGCTTCC
AGATTATTCAAAAATTTCGGAAGACAGAATAAAATTAATATTTACATAACGCACACTATGACAAAAGGGCAAAAATGCAAAATCGGGGGTTGATTTGTCTATATTTACGAATAAGGGCCG
CAGGCCCGTACACATTGCGCGGGAAATTCAAATTCGGACGCCAAATATTATTAAATACAATACACCGCCTTTTAGAAATCCGTGGACGAGAAGATGCCACGTAGCAAAACACTGTACTCC
TTGGCTCACTATAAATACCTGAGCCAAGGTCGGGCATAG

BBTA6 = alyu26 (Musa) = alyu27 (Commelina)

>BBTA6_alyu38
TATTACCCGACTTTGGCGCAGGGCGCACTATAAAAGATGTCTGTGAGAAATTGGGTCTTCACACGCAATTTCGAAGGCGAAAGACCAGAGCTCCTGTTCGGCGGCGAAACTCAATACGCC
GCTTGGCAACATGAAAGAAAGAATCACGACCACCTACAGGGTGTGATTCAGTTGAAGAAGAAGACCCGACTGAACGCAGTGAAGAGCATAATCGGTGGAAATCCACATGTCGAACCCATG
CGAGCGTCTTTCGACGATGCCCTAGCATACGTCATGAAAGACGAAACCCGAATCGATGGTCCATGGGAATTCGGTATTCGAATCCGTCAGGGTTCGAATAAACGAAAGCTGTTAGAAATA
CTGGAGGATTCGGACAACGAAGTCCTTCAACCCCAAAAATACAGACGAGCCATGGCCAAACAGGCCATGAGTGAGTCTAAGAAGAAAGCTGCAGAAGAAGGATTTAGTTATGAACTGCGA
GAGTGGCAAGTGCAATTGACGAAGTTGTTGGAAGAAGAACCCGATAACAGAACAATAATTTGGGTATATGGACCAAAGGGTGGAGAAGGTAAAACAGAGTTCGCTAAACATTTAGGGTTA
AAGGATGGTTGGACATATTTGCCCGGTGGTGAACTCAAAGATATGATGTACCTGTTATCAAAGGACATGAAAAATAATGTTGTAATTGATTTCCCCCGTTGTACCAAGGAATTTATTTCA
TATAAATTCCTTGAGATGGTTAAGAATAGAACTATATATAGTTATAAGTATGAACCAGTTGGTTCAATCGTATGTAATAAGGTTCATGTAGTGGTCTTCAGTAATGAGGAGCCAGATTAT
GTAAAATTATCAGTGGATCGAATAAAGTTGATTTCTTGCTGAACACGCTATGAAATACAACACACTATGAAATACTGTACGGGTATCTGTATGGTTTATCTTAACGCTATTGGGCCGAAG
GCCCAAGCCCAAGCCCATGCCCCCAAAATGAACGGTCAGAATTGATTGCTTCGCCACGAAGCAAACAAAAAATGCGTGGTGGAGAATTCGCCACGTCATTGGGTACTGTTATCCTTGGCG
CACTATATATAGGTGCGCCAAAGTCGGCCATAG

BBTA2 = alyu37 (Musa neighbour)

>BBTA2_alyu38_v1
TATTACCCACCTCCGCGCACTACCTCCGCGCACCTATAAAATGTCTGCCTCTCGATGGACATTTACGCTTCACTATTCCGACGCAACGGAGCGAGGCAAATTCCTCGCGACTTTGAAGGA
GGAAGATGTGCACTACGCCGTCGTCGGCGACGAAACTGCTCCGAATACTGGTCGAAAACATCTTCAAGGATATCTTTCCTTGAAGAAACGTTTTCGTATTAGCGGAATAAAGAAGAAATA
TTCGTCGAGAGCGCATTGGGAGAAAGCTCGAGGATCAGACTACGACAACAAGGCGTACTGTTCCAAAGAAGCCCTAATTCTTGAATTAGGGGTTCCTTGCCAAACAGGTTCGAATAAGCG
TAAATTAGCAGATATGGTTACAAGATCGCCGGAACGAATGAAAATTGAACAGCCAGAGATATTTCACCGATACGAATCGGTGAAGAAGATGAAAGAATTCAAAGAAAGGTATGTCTATCC
TATCCTCGATAGGCCATGGCAGGTACAATTAACGGAGTTAATTGAAGCAGAACCTGATGATCGAACGATCATCTGGGTATTCGGACCAAAAGGGAATGAAGGCAAATCAACGTATGCGAA
GTCATTAATCCAAAAGGATTGGTTCTACACAAGGGGAGGAAAGAAGGAGAACATATTGTTCGCCTACGTAGATGAAGGTTCGACCAAAAACGTTGTATTTGATCTTCCGCGTACAGTACA
AGAATTTATTAATTATGATGTTATCGAAGCATTGAAGGATAGAGTAATCGAGAGTACAAAATACAAGCCTGTGAAGTATTTAGAATTGAATACTGTACATGTACTAGTTATGGCTAATTT
TCTTCC**T**GATATGTGTAAAATATCTGAAGATCGAATAAAAATAGTTGCTTGCTGAACACGCTATGAAATACAACACGCTATGACAAACGGGGAAAAATGAAGAATCGGGGGTTGATTGGG
CTATCCTAACGATTAAGGGCCGCAGGCCCGTCAAGATGGATCCAATAACCCGTTAAGAAGTTAAACGGGTCTAAAACGATAACTTCGCCCGCAAGCAACACCTTTAACCTCTGCGCACCT
ATATATAGCGGAGGTGGGCTAG

C/T (7275/8316)

>BBTA2_alyu38_v2
TATTACCCACCTCCGCGCACTACCTCCGCGCACCTATAAAATGTCTGCCTCTCGATGGACATTTACGCTTCACTATTCCGACGCAACGGAGCGAGGCAAATTCCTCGCGACTTTGAAGGA
GGAAGATGTGCACTACGCCGTCGTCGGCGACGAAACTGCTCCGAATACTGGTCGAAAACATCTTCAAGGATATCTTTCCTTGAAGAAACGTTTTCGTATTAGCGGAATAAAGAAGAAATA
TTCGTCGAGAGCGCATTGGGAGAAAGCTCGAGGATCAGACTACGACAACAAGGCGTACTGTTCCAAAGAAGCCCTAATTCTTGAATTAGGGGTTCCTTGCCAAACAGGTTCGAATAAGCG
TAAATTAGCAGATATGGTTACAAGATCGCCGGAACGAATGAAAATTGAACAGCCAGAGATATTTCACCGATACGAATCGGTGAAGAAGATGAAAGAATTCAAAGAAAGGTATGTCTATCC
TATCCTCGATAGGCCATGGCAGGTACAATTAACGGAGTTAATTGAAGCAGAACCTGATGATCGAACGATCATCTGGGTATTCGGACCAAAAGGGAATGAAGGCAAATCAACGTATGCGAA
GTCATTAATCCAAAAGGATTGGTTCTACACAAGGGGAGGAAAGAAGGAGAACATATTGTTCGCCTACGTAGATGAAGGTTCGACCAAAAACGTTGTATTTGATCTTCCGCGTACAGTACA
AGAATTTATTAATTATGATGTTATCGAAGCATTGAAGGATAGAGTAATCGAGAGTACAAAATACAAGCCTGTGAAGTATTTAGAATTGAATACTGTACATGTACTAGTTATGGCTAATTT
TCTTCCCGATATGTGTAAAATATCTGAAGATCGAATAAAAATAGTTGCTTGCTGAACACGCTATGAAATACAACACGCTATGACAAACGGGGAAAAATGAAGAATCGGGGGTTGATTGGG
CTATCCTAACGATTAAGGGCCGCAGGCCCGTCAAGATGGATCCAATAACCCGTTAAGAAGTTAAACGGGTCTAAAACGATAACTTCGCCCGCAAGCAACACCTTTAACCTCTGCGCACCT
ATATATAGCGGAGGTGGGCTAG

BBTA3 = alyu26 (Musa) = alyu27 (Commelina)

>BBTA3_alyu38
TATTACCCGCTTCCTCGCCCCTCTTCCTCGCCCCAGTGCAAAGCACGTGATGTCTTCCTTTAAATGGTGCTTCACGCTGAATTACTCCAACGCAACAGAGAGAGAAGACTTTCTCTCTCT
TTTGAAGGAGGAGGACTTGCGCTACGCAGTAGTGGGAGACGAAGTCGCCCCGTCAACTGGTCAGAAGCACCTACAGGGATATATATCCCTAAGGAAGAGTATGAAACTGGGTGGTTTGAA
GAAAAAATACTCGGCGAAGGCACACTGGGAGAAGGCGAAAGGATCAGATGAAGATAATACAAAGTATTGTTCCAAGGAAACCCTAATTCTTGAATTAGGGGTTCCTACATCCCAGGGTTC
AAACAAGCGTAAGCTTTCAGAGATGGTTACCAGATCACCGGAACGCATGAGGATAGAACAGCCAGAGATATATCACAGATATCAATCTGTGAATAAGTTAAAACTATTCAAAGAGGAATT
CGTACATCCTTGCCTCGATAGACCATGGCAGATACAATTGACGGAGGCAATTGAAGAGGAACCAGATGATCGAAGTATCATCTGGGTCTATGGACCGAATGGCAATGAGGGGAAATCAAC
ATATGCGAAGTCTCTGATAAAACATGATTGGTTCTATACAAGGGGAGGCAAGAAGGAGAACATACTGTTCTCCTACGTAGACGAAGGGTCTACGAAGCATGTTGTGTTCGACATACCAAG
ATGTCATCAAGAATATCTAAATTATGATGTAATCGAGGCATTGAAGGATAGGGTGATCGAGAGCACTAAGTATAAGCCGATTAAAATAATCGAGTTGAATAGGGTACATGTAATTGTAAT
GGCTAACTTCATGCCAGAGTACTGTAAGATCTCTGAGGATCGAATAAAATTAATATTTGCATAGCGCACGCTATGACAAAAGGGCAAAAATGCAAAATCGGGGGTTGATTGGGCTATCCT
AACGAATAAGGGCCGCAGGCCCGTCAAGATGGATCCGTTAACCCGGTATCAGATTACTTCGGGACGAAGGAATCACTTTAAGTTCCTCGCCCCTATATATAGTTTTGCCCGAGGAAGCGG
CCTAG

DNA-C = alyu37

>C_alyu38
TATTACCCCCCCTGCTCGGGACGGGACATCACGTGCATCAAACAAATGCACGTGACAAAGAGGTAGCTTGTAGCGAAAGATAACCATCACTATCACAAAAGTGAGAGGAATATTCGTTGC
TTAGGGACTAAGCAACGAGGACAGATATATGTTCGAGAAGCGACAATGGAGGCTATTTAAACCTGATGGTTTTGTGATTTCCGAAATCACTCATCGGAGCAGAAATGGAGTTTTGGGAAT
CGTCTGCCATGCCAGACGATGTCAAGAGAGTGATCAAGGAAATATATTGGGAGCATCGAAATAAACTTCTGTTCTGTCAGAAGTTGAAAGGCTGTGTAAGAAGGATTCTGGAGCATGGAA
ATCAAGATGATGCTCTAGACGCAGTGAAGGAGATGAAGACTTCTATTATTCGCTATAGCGAATATTTGAAGAAACCTTGTGTGGTATTGTGTTTTAGTACTAATAAATCTATTGCTTATA
GGTTAAACACAATGGTGTTCTTTTATCATGAATATATTGAAGACCTAGGTGGTGACTACTCGGTATATCAAGATCTGTTTTGTGATGAGGAACTTCCTTATTCGTCGACAGAAGAAGAAG
ATGAAGAAGTAATATACAGGAATGTTATCATGGCATCGACAGAAGAGAAGATCTCTTGGAGTGAATGTCAAAAGATAGTTATATCAGATTATGATGTAACATTACTGTAATGAATATCCA
TTATCATAAATAAAATAATGGAATGATGATTATGTATTCTAGTATAAATACATAATGGTATACGTATAGCATAAAATACATAAACCAACATACAACACGCTATGACAAACGGGGAAAAAT
GAATAATCGGGGGTTGATTGGGCTATCTTAACGATTAAGGGCCGAAGGCCCGTTTAAATATGTGGTGGACGAAGTCCACAACACAAAAAAAGTGATCAGAACAATGGAATATAATGAGCT
GGCAACGTAGGGACCATGTCCCGAGTTAGTGCGCCACGTGAGCAGGGGGGACTAT

DNA-M = alyu37

>M_alyu38
TATTACCCCCAGCGCTCAGGACGGGACATCACGTGCAACTAACATATGCACGTGACTGATATATACTACATAACGGTTTAATGAACCGTTATATAATGTTATAACGAAAAGTCACGTGTG
ATAGAGACCTGCACGTGACATAGTCAAATGTATTGAATAAACATTTGACGTCCGGATGCTTCCGACGGAAGCTTATGTTACTTCGTGGCGAAGCAAAACATTTATATATTGCCCTGGACT
GCTGCCTATAAATAGGCAAGCAAGGAAATGGCATTAACAACAGAGCGAGTGAAACTATTCTTTGAATGGTTTCTGTTCATCGGTGCAATATTTATTGCGATAACAATATTATATATATTG
TTGGCGTTGCTCTTTGAGGTCCCCAAGTATATTAAGGATGTTGTGAGGTATCTCGTAGAATACCTGACCAGAAGACGTGTATGGATGCAGAGAACGCAGTTTACGGAGGCAACAGGAGAT
GTAGAGCTCGGCAGAGGTAGTGTGGAAGACAGACGGGATCAACAACCGGCTGTCATACCACATGCAACACAGGTTATCCCTTCGCATCAACCTAGAAGGGATGAGCAAGGAAGACGAGGA
AACGCCGGACCAATGTTTTAATACACGGTATTGTAATATACGAAATATAAATTGGTAATGATATCTATTGTGAAACATAATATACGTGAAACATAATATATGTTTGATAATTTACATATT
GTAATATGTAATATGTATACGAGTGTTGTATTTATAGAACATACAACACGCTATGACAAACAGGGAAAAATGAAGAATCGGGGGTTGATTGGTCTATCGTATCGCTTAAGGGCCGCAGGC
CCGTTGAAATGATTCTTAATAAAACAAATATACATGATACGGATAGTTGAATATATAAACAACGAAGTATAAATACAACAAAATGTTGTATACTAATTAAAATAATGAGAAGATAAGTAT
ATTTGTGAGGGATGATGATCACAACCACCACTTTAGTGGTGGGTCATATGTCCCGAGTTAGTGCGCCACGTAAGCGCTGGGGCTTAT

DNA-N = alyu37

>N_alyu38
TATTACCCCCCGTGCTTGGGACGGGACATGACGTCAGCATAGATTATAATGGGCCAACTAAAGGCCCATTTAATAGAATAGGCGGGCTTTTGACATATTTCAAAGGCCCAGCCTGGAAGT
GGATAATGTCACGTGCCTAATAAGAGGTTGCTTCGCCTCGAAGCAATCCGAATAAATGTTGCGTATTCAATACGCAACTGAAGTCTATTAATATGGATGTCTCTGCCGAATAAATCAGAG
CGTAAGCGAAGCAGAAGCGATGGATTGGGCAGAATCACAATTCAAGACATGTACTCATGGGTGCGATTGGAAGACGATATCATCGGATTCATCCGAGAATCGACAATATGTACCTTGCGT
CGACGCTGGCAGCGGAAGAAAGTCACCTCGCAAGGTACTGCTAAGATCTATTGAAGCTGTGTTTAATGGAAGCTTCAAAGGAAATAACAGGAATGTTCGTGGGTTTATATACGTATCAAT
ACGAGACGATGATGGAGAAATGCGTCCAGTACTCATAATACCATTCGGAGGATATGGATATCATAATGATTTCTATTATTTCGAAGGGAAGGGGAAAGTTGAATGTGATATATCATCAGA
TTATGTAGCGCCAGGAGTCGATTGGAGCAGAGACATGGAAGTTAGTATTAGTAACAGCAACAACTGTAATGAATTATGTGATCTGAAGTGTTATGTTGTTTGTTCGTTAAGAATAAAGGA
ATAACAGATGTGCTGTAATGATTATTAATAAATCTTATTTTCATGTAATTGATAGTTGTATAAAACATACAACACGCTATGACAAACAGGGAAAAATGAAAAATCAGGGGTTGATTGGTC
TATCGTATCGCTTAAGGGCCGCAGGCCCGTTGAAAAATAATAATCGAATTATAAACGTTTGATAATAATCAGAGATAGATGATAAGCATCCAAAAACATAGACGAAGTATATGGATGTAT
AATATAAAACAAGCATATAAATAAAATATGTGAACTAATCTCTGATTGGTGCAGAACGTAGCCCCACTAACTTTAAGTTAGTGGAAATGTCCCGATGACGTAAGCACGGGGGACTAT

DNA-R = alyu37

>R_alyu38
TATTACCCCCAGCGCTCGGGACGGGACATTTGCATCTATAAATAGACCTCCCCCCTCTCCACTACAAGATCATCATCGTCGACAGAAATGGCGCGATATGTGGTATGCTGGATGTTCACC
ATCAACAATCCCGCTTCACTACCAGTGATGCGGGATGAGTTCAAATATATGGTATATCAAGTGGAGAGGGGACAGGAGGGTACTCGTCATGTGCAGGGATACGTCGAGATGAAGAGACGA
AGTTCTCTGAAGCAGATGAGAGGCTTCTTCCCAGGCGCACACCTTGAGAAACGAAAGGGAAGCCAAGAAGAAGCACGGGCATACTGTATGAAGGAAGACACAAGAATCGAAGGTCCCTTC
GAGTTTGGTGCCTTTAAATTGTCATGTAATGATAATTTATTTGATGTCATACAGGATATGCGTGAAACGCACAAACGGCCTTTGGAGTATTTATATGAGTGTCCAAATACCTTCGATAGA
AGTAAGGATACATTATACAGAGTTCAAGCAGAGTTGAATAAAACGAAGGCGATGAATAGCTGGAAGACATCCTTCAGTGCATGGACATCGGAAGTTGAAAATATTATGGCGGAGCCATGT
CATCGAAGGATAATTTGGGTCTATGGCCCAAATGGAGGAGAAGGAAAGACAACCTATGCAAAGTATTTAATGAAGACGAAGAATGCGTTTTATTCTCCAGGAGGAAAATCATTGGATATA
TGTAGATTGTATAATTACGAGGAAATAGTTATATTTGATATTCCCAGATGCAAAGAGGAATATTTAAACTATGGTTTATTAGAAGAATTTAAAAATGGAATTATTCAAAGCGGAAATAT
GAACCCGTTTTGAAAATTGTAGAATATGTCGAAGTCATTGTAATGGCTAACTTCCTTCCGAAGGAAGGAATCTTTTCTGAAGATCGAATAAAGCTAGTTGCTTGCTGAACACGCTATGAC

AATCGTACGCTATGACAAAAGGGGAAAAGCAAAGATTCGGGGGTTGATTGGGCTATCCTAACGATTAAGGGCCGCAGGCCCGTCAAGATGGACGGCTTGATCAGATGTCCCGAGTTAGTG
CGCCACGTAAGCGCTGGGGCTTAT

DNA-S = alyu37

>S_alyu38
TATTACCCCCAGCGCTCGGGACGGGACATGGGCTAATGGATCATGGAATAAGGGCCCATCGGGCCCGTTAAGATGGGTTTTGGGCTTATGGGCTTTATCCAGAAGACCAAAAACAGGCGG
GAACCGTCCCAATTTCAAATTTAGATAGCTTGCCCCGCAAGCAAACTAAAAGTCTATATATACCAGTGTAGACATATTGTTCAGAGAACAAATGGCGAGGTTTCCGAAGAAATCGATCAA
GAAGAGGCGGGTTGGGCGACGGAAGTATGCAAGCAAGGCGGCAACTAGTCACGACTACTCGTCGTTAGGTTCAATATTGGTTCCTGAAAATACCGTCAAGGTATTTAGGATTGAGCCTAC
TGATAAAACATTACCCAGATATTTTATCTGGAAAATGTTTATGCTTTTGGTGTGCAAGGTGAAGCCCGGAAGAATACTTCACTGGGCTATGATTAAAAGTTCATGGGAAATCAACCAGCC
GACTACATGTCTGGAAGCACCAGGTTTATTTATTAAACCTGAACATAGCCATCTGGTGAAACTGGTATGCAGTGGGGAACTTGAAGCCGGAGTCGCAACAGGGACATCAGATGTTGAATG
TCTTCTAAGGAAGACAACCGTGTTGAGGAAGAATGTAACAGAGGTGGATTACTTGTATTTGGCATTTTATTGTAGTGCTGGAGTTAGTATTAACTACCAGAACAGAATTACATATCATGT
ATGATATGTTTATGTAAACATAAACCATTGTATGGAATAATGACCAAATAACATACAACACGCTATGAAATACAACACGCTATGACAAACAGGGAAAAATGAAGAATCGGGGGTTGATTG
GTCTATCGTATCGCTTAAGGGCCGCAGGCCCGTTGAAATGATTCTTTATAAAACAAATATACATGATACGGATTGTTGAAAATATAATCAACGATGTATAAATACAACAGAATGATGTAT
ACTAATTAAAATAATGAGAAGAAAAGAATATTTGTGAAGGATAAGCATCAGAACCACCACTTTAGTGGTGGGTCATATGTCCCGAGTTAGTGCGCCACGTAAGCGCTGGGGCTTAT

DNA-U3 = alyu37

>U3_alyu38
TATTACCCCCAGCCCCCAGGACGGGACATGGGCTTTTTAAATGGGCCGAGAGAGTTTGAACAGTTCAGTATCTTCGTTATTGGGCCATCTGGCCCAATAATTAAGAGAACGTGTTCAAAA
TCGGGGTTTGACCGAAGGTCAAGGTAGACGGTCAACAATATTCTGGCTTGCGAAGCAAGCAACACGAATTAAATATTTAATTCGTATGACACGTGGACGGACCGAAATACTCCTGTATCT
CTATAAATAGCTTAAATCTGGCTTGGATAATTGCTCTCGCTCTTCTGTCAAAGCTGTTGTGTTGAGGCGGAAGATCGCCATCGGCGATCATCGGACGAAAAGCTGCAAGAGAGACGGAGA
ACCATGCTACGAAGCGTATATCGGGTATTTATAGACTTCTAGCGCAGCTTGAAGTTTCCTTGTACTTGATATTGTATTTTGTAAATTACGAAGAAATTCGTATATTGATAATAATAAAAC
ATCTGGGATTGTTAATGTTTACATTAACCAGTATTAATAATGTACAATAACAAATATTGTATGAGGAACGAATACAAGAATATTAAAATATGAGGAGCGTAGCGTGATAAACAGGTGTTT
AAGGTATAATTAAATTAATTATGCAATGTCATGATAATACGGTATAAGTTGAAGTATGAGGTGAAAGAGGAGATATTAGAATATTAAAAACCCAATTATATTATTTATAATACATACAAC
ACGCTATGACAAACGGGGAAAAAAGAAAAATCGGGGGTTGATTGGTCTATCCTAACGATTAAGGGCCGCAGGCCCGTCAAGATGGATGAACGGTTAGATTTGATTGCTTAGCCACGAAGA
AAGGAATCGTTTTGGGACCACAGACAAGACAGCTGTCATTACTTTTAAAAAATAATATAATAACCAATTGACGATAGTACCCCTCCCAAAGATGACGTATAGGGGTGTCCCGAGGTAATT
TAACATAGCTCTGACAAGAGATAGTGGACGTTGGATGGCACGATCGGACGATCGTGGTTTGATGAACCAATGATGACGTAGGGGCTGGGGCTTAT

Bidens microvirus

Major capsid protein most related to:
QCS37406 Tortoise microvirus 100 source: Turtle Gopherus morafkai feces 100% coverage 45% identity, 59% positives; 13% gaps; AXH74625 Microviridae sp. source: Macaque stool 67% coverage, 64% identity, 78% positives; 13% gaps
DNA pilot protein most related to:
QCS37408 Tortoise microvirus 100 source: Turtle Gopherus morafkai feces 49% coverage, 35% identity, 50% positives; AXH73738 Microviridae sp. source: Macaque stool 86% coverage, 43% identity, 55% positives
Replication initiator protein most related to:
AXH74235 Microviridae sp. source: Mouse vaginal tissue 86% coverage, 40% identity, 55% positives, 11% gaps; AXH74625 Microviridae sp. source: Mouse vaginal tissue 98% coverage, 37% identity, 50% positives, 12% gaps; QCS37407 Tortoise microvirus 100 source: Turtle Gopherus morafkai feces 78% coverage, 38% identity, 55% positives, 12% gaps

>Bidens_microvirus_alyu38
ATGAAAAAAAGCAAATTCAAACTAACACACCTACACAGCACAACGCTTGACCCCGGCTATCTCGTTCCTTTCTGCTTAATCCCTACGCTCCCAAACGACTCTTTTAGGCTTGGTCTCTCA
ACATTCATTCGAGCCCAGCCAATGCTCGCCCCCTTGATGCATGAAGTTCGTCTCTTTACGCAATACTGGTTTGTACCGAATCGTATTATCTGGGAAGAATGGGAAGACTTCATCACCGGT
GGTAATGACCTCTCGCTAACGCCCCCGTTTCCTACAGTCAAACTCACAGCTGACGATTGCAAGGTAGGTTCTCTCACCGACTATTTTGGTTTTCCGCTGGTTCAAGATTTGGAGGTCTCA
GCTCTACCATATCGCGCTATGGCTGAAATCTGGAATACACGCTATCGCGATGAGGATTTGCAAGATGAGCTACCCATAGATTACGGCAGTAGTGAAGACACAACGACATCTCGTAAGCTT
CTCTCTCCAAGTTTCGCCAAAGACTACTTCACGACTGCTAGACCTTTTACGCAACGAGGCGGTGACGTGGTTGTTCCTGTTGTTTCAGCTCCTAATACTGGCTCCAATACTGGTAAAAAG
GTTTCCTTTTTTGCTATTATCGGAACAGCTACAAAATCTACTACTGGTGGTTCTGGAGCACTTGTTCAGGCTGGACAAAATTTTAGCTCTTCATCTGTTCTTGCTATTATTCAAAATTGG
ATTACTGCACGTATTGAAGATGGAACTATAGACCAACTTATTGTTTCACCTGATTTTAGGATTGCCATATGGAGTTCGAATTCGACTTCTATTGAGACTAATAATTCAACCCCTGACGAT
TGGTCTTCTCACCTTGCAGGGAGGATTGGTTCTAGCCAGTATGCAACTGGTAATAGTGCTGACTTTAACGTTTGGGTTTTTCCTTTGAGTGTTGAAAAATATGAACAACCTGACGTCTTT
GCTAGTAAGGTCGATTTTCCGATTTCGCCCAATTTTACTGCCCCGGGTGTTTGGTTCGCTCCATCTAATGTCGGCTATGGCGCTTTTGGCACTGGTGAAGGGACTTATTATCGCTTTGTT
GACCCTCAAGACATGACAGTCTTTGACGCACCTATTAACGTCACACGTCCTTTTTCACAGCTTAGAGATAAGAGTATTGGTACTGCTATTATTGCTGAAACTACTTCTGTCTCTGGTTTT
TCAATTCGAGACCTTCGTAGGTCAGCGGCTTTACAACGCTACGCAGAGAGGAGTTTGAAATATGGAAATCGATATGAGGAATTCATTCAGCGAGAATTTGGTATTAAGCCTCGAGACTAT
CGAATTCAGCGCCCCGAGTATCTTGGAGGTGGTAGTGGTATACTCAATATCTCTGAAGTCTTGCAAACTGCAGAATCAACTGATACGGGAGTTGGCACTATGCGTGGTCATGGCGTTGCT
TCGATATCTCAAAGACCAATTAGGTTTCGTTGTCCAGAGCACGGCATCATACTTGGACTTCTCTCAATTAGACCAAAATCGGTTTATACTCAAGGCGTCGATAGAGAATGGCTCAAACGC
TCAAGACTTGACTTCTTCACCCCAGAGCTTGCTAATATCGGCATGCAAGAAGTTCTTCAACAAGAGCTATTCGCTACCGCAGATAATGCAAATGAAGTTTTCGGATACAGTGAGCGGTAC
CAAGAATATCGCTATCAGAAGCCAGCAGTCACAGGTGAATTCAGAGGTACTTTGAATTTTTGGAATATGGCTCGTTTGTTTGCTAATTCTCCAAATTTGAATGCTGAGTTTATTAATATG
GGTAATGCTGTTGATGACTTCAAGCGTCCATTCCAAATTCAAGACAATTCGGCTCACGCTTTTGTATGTATGCTTAAAAATCATATTCGAGCATATAGGCCGATTCCAAAACGCGCAAAA
GATATTCTCAAGTAAGGATTAACAATGAAAAACAAGAATCGATTCAGAGAGCCGCTCGAGCTTAAGGAGGCTTAAGGATGAGTAGAATTGGCAATCTGATTAAAAAGGCACAAAGTTACA
AAGGTCAAGAATTTAAGCAACCTAGGATGTTTGTTCCCTCGGGAATGATTGTTCCTGAAACTGCTAAAAAtTCTATGGCTCGACAATTGTATCAAGCAGGTATCATCACAAAAGATGATT
ATGAGAAAATGCTTGGTGTGGTTTATGATATTAATGATGTTTCCCAAGAAAGGTTTGATGATGAGACATTTTCAGCACTTCACGAAGAGTTCGAACTCTCGAAGCTCTCGGAATACTACG
ATGACTACGGAGATGAAGAATCCCCCAGTGACACCAAAGGAACAGAAGTTGTTCAGTCAGGCTCTGGAACAGCAACAAACGAAGTTGTTCAAGCAGCTAGTACCGATACACCCTCAGAAG
CAAACGATGAGCCGGGCTTGCATAATTGAGTTTGAAGGCACAATGTGAGCAAGAAATCTAGCTCTAAAGGGCATTCTAGGGGCGTTCAGCCCCGAATGCGCAACGCGCGCTTGCGCGCTC
TTCGGACTGCTATCGCACAGCGGAATAGAACTCACTTGTCTTTATTCCGCCAAATGACACACCCACGCAAATTACGCATTCTCACACCCTCGAAACGCGTAAGCCAACAAGATGTGTTTC
GAGGTTTTCTCAACACTCGTAATTTGCACATAGATTTACAGCCTACGGTGTGTCAAAAACGGCAACAGCGAAGACGCTCTCTATTCGCTTTAGGTTTGACTGGTAAGGGCGCTAGAAGCC
CTAAAAAACACACGCAAGAATCCAAAATAAGGTGTTCGTGATGATAGGTTCAATTATTGGTGGAGCAGCTCAGGCTGCTTCTGGTATTGCGGGTTTGATTGGTGCTCGCAAAGATAAAAA
GAATGCTGAAGCTGCTGCTGAGCGCTCTTGGCAACGCAATTATGAAGCTCAGAAAGAGTTTGCACAAAACAGCATACAATGGCGCGTTCAAGACGCTAAGAACGCTGGTATTAATCCCTA
TGCTGTAGTATCAGGTCAATCTGCAGGTTATACACCGCAAGACACGAGCTATCAGACTAACTATCAACAGGCAGCTAGTCAAATGATGAATGGCTTTTCTGACGCGATGGGGCAACTTAA
TCTCGCTTCTGCAGCTGAGGATTTGAAGGGCAAAAAGCTTGACAATGATGCAAAAGCTCTTGAGCTGGTCAACAAGACGATAGCTAACGAGCTAGGGCAGTTATCGGCAACTCTTAAGCA
ACCTAGTTTAACGCATACAAATCCAATTAAGGAAGTCGATGGTAAGAAGCTTATCACTGATGCTGACGGCTCTCAATGGTTCAAGTCGACATCGGATGATATGGATCCATGGAATATTCA
GGCTGTCAGAGACGTTTTTACAGCTCTTTATGCACCAGAGCAATACAAAGCGCTACAAAAAGCCAATAAAGGTAAAGGTGAGCAGGCGCTTTATGTACTTGGACGCGCGTATCATCCTAA
AAAAGGCACAGCTCCAGCTCCCAATGTTGAAGCTGCTAAGCTTGCCGATAGAGCTGGAACACTTGCAGGTTGGCTGAGTTTGCCTTCGCAGTGGCTCAAACAGGGCATATATTCTCTTGA
GCGTGCTTTTCGTAGAGGTCGCTAAATTATGAAAGGAGGTAGACCAAAATGGCATTTAGATGGTATCGAAGACGGCGAAGACGTGGTTTTAGAAGACGTGGTTTACGCGGTAGACGACGT
TTTTTGCGTGGGCGAAGACGTAGGCGATTTCGCAGACGTAGGCGTCTCAAGGTTATTGGTTACAGGATGTAATTTCTGTTAACAATTCATTAACTATTCTTAGTTATACTTCGTTTCGAA
GCCATAAGCAGCTTCTTGTTTTTTTGCGATTTCAGGGAGACTGCTGCTTCGCGCGAGGTTCACATGGTCTGTTTTTCACCCTTCTTTTCTGTTTCTGACAATTCACGTCTTGTAGGCTTC
CCCTGCGGCAAATGCCCCGTTTGCCTTCAAAAGCGAGCTAACGAATGGTCTCTGCGTATCTCTTTGGAATCCCGTATGTATGACGATTTGGCATTTGTTACGCTTACTTACTCACCCGAA
GCTTTGTATAGCAATGAGTTAATCCCTTCTCATCTTTCGAGCTTTATCAAACGCCTTCGCACTTATTCTGAGCGTCTCGGTTTCACTGGTAAGATTCGTTTCTATGGTGTTGGAGAGTAT
GGAGAAAAACGCGGTCGCCCCCACTTTCACGTTGTTTTGTTTGGACTTCCCCAAAGGTTTTGGTCTTGTATCAAAGACGCTTGGCGCGGTTCAATTGTCGATATTCAGAAGCCACGCACC
GTTGATGCTGTAGCAAGCTATGTCGCTGGTTATGTTGTTAAGAAGCTTTATACGACATCGAAGGTTATTATGCGTCAATCTCAGGGGCTTGGTAAAACATTTGTCGACAAGTTACAGCAT
TACACGCAGACAATCGTTATGAATGGCAAAACGCGCTACATTGGACGCTATTTGCGCAATAGACTAGCTAAACGACTTGGTGTCTTTGAGCATGTTAAAGAGCAAGGTATGCGTGCCCTC
GAACAAGCATTTTCTGTTCTCATCCCTTATCACATACCACTGCTCCTCAAGGAAAGCAGAGAATCTCTTGTTCAGGAATGGCAACGTATTCACAAAGGGCAAATAGACTATGTGCAATCT
CTGCACAAACTTAAATTTTTGAAAGGTTTCACATGAAACACGTTCTTCTTGCTACGGCAATTTTTGGTCTTGTTATTGGTTGCAGTCTTAGCTACAAGCCTGAAGTCAAAGACAGCGAAA
TTTATCATCAGTCTGGCAATAAGCCTGATACTACACTAAACATAAGGAGTTCAAAATGAGACCCGATTCAGAGAGCCGCTCGAGCTTAAAGAAATCTGACCCTATCGATGCTATCGTTGA
GTGCATTGACACGTTTGTTGAAGCTTCGTATGAAGATAGCGAGGTTACAGACGTTGAGTTACGCATAGCTAAGAAACGCTTTCGTTCTGCTTTACAATATATCATCATAACAACAGTGGA
GGATTACTTCT

Bidens circovirus

BlastN best hit: KM392285 Swine cyclovirus (3% coverage; 75% identity)

Rep protein best match to AUM61980 uncultured virus from wastewater 91% coverage, 41% identity, 57% positives, 9% gaps; YP_009551384 False black widow spider associated circular virus 1 92% coverage, 35% identity, 52% positives, 10% gaps

>Bidens circovirus_alyu38

TAGTATTACCCTTCAACCTCTGTACACCTCTGTACACCTATTTCTCATTTTTTATGAGTGCAACGAAACATTGGCTGTTTACGCTCAACAACTTCACCGTAGATGAATTCAACAACTTGC
GAAGCCCACCCGAATGGGTGTCTTATCTCGTGTTCCAACATGAGCGCGGCAATGAAGGAACTGATCATTTGCAAGGGTATCTCGAACTTGCTGGACGATATAGGATGTCCCGATTACGCG
ATTTCCTTCCACGCGCCCACTGGGAGCCACGACGTGGATCTCAAGCTCAAGCTATTGCTTACTGCTCCAAAGCCGACACTCGAGTCGACGGACCATGGACAGTGGGGATTTTTGAAGAAA
CCCGGGCTGGACAAAGGAAAGATCTGGACGAACTTGCTCTACGAGCCTACTCCGGCACCGAGACCCTCCATTCTCTGCAAGAGGCCTACCCTGGACACTGTCTCCGATACAGAAAGTCCA
TCAAAGAGGCACTGCTCGACCACAGACGAAAGCTTGCCAAAACCATCCAACGTGACCTGGAGGTCGTCATCCTATGGGGCGACCCCGGCACTGGTAAGACAAGGAGCGTATACGAATCTG
AGGGAATGTCCAATGTTTACACCCTTAACACCGCCACCAATGGAACCCTTTGGTTCGATGGATACGATGGGGAGTCCGTCCTACTTATCGACGATTTTCGAGGATGGATTCGATTCAATG
AGGTTCTAAAGATCTGCGACCGTTATCCCTACCGTCTCGCCGTCAAGGGTTCTTTTGATTATGCAAGCTGGACGAAAGTCTACTTCACATCTAATCACAATCCTGAAACGTGGTGGAGTG
AAGACTCTGGCCATTGCTTTAGCGCTTTTCGCAGACGTGTTGCTCGTGTGGAGCATTTTTCATCCGACCACCCATGGGTACCTTCCGTCGCCCACTCCGACGACGCCGCTCGTACCGCCG
ACTAACCAGACGGAACTCTTTTCGCCGTGCTACTAACCGTAAAAGACGCCTGTCTCGAGTCGTTAAAAACTACCGCCGCAAGAACATGACCAGCCTTATCACTAAAGGCACCTTCAACTG
GGAAACCAATGATGATTCACAGGCTCGCCTTGGAGTCAACCACAACGGTATTCCCAACGCTACCGACGTACAACATGGTGTTATCTGCATCGAAGATAACGCTGGCACCATGCACAACGT
ATCCTTTGCTGGAGTCACCGCTTCTACGCAGGCACTTTACGCTGCTAACAACGGACTCTTCCTCAATCGCCGTATTGCTTGGGTCAAGATGACCTTCTTCCCTGTGATTACCGAAATCCC
TGCTCTTATCGAGGGCACGACACTGAAATACCAGGCAAACCGCCCTGTCTTCATTTGTCACGATTCTAACGGCCAAATTACCTCCTTCACATCTATCACCGTACCCCAGATGCTCGTCAA
CCTTACGGGAGTCAAACAGAAGTCCATGCTGAAACAATTCAAGGTCTTCAGGAAGTCCCGAAAGTTCCCCGCATTTCAGAAGTATGCTGCGCTCCCCCAAGCTGGTACCGTAGACAACAT
GTCTACTCCATCGGGAGCCTGGCACTACGCCTCAGACCTCTCTATTCTGGGCTCTACATTCCGACATACATCAATGCTCTTGGATCTCGACGCCACTCACTGGGAGGGCGGAGATGTGTT
CACTGTTGTTTTTGAAATCAAATATCAATGGTGCGATAGACGTCAGTAAAATCTTTTAATGGAGTTTAACAACTTGATCAACCACTTTTTCTCGAATTCCCACGCTGGACCATCAGGGCA
ATTCCTCACTGCGATCTGCAGCTTCTTATAAACCCCCCACATCCCCACAAACTTGGGATACATCCATCCAGTCATGCGATTCACATCATAGCGCCGCATCAATTCTTCCCAAACTTCAGA
ATTAAACCTGTCTTGCCACCGCTTCTCCCATGCATCTTTCGCATCCCAGAACTCATCAACCTTGGACGGCTGCTTCTTATGGACCCAATAGCCCTTTGCGTCTTTCGTGTAACCCCTACT
CTCCATGTGTAAGAATAACAGAATACCCGACAAGATTCTGAGAAAATTCCCAGAATTCTGCCCATAATTATGGGCATGGGCAAAAAAACATCATATTTGCGCTCGCTGTACCAAGGTTGA
AGGC

Host plant contigs

Bidens aristosa voucher Steele 1224 **plastid sequence**
Sequence ID: KP126872.1
5x contigs 35, 36, 70, 73, 94, 98 (ca. 98-99% identity)

ALYU-39 - **Musa sp.** - **Vietnam**

Virome

1. **BBTV** six components
2. **BBTA6** alyu39
3. **BBTA2** alyu39

BBTA6
Best matches to AJ005966 AJ132187 82% coverage 66.9-66.5% identity *Faba bean necrotic yellows C9 alphasatellite isolates;* MF510475 MF510474 83% coverage 66.7% identity *Faba bean necrotic yellows virus associated alphasatellite 2*

Rep protein best matches to ARI50297 100% coverage 62% identities, 77% positives Sophora alopecuroides yellow stunt alphasatellite 3; P0CK61 100% coverage 60% identities 76% positives Faba bean necrotic yellows C9 alphasatellite; ATU31571 100% coverage 59% identities 77% positives Faba bean necrotic yellows virus associated alphasatellite 2

>BBTA6_alyu39
TATTACCCGACCTTGGCGCAGTGGCGCACTATAAAAGATGTCTCTGAGAAATTGGGTCTTCACACGCAATTTCGAAGGCGAAAGACCAGAGCTCCTGTTCGGCGGCGAAACTCAATACGC
CGCTTGGCAACATGAAAGAAAGAATCACGACCACCTACAGGGCGTGATTCAGTTGAAGAAGAAGACCCGACTGAACGCAGTGAAGAGCATAATCGGTGGAAATCCACATGTCGAGCCCAT
GCGAGCGTCTTTCGACGATGCCCTAGCATACGTCATGAAAGACGAAACCCGAATCGATGGTCCATGGGAATTCGGTATTCGAATCCGTAAGGGTTCGAATAAACGAAAGCTGTTTGAAAT
ACTGGAGGATTCGGACAACGAAGTCCTTCAACCCCAAAAATACAGACGAGCCATGGCCAAACAGGCCATGAGTGAGTCTAAGAAGAAAGCTGCAGAAGAAGGATTTAGTTATGAACTGCG
AGAGTGGCAAGTGCAATTGACGAAGTTGTTGGAAGAAGAACCCGATAACAGATCAATAATTTGGGTATATGGACCAAAGGGTGGAGAAGGTAAAACAGAGTTCGCTAAACATTTAGGGTT
AAAGGATGGTTGGACATATTTGCCTGGTGGTGAACTCAAAGATATGATGTACCTGTTATCAAAGGACATGAAAAATAATGTTGTAATTGATTTCCCCCGTTGTACCAAGGAATTTATTTC
ATATAAATTCCTTGAGATGGTTAAGAATAGAACTATATATAGTTATAAGTATGAACCAGTTGGGTCAATCGTATGTAATAAGGTTCATGTAGTGGTCTTCAGTAATGAGGAGCCAGATTA
TGTAAAATTATCATTGGATCGAATAAAGTTGATTTCTTGCTGAACACGCTATGAAATACAACACGCTATGACAAAAGGGGAAAAATGAAGAATCGGGGGTTGATTGGGCTATCCTAACGA
ATAAGGGCCGCAGGCCCGTCAAGATGGATGAACGGTCAGAATTGATTGCTTCGCCACGAAGCAAACAAAAAATGCGTGGACGAGAACGTGCCACGTAGCAAAACACTGTACTCCTTGGCG
CACTATATATAGGTGCGCCAAGGTCGGCCATAG

BBTA2
Best match to EU430730 100% coverage 98% identity; MG545616 100% coverage 97% identity; NC_038953 100% coverage 96% identity, 26 SNPs (98%) to BBTA2_alyu29

Rep protein best matches to AZL93963 100% coverage 99% identities (2 aa) 99% positives (1 aa); YP_009508281 100% coverage 94% identities (11 gaps in middle) 94% positives Banana bunchy top alphasatellite 2; YP_009508283.1 100% coverage 73% identities 88% positives Banana bunchy top alphasatellite 3

>BBTA2_alyu39
TATTACCCACCTCCGCGCACTACCTCCGCGCACCTATAAAATGTCTGCCTCACGATGGACATTTACGCTTCACTATTCCGACGCAACGGAGCGAGGCAAATTCCTCGCGACTTTGAAGGA
GGAAGATGTGCACTACGCCGTCGTCGGCGACGAAACTGCTCCGAACACCGGTCGGAAACATCTTCAAGGATATCTTTCCTTGAAGAAACGTTTTCGTATTAGCGGCATAAAGAAGAAATA
TTCGTCGAGAGCGCATTGGGAGAAAGCTCGAGGATCAGATTACGACAACAAGGCGTACTGTTCCAAAGAAGCCCTAATTCTTGAATTAGGGGTTCCTTGCCAAACAGGTTCGAATAAGCG
TAAATTAGCAGATATGGTTACAAGATCTCCGGAACGAATGAAAATTGAACAGCCAGAGATATTTCACCGATACGAATCGGTGAAGAAGATGAAAGAATTCAAAGAAAGGTATGTCTATCC
TATCCTCGATAGGCCATGGCAGGTACAATTAACGGAGTTAATTGAAGCAGAACCTGATGATCGAACGATCATCTGGGTATTCGGACCAAAAGGGAATGAAGGCAAATCAACGTATGCGAA
GTCATTAATCCAAAAGGATTGGTTCTACACCAGGGGAGGAAAGAAGGAGAACATATTGTTCGCCTACGTAGATGAAGGTTCGACCAAAAACGTTGTATTTGATCTTCCGCGTACAGTACA
AGAATTTATTAACTACGATGTTATCGAGGCACTGAAGGATAGAGTAATCGAGAGTACAAAATACAAGCCTGTGAAGTATTTAGAATTGAATACTGTACATGTAATAGTTATGGCTAATTT
TCTTCCTGATATGTGTAAAATATCTGAAGATCGAATAAATATAGTTGCTTGCTGAACACGCTATGACAAACGCACGCTATGACAAAAAGGGAAAAAAGAAAAATCGGGGGTTGATTGGGC
TATCCTAACGATTAAGGGCCGCAGGCCCGTCAAGATGGATCCAATAACCCGATAAGAAGGTAAACGGGTCTAAAACGATTTCTTCGCCCGCAAGCAACACCTTTAACCTCTGCGCACCTA
TATATAGCGGAGGTGGGATAG

DNA-C
24 SNPs to KY427060 (**Thailand**). **23-28 SNPs** to KM607098 KM607099 (**China**). **27-29 SNPs** to MF039864 MF039876 MF039870 (**Thailand**)

C protein (161 aa): 1 aa to KY427060 (**Thailand**) **2 aa** to KM607098 KM607099 (**China**) MF039864 (**Thailand**)

>C_alyu39
TATTACCCCCCTGCTCGGGACGGGACATCACGTGCATCAAACAAATGCACGTGACAAAGCAGTAGCTTGTAGCGAAAGATAACCATCACTATCACAAAAGTGAACGGAATATTCGTTGC
TTAGGGACTAAGCAACGTGGACAGATATTTGTTCGAGAAGCGACAATGGAGGCTATTTAAACCTGATGGTTTTGTGATTTCCGAAATCACTCATCGGAGCAGAAATGGAGTTTTGGGAAT
CGTCTGCCATGCCAGACGATGTCAAGAGAGTGATCAAGGAAATATATTGGGAGCATCGAAATAAACTTCTGTTCTGTCAGAAGTTGAAAGGCTGTGTAAGAAGGATTCTGGAGCATGGAA
ATCAAGATGATGCTCTAGACGCAGTGAAGGAGATGAAGACTTCTATTATTCGCTATAGCGAATATTTGAAGAAACCTTGTGTGGTATTGTGTTTTAGTACTAATAAATCTATTGCATATA
GGTTAAACACAATGGTGTTCTTTTATCATGAATATATTGAAGACCTAGGTGGTGACTACTCGGTATATCAAGATCTGTTCTGTGATGAGGAACTTCCTTATTCGTCGACAGAAGAAGAAG
ATGAAGAAGTAATATACAGGAATGTTATCATGGCATCGACAGAAGAGAAGATCTCTTGGAGTGAATGTCAAAAGATAGTTATATCAGATTATGATGTAACATTACTGTAATGAATATCCA
TTATCATAAATAAAAATAATGGAATGATGATTATGTATTCTAGTATAAATACATAATGGTATACGTATAGCATAAAATACATAAACCAACATACAACACGCTATGACAAACGGGGAAAAA
TGAAGAATCGGGGGTTGATTGGGCTATCTTAACGATTAAGGGCCGAAGGCCCGTTTAAATATGTGGTGGACGAAGTCCACAACACACAAAAAGTGAGCAGAACAACGGAATATAATGAGC
TGGCAACGTAGGGTCCATGTCCCGAGTTAGTGCGCCACGTGAGCAGGGGGGCTTAT

DNA-M
23-28 SNPs to KM607239 KM607240 (**China**). **53-54 SNPs** to KY427061 MF039871 MF039865 (**Thailand**)

M protein (117 aa): 0 aa to KC581796 (**Thailand**) **1 aa** to MF039871 MF039865 MF039877 KY427061 (**Thailand**)

>M_alyu39
TATTACCCCCAGCGCTCAGGACGGGACATCACGTGCAACTAACAAATGCACGTGACTGATATATACTACATAACGGTTTAACGAACCGTTATATAATGTTATAACGATAAGTCACGTGTG
ATAGAGACATGCACGTGACATAGTCAAATGTATTGAATAAACATTTGACGTCCGGATGCTTCCGACGGAAGCTTAGGTTACTTCGCGGCGAAGCAAAACATTTATATATTGGCCaGGACT
cCTGCCTATAAATAGGCAAGCAAGGAAATGGCATTAACAACAGAGCGAGTGAAACTATTCTTTGAATGGTTTCTGTTCATCGGAGCAATATTTATTGCGATAACAATATTATATATATTG
TTGGCGTTGCTCTTTGAGGTCCCCAAGTATATTAAGGATGTTGTGAGGTATCTCGTAGAATACCTGACCAGAAGACGTGTATGGATGCAGAGAACGCAGTTGACGGAGGCAACAGGAGAT
GTAGAGCTCGGCAGAGGTAGTGTGGAAGACAGACGGGATCAACAACCGGCTGTCATACCACATGCAACACAGGTTATCCCTTCGCATCAACCTAGAAGGGATGAGCAAGGAAGACGAGGA
AACGCTGGACCAATGTTTTAATACACAGTATTGTAATATACGAAATATAAATGGATAATGATATGTATTGTGAAACATAATATACGTGAAACATAATATATGTTTGATAATTTACATATT
TTAATATGTAAATTGTATACGAGTGTTGTATTTATAGAACATACAACACGCTATGACAAACAGGGAAAAATGAAGAATCGGGGGTTGATTGGTCTATCGTATCGCTTAAGGGCCGCAGGC
CCGTTGAAATGATTCTTAATAAAACAAATATACATGATACGGATAGTTGAATATATAAACAACGTAGTATAAATACAACAAAATGTTGTATACTAATTAAAATAATGAGAAGATAAGTAT
ATTTGTGAGGGATGATGATCACAACCACCACTTTAGTGGTGGGTCATATGTCCCGAGTTAGTGCGCCACGTAAGCGCTGGGGCTTAT

DNA-N
24-28 SNPs to MF039872 MF039878 MF039866 KY427062 (**Thailand**). **27-38 SNPs** to KM607387 KM607385 KM607386 (**China**)

N protein (154 aa): 0 aa to MF039872 MF039878 KY427062 (**Thailand**) KM607387 KM607385 (**China**) **1 aa** to MF039866 (**Thailand**)

>N_alyu39
TATTACCCCCCGTGCTTGGGACGGGACATGACGTCAGCATAGATTATAATGGGCCAACTAAAGGCCCATTTAATAGAATAGGCGGGCTTTTGACATATTTCAAAGGCCCAGCCTGGAAGT
GGATAATGTCACGTGCCTAATAAGAGGTTGCTTCGCCTCGAAGCAATCCGAATAAAAGTTGCGTATTCAATACGCAACTGAAGTCTATTAATATGGATGTCTCTGCCGAATAAATCAGAG
CGTATGCGAAGCAGAAGCGATGGATTGGGCAGAATCACAATTCAAGACATGTACTCATGGGTGCGATTGGAAGACGATATCATCGGATTCATCCGAGAATCGACAATATGTACCTTGCGT
CGACGCTGGCAGCGGAAGAAAGTCACCTCGCAAGGTACTGCTAAGATCTATTGAAGCTGTGTTTAATGGAAGCTTCAAAGGAAATAACAGGAATGTTCGTGGGTTTATATACGTATCAAT
ACGAGACGATGATGGAGAAATGCGTCCAGTACTCATAATACCATTCGGAGGATATGGATATCATAATGATTTCTATTATTTCGAAGGGAAGGGGAAAGTTGAATGTGATATATCATCAGA
TTATGTAGCGCCAGGAGTCGATTGGAGCAGAGACATGGAAGTTAGTATTAGTAACAGCAACAACTGTAATGAATTATGTGATCTGAAGTGTTATGTTGTTTGTTCGTTAAGAATAAAGGA
ATAACAGATGTGCTGTAATGATTATTAATAAAACTTATTTTCATGAAATTGATAGTTGTATAAAACATACAACACGCTATGACAAACAGGGAAAAATGAAAAATCAGGGGTTGATTGGTC
TATCGTATCGCTTAAGGGCCGCAGGCCCGTTGAAAAATAATAATCGAATTATAAACGTTTGATAATAATCAGAGATAGATGATAAGCATCCTAAAACATAGACGAAGTATATGGATGTAT
AATATAAAACAAGCATATAAATAAAATATGTGAACTAATCTCTGATTGGTGCAGAACGTAGCCCCACTAACTTTAAGTTAGTGGAAATGTCCCGATGACGTAAGCACGGGGGACTAT

DNA-R
6 SNPs to AB113660 (**Viet Nam**). **10-12 SNPs** to KM607677 KM607678 KM607676 KM607679 (**China**). **16-20 SNPs** to MF039879 MF039873 MF039867 KY427063 (**Thailand**)

R protein (286 aa): 0 aa to KM607676 KM607677 KM607679 (**China**) **1 aa** to AB113660 (**Vietnam**) MF039879 (**Thailand**)…

>R_alyu39
TATTACCCCCAGCGCTCGGGACGGGACATTTGCATCTATAAATAGACCTCCCCCCTCTCCACTACAAGATCATCATCGTCGACAGAAATGGCGCGATATGTGGTATGCTGGATGTTCACC
ATCAACAATCCCGCTTCACTACCAGTGATGCGGGATGAGTTCAAATATATGGTATATCAAGTGGAGAGGGGACAGGAGGGTACTCGTCATGTGCAGGGATACGTCGAGATGAAGAGACGA
AGTTCTCTGAAGCAGATGAGAGGCTTCTTCCCTGGCGCACACCTTGAGAAACGAAAGGGAAGCCAAGAAGAAGCACGGGCATACTGTATGAAGGAAGACACAAGAATCGAAGGTCCCTTC
GAGTTTGGTGCGTTTAAATTGTCATGTAATGATAATTTATTTGATGTCATACAGGATATGCGTGAAACGCACAAACGGCCTTTGGAGTATTTATATGAGTGTCCAAATACCTTCGATAGA
AGTAAGGATACATTATACAGAGTTCAAGCAGAGTTGAATAAAACGAAGGCGATGAATAGCTGGAAAACATCCTTCAGTTCTTGGACATCGGAAGTTGAAAATATTATGGCGGAGCCATGT
CATCGAAGGATAATTTGGGTCTATGGCCCAAATGGAGGAGAAGGAAAGACAACCTATGCAAAATATTTAATGAAGACGAAGAATGCGTTTTATTCTCCAGGAGGAAAATCATTGGATATA
TGTAGATTGTATAATTACGAGGAAATAGTTATATTTGATATTCCCAGATGCAAAGAGGAATATTTAAACTATGGTTTACTAGAAGAATTTAAAAATGGAATTATTCAAAGCGGGAAATAT
GAACCCGTTTTGAAAATTGTAGAATATGTGGAAGTCATTGTAATGGCTAACTTCCTTCCGAAGGAAGGAATCTTTTCAGAAGATCGAATAAAGCTAGTTGCTTGCTGAACACGCTATGAC
AATCGTACGCTATGACAAAAGGGGAAAAGCAAAGATTCGGGGGTTGATTGTGCTATCCTAACGATTAAGGGCCGCAGGCCCGTCAAGATGGACGGCTTGATCAGATGTCCCGAGTTAGTG
CGCCACGTAAGCGCTGGGGCTTAT

DNA-S
11 SNPs to AB113662 (**Viet Nam**). **15/16 SNPs** to KM607535 KM607534 (**China**). **19 SNPs** to MF039874 (**Thailand**)

S protein (170 aa): 0 aa to AY33771 AY494786 HQ378191 (**China**) FJ787433 FJ787434 (**Philippines**) **1 aa** to several isolates

>S_alyu39
TATTACCCCCAGCGCTCGGGACGGGACATGGGCTAATGGATCATGGAATAAGGGCCCATCGGGCCCGTTAAGATGGGTTTTGGGCTTATGGGCTTTATCCAGAAGACCAAAAACAGGCGG
GAACCGTCCCAATTTCAAATTTAGATAGCTTGCCCCGCAAGCAAACTAAAAGTCTATATATACCAGTGTAGACATATTGTTCAGAGAACAAATGGCGAGGTTTCCGAAGAAATCGATCAA
GAAGAGGCGGGTTGGGCGACGGAAGTATGGAAGCAAGGCGGCAACCAGTCACGACTACTCGTCGTTAGGTTCAATATTGGTTCCTGAAAATACCGTCAAGGTATTTAGGATTGAGCCTAC
TGATAAAACATTACCCAGGTATTTTATCTGGAAAATGTTTATGCTTTTGGTGTGCAAGGTGAAGCCCGGAAGAATACTTCACTGGGCTATGATTAAAAGTTCATGGGAAATCAACCAGCC
GACTACATGTCTGGAAGCACCAGGTTTATTTATTAAACCTGAACATAGCCATCTGGTGAAACTGGTATGCAGTGGGGAACTTGAAGCCGGAGTCGCAACAGGGACATCAGATGTTGAATG
TCTTCTAAGGAAGACAACCGTGTTGAGGAAGAATGTAACAGAGGTGGATTACTTGTATTTGGCATTTTATTGTAGTGCTGGAGTTAGTATTAACTACCAGAACAGAATTACATATCATGT
ATGATATGTTTATGTAAACATAAACCTTTGTATGGAATAATGACCAAATAACATACAACACACTATGAAATACAACACGCTATGACAAACAGGGAAAAATGAAGAATCGGGGGTTGATTG
GTCTATCGTATCGCTTAAGGGCCGCAGGCCCGTTGAAATGATTCTTAATAAAACAAATATACATGATACGGATAGTTGAATATATAAACAACGATGTATAAATACAACAGAATGTTGTAT
ACCAATTAAAAATAATGAGAAGAAAAGAATATTTGTGAAGGATAAGCATCAGAACCACCACTTTAGTGGTGGGTCATATGTCCCGAGTTAGTGCGCCACGTAAGCGCTGGGGCTTAT

DNA-U3
42-46 SNPs to KY427065 MF039869 (**Thailand**)

U3 protein (39 aa): 0 aa to KY427065 (**Thailand)**

>U3_alyu39
TATTACCCCCAGCCCCCAGGACGGGACATGGGCTTTTTAAATGGGCCGAGAGAGTTTGAACAGTTCAGTATCTTCGTTATTGGGCCATCTGGCCCAATAATTAAGAGAACGTGTTCAAAA
TCGGGGTTTGACCGAAGGTCAAGGTAGACGGTCAACAATATTCTGGCTTGCGGAGCAAGCAACACGAATTAAATATTTAATTCGTATGACACGTGGACGGACCGAAATACTCCTGTAACT
cTATAAATAGCTTAAATCTGGCTTGGATAATTGCTCTCGCTCTTCTGTCAAAGCTGTTGTGTTGAGGCGGAAGATCGCCATCGGCGATCATCGGACGAAAAGCTGCAAGAGAGACGGAGA
ACCATGCTACGAAGCGTATATCGGGTATTTATAGACTTCTAGCGCAGCTAGAAGTTTCCTTGTACTTGATATTGTATTTTGTAAATTACGAAGAAATTCGTATATTGATAATAATAAAAC
ATCTGGGATTGTTAATGTTTACATTAACTAGTATTAATAATGTACAATAACAAATATTGTATGAGGAACGAATACAAGAATAATAAAATATGAGGAGCGTAGCGTGATAAACAGTTGTTT
AAGGTATAATTAAATTAATTATGCAATGTCCTGATAATACGGTATAAGTTGAAGTATGAGGTGAAAGAGGAGATATTAGAATATTAAAAACCCAATTATATTATTTATAATACATACAAC
ACGCTATGACAAACGGGGAAAAAAGAAAAATCGGGGGTTGATTGGTCTATCCTAACGATTAAGGGCCGCAGGCCCGTCAAGATGGATGAACGGTTAGATTTGATTGCTTAGCCACGAAGA
AAGGAATCGTTTTGGGACCACAGACAAGACAGCTGTCACTACTTTTAAAAAATAATATAATAACCAATTGACGATAGTACCCCTCCCAAAGATGACGTATAGGGGTGTCCCGAGGTAATT
TAACATAGCTCTGACAAGAGATAGTGGACGTTGGATGCCACGATCGGACGATCGTGGTTTGATGAACCAATGATGACGTAGGGGCTGGGGCTTAT

ALYU-40 - Musa AAA red - Vietnam

Virome
1. **BBTV** six components
2. **BBTA5** alyu40=38

BBTA5
Best matches to MF510475 MF510474 33% coverage 66.5% identity Faba bean necrotic yellows virus associated alphasatellite 2 isolates; MK291270 72% coverage 66.9% identity Sophora yellow stunt alphasatellite 3; AJ132187 AJ005966 73% coverage 66.3-65.8% identity Faba bean necrotic yellows C9 alphasatellite

Rep protein best matches to QBF29185 100% coverage 59% identities 77% positives Sophora yellow stunt alphasatellite 3; ATU31571 100% coverage 62% identities 80% positives Faba bean necrotic yellows virus associated alphasatellite 2; ARI50297 100% coverage 62% identities 80% positives Sophora alopecuroides yellow stunt alphasatellite 3

>BBTA5_alyu40

TATTACCCGACCTTGGCTCACTGGCTCACTATAAAAGGGGGAAATGTCGCAGCAGCGAAATTGGGTATTCACGCGTAACTTCTCCGGCGACCCGCCGATTTTAAAGTTCGCCGGAGAAAC
TCAATACGCGTGCTGGCAACATGAGAAGAAGAATCACGACCATCTTCAGGGAGTGATTCAATTGAAGAAGAAGCTCCGGATGAACGCAGTGAAGACGTTAATTGGGGGAAATCCACACCT
CGAAGCCATGAGAGGAACAATCGACGAAGCAATAAGGTACGTGACGAAAGAAGAAACACGAGTAGCAGGTCCGTGGGAATTCGGAGAATTACTACGAAAAGGATCTCATAAGAGGAAATT
GATGGAATTGCTCGACGATCCAGATAACGAAATAATGGAACCTCAAAAATATCGACGCGCCATTACTAAACAAGCAATGGACGCGTCAAAGAAGAAGGCGGAATTAGGGTTTCCTTACGA
CTTAAAGGAGTGGCAAAAGATGGTTATTGAGTTAATCGAAGAACAACCAGACAACAGAACTATTATCTGGGTCTATGGACCTAATGGTGGAGAAGGTAAAACGCAGTTCGCAAAGCATTA
TGGATTACTCAAAGGATGGACATATCTACCAGGTGGTGAACTGAAAGACATGATGTATCTGTTGGCAAAAGAAATTAATAATAACGTTATTATTGATTTCCCCCGATGTACAAAGGATTT
CATATCCTATAAGTTTATTGAGATGGTAAAGAACCGATGCATATTTAGCTATAAATATGAACCAATCGGGGCGATTGTAAGTAATGAAGTCCATGTAATTGTAATGTCGAATGAGCTTCC
AGATTATTCAAAAATTTCGGAAGACAGAATAAAATTAATATTTACATAACGCACACTATGACAAAAGGGCAAAAATGCAAAATCGGGGGTTGATTTGTCTATATTTACGAATAAGGGCCG
CAGGCCCGTACACATTGCGCGGGAAATTCAAATTCGGACGCCAAATATTATTAAATACAATACACCGCCTTTTAGAAATCCGTGGACGAGAAGATGCCACGTAGCAAAACACTGTACTCC
TTGGCTCACTATAAATACCTGAGCCAAGGTCGGGCATAG

DNA-C
23 SNPs to KY427060 (**Thailand**). **25-28 SNPs** to KM607098 KM607099 (**China**). **26-28 SNPs** to MF039864 MF039876 MF039870 (**Thailand**)

C protein (161 aa): 1 aa to KY427060 (**Thailand**) **2 aa** to KM607098 KM607099 (**China**) MF039864 (**Thailand**)

>C_alyu40
TATTACCCCCCCTGCTCGGGACGGGACATCACGTGCATCAACCAAATGCACGTGACAAAGGAGTAGCTTGTAGCGAAAGATAACCATCACTATCACAAAAGTGAGAAGAATATTCGTTGC
TTAGGGACTAAGCAACGTGGTCAGATATTTGTTCGAGAAGCGAAAATGGAGGCTATTTAAACCTGATGGTCTTGTGATTTCCGAAATCACTCATCGGAGCAGAAATGGAGTTCTGGGAAT
CGTCTGCTATGCCAGACGATGTCAAGAGAGTGATCAAGGAAATATATTGGGAGCATCGAAATAAACTTCTGTTCTGTCAGAAGTTGAAAGGCTGTGTAAGAAGGATTCTGGAGCATGGAA
ATCAAGATGACGCTCTAGACGCAGTGAAGGAGATGAAGACTTCTATTATTCGCTATAGCGAATATTTGAAGAAACCTTGTGTGGTATTGTGTTTTAGTACTAATAAATCTATTGCATATA
GGTTAAACACAATGGTGTTCTTTTATCATGAATATATTGAAGACCTAGGTGGTGACTACTCGGTATATCAAGATCTGTTCTGTGATGAGGAACTTCCTTATTCGTCGACAGAAGAAGAAG
ATGAAGAAGTAATATACAGGAATGTTATCATGGCATCGACAGAAGAGAAGATCTCTTGGAGTGAATGTCAAAAGATAGTTATATCAGATTATGATGTAACATTACTGTAATGAATATCCA
TTATCATAAATAAAATAATGGAATGATGATTATGTATTCTAGTATAAATACATAATGGTATACGTATAGCATAAAATACATAAACCAACATACAACACGCTATGACAAACAGGGAAAAAT
GAAGAATCGGGGGTTGATTGGGCTATCTTAACGATTAAGGGCCGAAGGCCCGTTTAAATATGTGGTGGACGAAGTCCACAACACATAAAAAGTGATCAGAACAATGGAATATAATGAGCT
GGCAACGTAGGGACCATGTCCCGAGTTAGTGCGCCACGTGAGCAGGGGGGCTTAT

DNA-M
16-21 SNPs to KM607239 KM607240 (**China**). **55-56 SNPs** to KY427061 MF039871 MF039865 MF039876 (**Thailand**)

M protein (117 aa): 1 aa to KC581796 (**Thailand**) **2 aa** to MF039871 MF039865 MF039877 KY427061 (**Thailand**)

>M_alyu40
TATTACCCCCAGCGCTCAGGACGGGACATCACGTGCAACTAACAAATGCACGTGACTGATATATACTACATAACGGTTTAACGAACCGTTATATAATGTTATAACGAAAAGTCACGTGTG
ATAGAGACCTGCACGTGACATAGTCAAATGAATTGAATAAACATTTGACGTCCGGATGCTTCCGACGGAAGCTTAGGTTACTTCGTGGCGAAGCAAAACATTTATATATTGGCCTGGACT
GCTGCCTATAAATAGGCAAGCTAGGAAATGGCATTAACAACAGAGCGGGTGAAACTATTCTTTGAATGGTTTCTGTTCATCGGTGCAATATTTATTGCGATAACAATATTATATATATTG
TTGGCGTTGCTCTTTGAAGTCCCCAAGTATATTAAGGATGTTGTGAGGTATCTCGTAGAATACCTGACCAGAAGACGTGTATGGATGCAGAGAACGCAGTTGACGGAGGCAACAGGAGAC
GTAGAGCTCGGCAGAGGCAGTGTGGAAGACAGACGGGATCAACAACCGGCTGTCATACCACATGCAACACAGGTTATCCCTTCGCATCAACCTAGAAGGGATGATCAAGGAAGACGAGGA
AACGCAGGACCAATGTTTTAATACACGGTATTGTAATATACGAAATATAAATGGATAATGATATGTAATGTGAAACATAATATACGTGAAACATAATATATGTTTGATAATTTACATATT
ATAATATGTAAATTGTATACGAGTGTTGTATTTATAGAACATACAACACGCTATGACAAACAGGGAAAAATGAAGAATCGGGGGTTGATTGGTCTATCGTATCGCTTAAGGGCCGCAGGC
CCGTTGAAATGATTCTTAATAAAACAAATATACATGATACGGATAGTTGAATATATAAACAACGAAGTATAAATACAACAGAATGGTGTATACTAATTAAAATAATGAGAAGATAAGTAT
ATTTGTGAGGGATGATGATCACAACCACCACTTTAGTGGTGGGTCATATGTCCCGAGTTAGTGCGCCACGTAAGCGCTGGGGCTTAT

DNA-N
21-25 SNPs to MF039872 MF039878 KY427062 MF039866 (**Thailand**). **26 SNPs** to KM607387 KM607385 (**China**)

N protein (154 aa): 0 aa to MF039872 MF039878 KY427062 (**Thailand**) KM607387 KM607385 (**China**) **1 aa** to MF039866 (**Thailand**)

>N_alyu40
TATTACCCCCGTGCTTGGGACGGGACATGACGTCAGCAAAGATTATAATGGGCCAACTAAAGGCCCATTTAATAGAATAGGCGGGCTTTTGACATATTTCAAAGGCCCAGCCTGGAAGT
GGATAATGTCACGTGCCTAATAAGAGGTTGCTTCGCCTCGAAGCAATCCGAATAAATGTTGCGTATTCAATACGCAACTGAAGTCTATTAATATGGATGTCTCTGCCGAATAAATCAGAG
CGTAAGCGAAGCAGAAGCGATGGATTGGGCAGAATCACAATTCAAGACATGTACTCATGGGTGCGATTGGAAGACGATATCTTCGGATTCATCCGAGAATCGACAATATGTACCTTGCGT
CGACGCTGGCAGCGGAAGAAAGTCTCCTCGCAAGGTACTGCTAAGATCTATTGAAGCTGTGTTTAATGGAAGCTTCAAAGGAAATAACAGGAATGTTCGTGGGTTTATATACGTATCAAT
ACGAGACGATGATGGAGAAATGCGTCCAGTACTCATAATACCATTCGGAGGATATGGATATCATAATGATTTCTATTATTTCGAAGGGAAGGGGAAAGTTGAATGTGATATATCATCAGA
TTATGTAGCGCCAGGAGTCGATTGGAGCAGAGACATGGAAGTTAGTATTAGTAACAGCAACAACTGTAATGAATTATGTGATCTGAAGTGTTATGTTGTTTGTTCGTTAAGAATAAAGGA
ATAACAGATGTGCTGTAATGATTATTAATAAAACATATTTTCATGTAATTGATAGTTGTATAAAACATACAACACGCTATGACAAACAGGGAAAAATGAAATATCGGGGGTTGATTGGTC
TATCGTATCGCTTAAGGGCCGCAGGCCCGTTGAAAAATAATAATCGAATTATAAACGTTTGATAATAATCAGAGAAAGATGATAAGCATCCAAAAACATAGACGAAGTATATGGCTGTAT
AATATAAAAGAAGCATATAAATAAAATATGTAAACTAATCTCTGATTGGTTTAGAATGTAACCCCACCAACTTTATGTTGGTGGAAATGTCCCGATGACGTAAGCACGGGGGACTAT

DNA-R
7 SNPs to AB113660 (**Viet Nam**). **8-10 SNPs** to KM607677 KM607678 KM607676 KM607679 (**China**). **16-19 SNPs** to MF039879 MF039873 KY427063 MF039867 (**Thailand: Chonburi**)

R protein (286 aa): 0 aa to KM607676 KM607677 KM607679 (**China**) **1 aa to** AB113660 (**Vietnam**) MF039879 (**Thailand**)…

>R_alyu40
TATTACCCCCAGCGCTCGGGACGGGACATTTGCATCTATAAATAGACCTCCCCCCTCTCCACTACAAGATCATCATCGTCGACAGAAATGGCGCGATATGTGGTATGCTGGATGTTCACC
ATCAACAATCCCGCTTCACTACCAGTGATGCGGGATGAGTTCAAATATATGGTATATCAAGTGGAGAGGGGACAGGAGGGTACTCGTCATGTGCAGGGATACGTCGAGATGAAGAGACGA
AGCTCTCTGAAGCAGATGAGAGGCTTCTTCCCTGGCGCACACCTTGAGAAACGAAAGGGAAGCCAAGAAGAAGCACGGGCATACTGTATGAAGGAAGACACAAGAATCGAAGGTCCCTTC
GAGTTTGGTGCCTTTAAATTGTCATGTAATGATAATTTATTTGATGTCATACAGGATATGCGTGAAACGCACAAACGGCCTCTGGAGTATTTATATGAGTGTCCAAATACCTTCGATAGA
AGTAAGGATACATTATACAGAGTTCAAGCAGAGTTGAATAAAACGAAGGCGATGAATAGCTGGAAAACATCCTTCAGTTCTTGGACATCGGAAGTTGAAAATATTATGGCGGAGCCATGT
CATCGAAGGATAATTTGGGTCTATGGCCCAAATGGAGGAGAAGGAAAGACAACTTATGCAAAATATTTAATGAAGACGAAGAATGCGTTTTATTCTCCAGGAGGAAAATCATTGGATATA
TGTAGATTGTATAATTACGAGGAAATAGTTATATTTGATATTCCCAGATGCAAAGAGGAATATTTAAACTATGGTTTACTAGAAGAATTTAAAAATGGAATTATTCAAAGCGGGAAATAT
GAACCCGTTTTGAAAATTGTAGAATATGTGGAAGTCATTGTAATGGCTAACTTCCTTCCGAAGGAAGGAATCTTTTCAGAAGATCGAATAAAGCTAGTTGCTTGCTGAACACGCTATGAC
AATCGTACGCTATGACAAAAGGGGAAAAGCAAAGATTCGGGGGTTGATTGTGCTATCCTAACGATTAAGGGCCGCAGGCCCGTCAAGATGGACGGCTTGATCAGATGTCCCGAGTTAGTG
CGCCACGTAAGCGCTGGGGCTTAT

DNA-S
18 SNPs to AB113662 (**Viet Nam**). **18/19/21 SNPs** to KM607535 KM607534 KM607536 (**China**). **20 SNPs** to MF039874 (**Thailand**)

S protein (170 aa): 0 aa to AY337715 AY494786 HQ378191 (**China**) FJ787433 FJ787434 (**Philippines**) **1 aa** to several isolates

>S_alyu40
TATTACCCCCAGCGCTCGGGACGGGACATGGGCTAATGGATCATGGAATAAGGGCCCATCGGGCCCGTTAAGATGGGTTTTGGGCTTATGGGCTTTATCCAGAAGACCAAAAACAGGCGG
GAACCGTCCCAATTTCAAATTTAGATAGCTTGCCCCGCAAGCAAACTAAAAGTCTATATATACCAGTGTAGACATATTGTTCAGATAACAAATGGCGAGGTTTCCGAAGAAATCGATCAA
GAAGAGGCGGGTTGGGCGACGGAAGTATGGAAGCAAGGCGGCAACCAGTCACGACTACTCGTCGTTAGGTTCAATATTGGTTCCTGAAAATACCGTCAAGGTATTTAGGATTGAGCCTAC
CGATAAAACATTACCCAGATATTTTATCTGGAAAATGTTTATGCTTTTGGTGTGCAAGGTGAAGCCCGGAAGAATACTTCACTGGGCTATGATTAAAAGTTCATGGGAAATCAACCAGCC
GACTACCTGTCTGGAAGCACCAGGTTTATTTATAAAACCTGAACATAGCCATCTGGTGAAACTGGTATGCAGTGGGGAACTTGAAGCCGGAGTCGCAACAGGGACATCAGATGTTGAATG
TCTTCTAAGGAAGACAACCGTGTTGAGGAAGAATGTAACAGAGGTGGATTACTTGTATTTGGCATTTTATTGTAGTGCTGGAGTTAGTATTAACTACCAGAACAGAATTACATATCATGT
ATGATATGTTTATGTAAACATAAACCTTTGTATGGAATAATGACCAAATAACATACAACACACTATGAAATACAACACGCTATGACAAACAGGGAAAAATGAAGAATCGGGGGTTGATTG
GTCTATCGTATCGCTTAAGGGCCGCAGGCCCGTTGAAATGATTCTTTATAAAACAAATATACAGGATACGGATAGTTGAATATATAAACAACGATGTATAAATACAACAGAATGTTGTAT
ACCAATTAAAAAAAATGAAAAGAAAAGAATATTTGTGAAGGATAAGCATCAGAACCACCACTTTAGTGGTGGGTCATATGTCCCGAGTTAGTGCGCCACGTAAGCGCTGGGGACTAT

DNA-U3

28-38 SNPs to MF039881 MF039875 KY427065 MF039869 (**Thailand: Chonburi**)

U3 protein (34 aa): a **truncated** version of a 39 aa protein encoded by KY427065 (**Thailand)**

>U3_alyu40
TATTACCCCCAGCCCCCAGGACGGGACATGGGCTTTTTAA**ATG**GGCCGAGAGAGTTTGAACAGTTCAGTATCTTCGTTATTGGGCCATCTGGCCCAATAATTAAGAGAACGTGTTCAAAA
TCGGGAGTT**TGA**CCGAAGGTCAAGGTAGACGGTCAACAATATTCTGGCTTGCGGAGCAAGCAACACGAATTAAATATTTAATTCGTATGACACGTGGACGGACCGAAATACTCCTGTACC
TCTATAAATAGCTTAAAACTGGCTTGGATAATTGCTCTCGCTCTTCTGTCAAAGCTGTTGTGTTGAGGCGGAAGATCGCCATCGGCGATCATCAGACGAAAAGCTGCAAGAGAGACGGAG
AACCATGCTGCGAAGCGTATATCGGGTATTTATAGACTTCTAGCGCAGCTAGAAGTTTCCTTGTACTTGATATTGTATTTTGTAAATTACGAAGAAATTCGTAGATTGATAATAATAAAA
CATCTGGGTTTGTTAATGTTTACATTAACCAGTATTAATAATGTACAATAACAAAAATTGTATGAGGAACGAATACAATAATATTAAAATATGAGGAGCGTAGCGTGATAAACAGGTGTT
TAAGGTATAATTAAATTAATTATGCAAAGTCATGATAATACGGTATAAGTTGAAGTATGAGGTGAAAGAGGAGATATTAGAATATTAAAAACCCAATTATATTATTTATAATACATACAA
CACGCTATGACAAAAGGGGAAAAAAGAAAAATCGGGGGTTGATTGGTCTATCCTAACGATTAAGGGCCGCAGGCCCGTCAAGATGGATGAACGGTCAGATTTTATTGCTTAGCCACGAAG
AAAGGAATCTTTTTGGGACCACAGATAAGACAGCTGTCACTACTTTTTAAAATAATATAATAACCAATTGACGATAGTACCCCTCCCAAAGATGACGTATAGGGGTGTCCCGAGGTAATT
TAACATAGCTCTGACAAGAGATAGTGGACGTTGGATGCCACGATCGGACGATCGTGGTTTGATGAACTAATGATGACGTAGGGGCTGGGGCTTAT

The insA in U (truncating ORF1) is confirmed by most reads (no minor SNP).

ALYU-41 - Chromolaena odorata - Laos

Neighbour to Musa ALYU-42

Virome:
1. **BBTV** 5 core components (distinct genetic variants) **DNA-N is at a cross-contamination level**
2. **BBTA5** alyu41 isolate: **1 SNP compared to BBTA5_alyu42 (Musa neighbour is invariant at this position)**
3. **Cucumber mosaic virus** (detected by IC-PCR)

At 5x – BBTA5, incomplete C, partial M

1. BBTA5 1 SNP to alyu42

>BBTA5_alyu41
TATTACCCGACCTTGGCTCACTTGCTCACTATAAAAGGGGGAGATGTCGCAGCAGCGAAATTGGGTATTCACGCGTAACTTCTCCGGCGACCCGCCGATTTTAAAGTTCGCCGGAGAAAC
TCAATACGCGTGCTGGCAACATGAGAAGAAGAATCACGACCATCTTCAGGGAGTGATTCAATTGAAGAAGAAGCTTCGGATGAACGCAGTGAAGACGTTAATTGGGGGAAATCCACACCT
CGAAGCCATGAGAGGAACAATCGACGAAGCAATAAGGTACGTGACGAAAGAAGAGACACGAGTAGCAGGTCCGTGGGAATTCGGAGAATTACTACGAAAAGGATCTCATAAGAGGAAATT
GATGGAATTGCTCGACGATCCAGATAACGAAATAATGGAACCTCAAAAATATCGACGCGCCATTACTAAACAAGCAATGGACGCGTCAAAGAAGAAGGCGGAATTAGGGTTTCCTTACGA
CTTAAAGGAGTGGCAAAAGATGGTTATTGAGTTAATCGAAGAACAACCCGACAACAGAACTATTATCTGGGTGTATGGTCCTAATGGTGGAGAAGGTAAAACGCAGTTCGCAAAGCATTA
TGGATTACTCAAAGGATGGACATATCTACCAGGTGGTGAACTGAAAGACATGATGTATCTGTTGGCAAAAGAAATTAATAATAACGTTATTATTGATTTCCCCCGATGTACAAAGGATTT
CATATCCTATAAGTTTATTGAGATGGTAAAGAACCGATGCATATTTAGTTATAAATATGAACCAATCGGGGCGATTGTAAGTAATGAAGTCCATGTAATTGTAATGTCGAATGAGCTTCC
AGATTATTCAAAAATTTCGGAAGACAGAATAAAATTAATATTTACATAACGCACACTATGACAAAAGGGCAAAAATGAAAAATCGGGGGTTGATTTGTCTATATTTACGATTAAGGGCCG
CAGGCCCGTACACTTTGCGCGGGAAATTCAAAATCCGGACGCCAAATTTTATTAAATACAATACACCGCCTTTTAGAAATCCGTGGACGAGAAGATGCCACGTATCAAAACACTGTACTC
CTTGGCTCACTATAAATACCTGAGCCAAGGTCGGGCATAG

A-to-G

DNA-C = ALYU-42

>C_alyu41
TATTACCCCCCCTGCTCGGGACGGGACATCACGTGCATCTAACAAATGCACGTGACAATGCAGTAGCTTGTAGCGAAAGATAACCATCACTATCATAAAAGTGAAAGGAATATTCGTTGC
TTAGGGACTAAGCAACGTGAACAGATATTTGTTCGAGAAGCGACAATGGAGGCTATTTAAACCTGATGGTTTTGTGATTTCCGAAATCACTCATCGGAGAAGAAATGGAGTTCTGGGAAT
CGTCTGCCATGCCAGACGATGTCAAGAGAGTGATCAAGGAAATATATTGGGAGCATCGAAACAAACTTCTGTTTTGTCAGAAGTTGAAAGGCTGTGTAAGAAGGATTCTGGAGCATGGAA
ATCAAGATGATGCTCTAGACGCAGTGAAGGAGATGAAGACTTCTATTATTCGCTATAGCGAATATTTGAAGAAACCTTGTGTGGTATTGTGTTTTAGTACTAATAAATCTATTGCATATA
GGTTAAACACAATGGTGTTCTTTTATCATGAATATATTGAAGACCTAGGTGGTGACTACTCGGTATATCAAGATTTGTTTTGTGATGAGGAACTTCCTTATTCGTCGACAGAAGAAGAAG
ATGAAGAAGTAATATACAGGAATGTTATCATGGCATCGACAGAAGAGAAGATCTCTTGGAGTGAATGTCAAAAGATAGTTATATCAGATTATGATGTAACATTACTGTAATGAATATCCA
TTATGATAAATAAAATAATGGAATGATGATTATGTATTCTAGTATAAATACATAATGGTATACGTATAGCATAAAATACATATACCTACATACAACACACTATAACAAACAGGGAAAAAT
GAAGAATCGGGGGTTGATTGGGCTATCTTAACGATTAAGGGCCGCAGGCCCGTTTAAATATGTGGTGGACGAAGTCCACAACACATAAAAAGGTATCAGAACAATGGAATATAATGAGCT
GGCAACGTAGGGACCATGTCCCGAGTTAGTGCGCCACGTGAGCAGGGGGGCTTAT

DNA-M 6 SNPs to ALYU-42
MP protein: identical to ALYU-42

>M_alyu41
TATTACCCCCAGCGCTCAGGACGGGACATCACGTGCAACTAACAAATGCACGTGACTGATATATACTACATAACGGTTTATTGAACCGTTATATATTGTTGTAACGCAAAGTCACGTGTG
ATAGCGAAATGCACGTGACCAAGTCAAATGTATTGAATAAACATTTGACGTCCGGATGCTTCCTACGGAAGCCATGGTTGCTTCGTGGCGAAGCAAAACATTTATATATTGGCTTGGATT
GCTGCCTATAAATAGGCACGCAGGGAAATGGCATTAACAACAGAGCGAGTGAAACTATTCTTTGAATGGTTTCTGTTTATTGGTGCAATATTTATTGCGATAACAATATTATACATATTG
TTGGCGTTGCTCTTTGAGGTCCCCAAGTATATTAAGGATGTTGTGAGGTATCTCGTAGAATACCTGACCAGACGACGTGTATGGATGCAGAGAACGCAGTTGACGGAGGCAACAGGAGAT
GTAGAACTCGGCAGAGGTAGTGTGGAAGACAGACGGGATCAACAACCGGCTGTCATTCCACATGCAACACAGGTTATCCCTTCACATCAACCTAGAAGGGATGAGCAAGGAAGACGAGGA
AACGCCGGACCGATGTTTTAATACACGGTATTGTAATATACGAAATATAAATGGATAATGATAAGTATTGTGAAACAAAATATATGTGAAACATAATATATGTTTGATAATTTACATATT
GTAATATGTGAATTGTATACGAGTGTTGTATTTATAAAACATACAACACGCTATGACAAACAGGGAAAAATGAAGAATCGGGGGTTGATTGGTCTATCGTATCGCTTAAGGGCCGCAGGC
CCGTTGAAATGATTCTTTATAAAACAAATATACATGATACGGATTGTTGAAAATATAATCAACGATGTATAAATACAACAGAATGTTGTATACCAATTAAAAAAAAGAGAAGATAAGTAT
ATTTGTGAAGGATATGGATCACAACCACCACTTTAGTGGTGGGTCATATGTCCCGAGTTAGTGCGCCACGTAAGCGCTGGGGCTTAT

DNA-N missing or at a very low level = ALYU-42

>N_alyu41
tattacccccgtgctcgggacgggacatgacgtcagcatagattataatgggccaactaaaggcccatttaatagaataggcgggcttttgacatatttcaaaggcccagcctggaagt
ggataatgtcacgtgccgaataagaggttgcttcgcctcgaagcaacccgaacaaatgttgcgtattcaatacgcaactgaagtctattaatatggatgtctctgccgaataaatcagag
cgtaagcgaagcagaagcgatggattgggcagaatcacaattcaagacatgtactcatgggtgcgattggaagacgatatcatcggattcatccgagaatcgacaatatgtaccttgcgt
cgacgcaggcagcggaagaaagtcacctcgcaaggtactgctaagatctattgaagctgtgtttaatggaagcttcaaaggaaataacaggaatgttcgtgggtttatatacgtatcaat
acgagacgatgatggagaaatgcgtccagtactcataataccattcggaggatatggatatcataatgatttctattatttcgaagggaaggggaaagttgaatgtgatatatcatcaga
ttatgtagcgccaggagtcgattggagcagagacatggaagttagtattagtaacagcaacaactgtaatgaattatgtgatctgaagtgttatgttgtttgttcgttaagaataaagga
ataacagatgtgctgtaatgaatattaataaaatatattttcatgtaattgatagttgtataaaacatacaacacgctatgacaaacagggaaaaatgaaaaatcaggggttgattggtc
tatcgtatcgcttaagggccgcaggcccgttgaaaaataataatcgaattattaacgtttgataataatcagagatagatgataagcatccaaaaaacatagacgaagtatatggctgta
taataaaaacaagcatataaataaaatatgtgaactaatctctgattggtttacaacgtagccccaccaactttatgttggtggaaatgtcccgatgacgtaagcacgggggactat

DNA-R 2 SNPs to ALYU-42
Rep protein: identical to ALYU-42

>R_alyu41
TATTACCCCCAGCGCTCGGGACGGGACATTTGCATCTATAAATAGACCTCCCCCCCCTCCACTACAAGATCATCATCGTCGACAGAAATGGCGCGATATGTGGTATGCTGGATGTTCACC
ATCAACAATCCCGCTTCACTACCAGTGATGCGGGATGAGTTTAAATATATGGTATATCAAGTGGAGAGGGGACAGGAGGGTACTCGTCATGTGCAGGGATACGTCGAGATGAAGAGACGA
AGTTCTCTGAAGCAGATGAGAGGCTTCTTCCCTGGCGCACACCTTGAGAAACGAAAGGGAAGCCAAGAAGAAGCACGGGCATACTGTATGAAGGAAGACACAAGAATCGAAGGTCCCTTC
GAGTTTGGTGCCTTTAAATTGTCATGTAATGATAATTTATTTGATGTCATACAGGATATGCGTGAAACGCACAAACGGCCTTTGGAGTATTTATATGAGTGTCCAAATACCTTCGATAGA

AGTAAGGATACATTATACAGAGTTCAAGCAGAGTTGAATAAAACGAAGGCGATGAATAGCTGGAAAACATCCTTCAGTTCTTGGACATCGGAAGTTGAAAATATTATGGCGGAGCCATGT
CATCGAAGGATAATTTGGGTCTATGGCCCAAATGGAGGAGAAGGAAAGACAACTTATGCAAAATATTTAATGAAGACGAAGAATGCGTTTTATTCGCCAGGAGGAAAATCTTTGGATATA
TGTAGATTGTATAATTATGAAGAAATAGTTATATTTGATATTCCCAGATGCAAAGAGGAATATTTAAACTATGGTTTATTAGAAGAATTTAAAAATGGAATTATTCAAAGCGGGAAATAT
GAACCCGTTTTGAAAATTGTAGAATATGTGGAAGTCATTGTAATGGCTAACTTCCTTCCGAAGGAAGGAATCTTCTCTGAAGATCGAATAAAGCTAGTTGCTTGCTGAACACGCTATGAC
AATCGTACGCTATGACAAAAGGGGAAAAGCAAAGA**t**TCGGGGGTTGATTG**t**GCTATCCTAACGATTAAGGGCCGCAGGCCCGTCAAGATGGACGGTTTGATCAGATGTCCCGAGTTAGTG
CGCCACGTAAGCGCTGGGGCTTAT

DNA-S 12 SNPs to ALYU-42
CP protein: identical to ALYU-42

>S_alyu41
TATTACCCCCAGCGCTCGGGACGGGACATGGGCTAATGGATCATGGAATAAGGGCCCATCGGGCCCGTTAAGATGGGTTTTGGGCTTATGGGCTTTATCCAGAAGACCAAAAACAGGCGG
GAACCGTCCCAATTTCAAATTTAGATAGCTTGCCCCGCAAGCAAACTAAAAGCCTATATATACCAGTGTAGACATATTGTTCAGATAACAAATGGCGAGGTTTCCGAAGAAATCGATCAA
GAAGCGGCGGGTTGGGCGACGGAAGTATGGAAGCAAGGCGGCAACCAGTCACGACTACTCGTCGTTAGGTTCAATATTGGTTCCTGAAAATACCGTCAAGGTATTTAGGATTGAGCCTAC
TGATAAAACATTACCCAGATATTTTATCTGGAAAATGTTTATGCTTTTGGTGTGCAAGGTGAAGCCCGGAAGAATACTTCACTGGGCTATGATTAAGAGTTCATGGGAAATCAACCAGCC
GACTACATGTCTGGAAGCACCAGGTTTATTTATAAAACCTGAACATAGCCATCTGGTGAAACTGGTATGCAGTGGGGAACTTGAAGCCGGAGTCGCAACAGGGACATCAGATGTTGAATG
TCTTCTAAGGAAGACAACCGTGTTGAGGAAGAATGTAACAGAGGTGGATTACTTGTATTTGGCATTTTATTGTAGTGCTGGAGTTAGTATTAACTACCAGAACAGAATTACATATCATGT
ATGATATGTTTATGTAAACATAAACCTTTGTATGGAATAATGACCAAATAACATACAACAcACTATGAAATACAACACGCTATGACAAACAGGGAAAAATGAAgAATCGGGgGTTGATTG
GTCTATCGTAaCGCTTAAGGGCCGCAGGCCCGTTGAAATGATTCTTTATAAAACAAATATACATGATACGGATaGTTGAAcATATAAaCAACGATGTATAAATACAACAGAATGTTGTAT
ACTAATTAAAAATAATGAGAAGAAAAGAATATTTGTGAAGGATAAGCATCAGAACCACCACTTTAGTGGTGGGTCAGATGTCCCGAGTTAGTGCGCCACGTAAGCGCTGGGGCTTAT

DNA-U3
Aligned_sequences: 2
1: U3_alyu41
2: U3_alyu42
Length: 1079
Identity: 988/1079 (91.6%)
Similarity: 988/1079 (91.6%)
Gaps: 49/1079 (4.5%)

39 aa protein-encoding ORF is preserved

>U3_alyu41
TATTACCCCCAGCCCCCAGGACGGGACATGGGCTTTTTAAATGGGCCGAGAGAGTTTGAACAGTTCAGTATCTTCGTTATTGGGCCATCTGGCCCAATAATTAAGAGAACGTGTTCAAAA
TCtGGGTTTGACCGAAGGTCAAGGTAGACGGTCAACAATATTCTGGCTTGCGGAGCAAGCAACAAGAATTAAATATTTAATTCGTATGACACGTGGACGGACCGAAATACTCCTGTATCT
CTATAAATAGCCTAAATCTGGCATGGATAATTGCTCTCGCTCTTCTGTCAAAGCTGTTGTGTTGAGGCGGAAGATCGCCATCGGCGATCATCAGACGAAAAGCTGCAAGAGAGACGGAGA
ACCATGCTGCGAAGCGTATATCGGGTATTTATAGACTTCTAGCGCAGCTAGAAGTTTCCTTGTACTTGTATTTGTATTTTGTAAATTACGAAGAAATTCGTACATTGATAATAATAAAAC
ATCTGGGATTGTTAATGTTTACATTAACTAGTATTAATAATGTACAATAACAAACATTGTATGAGGAACGAATACAAGAATATTAAAATATGAGGAGCGTAGCGTGATAAACAGGTGTTT
AAGGAATAATTAAAATAATTATGCAATGTTATGATAATACGGTATAAGTTGAAGTATGAGGTGAAAGAGGAGATATTAGAATATTAAAAACCCAATTATATTATTTATAATACATACAAC
ACGCTATGACAAAAGGGGAAAAAAGAAAAATCGGGGGTTGATTGGTCTATCCTAACGATTAAGGGCCGCAGGCCCGTCAAGATGGATCCAATAACCCGTTAAGAAGGTAAACGGGTCTAA
AACGATTTCTTCGCCCGCAAGCAACACCTTTAACCTCTGCGCACCTATATATATAATATAATAACCAAATGACCATAGTGCCCCTCCCAAAGATGACGTATAGGGGTGTCCCGAGGTAAT
TTAACATAGCTCTGATAAGAGATAGTGGACGTTGGATTCCACGATCGGACGATCGTGGTTTGATGAACTTATGATGACGTAGGGGCTGGGGCTTAT

ALYU-42 - Musa AAA Cavendish - Laos

Virome
1. **BBTV** six components
2. **BBTA5** alyu42

BBTA5
Best matches to MF510475, MF510474 73% coverage 66.4% identity Faba bean necrotic yellows virus associated alphasatellite 2; AJ132187 AJ005966 73% coverage 66.2-65.7% identity Faba bean necrotic yellows C9 alphasatellite; MK291270 72% coverage 67.0% identity Sophora yellow stunt alphasatellite 3

Rep protein best matches to QBF29185 100% coverage 63% identities 80% positives Sophora yellow stunt alphasatellite 3; ATU31571 100% coverage 620% identities 80% positives Faba bean necrotic yellows virus associated alphasatellite 2; ARI50297 100% coverage 62% identities, 80% positives Sophora alopecuroides yellow stunt alphasatellite 3

>BBTA5_alyu42
TATTACCCGACCTTGGCTCACTTGCTCACTATAAAAGGGGGAGATGTCGCAGCAGCGAAATTGGGTATTCACGCGTAACTTCTCCGGCGACCCGCCGATTTTAAAGTTCGCCGGAGAAAC
TCAATACGCGTGCTGGCAACATGAGAAGAAGAATCACGACCATCTTCAGGGAGTGATTCAATTGAAGAAGAAGCTTCGGATGAACGCAGTGAAGACGTTAATTGGGGGAAATCCACACCT
CGAAGCCATGAGAGGAACAATCGACGAAGCAATAAGGTACGTGACGAAAGAAGAGACACGAGTAGCAGGTCCGTGGGAATTCGGAGAATTACTACGAAAAGGATCTCATAAGAGGAAATT
GATGGAATTGCTCGACGATCCAGATAACGAAATAATGGAACCTCAAAAATATCGACGCGCCATTACTAAACAAGCAATGGACGCGTCAAAGAAGAAGGCGGAATTAGGGTTTCCTTACGA
CTTAAAGGAGTGGCAAAAGATGGTTATTGAGTTAATCGAAGAACAACCCGACAACAGAACTATTATCTGGGTGTATGGTCCTAATGGTGGAGAAGGTAAAACGCAGTTCGCAAAGCATTA
TGGATTACTCAAAGGATGGACATATCTACCAGGTGGTGAACTGAAAGACATGATGTATCTGTTGGCAAAAGAAATTAATAATAACGTTATTATTGATTTCCCCCGATGTACAAAGGATTT
CATATCCTATAAGTTTATTGAGATGGTAAAGAACCGATGCATATTTAGTTATAAATATGAACCAATCGGGGCGATTGTAAGTAATGAAGTCCATGTAATTGTAATGTCGAATGAGCTTCC
AGATTATTCAAAGATTTCGGAAGACAGAATAAAATTAATATTTACATAACGCACACTATGACAAAAGGGCAAAAATGAAAAATCGGGGGTTGATTTGTCTATATTTACGATTAAGGGCCG
CAGGCCCGTACACTTTGCGCGGGAAATTCAAAATCCGGACGCCAAATTTTATTAAATACAATACACCGCCTTTTAGAAATCCGTGGACGAGAAGATGCCACGTATCAAAACACTGTACTC
CTTGGCTCACTATAAATACCTGAGCCAAGGTCGGGCATAG

DNA-C
7-15 SNPs to KY427060 MF039864 MF039876 MF039870 (**Thailand**). **31 SNPs** to KM607098 KM607099 (**China**)

C protein (161 aa): 1 aa to KY427060 (**Thailand**) **2 aa** to KM607098 KM607099 (**China**) MF039864 (**Thailand**)

>C_alyu42
TATTACCCCCCCTGCTCGGGACGGGACATCACGTGCATCTAACAAATGCACGTGACAATGCAGTAGCTTGTAGCGAAAGATAACCATCACTATCATAAAAGTGAAAGGAATATTCGTTGC
TTAGGGACTAAGCAACGTGAACAGATATTTGTTCGAGAAGCGACAATGGAGGCTATTTAAACCTGATGGTTTTGTGATTTCCGAAATCACTCATCGGAGAAGAAATGGAGTTCTGGGAAT
CGTCTGCCATGCCAGACGATGTCAAGAGAGTGATCAAGGAAATATATTGGGAGCATCGAAACAAACTTCTGTTTTGTCAGAAGTTGAAAGGCTGTGTAAGAAGGATTCTGGAGCATGGAA
ATCAAGATGATGCTCTAGACGCAGTGAAGGAGATGAAGACTTCTATTATTCGCTATAGCGAATATTTGAAGAAACCTTGTGTGGTATTGTGTTTTAGTACTAATAAATCTATTGCATATA
GGTTAAACACAATGGTGTTCTTTTATCATGAATATATTGAAGACCTAGGTGGTGACTACTCGGTATATCAAGATTTGTTTTGTGATGAGGAACTTCCTTATTCGTCGACAGAAGAAGAAG
ATGAAGAAGTAATATACAGGAATGTTATCATGGCATCGACAGAAGAGAAGATCTCTTGGAGTGAATGTCAAAAGATAGTTATATCAGATTATGATGTAACATTACTGTAATGAATATCCA
TTATGATAAATAAAATAATGGAATGATGATTATGTATTCTAGTATAAATACATAATGGTATACGTATAGCATAAAATACATATACCTACATACAACACACTATAACAAACAGGGAAAAAT
GAAGAATCGGGGGTTGATTGGGCTATCTTAACGATTAAGGGCCGCAGGCCCGTTTAAATATGTGGTGGACGAAGTCCACAACACATAAAAAGGTATCAGAACAATGGAATATAATGAGCT
GGCAACGTAGGGACCATGTCCCGAGTTAGTGCGCCACGTGAGCAGGGGGGCTTAT

DNA-M
6-10 SNPs to MF039865 KY427061 MF039871 MF039876 (**Thailand**). **52-59 SNPs** to KM607239 KM607240 (**China**)

M protein (117 aa): 0 aa to KC581796 (**Thailand**) **1 aa** to MF039871 MF039865 MF039877 KY427061 (**Thailand**)

>M_alyu42

TATTACCCCCAGCGCTCAGGACGGGACATCACGTGCAACTAACAAATGCACGTGACTGATATATACTACATAACGGTTTATTGAACCGTTATATATTGTTGTAACGCAAAGTCACGTGTG
ATAGCGAAATGCACGTGACCAAGTCAAATGTATTGAATAAACATTTGACGTCCGGATGCTTCCTACGGAAGCCATGGTTGCTTCGTGGCGAAGCAAAACATTTATATATTGGCTTGGATT
GCTGCCTATAAATAGGCACGCAGGGAAATGGCATTAACAACAGAGCGAGTGAAACTATTCTTTGAATGGTTTCTGTTTATTGGTGCAATATTTATTGCGATAACAATATTATACATATTG
TTGGCGTTGCTCTTTGAGGTCCCCAAGTATATTAAGGATGTTGTGAGGTATCTCGTAGAATACCTGACCAGACGACGTGTATGGATGCAGAGAACGCAGTTGACGGAGGCAACAGGAGAT
GTAGAACTCGGCAGAGGTAGTGTGGAAGACAGACGGGATCAACAACCGGCTGTCATACCACATGCAACACAGGTTATCCCTTCGCATCAACCTAGAAGGGATGAGCAAGGAAGACGAGGA
AACGCCGGACCGATGTTTTAATACACGGTATTGTAATATACGAAATATAAATGGATAATGATAAGTATTGTGAAACAAAATATATGTGAAACATAATATATGTTTGATAATTTACATATT
GTAATATGTGAATTGTATACGAGTGTTGTATTTATAAAACATACAACACGCTATGACAAACAGGGAAAAATGAAGAATCGGGGGTTGATTGGTCTATCGTATCGCTTAAGGGCCGCAGGC
CCGTTGAAATGATTCTTTATAAAACAAATATACATGATACGGATTGTTGAAAATATAATCAACGATGTATAAATACAACAGAATGTTGTATAATAATTAAAATAATGAGAAGATAAGTAT
ATTTGTGAAGGATATGGATCACAACCACCACTTTAGTGGTGGGTCATATGTCCCGAGTTAGTGCGCCACGTAAGCGCTGGGGCTTAT

DNA-N
2-6 SNPs to MF039878 MF039866 KY427062 MF039866 (**Thailand**). **24-34 SNPs** to KM607387 KM607385 KM607386 (**China**)

N protein (154 aa): 0 aa to MF039872 MF039878 KY427062 (**Thailand**) KM607387 KM607385 (**China**) **1 aa** to MF039866 (**Thailand**)

>N_alyu42
TATTACCCCCCGTGCTCGGGACGGGACATGACGTCAGCATAGATTATAATGGGCCAACTAAAGGCCCATTTAATAGAATAGGCGGGCTTTTGACATATTTCAAAGGCCCAGCCTGGAAGT
GGATAATGTCACGTGCCGAATAAGAGGTTGCTTCGCCTCGAAGCAACCCGAACAAATGTTGCGTATTCAATACGCAACTGAAGTCTATTAATATGGATGTCTCTGCCGAATAAATCAGAG
CGTAAGCGAAGCAGAAGCGATGGATTGGGCAGAATCACAATTCAAGACATGTACTCATGGGTGCGATTGGAAGACGATATCATCGGATTCATCCGAGAATCGACAATATGTACCTTGCGT
CGACGCAGGCAGCGGAAGAAAGTCACCTCGCAAGGTACTGCTAAGATCTATTGAAGCTGTGTTTAATGGAAGCTTCAAAGGAAATAACAGGAATGTTCGTGGGTTTATATACGTATCAAT
ACGAGACGATGATGGAGAAATGCGTCCAGTACTCATAATACCATTCGGAGGATATGGATATCATAATGATTTCTATTATTTCGAAGGGAAGGGGAAAGTTGAATGTGATATATCATCAGA
TTATGTAGCGCCAGGAGTCGATTGGAGCAGAGACATGGAAGTTAGTATTAGTAACAGCAACAACTGTAATGAATTATGTGATCTGAAGTGTTATGTTGTTTGTTCGTTAAGAATAAAGGA
ATAACAGATGTGCTGTAATGAATATTAATAAAATATATTTTCATGTAATTGATAGTTGTATAAAACATACAACACGCTATGACAAACAGGGAAaAATGAAAAATCAGGGGTTGATTGGTC
TATCGTATCGCTTAAGGGCCGCAGGCCCGTTGAAAAATAATAATCGAATTATTAACGTTTGATAATAATCAGAGATAGATGATAAGCATCCAAAAAACATAGACGAAGTATATGGCTGTA
TAATAAAAACAAGCATATAAATAAAATATGTGAACTAATCTCTGATTGGTTTACAACGTAGCCCCACCAACTTTATGTTGGTGGAAATGTCCCGATGACGTAAGCACGGGGGACTAT

DNA-R
3-6 SNPs to MF039879 MF039873 MF039867 KY427063 (**Thailand**). **10 SNPs** to AF416475 (**Viet Nam**). **14-16 SNPs** to KM607677 KM607678 KM607676 KM607679 (**China**)

R protein (286 aa): 0 aa to KM60767 KM607677 KM607679 (**China**) **1 aa to** AB113660 (**Vietnam**) MF039879 (**Thailand**) KM607678 (**China**) …

>R_alyu42
TATTACCCCCAGCGCTCGGGACGGGACATTTGCATCTATAAATAGACCTCCCCCCCCTCCACTACAAGATCATCATCGTCGACAGAAATGGCGCGATATGTGGTATGCTGGATGTTCACC
ATCAACAATCCCGCTTCACTACCAGTGATGCGGGATGAGTTTAAATATATGGTATATCAAGTGGAGAGGGGACAGGAGGGTACTCGTCATGTGCAGGGATACGTCGAGATGAAGAGACGA
AGTTCTCTGAAGCAGATGAGAGGCTTCTTCCCTGGCGCACACCTTGAGAAACGAAAGGGAAGCCAAGAAGAAGCACGGGCATACTGTATGAAGGAAGACACAAGAATCGAAGGTCCCTTC
GAGTTTGGTGCCTTTAAATTGTCATGTAATGATAATTTATTTGATGTCATACAGGATATGCGTGAAACGCACAAACGGCCTTTGGAGTATTTATATGAGTGTCCAAATACCTTCGATAGA
AGTAAGGATACATTATACAGAGTTCAAGCAGAGTTGAATAAAACGAAGGCGATGAATAGCTGGAAAACATCCTTCAGTTCTTGGACATCGGAAGTTGAAAATATTATGGCGGAGCCATGT
CATCGAAGGATAATTTGGGTCTATGGCCCAAATGGAGGAGAAGGAAAGACAACTTATGCAAAATATTTAATGAAGACGAAGAATGCGTTTTATTCGCCAGGAGGAAAATCTTTGGATATA
TGTAGATTGTATAATTATGAAGAAATAGTTATATTTGATATTCCCAGATGCAAAGAGGAATATTTAAACTATGGTTTATTAGAAGAATTTAAAAATGGAATTATTCAAAGCGGGAAATAT
GAACCCGTTTTGAAAATTGTAGAATATGTGGAAGTCATTGTAATGGCTAACTTCCTTCCGAAGGAAGGAATCTTCTCTGAAGATCGAATAAAGCTAGTTGCTTGCTGAACACGCTATGAC
AATCGTACGCTATGACAAAAGGGGAAAAGCAAAGAATCGGGGGTTGATTGGGCTATCCTAACGATTAAGGGCCGCAGGCCCGTCAAGATGGACGGTTTGATCAGATGTCCCGAGTTAGTG
CGCCACGTAAGCGCTGGGGCTTAT

DNA-S
6-20 SNPs to MF039874 KY427064 MF039880 (**Thailand**). **24-27 SNPs** to AB113661 AB113662 (**Viet Nam**). **27-29 SNPs** to KM60753 KM607534 KM607536 KM607533 (**China**)

S protein (170 aa): 0 aa to AY337715 AY494786 HQ378191 (**China**) FJ787433 FJ787434 (**Philippines**), **1 aa** to several isolates

>S_alyu42
TATTACCCCCAGCGCTCGGGACGGGACATGGGCTAATGGATCATGGAATAAGGGCCCATCGGGCCCGTTAAGATGGGTTTTGGGCTTATGGGCTTTATCCAGAAGACCAAAAACAGGCGG
GAACCGTCCCAATTTCAAATTTAGATAGCTTGCCCCGCAAGCAAACTAAAAGTCTATATATACCAGTGTAGACATATTGTTCAGATAACAAATGGCGAGGTTTCCGAAGAAATCGATCAA
GAAGAGGCGGGTTGGGCGACGGAAGTATGGAAGCAAGGCGGCAACCAGTCACGACTACTCGTCGTTAGGTTCAATATTGGTTCCTGAAAATACCGTCAAGGTATTTAGGATTGAGCCTAC
TGATAAAACATTACCCAGATATTTTATCTGGAAAATGTTTATGCTTTTGGTGTGCAAGGTGAAACCTGGAAGAATACTTCATTGGGCTATGATTAAAAGTTCATGGGAAATCAACCAGCC
GACTACATGTCTGGAAGCACCAGGTTTATTCATAAAACCTGAACATAGCCATCTGGTGAAACTGGTATGCAGTGGGGAACTTGAAGCCGGAGTCGCAACAGGGACATCAGATGTAGAATG
TCTTCTAAGGAAGACAACCGTGTTGAGGAAGAATGTAACAGAGGTGGATTACTTGTATTTGGCATTTTATTGTAGTGCTGGAGTTAGTATTAACTACCAGAACAGAATTACATATCATGT
ATGATCTGTTTATGTAAACATAAACCATTGTATGGATTAATGACCAAATAACATACAACACGCTATGAAATACAACACGCTATGACAAACGGGGAAAAATGAAGAATCGGGGGTTGATTG
GTCTATCGTATCGCTTAAGGGCCGCAGGCCCGTTGAAATGATTCTTTATAAAACAAATATACATGATACGGATTgTTGAAAATATAATCAACGATGTATAAATACAACAGAATGTTGTAT
ACAAATTAAAAAAAAAGAGAAGATAAGTATATTTGTGAAGGATAAGCATCAGAACCACCACTTTAGTGGTGGGTCAGATGTCCCGAGTTAGTGCGCCACGTAAGCGCTGGGGCTTAT

DNA-U3
11-21 SNPs to MF039881 MF039875 KY427065 MF039869 (**Thailand**)

U3 protein (39 aa): 0 aa to KY427065 (**Thailand)**

>U3_alyu42
TATTACCCCCAGCCCCCAGGACGGGACATGGGCTTTTTAAATGGGCCGAGAGAGTTTGAACAGTTCAGTATCTTCGTTATTGGGCCATCTGGCCCAATAATTAAGAGAACGTGTTCAAAA
TCGGGGTTTGACCGAAGGTCAAGGTAGACGGTCAACAATATTCTGGCTTGCGGAGCAAGCAACACGAATTAAATATTTAATTCGTATGACACGTGGACGGACCGAAATACTCCTGTAAAT
CTATAAATAGCCTAAATCTGGCCTGGATAATTGCTCTCGCTCTTCTGTCAAAGCTGTTGTGTTGAGGCGGAAGATCGCCATCGGCGATCATCAGACGAAAAGCTGCAAGAGAGACGGAGA
AACATGCTGCGAAGCGTATATCGGGTATTTATAGACTTCTAGCGCAGCTAGAAGTTTCGTTGTATTTGATATTGTATTTTGTAAATTACGAAGAATTCGTACATTGATAATAATAAAACA
TCTGGGATTGTTAATGTTTACATTAACCAGTATTAATAATGTACAATAAAAAAAATTGTATGAGGATCGAATACAATAATATTAAAATATGAGGAGCGTAGCATGATAAACAGGTGTTTA
AGGTATAATTAAAAAAATTATGCAATGTTATGATAATACGGTATAAGTTGAAGTATGAGGTGAAAGAGGAGATATTAGAATATTAAAAACCCAATTATATTATTTATAATACATACAACA
CGCTATGACAAAAGGGGAAAAATGAAGAATCGGGGGTTGATTGGTCTATCGTATCGCTTAAGGGCCGCAGGCCCGTCAAGATGGATGAACGGTCAGATTTGATTGCTTAGCCACGAAGAA
AGGAATCTTTTTGGGACCACAGATAAGACAGCTGTCATTACCTTTTAAAATAATATAATAACCAATTGACGATAGTACCCCTCCCAAAGATGACGTATAGGGGTGTCCCGAGGTAATTTA
ACATAGCTCTGACAAGAGATAGTGGACGTTGGATGCCACGATCGGACGATCGTGGTTTGATGAACTTATGATGACGTAGGGGCTGGGGCTTAT

ALYU-43 - Musa AAA Cavendish - Laos

Virome
1. **BBTV** six components (all except N are represented with 2 genetic variants)
2. **BBTA5** alyu43

BBTA5 v1 - 1 SNP to alyu42 / v2 – 2 SNPs to alyu42

In both variants, Rep protein is identical to alyu42

>BBTA5_alyu43_v1
TATTACCCGACCTTGGCTCACTTGCTCACTATAAAAGGGGGAGATGTCGCAGCAGCGAAATTGGGTATTCACGCGTAACTTCTCCGGCGACCCGCCGATTTTAAAGTTCGCCGGAGAAAC
TCAATACGCGTGCTGGCAACATGAGAAGAAGAATCACGACCATCTTCAGGGAGTGATTCAATTGAAGAAGAAGCTTCGGATGAACGCAGTGAAGACGTTAATTGGGGAAATCCACACCT
CGAAGCCATGAGAGGAACAATCGACGAAGCAATAAGGTACGTGACGAAAGAAGAGACACGAGTAGCAGGTCCGTGGGAATTCGGAGAATTACTACGAAAAGGATCTCATAAGAGGAAATT
GATGGAATTGCTCGACGATCCAGATAACGAAATAATGGAACCTCAAAAATATCGACGCGCCATTACTAAACAAGCAATGGACGCGTCAAAGAAGAAGGCGGAATTAGGGTTTCCTTACGA
CTTAAAGGAGTGGCAAAAGATGGTTATTGAGTTAATCGAAGAACAACCCGACAACAGAACTATTATCTGGGTGTATGGTCCTAATGGTGGAGAAGGTAAAACGCAGTTCGCAAAGCATTA

```
TGGATTACTCAAAGGATGGACATATCTACCAGGTGGTGAACTGAAAGACATGATGTATCTGTTGGCAAAAGAAATTAATAATAACGTTATTATTGATTTCCCCCGATGTACAAAGGATTT
CATATCCTATAAGTTTATTGAGATGGTAAAGAACCGATGCATATTTAGTTATAAATATGAACCAATCGGGGCGATTGTAAGTAATGAAGTCCATGTAATTGTAATGTCGAATGAGCTTCC
AGATTATTCAAAAATTTCGGAAGACAGAATAAAATTAATATTTACATAACGCACACTATGACAAAAGGGCAAAAATGAAAAATCGGGGGTTGATTTGTCTATATTTACGATTAAGGGCCG
CAGGCCCGTACACTTTGCGCGGGAAATTCAAAATTCGGACGCCAAATTTTATTAAATACAATACACCGCCTTTTAGAAATCCGTGGACGAGAAGATGCCACGTATCAAAACACTGTACTC
CTTGGCTCACTATAAATACCTGAGCCAAGGTCGGGCATAG
```

G/C (64895/29398) A/G (111791/14216) T/C (99589/70722)

```
>BBTA5_alyu43_v2
TATTACCCGACCTTGGCTCACTTGCTCACTATAAAAGGcGGAGATGTCGCAGCAGCGAAATTGGGTATTCACGCGTAACTTCTCCGGCGACCCGCCGATTTTAAAGTTCGCCGGAGAAAC
TCAATACGCGTGCTGGCAACATGAGAAGAAGAATCACGACCATCTTCAGGGAGTGATTCAATTGAAGAAGAAGCTTCGGATGAACGCAGTGAAGACGTTAATTGGGGAAATCCACACCT
CGAAGCCATGAGAGGAACAATCGACGAAGCAATAAGGTACGTGACGAAAGAAGAGACACGAGTAGCAGGTCCGTGGGAATTCGGAGAATTACTACGAAAAGGATCTCATAAGAGGAAATT
GATGGAATTGCTCGACGATCCAGATAACGAAATAATGGAACCTCAAAAATATCGACGCGCCATTACTAAACAAGCAATGGACGCGTCAAAGAAGAAGGCGGAATTAGGGTTTCCTTACGA
CTTAAAGGAGTGGCAAAAGATGGTTATTGAGTTAATCGAAGAACAACCCGACAACAGAACTATTATCTGGGTGTATGGTCCTAATGGTGGAGAAGGTAAAACGCAGTTCGCAAAGCATTA
TGGATTACTCAAAGGATGGACATATCTACCAGGTGGTGAACTGAAAGACATGATGTATCTGTTGGCAAAAGAAATTAATAATAACGTTATTATTGATTTCCCCCGATGTACAAAGGATTT
CATATCCTATAAGTTTATTGAGATGGTAAAGAACCGATGCATATTTAGTTATAAATATGAACCAATCGGGGCGATTGTAAGTAATGAAGTCCATGTAATTGTAATGTCGAATGAGCTTCC
AGATTATTCAAAgATTTCGGAAGACAGAATAAAATTAATATTTACATAACGCACACTATGACAAAAGGGCAAAAATGAAAAATCGGGGGTTGATTTGTCTATATTTACGATTAAGGGCCG
CAGGCCCGTACACTTTGCGCGGGAAATTCAAAATcCGGACGCCAAATTTTATTAAATACAATACACCGCCTTTTAGAAATCCGTGGACGAGAAGATGCCACGTATCAAAACACTGTACTC
CTTGGCTCACTATAAATACCTGAGCCAAGGTCGGGCATAG
```

DNA-C v1 = alyu42

Clink protein is identical in v1 and v2

```
>C_alyu43_v1
TATTACCCCCCCTGCTCGGGACGGGACATCACGTGCATCTAACAAATGCACGTGACAATGCAGTAGCTTGTAGCGAAAGATAACCATCACTATCATAAAAGTGAAAGGAATATTCGTTGC
TTAGGGACTAAGCAACGTGaACAGATATTTGTTCGAGAAGCGACAATGGAGGCTATTTAAACCTGAtGGTTTTGTGATTTCCGAAATCACTCATCGGAGAAGAAATGGAGTTCTGGGAAT
CGTCTGCCATGCCAGACGATGTCAAGAGAGTGATCAAGGAAATATATTGGGAGCATCGAAACAAACTTCTGTTTTGTCAGAAGTTGAAAGGCTGTGTAAGAAGGATTCTGGAGCATGGAA
ATCAAGATGATGCTCTAGACGCAGTGAAGGAGATGAAGACTTCTATTATTCGCTATAGCGAATATTTGAAGAAACCTTGTGTGGTATTGTGTTTTAGTACTAATAAATCTATTGCATATA
GGTTAAACACAATGGTGTTCTTTTATCATGAATATATTGAAGACCTAGGTGGTGACTACTCGGTATATCAAGATTTGTTtTGTGATGAGGAACTTCCTTATTCGTCGACAGAAGAAGAAG
ATGAAGAAGTAATATACAGGAATGTTATCATGGCATCGACAGAAGAGAAGATCTCTTGGAGTGAATGTCAAAAGATAGTTATATCAGATTATGATGTAACATTACTGTAATGAATATCCA
TTATGATAAATAAAATAATGGAATGATGATTATGTATTCTAGTATAAATACATAATGGTATACGTATAGCATAAAATACATATACCTACATACAACACACTATAACAAACAGGGAAAAAT
GAAGAATCGGGGGTTGATTGGGCTATCTTAACGATTAAGGGCCGCAGGCCCGTTTAAATATGTGGTGGACGAAGTCCACAACACATAAAAAGGTATCAGAACAATGGAATATAATGAGCT
GGCAACGTAGGGACCATGTCCCGAGTTAGTGCGCCACGTGAGCAGGGGGGCTTAT
```

```
>C_alyu43_v2
TATTACCCCCCCTGCTCGGGACGGGACATCACGTGCATCTAACAAATGCACGTGACAATGCAGTAGCTTGTAGCGAAAGATAACCATCACTATCATAAAAGTGAAAGGAATATTCGTTGC
TTAGGGACTAAGCAACGTGGACAGATATTTGTTCGAGAAGCGACAATGGAGGCTATTTAAACCTGAAGGTTTTGTGATTTCCGAAATCACTCATCGGAGAAGAAATGGAGTTCTGGGAAT
CGTCTGCCATGCCAGACGATGTCAAGAGAGTGATCAAGGAAATATATTGGGAGCATCGAAACAAACTTCTGTTTTGTCAGAAGTTGAAAGGCTGTGTAAGAAGGATTCTGGAGCATGGAA
ATCAAGATGATGCTCTAGACGCAGTGAAGGAGATGAAGACTTCTATTATTCGCTATAGCGAATATTTGAAGAAACCTTGTGTGGTATTGTGTTTTAGTACTAATAAATCTATTGCATATA
GGTTAAACACAATGGTGTTCTTTTATCATGAATATATTGAAGACCTAGGTGGTGACTACTCGGTATATCAAGATTTGTTTTGTGATGAGGAACTTCCTTATTCGTCGACAGAAGAAGAAG
ATGAAGAAGTAATATACAGGAATGTTATCATGGCATCGACAGAAGAGAAGATCTCTTGGAGTGAATGTCAAAAGATAGTTATATCAGATTATGATGTAACATTACTGTAATGAATATCCA
TTATGATAAATAAAATAATGGAATGATGATTATGTATTCTAGTATAAATACATAATGGTATACGTATAGCATAAAATACATATACCTACATACAACACACTATAACAAACAGGGAAAAAT
GAAGAATCGGGGGTTGATTGGGCTATCTTAACGATTAAGGGCCGCAGGCCCGTTTAAATATGTGGTGGACGAAGTCCACAACACATAAAAAGTGATCAGAACAATGGAATATAATGAGCT
GGCAACGTAGGGACCATGTCCCGAGTTAGTGCGCCACGTGAGCAGGGGGGCTTAT
```

DNA-M v1 = alyu42

MP v1=v2

```
>M_alyu43_v1
TATTACCCCCAGCGCTCAGGACGGGACATCACGTGCAACTAACAAATGCACGTGACTGATATATACTACATAACGGTTTATTGAACCGTTATATATTGTTGTAACGCAAAGTCACGTGTG
ATAGCGAAATGCACGTGACCAAGTCAAATGTATTGAATAAACATTTGACGTCCGGAtGCTTCCTACGGAAGCCATGGTTGCTTCGTGGCGAAGCAAAACATTTATATATTGGCTTGGATT
GCTGCCTATAAATAGGCACGCAGGGAAATGGCATTAACAACAGAGCGAGTGAAACTATTCTTTGAATGGTTTCTGTTTATTGGTGCAATATTTATTGCGATAACAATATTATACATATTG
TTGGCGTTGCTCTTTGAGGTCCCCAAGTATATTAAGGATGTTGTGAGGTATCTCGTAGAATACCTGACCAGACGACGTGTATGGATGCAGAGAACGCAGTTGACGGAGGCAACAGGAGAT
GTAGAACTCGGCAGAGGTAGTGTGGAAGACAGACGGGATCAACAACCGGCTGTCATACCACATGCAACACAGGTTATCCCTTCGCATCAACCTAGAAGGGATGAGCAAGGAAGACGAGGA
AACGCCGGACCGATGTTTTAATACACGGTATTGTAATATACGAAATATAAATGGATAATGATAAGTATTGTGAAACAAAATATATGTGAAACATAATATATGTTTGATAATTTACATATT
GTAATATGTGAATTGTATACGAGTGTTGTATTTATAAAACATACAACACGCTATGACAAACAGGGAAAAATGAAGAATCGGGGGTTGATTGGTCTATCGTATCGCTTAAGGGCCGCAGGC
CCGTTGAAATGATTCTTTATAAAACAAATATACATGATACGGATTGTTGAAAATATAATCAACGATGTATAAATACAACAGAATGTTGTATAatAATTAAAAtAAtGAGAAGATGAGAAG
ATAAGTATATTTGTGAAGGATATGGATCACAACCACCACTTTAGTGGTGGGTCATATGTCCCGAGTTAGTGCGCCACGTAAGCGCTGGGGCTTAT
```

```
>M_alyu43_v2
TATTACCCCCAGCGCTCAGGACGGGACATCACGTGCAACTAACAAATGCACGTGACTGATATATACTACATAACGGTTTATTGAACCGTTATATATTGTTGTAACGCAAAGTCACGTGTG
ATAGCGAAATGCACGTGACCAAGTCAAATGTATTGAATAAACATTTGACGTCCGGAaGCTTCCTACGGAAGCCATGGTTGCTTCGTGGCGAAGCAAAACATTTATATATTGGCTTGGATT
GCTGCCTATAAATAGGCACGCAGGGAAATGGCATTAACAACAGAGCGAGTGAAACTATTCTTTGAATGGTTTCTGTTTATTGGTGCAATATTTATTGCGATAACAATATTATACATATTG
TTGGCGTTGCTCTTTGAGGTCCCCAAGTATATTAAGGATGTTGTGAGGTATCTCGTAGAATACCTGACCAGACGACGTGTATGGATGCAGAGAACGCAGTTGACGGAGGCAACAGGAGAT
GTAGAACTCGGCAGAGGTAGTGTGGAAGACAGACGGGATCAACAACCGGCTGTCATtCCACATGCAACACAGGTTATCCCTTCGCATCAACCTAGAAGGGATGAGCAAGGAAGACGAGGA
AACGCCGGACCGATGTTTTAATACACGGTATTGTAATATACGAAATATAAATGGATAATGATAAGTATTGTGAAACAAAATATATGTGAAACATAATATATGTTTGATAATTTACATATT
GTAATATGTGAATTGTATACGAGTGTTGTATTTATAAAACATACAACACGCTATGACAAACAGGGAAAAATGAAGAATCGGGGGTTGATTGGTCTATCGTATCGCTTAAGGGCCGCAGGC
CCGTTGAAATGATTCTTTATAAAACAAATATACATGATACGGATTGTTGAAAATATAATCAACGATGTATAAATACAACAGAATGTTGTATACCAATTAAAAAAAAaAaAAaATGAGAAG
ATAAGTATATTTGTGAAGGATATGGATCACAACCACCACTTTAGTGGTGGGTCATATGTCCCGAGTTAGTGCGCCACGTAAGCGCTGGGGCTTAT
```

DNA-N = alyu42

```
>N_alyu43
TATTACCCCCCGTGCTCGGGACGGGACATGACGTCAGCATAGATTATAATGGGCCAACTAAAGGCCCATTTAATAGAATAGGCGGGCTTTTGACATATTTCAAAGGCCCAGCCTGGAAGT
GGATAATGTCACGTGCCGAATAAGAGGTTGCTTCGCCTCGAAGCAACCCGAACAAATGTTGCGTATTCAATACGCAACTGAAGTCTATTAATATGGATGTCTCTGCCGAATAAATCAGAG
CGTAAGCGAAGCAGAAGCGATGGATTGGGCAGAATCACAATTCAAGACATGTACTCATGGGTGCGATTGGAAGACGATATCATCGGATTCATCCGAGAATCGACAATATGTACCTTGCGT
CGACGCAGGCAGCGGAAGAAAGTCACCTCGCAAGGTACTGCTAAGATCTATTGAAGCTGTGTTTAATGGAAGCTTCAAAGGAAATAACAGGAATGTTCGTGGGTTTATATACGTATCAAT
ACGAGACGATGATGGAGAAATGCGTCCAGTACTCATAATACCATTCGGAGGATATGGATATCATAATGATTTCTATTATTTCGAAGGGAAGGGGAAAGTTGAATGTGATATATCATCAGA
TTATGTAGCGCCAGGAGTCGATTGGAGCAGAGACATGGAAGTTAGTATTAGTAACAGCAACAACTGTAATGAATTATGTGATCTGAAGTGTTATGTTGTTTGTTCGTTAAGAATAAAGGA
ATAACAGATGTGCTGTAATGAATATTAATAAAATATATTTTCATGTAATTGATAGTTGTATAAAACATACAACACGCTATGACAAACAGGGAAAAATGAAAAATCAGGGGTTGATTGGTC
TATCGTATCGCTTAAGGGCCGCAGGCCCGTTGAAAAATAATAATCGAATTATTAACGTTTGATAATAATCAGAGATAGATGATAAGCATCCAAAAAACATAGACGAAGTATATGGCTGTA
TAATAAAAACAAGCATATAAATAAAATATGTGAACTAATCTCTGATTGGTTTACAACGTAGCCCCACCAACTTTATGTTGGTGGAAATGTCCCGATGACGTAAGCACGGGGGACTAT
```

DNA-R v2 - 1 SNPs to alyu42

Rep protein is identical

```
>R_alyu43_v1
TATTACCCCCAGCGCTCGGGACGGGACATTTGCATCTATAAATAGACCTCCCCCCCCTCCACTACAAGATCATCATCGTCGACAGAAATGGCGCGATATGTGGTATGCTGGATGTTCACC
ATCAACAATCCCGCTTCACTACCAGTGATGCGGGATGAGTTTAAATATATGGTATATCAAGTGGAGAGGGGACAGGAGGGTACTCGTCATGTGCAGGGATACGTCGAGATGAAGAGACGA
AGTTCTCTGAAGCAGATGAGAGGCTTCTTCCCTGGCGCACACCTTGAGAAACGAAAGGGAAGCCAAGAAGAAGCACGGGCATACTGTATGAAGGAAGACACAAGAATCGAAGGTCCCTTC
GAGTTTGGTGCATTTAAATTGTCATGTAATGATAATTTATTTGATGTCATACAGGATATGCGTGAAACGCACAAACGACCTTTGGAGTATTTATATGAGTGTCCAAATACCTTCGATAGA
AGTAAGGATACATTATACAGAGTTCAAGCAGAGTTGAATAAAACGAAGGCGATGAATAGCTGGAAAACATCCTTCAGTTCTTGGACATCGGAAGTTGAAAATATTATGGCGGAGCCATGT
```

CATCGAAGGATAATTTGGGTCTATGGCCCAAATGGAGGAGAAGGAAAGACAACTTATGCAAAATATTTAATGAAGACGAAGAATGCGTTTTATTCGCCAGGAGGAAAATCTTTGGATATA
TGTAGATTGTATAATTATGAAGaaatagTTATATTTGATATTCCCAGATGCAAAGAGGAATATTTAAACTATGGTTTATTAGAAGAATTTAAAAATGGAATTATTCAAAGCGGGAAATAT
GAACCCGTTTTGAAAATTGTAGAATATGTGGAAGTCATTGTAATGGCTAACTTCCTTCCGAAGGAAGGAATCTTCTCTGAAGATCGAATAAAGCTAGTTGCTTGCTGAACACGCTATGAC
AATCGTACGCTATGACAAAAGGGGAAAAGCAAAGATTCGGGGGTTGATTGTGCTATCCTAACGATTAAGGGCCGCAGGCCCGTCAAGATGGACGGTTTGATCAGATGTCCCGAGTTAGTG
CGCCACGTAAGCGCTGGGGCTTAT

A/C (9857/8782) **A/G** (10726/8560) **T/G** (7640/5914)

>R_alyu43_v2
TATTACCCCCAGCGCTCGGGACGGGACATTTGCATCTATAAATAGACCTCCCCCCCCTCCACTACAAGATCATCATCGTCGACAGAAATGGCGCGATATGTGGTATGCTGGATGTTCACC
ATCAACAATCCCGCTTCACTACCAGTGATGCGGGATGAGTTTAAATATATGGTATATCAAGTGGAGAGGGGACAGGAGGGTACTCGTCATGTGCAGGGATACGTCGAGATGAAGAGACGA
AGTTCTCTGAAGCAGATGAGAGGCTTCTTCCCTGGCGCACACCTTGAGAAACGAAAGGGAAGCCAAGAAGAAGCACGGGCATACTGTATGAAGGAAGACACAAGAATCGAAGGTCCCTTC
GAGTTTGGTGCcTTTAAATTGTCATGTAATGATAATTTATTTGATGTCATACAGGATATGCGTGAAACGCACAAACGgCCTTTGGAGTATTTATATGAGTGTCCAAATACCTTCGATAGA
AGTAAGGATACATTATACAGAGTTCAAGCAGAGTTGAATAAAACGAAGGCGATGAATAGCTGGAAAACATCCTTCAGTTCTTGGACATCGGAAGTTGAAAATATTATGGCGGAGCCATGT
CATCGAAGGATAATTTGGGTCTATGGCCCAAATGGAGGAGAAGGAAAGACAACTTATGCAAAATATTTAATGAAGACGAAGAATGCGTTTTATTCGCCAGGAGGAAAATCTTTGGATATA
TGTAGATTGTATAATTATGAAGAAATAGTTATATTTGATATTCCCAGATGCAAAGAGGAATATTTAAACTATGGTTTATTAGAAGAATTTAAAAATGGAATTATTCAAAGCGGGAAATAT
GAACCCGTTTTGAAAATTGTAGAATATGTGGAAGTCATTGTAATGGCTAACTTCCTTCCGAAGGAAGGAATCTTCTCTGAAGATCGAATAAAGCTAGTTGCTTGCTGAACACGCTATGAC
AATCGTACGCTATGACAAAAGGGGAAAAGCAAAGATTCGGGGGTTGATTGgGCTATCCTAACGATTAAGGGCCGCAGGCCCGTCAAGATGGACGGTTTGATCAGATGTCCCGAGTTAGTG
CGCCACGTAAGCGCTGGGGCTTAT

DNA-S v2 4 SNPs to alyu42

CP protein is identical

>S_alyu43_v1
TATTACCCCCAGCGCTCGGGACGGGACATGGGCTAATGGATCATGGAATAAGGGCCCATCGGGCCCGTTAAGATGGGTTTTGGGCTTATGGGCTTTATCCAGAAGACCAAAAACAGGCGG
GAACCGTCCCAATTTCAAATTTAGATAGCTTGCCCCGCAAGCAAACTAAAAGTCTATATATACCAGTGTAGACATATTGTTCAGATAACAAATGGCGAGGTTTCCGAAGAAATCGATCAA
GAAGAGGCGGGTTGGGCGACGGAAGTATGGAAGCAAGGCGGCAACCAGTCACGACTACTCGTCGTTAGGTTCAATATTGGTTCCTGAAAATACCGTCAAGGTATTTAGGATTGAGCCTAC
TGATAAAACATTACCCAGATATTTTATCTGGAAAATGTTTATGCTTTTGGTGTGCAAGGTGAAaCCTGGAAGAATACTTCATTGGGCTATGATTAAAAGTTCATGGGAAATCAACCAGCC
GACTACATGTCTGGAAGCACCAGGTTTATTCATAAAACCTGAACATAGCCATCTGGTGAAACTGGTATGCAGTGGGGAACTTGAAGCCGGAGTCGCAACAGGGACATCAGATGTAGAATG
TCTTCTAAGGAAGACAACCGTGTTGAGGAAGAATGTAACAGAGGTGGATTACTTGTATTTGGCATTTTATTGTAGTGCTGGAGTTAGTATTAACTACCAGAACAGAATTACATATCATGT
ATGATCTGTTTATGTAAACATAAACCTTTGTATGGCTTAATGACCAAATAACATACAACACGCTATGAAATACAACACGCTATGACAAACGGGGAAAAATGAAGAATCGGGGGTTGATTG
GTCTATCGTATCGCTTAAGGGCCGCAGGCCCGTTGAAATGATTCTTTATAAAACAAATATACATGATACGGATTGTTGAAAATATAATCAACGATGTATAAATACAACAGAATGTTGTAT
ACCAATTAAAAAAAAGAGAAGATAAGTATATTTGTGAAGGATAtGgATCAcAACCACCACTTTAGTGGTGGGTCATATGTCCCGAGTTAGTGCGCCACGTAAGCGCTGGGGCTTAT

C/T (1903/495) A/G (1352/1225) T/A (1165/923) C/A (1092/945) ins-G5723AG (3220/710) A/T (1654/2258) C/G (1253/2610) G/C (1282/2111)

>S_alyu43_v2
TATTACCCCCAGCGCTCGGGACGGGACATGGGCTAATGGATCATGGAATAAGGGCCCATCGGGCCCGTTAAGATGGGTTTTGGGCTTATGGGCTTTATCCAGAAGACCAAAAACAGGCGG
GAACCGTCCtAATTTCAAATTTAGATAGCTTGCCCCGCAAGCAAACTAAAAGTCTATATATACCAGTGTAGACATATTGTTCAGATAACAAATGGCGAGGTTTCCGAAGAAATCGATCAA
GAAGAGGCGGGTTGGGCGACGGAAGTATGGAAGCAAGGCGGCAACCAGTCACGACTACTCGTCGTTAGGTTCAATATTGGTTCCTGAAAATACCGTCAAGGTATTTAGGATTGAGCCTAC
TGATAAAACATTACCCAGATATTTTATCTGGAAAATGTTTATGCTTTTGGTGTGCAAGGTGAAgCCTGGAAGAATACTTCATTGGGCTATGATTAAAAGTTCATGGGAAATCAACCAGCC
GACTACATGTCTGGAAGCACCAGGTTTATTCATAAAACCTGAACATAGCCATCTGGTGAAACTGGTATGCAGTGGGGAACTTGAAGCCGGAGTCGCAACAGGGACATCAGATGTAGAATG
TCTTCTAAGGAAGACAACCGTGTTGAGGAAGAATGTAACAGAGGTGGATTACTTGTATTTGGCATTTTATTGTAGTGCTGGAGTTAGTATTAACTACCAGAACAGAATTACATATCATGT
ATGATCTGTTTATGTAAACATAAACCaTTGTATGGaTTAATGACCAAATAACATACAACACGCTATGAAATACAACACGCTATGACAAACGGGGAAAAATGAAGAATCGGGGGTTGATTG
GTCTATCGTATCGCTTAAGGGCCGCAGGCCCGTTGAAATGATTCTTTATAAAACAAATATACATGATACGGATTGTTGAAAATATAATCAACGATGTATAAATACAACAGAATGTTGTAT
ACCAATTAAAAAAAAaGAGAAGATAAGTATATTTGTGAAGGATAaGcATCAgAACCACCACTTTAGTGGTGGGTCATATGTCCCGAGTTAGTGCGCCACGTAAGCGCTGGGGCTTAT

DNA-U3 v1 = alyu42

ORF1 (39 aa) v1=v2

>U3_alyu43_v1
TATTACCCCCAGCCCCCAGGACGGGACATGGGCTTTTTAAATGGGCCGAGAGAGTTTGAACAGTTCAGTATCTTCGTTATTGGGCCATCTGGCCCAATAATTAAGAGAACGTGTTCAAAA
TCGGGGTTTGACCGAAGGTCAAGGTAGACGGTCAACAATATTCTGGCTTGCGGAGCAAGCAACACGAATTAAATATTTAATTCGTATGACACGTGGACGGACCGAAATACTCCTGTAAAT
CTATAAATAGCCTAAATCTGGCCTGGATAATTGCTCTCGCTCTTCTGTCAAAGCTGTTGTGTTGAGGCGGAAGATCGCCATCGGCGATCATCAGACGAAAAGCTGCAAGAGAGACGGAGA
AACATGCTGCGAAGCGTATATCGGGTATTtATAGACTTCTAGCGCAGCTAGAAGTTTCGTTGTATTTGATATTGTATTTTGTAAATTACGAAGAATTCGTACATTGATAATAATAAAACA
TCTGGGATTGTTAATGTTTACATTAACCAGTATTAATAATGTACAATAAAAAAAATTGTATGAGGATCGAATACAATAATATTAAAATATGAGGAGCGTAGCATGATAAACAGGTGTTTA
AGGTATAATTAAAAAAATTATGCAATGTTATGATAATACGGTATAAGTTGAAGTATGAGGTGAAAGAGGAGATATTAGAATATTAAAAACCCAATTATATTATTTATAATACATACAACA
CGCTATGACAAAAGGGGAAAAATGAAGAATCGGGGGTTGATTGGTCTATCGTATCGCTTAAGGGCCGCAGGCCCGTCAAGATGGATGAACGGTCAGATTTGATTGCTTAGCCACGAAGAA
AGGAATCTTTTTGGGACCACAGATAAGACAGCTGTCATTACCTTTTAAAATAATATAATAACCAATTGACGATAGTACCCCTCCCAAAGATGACGTATAGGGGTGTCCCGAGGTAATTTA
ACATAGCTCTGACAAGAGATAGTGGACGTTGGATGCCACGATCGGACGATCGTGGTTTGATGAACTTATGATGACGTAGGGGCTGGGGCTTAT

T/C (5206/18054) T/C (5922/19995) G/C (6323/21286) G/T (14217/4390) T/C (13764/4364) T/A (10443/3360) A/T (5860/1868) T/A (5939/2125)

>U3_alyu43_v2
TATTACCCCCAGCCCCCAGGACGGGACATGGGCTTTTTAAATGGGCCGAGAGAGTTTGAACAGTTCAGTATCTTCGTTATTGGGCCATCTGGCCCAATAATTAAGAGAACGTGTTCAAAA
TCGGGGTTTGACCGAAGGTCAAGGTAGACGGTCAACAATATTCTGGCTTGCGGAGCAAGCAACACGAATTAAATATTTAATTCGTATGACACGTGGACGGACCGAAATACTCCTGTAAAT
CTATAAATAGCTTAAATCTGGCTTGGATAATTGCTCTCGCTCTTCTGTCAAAGGTGTTGTGTTGAGGCGGAAGATCGCCATCGGCGATCATCAGACGAAAAGCTGCAAGAGAGACGGAGA
AACATGCTGCGAAGCGTATATCGGGTATTtATAGACTTCTAGCGCAGCTAGAAGTTTCtTTGTAcTTGATATTGTATTTTGTAAATTACGAAGAAaTCGTACATTGATAATAATAAAACA
TCTGGGATTGTTAATGTTTACATTAACCAGTATTAATAATGTACAATAAAAAAtATTGTATGAGGAaCGAATACAATAATATTAAAATATGAGGAGCGTAGCATGATAAACAGGTGTTTA
AGGTATAATTAAAAAAATTATGCAATGTTATGATAATACGGTATAAGTTGAAGTATGAGGTGAAAGAGGAGATATTAGAATATTAAAAACCCAATTATATTATTTATAATACATACAACA
CGCTATGACAAAAGGGGAAAAATGAAGAATCGGGGGTTGATTGGTCTATCGTATCGCTTAAGGGCCGCAGGCCCGTCAAGATGGATGAACGGTCAGATTTGATTGCTTAGCCACGAAGAA
AGGAATCTTTTTGGGACCACAGATAAGACAGCTGTCATTACCTTTTAAAATAATATAATAACCAATTGACGATAGTACCCCTCCCAAAGATGACGTATAGGGGTGTCCCGAGGTAATTTA
ACATAGCTCTGACAAGAGATAGTGGACGTTGGATGCCACGATCGGACGATCGTGGTTTGATGAACTTATGATGACGTAGGGGCTGGGGCTTAT

ALYU-44 - Musa ornata - Laos

Virome
1. **BBTV** six components

DNA-C
4-12 SNPs to KY427060 MF039864 MF039876 MF039870 (**Thailand**). **30 SNPs** to KM607099 KM607098 (**China**)

C protein (161 aa): 1 aa to KY427060 (**Thailand**) **2 aa** to KM607098 KM607099 (**China**) MF039864 (**Thailand**)

>C_alyu44
TATTACCCCCCCTGCTCGGGACGGGACATCACGTGCATCTAACAAATGCACGTGACAATGCAGTAGCTTGTAGCGAAAGATAACCATCACTATCATAAAAGTGAAAGGAATATTCGTTGC
TTAGGGACTAAGCAACGTGGATAGATATTTGTTCGAGAAGCGACAATGGAGGCTATTTAAACCTGATGGTTTTGTGATTTCCGAAATCACTCATCGGAGAAGAAATGGAGTTCTGGGAAT
CGTCTGCCATGCCAGACGATGTCAAGAGAGTGATCAAGGAAATATATTGGGAGCATCGAAACAAACTTCTGTTTTGTCAGAAGTTGAAAGGCTGTGTAAGAAGGATTCTGGAGCATGGAA
ATCAAGATGATGCTCTAGACGCAGTGAAGGAGATGAAGACTTCTATTATTCGCTATAGCGAATATTTGAAGAAACCTTGTGTGGTATTGTGTTTTAGTACTAATAAATCTATTGCATATA
GGTTAAACACAATGGTGTTCTTTTATCATGAATATATTGAAGACCTAGGTGGTGACTACTCGGTATATCAAGATTTGTTCTGTGATGAGGAACTTCCTTATTCGTCGACAGAAGAAGAAG
ATGAAGAAGTAATATACAGGAATGTTATCATGGCATCGACAGAAGAGAAGATCTCTTGGAGTGAATGTCAAAAGATAGTTATATCAGATTATGATGTAACATTACTGTAATGAATATCCA

TTATGATAAATAAAATAATGGAATGATGATTATGTATTCTAGTATAAATACATAATGGTATACGTATAGCATAAAATACATATACCTACATACAACACACTATAACAAACAGGGAAAAAT
GAAGAATCGGGGGTTGATTGGGCTATCTTAACGATTAAGGGCCGCAGGCCCGTTTAAATATGTGGTGGACGAAGTCCACAACACATAAAAAGTGATCAGAACAATGGAATATAATGAGCT
GGCAACGTAGGGACCATGTCCCGAGTTAGTGCGCCACGTGAGCAGGGGGGCTTAT

DNA-M
7-13 SNPs to KY427061 MF039865 MF039871 MF039876 (**Thailand**). **56-65 SNPs** to KM607239 KM607240 (**China**)

M protein (117 aa):0 aa to KC581796 (**Thailand**) **1 aa** to MF039871 MF039865 MF039877 KY427061 (**Thailand**)

>M_alyu44
TATTACCCCCAGCGCTCAGGACGGGACATCACGTGCAACTAACAAATGCACGTGACTGATATATACTACATAACGGTTTATTGAACCGTTATATATTGTTGTAACGCAAAGTCACGTGTG
ATAGCGAAAGGCACGTGACCAAGACAAATGTATTGAATAAACATTTGACGTCCGGATGCTTCCTACGGAAGCCATGGTTGCTTCGTGGCGAAGCAAAACATTTATATATTGGCTTGGATT
GCTGCCTATAAATAGGCACGCAGGGAAATGGCATTAACAACAGAGCGAGTGAAACTATTCTTTGAATGGTTTCTGTTTATTGGTGCAATATTTATTGCGATAACAATATTATACATATTG
TTGGCGTTGCTCTTTGAGGTCCCAAAGTATATTAAGGATGTTGTGAGGTATCTCGTAGAATACCTGACCAGACGACGTGTATGGATGCAGAGAACGCAGTTGACGGAGGCAACAGGAGAT
GTAGAACTCGGCAGAGGTAGTGTGGAAGACAGACGGGATCAACAACCGGCTGTCATACCACATGCAACACAGGTTATCCCTTCGCATCAACCTAGAAGGGATGAGCAAGGAAGACGAGGA
AACGCCGGACCGATGTTTTAATACACGGTATTGTAATATACGAAATATAAATGGATAATGATAAGTATTGTGAAACAAAATATATGTGAAACATAATATATGTTTGATAATTTACATATT
GTAATATGTGAATTGTATACGAGTGTTGTATTTATAAAACATACAACACGCTATGACAAACAGGGAAAAATGAAGAATCGGGGGTTGATTGGTCTATCGTATCGCTTAAGGGCCGCAGGC
CCGTTGAAATGATTCTTTATAAAACAAATATACATGATACGGATTGTTGAAAATATAATCAACGATGTATAAATACAACAGAATGTTGTATAGCAATTAAAATAATGAGAAGATAAGTAT
ATTTGTGAAGGATATGGATCACAACCACCACTTTAGTGGTGGGTCATATGTCCCGAGTTAGTGCGCCACGTAAGCGCTGGGGCTTAT

DNA-N
1-5 SNPs to MF039878 MF039866 KY427062 MF039866 (**Thailand**). **23-33 SNPs** to KM607387 KM607385 KM607386 (**China**)

N protein (154 aa): 0 aa to MF039872 MF039878 KY427062 (**Thailand**) KM607387 KM607385 (**China**) **1 aa** to MF039866 (**Thailand**)

>N_alyu44
TATTACCCCCCGTGCTCGGGACGGGACATGACGTCAGCATAGATTATAATGGGCCAACTAAAGGCCCATTTAaTAGAATAGGCGGGCTTTTGACATATTTCAAAGGCCCAGCCTGGAAGT
GGATAATGTCACGTGCCGAATAAGAGGTTGCTTCGCCTCGAAGCAACCCGAACAAATGTTGCGTATTCAATACGCAACTGAAGTCTATTAATATGGATGTCTCTGCCGAATAAATCAGAG
CGTAAGCGAAGCAGAAGCGATGGATTGGGCAGAATCACAATTCAAGACATGTACTCATGGGTGCGATTGGAAGACGATATCATCGGATTCATCCGAGAATCGACAATATGTACCTTGCGT
CGACGCAGGCAGCGGAAGAAAGTCACCTCGCAAGGTACTGCTAAGATCTATTGAAGCTGTGTTTAATGGAAGCTTCAAAGGAAATAACAGGAATGTTCGTGGGTTTATATACGTATCAAT
ACGAGACGATGATGGAGAAATGCGTCCAGTACTCATAATACCATTCGGAGGATATGGATATCATAATGATTTCTATTATTTCGAAGGGAAGGGGAAAGTTGAATGTGATATATCATCAGA
TTATGTAGCGCCAGGAGTCGATTGGAGCAGAGACATGGAAGTTAGTATTAGTAACAGCAACAACTGTAATGAATTATGTGATCTGAAGTGTTATGTTGTTTGTTCGTTAAGAATAAAGGA
ATAACAGATGTGCTGTAATGAATATTAATAAAATATATTTTCATGTAATTGATAGTTGTATAAAACATACAACACGCTATGACAAACAGGGAAAAATGAAAAATCGGGGGTTGATTGGTC
TATCGTATCGCTTAAGGGCCGCAGGCCCGTTGAAAAATAATAATCGAATTATTAACGTTTGATAATAATCAGAGATAGATGATAAGCATCCAAAAAACATAGACGAAGTATATGGCTGTA
TAATAAAAACAAGCATATAAATAAAATATGTGAACTAATCTCTGATTGGTTTACAACGTAGCCCCACCAACTTTATGTTGGTGGAAATGTCCCGATGACGTAAGCACGGGGGACTAT

a/c minor

DNA-R
4-7 SNPs to MF039879 MF039873 MF039867 KY427063 (**Thailand**). **10 SNPs** to AF416475 (**Viet Nam**). **15-17 SNPs** to KM607677 KM607678 KM607676 KM607679 (**China**)

R protein (286 aa): 0 aa to KM607676 KM607677 KM607679 (**China**) **1 aa** to AB113660 (**Vietnam**) MF039879 (**Thailand**) KM607678 (**China**) …

>R_alyu44
TATTACCCCCAGCGCTCGGGACGGGACATTTGCATCTATAAATAGACCTCCCCCCCCTCCACTACAAGATCATCATCGTCGACAGAAATGGCGCGATATGTGGTATGCTGGATGTTCACC
ATCAACAATCCCGCTTCACTACCAGTGATGCGGGATGAGTTTAAATATATGGTATATCAAGTGGAGAGGGGACAGGAGGGTACTCGTCATGTGCAGGGATACGTCGAGATGAAGAGACGA
AGTTCTCTGAAGCAGATGAGAGGCTTCTTCCCTGGCGCACACCTTGAGAAACGAAAGGGAAGCCAAGAAGAAGCACGGGCATACTGTATGAAGGAAGACACAAGAATCGAAGGTCCCTTC
GAGTTTGGTGCCTTTAAATTGTCATGTAATGATAATTTATTTGATGTCATACAGGATATGCGTGAAACGCACAAACGGCCTTTGGAGTATTTATATGAGTGTCCAAATACCTTCGATAGA
AGTAAGGATACATTATACAGAGTTCAAGCAGAGTTGAATAAAACGAAGGCGATGAATAGCTGGAAAACATCCTTCAGTTCTTGGACATCGGAAGTTGAAAATATTATGGCGGAGCCATGT
CATCGAAGGATAATTTGGGTCTATGGCCCAAATGGAGGAGAAGGAAAGACAACTTATGCAAAATATTTAATGAAGACGAAGAATGCGTTTTATTCGCCAGGAGGAAAATCTTTGGATATA
TGTAGATTGTATAATTATGAAGAAATAGTTATATTTGATATTCCCAGATGCAAAGAGGAATATTTAAACTATGGTTTATTAGAAGAATTTAAAAATGGAATTATTCAAAGCGGGAAATAT
GAACCCGTTTTGAAAATTGTAGAATATGTGGAAGTCATTGTAATGGCTAACTTCCTTCCGAAGGAAGGAATCTTCTCTGAAGATCGAATAAAGCTAGTTGCTTGCTGAACACGCTATGAC
AATCGTACGCTATGACAAAAGGGGAAAAGCAAAGAATCGGGGGTTGATTGGGCTATCCTAACGATTAAGGGCCGCAGGCCCGTCAAGATGGACGGTTTGATCAGATGTCCCGAGTTAGTG
CGCCACGTAAGCGCTGGGGCTTAT

DNA-S
8-19 SNPs to MF039874 KY427064 MF039880 (**Thailand**). **27-32 SNPs** to AB113661 AB113662 AF148945 (**Viet Nam**). **30-33 SNPs** to KM607535 KM607534 KM607536 KM607533 (**China**)

S protein (170 aa): 0 aa to AY337715 AY494786 HQ378191 (**China**) FJ787433 FJ787434 (**Philippines**) **1 aa** to several isolates

>S_alyu44
TATTACCCCCAGCGCTCGGGACGGGACATGGGCTAATGGATCATGGAATAAGGGCCCATCGGGCCCGTTAAGATGGGTTTTGGGCTTATGGGCTTTATCCAGAAGACCAAAAACAGGCGG
GAACCGTCCCAATTTCAAATTTAGATAGCTTGCCCCGCAAGCAAACTAAAAGTCTATATATACCAGTGTAGACATATTGTTCAGATAACAAATGGCGAGGTTTCCGAAGAAATCGATCAA
GAAGAGGCGGGTTGGGCGACGGAAGTATGGAAGCAAGGCGGCAACCAGTCACGACTACTCGTCGTTAGGTTCAATATTGGTTCCTGAAAATACCGTGAAGGTATTTAGGATTGAGCCTAC
TGATAAAACATTACCCAGATATTTTATCTGGAAAATGTTTATGCTTTTGGTGTGCAAGGTGAAGCCTGGAAGAATACTTCATTGGGCTATGATTAAAAGTTCATGGGAAATCAACCAGCC
GACTACATGTCTGGAAGCACCAGGTTTATTCATAAAACCTGAACATAGCCATCTGGTTAAACTGGTATGCAGTGGGGAACTTGAAGCCGGAGTCGCAACAGGGACATCAGATGTAGAATG
TCTTCTAAGGAAGACAACCGTGTTGAGGAAGAATGTAACAGAGGTGGATTACTTGTATTTGGCATTTTATTGTAGTGCTGGAGTTAGTATTAACTACCAGAACAGAATTACATATCATGT
ATGATCTGTTTATGTAAACATAAACCTTTGTATGGATTAATGACCAAATAACATACAACACGCTATGAAATACAACACGCTATGACAAAAGGGGAAAAATGAAGAATCGGGGGTTGATTG
GTCTATCGTATCGCTTAAGGGCCGCAGGCCCGTTGAAATGATTCTTTATAAACAAATATACATGATACGGATTGTTGAAAATATAATCAACGATGTATAAATACAACAGAATGTTGTATA
GCAATTAAAAAAAAAAAGAGAAGAAAAGAATATTTGTGAAGGATAAGCATCAGAACCACCACTTTAGTGGTGGGTCAGATGTCCCGAGTTAGTGCGCCACGTAAGCGCTGGGGCTTAT

DNA-U3
8/10/13/19/ SNPs to MF039881 MF039875 KY427065 MF039869 (**Thailand**)

U3 protein (39 aa): 0 aa to (**Thailand)**

>U3_alyu44
TATTACCCCCAGCCCCCAGGACGGGACATGGGCTTTTTAAATGGGCCGAGAGAGTTTGAACAGTTCAGTATCTTCGTTATTGGGCCATCTGGCCCAATAATTAAGAGAACGTGTTCAAAA
TCGGGGTTTGACCGAAGGTCAAGGTAGACGGTCAACAATATTCTGGCTTGCGGAGCAAGCAACACGAATTAAATATTTAATTCGTATGACACGTGGACGGACCGAAATACTCCTGTAACT
CTATAAATAGCTTAAATCTGGCTTGGATAATTGCTCTCGCTCTTCTGTCAAAGGTGTTGTGTTGAGGCGGAAGATCGCCATCGGCGATCATCAGACGAAAAGCTGCAAGAGAGACGGAGA
AACATGCTGCGAAGCGTATATCGGGTATTTATAGACTTCTAGCGCAGCTAGAAGTTTCGTTGTACTTGATATTGTATTTTGTAAATTACGAAGAAATCGTACATTGATAATAATAAAACA
TCTGGGATTGTTAATGTTTACATTAACCAGTATTAATAATGTACAATAAAAAATATTGTATGAGGAACGAATACAATAATATTAAAATATGAGGAGCGTAGCATGATAAACAGGTGTTTA
AGGTATAATTAAAAAAATTATGTAATGTTATGATAATACGGTATAAGTTGAAGTATGAGGTGAAAGAGGAGATATTAGAATATTAAAAACCCAATTATATTATTTATAATACATACAACA
CGCTATGACAAAAGGGGAAAAATGAAGAATCGGGGGTTGATTGGTCTATCGTATCGCTTAAGGGCCGCAGGCCCGTCAAGATGGATGAACGGCCAGATTTGATTGCTTAGCCACGAAGAA
AGGAATCTTTTTGGGACCACAGATAAGACAGCTGTCATTACCTTTTAAAATAATATAATAACCAATTGACGATAGTACCCCTCCCAAAGATGACGTATAGGGGTGTCCCGAGGTAATTTA
ACATAGCTCTGACAAGAGATAGTGGACGTTGGATGCCACGATCGGACGATCGTGGTTTGATGAACCTATGATGACGTAGGGGCTGGGGCTTAT

ALYU-45 – Musa sp. – Laos

Virome

1. **BBTV** six components (near identical to ALYU-44 in Musa ornata at the same location: 1 SNP in M and 1 nt del in S, both in non-coding sequence)

DNA-C = alyu44

>C_alyu45
TATTACCCCCCCTGCTCGGGACGGGACATCACGTGCATCTAACAAATGCACGTGACAATGCAGTAGCTTGTAGCGAAAGATAACCATCACTATCATAAAAGTGAAAGGAATATTCGTTGC
TTAGGGACTAAGCAACGTGGATAGATATTTGTTCGAGAAGCGACAATGGAGGCTATTTAAACCTGATGGTTTTGTGATTTCCGAAATCACTCATCGGAGAAGAAATGGAGTTCTGGGAAT
CGTCTGCCATGCCAGACGATGTCAAGAGAGTGATCAAGGAAATATATTGGGAGCATCGAAACAAACTTCTGTTTTGTCAGAAGTTGAAAGGCTGTGTAAGAAGGATTCTGGAGCATGGAA
ATCAAGATGATGCTCTAGACGCAGTGAAGGAGATGAAGACTTCTATTATTCGCTATAGCGAATATTTGAAGAAACCTTGTGTGGTATTGTGTTTTAGTACTAATAAATCTATTGCATATA
GGTTAAACACAATGGTGTTCTTTTATCATGAATATATTGAAGACCTAGGTGGTGACTACTCGGTATATCAAGATTTGTTCTGTGATGAGGAACTTCCTTATTCGTCGACAGAAGAAGAAG
ATGAAGAAGTAATATACAGGAATGTTATCATGGCATCGACAGAAGAGAAGATCTCTTGGAGTGAATGTCAAAAGATAGTTATATCAGATTATGATGTAACATTACTGTAATGAATATCCA
TTATGATAAATAAAATAATGGAATGATGATTATGTATTCTAGTATAAATACATAATGGTATACGTATAGCATAAAATACATATACCTACATACAACACACTATAACAAACAGGGAAAAAT
GAAGAATCGGGGGTTGATTGGGCTATCTTAACGATTAAGGGCCGCAGGCCCGTTTAAATATGTGGTGGACGAAGTCCACAACACATAAAAAGTGATCAGAACAATGGAATATAATGAGCT
GGCAACGTAGGGACCATGTCCCGAGTTAGTGCGCCACGTGAGCAGGGGGGCTTAT

DNA-M 1 SNP to alyu44

>M_alyu45
TATTACCCCCAGCGCTCAGGACGGGACATCACGTGCAACTAACAAATGCACGTGACTGATATATACAACATAACGGTTTATTGAACCGTTATATATTGTTGTAACGCAAAGTCACGTGTG
ATAGCGAAAGGCACGTGACCAAGACAAATGTATTGAATAAACATTTGACGTCCGGATGCTTCCTACGGAAGCCATGGTTGCTTCGTGGCGAAGCAAAACATTTATATATTGGCTTGGATT
GCTGCCTATAAATAGGCACGCAGGGAAATGGCATTAACAACAGAGCGAGTGAAACTATTCTTTGAATGGTTTCTGTTTATTGGTGCAATATTTATTGCGATAACAATATTATACATATTG
TTGGCGTTGCTCTTTGAGGTCCCAAAGTATATTAAGGATGTTGTGAGGTATCTCGTAGAATACCTGACCAGACGACGTGTATGGATGCAGAGAACGCAGTTGACGGAGGCAACAGGAGAT
GTAGAACTCGGCAGAGGTAGTGTGGAAGACAGACGGGATCAACAACCGGCTGTCATACCACATGCAACACAGGTTATCCCTTCGCATCAACCTAGAAGGGATGAGCAAGGAAGACGAGGA
AACGCCGGACCGATGTTTTAATACACGGTATTGTAATATACGAAATATAAATGGATAATGATAAGTATTGTGAAACAAAATATATGTGAAACATAATATATGTTTGATAATTTACATATT
GTAATATGTGAATTGTATACGAGTGTTGTATTTATAAAACATACAACACGCTATGACAAACAGGGAAAAATGAAGAATCGGGGGTTGATTGGTCTATCGTATCGCTTAAGGGCCGCAGGC
CCGTTGAAATGATTCTTTATAAAACAAATATACATGATACGGATTGTTGAAAATATAATCAACGATGTATAAATACAACAGAATGTTGTATAGCAATTAAAATAATGAGAAGATAAGTAT
ATTTGTGAAGGATATGGATCACAACCACCACTTTAGTGGTGGGTCATATGTCCCGAGTTAGTGCGCCACGTAAGCGCTGGGGCTTAT

A – T in alyu44

DNA-N = alyu44

>N_alyu45
TATTACCCCCCGTGCTCGGGACGGGACATGACGTCAGCATAGATTATAATGGGCCAACTAAAGGCCCATTTAATAGAATAGGCGGGCTTTTGACATATTTCAAAGGCCCAGCCTGGAAGT
GGATAATGTCACGTGCCGAATAAGAGGTTGCTTCGCCTCGAAGCAACCCGAACAAATGTTGCGTATTCAATACGCAACTGAAGTCTATTAATATGGATGTCTCTGCCGAATAAATCAGAG
CGTAAGCGAAGCAGAAGCGATGGATTGGGCAGAATCACAATTCAAGACATGTACTCATGGGTGCGATTGGAAGACGATATCATCGGATTCATCCGAGAATCGACAATATGTACCTTGCGT
CGACGCAGGCAGCGGAAGAAAGTCACCTCGCAAGGTACTGCTAAGATCTATTGAAGCTGTGTTTAATGGAAGCTTCAAAGGAAATAACAGGAATGTTCGTGGGTTTATATACGTATCAAT
ACGAGACGATGATGGAGAAATGCGTCCAGTACTCATAATACCATTCGGAGGATATGGATATCATAATGATTTCTATTATTTCGAAGGGAAGGGGAAAGTTGAATGTGATATATCATCAGA
TTATGTAGCGCCAGGAGTCGATTGGAGCAGAGACATGGAAGTTAGTATTAGTAACAGCAACAACTGTAATGAATTATGTGATCTGAAGTGTTATGTTGTTTGTTCGTTAAGAATAAAGGA
ATAACAGATGTGCTGTAATGAATATTAATAAAATATATTTTCATGTAATTGATAGTTGTATAAAACATACAACACGCTATGACAAACAGGGAAAAATGAAAAATCGGGGGTTGATTGGTC
TATCGTATCGCTTAAGGGCCGCAGGCCCGTTGAAAAATAATAATCGAATTATTAACGTTTGATAATAATCAGAGATAGATGATAAGCATCCAAAAAACATAGACGAAGTATATGGCTGTA
TAATAAAAACAAGCATATAAATAAAATATGTGAACTAATCTCTGATTGGTTTACAACGTAGCCCCACCAACTTTATGTTGGTGGAAATGTCCCGATGACGTAAGCACGGGGGACTAT

DNA-R = alyu44

>R_alyu45
TATTACCCCCAGCGCTCGGGACGGGACATTTGCATCTATAAATAGACCTCCCCCCCCTCCACTACAAGATCATCATCGTCGACAGAAATGGCGCGATATGTGGTATGCTGGATGTTCACC
ATCAACAATCCCGCTTCACTACCAGTGATGCGGGATGAGTTTAAATATATGGTATATCAAGTGGAGAGGGGACAGGAGGGTACTCGTCATGTGCAGGGATACGTCGAGATGAAGAGACGA
AGTTCTCTGAAGCAGATGAGAGGCTTCTTCCCTGGCGCACACCTTGAGAAACGAAAGGGAAGCCAAGAAGAAGCACGGGCATACTGTATGAAGGAAGACACAAGAATCGAAGGTCCCTTC
GAGTTTGGTGCCTTTAAATTGTCATGTAATGATAATTTATTTGATGTCATACAGGATATGCGTGAAACGCACAAACGGCCTTTGGAGTATTTATATGAGTGTCCAAATACCTTCGATAGA
AGTAAGGATACATTATACAGAGTTCAAGCAGAGTTGAATAAAACGAAGGCGATGAATAGCTGGAAAACATCCTTCAGTTCTTGGACATCGGAAGTTGAAAATATTATGGCGGAGCCATGT
CATCGAAGGATAATTTGGGTCTATGGCCCAAATGGAGGAGAAGGAAAGACAACTTATGCAAAATATTTAATGAAGACGAAGAATGCGTTTTATTCGCCAGGAGGAAAATCTTTGGATATA
TGTAGATTGTATAATTATGAAGAAATAGTTATATTTGATATTCCCAGATGCAAAGAGGAATATTTAAACTATGGTTTATTAGAAGAATTTAAAAATGGAATTATTCAAAGCGGGAAATAT
GAACCCGTTTTGAAAATTGTAGAATATGTGGAAGTCATTGTAATGGCTAACTTCCTTCCGAAGGAAGGAATCTTCTCTGAAGATCGAATAAAGCTAGTTGCTTGCTGAACACGCTATGAC
AATCGTACGCTATGACAAAAGGGGAAAAGCAAAGAATCGGGGGTTGATTGGGCTATCCTAACGATTAAGGGCCGCAGGCCCGTCAAGATGGACGGTTTGATCAGATGTCCCGAGTTAGTG
CGCCACGTAAGCGCTGGGGCTTAT

DNA-S 1 nt indel to alyu44

>S_alyu45
TATTACCCCCAGCGCTCGGGACGGGACATGGGCTAATGGATCATGGAATAAGGGCCCATCGGGCCCGTTAAGATGGGTTTTGGGCTTATGGGCTTTATCCAGAAGACCAAAAACAGGCGG
GAACCGTCCCAATTTCAAATTTAGATAGCTTGCCCCGCAAGCAAACTAAAAGTCTATATATACCAGTGTAGACATATTGTTCAGATAACAAATGGCGAGGTTTCCGAAGAAATCGATCAA
GAAGAGGCGGGTTGGGCGACGGAAGTATGGAAGCAAGGCGGCAACCAGTCACGACTACTCGTCGTTAGGTTCAATATTGGTTCCTGAAAATACCGTGAAGGTATTTAGGATTGAGCCTAC
TGATAAAACATTACCCAGATATTTTATCTGGAAAATGTTTATGCTTTTGGTGTGCAAGGTGAAGCCTGGAAGAATACTTCATTGGGCTATGATTAAAAGTTCATGGGAAATCAACCAGCC
GACTACATGTCTGGAAGCACCAGGTTTATTCATAAAACCTGAACATAGCCATCTGGTTAAACTGGTATGCAGTGGGGAACTTGAAGCCGGAGTCGCAACAGGGACATCAGATGTAGAATG
TCTTCTAAGGAAGACAACCGTGTTGAGGAAGAATGTAACAGAGGTGGATTACTTGTATTTGGCATTTTATTGTAGTGCTGGAGTTAGTATTAACTACCAGAACAGAATTACATATCATGT
ATGATCTGTTTATGTAAACATAAACCTTTGTATGGATTAATGACCAAATAACATACAACACGCTATGAAATACAACACGCTATGACAAAAGGGGAAAAATGAAGAATCGGGGGTTGATTG
GTCTATCGTATCGCTTAAGGGCCGCAGGCCCGTTGAAATGATTCTTTATAAACAAATATACATGATACGGATTGTTGAAAATATAATCAACGATGTATAAATACAACAGAATGTTGTATA
GCAATTAAAAAAAAAAGAGAAGAAAAGAATATTTGTGAAGGATAAGCATCAGAACCACCACTTTAGTGGTGGGTCAGATGTCCCGAGTTAGTGCGCCACGTAAGCGCTGGGGCTTAT

A - AA in_alyu44

DNA-U3 = alyu44

>U3_alyu45
TATTACCCCCAGCCCCCAGGACGGGACATGGGCTTTTTAAATGGGCCGAGAGAGTTTGAACAGTTCAGTATCTTCGTTATTGGGCCATCTGGCCCAATAATTAAGAGAACGTGTTCAAAA
TCGGGGTTTGACCGAAGGTCAAGGTAGACGGTCAACAATATTCTGGCTTGCGGAGCAAGCAACACGAATTAAATATTTAATTCGTATGACACGTGGACGGACCGAAATACTCCTGTAACT
CTATAAATAGCTTAAATCTGGCTTGGATAATTGCTCTCGCTCTTCTGTCAAAGGTGTTGTGTTGAGGCGGAAGATCGCCATCGGCGATCATCAGACGAAAAGCTGCAAGAGAGACGGAGA
AACATGCTGCGAAGCGTATATCGGGTATTTATAGACTTCTAGCGCAGCTAGAAGTTTCGTTGTACTTGATATTGTATTTTGTAAATTACGAAGAAATCGTACATTGATAATAATAAAACA
TCTGGGATTGTTAATGTTTACATTAACCAGTATTAATAATGTACAATAAAAAAATATTGTATGAGGAACGAATACAATAATATTAAAATATGAGGAGCGTAGCATGATAAACAGGTGTTTA
AGGTATAATTAAAAAAATTATGTAATGTTATGATAATACGGTATAAGTTGAAGTATGAGGTGAAAGAGGAGATATTAGAATATTAAAAACCCAATTATATTATTTATAATACATACAACA
CGCTATGACAAAAGGGGAAAAATGAAGAATCGGGGGTTGATTGGTCTATCGTATCGCTTAAGGGCCGCAGGCCCGTCAAGATGGATGAACGGCCAGATTTGATTGCTTAGCCACGAAGAA
AGGAATCTTTTTGGGACCACAGATAAGACAGCTGTCATTACCTTTTAAAATAATATAATAACCAATTGACGATAGTACCCCTCCCAAAGATGACGTATAGGGGTGTCCCGAGGTAATTTA
ACATAGCTCTGACAAGAGATAGTGGACGTTGGATGCCACGATCGGACGATCGTGGTTTGATGAACCTATGATGACGTAGGGGCTGGGGCTTAT

ALYU-46 S22 La5 V7-Laos - Musa ABB Klue Tiparot - Laos

Virome **(-)**
“Virus-free”: Reads representing BBTV genome components are at a cross-contamination level (see Supplementary Figure S3H). BBTA5: 96 reads; BBTA6: 21 reads; BBTA2: 44 reads + spot 30 reads; BBTA3: 17 reads

ALYU-47 - Musa yunnanensis - Laos

Virome

1. **BBTV** six components

DNA-C

28-36 SNPs to KM607098 KM607099 (**China**). **31-36 SNPs** to KY427060 MF039864 MF039876 MF039870 (**Thailand**)

C protein (161 aa): 1 aa to KY427060 (**Thailand**) **2 aa** to KM607098 KM607099 (**China**) MF039864 (**Thailand**)

>C_alyu47
TATTACCCCCAGCGCTCAGGACGGGACATCACGTGCATCTAACAAATGCACGTGACAAAGCAGTAGCTTGTAGCGAAAGATAACCATCACTATCACAAAAGTGAGAGGAATATTCGTTGC
TTAGGGACTAAGCAACGTGAACAGATATTTGTTCGAGAAGCGAAAATGGAGGCTATTTAAACCTGATGGTTTTGTGATTTCCGAAATCACTCATCGGAGCAGAAATGGAGTTCTGGGAAT
CGTCTGCCATGCCAGACGATGTCAAGAGAGTGATCAAGGAAATATATTGGGAGCATCGAAATAAACTTCTGTTCTGTCAGAAGTTGAAAGGCTGTGTAAGAAGGATTCTGGAGCATGGAA
ATCAAGATGATGCTCTAGACGCAGTGAAGGAGATGAAGACTTCTATTATTCGCTATAGCGAATATTTGAAGAAACCTTGTGTGGTATTGTGTTTTAGTACTAATAAATCTATTGCATATA
GGTTAAACACAATGGTGTTCTTTTATCATGAATATATTGAAGACCTAGGTGGTGACTATTCGGTATATCAAGATCTGTTCTGTGATGAGGAACTTCCTTATTCGTCGACAGAAGAAGAAG
ATGAAGAAGTAATATACAGGAATGTTATCATGGCATCGACAGAAGAGAAGATCTCTTGGAGTGAATGTCAAAAGATAGTTATATCAGATTATGATGTAACATTACTGTAATGAATATCCA
TTATCATAAATAAAATAATGGAATGATGATTATGTATTCTAGTATATATACATAATGGTATACGTATAGCATAAAATACATAAACCAACATACAACACGCTATGACAAACGGGGAAAAAT
GAAGAATCGGGGGTTGATTGGGCTATCTTAACGATTAAGGGCCGAAGGCCCGTTTAAATATGTGGTGGACGAAGTCCACAACACATAAAAAGTGCTCAGAACAAGGGAATATAATGAGCT
GGCAACGTAGGGACCATGTCCCGAGTTAGTGCGCCACGTAAGCGCTGGGGCTTAT

DNA-M

13-18 SNPs to KM607239 KM607240 (**China**). **55-57 SNPs** to MF039865 KY427061 MF039871 MF039876 (**Thailand**)

M protein (117 aa): 2 aa MF039865 MF039877 (**Thailand**) **3 aa** to KC581796 (**Thailand**) **4 aa** to MF039871 KY427061 (**Thailand**)

>M_alyu47
TATTACCCCCAGCGCTCAGGACGGGACATCACGTGCAACTAACAAATGCACGTGACTGATATATACTACATAAAGGTTTAACGAACCGTTATATAATGTTATAACGAAAAGTCACGTGTG
ATAGAGACCTGCACGTGACATAGTCAAATGTATTGAATAAACATTTGACGTCCGGATGCTTCCGACGGAAGCTTAGGTTACTTCGTGGCGAAGCAAAACATTTATATATTGGCCTGGACT
GCTGCCTATAAATAGGCAAGCTAGGAAATGGCATTAACAACAGAGCGGGTGAAACTATTCTTTGAATGGTTTCTGTTCATCGGTGCAATATTTATTGCGATAACAATATTATATATATTG
TTGGTGTTGCTCTTTGAGGTCCCCAAGTATATTAAGGATGTTGTGAGGTATCTCGTAGAATACCTGACCAGAAGACGTGTATGGATGCAGAGAACGCAGTTGACGGAGGCAACAGGAGAT
GTAGAGCTCGGCAGAGGTATTGTGGAAGACAGACGGGATCAACAACCGGCTGTCATACCACATGCAACACAGGTTATCCCTTCGCATCAACCTAGAAGGGATGATCAAGGAAGACGAGGA
AACGCAGGACCAATGTTTTAATACACGGTATTGTAATATACAAAATATAAATGGATAATGATATGTATTGTGAAACATAATATACGTGAAACATAATATATGTTTGATAATTTACATATT
ATAATATGTAAATTGTATACGAGTGTTGTATTTATAGAACATACAACACGCTATGACAAACAGGGAAAAATGAAGAATCGGGGGTTGATTGGTCTATCGTATCGCTTAAGGGCCGCAGGC
CCGTTGAAATGATTCTTAATAAAACAAATATACATGATACGGATAGTTGAATATATAAACAACGAAGTATAAATACAACAGAATGGTGTATACTAATTAAAATAATGTGAAGATAAGTAT
ATTTGTGCGGGATGATGATCACAACCACCACTTTAGTGGTGGGTCATATGTCCCGAGTTAGTGCGCCACGTAAGCGCTGGGGCTTAT

DNA-N

26-29 SNPs to KX787074 EF470243 AF238879 KX787071 KX787072 (**China**)

N protein (154 aa): 1 aa to AF238879 KX787070 KX787071 KX787072 KX787074 (**China**) **3 aa** to AY494787 KX787073 (**China**) KM607304 KM607313 KM607314 (**Philippines**) KM607316 KM607319 (**Taiwan**)

>N_alyu47
TATTACCCCCCGTGCTCGGGACGGGACATGACGTCAGCATATATTATAATGGGCTTTTTAAAGCCCATATAAGTGAAGTGGGCCGGGTTTGAGACATATTTCGAAAGCCCGACCCGGAAA
AGGATAAAGTCACGTGCCGAATAATAGGTTGCTTCGCCTCGAAGCAAACTAATAAAAAGTTGCGTATTCAATACGCAACTAAGTCTATTAATAGGGTTGTCTCTGCCGAATAAATCAGAG
CGTATGCGAAGCAGAAGCGATGGATTGGGCAGAATCACAATTCAAGACATGTACCCATGGCTGTGATTGGAAGACGATATCATCGGATTCATCAGAAAATCGGCAATATGTACCTTGCGT
CGACTCTGGAGCTGGAAGAAAGACTCCTCGCAAGGTACTTCTTAGATCTATTGAAGCTGTGTTTAATGGAAGCTTCAAAGGGAATAATAGGAATGTTCGTGGCTTCTTATACGTATCAAT
CCGAGACGATGATGGAGCAATGCGTCCAGTACTTATAGTACCATTTGGAGGATATGGATATCATAATGATTTCTATTATTTCGAAGGGAAGGGGAAAGTTGAATGTGATATATCATCAGA
TTATGTTGCGCCAGAAGTCGATTGGAGCAGAGACATGGAAGTTAGTATTAGTAACAGCAACAACTGTAATGAATCATGTGATCTGAAGTGTTATGTTGTTTGTTCGTTAAGAATAAAGGA
ATAACAGATGTGCTGTAATGATTATTAATAAAACATATTTTTCATGCAATTGATAGTTGTATAAAACATACAACACGCTATGACAAACGGGGAAAAATGAAAAATCAGGGGTTGATTGTT
CTATAGTATCGCTTAAGGGCCGCAGGCCCGTTGAAAAATAATAATCGAATTATATACGATTGATAATAATCAGAGATAGATGATAAGGATATATAAACATAAACGAAGTATATGGCGGTA
TAATTAAACAGAAGCATATAATATAAAAATATGTAAACTAATCTCTGATTGGTGCAGAGTGTAGGTCCACTAACTTTAAGTTAGTGGAAATGTCCCGATGACGTAAGCACGGGGGACTAT

DNA-R

4-6 SNPs to KM607677 KM607678 KM607676 KM607679 (**China**). **9-14 SNPs** to AB113660 AB113659 (**Viet Nam**). **14-18 SNPs** to MF039879 MF039873 MF039867 KY427063 (**Thailand**). **14 SNPs** to AF416475 (**Viet Nam**)

R protein (286 aa): 0 aa to KM607676 KM607677 KM607679 (**China**) **1 aa to** AB113660 (**Vietnam**) MF039879 (**Thailand**) KM607678 (**China**)…

>R_alyu47
TATTACCCCCAGCGCTCGGGACGGGACATTTGCATCTATAAATAGACCTCCCCCCCCTCCACTACAAGATCATCATCGTCGACAGAAATGGCGCGATATGTGGTATGCTGGATGTTCACC
ATCAATAATCCCGCTTCACTACCAGTGATGCGGGATGAGTTCAAATATATGGTATATCAAGTGGAGAGGGGACAGGAGGGTACTCGTCATGTGCAGGGATACGTCGAGATGAAGAGACGA
AGTTCTCTGAAGCAGATGAGAGGCTTCTTCCCTGGCGCACACCTTGAGAAACGAAAGGGAAGCCAAGAAGAAGCACGGGCATACTGTATGAAGGAAGACACAAGAATCGAAGGTCCCTTC
GAGTTTGGTGCCTTTAAATTGTCATGTAATGATAATTTATTTGATGTCATACAGGATATGCGTGAAACGCACAAACGGCCTCTGGAGTATTTATATGAGTGTCCAAATACCTTCGATAGA
AGTAAGGATACATTATACAGAGTTCAAGCAGAGTTGAATAAAACGAAGGCGATGAATAGCTGGAAAACATCCTTCAGTTCATGGACATCGGAAGTTGAAAATATTATGGCGGAGCCATGT
CATCGAAGGATAATTTGGGTCTATGGCCCAAATGGAGGAGAAGGAAAGACAACTTATGCAAAATATTTAATGAAGACGAAGAATGCGTTTTATTCTCCAGGAGGAAAATCATTGGATATA
TGTAGATTGTATAATTACGAGGAAATAGTTATATTTGATATTCCCAGATGCAAAGAGGAATATTTAAACTATGGTTTATTAGAAGAATTTAAAAATGGAATTATTCAAAGCGGGAAATAT
GAACCCGTTTTGAAAATTGTAGAATATGTGGAAGTCATTGTAATGGCTAACTTCCTTCCGAAGGAAGGAATCTTTTCAGAAGATCGAATAAAGCTAGTTGCTTGCTGAACACGCTATGAC
AATCGTACGCTATGACAAAAGGGGAAAAGCAAAGATTCGGGGGTTGATTGGGCTATCCTAACGATTAAGGGCCGCAGGCCCGTCAAGATGGACGGCTTGATCAGATGTCCCGAGTTAGTG
CGCCACGTAAGCGCTGGGGCTTAT

DNA-S

5-8 SNPs to KM607535 KM607534 KM607536 KM607533 (**China**). **15-19 SNPs** to AB113662 AB113661 (**Viet Nam**). **19 SNPs** to AF148945 (**Viet Nam**). **20-32 SNPs** to MF039874 KY427064 MF039880 (**Thailand**)

S protein (170 aa): 0 aa to AY337715 AY494786 HQ378191 (**China**) FJ787433 FJ787434 (**Philippines**) **1 aa** to several isolates

>S_alyu47
TATTACCCCCAGCGCTCGGGACGGGACATGGGCTAATGGATCATGGAATAAGGGCCCATCGGGCCCGTTAAGATGGGTTTTGGGCTTATGGGCTTTATCCAGAAGACCAAAAACAGGCGG
GAACCGTCCCAATTTCAAATTTAGATAGCTTGCCCCGCAAGCAAACTAAAAGCCTATATATACCAGTGTAGACATATTGTTCAGATAACAAATGGCGAGGTTTCCGAAGAAATCGATCAA
GAAGCGGCGGGTTGGGCGACGGAAGTATGGAAGCAAGGCGGCAACCAGTCACGACTACTCGTCGTTAGGTTCAATATTGGTTCCTGAAAATACCGTCAAGGTATTTAGGATTGAGCCTAC
TGATAAAACATTACCCAGATATTTTATCTGGAAAATGTTTATGCTTTTGGTGTGCAAGGTGAAGCCCGGAAGAATACTTCACTGGGCTATGATTAAGAGTTCATGGGAAATCAACCAGCC
GACTACATGTCTGGAAGCACCAGGTTTATTTATAAAACCTGAACATAGCCATCTGGTGAAACTGGTATGCAGTGGGGAACTTGAAGCCGGAGTCGCAACAGGGACATCAGATGTTGAATG
TCTTCTAAGGAAGACAACCGTGTTGAGGAAGAATGTAACAGAGGTGGATTACTTGTATTTGGCATTTTATTGTAGTGCTGGAGTTAGTATTAACTACCAGAACAGAATTACATATCATGT
ATGATATGTTTATGTAAACATAAACCTTTGTATGGAATAATGACCAAATAACATACAACACACTATGAAATACAACACGCTATGACAAACAGGGAAAAATGAAGAATCGGGGGTTGATTG
GTCTATCGTAACGCTTAAGGGCCGCAGGCCCGTTGAAATGATTCTTTATAAAACAAATATACATGATACGGATAGTTGAACATATAAACAACGATGTATAAATACAACAGAATGTTGTAT
ACTAATTAAAATAATGAGAAGAAAAGAATATTTGTGAAGGATAAGCATCAGAACCACCACTTTAGTGGTGGGTCAGATGTCCCGAGTTAGTGCGCCACGTAAGCGCTGGGGCTTAT

DNA-U3

35-48 SNPs to KY427065 MF039869 MF039875 MF039881 (**Thailand**)

U3 protein (39 aa): 0 aa to KY427065 (**Thailand)**

>U3_alyu47

TATTACCCCCAGCCCCCAGGACGGGACATGGGCTTTTTAAATGGGCCGAGAGAGTTTGAACAGTTCAGTATCTTCGTTATTGGGCCATCTGGCCCAATAATTAAGAGAACGTGTTCAAAA
TCTGGGTTTGACCGAAGGTCAAGGTAGACGGTCAACAATATTCTGGCTTGCGGAGCAAGCAACAAGAATTAAATATTTAATTCGTATGACACGTGGACGGACCGAAATACTCCTGTATCT
CTATAAATAGCCTAAATCTGGCATGGATAATTGCTCTCGCTCTTCTGTCAAAGCTGTTGTGTTGAGGCGGAAGATCGCCATCGGCGATCATCAGACGAAAAGCTGCAAGAGAGACGGAGA
ACCATGCTGCGAAGCGTATATCGGGTATTTATAGACTTCTAGCGCAGCTAGAAGTTTCCTTGTACTTGTATTTGTATTTTGTAAATTACGAAGAAATTCGTACATTGATAATAATAAAAC
ATCTGGGATTGTTAATGTTTACATTAACTAGTATTAATAATGTACAATAACAAACATTGTATGAGGAACGAATACAAGAATATTAAAATATGAGGAGCGTAGCGTGATAAACAGGTGTTT
AAGGAATAATTAAAATAATTATGCAATGTTATGATAATACGGTATAAGTTGAAGTATGAGGTGAAAGAGGAGATATTAGAATATTAAAAACCCAATTATATTATTTATAATACATACAAC
ACGCTATGACAAAAGGGGAAAAAAGAAAAATCGGGGGTTGATTGGTCTATCCTAACGATTAAGGGCCGCAGGCCCGTCAAGATGGATGAACGGTTAGATTTGATTGCTTAGACACGAAGA
AAGGAATCTTTTTGGGACCACAGATAAGACAGCTGTCACTACTTTTAAAAAATAATATAATAACCAAATGACCATAGTGCCCCTCCCAAAGATGACGTATAGGGGTGTCCCGAGGTAATT
TAACATAGCTCTGACAAGAGATAGTGGACGTTGGATTCCACGATCGGACGATCGTGGTTTGATGAACTTATGATGACGTAGGGGCTGGGGCTTAT

ALYU-48 - Musa sp. - Laos

Virome

1. **BBTV** six components (2 dominant genetic variants for DNA-C and DNA-U3 but not other components)

Close to ALYU-47 in M. yunnanensis at a nearby location

DNA-C v1 - 1 SNP to alyu47

CP is identical in v2 and alyu47

>C_alyu48_v1
TATTACCCCCAGCGCTCAGGACGGGACATCACGTGCATCTAACAAATGCACGTGACAAAGCAGTAGCTTGTAGCGAAAGATAACCATCACTATCACAAAAGTGAGAGGAATATTCGTTGC
TTAGGGACTAAGCAACGTGAACAGATATTTGTTCGAGAAGCGAAAATGGAGGCTATTTAAACCTGATGGTTTTGTGATTTCCGAAATCACTCATCGGAGCAGAAATGGAGGTCTGGGAAT
CGTCTGCCATGCCAGACGATGTCAAGAGAGTGATCAAGGAAATATATTGGGAGCATCGAAATAAACTTCTGTTCTGTCAGAAGTTGAAAGGCTGTGTAAGAAGGATTCTGGAGCATGGAA
ATCAAGATGATGCTCTAGACGCAGTGAAGGAGATGAAGACTTCTATTATTCGCTATAGCGAATATTTGAAGAAACCTTGTGTGGTATTGTGTTTTAGTACTAATAAATCTATTGCATATA
GGTTAAACACAATGGTGTTCTTTTATCATGAATATATTGAAGACCTAGGTGGTGACTATTCGGTATATCAAGATCTGTTCTGTGATGAGGAACTTCCTTATTCGTCGACAGAAGAAGAAG
ATGAAGAAGTAATATACAGGAATGTTATCATGGCATCGACAGAAGAGAAGATCTCTTGGAGTGAATGTCAAAAGATAGTTATATCAGATTATGATGTAACATTACTGTAATGAATATCCA
TTATCATAAATAAAATAATGGAATGATGATTATGTATTCTAGTATATATACATAATGGTATACGTATAGCATAAAATACATAAACCAACATACAACACGCTATGACAAACGGGGAAAAAT
GAAGAATCGGGGGTTGATTGGGCTATCTTAACGATTAAGGGCCGAAGGCCCGTTTAAATATGTGGTGGACGAAGTCCACAACACATAAAAAGTGCTCAGAACAAGGGAATATAATGAGCT
GGCAACGTAGGGACCATGTCCCGAGTTAGTGCGCCACGTAAGCGCTGGGGCTTAT

G to T in alyu47

>C_alyu48_v2
TATTACCCCCAGCGCTCAGGACGGGACATCACGTGCATCTAACAAATGCACGTGACAAAGCAGTAGCTTGTAGCGAAAGATAACCATCACTATCACAAAAGTGAGAGGAATATTCGTTGC
TTAGGGACTAAGCAACGTGAACAGATATTTGTTCGAGAAGCGAAAATGGAGGCTATTTAAACCTGATGGTTTTGTGATTTCCGAAATCACTCATCGGAGCAGAAATGGAGtTCTGGGAAT
CGTCTGCCATGCCAGACGATGTCAAGAGAGTGATCAAGGAAATATATTGGGAGCATCGAAATAAACTTCTGTTCTGTCAGAAGTTGAAAGGCTGTGTAAGAAGGATTCTGGAGCATGGAA
ATCAAGATGATGCTCTAGACGCAGTGAAGGAGATGAAGACTTCTATTATTCGCTATAGCGAATATTTGAAGAAACCTTGTGTGGTATTGTGTTTTAGTACTAATAAATCTATTGCATATA
GGTTAAACACAATGGTGTTCTTTTATCATGAATATATTGAAGACCTAGGTGGTGACTATTCGGTATATCAAGATCTGTTCTGTGATGAGGAACTTCCTTATTCGTCGACAGAAGAAGAAG
ATGAAGAAGTAATATACAGGAATGTTATCATGGCATCGACAGAAGAGAAGATCTCTTGGAGTGAATGTCAAAAGATAGTTATATCAGATTATGATGTAACATTACTGTAATGgATATCCA
TTATCATAAATAAAATAATGGAATGATGATTATGTATTCTAGTATATATACATAATGGTATACGTATAGCATAAAATACATAAACCAACATACAACACGCTATGACAAACGGGGAAAAAT
GAAGAATCGGGGGTTGATTGGGCTATCTTAACGATTAAGGGCCGAAGGCCCGTTTAAATATGTGGTGGACGAAGTCCACAACACATAAAAAGTGCTCAGAACAAtGGAATATAATGAGCT
GGCAACGTAGGGACCATGTCCCGAGTTAGTGCGCCACGTAAGCGCTGGGGCTTAT

DNA-M 2 SNPs to alyu47

1 aa substitution in MP

>M_alyu48
TATTACCCCCAGCGCTCAGGACGGGACATCACGTGCAACTAACAAATGCACGTGACTGATATATACTACATAACGGTTTAACGAACCGTTATATAATGTTATAACGAAAAGTCACGTGTG
ATAGAGACCTGCACGTGACATAGTCAAATGTATTGAATAAACATTTGACGTCCGGATGCTTCCGACGGAAGCTTAGGTTACTTCGTGGCGAAGCAAAACATTTATATATTGGCCTGGACT
GCTGCCTATAAATAGGCAAGCTAGGAAATGGCATTAACAACAGAGCGGGTGAAACTATTCTTTGAATGGTTTCTGTTCATCGGTGCAATATTTATTGCGATAACAATATTATATATATTG
TTGGTGTTGCTCTTTGAGGTCCCCAAGTATATTAAGGATGTTGTGAGGTATCTCGTAGAATACCTGACCAGAAGACGTGTATGGATGCAGAGTACGCAGTTGACGGAGGCAACAGGAGAT
GTAGAGCTCGGCAGAGGTATTGTGGAAGACAGACGGGATCAACAACCGGCTGTCATACCACATGCAACACAGGTTATCCCTTCGCATCAACCTAGAAGGGATGATCAAGGAAGACGAGGA
AACGCAGGACCAATGTTTTAATACACGGTATTGTAATATACAAAATATAAATGGATAATGATATGTATTGTGAAACATAATATACGTGAAACATAATATATGTTTGATAATTTACATATT
ATAATATGTAAATTGTATACGAGTGTTGTATTTATAGAACATACAACACGCTATGACAAACAGGGAAAAATGAAGAATCGGGGGTTGATTGGTCTATCGTATCGCTTAAGGGCCGCAGGC
CCGTTGAAATGATTCTTAATAAAACAAATATACATGATACGGATAGTTGAATATATAAACAACGAAGTATAAATACAACAGAATGGTGTATACTAATTAAAATAATGTGAAGATAAGTAT
ATTTGTGCGGGATGATGATCACAACCACCACTTTAGTGGTGGGTCATATGTCCCGAGTTAGTGCGCCACGTAAGCGCTGGGGCTTAT

DNA-N = alyu47

>N_alyu48
TATTACCCCCCGTGCTCGGGACGGGACATGACGTCAGCATATATTATAATGGGCTTTTTAAAGCCCATATAAGTGAAGTGGGCCGGGTTTGAGACATATTTCGAAAGCCCGACCCGGAAA
AGGATAAAGTCACGTGCCGAATAATAGGTTGCTTCGCCTCGAAGCAAACTAATAAAAAGTTGCGTATTCAATACGCAACTAAGTCTATTAATAGGGTTGTCTCTGCCGAATAAATCAGAG
CGTATGCGAAGCAGAAGCGATGGATTGGGCAGAATCACAATTCAAGACATGTACCCATGGCTGTGATTGGAAGACGATATCATCGGATTCATCAGAAAATCGGCAATATGTACCTTGCGT
CGACTCTGGAGCTGGAAGAAAGACTCCTCGCAAGGTACTTCTTAGATCTATTGAAGCTGTGTTTAATGGAAGCTTCAAAGGGAATAATAGGAATGTTCGTGGCTTCTTATACGTATCAAT
CCGAGACGATGATGGAGCAATGCGTCCAGTACTTATAGTACCATTTGGAGGATATGGATATCATAATGATTTCTATTATTTCGAAGGGAAGGGGAAAGTTGAATGTGATATATCATCAGA
TTATGTTGCGCCAGAAGTCGATTGGAGCAGAGACATGGAAGTTAGTATTAGTAACAGCAACAACTGTAATGAATCATGTGATCTGAAGTGTTATGTTGTTTGTTCGTTAAGAATAAAGGA
ATAACAGATGTGCTGTAATGATTATTAATAAAACATATTTTTCATGCAATTGATAGTTGTATAAAACATACAACACGCTATGACAAACGGGGAAAAATGAAAAATCAGGGGTTGATTGTT
CTATAGTATCGCTTAAGGGCCGCAGGCCCGTTGAAAAATAATAATCGAATTATATACGATTGATAATAATCAGAGATAGATGATAAGGATATATAAACATAAACGAAGTATATGGCGGTA
TAATTAAACAGAAGCATATAATATAAAAATATGTAAACTAATCTCTGATTGGTGCAGAGTGTAGGTCCACTAACTTTAAGTTAGTGGAAATGTCCCGATGACGTAAGCACGGGGGACTAT

DNA-R 2 SNPs to alyu47

1 aa substitution in Rep

>R_alyu48
TATTACCCCCAGCGCTCGGGACGGGACATTTGCATCTATAAATAGACCTCCCCCCCCTCCACTACAAGATCATCATCGTCGACAGAAATGGCGCGATATGTGGTATGCTGGATGTTCACC
ATCAACAATCCCGCTTCACTACCAGTGATGCGGGATGAGTTCAAATATATGGTATATCAAGTGGAGAGGGGACAGGAGGGTACTCGTCATGTGCAGGGATACGTCGAGATGAAGAGACGA
AGTTCTCTGAAGCAGATGAGAGGCTTCTTCCCTGGCGCACACCTTGAGAAACGAAAGGGAAGCCAAGAAGAAGCACGGGCATACTGTATGAAGGAAGACACAAGAATCGAAGGTCCCTTC
GAGTTTGGTGCCTTTAAATTGTCATGTAATGATAATTTATTTGATGTCATACAGGATATGCGTGAAACGCACAAACGGCCTCTGGAGTATTTATATGAGTGTCCAAATACCTTCGATAGA
AGTAAGGATACATTATACAGAGTTCAAGCAGAGTTGAATAAAACGAAGGCGATGAATAGCTGGAAAACATCCTTCAGTTCATGGACATCGGAAGTTGAAACTATTATGGCGGAGCCATGT
CATCGAAGGATAATTTGGGTCTATGGCCCAAATGGAGGAGAAGGAAAGACAACTTATGCAAAATATTTAATGAAGACGAAGAATGCGTTTTATTCTCCAGGAGGAAAATCATTGGATATA
TGTAGATTGTATAATTACGAGGAAATAGTTATATTTGATATTCCCAGATGCAAAGAGGAATATTTAAACTATGGTTTATTAGAAGAATTTAAAAATGGAATTATTCAAAGCGGGAAATAT
GAACCCGTTTTGAAAATTGTAGAATATGTGGAAGTCATTGTAATGGCTAACTTCCTTCCGAAGGAAGGAATCTTTTCAGAAGATCGAATAAAGCTAGTTGCTTGCTGAACACGCTATGAC
AATCGTACGCTATGACAAAAGGGGAAAAGCAAAGATTCGGGGGTTGATTGGGCTATCCTAACGATTAAGGGCCGCAGGCCCGTCAAGATGGACGGCTTGATCAGATGTCCCGAGTTAGTG
CGCCACGTAAGCGCTGGGGCTTAT

DNA-S = alyu47

>S_alyu48
TATTACCCCCAGCGCTCGGGACGGGACATGGGCTAATGGATCATGGAATAAGGGCCCATCGGGCCCGTTAAGATGGGTTTTGGGCTTATGGGCTTTATCCAGAAGACCAAAAACAGGCGG
GAACCGTCCCAATTTCAAATTTAGATAGCTTGCCCCGCAAGCAAACTAAAAGCCTATATATACCAGTGTAGACATATTGTTCAGATAACAAATGGCGAGGTTTCCGAAGAAATCGATCAA

GAAGCGGCGGGTTGGGCGACGGAAGTATGGAAGCAAGGCGGCAACCAGTCACGACTACTCGTCGTTAGGTTCAATATTGGTTCCTGAAAATACCGTCAAGGTATTTAGGATTGAGCCTAC
TGATAAAACATTACCCAGATATTTTATCTGGAAAATGTTTATGCTTTTGGTGTGCAAGGTGAAGCCCGGAAGAATACTTCACTGGGCTATGATTAAGAGTTCATGGGAAATCAACCAGCC
GACTACATGTCTGGAAGCACCAGGTTTATTTATAAAACCTGAACATAGCCATCTGGTGAAACTGGTATGCAGTGGGGAACTTGAAGCCGGAGTCGCAACAGGGACATCAGATGTTGAATG
TCTTCTAAGGAAGACAACCGTGTTGAGGAAGAATGTAACAGAGGTGGATTACTTGTATTTGGCATTTTATTGTAGTGCTGGAGTTAGTATTAACTACCAGAACAGAATTACATATCATGT
ATGATATGTTTATGTAAACATAAACCTTTGTATGGAATAATGACCAAATAACATACAACACACTATGAAATACAACACGCTATGACAAACAGGGAAAAATGAAGAATCGGGGGTTGATTG
GTCTATCGTAACGCTTAAGGGCCGCAGGCCCGTTGAAATGATTCTTTATAAAACAAATATACATGATACGGATAGTTGAACATATAAACAACGATGTATAAATACAACAGAATGTTGTAT
ACTAATTAAAATAATGAGAAGAAAAGAATATTTGTGAAGGATAAGCATCAGAACCACCACTTTAGTGGTGGGTCAGATGTCCCGAGTTAGTGCGCCACGTAAGCGCTGGGGCTTAT

DNA-U3 v1 – 1 SNP to alyu47 / v2 - 2 SNPs to alyu47

ORF1 is identical in all

>U3_alyu48_v1
TATTACCCCCAGCCCCCAGGACGGGACATGGGCTTTTTAAATGGGCCGAGAGAGTTTGAACAGTTCAGTATCTTCGTTATTGGGCCATCTGGCCCAATAATTAAGAGAACGTGTTCAAAA
TCTGGGTTTGACCGAAGGTCAAGGTAGACGGTCAACAATATTCTGGCTTGCGGAGCAAGCAACAAGAATTAAATATTTAATTCGTATGACACGTGGACGGACCGAAATACTCCTGTATCT
CTATAAATAGCCTAAATCTGGCATGGATAATTGCTCTCGCTCTTCTGTCAAAGCTGTTGTGTTGAGGCGGAAGATCGCCATCGGCGATCATCAGACGAAAAGCTGCAAGAGAGACGGAGA
ACCATGCTGCGAAGCGTATATCGGGTATTTATAGACTTCTAGCGCAGCTAGAAGTTTCCTTGTACTTGTATTTGTATTTTGTAAATTACGAAGAAATTCGTACATTGATAATAATAAAAC
ATCTGGGATTGTTAATGTTTACATTAACTAGTATTAATAATGTACAATAACAAACATTGTATGAGGAACGAATACAAGAATATTAAAATATGAGGAGCGTAGCGTGATAAACAGGTGTTT
AAGGAATAATTAAAATAATTATGCAATGTTATGATAATACGGTATAAGTTGAAGTATGAGGTGAAAGAGGAGATATTAGAATATTAAAAACCCAATTATATTATTTATAATACATACAAC
ACGCTATGACAAAAGGGGAAAAAAGAAAAATCGGGGGTTGATTGGTCTATCCTAACGATTAAGGGCCGCAGGCCCGTCAAGATGGATGAACGGTTAGATTTGATTGCTTAGACACGAAGA
AAGGAATCTTTTTAGGACCACAGATAAGACAGCTGTCACTACTTTTAAAAAATAATATAATAACCAAATGACCATAGTGCCCCTCCCAAAGATGACGTATAGGGGTGTCCCGAGGTAATT
TAACATAGCTCTGACAAGAGATAGTGGACGTTGGATTCCACGATCGGACGATCGTGGTTTGATGAACTTATGaTGACGTAGGGGCTGGGGCTTAT

>U3_alyu48_v2
TATTACCCCCAGCCCCCAGGACGGGACATGGGCTTTTTAAATGGGCCGAGAGAGTTTGAACAGTTCAGTATCTTCGTTATTGGGCCATCTGGCCCAATAATTAAGAGAACGTGTTCAAAA
TCTGGGTTTGACCGAAGGTCAAGGTAGACGGTCAACAATATTCTGGCTTGCGGAGCAAGCAACAAGAATTAAATATTTAATTCGTATGACACGTGGACGGACCGAAATACTCCTGTATCT
CTATAAATAGCCTAAATCTGGCATGGATAATTGCTCTCGCTCTTCTGTCAAAGCTGTTGTGTTGAGGCGGAAGATCGCCATCGGCGATCATCAGACGAAAAGCTGCAAGAGAGACGGAGA
ACCATGCTGCGAAGCGTATATCGGGTATTTATAGACTTCTAGCGCAGCTAGAAGTTTCCTTGTACTTGTATTTGTATTTTGTAAATTACGAAGAAATTCGTACATTGATAATAATAAAAC
ATCTGGGATTGTTAATGTTTACATTAACTAGTATTAATAATGTACAATAACAAACATTGTATGAGGAACGAATACAAGAATATTAAAATATGAGGAGCGTAGCGTGATAAACAGGTGTTT
AAGGAATAATTAAAATAATTATGCAATGTTATGATAATACGGTATAAGTTGAAGTATGAGGTGAAAGAGGAGATATTAGAATATTAAAAACCCAATTATATTATTTATAATACATACAAC
ACGCTATGACAAAAGGGGAAAAAAGAAAAATCGGGGGTTGATTGGTCTATCCTAACGATTAAGGGCCGCAGGCCCGTCAAGATGGATGAACGGTTAGATTTGATTGCTTAGACACGAAGA
AAGGAATCTTTTTAGGACCACAGATAAGACAGCTGTCACTACTTTTAAAAAATAATATAATAACCAAATGACCATAGTGCCCCTCCCAAAGATGACGTATAGGGGTGTCCCGAGGTAATT
TAACATAGCTCTGACAAGAGATAGTGGACGTTGGATTCCACGATCGGACGATCGTGGTTTGATGAACTTATGcTGACGTAGGGGCTGGGGCTTAT

ALYU-49 - Musa sp. - Laos

Virome
1. **BBTV** six components (close to ALYU-47 and ALYU-48 at nearby locations)

DNA-C = alyu47 / 1 SNP to alyu48

>C_alyu49
TATTACCCCCAGCGCTCAGGACGGGACATCACGTGCATCTAACAAATGCACGTGACAAAGCAGTAGCTTGTAGCGAAAGATAACCATCACTATCACAAAAGTGAGAGGAATATTCGTTGC
TTAGGGACTAAGCAACGTGAACAGATATTTGTTCGAGAAGCGAAAATGGAGGCTATTTAAACCTGATGGTTTTGTGATTTCCGAAATCACTCATCGGAGCAGAAATGGAGTTCTGGGAAT
CGTCTGCCATGCCAGACGATGTCAAGAGAGTGATCAAGGAAATATATTGGGAGCATCGAAATAAACTTCTGTTCTGTCAGAAGTTGAAAGGCTGTGTAAGAAGGATTCTGGAGCATGGAA
ATCAAGATGATGCTCTAGACGCAGTGAAGGAGATGAAGACTTCTATTATTCGCTATAGCGAATATTTGAAGAAACCTTGTGTGGTATTGTGTTTTAGTACTAATAAATCTATTGCATATA
GGTTAAACACAATGGTGTTCTTTTATCATGAATATATTGAAGACCTAGGTGGTGACTATTCGGTATATCAAGATCTGTTCTGTGATGAGGAACTTCCTTATTCGTCGACAGAAGAAGAAG
ATGAAGAAGTAATATACAGGAATGTTATCATGGCATCGACAGAAGAGAAGATCTCTTGGAGTGAATGTCAAAAGATAGTTATATCAGATTATGATGTAACATTACTGTAATGAATATCCA
TTATCATAAATAAAATAATGGAATGATGATTATGTATTCTAGTATATATACATAATGGTATACGTATAGCATAAAATACATAAACCAACATACAACACGCTATGACAAACGGGGAAAAAT
GAAGAATCGGGGGTTGATTGGGCTATCTTAACGATTAAGGGCCGAAGGCCCGTTTAAATATGTGGTGGACGAAGTCCACAACACATAAAAAGTGCTCAGAACAAGGGAATATAATGAGCT
GGCAACGTAGGGACCATGTCCCGAGTTAGTGCGCCACGTAAGCGCTGGGGCTTAT

DNA-M 3 SNPs to alyu47 / 3 SNPs to alyu48

MP is identical to alyu47

>M_alyu49
TATTACCCCCAGCGCTCAGGACGGGACATCACGTGCAACTAACAAATGCACGTGACTGATATATACTACATAACGGTTTAACGAACCGTTATATAATGATATAACGAAAAGTCACGTGTG
ATAGAGACCTGCACGTGACATAGTCAAATGTATTGAATAAACATTTGACGTCCGGATGCTTCCGACGGAAGCTTAGGTTACTTCGTGGCGAAGCAAAACATTTATATATTGGCCTGGACT
GCTGCCTATAAATAGGCAAGCTAGGAAATGGCATTAACAACAGAGCGGGTGAAACTATTCTTTGAATGGTTTCTGTTCATCGGTGCAATATTTATTGCGATAACAATATTATATATATTG
TTGGTGTTGCTCTTTGAGGTCCCCAAGTATATTAAGGATGTTGTGAGGTATCTCGTAGAATACCTGACCAGAAGACGTGTATGGATGCAGAGAACGCAGTTGACGGAGGCAACAGGAGAT
GTAGAGCTCGGCAGAGGTATTGTGGAAGACAGACGGGATCAACAACCGGCTGTCATTCCACATGCAACACAGGTTATCCCTTCGCATCAACCTAGAAGGGATGATCAAGGAAGACGAGGA
AACGCAGGACCAATGTTTTAATACACGGTATTGTAATATACAAAATATAAATGGATAATGATATGTATTGTGAAACATAATATACGTGAAACATAATATATGTTTGATAATTTACATATT
ATAATATGTAAATTGTATACGAGTGTTGTATTTATAGAACATACAACACGCTATGACAAACAGGGAAAAATGAAGAATCGGGGGTTGATTGGTCTATCGTATCGCTTAAGGGCCGCAGGC
CCGTTGAAATGATTCTTAATAAAACAAATATACATGATACGGATAGTTGAATATATAAACAACGAAGTATAAATACAACAGAATGGTGTATACTAATTAAAATAATGTGAAGATAAGTAT
ATTTGTGCGGGATGATGATCACAACCACCACTTTAGTGGTGGGTCATATGTCCCGAGTTAGTGCGCCACGTAAGCGCTGGGGCTTAT

DNA-N 4 SNPs to alyu47

NSP is identical to alyu47

>N_alyu49
TATTACCCCCCGTGCTCGGGACGGGACATGACGTCAGCATATATTATAATGGGCTTTTTAAAGCCCATATAAGTGAAGTGGGCCGGGTTTGAGACATATTTCGAAAGCCCGACCCGGAAA
AGGATAAAGTCACGTGCCGAATAATAGGTTGCTTCGCCTCGAAGCAAACTAATAAAAAGTTGCGTATTCAATACGCAACTAAGTCTATTAATAGGGTTGTCTCTGCCGAATAAATCAGAG
CGTATGCGAAGCAGAAGCGATGGATTGGGCAGAATCACAATTCAAGACATGTACCCATGGCTGTGATTGGAAGACGATATCATCGGATTCATCAGAAAATCAGCAATATGTACCTTGCGT
CGACTCTGGAGCTGGAAGAAAGACTCCTCGCAAGGTACTTCTTAGATCTATTGAAGCTGTGTTTAATGGAAGCTTCAAAGGGAATAATAGGAATGTTCGTGGCTTCTTATACGTATCAAT
CCGAGACGATGATGGAGCAATGCGTCCAGTACTTATAATACCATTTGGAGGATATGGATATCATAATGATTTCTATTATTTCGAAGGGAAGGGGAAAGTTGAATGTGATATATCATCAGA
TTATGTTGCGCCAGAAGTCGATTGGAGCAGAGACATGGAAGTTAGTATTAGTAACAGCAACAACTGTAATGAATCATGTGATCTGAAGTGTTATGTTGTTTGTTCGTTAAGAATAAAGGA
ATAACAGATGTGCTGTAATGATTATTAATAAAACATATTTTTCATGCAATTGATAGTTGTATAAAACATACAACACGCTATGACAAACGGGGAAAAATGAAAAATCAGGGGTTGATTGTT
CTATAGTATCGCTTAAGGGCCGCAGGCCCGTTGAAAAATAATAATCGAATTATATACGATTGATAATAATCAGAGATAGATGATAAGGATATATATAAACATAAACGAAGTATATGGCGG
TATAATTAAACAGAAGCATATAATATAAAAATATGTAAACTAATCTCTGATTGGTGCAGAGTGTAGGTCCACTAACTTTAAGTTAGTGGAAATGTCCCGATGACGTAAGCACGGGGGACT
AT

DNA-R 4 SNPs to alyu47 / 4 SNPs to alyu48

Rep is identical to alyu47

>R_alyu49
TATTACCCCCAGCGCTCGGGACGGGACATTTGCATCTATAAATAGACCTCCCCCCTCTCCATTACAAGATCATCATCGTCGACAGAAATGGCGCGATATGTGGTATGCTGGATGTTCACC
ATCAACAATCCCGCTTCACTACCAGTGATGCGGGATGAGTTCAAATATATGGTATATCAAGTGGAGAGGGGACAGGAGGGTACTCGTCATGTGCAGGGATACGTCGAGATGAAGAGACGA
AGTTCTCTGAAGCAGATGAGAGGCTTCTTCCCTGGCGCACACCTTGAGAAACGAAAGGGAAGCCAAGAAGAAGCACGGGCATACTGTATGAAGGAAGACACAAGAATCGAAGGTCCTTTC
GAGTTTGGTGCCTTTAAATTGTCATGTAATGATAATTTATTTGATGTCATACAGGATATGCGTGAAACGCACAAACGGCCTCTGGAGTATTTATATGAGTGTCCAAATACCTTCGATAGA
AGTAAGGATACATTATACAGAGTTCAAGCAGAGTTGAATAAAACGAAGGCGATGAATAGCTGGAAAACATCCTTCAGTTCATGGACATCGGAAGTTGAAAATATTATGGCGGAGCCATGT

CATCGAAGGATAATTTGGGTCTATGGCCCAAATGGAGGAGAAGGAAAGACAACTTATGCAAAATATTTAATGAAGACGAAGAATGCGTTTTATTCTCCAGGAGGAAAATCATTGGATATA
TGTAGATTGTATAATTACGAGGAAATAGTTATATTTGATATTCCCAGATGCAAAGAGGAATATTTAAACTATGGTTTATTAGAAGAATTTAAAAATGGAATTATTCAAAGCGGGAAATAT
GAACCCGTTTTGAAAATTGTAGAATATGTGGAAGTCATTGTAATGGCTAACTTCCTTCCGAAGGAAGGAATCTTTTCAGAAGATCGAATAAAGCTAGTTGCTTGCTGAACACGCTATGAC
AATCGTACGCTATGACAAAAGGGGAAAAGCAAAGATTCGGGGGTTGATTGGGCTATCCTAACGATTAAGGGCCGCAGGCCCGTCAAGATGGACGGCTTGATCAGATGTCCCGAGTTAGTG
CGCCACGTAAGCGCTGGGGCTTAT

DNA-S = alyu47 = alyu48

>S_alyu49
TATTACCCCCAGCGCTCGGGACGGGACATGGGCTAATGGATCATGGAATAAGGGCCCATCGGGCCCGTTAAGATGGGTTTTGGGCTTATGGGCTTTATCCAGAAGACCAAAAACAGGCGG
GAACCGTCCCAATTTCAAATTTAGATAGCTTGCCCCGCAAGCAAACTAAAAGCCTATATATACCAGTGTAGACATATTGTTCAGATAACAAATGGCGAGGTTTCCGAAGAAATCGATCAA
GAAGCGGCGGGTTGGGCGACGGAAGTATGGAAGCAAGGCGGCAACCAGTCACGACTACTCGTCGTTAGGTTCAATATTGGTTCCTGAAAATACCGTCAAGGTATTTAGGATTGAGCCTAC
TGATAAAACATTACCCAGATATTTTATCTGGAAAATGTTTATGCTTTTGGTGTGCAAGGTGAAGCCCGGAAGAATACTTCACTGGGCTATGATTAAGAGTTCATGGGAAATCAACCAGCC
GACTACATGTCTGGAAGCACCAGGTTTATTTATAAAACCTGAACATAGCCATCTGGTGAAACTGGTATGCAGTGGGGAACTTGAAGCCGGAGTCGCAACAGGGACATCAGATGTTGAATG
TCTTCTAAGGAAGACAACCGTGTTGAGGAAGAATGTAACAGAGGTGGATTACTTGTATTTGGCATTTTATTGTAGTGCTGGAGTTAGTATTAACTACCAGAACAGAATTACATATCATGT
ATGATATGTTTATGTAAACATAAACCTTTGTATGGAATAATGACCAAATAACATACAACACACTATGAAATACAACACGCTATGACAAACAGGGAAAAATGAAGAATCGGGGGTTGATTG
GTCTATCGTAACGCTTAAGGGCCGCAGGCCCGTTGAAATGATTCTTTATAAAACAAATATACATGATACGGATAGTTGAACATATAAACAACGATGTATAAATACAACAGAATGTTGTAT
ACTAATTAAAATAATGAGAAGAAAAGAATATTTGTGAAGGATAAGCATCAGAACCACCACTTTAGTGGTGGGTCAGATGTCCCGAGTTAGTGCGCCACGTAAGCGCTGGGGCTTAT

DNA-U3 1 SNP to alyu48 and alyu47_v1

ORF1 is identical

>U3_alyu49
TATTACCCCCAGCCCCCAGGACGGGACATGGGCTTTTTAAATGGGCCGAGAGAGTTTGAACAGTTCAGTATCTTCGTTATTGGGCCATCTGGCCCAATAATTAAGAGAACGTGTTCAAAA
TCTGGGTTTGACCGAAGGTCAAGGTAGACGGTCAACAATATTCTGGCTTGCGGAGCAAGCAACAAGAATTAAATATTTAATTCGTATGACACGTGGACGGACCGAAATACTCCTGTATCT
CTATAAATAGCCTAAATCTGGCATGGATAATTGCTCTCGCTCTTCTGTCAAAGCTGTTGTGTTGAGGCGGAAGATCGCCATCGGCGATCATCAGACGAAAAGCTGCAAGAGAGACGGAGA
ACCATGCTGCGAAGCGTATATCGGGTATTTATAGACTTCTAGCGCAGCTAGAAGTTTCCTTGTACTTGTATTTGTATTTTGTAAATTACGAAGAAATTCGTACATTGATAATAATAAAAC
ATCTGGGATTGTTAATGTTTACATTAACTAGTATTAATAATGTACAATAACAAACATTGTATGAGGAACGAATACAAGAATATTAAAATATGAGGAGCGTAGCGTGATAAACAGGTGTTT
AAGGAATAATTAAAATAATTATGCAATGTTATGATAATACGGTATAAGTTGAAGTATGAGGTGAAAGAGGAGATATTAGAATATTAAAAACCCAATTATATTATTTATAATACATACAAC
ACGCTATGACAAAAGGGGAAAAAAGAAAAATCGGGGGTTGATTGGTCTATCCTAACGATTAAGGGCCGCAGGCCCGTCAAGATGGATGAACGGTTAGATTTGATTGCTTAGACACGAAGA
AAGGAATCTTTTTGGGACCACAGATAAGACAGCTGTCACTACTTTTAAAAAAATAATATAATAACCAAATGACCATAGTGCCCCTCCCAAAGATGACGTATAGGGGTGTCCCGAGGTAATT
TAACATAGCTCTGACAAGAGATAGTGGACGTTGGATTCCACGATCGGACGATCGTGGTTTGATGAACTTATGATGACGTAGGGGCTGGGGCTTAT

ALYU-50 - Musa AA sucrier Kouay niew mung - Laos

Virome
1. **BBTV** six components (close to ALYU-47, ALYU-48 and ALYU-49 at nearby locations)

DNA-C 2 SNPs to alyu47

Clink is identical

>C_alyu50
TATTACCCCCAGCGCTCAGGACGGGACATCACGTGCATCTAACAAATGCACGTGACAAAGCAGTAGCTTGTAGCGAAAGATAACCATCACTATCACAAAAGTGAGAGGAATATTCGTTGC
TTAGGGACTAAGCAACGTGAACAGATATTTGTTCGAGAAGCGAAAATGGAGGCTATTTAAACCTGATGGTTTTGTGATTTCCGAAATCACTCATCGGAGCAGAAATGGAGTTCTGGGAAT
CGTCTGCCATGCCAGACGATGTCAAGAGAGTGATCAAGGAAATATATTGGGAGCATCGAAATAAACTTCTGTTCTGTCAGAAGTTGAAAGGCTGTGTAAGAAGGATTCTGGAGCATGGAA
ATCAAGATGATGCTCTAGACGCAGTGAAGGAGATGAAGACTTCTATTATTCGCTATAGCGAATATTTGAAGAAACCTTGTGTGGTATTGTGTTTTAGTACTAATAAATCTATTGCATATA
GGTTAAACACAATGGTGTTCTTTTATCATGAATATATTGAAGACCTAGGTGGTGACTATTCGGTATATCAAGATCTGTTCTGTGATGAGGAACTTCCTTATTCGTCGACAGAAGAAGAAG
ATGAAGAAGTAATATACAGGAATGTTATCATGGCATCGACAGAAGAGAAGATCTCTTGGAGTGAATGTCAAAAGATAGTTATATCAGATTATGATGTAACATTACTGTAATGAATATCCA
TTATCATTAATAAAATAATGGAATGATGATTATGTATTTTAGTATATATACATAATGGTATACGTATAGCATAAAATACATAAACCAACATACAACACGCTATGACAAACGGGGAaAAAT
GAAGAATCGGGGGTTGATTGGGCTATCTTAACGATTAAGGGCCGAAGGCCCGTTTAAATATGTGGTGGACGAAGTCCACAACACATAAAAAGTGCTCAGAACAAGGGAATATAATGAGCT
GGCAACGTAGGGACCATGTCCCGAGTTAGTGCGCCACGTAAGCGCTGGGGCTTAT

DNA-M 1 SNP to alyu47

MP is identical

>M_alyu50
TATTACCCCCAGCGCTCAGGACGGGACATCACGTGCAACTAACAAATGCACGTGACTGATATATACTACATAACGGTTTAACGAACCGTTATATAATGTTATAACGAAAAGTCACGTGTG
ATAGAGACCTGCACGTGACATAGTCAAATGTATTGAATAAACATTTGACGTCCGGATGCTTCCGACGGAAGCTTAGGTTACTTCGTGGCGAAGCAAAACATTTATATATTGGCCTGGACT
GCTGCCTATAAATAGGCAAGCTAGGAAATGGCATTAACAACAGAGCGGGTGAAACTATTCTTTGAATGGTTTCTGTTCATCGGTGCAATATTTATTGCGATAACAATATTATATATATTG
TTGGTGTTGCTCTTTGAGGTCCCCAAGTATATTAAGGATGTTGTGAGGTATCTCGTAGAATACCTGACCAGAAGACGTGTATGGATGCAGAGAACGCAGTTGACGGAGGCAACAGGAGAT
GTAGAGCTCGGCAGAGGTATTGTGGAAGACAGACGGGATCAACAACCGGCTGTCATACCACATGCAACACAGGTTATCCCTTCGCATCAACCTAGAAGGGATGATCAAGGAAGACGAGGA
AACGCAGGACCAATGTTTTAATACACGGTATTGTAATATACAAAATATAAATGGATAATGATATGTATTGTGAAACATAATATACGTGAAACATAATATATGTTTGATAATTTACATATT
ATAATATGTAAATTGTATACGAGTGTTGTATTTATAGAACATACAACACGCTATGACAAACAGGGAAAAATGAAGAATCGGGGGTTGATTGGTCTATCGTATCGCTTAAGGGCCGCAGGC
CCGTTGAAATGATTCTTAATAAAACAAATATACATGATACGGATAGTTGAATATATAAACAACGAAGTATAAATACAACAGAATGGTGTATACTAATTAAAATAATGTGAAGATAAGTAT
ATTTGTGCGGGATGATGATCACAACCACCACTTTAGTGGTGGGTCATATGTCCCGAGTTAGTGCGCCACGTAAGCGCTGGGGCTTAT

DNA-N = alyu47

>N_alyu50
TATTACCCCCCGTGCTCGGGACGGGACATGACGTCAGCATATATTATAATGGGCTTTTTAAAGCCCATATAAGTGAAGTGGGCCGGGTTTGAGACATATTTCGAAAGCCCGACCCGGAAA
AGGATAAAGTCACGTGCCGAATAATAGGTTGCTTCGCCTCGAAGCAAACTAATAAAAAGTTGCGTATTCAATACGCAACTAAGTCTATTAATAGGGTTGTCTCTGCCGAATAAATCAGAG
CGTATGCGAAGCAGAAGCGATGGATTGGGCAGAATCACAATTCAAGACATGTACCCATGGCTGTGATTGGAAGACGATATCATCGGATTCATCAGAAAATCGGCAATATGTACCTTGCGT
CGACTCTGGAGCTGGAAGAAAGACTCCTCGCAAGGTACTTCTTAGATCTATTGAAGCTGTGTTTAATGGAAGCTTCAAAGGGAATAATAGGAATGTTCGTGGCTTCTTATACGTATCAAT
CCGAGACGATGATGGAGCAATGCGTCCAGTACTTATAGTACCATTTGGAGGATATGGATATCATAATGATTTCTATTATTTCGAAGGGAAGGGGAAAGTTGAATGTGATATATCATCAGA
TTATGTTGCGCCAGAAGTCGATTGGAGCAGAGACATGGAAGTTAGTATTAGTAACAGCAACAACTGTAATGAATCATGTGATCTGAAGTGTTATGTTGTTTGTTCGTTAAGAATAAAGGA
ATAACAGATGTGCTGTAATGATTATTAATAAAACATATTTTTCATGCAATTGATAGTTGTATAAAACATACAACACGCTATGACAAACGGGGAAAAATGAAAAATCAGGGGTTGATTGTT
CTATAGTATCGCTTAAGGGCCGCAGGCCCGTTGAAAAATAATAATCGAATTATATACGATTGATAATAATCAGAGATAGATGATAAGGATATATAAACATAAACGAAGTATATGGCGGTA
TAATTAAACAGAAGCATATAATATAAAAATATGTAAACTAATCTCTGATTGGTGCAGAGTGTAGGTCCACTAACTTTAAGTTAGTGGAAATGTCCCGATGACGTAAGCACGGGGGACTAT

DNA-R 2 SNPs to alyu47

Rep is identical

>R_alyu50
TATTACCCCCAGCGCTCGGGACGGGACATTTGCATCTATAAATAGACCTCCCCCCCCTCCACTACAAGATCATCATCGTCGACAGAAATGGCGCGATATGTGGTATGCTGGATGTTCACC
ATCAACAATCCCGCTTCACTACCAGTGATGCGGGATGAGTTCAAATATATGGTATATCAAGTGGAGAGGGGACAGGAGGGTACTCGTCATGTGCAGGGATACGTTGAGATGAAGAGACGA
AGTTCTCTGAAGCAGATGAGAGGCTTCTTCCCTGGCGCACACCTTGAGAAACGAAAGGGAAGCCAAGAAGAAGCACGGGCATACTGTATGAAGGAAGACACAAGAATCGAAGGTCCCTTC
GAGTTTGGTGCCTTTAAATTGTCATGTAATGATAATTTATTTGATGTCATACAGGATATGCGTGAAACGCACAAACGGCCTCTGGAGTATTTATATGAGTGTCCAAATACCTTCGATAGA
AGTAAGGATACATTATACAGAGTTCAAGCAGAGTTGAATAAAACGAAGGCGATGAATAGCTGGAAAACATCCTTCAGTTCATGGACATCGGAAGTTGAAAATATTATGGCGGAGCCATGT
CATCGAAGGATAATTTGGGTCTATGGCCCAAATGGAGGAGAAGGAAAGACAACTTATGCAAAATATTTAATGAAGACGAAGAATGCGTTTTATTCTCCAGGAGGAAAATCATTGGATATA
TGTAGATTGTATAATTACGAGGAAATAGTTATATTTGATATTCCCAGATGCAAAGAGGAATATTTAAACTATGGTTTATTAGAAGAATTTAAAAATGGAATTATTCAAAGCGGGAAATAT

GAACCCGTTTTGAAAATTGTAGAATATGTGGAAGTCATTGTAATGGCTAACTTCCTTCCGAAGGAAGGAATCTTTTCAGAAGATCGAATAAAGCTAGTTGCTTGCTGAACACGCTATGAC
AATCGTACGCTATGACAAAAGGGGAAAAGCAAAGATTCGGGGGTTGATTGGGCTATCCTAACGATTAAGGGCCGCAGGCCCGTCAAGATGGACGGCTTGATCAGATGTCCCGAGTTAGTG
CGCCACGTAAGCGCTGGGGCTTAT

DNA-S = alyu47 = alyu48 = alyu49

>S_alyu50
TATTACCCCCAGCGCTCGGGACGGGACATGGGCTAATGGATCATGGAATAAGGGCCCATCGGGCCCGTTAAGATGGGTTTTGGGCTTATGGGCTTTATCCAGAAGACCAAAAACAGGCGG
GAACCGTCCCAATTTCAAATTTAGATAGCTTGCCCCGCAAGCAAACTAAAAGCCTATATATACCAGTGTAGACATATTGTTCAGATAACAAATGGCGAGGTTTCCGAAGAAATCGATCAA
GAAGCGGCGGGTTGGGCGACGGAAGTATGGAAGCAAGGCGGCAACCAGTCACGACTACTCGTCGTTAGGTTCAATATTGGTTCCTGAAAATACCGTCAAGGTATTTAGGATTGAGCCTAC
TGATAAAACATTACCCAGATATTTTATCTGGAAAATGTTTATGCTTTTGGTGTGCAAGGTGAAGCCCGGAAGAATACTTCACTGGGCTATGATTAAGAGTTCATGGGAAATCAACCAGCC
GACTACATGTCTGGAAGCACCAGGTTTATTTATAAAACCTGAACATAGCCATCTGGTGAAACTGGTATGCAGTGGGGAACTTGAAGCCGGAGTCGCAACAGGGACATCAGATGTTGAATG
TCTTCTAAGGAAGACAACCGTGTTGAGGAAGAATGTAACAGAGGTGGATTACTTGTATTTGGCATTTTATTGTAGTGCTGGAGTTAGTATTAACTACCAGAACAGAATTACATATCATGT
ATGATATGTTTATGTAAACATAAACCTTTGTATGGAATAATGACCAAATAACATACAACACACTATGAAATACAACACGCTATGACAAACAGGGAAAAATGAAGAATCGGGGGTTGATTG
GTCTATCGTAACGCTTAAGGGCCGCAGGCCCGTTGAAATGATTCTTTATAAAACAAATATACATGATACGGATAGTTGAACATATAAACAACGATGTATAAATACAACAGAATGTTGTAT
ACTAATTAAAAtAATGAGAAGAAAAGAATATTTGTGAAGGATAAGCATCAGAACCACCACTTTAGTGGTGGGTCAGATGTCCCGAGTTAGTGCGCCACGTAAGCGCTGGGGCTTAT

DNA-U3 1 SNP to alyu47

ORF1 is identical

>U3_alyu50
TATTACCCCCAGCCCCCAGGACGGGACATGGGCTTTTTAAATGGGCCGAGAGAGTTTGAACAGTTCAGTATCTTCGTTATTGGGCCATCTGGCCCAATAATTAAGAGAACGTGTTCAAAA
TCTGGGTTTGACCGAAGGTCAAGGTAGACGGTCAACAATATTCTGGCTTGCGGAGCAAGCAACAAGAATTAAATATTTAATTCGTATGACACGTGGACGGACCGAAATACTCCTGTATCT
CTATAAATAGCCTAAATCTGGCATGGATAATTGCTCTCGCTCTTCTGTCAAAGCTGTTGTGTTGAGGCGGAAGATCGCCATCGGCGATCATCAGACGAAAAGCTGCAAGAGAGACGGAGA
ACCATGCTGCGAAGCGTATATCGGGTATTTATAGACTTCTAGCGCAGCTAGAAGTTTCCTTGTACTTGTATTTGTATTTTGTAAATTACGAAGAAATTCGTACATTGATAATAATAAAAC
ATCTGGGATTGTTAATGTTTACATTAACTAGTATTAATAATGTACAATAACAAACATTGTATGAGGAACGAATACAAGAATATTAAAATATGAGGAGCGTAGCGTGATAAACAGGTGTTT
AAGGAATAATTAAAATAATTATGCAATGTTATGATAATACGGTATAAGTTGAAGTATGAGGTGAAAGAGGAGATATTAGAATATTAAAAACCCAATTATATTATTTATAATACATACAAC
ACGCTATGACAAAAGGGGAAAAAAGAAAAATCGGGGGTTGATTGGTCTATCCTAACGATTAAGGGCCGCAGGCCCGTCAAGATGGATGAACGGTTAGATTTGATTGCTTAGACACGAAGA
AAGGAATCTTTTTGGGACCACAGATAAGACAGCTGTCACTACTTTTAAAAAATAATATAATAACCAAATGACCATATTGCCCCTCCCAAAGATGACGTATAGGGGTGTCCCGAGGTAATT
TAACATAGCTCTGACAAGAGATAGTGGACGTTGGATTCCACGATCGGACGATCGTGGTTTGATGAACTTATGATGACGTAGGGGCTGGGGCTTAT

ALYU-51 - Musa ABB Pisang Awak ? - Laos

Virome
1. **BBTV** six components (close to ALYU-47, ALYU-48, ALYU-49 and ALYU-50 at nearby locations)

DNA-C 1 SNP to alyu47

Clink is identical

>C_alyu51
TATTACCCCCAGCGCTCAGGACGGGACATCACGTGCATCTAACAAATGCACGTGACAAAGCAGTAGCTTGTAGCGAAAGATAACCATCACTATCACAAAAGTGAGAGGAATATTCGTTGC
TTAGGGACTAAGCAACGTGAACAGATATTTGTTCGAGAAGCGAAAATGGAGGCTATTTAAACCTGATGGTTTTGTGATTTCCGAAATCACTCATCGGAGCAGAAATGGAGTTCTGGGAAT
CGTCTGCCATGCCAGACGATGTCAAGAGAGTGATCAAGGAAATATATTGGGAGCATCGAAATAAACTTCTGTTCTGTCAGAAGTTGAAAGGCTGTGTAAGAAGGATTCTGGAGCATGGAA
ATCAAGATGATGCTCTAGACGCAGTGAAGGAGATGAAGACTTCTATTATTCGCTATAGCGAATATTTGAAGAAACCTTGTGTGGTATTGTGTTTTAGTACTAATAAATCTATTGCATATA
GGTTAAACACAATGGTGTTCTTTTATCATGAATATATTGAAGACCTAGGTGGTGACTATTCGGTATATCAAGATCTGTTCTGTGATGAGGAACTTCCTTATTCGTCGACAGAAGAAGAAG
ATGAAGAAGTAATATACAGGAATGTTATCATGGCATCGACAGAAGAGAAGATCTCTTGGAGTGAATGTCAAAAGATAGTTATATCAGATTATGATGTAACATTACTGTAATGAATATCCA
TTATCATAAATAAAATAATGGAATGATGATTATGTATTCTAGTATATATACATAATGGTATACGTATAGCATAAAATACATAAACCAACATACAACACGCTATGACAAACGGGGAAAAAT
GAAGAATCGGGGGTTGATTGGGCTATCTTAACGATTAAGGGCCGAAGGCCCGTTTAAATATGTGGTGGACGAAGTCCACAACACATAAAAAGTGCTAAGAACAAGGGAATATAATGAGCT
GGCAACGTAGGGACCATGTCCCGAGTTAGTGCGCCACGTAAGCGCTGGGGCTTAT

DNA-M 1 SNP to alyu47

MP is identical

>M_alyu51
TATTACCCCCAGCGCTCAGGACGGGACATCACGTGCAACTAACAAATGCACGTGACTGATATATACTACATAACGGTTTAACGAACCGTTATATAATGTTATAACGAAAAGTCACGTGTG
ATAGAGACCTGCACGTGACATAGTCAAATGTATTGAATAAACATTTGACGTCCGGATGCTTCCGACGGAAGCTTAGGTTACTTCGTGGCGAAGCAAAACATTTATATATTGGCCTGGACT
GCTGCCTATAAATAGGCAAGCTAGGAAATGGCATTAACAACAGAGCGGGTGAAACTATTCTTTGAATGGTTTCTGTTCATCGGTGCAATATTTATTGCGATAACAATATTATATATATTG
TTGGTGTTGCTCTTTGAGGTCCCCAAGTATATTAAGGATGTTGTGAGGTATCTCGTAGAATACCTGACCAGAAGACGTGTATGGATGCAGAGAACGCAGTTGACGGAGGCAACAGGAGAT
GTAGAGCTCGGCAGAGGTATTGTGGAAGACAGACGGGATCAACAACCGGCTGTCATACCACATGCAACACAGGTTATCCCTTCGCATCAACCTAGAAGGGATGATCAAGGAAGACGAGGA
AACGCAGGACCAATGTTTTAATACACGGTATTGTAATATACAAAATATAAATGGATAATGATATGTATTGTGAAACATAATATACGTGAAACATAATATATGTTTGATAATTTACATATT
ATAATATGTAAATTGTATACGAGTGTTGTATTTATAGAACATACAACACGCTATGACAAACAGGGAAAAATGAAGAATCGGGGGTTGATTGGTCTATCGTATCGCTTAAGGGCCGCAGGC
CCGTTGAAATGATTCTTAATAAAACAAATATACATGATACGGATAGTTGAATATATAAACAACGAAGTATAAATACAACAGAATGGTGTATACTAATTAAAATAATGTGAAGATAAGTAT
ATTTGTGCGGGATGATGATCACAACCACCACTTTAGTGGTGGGTCATATGTCCCGAGTTAGTGCGCCACGTAAGCGCTGGGGCTTAT

DNA-N 2 SNP to alyu47

NSP is identical

>N_alyu51
TATTACCCCCCGTGCTCGGGACGGGACATGACGTCAGCATATATTATAATGGGCTTTTTAAAGCCCATATAAGTGAAGTGGGCCGGGTTTGAGACATATTTCGAAAGCCCGACCCGGAAA
AGGATAAAGTCACGTGCCGAATAATAGGTTGCTTCGCCTCGAAGCAAACTAATAAAAAGTTGCGTATTCAATACGCAACTAAGTCTATTAATAGGGTTGTCTCTGCCGAATAAATCAGAG
CGTATGCGAAGCAGAAGCGATGGATTGGGCAGAATCACAATTCAAGACATGTACCCATGGCTGTGATTGGAAGACGATATCATCGGATTCATCAGAAAATCGGCAATATGTACCTTGCGT
CGACTCTGGAGCTGGAAGAAAGACTCCTCGCAAGGTACTTCTTAGATCTATTGAAGCTGTGTTTAATGGAAGCTTCAAAGGGAATAATAGGAATGTTCGTGGCTTCTTATACGTATCAAT
CCGAGACGATGATGGAGCAATGCGTCCAGTACTTATAGTACCATTTGGAGGATATGGATATCATAATGATTTCTATTATTTCGAAGGGAAGGGGAAAGTTGAATGTGATATATCATCAGA
TTATGTTGCGCCAGAAGTCGATTGGAGCAGAGACATGGAAGTTAGTATTAGTAACAGCAACAACTGTAATGAATCATGTGATCTGAAGTGTTATGTTGTTTGTTCGTTAAGAATAAAGGA
ATAACAGATGTGCTGTAATGATTATTAATAAAACATATTTTTCATGTAATTGATAGTTGTATAAAACATACAACACGCTATGACAAACGGGGAAAAATGAAAAATCAGGGGTTGATTGTT
CTATAGTATCGCTTAAGGGCCGCAGGCCCGTTGAAAAATAATAATCGAATTATATACGATTGATAATAATCAGAGATAGATGATAAGGATATATAAACATAAACGAAGTATATGGCGGTA
TAATTAAACAGAAGCATATAATATAAAAATATGTAAACTAATCTCTGATTGGTGCAGAGTGTAGGTCCACTACCTTTAAGTTAGTGGAAATGTCCCGATGACGTAAGCACGGGGGACTAT

DNA-R 1 SNP to alyu47

Rep is identical

>R_alyu51
TATTACCCCCAGCGCTCGGGACGGGACATTTGCATCTATAAATAGACCTCCCCCCCCTCCACTACAAGATCATCATCGTCGACAGAAATGGCGCGATATGTGGTATGCTGGATGTTCACC
ATCAACAATCCCGCTTCACTACCAGTGATGCGGGATGAGTTCAAATATATGGTATATCAAGTGGAGAGGGGACAGGAGGGTACTCGTCATGTGCAGGGATACGTCGAGATGAAGAGACGA
AGTTCTCTGAAGCAGATGAGAGGCTTCTTCCCTGGCGCACACCTTGAGAAACGAAAGGGAAGCCAAGAAGAAGCACGGGCATACTGTATGAAGGAAGACACAAGAATCGAAGGTCCCTTC
GAGTTTGGTGCCTTTAAATTGTCATGTAATGATAATTTATTTGATGTCATACAGGATATGCGTGAAACGCACAAACGGCCTCTGGAGTATTTATATGAGTGTCCAAATACCTTCGATAGA
AGTAAGGATACATTATACAGAGTTCAAGCAGAGTTGAATAAAACGAAGGCGATGAATAGCTGGAAAACATCCTTCAGTTCATGGACATCGGAAGTTGAAAATATTATGGCGGAGCCATGT
CATCGAAGGATAATTTGGGTCTATGGCCCAAATGGAGGAGAAGGAAAGACAACTTATGCAAAATATTTAATGAAGACGAAGAATGCGTTTTATTCTCCAGGAGGAAAATCATTGGATATA
TGTAGATTGTATAATTACGAGGAAATAGTTATATTTGATATTCCCAGATGCAAAGAGGAATATTTAAACTATGGTTTATTAGAAGAATTTAAAAATGGAATTATTCAAAGCGGGAAATAT

GAACCCGTTTTGAAAATTGTAGAATATGTGGAAGTCATTGTAATGGCTAACTTCCTTCCGAAGGAAGGAATCTTTTCAGAAGATCGAATAAAGCTAGTTGCTTGCTGAACACGCTATGAC
AATCGTACGCTATGACAAAAGGGGAAAAGCAAAGATTCGGGGGTTGATTGGGCTATCCTAACGATTAAGGGCCGCAGGCCCGTCAAGATGGACGGCTTGATCAGATGTCCCGAGTTAGTG
CGCCACGTAAGCGCTGGGGCTTAT

DNA-S = alyu47

>S_alyu51
TATTACCCCCAGCGCTCGGGACGGGACATGGGCTAATGGATCATGGAATAAGGGCCCATCGGGCCCGTTAAGATGGGTTTTGGGCTTATGGGCTTTATCCAGAAGACCAAAAACAGGCGG
GAACCGTCCCAATTTCAAATTTAGATAGCTTGCCCCGCAAGCAAACTAAAAGCCTATATATACCAGTGTAGACATATTGTTCAGATAACAAATGGCGAGGTTTCCGAAGAAATCGATCAA
GAAGCGGCGGGTTGGGCGACGGAAGTATGGAAGCAAGGCGGCAACCAGTCACGACTACTCGTCGTTAGGTTCAATATTGGTTCCTGAAAATACCGTCAAGGTATTTAGGATTGAGCCTAC
TGATAAAACATTACCCAGATATTTTATCTGGAAAATGTTTATGCTTTTGGTGTGCAAGGTGAAGCCCGGAAGAATACTTCACTGGGCTATGATTAAGAGTTCATGGGAAATCAACCAGCC
GACTACATGTCTGGAAGCACCAGGTTTATTTATAAAACCTGAACATAGCCATCTGGTGAAACTGGTATGCAGTGGGGAACTTGAAGCCGGAGTCGCAACAGGGACATCAGATGTTGAATG
TCTTCTAAGGAAGACAACCGTGTTGAGGAAGAATGTAACAGAGGTGGATTACTTGTATTTGGCATTTTATTGTAGTGCTGGAGTTAGTATTAACTACCAGAACAGAATTACATATCATGT
ATGATATGTTTATGTAAACATAAACCTTTGTATGGAATAATGACCAAATAACATACAACACACTATGAAATACAACACGCTATGACAAACAGGGAAAAATGAAGAATCGGGGGTTGATTG
GTCTATCGTAACGCTTAAGGGCCGCAGGCCCGTTGAAATGATTCTTTATAAAACAAATATACATGATACGGATAGTTGAACATATAAACAACGATGTATAAATACAACAGAATGTTGTAT
ACTAATTAAAATAATGAGAAGAAAAGAATATTTGTGAAGGATAAGCATCAGAACCACCACTTTAGTGGTGGGTCAGATGTCCCGAGTTAGTGCGCCACGTAAGCGCTGGGGCTTAT

DNA-U3 1 SNP to alyu47

ORF1 is identical

>U3_alyu51
TATTACCCCCAGCCCCCAGGACGGGACATGGGCTTTTTAAATGGGCCGAGAGAGTTTGAACAGTTCAGTATCTTCGTTATTGGGCCATCTGGCCCAATAATTAAGAGAACGTGTTCAAAA
TCTGGGTTTGACCGAAGGTCAAGGTAGACGGTCAACAATATTCTGGCTTGCGGAGCAAGCAACAAGAATTAAATATTTAATTCGTATGACACGTGGACGGACCGAAATACTCCTGTATCT
CTATAAATAGCCTAAATCTGGCATGGATAATTGCTCTCGCTCTTCTGTCAAAGCTGTTGTGTTGAGGCGGAAGATCGCCATCGGCGATCATCAGACGAAAAGCTGCAAGAGAGACGGAGA
ACCATGCTGCGAAGCGTATATCGGGTATTTATAGACTTCTAGCGCAGCTAGAAGTTTCCTTGTACTTGTATTTGTATTTTGTAAATTACGAAGAAATTCGTACATTGATAATAATAAAAC
ATCTGGGATTGTTAATGTTTACATTAACTAGTATTAATAATGTACAATAACAAACATTGTATGAGGAACGAATACAAGAATATTAAAATATGAGGAGCGTAGCGTGATAAACAGGTGTTT
AAGGAATAATTAAAATAATTATGCAATGTTATGATAATACGGTATAAGTTGAAGTATGAGGTGAAAGAGGAGATATTAGAATATTAAAAACCCAATTATATTATTTATAATACATACAAC
ACGCTATGACAAAAGGGGAAAAAAGAAAAATCGGGGGTTGATTGGTCTATCCTAACGATTAAGGGCCGCAGGCCCGTCAAGATGGATGAACGGTTAGATTTGATTGCTTAGACACGAAGA
AAGGAATCTTTTTGGGACCACAGATAAGACAGCTGTCACTACTTTTAAAAAATAATATAATAACCAAATGACCATAGTGCCCCTCCCAAAGATGACGTATAGGGGTGTCCCGAGGTAATT
TAACATAGCTCTGATAAGAGATAGTGGACGTTGGATTCCACGATCGGACGATCGTGGTTTGATGAACTTATGATGACGTAGGGGCTGGGGCTTAT

ALYU-52 - Musa acuminata wild - China

Virome
BBTV free - all six components at a cross-contamination level (Supplementary Figure S3H); Babu2 200 reads
Badnavirus BSVNV partial sequence (mapping revealed a new isolate of BSVNV with ca. 90% identity to ALYU-33)

ALYU-53 – Musa yunnanensis – China

Virome
1. **BBTV** six components (two variants of DNA-C and DNA-M: v1 > v2)

DNA-C
12-13 SNPs to KM607099 KM607098 (**China**). **23-27 SNPs** to KY427060 MF039870 MF039864 MF039876 (**Thailand**)

C protein (161 aa): v1 **2 aa** to KM607098 KM607099 (**China**) **3 aa** to KY427060 (**Thailand**) **4 aa** to MF039864 (**Thailand**). v2: 1 aa to v1

>C_alyu53_v1
TATTACCCCCCCTGCTCGGGACGGGACATCACGTGCTTCTAACAAATGCACGTGACAAAGCAGTAGCTTGTAGCGAAAGATAACCATCACTTTCACAAAAGTGAAAGGAATATTCGTTGC
TTAGGGACTAAGCAACGAGGaCAGATATTTGTTCGAGAAGCGACAATGGAGGCTATTTAAACCTGATGGTTTTGTGATTTCCGAAATCACTCATCGGAGCAGAAATGGAGTTCTGGGAAT
CGTCTGCTATGCCAGAcGATGTCAAGAGAGTGATCAAGGAAATATATTGGGAGCATCGAAATAAACTTCTGTTTTGTCAGAAGTTGAAAGGCTGTGTAAGAAGGATTCTGGAGCATGGAA
ATCAAGATGATGCTCTAGACGCAGTGAAGGAGATGAAGACTTCTATTATTCGCTATAGCGAATATTTGAAGAAACCTTGTGTGGTATTGTGTTTTAGTACTAATAAATCTATTGTATATA
GGTTAAACACAATGGTGTTCTTTTATCATGAATATATTGAAGACCTAGGTGGTGACTACtCGGTATATCAAGATCTGTTTTGTGATGAGGAACTTCCTTCGTCTTCGACAGAAGAAGAAG
ATGAAGAAGTAATATACAGGAATGTTATCATGGCATCGACAGAAGAGAAGATCTCTTGGAGTGAATGTCAAAAGATAGTTATATCAGATTATGATGTAACATTACTGTAATGAATATCCA
TTATCATAAATAAAATAATGGAATGATGATTATGTATTCTAGTATAAATACATAATGGTATACGTATAGCATAAAATACATAAACCAACATACAACACACTATGACAAACAGGGAAAAAT
GAAGAATCGGGGGTTGATTGGGCTATCTTAACGATTAAGGGCCGAAGGCCCGTTTAAATATGTGGTGGACGAAGTCCACAACACAAAAAAGTGATCAGAACAATGGAATATAATGAGCTG
GCAATGTAGGGACCATGTCCCGAGTTAGTGCGCCACGTGAGCAGGGGGGCTTAT

A/T (159758/57046) T/A (80706/48469)

>C_alyu53_v2
TATTACCCCCCCTGCTCGGGACGGGACATCACGTGCTTCTAACAAATGCACGTGACAAAGCAGTAGCTTGTAGCGAAAGATAACCATCACTTTCACAAAAGTGAAAGGAATATTCGTTGC
TTAGGGACTAAGCAACGAGGtCAGATATTTGTTCGAGAAGCGACAATGGAGGCTATTTAAACCTGATGGTTTTGTGATTTCCGAAATCACTCATCGGAGCAGAAATGGAGTTCTGGGAAT
CGTCTGCTATGCCAGAcGATGTCAAGAGAGTGATCAAGGAAATATATTGGGAGCATCGAAATAAACTTCTGTTTTGTCAGAAGTTGAAAGGCTGTGTAAGAAGGATTCTGGAGCATGGAA
ATCAAGATGATGCTCTAGACGCAGTGAAGGAGATGAAGACTTCTATTATTCGCTATAGCGAATATTTGAAGAAACCTTGTGTGGTATTGTGTTTTAGTACTAATAAATCTATTGTATATA
GGTTAAACACAATGGTGTTCTTTTATCATGAATATATTGAAGACCTAGGTGGTGACTACaCGGTATATCAAGATCTGTTTTGTGATGAGGAACTTCCTTCGTCTTCGACAGAAGAAGAAG
ATGAAGAAGTAATATACAGGAATGTTATCATGGCATCGACAGAAGAGAAGATCTCTTGGAGTGAATGTCAAAAGATAGTTATATCAGATTATGATGTAACATTACTGTAATGAATATCCA
TTATCATAAATAAAATAATGGAATGATGATTATGTATTCTAGTATAAATACATAATGGTATACGTATAGCATAAAATACATAAACCAACATACAACACACTATGACAAACAGGGAAAAAT
GAAGAATCGGGGGTTGATTGGGCTATCTTAACGATTAAGGGCCGAAGGCCCGTTTAAATATGTGGTGGACGAAGTCCACAACACAAAAAAGTGATCAGAACAATGGAATATAATGAGCTG
GCAATGTAGGGACCATGTCCCGAGTTAGTGCGCCACGTGAGCAGGGGGGCTTAT

DNA-M
16/21 SNPs to KM607239, KM607240 (**China**). **53-54 SNPs** to KY427061 MF039865 MF039871 MF039876 (**Thailand**)

M protein (117 aa): 3 aa to KC581796 (**Thailand**) **4 aa** to KM607239 (**China**) MF039871 MF039865 MF039877 KY427061 (**Thailand**)
v2: 1 aa to v1

>M_alyu53_v1
TATTACCCCCAGCGCTCAGGACGGGACATCACGTGCAACTAACAAATGCACGTGACTGATATATACTACATAACGGTTTAACGAACCGTTATATAATGTTATAACGAAAAGTCACGTGAG
ATAGAGACCTGCACGTGACATAGTCAAATGTATTGAATAAACATTTGACGTCCGGATGCTTCCGACGGAAGCTTAGGTTACTTCGTGGCGAAGCAAAACATTTATATATTGGCCTGGACT
GCTGCCTATAAATAGGCAAGCTAGGTAATGGCATTAACAACAGAGCGGGTGAAACTATTCTTTGAATGGTTTCTGTTCATCGGTTCAATATTTATTGCGATAACAATATTATATATATTG
TTGGCGTTGCTCTTTGAGGTCCCCAAGTATATTAAGGATGTTGTGAGGTATCTCGTAGAATACCTGACCAGAAGACGTGTATGGATGCAGAGAACGCAGTTGACGGAGGCAACAGGAGAT
GTAGAGCTCGGCAGAGGTAGTGTGGAAGACAGACGGGATCAACAACCGGCTGTCATACCACATGCAACACAGGTTATCCCTTCGCATCAACCTAGAAGGGAAGATCAAGGAAGACGAGGA
AACGCAGGACCAATGTTTTAATACACGGTATTGTAATATACGAAATATAAATGGATAATGATATGTATTGTGAAACATAATATACGTGAAACATAATATATGTTTGATAATTTACATATT
ATAATATGTAAATTGTATACGAGTGTTGTATTTATAGAACATACAACACGCTATGACAAACAGGGAAAAATGAAGAATCGGGGGTTGATTGGTCTATCGTATCGCTTAAGGGCCGCAGGC
CCGTTGAAATGATTCTTAATAAAACAAATATACATGATACGGATAGTTGAATATATAAACAACGAAGTATAAATACAACAGAATGTTGTATACTAATTAAAATAATGAGAAGATAAGTAT
ATTTGTGAGGGATGATGATCACAACCACCACTTTAGTGGTGGGTCATATGTCCCGAGTTAGTGCGCCACGTAAGCGCTGGGGCTTAT

C/G (62472/28164)

>M_alyu53_v2

TATTACCCCCAGCGCTCAGGACGGGACATCACGTGCAACTAACAAATGCACGTGACTGATATATACTACATAACGGTTTAACGAACCGTTATATAATGTTATAACGAAAAGTCACGTGAG
ATAGAGACCTGCACGTGACATAGTCAAATGTATTGAATAAACATTTGACGTCCGGATGCTTCCGACGGAAGCTTAGGTTACTTCGTGGCGAAGCAAAACATTTATATATTGGCCTGGACT
GCTGCCTATAAATAGGCAAGCTAGGTAATGGCATTAACAACAGAGCGGGTGAAACTATTCTTTGAATGGTTTCTGTTCATCGGTTCAATATTTATTGCGATAACAATATTATATATATTG
TTGGCGTTGCTCTTTGAGGTCCCCAAGTATATTAAGGATGTTGTGAGGTATgTCGTAGAATACCTGACCAGAAGACGTGTATGGATGCAGAGAACGCAGTTGACGGAGGCAACAGGAGAT
GTAGAGCTCGGCAGAGGTAGTGTGGAAGACAGACGGGATCAACAACCGGCTGTCATACCACATGCAACACAGGTTATCCCTTCGCATCAACCTAGAAGGGAAGATCAAGGAAGACGAGGA
AACGCAGGACCAATGTTTTAATACACGGTATTGTAATATACGAAATATAAATGGATAATGATATGTATTGTGAAACATAATATACGTGAAACATAATATATGTTTGATAATTTACATATT
ATAATATGTAAATTGTATACGAGTGTTGTATTTATAGAACATACAACACGCTATGACAAACAGGGAAAAATGAAGAATCGGGGGTTGATTGGTCTATCGTATCGCTTAAGGGCCGCAGGC
CCGTTGAAATGATTCTTAATAAAACAAATATACATGATACGGATAGTTGAATATATAAACAACGAAGTATAAATACAACAGAATGTTGTATACTAATTAAAATAATGAGAAGATAAGTAT
ATTTGTGAGGGATGATGATCACAACCACCACTTTAGTGGTGGGTCATATGTCCCGAGTTAGTGCGCCACGTAAGCGCTGGGGCTTAT

DNA-N
11-13 SNPs to KM607387 KM607385 KM607386 (**China**). **25-18 SNPs** to MF039872 MF039878 MF039866 KY427062 (**Thailand**)

N protein (154 aa): 0 aa to KM607387 KM607385 (**China**) MF039872 MF039878 KY427062 (**Thailand**) **1 aa** to MF039866 (**Thailand**)

>N_alyu53
TATTACCCCCCGTGCTCGGGACGGGACATGACGTCAGCATAGATTATAATGGGCCAACTAAAGGCCCATTTAATAGAAAAGGCGGGCTTTTGACATATTTCAAAGGCCCAGCCTGGAAGT
GGATAATGTCACGTGCCGAATAAGAGGTTGCTTCGCCTCGAAGCAATCCGAATAAATGTTGCGTATTCAATACGCAACTGAAGTCTATTAATATGGTTGTCTCTGCCGAATAAATCAGAG
CGTAAGCGAAGCAGAAGCGATGGATTGGGCAGAATCACAATTCAAGACCTGTACTCATGGGTGCGATTGGAAGACGATATCATCGGATTCATCCGAGAATCGACAATATGTACCTTGCGT
CGACGCAGGCAGTGGAAGAAAGTCACCTCGCAAGGTACTGCTTAGATCTATTGAAGCTGTGTTTAATGGAAGCTTCAAAGGAAATAACAGGAATGTTCGTGGGTTTATATACGTATCAAT
ACGAGACGATGATGGAGAAATGCGTCCAGTACTCATAATACCATTCGGAGGATATGGATATCATAATGATTTCTATTATTTCGAAGGGAAGGGGAAAGTTGAATGTGATATATCATCAGA
TTATGTAGCGCCAGGAGTCGATTGGAGCAGAGACATGGAAGTTAGTATTAGCAACAGCAACAACTGTAATGAATTATGTGATCTGAAGTGTTATGTTGTTTGTTCGTTAAGAATAAAGGA
ATAACAGATGTGCTGTAATGATTATTAATAAAACTTATTTTCATGTACTTGATAGTTGTATAAAACATACAACACGCTATGACAAACAGGGAAAAATGAAAAATCGGGGTTTGATTGTTC
TATAGTATCGCTTAAGGGCCGCAGGCCCGTTGAAAAATAATAATCGAATTATAAACGTTAGATAATAATCAGAGATAGATGATAAGCATCAAAAAACATAGACGAAGTATATGGCTGTAT
AATATAAAAGAAGCATATAAATAAATAAATGTGAACTAATCTCTGATTGGTTCAGAACGTAGCCCCACCAACTTTATGTTGGTGGAAATGTCCCGATGACGTAAGCACGGGGGACTAT

DNA-R
10-12 SNPs to KM607677 KM607678 KM607676 KM607679 (**China**). **11-17 SNPs** to AB113660 AF416475 AB113659 (**Viet Nam**). **16-20 SNPs** to MF039879 MF039873 MF039867 KY427063 (**Thailand**)

R protein (286 aa): 0 aa to KM607676 KM607677 KM607679 (**China**) **1 aa** to AB113660 (**Vietnam**) MF039879 (**Thailand**) KM607678 (**China**) …

>R_alyu53
TATTACCCCCAGCGCTCGGGACGGGACATTTGCATCTATAAATAGACCTCCCCCCTCTCCACTACAAGATCATCATCGTCGACAGAAATGGCGCGATATGTGGTATGCTGGATGTTCACC
ATCAACAATCCCGCTTCACTACCAGTGATGCGGGATGAGTTCAAATATATGGTATATCAAGTGGAGAGGGGACAGGAGGGTACTCGTCATGTGCAGGGATACGTCGAGATGAAGAGACGA
AGTTCTCTGAAGCAGATGAGAGGCTTCTTCCCTGGCGCACACCTTGAGAAACGAAAGGGAAGCCAAGAAGAAGCACGGGCATACTGTATGAAGGAAGACACACGAATCGAAGGTCCCTTC
GAGTTTGGTGCCTTTAAATTGTCATGTAATGATAATTTATTTGATGTCATACAGGATATGCGTGAAACGCACAAACGGCCTTTGGAGTATTTATATGAGTGTCCAAATACCTTCGATAGA
AGTAAGGATACATTATACAGAGTTCAAGCAGAGTTGAATAAAACGAAGGCGATGAATAGCTGGAAAACATCCTTCAGTTCTTGGACATCGGAAGTTGAAAATATTATGGCGGAGCCATGT
CATCGGAGGATAATTTGGGTCTATGGCCCAAATGGAGGAGAAGGAAAGACAACTTATGCAAAATATTTAATGAAGACGAAGAATGCGTTTTATTCTCCAGGAGGAAAATCATTGGATATA
TGTAGATTGTATAATTACGAGGAAATAGTTATATTTGATATTCCCAGATGCAAAGAGGAATATTTAAATTATGGTTTATTAGAAGAATTTAAAAATGGAATTATTCAAAGCGGGAAATAT
GAACCCGTTTTGAAAATTGTAGAATATGTGGAAGTCATTGTAATGGCTAACTTCCTTCCGAAGGAAGGAATCTTTTCAGAAGATCGAATAAAGCTAGTTGCTTGCTGAACACGCTATGAC
AATCGTACGCTATGACAAAAGGGGAAAAGCAAAGATTCGGGGGTTGATTGGGCTATCCTAACGATTAAGGGCCGCAGGCCCGTCAAGATGGACGGCTCGATCAGATGTCCCGAGTTAGTG
CGCCACGTAAGCGCTGGGGCTTAT

DNA-S
14-16 SNPs to KM607534 KM607535 KM607536, KM607533 (**China**). **17-20 SNPs** to AB113662 AF148945 AB113661 (**Viet Nam**). **21-33 SNPs** to MF039874 KY427064 MF039880 (**Thailand**)

S protein (170 aa): 0 aa to AY337715 AY494786 HQ378191 (**China**) FJ787433 FJ787434 (**Philippines**) **1 aa** to several isolates

>S_alyu53
TATTACCCCCAGCGCTCGGGACGGGACATGGGCTAATGGATCATGGAATAAGGGCCCATCGGGCCCGTTAGGATGGGTTTTGGGCTGATGGGCTTTATCCAGAAGACCAAAAACAGGCGG
GAACCGTCCCAATTTCAAATTTAGATAGCTTGCCCCGCAAGCAAACTAAAAGTCTATATATACCAGTGTAGACATATTGTTCAGATAACAAATGGCGAGGTTTCCGAAGAAATCGATCAA
GAAGAGGCGGGTTGGGCGACGGAAGTATGGAAGCAAGGCGGCAACCAGTCACGACTACTCGTCGTTAGGTTCAATATTGGTTCCTGAAAATACCGTCAAGGTATTTAGGATTGAGCCTAC
TGATAAAACATTACCCAGATATTTTATCTGGAAAATGTTTATGCTTTTGGTGTGCAAGGTGAAGCCCGGAAGAATACTTCACTGGGCTATGATTAAAAGTTCATGGGAAATCAACCAGCC
GACTACATGTCTGGAAGCACCAGGTTTATTTATAAAACCTGAACATAGCCATCTGGTGAAACTGGTATGCAGTGGGGAACTTGAAGCCGGAGTCGCAACAGGGACATCAGATGTTGAATG
TCTTCTAAGGAAGACAACCGTGTTGAGGAAGAATGTAACAGAGGTGGATTACTTGTATTTGGCATTTTATTGTAGTGCTGGAGTTAGTATTAACTACCAGAACAGAATTACATATCATGT
ATGATATGTTTATGTAAACATAAACCTTTGTATGGAATAATGACCAAATAACATACAACACACTATGAAATACAACACGCTATGACAAACAGGGAAAAATGAAGAATCGGGGGTTGATTG
GTCTATCGTATCGCTTAAGGGCCGCAGGCCCGTTGAAATGATTCTTTATAAAACAAATATACATGATACGGATAGTTGAATATATAAACAACGATGTATAAATACAACAGAATGTTGTAT
ACTAATTAAACAAATGAGAAGAAAAGGATATTTGTGAAGGATAAGCATCAGAACCACCACTTTAGTGGTGGGTCAGATGTCCCGAGTTAGTGCGCCACGTAAGCGCTGGGGCTTAT

DNA-U3
32-42SNPs to MF039875 MF039881 KY427065 MF039869 (**Thailand**)

U3 protein (39 aa): 0 aa to KY427065 (**Thailand)**

>U3_alyu53
TATTACCCCCAGCCCCCAGGACGGGACATGGGCTTTTTAAATGGGCCGAGAGAGTTTGAACAGTTCAGTATCTTCGTTATTGGGCCATCTGGCCCAATAATTAAGAGAACGTGTTCAAAA
TCTGGGTTTGACCGAAGGTCAAGGTAGACGGTCAACAATATTCTGGCTTGCGGAGCAAGCAACAAGAATTAAATATTTAATTCGTATGACACGTGGACGGACCGAAATACTCCTGTATCT
CTATAAATAGCCTAAATCTGCTATGGATAATTGCTCTCGCTCTTCTGTCAAAGGTGTTGTGTTGAGGCGGAAGATCGCCATCGGCGATCATCAGACGAAAAGCTGCAAGAGAGACGGAGA
ACCATGCTGCGAAGCGTATATCGGGTATTTATAGACTTATAGCGCAGCTAGAAGTATACTTGTACTTGATATTGTATTTTGTAAATTACGAAGAAATTCGTATTGATAATAATAAAACAT
CTGGGATTGTTAATGTTTACATTAACTAGTATTAATAATGTACAATAACAAAAATTGTATGAGGAACGAATACAAGAATATTAAAATAAGAGGAGCGTAGCGTGATAAACAGGTGTTTAA
GGTATAATTAAAATAATTATGCAAATGTCATGATAATACGGTATAAGTTGAAGTATGAGGTGAAAGAGGAGATATTAGAATATTAAAAACCCAATTATATTATTTATAATACATACAACA
CGCTATGACAAAAGGGGAAAAATGAAGAATCGGGGGTTGATTGGTCTATCCTAACGATTAAGGGCCGCAGGCCCGTCAAGATGGATGAACGGTTAGATTTGATTGCTTAGCCACGAAGAA
AGGAATCTTTTTGGGACCACAGATAAGACAGCTGTCACTACCTTTAAAAATAATATAATAACCAATTGACGATAGTACCCCTCCCAAAGATGACGTATAGGGGTGTCCCGAGGTAATTTA
ACATAGCTCTGACAAGAGATAGTGGACGTTGGATGCCACGATCGGACGATCGTGGTTTGATGAACTTATGATGACGTAGGGGCTGGGGCTTAT

ALYU-54 - Musa AAA Cavendish - China

Virome
1. **BBTV** six components (two variants of DNA-U3: v1 > v2)
2. **BBTA6** alyu54
3. **BBTA2** alyu54
4. **BBTA6def** 690 nt circle (**deletion in Rep ORF**)

BBTA6
BlastN preferentially finds matches to short sequences of BBTV U (including a 150 nt region upstream of the SL-CR)

>BBTA6_alyu54
TATTACCCGACCTTGGCGCAGGGCGCACTATAAAAGATGTCTGTGAGAAATTGGGTCTTCACACGCAATTTCGAAGGCGAAAGACCAGAGCTCCTGTTTGGCGGCGAAACTCAATACGCC
GCTTGGCAACATGAAAGAAAGAATCACGACCACCTACAGGGCGTGATTCAGTTGAAGAAGAAGACCCGACTGAACGCAGTGAAGAGCATAATCGGTGGAAATCCACATGTCGAACCCATG
CGAGCGTCTTTCGACGATGCCCTAGCATACGTCATGAAAGACGAAACCCGAATCGATGGTCCATGGGAATTCGGTATTCGAATCCGTAAGGGTTCGAATAAACGAAAGGTGTTAGAAATA
CTGGAGGATTCGGACAACGAAGTCCTTCAACCCCAAAAATACAGACGAGCCATGGCCAAACAGGCCATGAGTGAGTCTAAGAAGAAAGCTGCAGAGAAGGATTTTGTTATGAACTGCGA

GAGTGGCAAGTGCAATTGACGAAGTTGTTGGAAGAAGAACCCGATAACAGAACAATAATATGGGTATATGGACCAAAGGGTGGAGAAGGTAAAACAGAGTTCGCTAAACATTTAGGGTTA
AAGGATGGTTGGACATATTTGCCCGGTGGTGAACTCAAAGATATGATGTACCTGTTATCAAAGGACATGAAAAATAATGTTGTAATTGATTTCCCCCGTTGTACCAAGGAATTTATTTCA
TATAAATTCCTTGAGATGGTTAAGAATAGAACTATATATAGTTATAAGTATGAACCAGTTGGTTCAATCGTATGTAACAAGGTTCATGTAGTGGTCTTCAGTAATGAGGAGCCAGATTAT
GTAAAATTATCATTGGATCGAATAAAGTTGATTTCTTGCTGAACACGCTATGAAATACAACACGCTATGACAAACGGGGAAAAAAGAAAAATCGGGGGTTGATTGGTCTATCCTAACGAT
TAAGGGCCGCAGGCCCGTCAAGATGGATGAACGGTCAGAATTGATTGCTTCGCCACGAAGCAAACAAAAAAATGCGTGGTGGAGAATTCGCCACGTCATTGGTTACTGTTATCCTTGGCGC
ACTATATATATAGGTGCGCCGAGGTCGGCCATAG

BBTV6def

Defective BBTA6_alyu54 derivative with a 422 nt deletion

>BBTA6def_alyu54
TATTACCCGACCTTGGCGCAGGGCGCACTATAAAAGATGTCTGTGAGAAATTGGGTCTTCACACGCAATTTCGAAGGCGAAAGACCAGAGCTCCTGTTTGGCGGCGAAACTCAATACGCC
GCTTGGCAACATGAAAGAAAGAATCACGACCACCTACAGGGCGTGATTCAGTTGAAGAAGAAGACCCGACTGAACGCAGTGAAGAGCATAATCGGTGGAAATCCACATGTCGTTATCAAA
GGACATGAAAAATAATGTTGTAATTGATTTCCCCCGTTGTACCAAGGAATTTATTTCATATAAATTCCTTGAGATGGTTAAGAATAGAACTATATATAGTTATAAGTATGAACCAGTTGG
TTCAATCGTATGTAACAAGGTTCATGTAGTGGTCTTCAGTAATGAGGAGCCAGATTATGTAAAATTATCATTGGATCGAATAAAGTTGATTTCTTGCTGAACACGCTATGAAATACAACA
CGCTATGACAAACGGGGAAAAAAGAAAAATCGGGGGTTGATTGGTCTATCCTAACGATTAAGGGCCGCAGGCCCGTCAAGATGGATGAACGGTCAGAATTGATTGCTTCGCCACGAAGCA
AACAAAAAAATGCGTGGTGGAGAATTCGCCACGTCATTGGTTACTGTTATCCTTGGCGCACTATATATATAGGTGCGCCGAGGTCGGCCATAG

BBTA2

Best match to EU430730 100% coverage 97% identity; MG545616 100% coverage 97% identity; NC_038953 98% coverage 95% identity

Rep protein best matches to AZL93963 100% coverage 99% identities (2 aa) 100% positives; ACB86656 100% coverage 99% identities (3 aa) 99% positives; YP_009508281 100% coverage 94% identities (11 gaps in middle) 94% positives Banana bunchy top alphasatellite 2

>Babu2_alyu54
TATTACCCACCTCCGCGCACCACCTCCGCGCACCTATAAAATGTCTGCCTCTCGCTGGACATTTACGCTTCATTATTCCGACGCAACGGAGCGAGGCAAATTCCTCGCGACTTTGAAGGA
GGAAGATGTGCACTACGCCGTCGTCGGCGACGAAACTGCTCCGAATACTGGTCGAAAACATCTTCAAGGATATCTTTCCTTGAAGAAACGTTTTCGTATTAGCGGCATAAAGAAGAAATA
TTCGTCGAGAGCGCATTGGGAGAAAGCTCGAGGATCAGATTACGACAACAAGGCGTACTGCTCAAAAGAAGCCCTAATTCTTGAATTAGGGGTTCCTTGCCAAACAGGTTCGAATAAGCG
TAAATTATCAGATATGGTTACAAGATCGCCGGAACGAATGAAAATTGAACAGCCAGAGATATTTCACCGATACGAATCGGTGAAGAAGATGAAAGAATTCAAAGAAAGGTATGTCTATCC
TATCCTCGATAGGCCATGGCAGGTACAATTAACGGAGTTAATTGAAGCAGAACCGGATGATCGAACGATCATCTGGGTATTCGGACCAAAAGGGAATGAAGGCAAATCAACGTACGCGAA
GTCATTAATCCAAAAGGATTGGTTCTACACCAGGGGAGGAAAGAAGGAGAACATACTGTTCGCCTACGTAGATGAAGGTTCGACAAAAAACGTTGTATTTGATCTTCCGCGTACAGTACA
AGAATTTATTAATTATGATGTTATCGAAGCACTGAAGGATAGAGTAATCGAGAGTACAAAATACAAGCCTGTGAAGTATTTAGAATTGAATACTGTACATGTAATAGTTATGGCTAATTT
TCTTCCTGATATGTGTAAAATATCTGAAGATCGAATAAAAATAGTTGCTTGCTGAACACGCTATGAAATACAACACGCTATGACAAACGGGGAAAAAAGAAAAATCGGGGGTTGATTGGT
CTATCCTAACGATTAAGGGCCGCAGGCCCGTCAAGATGGATCCAATAACCCGTTAAGAAGGTAAACGGGTCTAAAACGATTTCTTCGCCCGCAAGCAACACCTTTAACCTCTGCGCACCT
ATATATAGCGGAGGTGGGCTAG

DNA-C

15 SNPs to KM607099 KM607098 (**China**). **24-28 SNPs** to KY427060 MF039870 MF039864 MF039876 (**Thailand**)

C protein (161 aa): 2 aa to KM607098 KM607099 (**China**) **3 aa** to KY427060 (**Thailand**) **4 aa** to MF039864 (**Thailand**)

>C_alyu54
TATTACCCCCCCTGCTCGGGACGGGACATCACGTGCTTCTAACAAATGCACGTGACAAAGCAGTAGCTTGTAGCGAAAGATAACCATCACTTTCACAAAAGTGAAAGGAATATTCGTTGC
TTAGGGACTAAGCAACGAGGACAGATATTTGTTCGAGAAGCGAAAATGGAGGCTATTTAAACCTGATGGTTTTGTGATTTCCGAAATCACTCATCGGAGCAGAAATGGAGTTCTGGGAAT
CGTCTGCTATGCCAGACGATGTCAAGAGAGTGATCAAGGAAATATATTGGGAGCATCGAAATAAACTTCTGTTCTGTCAGAAGTTGAAAGGCTGTGTAAGAAGGATTCTGGAGCATGGAA
ATCAAGATGATGCTCTAGACGCAGTGAAGGAGATGAAGACTTCTATTATTCGCTATAGTGAATATTTGAAGAAACCTTGTGTGGTATTGTGTTTTAGTACTAATAAATCTATTGCATATA
GGTTAAACACAATGGTGTTCTTTTATCATGAATATATTGAAGACCTAGGTGGTGACTACTCGGTATATCAAGATCTGTTTTGTGATGAGGAACTTCCTTCGTCTTCGACAGAAGAAGAAG
ATGAAGAAGTAACATACAGGAATGTTATCATGGCATCGACAGAAGAGAAGATCTCTTGGAGTGAATGTCAAAAGATAGTTATATCAGATTATGATGTAACATTACTGTAATGAATATCCA
TTATCATAAATAAAATAATGGAATGATGATTATGTATTCTAGTATAAATACATAATggtatACGTATAGCATAAAATACATAAACCTACATACAACACACTATGACAAACAGGGAAAAAT
GAAGAATCGGGGGTTGATTGGGCTATCTTAACGATTAAGGGCCGAAGGCCCGTTTAAATATGTGGTGGACGAAGTCCACAACACAAAAAAAGTGATCAGAACAATGGAATATTATGAGCT
GGCAACGTAGGGACCATGTCCCGAGTTAGTGCGCCACGTGAGCAGGGGGGCTTAT

DNA-M

16-21 SNPs to KM607239, KM607240 (**China**). **52-54 SNPs** to MF03986 KY427061 MF039871 MF039876 (**Thailand**)

M protein (117 aa): 2 aa to MF039865 (**Thailand**) MF039877 (**Thailand**) **3 aa** to KC581796 (**Thailand**)

>M_alyu54
TATTACCCCCAGCGCTCAGGACGGGACATCACGTGCAACTAACAAATGCACGTGACTGATATATACTACATAACGGTTTAACGAACCGTTATATAATGTTATAACGAAAAGTCACGTGAG
ATAGAGACCTGCACGTGACATAGTCAAATGTATTGAATAAACATTTGACGTCCGGATGCTTCCGACGGAAGCTTAGGTTACTTCGTGGCGAAGCAAAACATTTATATATTGGCCTGGACT
GCTGCCTATAAATAGGCAAGCTAGGTAATGGCATTAACAACAGAGCGGGTGAAACTATTCTTTGAATGGTTTCTGTTCATCGGTGCAATATTTATTGCGATAACAATATTATATATATTG
TTGGTGTTGCTCTTTGAGGTCCCCAAGTATATTAAGGATGTTGTGAGGTATCTCGTAGAATACCTGACCAGAAGACGTGTATGGATGCAGAGAACGCAGTTGACGGAGGCAACAGGAGAT
GTAGAGCTCGGCAGAGGTAGTGTGGAAGACAGACGGGATCAACAACCGGCTGTCATACCACATGCAACACAGGTTATCCCTTCGCATCAACCTAGAAGGGAAGATCAAGGAAGACGAGGA
AACGCAGGACCAATGTTTTAATACACGGTATTGTAATATACGAAATATAAATGGATAATGATATGTATTGTGAAACATAATATACGTGAAACATAATATATGTTTGATAATTTACATATT
ATAATATGTAAATTGTATACGAGTGTTGTATTTATAGAACATACAACACGCTATGACAAACAGGGAAAAATGAAGAATCGGGGGTTGATTGGTCTATCGTATCGCTTAAGGGCCGCAGGC
CCGTTGAAATGATTCTTAATAAAACAAATATACATGATACGGATAGTTGAATATATAAACAACGAAGTATAAATACAACAGAATGTTGTATACTAATTAAAATAATGAGAAGATAAGTAT
ATTTGTGAGGGATGATGATCACAACCACCACTTTAGTGGTGGGTCATATGTCCCGAGTTAGTGCGCCACGTAAGCGCTGGGGCTTAT

DNA-N

2-14 SNPs to KM607387 KM607385 KM607386 (**China**). **26-30 SNPs** to MF039872 MF03987 MF039866 KY427062 (**Thailand**)

N protein (154 aa): 0 aa to KM607387 KM607385 (**China**) MF039872 MF039878 KY427062 (**Thailand**) **1 aa** to MF039866 (**Thailand**)

>N_alyu54
TATTACCCCCCGTGCTCGGGACGGGACATGACGTCAGCATAGATTATAATGGGCCAACTAAAGGCCCATTTAATAGACAAGGCGGGCTTTTGACATATTTCAAAGGCCCAGCCTGGAAGT
GGATAATGTCACGTGCCGAATAAGAGGTTGCTTCGCCTCGAAGCAATCCGAATAAATGTTGCGTATTCAATACGCAACTGAAGTCTATTAATATGGTTGTCTCTGCCGAATAAATCAGAG
CGTAAGCGAAGCAGAAGCGATGGATTGGGCAGAATCACAATTCAAGACCTGTACTCATGGGTGCGATTGGAAGACGATATCATCGGATTCATCCGAGAATCGACAATATGTACCTTGCGT
CGACGCAGGCAGTGGAAGAAAGTCACCTCGCAAGGTACTGCTTAGATCTATTGAAGCTGTGTTTAATGGAAGCTTCAAAGGAAATAACAGGAATGTTCGTGGGTTTATATACGTATCAAT
ACGAGACGATGATGGAGAAATGCGTCCAGTACTCATAATACCATTCGGAGGATATGGATATCATAATGATTTCTATTATTTCGAAGGGAAGGGGAAAGTTGAATGTGATATATCATCAGA
TTATGTAGCGCCAGGAGTCGATTGGAGCAGAGACATGGAAGTTAGTATTAGCAACAGCAACAACTGTAATGAATTATGTGATCTGAAGTGTTATGTTGTTTGTTCGTTAAGAATAAAGGA
ATAACAGATGTGCTGTAATGATTATTAATAAAACTTATTTTCATGTAATTGATAGTTGTATAAAACATACAACACGCTATGACAAACAGGGAAAAATGAAAAATCGGGGTTTGATTGTTC
TATAGTATCGCTTAAGGGCCGCAGGCCCGTTGAAAAATAATAATCGAATTATAAACGTTAGATAATAATCAGAGATAGATGATAAgCATCAAAAAACATAGACGAAGTATATGGCTGTAT
AATATAAAAGAAGCATATAAATAAATAAATGTGAACTAATCTCTGATTGGTTCAGAACGTAGACCCACCAACTTTATGTTGGTGGAAATGTCCCGATGACGTAAGCACGGGGGACTAT

DNA-R

10-12 SNPs to KM607677 KM607678 KM607676 KM607679 (**China**). **10-17 SNPs** to AB113660 AB113659 (**Viet Nam**). **15 SNPs** to AF416475 (**Viet Nam**). **16-20 SNPs** to MF039879 MF039873 MF039867 KY427063 (**Thailand**)

R protein (286 aa): 1 aa to KM607676 KM607677 KM607679 (**China**) **2 aa** to AB113660 (**Vietnam**) MF039879 (**Thailand**) KM607678 (**China**) …

>R_alyu54

TATTACCCCCAGCGCTCGGGACGGGACATTTGCATCTATAAATAGACCTCCCCCCTCTCCACTACAAGATCATCATCGTCGACAGAAATGGCGCGATATGTGGTATGCTGGATGTTCACC
ATCAACAATCCCGCTTCACTACCAGTGATGCGGGATGAGTTCAAATATATGGTATATCAAGTGGAGAGGGGACAGGAGGGTACTCGTCATGTGCAGGGATACGTCGAGATGAAGAGACGA
AGTTCTCTGAAGCAGATGAGAGGCTTCTTCCCTGGCGCACACCTTGAGAAACGAAAGGGAAGCCAAGAAGAAGCACGGGCATACTGTATGAAGGAAGACACAAGAATCGAAGGTCCCTTC
GAGTTTGGTGCCTTTAAATTGTCATGTAATGATAATTTATATGATGTCATACAGGATATGCGTGAAACGCACAAACGGCCTTTGGAGTATTTATATGAGTGTCCAAATACCTTCGATAGA
AGTAAGGATACATTATACAGAGTTCAAGCAGAGTTGAATAAAACGAAGGCGATGAATAGCTGGAAAACATCCTTCAGTTCTTGGACATCGGAAGTTGAAAATATTATGGCGGAGCCATGT
CATCGGAGGATAATTTGGGTCTATGGCCCAAATGGAGGAGAAGGAAAGACAACTTATGCAAAATATTTAATGAAGACGAAGAATGCGTTTTATTCTCCAGGAGGAAAATCATTGGATATA
TGTAGATTGTATAATTACGAGGAAATAGTTATATTTGATATTCCCAGATGCAAAGAGGAATATTTAAACTATGGTTTATTAGAAGAATTTAAAAATGGAATTATTCAAAGCGGGAAATAT
GAACCCGTTTTGAAAATTGTAGAATATGTGGAAGTCATTGTAATGGCTAACTTCCTTCCGAAGGAAGGAATCTTTTCAGAAGATCGAATAAAGCTAGTTGCTTGCTGAACACGCTATGAC
AATCGTACGCTATGACAAAAGGGGAAAAGCAAAGATTCGGGGGTTGATTGTGCTATCCTAACGATTAAGGGCCGCAGGCCCGTCAAGATGGACGGCTCGATCAGATGTCCCGAGTTAGTG
CGCCACGTAAGCGCTGGGGCTTAT

DNA-S
4-7 SNPs to KM607535 KM607534 KM607536 KM607533 (**China**). **14-19 SNPs** to AB113662 AF148945 AB113661 (**Viet Nam**). **20-32 SNPs** to MF039874 KY427064 MF039880 (**Thailand**)

S protein (170 aa): 0 aa to AY337715 AY494786 HQ378191 (**China**) FJ787433 FJ787434 (**Philippines**) **1 aa** to several isolates

>S_alyu54
TATTACCCCCAGCGCTCGGGACGGGACATGGGCTAATGGATCATGGAATAAGGGCCCATCGGGCCCGTTAAGATGGGTTTTGGGCTTATGGGCTTTATCCAGAAGACCAAAAACAGGCGG
GAACCGTCCCAATTTCAAATTTAGATAGCTTGCCCCGCAAGCAAACTAAAAGCCTATATATACCAGTGTAGACATATTGTTCAGATAACAAATGGCGAGGTTTCCGAAGAAATCGATCAA
GAAGCGGCGGGTTGGGCGACGGAAGTATGGAAGCAAGGCGGCAACCAGTCACGACTACTCGTCGTTAGGTTCAATATTGGTTCCTGAAAATACCGTCAAGGTATTTAGGATTGAGCCTAC
TGATAAAACATTACCCAGATATTTTATCTGGAAAATGTTTATGCTTTTGGTGTGCAAGGTGAAGCCCGGAAGAATACTTCACTGGGCTATGATTAAGAGTTCATGGGAAATCAACCAGCC
GACTACATGTCTGGAAGCACCAGGTTTATTTATAAAACCTGAACATAGCCATCTGGTGAAACTGGTATGCAGTGGGGAACTTGAAGCCGGAGTCGCAACAGGGACATCAGATGTTGAATG
TCTTCTAAGGAAGACAACCGTGTTGAGGAAGAATGTAACAGAGGTGGATTACTTGTATTTGGCATTTTATTGTAGTGCTGGAGTTAGTATTAACTACCAGAACAGAATTACATATCATGT
ATGATATGTTTATGTAAACATAAACCTTTGTATGGAATAATGACCAAATAACATACAACACACTATGAAATACAACACGCTATGACAAACAGGGAAAAATGAAGAATCGGGGGTTGATTG
GTCTATCGTAACGCTTAAGGGCCGCAGGCCCGTTGAAATGATTCTTTATAAAACAAATATACATGATACGGATAGTTGAATATATAAACAACGATGTATAAATACAACAGAATGTTGTAT
ActaattaaaatAATGAGAAGAAAAGAATATTTGTGAAGGATAAGCATCAGAACCACCACTTTAGTGGTGGGTCAGATGTCCCGAGTTAGTGCGCCACGTAAGCGCTGGGGCTTAT

DNA-U3
42/42/44/49 SNPs to MF039875 MF03988 KY427065 MF039869 (**Thailand**)

U3 protein (39 aa): 0 aa to KY427065 (**Thailand)**

>U3_alyu54_v1
TATTACCCCCAGCCCCCAGGACGGGACATGGGCTTTTTAAATGGGCCGAGAGAGTTTGAACAGTTCAGTATCTTCGTTATTGGGCCATCTGGCCCAATAATTAAGAGAACGTGTTCAAAA
TCTGGGTTTGACCGAAGGTCAAGGTAGACGGTCAACAATATTCTGGCTTGCGGAGCAAGCAACAAGAATTAAATATTTAATTCGTATGACACGTGGACGGACCGAAATACTCCTGCATCT
CTATAAATAGCCTAAATCTGGCATGGATAATTGCTCTCGCTCTTCTGTCAAAGGTTTTGTGTTGAGGCGGAAGATCGCCATCGGCGATCATCGGACGAAAAGCTGCAAGAGAGACGGAGA
ACCATGCTGCGAAGCGTATAACGGGTATTTATAGACTTCTAGCGCAGCTAGAAGTATTCTTGTACTTGATATTGTATTTTGTAAATTACGAAGAAATTCGTACATTGATAATAATAAAAC
ATCTGGGATTGTTAATTTTTACATTAACCAGTATTAATAATGTACAATAACAAATATTGTATGAGGAACGAATACAAGAATATTAAAATATGAGGAGCGTAGCGTGATAAACAGGTGTTT
AAGGTATAATTAAATTAATTATGCAATGTCATGATAATACGGTATAAGTTGAAGTATGAGGTGAAAGAGGAGATATTAGAATATTAAAAACCCAATTATATTATTTATAATACATACAAC
ACGCTATGACAAACGGGGAAAAAAGAAAAATCGGGGGTTGATTGGTCTATCCTAACGATTAAGGGCCGCAGGCCCGTCAAGATGGATGAACGGTTAGATTTGATTGCTTAGCCACGAAGA
AAGGAATCGTTTTGGGACCACAGACAAGACAGCTGTCACTACTTTTAAAAAATAATATAATAACCAAATGACGATAGTACCCCTCCCAAAGATGACGTATAGGGGTGTCCCGAGGTAATT
TAACATAGCTCTGACAAGAGATAGTGGACGTTGGATGCCACGATCGGACGATCGTGGTTTGACGAACCAATGATGACGTAGGGGCTGGGGCTTAT

T/G (13643/8939)

>U3_alyu54_v1
TATTACCCCCAGCCCCCAGGACGGGACATGGGCTTTTTAAATGGGCCGAGAGAGTTTGAACAGTTCAGTATCTTCGTTATTGGGCCATCTGGCCCAATAATTAAGAGAACGTGTTCAAAA
TCTGGGTTTGACCGAAGGTCAAGGTAGACGGTCAACAATATTCTGGCTTGCGGAGCAAGCAACAAGAATTAAATATTTAATTCGTATGACACGTGGACGGACCGAAATACTCCTGCATCT
CTATAAATAGCCTAAATCTGGCATGGATAATTGCTCTCGCTCTTCTGTCAAAGGTTgTGTGTTGAGGCGGAAGATCGCCATCGGCGATCATCGGACGAAAAGCTGCAAGAGAGACGGAGA
ACCATGCTGCGAAGCGTATAACGGGTATTTATAGACTTCTAGCGCAGCTAGAAGTATTCTTGTACTTGATATTGTATTTTGTAAATTACGAAGAAATTCGTACATTGATAATAATAAAAC
ATCTGGGATTGTTAATTTTTACATTAACCAGTATTAATAATGTACAATAACAAATATTGTATGAGGAACGAATACAAGAATATTAAAATATGAGGAGCGTAGCGTGATAAACAGGTGTTT
AAGGTATAATTAAATTAATTATGCAATGTCATGATAATACGGTATAAGTTGAAGTATGAGGTGAAAGAGGAGATATTAGAATATTAAAAACCCAATTATATTATTTATAATACATACAAC
ACGCTATGACAAACGGGGAAAAAAGAAAAATCGGGGGTTGATTGGTCTATCCTAACGATTAAGGGCCGCAGGCCCGTCAAGATGGATGAACGGTTAGATTTGATTGCTTAGCCACGAAGA
AAGGAATCGTTTTGGGACCACAGACAAGACAGCTGTCACTACTTTTAAAAAATAATATAATAACCAAATGACGATAGTACCCCTCCCAAAGATGACGTATAGGGGTGTCCCGAGGTAATT
TAACATAGCTCTGACAAGAGATAGTGGACGTTGGATGCCACGATCGGACGATCGTGGTTTGACGAACCAATGATGACGTAGGGGCTGGGGCTTAT

ALYU-55 - Musa AAA Cavendish - China

Virome
1. **BBTV** six components
2. **BBTA6** alyu55
3. **BBTA2** alyu55
4. **BBTA3** alyu55
5. **BBTA5-U3** chimera (2 or more variants)
6. **Badnavirus BSVNV** partial coverage of BSVNV_alyu33 (confirmed by PCR with YN-specific primers)

Two major variants (v1 > v2) of DNA-N, BBTA2 and BBTA3, while only minor variants of DNA-C, DNA-M, DNA-U2 and BBTA5

BBTA6
Best matches to
MF510475 MF510474 low coverage, short stretches of identity *Faba bean necrotic yellows virus associated alphasatellite 2*

Rep protein best matches to RI50297 100% coverage 61% identities 77% positives Sophora yellow stunt alphasatellite 3; P0CK61 100% coverage 60% identities 77% positives Faba bean necrotic yellows C9 alphasatellite; ATU31571 100% coverage 60% identities 77% positives Faba bean necrotic yellows virus associated alphasatellite 2

>BBTA6_alyu55
TATTACCCGACTTTGGCGCAGGGCGCACTATAAAAGATGTCTGTGAGAAATTGGGTCTTCACACGCAATTTCGAAGGCGAAAGACCAGAGCTCCTGTTCGGCGGCGAAACTCAATACGCC
GCTTGGCAACATGAAAGAAAGAATCACGACCACCTACAGGGCGTGATTCAGTTGAAGAAGAAGACCCGCCTGAACGCAGTGAAGAGCATAATCGGTGGAAATCCACATGTCGAACCCATG
CGAGCGTCTTTCGACGATGCCCTAGCATACGTCATGAAAGACGAAACCCGAATCGATGGTCCATGGGAATTCGGTATTCGAATCCGTAAGGGTTCGAATAAACGAAAGCTGTTAGAAATA
CTGGAGGATTCGGACAACGAAGTCCTTCAACCCCAAAAATACAGACGAGCCATGGCCAAACAGGCCATGAGTGAGTCTAAGAAGAAAGCTGCAGAAGAAGGATTTAGTTATGAACTGCGA
GAGTGGCAAGTGCAATTGACGAAGTTGTTGGAAGAAGAACCCGATAACAGAACAATAATTTGGGTATATGGACCAAAGGGTGGAGAAGGTAAAACAGAGTTCGCTAAACATTTAGGGTTA
AAGGATGGTTGGACATATTTGCCCGGTGGTGAACTCAAAGATATGATGTACCTGTTATCAAAGGACATGAAAAATAATGTTGTAATTGATTTCCCCCGTTGTACCAAGGAATTTATTTCA
TATAAATTCCTTGAGATGGTTAAGAATAGAACTATATATAGTTATAAGTATGAACCAGTTGGTTCAATCGTATGTAATAAGGTTCATGTAGTGGTCTTCAGTAATGAGGAGCCAGATTAT
GTAAAATTATCATTGGATCGAATAAAGTTGATTTCTTGCTGAACACGCTATGAAATACAACACGCTATGACAAAAGGGGAAAAAATGAAAAATCGGGGGTTGATTGGTCTATCCTACCGA
CTAAGGGCCGCAGGCCCGTCAAGATGGATGAACGGTTAGATTTGATTGCTTCGCCACGAAGCAAACAAAAAAATGCGTGGTGGAGAATTCGCCACGTCATTGGTTACTGTTATCCTTGGCG
CACTATATATAGGTGCGCCAAAGTCGGCCATAG

BBTA2
best match to EU430730 98% coverage 97% identity; MG545616 99% coverage 97% identity; NC_038953 98% coverage, 95% identity

Rep protein best matches to AZL93963 100% coverage 99% identities (1 aa) 100% positives; ACB86656 100% coverage 99% identities (2 aa) 99% positives; YP_009508281 100% coverage 94% identities (11 gaps in middle) 94% positives Banana bunchy top alphasatellite 2

>BBTA2_alyu55_v1
TATTACCCACCTCCGCGCACCACCTCCGCGCACCTATAAAATGTCTGCCTCTCGCTGGACATTTACGCTTCATTATTCCGACGCAACGGAGCGAGGCAAATTCCTCGCGACTTTGAAGGA
GGAAGATGTGCACTACGCCGTCGTCGGCGACGAAACTGCTCCGAATACTGGTCGAAAACATCTTCAAGGATATCTTTCCTTGAAGAAACGTTTTCGTATTAGCGGCATAAAGAAGAAATA
TTCGTCGAGAGCGCATTGGGAGAAAGCTCGAGGATCAGATTACGACAACAAGGCGTACTGTTCAAAAGAAGCCCTAATTCTTGAATTAGGGGTTCCaTGCCAAACAGGTTCGAATAAGCG
TAAATTAGCAGATATGGTTACAAGATCGCCGGAACGcATGAAAATTGAACAGCCAGAGATATTTCACCGATACGAATCGGTGAAGAAGATGAAAGAATTCAAAGAAAGGTATGTCTATCC
TATCCTCGATAGGCCATGGCAGGTACAATTAACGGAGTTAATTGAAGCAGAACCGGATGATCGAACGATCATCTGGGTATTTGGACCAAAAGGGAATGAAGGCAAATCAACGTACGCGAA
GTCATTAATCCAAAAGGATTGGTTCTACACCAGGGGAGGAAAGAAGGAGAACATACTGTTCGCCTACGTAGATGAAGGTTCGACAAAAAACGTTGTATTTGATCTTCCGCGTACAGTACA
AGAATTTATTAATTATGATGTTATCGAAGCACTGAAGGATAGAGTAATCGAGAGTACAAAATACAAGCCTGTGAAGTATTTAGAATTGAATACTGTACATGTAATAGTTATGGCTAATTT
TCTTCCTGATATGTGTAAAATATCTGAAGATCGAATAAAAATAGTTGCTTGCTGAACACGCTATGAAATACAACACGCTATGACAAACGGGGAAAAATGAAAAATCGGGGGTTGATTGGG
CTATCCTAACGATTAAGGGCCGCAGGCCCGTCAAGATGGATCCAATAACCCGTTAAGAAGGTAAACGGGTCTAAAACGATTCCtTCGCCCGCAAGCAACACCTTTAACCTCTGCGCACCT
ATATATAGCGGAGGTGGGCTAG

C/T (6278/1543) T/A (14489/22452) A/C (17821/27199) TT/AC A/T A/G T/A G/T C/T C/A 2 substitutions (similar aa) in Rep

>BBTA2_alyu55_v2
TATTACCCACCTCCGCGCACtACCTCCGCGCACCTATAAAATGTCTGCCTCTCGCTGGACATTTACGCTTCATTATTCCGACGCAACGGAGCGAGGCAAATTCCTCGCGACTTTGAAGGA
GGAAGATGTGCACTACGCCGTCGTCGGCGACGAAACTGCTCCGAATACTGGTCGAAAACATCTTCAAGGATATCTTTCCTTGAAGAAACGTTTTCGTATTAGCGGCATAAAGAAGAAATA
TTCGTCGAGAGCGCATTGGGAGAAAGCTCGAGGATCAGATTACGACAACAAGGCGTACTGTTCAAAAGAAGCCCTAATTCTTGAATTAGGGGTTCCtTGCCAAACAGGTTCGAATAAGCG
TAAATTAGCAGATATGGTTACAAGATCGCCGGAACGaATGAAAATTGAACAGCCAGAGATATTTCACCGATACGAATCGGTGAAGAAGATGAAAGAATTCAAAGAAAGGTATGTCTATCC
TATCCTCGATAGGCCATGGCAGGTACAATTAACGGAGTTAATTGAAGCAGAACCGGATGATCGAACGATCATCTGGGTATacGGACCAAAAGGGAATGAAGGCAAATCAACGTACGCGAA
GTCATTAATCCAAAAGGATTGGTTCTACACCAGGGGAGGAAAGAAGGAGAACATACTGTTCGCCTACGTAGATGAAGGTTCGACAAAAAACGTTGTATTTGATCTTCCGCGTACAGTACA
AGAATTTATTAATTATGATGTTATCGAAGCACTGAAGGATAGAGTAATCGAGAGTACAAAATACAAGCCTGTGAAGTATTTAGAATTGAATtCTGTACATGTAATAGTTATGGCTAATTT
TCTTCCTGATATGTGTAAAATATCTGAAGATCGAATAAAAATAGTTGCTTGCTGAACACGCTATGAAATACAACACGCTATGACAAACGGGGAAAAATGAAgAATCGGGGGTTGATTGGG
CTATCCTAACGATTAAGGGCCGCAGGCCCGTCAAGATGGATCCAATAACCCGaTAAGAAGtTAAACGGGTCTAAAACGATTtCtTCGCCCGCAAGCAACACCTTTAACCTCTGCGCACCT
ATATATAGCGGAGGTGGGaTAG

BBTA3
best match to GU074391 ca 100% coverage 97% identity; NC_038955 ca 100% coverage 97% identity; GU074392 ca 100% coverage, 97% identity

Rep protein best matches to: YP_009508283 100% coverage 99% identities (1 aa) 100% positives Banana bunchy top alphasatellite 3; ADV18492 100% coverage 98% identities, 99% positive; ACJ36781 100% coverage 89% identities 95% positives

>BBTA3_alyu55
TATTACCCACTTCCTCGCCCTACTTCCTCGCCCTGACGTCATAAGATGTCATCATTCAAATGGTGTTTCACTCTGAATTATTCCTCCGCAGCCGAGAGAGAAGACTTTCTCTCGCGTCTG
AAGGAGGATGATGTTTATTACGCGGTCGTCGGCGACGAAGTCGCTCCGAGCTCCGGCCAGAAACACCTTCAGGGATATCTATCCCTGAAGAAATCGATGAAGCTAGGCGGACTGAAGAAG
AGGTACTCTTCGAAGGCTCACTGGGAGAAGGCGAGAGGAACAGACAAAGAGAATTCGAAGTATTGCTCCAAGGAAACCCTAATTCTCGAAATAGGGTTTCCTGCTACTCAGGGTTCTAAT
AAACGAAAACTATCAGAGATGGTATCCAGATCACCGGAACGCATGAGAATCGAACAGCCGGAGATATATAACAGATATCTATCTGTGAGTAAGCTAAAAAAATTCAAGGAgGAATTTGTT
CATCCTTGCCTCGAAAGACCATGGCAAATTCAATTGACGGAGGCAATTGCCGAGGAACCCGATGATCGCAGTATCATCTGGGTTTACGGTCCGAATGGCAATGAGGGAAAGTCAACATAT
GCGAAGTCGCTGATTAAGAAGGATTGGTTCTACACCAGAGGTGGGAAGAAGGAGAATATCTTATTCTCCTATGTGGACGAAGGATCGACGAAACATATAGTATTTGATATCCCTCGATGT
AATCAGGATTATCTAAATTATGATGTAATAGAGGCTTTAAAGGATAGAGTAATAGAGAGTACGAAATATAAACCCATAAAGATAGTTGAATTAGATAGGATTCATGTAATCGTGATGGCT
AATTTCATGCCAGATTTCTGTAAAATCTCTGAGGATCGAATAAATATTATATATTGTTGAACACGCTATGAAATACAACACACTATGAAATACAACACAGGTAAGATTGTGTATCCTAAC
GAATATGGGCCGCAGGCCCAACACTATTCTGGGCCGACGGCCCAGAGCGAAGCGCTTGCGCTGAAGCTTCTCGAAGAAGGAACCACTTTAAGTCCCTCGCCCTATAAATAGTTTTCGCCC
GAGGAAGTGGCCTAG

A/G (26975/76754) No change in Rep aa

>Babu3chn_alyu55_uncorrected
TATTACCCACTTCCTCGCCCTACTTCCTCGCCCTGACGTCATAAGATGTCATCATTCAAATGGTGTTTCACTCTGAATTATTCCTCCGCAGCCGAGAGAGAAGACTTTCTCTCGCGTCTG
AAGGAGGATGATGTTTATTACGCGGTCGTCGGCGACGAAGTCGCTCCGAGCTCCGGCCAGAAACACCTTCAGGGATATCTATCCCTGAAGAAATCGATGAAGCTAGGCGGACTGAAGAAG
AGGTACTCTTCGAAGGCTCACTGGGAGAAGGCGAGAGGAACAGACAAAGAGAATTCGAAGTATTGCTCCAAGGAAACCCTAATTCTCGAAATAGGGTTTCCTGCTACTCAGGGTTCTAAT
AAACGAAAACTATCAGAGATGGTATCCAGATCACCGGAACGCATGAGAATCGAACAGCCGGAGATATATAACAGATATCTATCTGTGAGTAAGCTAAAAAAATTCAAGGAaGAATTTGTT
CATCCTTGCCTCGAAAGACCATGGCAAATTCAATTGACGGAGGCAATTGCCGAGGAACCCGATGATCGCAGTATCATCTGGGTTTACGGTCCGAATGGCAATGAGGGAAAGTCAACATAT
GCGAAGTCGCTGATTAAGAAGGATTGGTTCTACACCAGAGGTGGGAAGAAGGAGAATATCTTATTCTCCTATGTGGACGAAGGATCGACGAAACATATAGTATTTGATATCCCTCGATGT
AATCAGGATTATCTAAATTATGATGTAATAGAGGCTTTAAAGGATAGAGTAATAGAGAGTACGAAATATAAACCCATAAAGATAGTTGAATTAGATAGGATTCATGTAATCGTGATGGCT
AATTTCATGCCAGATTTCTGTAAAATCTCTGAGGATCGAATAAATATTATATATTGTTGAACACGCTATGAAATACAACACACTATGAAATACAACACAGGTAAGATTGTGTATCCTAAC
GAATATGGGCCGCAGGCCCAACACTATTCTGGGCCGACGGCCCAGAGCGAAGCGCTTGCGCTGAAGCTTCTCGAAGAAGGAACCACTTTAAGTCCCTCGCCCTATAAATAGTTTTCGCCC
GAGGAAGTGGCCTAG

BBTA5-U3 chimera
830-1056/1-260 – BBTV5 (with CR-M and CR-SL)
260-914 – DNA-U3 (share CR-M)

>BBTA5_U3_alyu55_uncorrected contig
TATTACCCGACTTTGGCGCAGGGCGCACTATAAAAG**ATG**TCGCAGCAGCGAAATTGGGTCTTCACACGCAATTTCGAAGGCGAAAGACCAGAGCTCCTGTTCGGCGGCGAAACTCAATAC
GCGTGTTGGCAACATGAGAAGAAGAATCACGACCATCTTCAGGGAGTGATTCAATTGAAGAAGAAGCTCCGGATGAACGCAGTGAAGACGTTAATTGGGGAAATCCACACCTCGAAGCC
ATGAGAGGAACAATCGATGAACAATATTCTGGCTTGCGAAGCGAGCAACAAGAATTAAATAAT**TAA**TTCGTAGGACACATGGACGGACCGAAATACTCTTGTATCTATATAAATACCCTA
AACTTGGC**ATG**GATAATTGCTCTCGGTGTTCTAGCAAAGCTCTGTTCTTGAGGCGGAAGATCGCCGTCGGCGATCATCAGACGAAATGCTGCAAGAGAGACGGAGAATCATGTTGCGAAG
CGTATATTTGGTATTTATATACTTATAGCGAAGCTAGAAGTAACCATGTACATGATACTGTATTTATG**TAA**AATACGAAGAAATACGTATATTGATAATAATAAAACAACTGGGTTTGTT
AATGTTTACATTAACTAGTATTAATAATGTACAATAACAAATATTGTATGCGGAACGAATACAAGAATATAAAAAATATGAGGTGCGAAGCGTGATAAACAGAtGTTTAAGGTATAATTA
AAATAATTATGAAATGTTAATATAATACGGTATAAGTTGAAGTATGAGGTGAAAGAGGAGATATTAGAATATTAAAAACCCAATTATATTATTTATAATACATACAACACGCTATGACA
AAAGGGGAAAAATGAAGAATCGGGGGTTGATTGGGCTATCCTAACGATTAAGGGCCGCAGGCCCGTACAAAATGAGCGGGAAATTCAAAATCGGACGCCAAATATTATTAAATACAATAC
ACCGCCTTTTAGAAATCCGTGGACGAGAAGATGCCACGTAGCAAAACACTGTACTCCTTGGCGCACTATATATAGGTGCGCCAAAGTCGGCCATAG

>BBTA5_U3_alyu55_correction1
TATTACCCGACTTTGGCGCAGGGCGCACTATAAAAG**ATG**TCGCAGCAGCGAAATTGGGTCTTCACACGCAATTTCGAAGGCGAAAGACCAGAGCTCCTGTTCGGCGGCGAAACTCAATAC
GCGTGTTGGCAACATGAGAAGAAGAATCACGACCATCTTCAGGGAGTGATTCAATTGAAGAAGAAGCTCCGGATGAACGCAGTGAAGACGTTAATTGGGGAAATCCACACCTCGAAGCC
ATGAGAGGAACAATCGATGAACAATATTCTGGCTTGCGAAGCGAGCAACAAGAATTAAATAAT**TAA**TTCGTAGGACACgTGGACGGtCCGAAATACTCcTGcATCTcTATAAATACCtaA
AACcTGGC**ATG**GATAATTGCTCTCGGTcTTCTAGCAAAGCTCTGgTgTTGAGGCGGAAGATCGCCGTCGGCGATCATCAGACGAAATGCTGCAAGAGAGACGGAGAATCATGcTGCGAAG
CGTATATcgGGTATTTATATACgTATAGCGAAGCTAGAAGTAACCtTGTACATGATACTGTATTTtTG**TAA**AATAtGAAGAAATtCGaATATTGAcAATAATAAAACAtCTGGGTTTGTT
AATGTTTACATTAACcAGTATTAATAATGTACAATAACAAATATTGTATGCGGAACGAATACAAGAATATAAAAAATATGAGGTGCGAAGCGTGATAAACAGATGTTTAAGGTATAATTA
AAATAATTATGcAATGTaAtTATAATACGGTATAAGTTGAAGTATGAGGaGAAAGAGGAGATATTAGAATATTAAAAAgCCAATTATATTATTTTATAgaACATACAACACGCTATGACA
AAAGGGGAAAAATGAAGAATCGGGGGTTGATTGGGCTATCCTAACGATTAAGGGCCGCAGGCCCGTACAAAATGAGCGGGAAATTCAAAATCGGACGCCAAATATTATTAAATACAATAC
ACCGCCTTTTAGAAATCCGTGGACGAGAAGATGCCACGTAGCAAAACACTGTACTCCTTGGCGCACTATATATAGGTGCGCCAAAGTCGGCCATAG

>BBTA5_U3_alyu55_correction2
TATTACCCGACTTTGGCGCAGGGCGCACTATAAAAG**ATG**TCGCAGCAGCGAAATTGGGTCTTCACACGCAATTTCGAAGGCGAAAGACCAGAGCTCCTGTTCGGCGGCGAAACTCAATAC
GCGTGTTGGCAACATGAGAAGAAGAATCACGACCATCTTCAGGGAGTGATTCAATTGAAGAAGAAGCTCCGGATGAACGCAGTGAAGACGTTAATTGGGGAAATCCACACCTCGAAGCC
ATGAGAGGAACAATCGATGAACAATATTCTGGCTTGCGAAGCGAGCAACAAGAATTAAATAAT**TAA**TTCGTAGGACACgTGGACGGtCCGAAATACTCcTGcATCTcTATAAATACCtaA

AACcTGGC**ATG**GATAATTGCTCTCGGTcTTCTAGCAAAGCTCTGgTgTTGAGGCGGAAGATCGCCGTCGGCGATCATCAGACGAAATGCTGCAAGAGAGACGGAGAATCATGcTGCGAAG
CGTATATcgGGTATTTATATACgTATAGCGAAGCTAGAAGTAACCtTGTACATGATACTGTATTTtTG**TAA**AATAtGAAGAAATtCGaATATTGAcAATAATAAAACAtCTGGGTTTGTT
AATGTTTACATTAACcAGTATTAATAATGTACAATAACAAATATTGTATGCGGAACGAATACAAGAATATAAAAAATATGAGGTGCGAAGCGTGATAAACAGATGTTTAAGGTATAATTA
AAATAATTATGcAATGTaAtTATAATACGGTATAAGTTGAAGTATGAGGaGAAAGAGGAGATATTAGAATATTAAAAAgCCAATTATATTATTTTATAgaACATACAACACGCTATGACA
AAAaGGGAAAAATGAAaAATCaGGGGTTGATTGGGCTATCCTAACGATTAAGGGCCGCAGGCCCGTACAAAATGAGCGGGAAATTCAAAATCGGACGCCAAATATTATTAAATACAATAC
ACCGCCTTTTAGAAATCCGTGGACGAGAAGATGCCACGTAGCAAAACACTGTACTCCTTGGCGCACTATATATAGGTGCGCCAAAGTCGGCCATAG

>BBTA5_U3_alyu55_correction3
TATTACCCGACTTTGGCGCAGGGCGCACTATAAAAGATGTCGCAGCAGCGAAATTGGGTCTTCACACGCAATTTCGAAGGCGAAAGACCAGAGCTCCTGTTCGGCGGCGAAACTCAATAC
GCGTGTTGGCAACATGAGAAGAAGAATCACGACCATCTTCAGGGAGTGATTCAATTGAAGAAGAAGCTCCGGATGAACGCAGTGAAGACGTTAATTGGGGGAAATCCACACCTCGAAGCC
ATGAGAGGAACAATCGATGAACAATATTCTGGCTTGCGAAGCGAGCAACAAGAATTAAATAATTAATTCGTAGGACACGTGGACGGTCCGAAATACTCCTGCATCTCTATAAATACCTAA
AACCTGGCATGGATAATTGCTCTCGGTCTTCTAGCAAAGCTCTGGTGTTGAGGCGGAAGATCGCCGTCGGCGATCATCAGACGAAATGCTGCAAGAGAGACGGAGAATCATGCTGCGAAG
CGTATATCGGGTATTTATATACGTATAGCGAAGCTAGAAGTAACCTTGTACATGATACTGTATTTTTGTAAAATATGAAGAAATTCGAATATTGACAATAATAAAACATCTGGGTTTGTT
AATGTTTACATTAACCAGTATTAATAATGTACAATAACAAATgTTGTATGaGGAACGAATACAAcAATATAtAAAATATGAGGaGCGtAGCGTGATtAACAGATGTTTAAGGTATAATTA
AAATAATTATGCAATGTAATTATAATACGGTATAAGTTGAAGTATGAGGAGAAAGAGGAGATATTAGAATATTAAAAAGCCAATTATATTATTTTATAGAACATACAACACGCTATGACA
AAAaGGGAAAAATGAAaAATCaGGGGTTGATTGGGCTATCCTAACGATTAAGGGCCGCAGGCCCGTACAAAATGAGCGGGAAATTCAAAATCGGACGCCAAATATTATTAAATACAATAC
ACCGCCTTTTAGAAATCCGTGGACGAGAAGATGCCACGTAGCAAAACACTGTACTCCTTGGCGCACTATATATAGGTGCGCCAAAGTCGGCCATAG

DNA-C
12 SNPs to KM607099 KM607098 (**China**). **20-24 SNPs** to KY427060 MF039864 MF039876 MF039870 (**Thailand**)

C protein (161 aa): 1 aa to KM607098 KM607099 (**China**) **2 aa** to KY427060 (**Thailand**) **3 aa** to MF039864 (**Thailand**)

>C_alyu55
TATTACCCCCCCTGCTCGGGACGGGACATCACGTGCTTCTAACAAATGCACGTGACAATGCAGTAGCTTGTAGCGAAAGATAACCATCACTTTCACAAAAGTGAAAGGAATATTCGTTGC
TTAGGGACTAAGCAACGAGGACAGATATTTGTTCGAGAAGCGACAATGGAGGCTATTTAAACCTGATGGTTTTGTGATTTCCGAAATCACTCATCGGAGCAGAAATGGAGTTCTGGGAAT
CGTCTGCTATGCCAGACGATGTCAAGAGAGTGATCAAGGAAATATATTGGGAGCATCGAAATAAACTTCTGTTCTGTCAGAAGTTGAAAGGCTGTGTAAGAAGGATTCTGGAGCATGGAA
ATCAAGATGATGCTCTAGACGCAGTGAAGGAGATGAAGACTTCTATTATTCGCTATAGCGAATATTTGAAGAAACCTTGTGTGGTATTGTGTTTTAGTACTAATAAATCTATTGCATATA
GGTTAAACACAATGGTGTTCTTTTATCATGAATATATTGAAGACCTAGGTGGTGACTACTCGGTATATCAAGATCTGTTTTGTGATGAGGAACTTCCTTCGTCTTCGACAGAAGAAGAAG
ATGAAGAAGTAATATACAGGAATGTTATCATGGCATCGACAGAAGAGAAGATCTCTTGGAGTGAATGTCAAAAGATAGTTATATCAGATTATGATGTAACATTACTGTAATGAATATCCA
TTATCATAAATAAAATAATGGAATGATGATTATGTATTCTAGTATAAATACATAATGGTATACGTATAGCATAAAATACATAAACCTACATACAACACACTATGACAAACAGGGAAAAAT
GAAGAATCGGGGGTTGATTGGGCTATCTTAACGATTAAGGGCCGAAGGCCCGTTTAAATATGTGGTGGACGAAGTCCTCAACACAAAAAAGTGATCAGAACAATGGAATATAATGAGCTG
GCAACGTAGGGACCATGTCCCGAGTTAGTGCGCCACGTGAGCAGGGGGGCTTAT

DNA-M
18-23 SNPs to KM607239 KM607240 (**China**). **51-52 SNPs** to KY427061 MF039871 MF039865 MF039876 (**Thailand**)

M protein (117 aa): 3 aa to KM607239 (**China**) KC581796 MF039871 KY427061 MF039865 MF039877 (**Thailand**)

>M_alyu55
TATTACCCCCAGCGCTCAGGACGGGACATCACGTGCAACTAACAAATGCACGTGACTGATATATACTACATAACGGTTCAACGAACCGTTATATAATGTTATAACGACAAGTCACGTGTG
ATAGAGACATGCACGTGACATAGTCAAATGTATTGAATAAACATTTGACGTCCGGATGCTTCCGACGGAAGCTTAGGTTACTTCGTGGCGAAGCAAAACATTTATATATTGGCCTGGATT
GCTGCCTATAAATAGGCAAGCAAGGAAATGGCATTAACAACAGAGCGGGTGAAACTATTCTTCGAATGGTTTCTGTTCATCGGTGCAATATTTATTGCGATAACAATATTATATATATTG
TTGGCGTTGCTCTTTGAGGTCCCCAAGTATATTAAGGATGTTGTGAGGTATCTCGTAGAATACCTGACCAGAAGACGTGTATGGATGCAGAGAACGCAGTTGACGGAGGCAACAGGAGAT
GTAGAGCTCGGCAGAGGTAGTGTGGAAGACAGACGGGATCTACAACCGGCTGTCATACCACATGCAACACAGGTTATCCCTTCGCATCAACCTAGAAGGGATGATCAAGGAAGACGAGGA
AACGCAGGACCAATGTTTTAATACACGGTATTGTAATATACGAAATATAAATGGATAATGATATGTATTGTGAAACATAATATACGTGAAACATAATATATGTTTGTTAATTTACATATT
ATAATATGTAAATTGTATACGAGTGTTGTATTTATAGAACATACAACACGCTATGACAAACAGGGAAAAATGAAGAATCGGGGGTTGATTGGTCTATCGTATCGCTTAAGGGCCGCAGGC
CCGTTGAAATGATTCTTAATAAAACAAATATACATGATACGGATAGTTGAATATATAAACAACGAAGTATAAATACAACAGAATGTTGTATACTAATTAAAATAATGAGAAGATAAGTAT
ATTTGTGAGGGATGATGATCACAACCACCACTTTAGTGGTGGGTCATATGTCCCGAGTTAGTGCGCCACGTAAGCGCTGGGGCTTAT

DNA-N
26 SNPs to KM607387 KM607385 (**China**). **26-29 SNPs** to MF039872 MF039878 MF039866 KY427062 (**Thailand**). **36 SNPs** to KM607386 (**China**)

N protein (154 aa): 0 aa to KM607387 KM607385 (**China**) MF039872 MF039878 KY427062 (**Thailand**) **1 aa** to MF039866 (**Thailand**)

>N_alyu55_v1
TATTACCCCCCGTGCTTGGGACGGGACATGACGTCAGCATAGATTATAATGGGCCAACTAAAGGCCCATTTAATAGAATAGGCGGGCTTTTGACATATTTCAAAGGCCCAGCCTGGAAGT
GGATAATGTCACGTGCCAAATAAGAGGTTGCTTCGCCTCGAAGCAAACCGAATAAATGTTGCGTATTCAATACGCAACTGAAGTCTATTAATATGGATGTCTCTGCCGAATAAATCAGAG
CGTAAGCGAAGCTGAAGCGATGGATTGGGCAGAATCACAATTCAAGACATGTACTCATGGGTGCGATTGGAAGACGATATCATCGGATTCATCCGAGAATCGACAATATGTACCTTGCGT
CGACGCTGGCAGCGGAAGAAAGTCTCCTCGCAAGGTACTGCTAAGATCTATTGAAGCTGTGTTTAATGGAAGCTTCAAAGGAAATAACAGGAATGTTCGTGGGTTTATATACGTATCAAT
ACGAGACGATGATGGAGAAATGCGTCCAGTACTCATAATACCATTCGGAGGATATGGATATCATAATGATTTCTATTATTTCGAAGGGAAGGGGAAAGTTGAATGTGATATATCATCAGA
TTATGTAGCGCCAGGAGTCGATTGGAGCAGAGACATGGAAGTTAGTATTAGTAACAGCAACAACTGTAATGAATTATGTGATCTGAAGTGTTATGTTGTTTGTTCGTTAAGAATAAAGGA
ATAACAGATGTGCTGTAATGATTATTAATAAAACTTATTTTCATGTAATTGATAGTTGTATAAAACATACAACACGCTATGACAAACAGGGAAAAATGAAAAATCAGGGGTTGATTGGTC
TATCGTATCGCTTAAGGGCCGCAGGCCCGTTGAAAAATAATAATCGAATTATAAACGTATGATAATAATCAGAGATAGATGATAAGCATCCAAAAACATAGACGAAGTATATGGCTGTAT
AATATAAAACAAGCATATAAATAAATAAATGTGAACTAATCTCTGATTGGTTCAGAAGTGTACCCCACTAACTTTAAGTTAGTGGAAATGTCCCGATGACGTAAGCACGGGGGACTAT

DNA-R
9 SNPs to AB113660 (**Viet Nam**). **10-13 SNPs** to KM607678 KM607677 KM607676 KM607679 (**China**). **17 SNPs** to MF039879 (**Thailand**)

R protein (286 aa): 0 aa to AB113660 (**Vietnam**) **1 aa** to KM607676 KM607677 KM607679 (**China**)…

>R_alyu55
TATTACCCCCAGCGCTCGGGACGGGACATTTGCATCTATAAATAGACCTCCCCCCTCTCCACTACAAGATCATCATCGTCGACAGAAATGGCGCGATATGTGGTATGCTGGATGTTCACC
ATCAACAATCCCGCTTCACTACCAGTGATGCGGGATGAGTTCAAATATATGGTATATCAAGTGGAGAGGGGACAGGAGGGTACTCGTCATGTGCAGGGATACGTCGAGATGAAGAGACGA
AGTTCTCTGAAGCAGATGAGAGGCTTCTTCCCTGGCGCACACCTTGAGAAACGAAAGGGAAGCCAAGAAGAAGCACGGGCATACTGTATGAAGGAAGACACAAGAATCGAAGGTCCCTTC
GAGTTTGGTGCCTTTAAATTGTCATGTAATGATAATTTATTTGATGTCATACAGGATATGCGTGAAACGCACAAACGGCCTTTGGAGTATTTATATGAGTGTCCAAATACCTTCGATAGA
AGTAAGGATACATTATACAGAGTTCAAGCAGAGTTGAATAAAACGAAGGCGATGAATAGCTGGAAAACATCCTTCAGTaCTTGGACATCGGAAGTTGAAAATATTATGGCGGAGCCATGT
CATCGGAGGATAATTTGGGTCTATGGCCCAAATGGAGGAGAAGGAAAGACAACTTATGCAAAATATTTAATGAAGACGAAGAATGCGTTTTATTCTCCAGGAGGAAAATCATTGGATATA
TGTAGATTGTATAATTACGAGGAAATAGTTATATTTGATATTCCCAGATGCAAAGAGGAATATTTAAACTATGGTtTATTAGAAGAATTTAAAAATGGAATTATTCAAAGCGGGAAATAT
GAACCCGTTTTGAAAATTGTAGAATATGTtGAAGTCATTGTAATGGCTAACTTCCTTCCGAAGGAAGGAATCTTtTCAGAAGATCGAATAAAGCTAGTTGCTTGCTGAACACGCTATGAC
AATCGTACGCTATGACAAAAGGGGAAAAGCAAAGATTCGGGGGTTGATTGTGCTATCCTAACGATTAAGGGCCGCAGGCCCGTCAAGATGGACGGCTCGATCAGATGTCCCGAGTTAGTG
CGCCACGTAAGCGCTGGGGCTTAT

DNA-S
13-15 SNPs to KM607534 KM607535 KM607536 KM607533 (**China**). **14-17 SNPs** to AB113662 KY427064 AB113661 (**Viet Nam**). **20 SNPs** to MF039874 (**Thailand**)

S protein (170 aa): 1 aa to AY337715 AY494786 HQ378191 (**China**) FJ787433 FJ787434 (**Philippines**) **2 aa** to several isolates

>S_alyu55
TATTACCCCCAGCGCTCGGGACGGGACATGGGCTAATGGATCATGGAATAAGGGCCCATCGGGCCCGTTAAGATGGGTTTTGGGCTGATGGGCTTTATCCAGAAGACCAAAAACAGGCGG
GAACCGTCCCAATTTCAAATTTAGATAGCTTGCCCCGCAAGCAAACTAAAAGTCTATATATACCAGTGTAGACATATTGTTCAGATAACAAATGGCGAGGTTTCCGAAGAGATCGATCAA
GAAGAGGCGGGTTGGGCGACGGAAGTATGGAAGCAAGGCGGCAACCAGTCACGACTACTCGTCGTTAGGTTCAATATTGGTTCCTGAAAATACCGTCAAGGTATTTAGGATTGAGCCTAC
TGATAAAACATTACCCAGATATTTTATCTGGAAAATGTTTATGCTTTTGGTGTGCAAGGTGAAGCCCGGAAGAATACTTCACTGGGCTATGATTAAAAGTTCATGGGAAATCAACCAGCC

GACTACATGTCTGGAAGCACCAGGTTTATTTATAAAACCTGAACATAGCCATCTGGTGAAACTGGTATGCAGTGGGGAACTTGAAGCCGGAGTCGCAACAGGGACATCAGATGTTGAATG
TCTTCTAAGGAAGACAACCGTGTTGAGGAAGAATGTAACAGAGGTGGATTACTTGTATTTGGCATTTTATTGTAGTGCTGGAGTTAGTATTAACTACCAGAACAGAATTACATATCATGT
ATGATATGTTTATGTAAACATAAACCTTTGTATGGAATAATGCCCAAATAACATACAACACACTATGAAATACAACACGCTATGACAAACAGGGAAAAATGAAGAATCGGGGGTTGATTG
GTCTATCGTATCGCTTAAGGGCCGCAGGCCCGTTGAAATGATTCTTTATAAAACAAATATACATGATACGGATAGTTGAATATATAAACAACGATGTATAAATACAACAGAATGTTGTAT
ACTAATTAAAAAAATGAGAAGAAAAGGATATTTGTGAAGGATAAGCATCAGAACCACCACTTTAGTGGTGGGTCATATGTCCCGAGTTAGTGCGCCACGTAAGCGCTGGGGCTTAT

DNA-U3
49-55 SNPs to KY427065 MF039869 (**Thailand**)

U3 protein (39 aa): 0 aa to KY427065 (**Thailand)**

>U3_alyu55
TATTACCCCCAGCCCCCAGGACGGGACATGGGCTTTTTAAATGGGCCGAGAGAGTTTGAACAGTTCAGTATCTTCGTTATTGGGCCATCTGGCCCAATAATTAAGAGAACGTGTTCAAAA
TCTGGGTTTGACCGAAGGTCAAGGTAGACGGTCAACAATATTCTGGCTTGCGGAGCAAGCAACAAGAATTAAATATTTAATTCTTAAGACACGTGGACGGACCGAAATACTCCTGTATCT
CTATAAATAGCCTAAATCTGGCCTGGATAATTGCTCTCGCTCTTCTGTCAAAGCTGTTGTGTTGAGGCGGAAGATCGCCACCGGCGATCATCGGACGAAAAGCTGCAAGAGAGACGGAGA
ACCATGCTACGAAGCGTATATCGGGTATTTATAGACTTCTAGCGCAGCTAGAAGTTTCCTTGTACTTGTTATTGTATTTTGTAAAGTACGAAGAAATTCGTATATTGATAATAATAAAAC
ATCTGGGTTTGTTAATGTTTACATTAACCAGTATTAATAATGTACAATAACAAACATTGTATGAGGAACGAATACAAGAATATTAAAAAATGAGGAGCGTAGCGTGATAAACAGGTGTTT
AAGGTATAATTACAATAATTATGCAAAGTCATGATAATACGGTATAAGTTGAAGTATGAGGTGAAAGAGGAGGTATTAGAATATTAAAAACCCAATTATATTATTTATAATACATACAAC
ACGCTATGACAAACGGGGAAAAAAGAAAAATCGGGGGTTGATTGGTCTATCCTAACGATTAAGGGCCGCAGGCCCGTCAAGATGGATGAACGGTTAGATTTGATTGCTTAGCCACGAAGA
AAGGAATCGTTTTGGGACCACAGACAAGACAGCTGTCACTACCTTTAAAAAATAATATAATAACCAATTGACGATAGTACCCCTCCCAAAGATGACGTATAGGGGTGTCCCGAGGTAATT
TAACATAGCTCTGACAAGAGATAGTGGACGTTGGATGCCACGATCGGACGATCGTGGTTTGATGAACTTATGATGACGTAGGGGCTGGGGCTTAT

ALYU-56 - **Musa acuminata Cavendish** - **China**

Virome
1. **BBTV** six components
2. **BBTA2** alyu56

BBTA2
best match to EU430730 100% coverage 98% identity; host: MG545616 100% coverage, 97% identity; NC_038953 98% coverage, 96% identity

Rep protein best matches to
AZL93963 100% coverage 99% identities (1 aa) 100% positive; ACB86656 100% coverage, 99% identities (2 aa) 99% positives; YP_009508281 (100% coverage 94% identities (11 gaps in middle) 94% positives Banana bunchy top alphasatellite 2

>BBTA2_alyu56
TATTACCCACCTCCGCGCACCACCTCCGCGCACCTATAAAATGTCTGCCTCTCGCTGGACATTTACGCTTCATTATTCCGACGCAACGGAGCGAGGCAAATTCCTCGCGACTTTGAAGGA
GGAAGATGTGCACTACGCCGTCGTCGGCGACGAAACTGCTCCGAATACTGGTCGGAAACATCTTCAAGGATATCTTTCCTTGAAGAAACGTTTTCGTATTAGCGGAATAAAGAAGAAATA
TTCGTCGAGAGCGCATTGGGAGAAAGCTCGAGGATCAGATTACGACAACAAGGCGTACTGCTCCAAAGAAGCCCTAATTCTTGAATTAGGGGTTCCTTGCCAAACAGGTTCGAATAAGCG
TAAATTAGCAGATATGGTTACAAGATCTCCAGAACGAATGAAAATTGAACAGCCAGAGATATTTCACCGATACGAATCGGTGAAGAAGATGAAAGAATTCAAAGAAAGGTATGTCTATCC
TATCCTCGATAGGCCATGGCAGGTACAATTAACGGAGTTAATTGAAGCAGAACCTGATGATCGAACGATCATCTGGGTATTCGGACCAAAAGGGAATGAAGGCAAATCAACGTATGCGAA
GTCATTAATCCAAAAGGATTGGTTCTACACAAGGGGCGGAAAGAAGGAGAACATATTGTTCGCCTACGTAGATGAAGGTTCGACCAAAAACGTTGTATTTGATCTTCCGCGTACAGTACA
AGAATTTATTAATTATGATGTTATCGAAGCACTGAAGGATAGAGTAATCGAGAGTACAAAATACAAGCCTGTGAAGTATTTAGAGTTGAATACTGTACATGTAATAGTTATGGCTAATTT
TCTTCCTGATATGTGTAAAATATCTGAAGATCGAATAAAAATAGTTGCTTGCTGAACACGCTATGAAATACAACACGCTATGACAAAAGGGGAAAAAAGAAAAATCGGGGGTTGATTGGG
CTATCCTAACGATTAAGGGCCGCAGGCCCGTCAAGATGGATCCAATAACCCGATAAGAAGGTAAACGGGTCTAAAACGCTTTCTTCGCCCGCAAGCAACACCTTTAACCTCTGCGCACCT
ATATATAGCGGAGGTGGGCTAG

DNA-C
15 SNPs to KM607099 KM607098 (**China**). **23-27 SNPs** to KY427060 MF039870 MF039864 MF039876 (**Thailand**)

C protein (161 aa): 4 aa to KM607098 KM607099 (**China**) **5 aa** to KY427060 (**Thailand**)

>C_alyu56
TATTACCCCCCCTGCTCGGGACGGGACATCACGTGCTTCTAACAAATGCACGTGACAAAGCAGTAGCTTGTAGCGAAAGATAACCATCACTTTCACAAAAGTGAAAGGAATATTCGTTGC
TTAGGGACTAAGCAACGAGGACAGATATTTGTTCGAGAAGCGACAATGGAGGCTATTTAAACCTGATGGTTTTGTGATTTCCGAAATCACTCATCGGAGCAGAAATGGAGTTCTGGGAAT
CGTCTGCTATGCCAGACGATGTCAAGAGAGTGATCAAGGAAATATATTGGGAGCATCGAAATAAACTTCTGTTTTGTCAGAAGTTGAAAGGCTGTGTAAGAAGGATTCTGGAGCATGGAA
ATCAAGATGATGCTCTAGACGCAGTGAAGGAGATGAAGACTTCTATTATTCGCTATAGCGAATATTTGAAGAAACCTTGTGTGGTATTGTGTTTTAGTACTAATAAATCTATTGTATATA
GGTTAAACACAATGGTGTTCTTTTATCATGAATATATTGAAGACCTAGGTGGTGACTACTCGGTATATCAAGATCTGTATTGTGATGAGGAACTTCCTTCGTCTTCGACAGAAGAAGAAG
ATGAAGAAGTAACATACAGGAATGTTATCATGGCATCGACAGAAGAGAAGATCTCTTGGAGTGAATGTCAAAAGATAGTTATATCAGATTATGATGTAACATTACTGTAATGAATATCCA
TTATCATAAATAAAATAATGGAATGATGATTATGTATTCTAGTATAAATACATAATGGTATACGTATAGCATAAGATACATAAACCTACATACAACACACTATGACAAACAGGGAAAAAT
GAAGAATCGGGGGTTGATTGGGCTATCTTAACGATTAAGGGCCGAAGGCCCGTTTAAATATGTGGTGGACGAAGTCCACAACACAAAAAAGTGATCAGAACAATGGAATATAATGAGCTG
GCAACGTAGGGACCATGTCCCGAGTTAGTGCGCCACGTGAGCAGGGGGGCTTAT

DNA-M
19-24 SNPs to KM607239, KM607240 (**China**). **52-53 SNPs** to KY42706 MF039865 MF039871 MF039876 (**Thailand**)

M protein (117 aa): 3 aa to KC581796 MF039871 (**Thailand**) **4 aa** to KM607239 (**China**) KY427061 MF039865 MF039877 (**Thailand**)

>M_alyu56
TATTACCCCCAGCGCTCAGGACGGGACATCACGTGCAACTAACAAATGCACGTGACTGATATATACTACATAACGGTTCAACGAACCGTTATATAATGTTATAACGACAAGTCACGTGTG
ATAGAGACATGCACGTGACATAGTCAAATGTATTGAATAAACATTTGACGTCCGGATGCTTCCGACGGAAGCTTAGGTTACTTCGTGGCGAAGCAAAACATTTATATATTGGCCTGGATT
GCTGCCTATAAATAGGCAAGCAAGGAAATGGCATTAACAACAGAGCGGGTGAAACTATTCTTCGAATGGTTTCTGTTCATCGGTGCAATATTTATTGCGATAACAATATTATATATATTG
TTGGCGTTGCTCTTTGAGGTCCCCAAGTATATTAAGGATTTTGTGAGGTATCTCGTAGAATACCTGACCAGAAGACGTGTATGGATGCAGAGAACGCAGTTGACGGAGGCAACAGGAGAT
GTAGAGCTCGGCAGAGGTATTGTGGAAGACAGACGGGATCAACAACCGGCTGTCATACCACATGCAACACAGGTTATCCCTTCGCATCAACCTAGAAGGGATGATCAAGGAAGACGAGGA
AACGCAGGACCAATGTTTTAATACACGGTATTGTAATATACGAAATATAAATGGATAATGATATGTATTGTGAAACATAATATACGTGAAACATAATATATGTTTGTTAATTTACATATT
ATAATATGTAAATTGTATACGAGTGTTGTATTTATAGAACATACAACACGCTATGACAAACAGGGAAAAATGAAGAATCGGGGGTTGATTGGTCTATCGTATCGCTTAAGGGCCGCAGGC
CCGTTGAAATGATTCTTAATAAAACAAATATACATGATACGGATAGTTGAATATATAAACAACGAAGTATAAATACAACAGAATGTTGTATACTAATTAAAATAATGAGAAGATAAGTAT
ATTTGTGAGGGATGATGATCACAACCACCACTTTAGTGGTGGGTCATATGTCCCGAGTTAGTGCGCCACGTAAGCGCTGGGGCTTAT

DNA-N
2-14 SNPs to KM607387 KM607385 KM607386 (**China**). **26-30 SNPs** to MF039872 MF039878 MF039866 KY427062 (**Thailand**)

N protein (154 aa): 0 aa to KM607387 KM607385 (**China**) MF039872 MF039878 KY427062 (**Thailand**) **1 aa** to MF039866 (**Thailand**)

>N_alyu56
TATTACCCCCCGTGCTCGGGACGGGACATGACGTCAGCATAGATTATAATGGGCCAACTAAAGGCCCATTTAATAGAAAAGGCGGGCTTTTGACATATTTCAAAGGCCCAGCCTGGAAGC
GGATAATGTCACGTGCCGAATAAGAGGTTGCTTCGCCTCGAAGCAATCCGAATAAATGTTGCGTATTCAATACGCAACTGAAGTCTATTAATATGGTTGTCTCTGCCGAATAAATCAGAG
CGTAAGCGAAGCAGAAGCGATGGATTGGGCAGAATCACAATTCAAGACCTGTACTCATGGGTGCGATTGGAAGACGATATCATCGGATTCATCCGAGAATCGACAATATGTACCTTGCGT
CGACGCAGGCAGTGGAAGAAAGTCACCTCGCAAGGTACTGCTTAGATCTATTGAAGCTGTGTTTAATGGAAGCTTCAAAGGAAATAACAGGAATGTTCGTGGGTTTATATACGTATCAAT
ACGAGACGATGATGGAGAAATGCGTCCAGTACTCATAATACCATTCGGAGGATATGGATATCATAATGATTTCTATTATTTCGAAGGGAAGGGGAAAGTTGAATGTGATATATCATCAGA
TTATGTAGCGCCAGGAGTCGATTGGAGCAGAGACATGGAAGTTAGTATTAGCAACAGCAACAACTGTAATGAATTATGTGATCTGAAGTGTTATGTTGTTTGTTCGTTAAGAATAAAGGA
ATAACAGATGTGCTGTAATGATTATTAATAAAACTTATTTTCATGTAATTGATAGTTGTATAAAACATACAACACGCTATGACAAACAGGGAAAAATGAAAAATCGGGGTTTGATTGTTC

TATAGTATCGCTTAAGGGCCGCAGGCCCGTTGAAAAATAATAATCGAATTATAAACGTTAGATAATAATCAGAGATAGATGATAAGCATCAAAAAACATAGACGAAGTATATGGCTGTAT
AATATAAAAGAAGCATATAAATAAATAAATGTGAACTAATCTCTGATTGGTTCAGAGCGTAGCCCCACCAACTTTATGTTGGTGGAAATGTCCCGATGACGTAAGCACGGGGGACTAT

DNA-R
7-9 SNPs to KM607677 KM607678 KM607676 KM607679 (**China**). **8-14 SNPs** to AB113660 AF416475 AB113659 (**Viet Nam**). **13-17 SNPs** to MF039879 MF039873 MF039867 KY427063 (**Thailand**)

R protein (286 aa): 0 aa to KM607676 KM607677 KM607679 (**China**) **1 aa** to AB113660 (**Vietnam**) MF039879 (**Thailand**) KM607678 (**China**) …

>R_alyu56
TATTACCCCCAGCGCTCGGGACGGGACATTTGCATCTATAAATAGACCTCCCCCCTCTCCACTACAAGATCATCATCGTCGACAGAAATGGCGCGATATGTGGTATGCTGGATGTTCACC
ATCAACAATCCCGCTTCACTACCAGTGATGCGGGATGAGTTCAAATATATGGTATATCAAGTGGAGAGGGGACAGGAGGGTACTCGTCATGTGCAGGGATACGTCGAGATGAAGAGACGA
AGTTCTCTGAAGCAGATGAGAGGCTTCTTCCCTGGCGCACACCTTGAGAAACGAAAGGGAAGCCAAGAAGAAGCACGGGCATACTGTATGAAGGAAGACACAAGAATCGAAGGTCCCTTC
GAGTTTGGTGCCTTTAAATTGTCATGTAATGATAATTTATTTGATGTCATACAGGATATGCGTGAAACGCACAAACGGCCTTTGGAGTATTTATATGAGTGTCCAAATACCTTCGATAGA
AGTAAGGATACATTATACAGAGTTCAAGCAGAGTTGAATAAAACGAAGGCGATGAATAGCTGGAAAACATCCTTCAGTTCTTGGACATCGGAAGTTGAAAATATTATGGCGGAGCCATGT
CATCGAAGGATAATTTGGGTCTATGGCCCAAATGGAGGAGAAGGAAAGACAACCTATGCAAAATATTTAATGAAGACGAAGAATGCGTTTTATTCTCCAGGAGGAAAATCATTGGATATA
TGTAGATTGTATAATTACGAGGAAATAGTTATATTTGATATTCCCAGATGCAAAGAGGAATATTTAAACTATGGTTTATTAGAAGAATTTAAAAATGGAATTATTCAAAGCGGGAAATAT
GAACCCGTTTTGAAAATTGTAGAATATGTGGAAGTCATTGTAATGGCTAACTTCCTTCCGAAGGAAGGAATCTTTTCAGAAGATCGAATAAAGCTAGTTGCTTGCTGAACACGCTATGAC
AATCGTACGCTATGACAAAAGGGGAAAAGCAAAGATTCGGGGGTTGATTGGGCTATCCTAACGATTAAGGGCCGCAGGCCCGTCAAGATGGACGGCTTGATCAGATGTCCCGAGTTAGTG
CGCCACGTAAGCGCTGGGGCTTAT

DNA-S
11-13 SNPs to KM607534 KM607535 KM607536 KM607533 (**China**). **14-19 SNPs** to AB113662 AB113661 (**Viet Nam**). **16 SNPs** to AF148945 (**Viet Nam**). **20-30 SNPs** to MF039874 KY427064 MF039880 (**Thailand**)

S protein (170 aa): 0 aa to AY337715 AY494786 HQ378191 (**China**) FJ787433 FJ787434 (**Philippines**) **1 aa** to several isolates

>S_alyu56
TATTACCCCCAGCGCTCGGGACGGGACATGGGCTAATGGATCATGGAATAAGGGCCCATCGGGCCCGTTATGATGGGTTTTGGGCTGATGGGCTTTATCCAGAAGACCAAAAACAGGCGG
GAACCGTCCCAATTTCAAATTTAGATAGCTTGCCCCGCAAGCAAACTAAAAGTCTATATATACCAGTGTAGACATATTGTTCAGATAACAAATGGCGAGGTTTCCGAAGAAATCGATCAA
GAAGAGGCGGGTTGGGCGACGGAAGTATGGAAGCAAGGCGGCAACCAGTCACGACTACTCGTCGTTAGGTTCAATATTGGTTCCTGAAAATACCGTCAAGGTATTTAGGATTGAGCCTAC
TGATAAAACATTACCCAGATATTTTATCTGGAAAATGTTTATGCTTTTGGTGTGCAAGGTGAAGCCCGGAAGAATACTTCACTGGGCTATGATTAAAAGTTCATGGGAAATCAACCAGCC
GACTACATGTCTGGAAGCACCAGGTTTATTTATAAAACCTGAACATAGCCATCTGGTGAAACTGGTATGCAGTGGGGAACTTGAAGCCGGAGTCGCAACAGGGACATCAGATGTTGAATG
TCTTCTAAGGAAGACAACCGTGTTGAGGAAGAATGTAACAGAGGTGGATTACTTGTATTTGGCATTTTATTGTAGTGCTGGAGTTAGTATTAACTACCAGAACAGAATTACATATCATGT
ATGATATGTTTATGTAAACATAAACCTTTGTATGGAATAATGACCAAATAACATACAACACACTATGAAATACAACACGCTATGACAAACAGGGAAAAATGAAGAATCGGGGGTTGATTG
GTCTATCGTATCGCTTAAGGGCCGCAGGCCCGTTGAAATGATTCTTTATACAACAAATATACATGATACGGATAGTTGAATATATAAACAACGATGTATAAATACAACAGAATGTTGTAT
ACTAATTAAAATAATGAGAAGAAAAGGATATTTGTGAAGGATAAGCATCAGAACCACCACTTTAGTGGTGGGTCAGATGTCCCGAGTTAGTGCGCCACGTAAGCGCTGGGGCTTAT

DNA-U3
42/44/46/50 SNPs to MF039875 MF039881 KY427065 MF039869 (**Thailand**)

U3 protein (39 aa): 0 aa to KY427065 (**Thailand)**

>U3_alyu56
TATTACCCCCAGCCCCCAGGACGGGACATGGGCTTTTTAAATGGGCCGAGAGAGTTTGAACAGTTCAGTATCTTCGTTATTGGGCCATCTGGCCCAATAATTAAGAGAACGTGTTCAAAA
TCGGGGTTTGACCGAAGGTCAAGGTAGACGGTCAACATTATTCTGGCTTGCGGAGCAAGCAACACGAATTAAATATTTAATTCGTATGACACGTGGACGGACCGAAATACTCCTGTATCT
CTATAAATAGCCTAAATCTGGCTTGGATAATTGCTCTCGCTCTTCTGTCAAAGCTGCTGTGTTGAGGCGGAAGATCGCCATCGGCGATCATCGGACGAAAAGCTGCAAGAGAGACGGAGA
ACCATGCTACGAAGCGTATATCGGGTATTTATAGACTTCTAGCGCAGCTAGAAGTTTCCTTGTACTTGATATTGTATTTTGTAAATTACGAAGAAATTCGTATATTGATAATAATAAAAC
ATCTGGGATTGTTAATGTTTACATTAACCAGTATTAATAATGTACAATAACAAATATTGTATGAGGAACGAATACAAGATTATTAAAATATGAGGAGCGTAGCGTGATAAACAGGTGTTT
AAGGTATAATTAAATTAATTATGTAATGTCATTATAATACGGTATAAGTTGAAGTATGAGGTGAAAGAGGAGATATTAGAATATTAAAAACCCAATTATATTATTTATAATACATACAAC
ACGCTATGACAAACGGGGAAAAAAGAAAAATCGGGGGTTGATTGGTCTATCCTAACGATTAAGGGCCGCAGGCCCGTCAAGATGGATGAACGGTTAGATTTGATTGCTTAGCTACGAAGA
AAGGAATCGTTTTGGGACCACAGACAAGACAGCTGTCACTACTTTTAAAAAATAATATAATAACCAATTGACGATAGTACCCCTACCAAAGATGACGTATAGGGGTGTCCCGAGGTAATT
TAACATAGCTCTGACAAGAGATAGTGGACGTTGGATGCCACGATCGGACGATCGTGGTTTGATGAACCAATGATGACGTAGGGGCTGGGGCTTAT

Dataset S1B. DNA-U3 components of BBTV PIO (JGF-1, ALYU-14 to ALYU-24) and SEA (ALYU-25 to ALYU-56) isolates and their multiple alignment with conserved features highlighted

Conserved **TATA-boxes** and **poly(A) signal**

miR156 family target sequence: TTGCTCTCTCTCTTCTGTCAA in PIO isolates; TTGCTCTCGCTCTTCTGTCAA in SEA isolates

>U3_jgf1_v1
TATTACCCCCCGTGCTCGGGACGGGACATGGGCTTTTTAAATGGGCCTTGAGAGTTTGAACAGTTCAGTATCTTCGTTATTGGGCCAACTCGGCC**CAATAATTAA**GAGAACGTGTTCAAA
TTCGTGGTATGACCGAAGGTCAAGGTAACCGGTCAACATTATTCTGGCTTGCGCAGCAAGATACACGAATTAAATTATTAATTCGTAGGACACGTGGACGGACCGAAATACTCCTGCATC
T**CTATAAATA**CCCTAGTCCTGTGAAGGATAATTGCTCTCTCTCTTCTGTCAAGGTGGTTGTGCTGAGGCGGAAGATCGCCAGCGGCGATCGTCGGAACGATGTGCATCTAGAGAGGCGGT
GAAGCAAACTACGAAGCGTATATCGGGTATTTATAGACTTATAGCGCAGCTAGAAGTATACACTGTACAGATTATGTATTTTGTAAATTACGAAGAATTTCGTATATGATATT**AATAAA**A
CACCTGGGTGTGTTAATGTTTACATTAAATAGTATCCTGAATGTACACAATAAAATACAGTATACGGAACGTATACTGACAAAGTAATAAATGATAGGCGAAGCATGATTAACAGGTGTT
TAGGTATAATTAACATAATTATGAGAAGTAATAATAATACGGAAAATGAATAAGTATGAGGTGAAAGAGGAGATATTAGAATATTTAAAAACCCAATTATATTATTTTGGAACGAAATAC
AACACGCTATGAAATACAAGACGCTATGACAAATGTACGGGTATCTGATTGTATATCTTAACGATCTAGGGCCGTAGGCCCGTGAGCAATGAACGGTCCAGATTAATTCCTTAGCGACGA
AGAAAGGAATCTTAATAGGGACCACATTAAAGACAGCTGTCATTGATTAAATAAATAATATAATATACAAAAGACTTTTGTACCCCTGATAATGATGACGTGTAGGGGTGTCCCGATGTA
ATATAGCATAGCTATGTGAAGAGATAAGCATGTGGCCGACAACTTTAGTGGTGGGCCAGATGTCCCGAGTTAGTGCGCCACGTAAGCACGGGGGACTAT

>U3_jgf1_v2
TATTACCCCCCGTGCTCGGGACGGGACATGGGCTTTTTAAATGGGCCTTGAGAGTTTGAACAGTTCAGTATCTTCGTTATTGGGCCAACTCGGCC**CAATAATTAA**GAGAACGTGTTCAAA
TTCGTGGTATGACCGAAGGTCAAGGTAACCGGTCAACATTATTCTGGCTTGCGCAGCAAGATACACGAATTAAATTATTAATTCGTAGGACACGTGGACGGACCGAAATACTCCTGCATC
T**CTATAAATA**CCCTAGTCCTGTGAAGGATAATTGCTCTCTCTCTTCTGTCAAGGTGGTTGTGCTGAGGCGGAAGATCGCCAGCGGCGATCGTCGGAACGATGTGCATCTAGAGAGGCGGT
GAAGCAAACTACGAAGCGTATATCGGGTATTTATAGACTTATAGCGCAGCTAGAAGTATACACTGTACAGATTATGTATTTTGTAAATTACGAAGAATTTCGTATATGATATT**AATAAA**A
CACCTGGGTGTGTTAATGTTTACATTAAATAGTATCCTGAATGTACACAATAAAATACAGTATACGGAACGTATACTGACAAAGTAATAAATGATAGGCGAAGCATGATTAACAGGTGTT
TAGGTATAATTAACATAATTATGAGAAGTAATAATAATACGGAAAATGAATAAGTATGAGGTGAAAGAGGAGATATTAGAATATTTAAAAACCCAATTATATTATTTTGGAACGAAATAC
AACACGCTATGAAATACAAGACGCTATGACAAATGTACGGGTATTTGATTGTATATCTTAACGATCTAGGGCCGTAGGCCCGTGAGCAATGAACGGTATAGATTAATTCCTTAGCGACGA
AGAAAGGAATCTTAATAGGGACCACATTAAAGACAGCTGTCATTGATTAAATAAATAATATAATATACAAAAGACTTTTGTACCCCTGATAATGATGACGTGTAGGGGTGTCCCGATGTA
ATATAGCATAGCTATGTGAAGAGATAAGCATGTGGCCGACAACTTTAGTGGTGGGCCAGATGTCCCGAGTTAGTGCGCCACGTAAGCACGGGGGACTAT

>U3_alyu14
TATTACCCCCcGtGCTCGGGACGGGACATGGGCTTTTTAAATGGGCCTTGAGAGTTTGAACAGTTCAGTATCTTCGTTATTGGGCCAACTCGGCC**CAATAATTAA**GAGAACGTGTTCAAA
TTCGTGGTATGACCGAAGGTCAAGGTAACCGGTCAACATTATTCTGGCTTGCGCAGCAAGCTACACGAATTAAATTATTAATTCGTAGGACACGTGGACGGACCGAAATACTCCTGCATC
T**CTATAAATA**CCCTAGTCTTGTGAAGGATAATTGCTCTCTCTCTTCTGTCAAGGTGGTTGTGCGGAGGCGGAAGATCGCCAGCGGCGATCGTCGGAACGATGTGCATCTAGAGAGGCGGT
GAAGGAAACTACGAAGCATATATCGGGTATTTATAGACTTATAGCGCAGCTAGAAGTATACACTGTACAGATTTTGTATTTTGTAAATATACGAAGAATTTCGTATATGATATT**AATAAA**
ACACCTGGGTGTTTTTAATGTTTACATTAAATAGTATCCTGAATGTACACAATAAAATACAGTATACGGAACGTATACTGACAAAGTAATAAATGATAGGCGAAGCATGATTAACAGGTG
TTTAGGTATAATTAACATAATTATGAGAAGTAATTATAATACGGAAAATGAATAAGTATGAGGTGAAAGAGGAGATATTAGAATATTTAAAAACCCAATTATATTATTTTGGAACGAAAT

ACAACACGCTATGAAATACAAGACGCTATGACAAATACGGGTATCTGATTGTTTATCTTAACGCTTAAGGGCCGCAGGCCCGTCAAGTTGAATGAACGGTCCAGATTAATTCCTTAGCGA
CGAAGAAAGGAATCTTAATGGGGATAACATTAAAGACAGCTGTCATTGATTAAATAAATAATATAATATACAAAAGACTTATGTACCCCTGATAATGATGACGTGTAGGGGTGTCCCGAT
GTAATATAGCATAGCTATGTGAAGAGATAAGCATGTGGCCGACAACTTTAGTTGTGGGCCAGATGTCCCGAGTTAGTGCGCCACGTAAGCaCgGGGGACTAT

>U3_alyu15
TATTACCCCCCGTGCTCGGGACGGGACATGGGCTTTTTAAATGGGCCTTGAGAGTTTGAACAGTTCAGTATCTTCGTTATTGGGCCAACTCGGCC**CAATAATTAA**GAGAACGTGTTCAAA
TTCGTGGTATGACCGAAGGTCAAGGTAACCGGTCAACATTATTCTGGCTTGCGCAGCAAGATACACGAATTAAATTATTAATTCGTAGGACACGTGGACGGACCGAAATACTCCTGCATC
T**CTATAAATA**CCCTAGTCTTGTGAAGGATAATTGCTCTCTCTCTTCTGTCAAGGTGGTTGTGCGGAGGCGGAAGATCGCCAGCGGCGATCGTCGGAACGATGTGCATCTAGAGAGGCGGT
GAAGGAAACTACGAAGCATATATCGGGTATTTATAGACTTATAGCGCAGCTAGAAGTATACACTGTACAGATTTTGTATTTTGTAAATATACGAAGAATTTCGTATATGATATT**AATAAA**
ACACCTGGGTGTTTTAATGTTTACATTAAATAGTATCCTGAATGTACACAATAAAATACAGTATACGGAACGTATACTGACAAAGTAATAAATGATAGGCGAAGCATGATTAACAGGTGT
TTAGGTATAATTAACATAATTATGAGAAGTAATTATAATACGGAAAATGAATAAGTATGAGGTGAAAGAGGAGATATTAGAATATTTAAAAACCCAATTATATTATTTTGGAACGAAATA
CAACACGGTATGAAATACAAGACGCTATGACAAATACGGGTATCTGATTGTTTATCTTAACGCTTAAGGGCCGCAGGCCCGTCAAGTTGAATGAACGGTCCAGATTAATTCCTTAGCGAC
GAAGAAAGGAATCTTGATGGGGATCACATTAAAGACAGCTGTCATTGATTAAATAAATAATATAATATACAAAAGACTTATGTACCCCTGATAATGATGACGTGTAGGGGTGTCCCGATG
TAATATAGCATAGCTATGTGAAGAGATAAGCATGTGGCCGACAACTTTAGTTGTGGGCCAGATGTCCCGAGTTAGTGCGCCACGTAAGCACGGGGGACTAT

>U3_alyu16
TATTACCCCCcGtGCTCgGGACGGGACATGGGCTTTTTAAATGGGCCTTGAGAGTTTGAACAGTTCAGTATCTTCGTTATTGGGCCAACTCGGCC**CAATAATTAA**GAGAACGTGTTCAAA
TTCGTGGTATGACCGAAGGTCAAGGTAACCGGTCAACATTATTCTGGCTTGCGCAGCAAGATACACGAATTAAATTATTAATTCGTAGGACACGTGGACGGACCGAAATACTCCTGCATC
T**CTATAAATA**CCCTAGTCCTGTGAAGGATAATTGCTCTCTCTCTTCTGTCAAGGTGGTTGTGCTGAGGCGGAAGATCGCCAGCGGCGATCGTCGGAACGATGTGCATCTAGAGAGGCGGT
GAAGCAAACTACGAAGCATATATCGGGTATTTATAGACTTATAGCGCAGCTAGAAGTATACACTGTACAGATTTTGTATTTTGTAAATTACGAAGAATTTCGTATATGATATT**AATA**G**A**A
CACCTGGGTGTGTTAATGTTTACATTAAATAGTATCCTGAATGTACACAATAAAATACAGTATACGGAACGTATACTGACAAAGTAATAAATGATAGGCGAAGCATGATTAACAGGTGTT
TAGGTATAATTAACATAATTATGAGAAGTAATAATAATACGGAAAATGAATAAGTATGAGGTGAAAGAGGAGATATTAGAATATTTAAAAACCCAATTATATTATTTTGGAACGAAATAC
AACACGCTATGAAATACAAGACGCTATGACAAATGTACGGGTATCTGATTGTGTATCTGAACGCTTAAGGGCCGCAGGCCCGTCAAGTTGAATGAACGGTCCAGATTAATTCCTTAGCGA
CGAAGAAAGGAATCTTAATGGGGACCATATTAAAGACAGCTGTTATTGATTAAATAAATAATATAATATCCAAAAGACTTATGTACCCCTGATAATGTTGACGTGTAGGGGTGTCCCGAT
GTAATATAGCATAGCTATGTGAAGAGATAAGCATGTGGCCGACAACTTTAGTGGTGGGCCAGATGTCCCGAGTTAGTGCGCCACGTAAGCaCgGGGGacTAT

>U3_alyu17_v1
TATTACCCCCaGcGCTCaGGACGGGACATGGGCTTTTTAAATGGGCCTTGAGAGTTTGAACAGTTCAGTATCTTCGTTATTGGGCCAACCCGGCC**CAATAATTAA**GAGAACGTGTTCAAA
TTCGTGGTATGACCGAAGGTCAAGGTAACCGGTCAACATTATTCTGGCTTGCGCAGCAAGATACACGAATTAAA**TAA**TTAATTCGTAGGACACGTGGACGGACCGAAATACTCCTGCATC
T**CTATAAATA**CCCTAGTCCTGTGAAGGATAATTGCTCTCTCTCTTCTGTCAAGGTGGTTGTGCTGAGGCGGAAGATCGCCAGCGGCGATCGTCGGAACGATGTGCATCTAGAGAGGCGGT
GAAGgAAACTACGAAGCATATATCGGGTATTTATAGACTTATAGCGCAGCTAGAAGTATACACTGTACAGATTtTGTATTTTGTAAATTACGAAGAATATCGTATATGATATT**AATAAA**A
CACCTGGGTGTGTTAATGTTTACATTAAATAGTATCCTGAATGTACACAATAAAATACAGTATACGGAAcGTATACTGACAAAAGTAATACATGATAGGCGAAGCATGATTAACAGGTGT
TTAGGTATAATTAACATAATTATGAGAAGTAATAATAATACGGAAAATGAATAAGTATGAGGTGAAAGAGGAGATATTAGAATATTTAAAAACCCAATTATATTATTTTGGAACGAAATA
CAACACACTATGAAATACAAGACGCTATGACAAATGTACGGGTATCTGATTGTGTATCTTAACGATCTAGGGCCGTAGGCCCGTGAGCAATGAACGGTCCAGATTAATTCCTTAGCGACG
AAGAAAGGAATCTTAGTGGGACCATATTAAAGACAGCTGTTATTGATTAAATAAATAATATAATATCCAAAAGACTTTTGTACCCCTGATAATGATGACGTGTAGGGGTGTCCCGATGTA
ATATAGCATAGCTATGTGAAGAGATAAGCATGTGGCCGACAACTTTAGTgGTGGGCCAGATGTCCCGAGTTAGTGCGCCACGTAAGCgCtGGGGctTAT

>U3_alyu17_v2
TATTACCCCCCGTGCTCGGGACGGGACATGGGCTTTTTAAATGGGCCTTGAGAGTTTGAACAGTTCAGTATCTTCGTTATTGGGCCAACCCGGCC**CAATAATTAA**GAGAACGTGTTCAAA
TTCGTGGTATGACCGAAGGTCAAGGTAACCGGTCAACATTATTCTGGCTTGCGCAGCAAGATACACGAATTAAATtATTAATTCGTAGGACACGTGGACGGACCGAAATACTCCTGCATC
T**CTATAAATA**CCCTAGTCCTGTGAAGGATAATTGCTCTCTCTCTTCTGTCAAGGTGGTTGTGCTGAGGCGGAAGATCGCCAGCGGCGATCGTCGGAACGATGTGCATCTAGAGAGGCGGT
GAAGCAAACTACGAAGCATATATCGGGTATTTATAGACTTATAGCGCAGCTAGAAGTATACACTGTACAGATTATGTATTTTGTAAATTACGAAGAATATCGTATATGATATT**AATAAA**A
CACCTGGGTGTGTTAATGTTTACATTAAATAGTATCCTGAATGTACACAATAAAATACAGTATACGGAAcGTATACTGACAAAAGTAATACATGATAGGCGAAGCATGATTAACAGGTGT
TTAGGTATAATTAACATAATTATGAGAAGTAATAATAATACGGAAAATGAATAAGTATGAGGTGAAAGAGGAGATATTAGAATATTTAAAAACCCAATTATATTATTTTGGAACGAAATA
CAACACACTATGAAATACAAGACGCTATGACAAATGTACGGGTATCTGATTGTGTATCTTAACGATCTAGGGCCGTAGGCCCGTGAGCAATGAACGGTCCAGATTAATTCCTTAGCGACG
AAGAAAGGAATCTTAGTGGGACCATATTAAAGACAGCTGTTATTGATTAAATAAATAATATAATATCCAAAAGACTTTTGTACCCCTGATAATGATGACGTGTAGGGGTGTCCCGATGTA
ATATAGCATAGCTATGTGAAGAGATAAGCATGTGGCCGACAACTTTAGTAGTGGGCCAGATGTCCCGAGTTAGTGCGCCACGTAAGCACGGGGGACTAT

>U3_alyu18
TATTACCCCCCGTGCTCGGGACGGGACATGGGCTTTTTAAATGGGCCTTGAGAGTTTGAACAGTTCAGTATCTTCGTTATTGGGCCAACTCGGCC**CAATAATTAA**GAGAACGTGTTCAAA
TTCGTGGTATGACCGAAGGTCAAGGTAACCGGTCAACATTATTCTGGCTTGCGCAGCAAGATACACGAATTAAATTATTAATTCGTAGGACACGTGGACGGACCGAAATACTCCTGCATC
T**CTATAAATA**CCCTAGTCCTGTGAAGGATAATTGCTCTCTCTCTTCTGTCAAGGTTGTTGTGCTGAGGCGGAAGATCGCCAGCGGCGATCGTCGGAACGATGTGCATCTAGAGAGGCGGT
GAAGCAAACTACGAAGCATATATCGGGTATTTATAGACTTATAGCGCAGCTAGAAGTATACACTGTACAGATATTGTATTTTGTATCATTTACGAAGAATTTCGTATATGATATT**AATAA**
AACACCTGGGTGTTTTAATGTTTACATTAAATAGTATCCTGAATGTACACAATAAAATACAGTATACGGAACGTATACTGACAAAGTAATAAATGATAGGCGAAGCATGATTAACAGGTG
TTTAGGTATAATTAACATAATTATGAGAAGTAATAATAATACGGAAAATGAATAAGTATGAGGTGAAAGAGGAGATTTTAGAATATTTAAAAACCCAATTATATTATTTTGGAACGAAAT
ACAACACGCTATGAAATACAAGACGCTATGACAAATGTACGGGTATCTGATTGTGTATCTGAACGCTTAAGGGCCGCAGGCCCGTCAAGATGAATGAACGGTCCAGATTAATTCCTTAGC
GACGAAGAAAAGAATCTTAATGGGGATCACATTAAAGACAGCTGTCATTGATTAAATAAATAATATAATATACAAAAGACTTTTGTACCCCTGATAATGATGACGTGTAGGGGTGTCCCG
ATGTAATATAGCATAGCTATGTGAAGAGATAAGCATGTGGCCGACAACTTTAGTGGTGGGCCAAATGTCCCGAGTTAGTGCGCCACGTAAGCACGGGGGACTAT

>U3_alyu19
TATTACCCCCAGCGCTCAGGACGGGACATGGGCTTTTTAAATGGGCCTTGAGAGTTTGAACAGTTCAGTATCTTCGTTATTGGGCCAACTCGGCC**CAATAATTAA**GAGAACGTGTTCAAA
TTCGTGGTATGACCGAAGGTCAAGGTAACCGGTCAACATTATTCTGGCTTGCGCAGCAAGATACACGAATTAAATTATTAATTCGTAGGACACGTGGACGGACCGAAATACTCCTGCATC
T**CTATAAATA**CCCTAGTCCTGTGAAGGATAATTGCTCTCTCTCTTCTGTCAAGGTGGTTGTGCTGAGGCGGAAGATCGCCAGCGGCGATCGTCGGAACGATGTGCATCTAGAGAGGCGGT
GAAGCAAACTACGAAGCATATATCGGGTATTTATAGACTTATAGCGCAGCTAGAAGTATATACTGTACAGATTTTGTATTTTGTAAATTACGAAGAATATCGTATATGATATT**AATAAA**A
CACCTGGGTGTGTTAATGTTTACATTAAATAGTATCCTGAATGTACACAATAAAATACAGTATACGGAACGTATACTGACAAAGTAATAAATGATAGGCGAAGCATGATTAACAGGTGTT
TAGGTATAATTAACATAATTATGAGAAGTAATAATAATACGGAAAATGAATAAGTATGAGGTGAAAGAGGAGATATTAGAATATTTAAAAACCCAATTATATTATTTTGGAACGAAATAC
AACACGCTATGAAATACAAGACGCTATGACAAATGTACGGGTATCTGATTGGTTATCTTAACGATCTAGGGCCGTAGGCCCGTGAGCAATGAACGGTCCAGATTAATTCCTTAGCGACGA
AGAAAGGAATCTTAATAGGGACCACATTAAAGACAGCTGTCATTGATTAAATAAATAATATAATAACCAAAAGACTTTTGTACCCCTGATAATGATGACGTGTAGGGGTGTCCCGATGTA
ATATAGCATAGCTATGTGAAGAGATAAGCATTTGGCCGACAACTTTAGTGGTGGGCCAGATGTCCCGAGTTAGTGCGCCACGTAAGCGCTGGGGCTTAT

>U3_alyu21_v1
TATTACCCCCAGCGCTCGGGACGGGACATGGGCTTTTTAAATGGGCCTTGAGAGTTTGAACAGTTCAGTATCTTCGTTATTGGGCCAACTCGGCC**CAATAATTAA**GAGAACGTGTTCAAA
TTCGTGGTATGACCGAAGGTCAAGGTAACCGGTCAACATTATTCTGGCTTGCGCAGCAAGATACACGAATTAAATTATTAATTCGTAGGACACGTGGACGGACCGAAATACTCCTGCATC
T**CTATAAATA**CCCTAGTCATGTGAAGGATAATTGCTCTCTCTCTTCTGTCAAGGTGGTTGTGCTGAGGCGGAAGATCGCCAGCGGCGATCGTCGGAACGATGTGCATCTAGAGAGGCGGT
GAAGCAAACTACGAAGCATATATCGGGTATTTATAGACTTATAGCGCAGCTAGAAGTATACACTGTACAGATTTTGTATTTTGTAAATTACGAAGAATTTCGTATATGATATT**AATAAA**A
CACCTGGGTGTGTTAATGTTTACATTAAATTGTATCCTGAATGTACACAATAAAATACAGTATACGGAACGTATACTGACAAAGTAATAAATGATAGGCGAAGCATGATTAACAGGTGTT
TAGGTATAATTAACATAATTATGAGAAGTAATAATAATACGGAAAATGAATAAGTATGAGGTGAAAGAGGAGATATTAGAATATTTAAAAACCCAATTATATTATTTTGGAACGAAATAC
AACACACTATGAAATACAAGGCGCTATGACAAATGTACGGGTATTTGATTGTGTATCTTAACGCTTAAGGGCCGCAGGCCCGTCAAGTTGAATGAACGGTCTAGATTAATTCCTTAGCGA
CGAAGAAAGGAATCTTAGTGGGGACCATATTAAAGACAGCTGTCATTGATTAAATAAATAATATAATATACAAAAGACTATTGTACCCCTGATAATGATGACGTGTAGGGGTGTCCCGAT
GTAATATAGCATAGCTATGTGAAGAGATAAGCATGTGGCCGACAACTTTAGTGGTGGGCCAGATGTCCCGAGTTAGTGCGCCACGTAAGCGCTGGGGACTAT

>U3_alyu21_v2
TATTACCCCCCGTGCTCGGGACGGGACATGGGCTTTTTAAATGGGCCTTGAGAGTTTGAACAGTTCAGTATCTTCGTTATTGGGCCAACTCGGCC**CAATAATTAA**GAGAACGTGTTCAAA
TTCGTGGTATGACCGAAGGTCAAGGTAACCGGTCAACATTATTCTGGCTTGCGCAGCAAGATACACGAATTAAATTATTAATTCGTAGGACACGTGGACGGACCGAAATACTCCTGCATC
T**CTATAAATA**CCCTAGTCATGTGAAGGATAATTGCTCTCTCTCTTCTGTCGAGGTGGTTGTGCTGAGGCGGAAGATCGCCAGCGGCGATCGTCGGAACGATGTGCATCTAGAGAGGCGGT
GAAGCAAACTACGAAGCATATATCGGGTATTTATAGACTTATAGCGCAGCTAGAAGTATACACTGTACAGATTTTGTATTTTGTAAATTACGAAGAATTTCGTATATGATATT**AATAAA**A
CACCTGGGTGTGTTAATGTTTACATTAAATTGTATCCTGAATGTACACAATAAAATACAGTATACGGAACGTATACTGACAAAGTAATAAATGATAGGCGAAGCATGATTAACAGGTGTT
TAGGTATAATTAACATAATTATGAGAAGTAATAATAATACGGAAAATGAATAAGTATGAGGTGAAAGAGGAGATATTAGAATATTTAAAAACCCAATTATATTATTTTGGAACGAAATAC
AACACACTATGAAATACAAGGCGCTATGACAAATGTACGGGTATTTGATTGTGTATCTTAACGCTTAAGGGCCGCAGGCCCGTCAAGTTGAATGAACGGTCTAGATTAATTCCTTAGCGA
CGAAGAAAGGAATCTTAGTGGGGACCATATTAAAGACAGCTGTCATTGATTAAATAAATAATATAATATTCAAAAGACTATTGTACCCCTGATAATGATGACGTGTAGGGGTGTCCCGAT
GTAATATAGCATAGCTATGTGAAGAGATAAGCATGTGGCCGTGGTGGGCCAGATGTCCCGAGTTAGTGCGCCACGTAAGCACGGGGGACTAT

>U3_alyu22
TATTACCCCCCGTGCTCGGGACGGGACATGGGCTTTTTAAATGGGCCTTGAGAGTTTGAACAGTTCAGTATCTTCGTTATTGGGCCAACtCGGCC**CAATAATTAA**GAGAACGTGTTCAAA
TTCGTGGTATGACCGAAGGTCAAGGTAACCGGTCAACATTATTCTGGCTTGCGCAGCAAGATACACGAATTAAATTATTAATTCGTAGGACACGTGGACGGACCGAAATACTCCTGCATC

T**CTATAAATA**CCCTAGTCCTGTGAAGGATAATTGCTCTCTCTCTTCTGTCAAGGTGGTTGTGCTGAGGCGGAAGATCGCCAGCGGCGATCGTCGGAACGATGTGCATCTAGAGAGGCGGT
GAAGCAAACTACGAAGCATATATCGGGTATTTATAGACTTATAGCGCAGCTAGAAGTATACACTGTACAGATTTTGTATTTTGTAaATTACGAAGAATTTCGTATATGATATT**AATAAA**A
CACCTGGGTGTGTTAATGTTTACATTAAATAGTATCCTGAATGTACACAATAAAATACAGTATACGGAACGTATACTGACAAAGTAATAACTGATAGGCGAAGCATGATTAACAGGTGTT
TAGGTATAATTAACATAATTATGAGAAGTAATAATAATACGGAAAATGAATAAGTATGAGGTGAAAGAGGAGATATTAGAATATTTAAAAACCCAATTATATTATTTTGGAACGAAATAC
AACACGCTATGAAATACAAGACGCTATGACAAATGTACGGGTATCTGATTGTGTATCTTAACGCTTAAGGGCCGCAGGCCCGTCAAGTTTGATGAACGGTCCAGATTAATTCCTTAGCGA
CGAAGAAAGGAATCTTAgTGGGGACCATATTAAAGACAACTGTcATTGATTAAATAAATAATATAATATCCAAAAGACTTTTGTACCCCTGAgAATGATGACGTGTAGGGGTGTCCCGAT
GTAATATAGCATAGCTATGCGAAGAGATAAGCATGTGGCCGACAACTTTAGTGGTGGGCCAGATGTCCCGAGTTAGTGCGCCACGTAAGCACGGGGGACTAT

>U3_alyu24
TATTACCCCCCGTGCTCGGGACGGGACATGGGCTTTTTAAATGGGCCTTGAGAGTTTGAACAGTTCAGTATCTTCGTTATTGGGCCAACTCGGCC**CAATAATTAA**GAGAACGTGTTCAAA
TTCGTGGTATGACCGAAGGTCAAGGTAACCGGTCAACATTATTCTGGCTTGCGCAGCAAGATACACGAATTAAATTATTAATTCGTAGGACACGTGGACGGACCGAAATACTCCTGCATC
T**CTATAAATA**CCCTAGTCCTGTGAAGGATAATTGCTCTCTCTCTTCTGTCAAGGTGGTTGTGCTGAGGCGGAAGATCGCCAGCGGCGATCGTCGGAACGATGTGCATCTAGAGAGGCGGT
GAAGCAAACTACGAAGCATATATCGGGTATTTATAGACTTATAGCGCAGCTAGAAGTATACACTGTACAGATTTTGTATTTTGTACATTACGAAGAATTTCGTATATGATATT**AATAAA**A
CACCTGGGTGTGTTAATGTTTACATTAAATAGTATCCTGAATGTACACAATAAAATACAGTATACGGAACGTATACTGACAAAGTAATAACTGATAGGCGAAGCATGATTAACAGGTGTT
TAGGTATAATTAACATAATTATGAGAAGTAATAATAATACGGAAAATGAATAAGTATGAGGTGAAAGAGGAGATATTAGAATATTTAAAAACCCATTTATATTATTTTGGAACGAAATAC
AAgACGCTATGAAATACAAGACGCTATGACAAATGTACGGGTATCTGATTGGGTTATCTTAACGATCTAGGGCCGTAGGCCCGTGAGCAATGAACGGTCCAGATTAATTCCTTAGCGACG
AAGAAAGGAATCTTAGTGGGGACCATATTAAAGACAACTGTTATTGATTAAATAAATAATATAATATCTAAAAGACTTTTGTACCCCTGAGAATGATGACGTGTAGGGGTGTCCCGATGT
AATATAGCATAGCTATGCGAAGAGATAAGCATGTGGCCGACAACTTTAGTGGTGGGCCAGATGTCCCGAGTTAGTGCGCCACGTAAGCACGGGGGACTAT

>U3_alyu25
TATTACCCCCAGCCCCCAGGACGGGACATGGGCTTTTTAAATGGGCCGAGAGAGTTTGAACAGTTCAGTATCTTCGTTATTGGGCCATCTGGCC**CAATAATTAA**GAGAACGTGTTCAAAA
TCGGGGTTTGACCGAAGGTCAAGGTAGACGGTCAACAATATTCTGGCTTGCGGAGCAAGCAACACGAATTAAATATTTAATTCGTAAGACACGTGGACGGACCGAAATACTCCTGTATCT
CTATAAATAGCTTAAATCTGGCTTGGATAATTGCTCTCGCTCTTCTGTCAAAGGTGTTGTGTTGAGGCGGAAGATCGCCATCGGCGATCATCGGACGAAAAGCTGCAAGAGAGACGGAGA
ACCATGCTACGAAGCGTATATCGGGTATTTATAGACTTCTAGCGCAGCTAGAAGTATCCTTGTACTTGATATTGTATTTTGTAAATTACGAAGAAATTCGTATATTGATAAT**AATAAA**AC
ATCTGGGACTGTTAATGTTTACATTAACCAGTATTAATAATGTATAATAACAAATATTGTATGAGGAACGAATACAATAATATTAAAATATGTGGAGCGTAGCGTGATAAACAGGTGTTT
AAGGTATAATTAAATTAATTATGCAATGTCATGATAATACGGTATAAGTTGAAGTATGAGGTGAAAGAGGAGATATTAGAATATTAAAAACCCAATTATATTATTTATAATACATACAAC
ACGCTATGACAAACGGGGAAAAAAGAAAAATCGGGGGTTGATTGGTCTATCCTAACGATTAAGGGCCGCAGGCCCGTCAAGATGGATGAACGGTTAGATTTGATTGCTTAGCCACGAAGA
AAGGAATCGTTTTGGGACCACTGACAAGACAGCTGTCACTACTTTTAAAAAATAATATAATAACTAATTGACGATAGTACCCCTCCCAAAGATGACGTATAGGGGTGTCCCGAGGTAATT
TAACATAGCTCTGACAAGAGATAGTGGACGTTGGATGCCACGATCGGACGATCGTGGTTTGATGAACTAATGATGACGTAGGGGCTGGGGCTTAT

>U3_alyu26
TATTACCCCCAGCCCCCAGGACGGGACATGGGCTTTTTAAATGGGCCGAGAGAGTTTGAACAGTTCAGTATCTTCGTTATTGGGCCATCTGGCC**CAATAATTAA**GAGAACGTGTTCAAAA
TCTGGGTTTGACCGAAGGTCAAGGTAGACGGTCAACAATATTCTGGCTTGCGGAGCAAGCAACAAGAATTAAATATTTAATTCGTATGACACGTGGACGGACCGAAATACTCCTGCATCT
CTATAAATAGCCTAAAACTGGCATGGATAATTGCTCTCGCTCTTCTGTCAAAGCTATTGTGTTGAGGCGGAAGATCGCCATCGGCGATCATCGGACGAAAAGCTGCAAGAGAGACGGATA
ACCATGCTGCGAAGCGTATATCGGGTATTTATAGACTTCTAGCGCAGCTAGAAGTTTCCTTGTATTTGATATTGTATTTTGTAAATTACGAAGAAATTCGTACATTGATAAT**AATAAA**AC
ATCTGGGATTGTTAATGTTTACATTAACCAGTATTAATAATGTACAATAACAAATATTGTATGAGGAACGAATACAATAATATTAAAATATGAGGAGCGTAGCGTGATAAACAGGTGTTT
AAGGTATAATTAAATTAATTATGCAATGTAATGATAATACGGTATAAGTTGAAGTATGAGGTGAAAGAGGAGATATTAGAATATTAAAAACCCAATTATATTATTTATAATACATACAAC
ACGCTATGACAAACGGGGAAAAAAGAAAAATCGGGGGTTGATTGGTCTATCCTAACGATTAAGGGCCGCAGGCCCGTCAAGATGGATGAACGGTTAGATTTGATTGCTTAGCGACGAAGA
AAGGAATCGTTTTGGGACCACAGACAAGACAGCTGTCACTACTTTTAAAAAATAATATAATAACCAATTGACGATAGTACCCCTCCCAAAGATGACGTATAGGGGTGTCCCGAGGTAATT
TAACATAGCTCTGACAAGAGATAGTGGACGTTGGATGCCACGATCGGACGATCGTGGTTTGATGAACCAATGATGACGTAGGGGCTGGGGCTTAT

>U3_alyu29
TATTACCCCCAGCCCCCAGGACGGGACATGGGCTTTTTAAATGGGCCGAGAGAGTTTGAACAGTTCAGTATCTTCGTTATTGGGCCATCTGGCC**CAATAATTAA**GAGAACGTGTTCAAAA
TCTGGGTGTGACCGAAGGTCAAGGTAGACGGTCAACAATATTCTGGCTTGCGGAGCAAGCAACACGAATTAAATATCTAATTCGTAAGACACGTGGACGGACCGAAATACTCCTGTATCT
CTATAAATAGCTTAAATCTGGCTTGGATAATTACTCTCGCTCTTCTGTCAAAGCTGTTGTGTTGAGGCGGAAGATCGCCATCGGCGATCATCAGACGAAAAGCTGCAAGAGAGACGGAGA
ACCATGCTGCGAAGCGTATATCGGGTATTTATAGACTTCTAGCGCAGCTAGAAGTTTCCTTGTACTTGATATTGTATTTTGTAAATcACGAAGAAATTCGTACATTGATAAT**AATAAA**AC
ATCTGGGATTGTTAATGTTTACATTAACCAGTATTAAAAATGTACAATAACAAATATTGTATGAGGAACGAATACAATAATATTAAAATATGAGGAGCGTAGCGTGATAAACAGGTGTTT
AAGGTATAATTAAATTAATTATGCAATGTCATGATAATACGGTATAAGTTGAAGTATGAGGTGAAAGAGGAGATATTAGAATATTAAAAACCCAATTATATTATTTATAATACATACAAC
ACGCTATGACAAAAGGGGAAAAAAGAAAAATCGGGGGTTGATTGGTCTATCCTAACGATTAAGGGCCGCAGGCCCGTCAAGATGGATGAACGGTTAGATTTGATTGCTTAGCCACGAAGA
AAGGAATCTTTTGTGGGACCACAGACAAGACAGCTGTCACTACTTTTTAAAATAATATAATAACCAATGGACGATACTACCCCTCCCAAAGATGACGTATAGGGGTGTCCCGAGGTAATT
TAACATAGCTCTGACAAGAGATAGTGGACGTTGGATGCCACGATCGGACGATCGTGGTTTGATGAACTAATGATGACGTAGGGGCTGGGGCTTAT

>U3_alyu32
TATTACCCCCAGCCCCCAGGACGGGACATGGGCTTTTTAAATGGGCCGAGAGAGTTTGAACAGTTCAGTATCTTCGTTATTGGGCCATCTGGCC**CAATAATTAA**GAGAACGTGTTCAAAA
TCTGGGTTTGACCGAAGGTCAAGGTAGACGGTCAACAATATTCTGGCTTGCGGAGCAAGCAACACGAATTAAATATTTAATTCGTATGACACGTGGACGGACCGAAATACTCCTGCATCT
CTATAAATAGCTTAAATCTGGCTTGGATAATTGCTCTCGCTCTTCTGTCAAAGATGTTGTGTTGAGGCGGAAGATCGCCATCGGCGATCATCAGACGAAAAGCTGCAAGAGAGACGGAGA
ACCATGCTGCGAAGCGTATATCGGGTATTTATAGACTTCTAGCGCAGCTAGAAGTTTCCTTGTACTTGATTTTGTATTTTGTAAATTACGAAGAAATTCGTATATTGATAAT**AATAAA**AC
ATCTGGGATTGTTAATGTTTACATTAACCAGTATTAATAATGTACAATAACAAATATTGTATGAGGAACGAATACAATAATATTAAAATATGAGGAGCGTAGCGTGATAAACAGGTGTTT
AAGGTATAATTAAATTAATTATGCAATGTCATGATAATACGGTATAAGTTGAAGTATGAGGTGAAAGAGGAGATATTAGAATATTAAAAACCCAATTATATTATTTATAATACATACAAC
ACGCTATGACAAAAGGGGAAAAAAGAAAAATCGGGGGTTGATTGGTCTATCGTATCGCTTAAGGGCCGCAGGCCCGTCAAGATGGATGAACGGTTAGATTTGATTGCTTAGCCACGAAGA
AAGGAATCTTTTTGGGACCACAGATAAGACAGCTGTCATTACTTTTTAAAATAATATAATAACCAAAGGACGATAGTACCCCTCCCAAAGATGACGTATAGGGGTGTCCCGAGGTAATTT
AACATAGCTCTGACAAGAGATAGTGGACGTTGGATGCCACGATCGGACGATCGTGGTTTGATGAACCAATGATGACGTAGGGGCTGGGGCTTAT

>U3_alyu33
TATTACCCCCAGCCCCCAGGACGGGACATGGGCTTTTTAAATGGGCCGAGAGAGTTTGAACAGTTCAGTATCTTCGTTATTGGGCCATCTGGCC**CAATAATTAA**GAGAACGTGTTCAAAA
TCGGGGTTTGACCGAAGGTCAAGGTAGACGGTCAACAATATTCTGGCTTGCGGAGCAAGCAACACGAATTAAATATTTAATTCGTAGACACGTGGACGGACCGAAATACTCCTGTATCT**C**
TATAAATAGCTTAAATCTGGCTTGGATATTTGCTCTCGCTCTTCTGTCAAAGCTGTTGTGTTGAGGCGGAAGATCGCCATCGGCGATCATCAGACGAAAAGCTGCAAGAGAGACGGAGAA
CCATGCTGCGAAGCGTATATCCGGTATTTATAGACTTCTAGCGCAGCTAGAAGTTTCCCTGTACTTGATATTGTATTTTGTAAATTACGAAGAAATTCGTACATTGATAAT**AATAAA**ACA
TCTGGGTTTGTTAATGTTTACATTAACCAGTATCAATAATGTACAATAACAAATATTGTATGAGGAACGAATACAATAATATTAAAATATGAGGAGCGTAGCGTGATAAACAGGTGTTTA
AGGTATAATTAAATTAATTATGCAATGTCATGATAATACGGTATAAGTTGAAGTATGAGGTGAAAGAGGAGATATTAGAATATTAAAAAACCCAATTATATTATTTATAATACATACAAC
ACGCTATGACAAAAGGGGAAAAATGAAGAATCGGGGGTTGATTGGTCTATCCTAACGATTAAGGGCCGCAGGCCCGTCAAGATGGATGAACGGTTAGATTTGATTGCTTAGCCAAGAAGA
AAGGAATCTTTTTGGGACCACAGATAAGACAGCTGTTACTACCTTTAAAAATAATATAATATCCAATTGACGATAGTACCCCTCCCAAAGATGACGTATAGGGGTGTCCCGAGGTAATTT
AACATAGCTCTGACAAGAGATAGTGGACGTTGGATGCCACGATCGGACGATCGTGGTTTGATGAACTAATGATGACGTAGGGGCTGGGGCTTAT

>U3_alyu34_v1
TATTACCCCCAGCCCCCAGGACGGGACATGGGCTTTTTAAATGGGCCGAGAGAGTTTGAACAGTTCAGTATCTTCGTTATTGGGCCATCTGGCC**CAATAATTAA**GAGAACGTGTTCAAAA
TCGGGGTTTGACCGAAGGTCAAGGTAGACGGTCAACAATATTCTGGCTTGCGGAGCAAGCAACAcGAATTAAATATTTAATTCgTATGACACGTGGACGGACCGAAATACTCCTGTATCT
CTATAAATAGCcTAAATCTGGCTTGGATAATTGCTCTCGCTCTTCTGTCAAAGcTGTTGTGTTGAGGCGGAAGATCGCCATCGGCGATCATCGGACGAAAAGCTGCAAGAGAGACGGAGA
ACCATGCTaCGAAGCGTATATCGGGTATTTATAGACTTCTAGCGCAGCTAGAAGTTTCCTTGTACTTGtTATTGTATTTTGTAAATTACGAAGAAATTCGTATATTGATAAT**AATAAA**AC
ATCTGGGATTGTTAATGTTTACATTAACcAGTATTAATAATGTACAATAACAAATATTGTATGAGGAACGAATACAAGAATATTAAAATATGAGGAGCGTAGCGTGATAAACAGGTGTTT
AAGGTATAATTAAAaTAATTATGCAAaGTaATGATAATACGGTATAAGTTGAAGTATGAGGTGAAAGAGGAGATATTAGAATATTAAAAACCCAATTATATTATTTATAATACATACAAC
ACGCTATGACAAACGGGGAAAAAAGAAAAATCGGGGGTTGATTGGTCTATCCTAACGATTAAGGGCCGCAGGCCCGTCAAGATGGATGAACGGTTAGATTTGATTGCTTAGCCACGAAGA
AAGGAATCGTTTTGGGACCACAGACAAGACAGCTGTCACTACTTTTAAAAAATAATATAATAACCAATTGACGATAGTACCCCTCCCAAAGATGACGTATAGGGGTGTCCCGAGGTAATT
TAACATAGCTCTGACAAGAGATAGTGGACGTTGGATGCCACGATCGGACGATCGTGGTTTGATGAACcAATGATGACGTAGGGGCTGGGGCTTAT

>U3_alyu34_v2
TATTACCCCCAGCCCCCAGGACGGGACATGGGCTTTTTAAATGGGCCGAGAGAGTTTGAACAGTTCAGTATCTTCGTTATTGGGCCATCTGGCC**CAATAATTAA**GAGAACGTGTTCAAAA
TCGGGGTTTGACCGAAGGTCAAGGTAGACGGTCAACAATATTCTGGCTTGCGGAGCAAGCAACAaGAATTAAATATTTAATTCtTATGACACGTGGACGGACCGAAATACTCCTGTATCT
CTATAAATAGCtTAAATCTGGCTTGGATAATTGCTCTCGCTCTTCTGTCAAAGgTGTTGTGTTGAGGCGGAAGATCGCCATCGGCGATCATCGGACGAAAAGCTGCAAGAGAGACGGAGA
ACCATGCTgCGAAGCGTATATCGGGTATTTATAGACTTCTAGCGCAGCTAGAAGTTTCCTTGTACTTGaTATTGTATTTTGTAAATTACGAAGAAATTCGTATATTGATAAT**AATAAA**AC
ATCTGGGATTGTTAATGTTTACATTAACtAGTATTAATAATGTACAATAACAAATATTGTATGAGGAACGAATACAAGAATATTAAAAATGAGGAGCGTAGCGTGATAAACAGGTGTTTA
AGGTATAATTAAAtTAATTATGCAAtGTcATGATAATACGGTATAAGTTGAAGTATGAGGTGAAAGAGGAGATATTAGAATATTAAAAACCCAATTATATTATTTATAATACATACAACA
CGCTATGACAAACGGGGAAAAAAGAAAAATCGGGGGTTGATTGGTCTATCCTAACGATTAAGGGCCGCAGGCCCGTCAAGATGGATGAACGGTTAGATTTGATTGCTTAGCCACGAAGAA
AGGtATCGTTTTGGGACCACAGACAAGACAGCTGTCACTACTTTTAAAAAATAATATAATAACCAAaTGACGATAGTACCCCTCCCAAAGATGACGTATAGGGGTGTCCCGAGGTAATTT
AACATAGCTCTGACAAGAGATAGTGGACGTTGGATcCCACGATCGGACGATCGTGGTTTGATGAACtAATGATGACGTAGGGGCTGGGGCTTAT

>U3_alyu35
TATTACCCCCAGCCCCCAGGACGGGACATGGGCTTTTTAAATGGGCCGAGAGAGTTTGAACAGTTCAGTATCTTCGTTATTGGGCCATCTGGCC**CAATAATTAA**GAGAACGTGTTCAAAA
TCGGGGTTTGACCGAAGGTCAAGGTAGACGGTCAACAATATTCTGGCTTGCGGAGCAAGCAACACGAATTAAATATTTAATTCGTATGACACGTGGACGGACCGAAATACTCCTGTATCT
CTATAAATAGCTTAAATCTGGCTTGGATAATTGCTCTCGCTCTTCTGTCAAAGCTGTTGTGTTGAGGCGGAAGATCGCCATCGGCGATCATCGGACGAAAAGCTGCAAGAGAGACGGAGA
ACCATGCCACGAAGCGTATATCGGGTATTTATAGACTTCTAGCGCAGCTAGAAGTTTCCTTGTACTTGATATTGTATTTTGTAAATTACGAAGAAATTCGTATATTGATAAT**AATAAA**AC
ATCTGGGATTGTTAATGTTTACATTAACCAGTATTAATAATGTACAATAACAAATATTGTATGAGGAACGAATACAAAAATATTAAAATATGAGGAGCGTAGCATGATAAACAGGTGTTT
AAGGTATAATTAAATTAATTATGCAATGTCATGATAATACGGTATAAGTTGAAGTATGAGGTGAAAGAGGAGATATAAGAATATTAAAAACCCAATTATATTATTTATAGGACATACAAC
ACGCTATGACAAAAGGGGAAAAAAGAAAAATCGGGGGTTGATTGGTCTATCCTAACGATTAAGGGCCGCAGGCCCGTCAAGATGGATGAACGGTTAGATTTGATTGCTTAGCTACGAAGA
AAGGAATCCTTTTGGGACCACAGACAAGACAGCTGTCACTACTTTTAAAAAATAATATAATAACCAAATGACGATAGTACCCCTCCCAAAGATGACGTATAGGGGTGTCCCGAGGTAATT
TAACATAGCTCTGACAAGAGATAGTGGACGTTGGATGCCACGATCGGACGATCGTGGTTTGATGAACTAATGATGACGTAGGGGCTGGGGCTTAT

>U3_alyu36
TATTACCCCCAGCCCCCAGGACGGGACATGGGCTTTTTAAATGGGCCGAGAGAGTTTGAACAGTTCAGTATCTTCGTTATTGGGCCATCTGGCC**CAATAATTAA**GAGAACGTGTTCAAAA
TCGGGGTTTGACCGAAGGTCAAGGTAGACGGTCAACAATATTCTGGCTTGCGGAGCAAGCAACACGAATTAAATATTTAATTCGTAAGACACGTGGACGGACCGAAATACTCCTGAATCT
CTATAAATAGCTTAAATCTGGCTTGGATAATTGCTCTCGCTCTTCTGTCAAAGCTGTTGTGTTGAGGCGGAAGATCGCCATCGGCGATCATCGGACGAAAAGCTGCAAGAGAGACGGAGA
ACCATGCTACGAAGCGTATATCGGGTATTTATAGACTTCTAGCGCAGCTAGAAGTTTCCTTGTACTTGATATTGTATTTTGTAAATTACGAAGAAATTCGTATATTGATAAT**AATAAA**AC
ATCTGGGACTGTTAATGTTTACATTAACCAGTATTAATAATGTATAATAACAAATATTGTATGAGGAACGAATACAAGAATATTAAAATATGAGGAGCGTAGCGTGATAAACAGGTGTTT
AAGGTATAATTAAATTAATTATGCAATGTCATGATAATACGGTATAAGTTGAAGTATGAGGTGAAAGAGGAGATATTAGAATATTAAAAACCCAATTATATTATTTATAATACATACAAC
ACGCTATGACAAACGGGGAAAAAAGAAAAATCGGGGGTTGATTGGTCTATCCTAACGATTAAGGGCCGCAGGCCCGTCAAGATGGATGAACGGTTAGATTTGATTGCTTAGCCACGAAGA
AAGGAATCGTTTTGGGACCACTGACAAGACAGCTGTCACTACTTTTAAAAAATAATATAATAACCAATTGACGATAGTACCCCTCCCAAAGATGACGTATAGGGGTGTCCCGAGGTAATT
TAACATAGCTCTGACAAGAGATAGTGGACGTTGGATGCCACGATCGGACGATCGTGGTTTGATGAACTAATGATGACGTAGGGGCTGGGGCTTAT

>U3_alyu37
TATTACCCCCAGCCCCCAGGACGGGACATGGGCTTTTTAAATGGGCCGAGAGAGTTTGAACAGTTCAGTATCTTCGTTATTGGGCCATCTGGCC**CAATAATTAA**GAGAACGTGTTCAAAA
TCGGGGTTTGACCGAAGGTCAAGGTAGACGGTCAACAATATTCTGGCTTGCGAAGCAAGCAACACGAATTAAATATTTAATTCGTATGACACGTGGACGGACCGAAATACTCCTGTATCT
CTATAAATAGCTTAAATCTGGCTTGGATAATTGCTCTCGCTCTTCTGTCAAAGCTGTTGTGTTGAGGCGGAAGATCGCCATCGGCGATCATCGGACGAAAAGCTGCAAGAGAGACGGAGA
ACCATGCTACGAAGCGTATATCGGGTATTTATAGACTTCTAGCGCAGCTTGAAGTTTCCTTGTACTTGATATTGTATTTTGTAAATTACGAAGAAATTCGTATATTGATAAT**AATAAA**AC
ATCTGGGATTGTTAATGTTTACATTAACCAGTATTAATAATGTACAATAACAAATATTGTATGAGGAACGAATACAAGAATATTAAAATATGAGGAGCGTAGCGTGATAAACAGGTGTTT
AAGGTATAATTAAATTAATTATGCAATGTCATGATAATACGGTATAAGTTGAAGTATGAGGTGAAAGAGGAGATATTAGAATATTAAAAACCCAATTATATTATTTATAATACATACAAC
ACGCTATGACAAACGGGGAAAAAAGAAAAATCGGGGGTTGATTGGTCTATCCTAACGATTAAGGGCCGCAGGCCCGTCAAGATGGATGAACGGTTAGATTTGATTGCTTAGCCACGAAGA
AAGGAATCGTTTTGGGACCACAGACAAGACAGCTGTCATTACTTTTAAAAAATAATATAATAACCAATTGACGATAGTACCCCTCCCAAAGATGACGTATAGGGGTGTCCCGAGGTAATT
TAACATAGCTCTGACAAGAGATAGTGGACGTTGGATGGCACGATCGGACGATCGTGGTTTGATGAACCAATGATGACGTAGGGGCTGGGGCTTAT

>U3_alyu39
TATTACCCCCAGCCCCCAGGACGGGACATGGGCTTTTTAAATGGGCCGAGAGAGTTTGAACAGTTCAGTATCTTCGTTATTGGGCCATCTGGCC**CAATAATTAA**GAGAACGTGTTCAAAA
TCGGGGTTTGACCGAAGGTCAAGGTAGACGGTCAACAATATTCTGGCTTGCGGAGCAAGCAACACGAATTAAATATTTAATTCGTATGACACGTGGACGGACCGAAATACTCCTGTAACT
cTATAAATAGCTTAAATCTGGCTTGGATAATTGCTCTCGCTCTTCTGTCAAAGCTGTTGTGTTGAGGCGGAAGATCGCCATCGGCGATCATCGGACGAAAAGCTGCAAGAGAGACGGAGA
ACCATGCTACGAAGCGTATATCGGGTATTTATAGACTTCTAGCGCAGCTAGAAGTTTCCTTGTACTTGATATTGTATTTTGTAAATTACGAAGAAATTCGTATATTGATAAT**AATAAA**AC
ATCTGGGATTGTTAATGTTTACATTAACTAGTATTAATAATGTACAATAACAAATATTGTATGAGGAACGAATACAAGAATAATAAAATATGAGGAGCGTAGCGTGATAAACAGTTGTTT
AAGGTATAATTAAATTAATTATGCAATGTCCTGATAATACGGTATAAGTTGAAGTATGAGGTGAAAGAGGAGATATTAGAATATTAAAAACCCAATTATATTATTTATAATACATACAAC
ACGCTATGACAAACGGGGAAAAAAGAAAAATCGGGGGTTGATTGGTCTATCCTAACGATTAAGGGCCGCAGGCCCGTCAAGATGGATGAACGGTTAGATTTGATTGCTTAGCCACGAAGA
AAGGAATCGTTTTGGGACCACAGACAAGACAGCTGTCACTACTTTTAAAAAATAATATAATAACCAATTGACGATAGTACCCCTCCCAAAGATGACGTATAGGGGTGTCCCGAGGTAATT
TAACATAGCTCTGACAAGAGATAGTGGACGTTGGATGCCACGATCGGACGATCGTGGTTTGATGAACCAATGATGACGTAGGGGCTGGGGCTTAT

>U3_alyu40
TATTACCCCCAGCCCCCAGGACGGGACATGGGCTTTTTAAATGGGCCGAGAGAGTTTGAACAGTTCAGTATCTTCGTTATTGGGCCATCTGGCC**CAATAATTAA**GAGAACGTGTTCAAAA
TCGGGAGTTTGACCGAAGGTCAAGGTAGACGGTCAACAATATTCTGGCTTGCGGAGCAAGCAACACGAATTAAATATTTAATTCGTATGACACGTGGACGGACCGAAATACTCCTGTACC
T**CTATAAATA**GCTTAAAACTGGCTTGGATAATTGCTCTCGCTCTTCTGTCAAAGCTGTTGTGTTGAGGCGGAAGATCGCCATCGGCGATCATCAGACGAAAAGCTGCAAGAGAGACGGAG
AACCATGCTGCGAAGCGTATATCGGGTATTTATAGACTTCTAGCGCAGCTAGAAGTTTCCTTGTACTTGATATTGTATTTTGTAAATTACGAAGAAATTCGTAGATTGATAAT**AATAAA**A
CATCTGGGTTTGTTAATGTTTACATTAACCAGTATTAATAATGTACAATAACAAAAATTGTATGAGGAACGAATACAATAATATTAAAATATGAGGAGCGTAGCGTGATAAACAGGTGTT
TAAGGTATAATTAAATTAATTATGCAAAGTCATGATAATACGGTATAAGTTGAAGTATGAGGTGAAAGAGGAGATATTAGAATATTAAAAACCCAATTATATTATTTATAATACATACAA
CACGCTATGACAAAAGGGGAAAAAAGAAAAATCGGGGGTTGATTGGTCTATCCTAACGATTAAGGGCCGCAGGCCCGTCAAGATGGATGAACGGTCAGATTTTATTGCTTAGCCACGAAG
AAAGGAATCTTTTTGGGACCACAGATAAGACAGCTGTCACTACTTTTTAAAATAATATAATAACCAATTGACGATAGTACCCCTCCCAAAGATGACGTATAGGGGTGTCCCGAGGTAATT
TAACATAGCTCTGACAAGAGATAGTGGACGTTGGATGCCACGATCGGACGATCGTGGTTTGATGAACTAATGATGACGTAGGGGCTGGGGCTTAT

>U3_alyu41
TATTACCCCCAGCCCCCAGGACGGGACATGGGCTTTTTAAATGGGCCGAGAGAGTTTGAACAGTTCAGTATCTTCGTTATTGGGCCATCTGGCC**CAATAATTAA**GAGAACGTGTTCAAAA
TCtGGGTTTGACCGAAGGTCAAGGTAGACGGTCAACAATATTCTGGCTTGCGGAGCAAGCAACAAGAATTAAATATTTAATTCGTATGACACGTGGACGGACCGAAATACTCCTGTATCT
CTATAAATAGCCTAAATCTGGCATGGATAATTGCTCTCGCTCTTCTGTCAAAGCTGTTGTGTTGAGGCGGAAGATCGCCATCGGCGATCATCAGACGAAAAGCTGCAAGAGAGACGGAGA
ACCATGCTGCGAAGCGTATATCGGGTATTTATAGACTTCTAGCGCAGCTAGAAGTTTCCTTGTACTTGTATTTGTATTTTGTAAATTACGAAGAAATTCGTACATTGATAAT**AATAAA**AC
ATCTGGGATTGTTAATGTTTACATTAACTAGTATTAATAATGTACAATAACAAACATTGTATGAGGAACGAATACAAGAATATTAAAATATGAGGAGCGTAGCGTGATAAACAGGTGTTT
AAGGAATAATTAAAATAATTATGCAATGTTATGATAATACGGTATAAGTTGAAGTATGAGGTGAAAGAGGAGATATTAGAATATTAAAAACCCAATTATATTATTTATAATACATACAAC
ACGCTATGACAAAAGGGGAAAAAAGAAAAATCGGGGGTTGATTGGTCTATCCTAACGATTAAGGGCCGCAGGCCCGTCAAGATGGATCCAATAACCCGTTAAGAAGGTAAACGGGTCTAA
AACGATTTCTTCGCCCGCAAGCAACACCTTTAACCTCTGCGCACCTATATATATAATATAATAACCAAATGACCATAGTGCCCCTCCCAAAGATGACGTATAGGGGTGTCCCGAGGTAAT
TTAACATAGCTCTGATAAGAGATAGTGGACGTTGGATTCCACGATCGGACGATCGTGGTTTGATGAACTTATGATGACGTAGGGGCTGGGGCTTAT

>U3_alyu42_43
TATTACCCCCAGCCCCCAGGACGGGACATGGGCTTTTTAAATGGGCCGAGAGAGTTTGAACAGTTCAGTATCTTCGTTATTGGGCCATCTGGCC**CAATAATTAA**GAGAACGTGTTCAAAA
TCGGGGTTTGACCGAAGGTCAAGGTAGACGGTCAACAATATTCTGGCTTGCGGAGCAAGCAACACGAATTAAATATTTAATTCGTATGACACGTGGACGGACCGAAATACTCCTGTAAAT
CTATAAATAGCCTAAATCTGGCCTGGATAATTGCTCTCGCTCTTCTGTCAAAGCTGTTGTGTTGAGGCGGAAGATCGCCATCGGCGATCATCAGACGAAAAGCTGCAAGAGAGACGGAGA
AACATGCTGCGAAGCGTATATCGGGTATTTATAGACTTCTAGCGCAGCTAGAAGTTTCGTTGTATTTGATATTGTATTTTGTAAATTACGAAGAATTCGTACATTGATAAT**AATAAA**ACA
TCTGGGATTGTTAATGTTTACATTAACCAGTATTAATAATGTACAATAAAAAAAATTGTATGAGGATCGAATACAATAATATTAAAATATGAGGAGCGTAGCATGATAAACAGGTGTTTA
AGGTATAATTAAAAAAATTATGCAATGTTATGATAATACGGTATAAGTTGAAGTATGAGGTGAAAGAGGAGATATTAGAATATTAAAAACCCAATTATATTATTTATAATACATACAACA
CGCTATGACAAAAGGGGAAAAATGAAGAATCGGGGGTTGATTGGTCTATCGTATCGCTTAAGGGCCGCAGGCCCGTCAAGATGGATGAACGGTCAGATTTGATTGCTTAGCCACGAAGAA
AGGAATCTTTTTGGGACCACAGATAAGACAGCTGTCATTACCTTTTAAAATAATATAATAACCAATTGACGATAGTACCCCTCCCAAAGATGACGTATAGGGGTGTCCCGAGGTAATTTA
ACATAGCTCTGACAAGAGATAGTGGACGTTGGATGCCACGATCGGACGATCGTGGTTTGATGAACTTATGATGACGTAGGGGCTGGGGCTTAT

>U3_alyu43_v2
TATTACCCCCAGCCCCCAGGACGGGACATGGGCTTTTTAAATGGGCCGAGAGAGTTTGAACAGTTCAGTATCTTCGTTATTGGGCCATCTGGCC**CAATAATTAA**GAGAACGTGTTCAAAA
TCGGGGTTTGACCGAAGGTCAAGGTAGACGGTCAACAATATTCTGGCTTGCGGAGCAAGCAACACGAATTAAATATTTAATTCGTATGACACGTGGACGGACCGAAATACTCCTGTAAAT
CTATAAATAGCTTAAATCTGGCTTGGATAATTGCTCTCGCTCTTCTGTCAAAGGTGTTGTGTTGAGGCGGAAGATCGCCATCGGCGATCATCAGACGAAAAGCTGCAAGAGAGACGGAGA
AACATGCTGCGAAGCGTATATCGGGTATTtATAGACTTCTAGCGCAGCTAGAAGTTTCtTTGTAcTTGATATTGTATTTTGTAAATTACGAAGAAaTCGTACATTGATAAT**AATAAA**ACA
TCTGGGATTGTTAATGTTTACATTAACCAGTATTAATAATGTACAATAAAAAAtATTGTATGAGGAaCGAATACAATAATATTAAAATATGAGGAGCGTAGCATGATAAACAGGTGTTTA
AGGTATAATTAAAAAAATTATGCAATGTTATGATAATACGGTATAAGTTGAAGTATGAGGTGAAAGAGGAGATATTAGAATATTAAAAACCCAATTATATTATTTATAATACATACAACA
CGCTATGACAAAAGGGGAAAAATGAAGAATCGGGGGTTGATTGGTCTATCGTATCGCTTAAGGGCCGCAGGCCCGTCAAGATGGATGAACGGTCAGATTTGATTGCTTAGCCACGAAGAA
AGGAATCTTTTTGGGACCACAGATAAGACAGCTGTCATTACCTTTTAAAATAATATAATAACCAATTGACGATAGTACCCCTCCCAAAGATGACGTATAGGGGTGTCCCGAGGTAATTTA
ACATAGCTCTGACAAGAGATAGTGGACGTTGGATGCCACGATCGGACGATCGTGGTTTGATGAACTTATGATGACGTAGGGGCTGGGGCTTAT

>U3_alyu44_45
TATTACCCCCAGCCCCCAGGACGGGACATGGGCTTTTTAAATGGGCCGAGAGAGTTTGAACAGTTCAGTATCTTCGTTATTGGGCCATCTGGCC**CAATAATTAA**GAGAACGTGTTCAAAA
TCGGGGTTTGACCGAAGGTCAAGGTAGACGGTCAACAATATTCTGGCTTGCGGAGCAAGCAACACGAATTAAATATTTAATTCGTATGACACGTGGACGGACCGAAATACTCCTGTAACT
CTATAAATAGCTTAAATCTGGCTTGGATAATTGCTCTCGCTCTTCTGTCAAAGGTGTTGTGTTGAGGCGGAAGATCGCCATCGGCGATCATCAGACGAAAAGCTGCAAGAGAGACGGAGA
AACATGCTGCGAAGCGTATATCGGGTATTTATAGACTTCTAGCGCAGCTAGAAGTTTCGTTGTACTTGATATTGTATTTTGTAAATTACGAAGAAATCGTACATTGATAAT**AATAAA**ACA
TCTGGGATTGTTAATGTTTACATTAACCAGTATTAATAATGTACAATAAAAAATATTGTATGAGGAACGAATACAATAATATTAAAATATGAGGAGCGTAGCATGATAAACAGGTGTTTA

AGGTATAATTAAAAAAATTATGTAATGTTATGATAATACGGTATAAGTTGAAGTATGAGGTGAAAGAGGAGATATTAGAATATTAAAAACCCAATTATATTATTTATAATACATACAACA
CGCTATGACAAAAGGGGAAAAATGAAGAATCGGGGGTTGATTGGTCTATCGTATCGCTTAAGGGCCGCAGGCCCGTCAAGATGGATGAACGGCCAGATTTGATTGCTTAGCCACGAAGAA
AGGAATCTTTTTGGGACCACAGATAAGACAGCTGTCATTACCTTTTAAAATAATATAATAACCAATTGACGATAGTACCCCTCCCAAAGATGACGTATAGGGGTGTCCCGAGGTAATTTA
ACATAGCTCTGACAAGAGATAGTGGACGTTGGATGCCACGATCGGACGATCGTGGTTTGATGAACCTATGATGACGTAGGGGCTGGGGCTTAT

>U3_alyu47
TATTACCCCCAGCCCCCAGGACGGGACATGGGCTTTTTAAATGGGCCGAGAGAGTTTGAACAGTTCAGTATCTTCGTTATTGGGCCATCTGGCC**CAATAATTAA**GAGAACGTGTTCAAAA
TCTGGGTTTGACCGAAGGTCAAGGTAGACGGTCAACAATATTCTGGCTTGCGGAGCAAGCAACAAGAATTAAATATTTAATTCGTATGACACGTGGACGGACCGAAATACTCCTGTATCT
CTATAAATAGCCTAAATCTGGCATGGATAATTGCTCTCGCTCTTCTGTCAAAGCTGTTGTGTTGAGGCGGAAGATCGCCATCGGCGATCATCAGACGAAAAGCTGCAAGAGAGACGGAGA
ACCATGCTGCGAAGCGTATATCGGGTATTTATAGACTTCTAGCGCAGCTAGAAGTTTCCTTGTACTTGTATTTGTATTTTGTAAATTACGAAGAAATTCGTACATTGATAAT**AATAAA**AC
ATCTGGGATTGTTAATGTTTACATTAACTAGTATTAATAATGTACAATAACAAACATTGTATGAGGAACGAATACAAGAATATTAAAATATGAGGAGCGTAGCGTGATAAACAGGTGTTT
AAGGAATAATTAAAATAATTATGCAATGTTATGATAATACGGTATAAGTTGAAGTATGAGGTGAAAGAGGAGATATTAGAATATTAAAAACCCAATTATATTATTTATAATACATACAAC
ACGCTATGACAAAAGGGGAAAAAAGAAAAATCGGGGGTTGATTGGTCTATCCTAACGATTAAGGGCCGCAGGCCCGTCAAGATGGATGAACGGTTAGATTTGATTGCTTAGACACGAAGA
AAGGAATCTTTTTGGGACCACAGATAAGACAGCTGTCACTACTTTTAAAAAATAATATAATAACCAAATGACCATAGTGCCCCTCCCAAAGATGACGTATAGGGGTGTCCCGAGGTAATT
TAACATAGCTCTGACAAGAGATAGTGGACGTTGGATTCCACGATCGGACGATCGTGGTTTGATGAACTTATGATGACGTAGGGGCTGGGGCTTAT

>U3_alyu48_v1
TATTACCCCCAGCCCCCAGGACGGGACATGGGCTTTTTAAATGGGCCGAGAGAGTTTGAACAGTTCAGTATCTTCGTTATTGGGCCATCTGGCC**CAATAATTAA**GAGAACGTGTTCAAAA
TCTGGGTTTGACCGAAGGTCAAGGTAGACGGTCAACAATATTCTGGCTTGCGGAGCAAGCAACAAGAATTAAATATTTAATTCGTATGACACGTGGACGGACCGAAATACTCCTGTATCT
CTATAAATAGCCTAAATCTGGCATGGATAATTGCTCTCGCTCTTCTGTCAAAGCTGTTGTGTTGAGGCGGAAGATCGCCATCGGCGATCATCAGACGAAAAGCTGCAAGAGAGACGGAGA
ACCATGCTGCGAAGCGTATATCGGGTATTTATAGACTTCTAGCGCAGCTAGAAGTTTCCTTGTACTTGTATTTGTATTTTGTAAATTACGAAGAAATTCGTACATTGATAAT**AATAAA**AC
ATCTGGGATTGTTAATGTTTACATTAACTAGTATTAATAATGTACAATAACAAACATTGTATGAGGAACGAATACAAGAATATTAAAATATGAGGAGCGTAGCGTGATAAACAGGTGTTT
AAGGAATAATTAAAATAATTATGCAATGTTATGATAATACGGTATAAGTTGAAGTATGAGGTGAAAGAGGAGATATTAGAATATTAAAAACCCAATTATATTATTTATAATACATACAAC
ACGCTATGACAAAAGGGGAAAAAAGAAAAATCGGGGGTTGATTGGTCTATCCTAACGATTAAGGGCCGCAGGCCCGTCAAGATGGATGAACGGTTAGATTTGATTGCTTAGACACGAAGA
AAGGAATCTTTTTAGGACCACAGATAAGACAGCTGTCACTACTTTTAAAAAATAATATAATAACCAAATGACCATAGTGCCCCTCCCAAAGATGACGTATAGGGGTGTCCCGAGGTAATT
TAACATAGCTCTGACAAGAGATAGTGGACGTTGGATTCCACGATCGGACGATCGTGGTTTGATGAACTTATGaTGACGTAGGGGCTGGGGCTTAT

>U3_alyu48_v2
TATTACCCCCAGCCCCCAGGACGGGACATGGGCTTTTTAAATGGGCCGAGAGAGTTTGAACAGTTCAGTATCTTCGTTATTGGGCCATCTGGCC**CAATAATTAA**GAGAACGTGTTCAAAA
TCTGGGTTTGACCGAAGGTCAAGGTAGACGGTCAACAATATTCTGGCTTGCGGAGCAAGCAACAAGAATTAAATATTTAATTCGTATGACACGTGGACGGACCGAAATACTCCTGTATCT
CTATAAATAGCCTAAATCTGGCATGGATAATTGCTCTCGCTCTTCTGTCAAAGCTGTTGTGTTGAGGCGGAAGATCGCCATCGGCGATCATCAGACGAAAAGCTGCAAGAGAGACGGAGA
ACCATGCTGCGAAGCGTATATCGGGTATTTATAGACTTCTAGCGCAGCTAGAAGTTTCCTTGTACTTGTATTTGTATTTTGTAAATTACGAAGAAATTCGTACATTGATAAT**AATAAA**AC
ATCTGGGATTGTTAATGTTTACATTAACTAGTATTAATAATGTACAATAACAAACATTGTATGAGGAACGAATACAAGAATATTAAAATATGAGGAGCGTAGCGTGATAAACAGGTGTTT
AAGGAATAATTAAAATAATTATGCAATGTTATGATAATACGGTATAAGTTGAAGTATGAGGTGAAAGAGGAGATATTAGAATATTAAAAACCCAATTATATTATTTATAATACATACAAC
ACGCTATGACAAAAGGGGAAAAAAGAAAAATCGGGGGTTGATTGGTCTATCCTAACGATTAAGGGCCGCAGGCCCGTCAAGATGGATGAACGGTTAGATTTGATTGCTTAGACACGAAGA
AAGGAATCTTTTTAGGACCACAGATAAGACAGCTGTCACTACTTTTAAAAAATAATATAATAACCAAATGACCATAGTGCCCCTCCCAAAGATGACGTATAGGGGTGTCCCGAGGTAATT
TAACATAGCTCTGACAAGAGATAGTGGACGTTGGATTCCACGATCGGACGATCGTGGTTTGATGAACTTATGcTGACGTAGGGGCTGGGGCTTAT

>U3_alyu49
TATTACCCCCAGCCCCCAGGACGGGACATGGGCTTTTTAAATGGGCCGAGAGAGTTTGAACAGTTCAGTATCTTCGTTATTGGGCCATCTGGCC**CAATAATTAA**GAGAACGTGTTCAAAA
TCTGGGTTTGACCGAAGGTCAAGGTAGACGGTCAACAATATTCTGGCTTGCGGAGCAAGCAACAAGAATTAAATATTTAATTCGTATGACACGTGGACGGACCGAAATACTCCTGTATCT
CTATAAATAGCCTAAATCTGGCATGGATAATTGCTCTCGCTCTTCTGTCAAAGCTGTTGTGTTGAGGCGGAAGATCGCCATCGGCGATCATCAGACGAAAAGCTGCAAGAGAGACGGAGA
ACCATGCTGCGAAGCGTATATCGGGTATTTATAGACTTCTAGCGCAGCTAGAAGTTTCCTTGTACTTGTATTTGTATTTTGTAAATTACGAAGAAATTCGTACATTGATAAT**AATAAA**AC
ATCTGGGATTGTTAATGTTTACATTAACTAGTATTAATAATGTACAATAACAAACATTGTATGAGGAACGAATACAAGAATATTAAAATATGAGGAGCGTAGCGTGATAAACAGGTGTTT
AAGGAATAATTAAAATAATTATGCAATGTTATGATAATACGGTATAAGTTGAAGTATGAGGTGAAAGAGGAGATATTAGAATATTAAAAACCCAATTATATTATTTATAATACATACAAC
ACGCTATGACAAAAGGGGAAAAAAGAAAAATCGGGGGTTGATTGGTCTATCCTAACGATTAAGGGCCGCAGGCCCGTCAAGATGGATGAACGGTTAGATTTGATTGCTTAGACACGAAGA
AAGGAATCTTTTTGGGACCACAGATAAGACAGCTGTCACTACTTTTAAAAAATAATATAATAACCAAATGACCATAGTGCCCCTCCCAAAGATGACGTATAGGGGTGTCCCGAGGTAATT
TAACATAGCTCTGACAAGAGATAGTGGACGTTGGATTCCACGATCGGACGATCGTGGTTTGATGAACTTATGATGACGTAGGGGCTGGGGCTTAT

>U3_alyu50
TATTACCCCCAGCCCCCAGGACGGGACATGGGCTTTTTAAATGGGCCGAGAGAGTTTGAACAGTTCAGTATCTTCGTTATTGGGCCATCTGGCC**CAATAATTAA**GAGAACGTGTTCAAAA
TCTGGGTTTGACCGAAGGTCAAGGTAGACGGTCAACAATATTCTGGCTTGCGGAGCAAGCAACAAGAATTAAATATTTAATTCGTATGACACGTGGACGGACCGAAATACTCCTGTATCT
CTATAAATAGCCTAAATCTGGCATGGATAATTGCTCTCGCTCTTCTGTCAAAGCTGTTGTGTTGAGGCGGAAGATCGCCATCGGCGATCATCAGACGAAAAGCTGCAAGAGAGACGGAGA
ACCATGCTGCGAAGCGTATATCGGGTATTTATAGACTTCTAGCGCAGCTAGAAGTTTCCTTGTACTTGTATTTGTATTTTGTAAATTACGAAGAAATTCGTACATTGATAAT**AATAAA**AC
ATCTGGGATTGTTAATGTTTACATTAACTAGTATTAATAATGTACAATAACAAACATTGTATGAGGAACGAATACAAGAATATTAAAATATGAGGAGCGTAGCGTGATAAACAGGTGTTT
AAGGAATAATTAAAATAATTATGCAATGTTATGATAATACGGTATAAGTTGAAGTATGAGGTGAAAGAGGAGATATTAGAATATTAAAAACCCAATTATATTATTTATAATACATACAAC
ACGCTATGACAAAAGGGGAAAAAAGAAAAATCGGGGGTTGATTGGTCTATCCTAACGATTAAGGGCCGCAGGCCCGTCAAGATGGATGAACGGTTAGATTTGATTGCTTAGACACGAAGA
AAGGAATCTTTTTGGGACCACAGATAAGACAGCTGTCACTACTTTTAAAAAATAATATAATAACCAAATGACCATATTGCCCCTCCCAAAGATGACGTATAGGGGTGTCCCGAGGTAATT
TAACATAGCTCTGACAAGAGATAGTGGACGTTGGATTCCACGATCGGACGATCGTGGTTTGATGAACTTATGATGACGTAGGGGCTGGGGCTTAT

>U3_alyu51
TATTACCCCCAGCCCCCAGGACGGGACATGGGCTTTTTAAATGGGCCGAGAGAGTTTGAACAGTTCAGTATCTTCGTTATTGGGCCATCTGGCC**CAATAATTAA**GAGAACGTGTTCAAAA
TCTGGGTTTGACCGAAGGTCAAGGTAGACGGTCAACAATATTCTGGCTTGCGGAGCAAGCAACAAGAATTAAATATTTAATTCGTATGACACGTGGACGGACCGAAATACTCCTGTATCT
CTATAAATAGCCTAAATCTGGCATGGATAATTGCTCTCGCTCTTCTGTCAAAGCTGTTGTGTTGAGGCGGAAGATCGCCATCGGCGATCATCAGACGAAAAGCTGCAAGAGAGACGGAGA
ACCATGCTGCGAAGCGTATATCGGGTATTTATAGACTTCTAGCGCAGCTAGAAGTTTCCTTGTACTTGTATTTGTATTTTGTAAATTACGAAGAAATTCGTACATTGATAAT**AATAAA**AC
ATCTGGGATTGTTAATGTTTACATTAACTAGTATTAATAATGTACAATAACAAACATTGTATGAGGAACGAATACAAGAATATTAAAATATGAGGAGCGTAGCGTGATAAACAGGTGTTT
AAGGAATAATTAAAATAATTATGCAATGTTATGATAATACGGTATAAGTTGAAGTATGAGGTGAAAGAGGAGATATTAGAATATTAAAAACCCAATTATATTATTTATAATACATACAAC
ACGCTATGACAAAAGGGGAAAAAAGAAAAATCGGGGGTTGATTGGTCTATCCTAACGATTAAGGGCCGCAGGCCCGTCAAGATGGATGAACGGTTAGATTTGATTGCTTAGACACGAAGA
AAGGAATCTTTTTGGGACCACAGATAAGACAGCTGTCACTACTTTTAAAAAATAATATAATAACCAAATGACCATAGTGCCCCTCCCAAAGATGACGTATAGGGGTGTCCCGAGGTAATT
TAACATAGCTCTGATAAGAGATAGTGGACGTTGGATTCCACGATCGGACGATCGTGGTTTGATGAACTTATGATGACGTAGGGGCTGGGGCTTAT

>U3_alyu53
TATTACCCCCAGCCCCCAGGACGGGACATGGGCTTTTTAAATGGGCCGAGAGAGTTTGAACAGTTCAGTATCTTCGTTATTGGGCCATCTGGCC**CAATAATTAA**GAGAACGTGTTCAAAA
TCTGGGTTTGACCGAAGGTCAAGGTAGACGGTCAACAATATTCTGGCTTGCGGAGCAAGCAACAAGAATTAAATATTTAATTCGTATGACACGTGGACGGACCGAAATACTCCTGTATCT
CTATAAATAGCCTAAATCTGCTATGGATAATTGCTCTCGCTCTTCTGTCAAAGGTGTTGTGTTGAGGCGGAAGATCGCCATCGGCGATCATCAGACGAAAAGCTGCAAGAGAGACGGAGA
ACCATGCTGCGAAGCGTATATCGGGTATTTATAGACTTATAGCGCAGCTAGAAGTATACTTGTACTTGATATTGTATTTTGTAAATTACGAAGAAATTCGTATTGATAAT**AATAAA**ACAT
CTGGGATTGTTAATGTTTACATTAACTAGTATTAATAATGTACAATAACAAAAATTGTATGAGGAACGAATACAAGAATATTAAAATAAGAGGAGCGTAGCGTGATAAACAGGTGTTTAA
GGTATAATTAAAATAATTATGCAAATGTCATGATAATACGGTATAAGTTGAAGTATGAGGTGAAAGAGGAGATATTAGAATATTAAAAACCCAATTATATTATTTATAATACATACAACA
CGCTATGACAAAAGGGGAAAAATGAAGAATCGGGGGTTGATTGGTCTATCCTAACGATTAAGGGCCGCAGGCCCGTCAAGATGGATGAACGGTTAGATTTGATTGCTTAGCCACGAAGAA
AGGAATCTTTTTGGGACCACAGATAAGACAGCTGTCACTACCTTTAAAAATAATATAATAACCAATTGACGATAGTACCCCTCCCAAAGATGACGTATAGGGGTGTCCCGAGGTAATTTA
ACATAGCTCTGACAAGAGATAGTGGACGTTGGATGCCACGATCGGACGATCGTGGTTTGATGAACTTATGATGACGTAGGGGCTGGGGCTTAT

>U3_alyu54_v1
TATTACCCCCAGCCCCCAGGACGGGACATGGGCTTTTTAAATGGGCCGAGAGAGTTTGAACAGTTCAGTATCTTCGTTATTGGGCCATCTGGCC**CAATAATTAA**GAGAACGTGTTCAAAA
TCTGGGTTTGACCGAAGGTCAAGGTAGACGGTCAACAATATTCTGGCTTGCGGAGCAAGCAACAAGAATTAAATATTTAATTCGTATGACACGTGGACGGACCGAAATACTCCTGCATCT
CTATAAATAGCCTAAATCTGGCATGGATAATTGCTCTCGCTCTTCTGTCAAAGGTTTTGTGTTGAGGCGGAAGATCGCCATCGGCGATCATCGGACGAAAAGCTGCAAGAGAGACGGAGA
ACCATGCTGCGAAGCGTATAACGGGTATTTATAGACTTCTAGCGCAGCTAGAAGTATTCTTGTACTTGATATTGTATTTTGTAAATTACGAAGAAATTCGTACATTGATAAT**AATAAA**AC
ATCTGGGATTGTTAATTTTTACATTAACCAGTATTAATAATGTACAATAACAAATATTGTATGAGGAACGAATACAAGAATATTAAAATATGAGGAGCGTAGCGTGATAAACAGGTGTTT
AAGGTATAATTAAATTAATTATGCAATGTCATGATAATACGGTATAAGTTGAAGTATGAGGTGAAAGAGGAGATATTAGAATATTAAAAACCCAATTATATTATTTATAATACATACAAC
ACGCTATGACAAACGGGGAAAAAAGAAAAATCGGGGGTTGATTGGTCTATCCTAACGATTAAGGGCCGCAGGCCCGTCAAGATGGATGAACGGTTAGATTTGATTGCTTAGCCACGAAGA
AAGGAATCGTTTTGGGACCACAGACAAGACAGCTGTCACTACTTTTAAAAAATAATATAATAACCAAATGACGATAGTACCCCTCCCAAAGATGACGTATAGGGGTGTCCCGAGGTAATT
TAACATAGCTCTGACAAGAGATAGTGGACGTTGGATGCCACGATCGGACGATCGTGGTTTGACGAACCAATGATGACGTAGGGGCTGGGGCTTAT

>U3_alyu54_v1

TATTACCCCCAGCCCCCAGGACGGGACATGGGCTTTTTAAATGGGCCGAGAGAGTTTGAACAGTTCAGTATCTTCGTTATTGGGCCATCTGGCC**CAATAATTAA**GAGAACGTGTTCAAAA
TCTGGGTTTGACCGAAGGTCAAGGTAGACGGTCAACAATATTCTGGCTTGCGGAGCAAGCAACAAGAATTAAATATTTAATTCGTATGACACGTGGACGGACCGAAATACTCCTGCATCT
CTATAAATAGCCTAAATCTGGCATGGATAATTGCTCTCGCTCTTCTGTCAAAGGTTgTGTGTTGAGGCGGAAGATCGCCATCGGCGATCATCGGACGAAAAGCTGCAAGAGAGACGGAGA
ACCATGCTGCGAAGCGTATAACGGGTATTTATAGACTTCTAGCGCAGCTAGAAGTATTCTTGTACTTGATATTGTATTTTGTAAATTACGAAGAAATTCGTACATTGATAAT**AATAAA**AC
ATCTGGGATTGTTAATTTTTACATTAACCAGTATTAATAATGTACAATAACAAATATTGTATGAGGAACGAATACAAGAATATTAAAATATGAGGAGCGTAGCGTGATAAACAGGTGTTT
AAGGTATAATTAAATTAATTATGCAATGTCATGATAATACGGTATAAGTTGAAGTATGAGGTGAAAGAGGAGATATTAGAATATTAAAAACCCAATTATATTATTTATAATACATACAAC
ACGCTATGACAAACGGGGAAAAAAGAAAAATCGGGGGTTGATTGGTCTATCCTAACGATTAAGGGCCGCAGGCCCGTCAAGATGGATGAACGGTTAGATTTGATTGCTTAGCCACGAAGA
AAGGAATCGTTTTGGGACCACAGACAAGACAGCTGTCACTACTTTTAAAAAATAATATAATAACCAAATGACGATAGTACCCCTCCCAAAGATGACGTATAGGGGTGTCCCGAGGTAATT
TAACATAGCTCTGACAAGAGATAGTGGACGTTGGATGCCACGATCGGACGATCGTGGTTTGACGAACCAATGATGACGTAGGGGCTGGGGCTTAT

>U3_alyu55
TATTACCCCCAGCCCCCAGGACGGGACATGGGCTTTTTAAATGGGCCGAGAGAGTTTGAACAGTTCAGTATCTTCGTTATTGGGCCATCTGGCC**CAATAATTAA**GAGAACGTGTTCAAAA
TCTGGGTTTGACCGAAGGTCAAGGTAGACGGTCAACAATATTCTGGCTTGCGGAGCAAGCAACAAGAATTAAATATTTAATTCTTAAGACACGTGGACGGACCGAAATACTCCTGTATCT
CTATAAATAGCCTAAATCTGGCCTGGATAATTGCTCTCGCTCTTCTGTCAAAGCTGTTGTGTTGAGGCGGAAGATCGCCACCGGCGATCATCGGACGAAAAGCTGCAAGAGAGACGGAGA
ACCATGCTACGAAGCGTATATCGGGTATTTATAGACTTCTAGCGCAGCTAGAAGTTTCCTTGTACTTGTTATTGTATTTTGTAAAGTACGAAGAAATTCGTATATTGATAAT**AATAAA**AC
ATCTGGGTTTGTTAATGTTTACATTAACCAGTATTAATAATGTACAATAACAAACATTGTATGAGGAACGAATACAAGAATATTAAAAAATGAGGAGCGTAGCGTGATAAACAGGTGTTT
AAGGTATAATTACAATAATTATGCAAAGTCATGATAATACGGTATAAGTTGAAGTATGAGGTGAAAGAGGAGGTATTAGAATATTAAAAACCCAATTATATTATTTATAATACATACAAC
ACGCTATGACAAACGGGGAAAAAAGAAAAATCGGGGGTTGATTGGTCTATCCTAACGATTAAGGGCCGCAGGCCCGTCAAGATGGATGAACGGTTAGATTTGATTGCTTAGCCACGAAGA
AAGGAATCGTTTTGGGACCACAGACAAGACAGCTGTCACTACCTTTAAAAAATAATATAATAACCAATTGACGATAGTACCCCTCCCAAAGATGACGTATAGGGGTGTCCCGAGGTAATT
TAACATAGCTCTGACAAGAGATAGTGGACGTTGGATGCCACGATCGGACGATCGTGGTTTGATGAACTTATGATGACGTAGGGGCTGGGGCTTAT

>U3_alyu56
TATTACCCCCAGCCCCCAGGACGGGACATGGGCTTTTTAAATGGGCCGAGAGAGTTTGAACAGTTCAGTATCTTCGTTATTGGGCCATCTGGCC**CAATAATTAA**GAGAACGTGTTCAAAA
TCGGGGTTTGACCGAAGGTCAAGGTAGACGGTCAACATTATTCTGGCTTGCGGAGCAAGCAACACGAATTAAATATTTAATTCGTATGACACGTGGACGGACCGAAATACTCCTGTATCT
CTATAAATAGCCTAAATCTGGCTTGGATAATTGCTCTCGCTCTTCTGTCAAAGCTGCTGTGTTGAGGCGGAAGATCGCCATCGGCGATCATCGGACGAAAAGCTGCAAGAGAGACGGAGA
ACCATGCTACGAAGCGTATATCGGGTATTTATAGACTTCTAGCGCAGCTAGAAGTTTCCTTGTACTTGATATTGTATTTTGTAAATTACGAAGAAATTCGTATATTGATAAT**AATAAA**AC
ATCTGGGATTGTTAATGTTTACATTAACCAGTATTAATAATGTACAATAACAAATATTGTATGAGGAACGAATACAAGATTATTAAAATATGAGGAGCGTAGCGTGATAAACAGGTGTTT
AAGGTATAATTAAATTAATTATGTAATGTCATTATAATACGGTATAAGTTGAAGTATGAGGTGAAAGAGGAGATATTAGAATATTAAAAACCCAATTATATTATTTATAATACATACAAC
ACGCTATGACAAACGGGGAAAAAAGAAAAATCGGGGGTTGATTGGTCTATCCTAACGATTAAGGGCCGCAGGCCCGTCAAGATGGATGAACGGTTAGATTTGATTGCTTAGCTACGAAGA
AAGGAATCGTTTTGGGACCACAGACAAGACAGCTGTCACTACTTTTAAAAAATAATATAATAACCAATTGACGATAGTACCCCTACCAAAGATGACGTATAGGGGTGTCCCGAGGTAATT
TAACATAGCTCTGACAAGAGATAGTGGACGTTGGATGCCACGATCGGACGATCGTGGTTTGATGAACCAATGATGACGTAGGGGCTGGGGCTTAT

CLUSTAL multiple sequence alignment by MUSCLE (3.8)

```
                                                   Start codon SEA      Premature stop PIO
U3_alyu14         TATTACCCCCCGTGCTCGGGACGGGACATGGGCTTTTTAAATGGGCCTTGAGAGTTTGAA
U3_alyu15         TATTACCCCCCGTGCTCGGGACGGGACATGGGCTTTTTAAATGGGCCTTGAGAGTTTGAA
U3_alyu19         TATTACCCCCAGCGCTCAGGACGGGACATGGGCTTTTTAAATGGGCCTTGAGAGTTTGAA
U3_jgf1_v1        TATTACCCCCCGTGCTCGGGACGGGACATGGGCTTTTTAAATGGGCCTTGAGAGTTTGAA
U3_jgf1_v2        TATTACCCCCCGTGCTCGGGACGGGACATGGGCTTTTTAAATGGGCCTTGAGAGTTTGAA
U3_alyu17_v1      TATTACCCCCAGCGCTCAGGACGGGACATGGGCTTTTTAAATGGGCCTTGAGAGTTTGAA
U3_alyu17_v2      TATTACCCCCCGTGCTCGGGACGGGACATGGGCTTTTTAAATGGGCCTTGAGAGTTTGAA
U3_alyu21_v1      TATTACCCCCAGCGCTCGGGACGGGACATGGGCTTTTTAAATGGGCCTTGAGAGTTTGAA
U3_alyu21_v2      TATTACCCCCCGTGCTCGGGACGGGACATGGGCTTTTTAAATGGGCCTTGAGAGTTTGAA
U3_alyu18         TATTACCCCCCGTGCTCGGGACGGGACATGGGCTTTTTAAATGGGCCTTGAGAGTTTGAA
U3_alyu24         TATTACCCCCCGTGCTCGGGACGGGACATGGGCTTTTTAAATGGGCCTTGAGAGTTTGAA
U3_alyu16         TATTACCCCCCGTGCTCGGGACGGGACATGGGCTTTTTAAATGGGCCTTGAGAGTTTGAA
U3_alyu22         TATTACCCCCCGTGCTCGGGACGGGACATGGGCTTTTTAAATGGGCCTTGAGAGTTTGAA
U3_alyu41         TATTACCCCCAGCCCCCAGGACGGGACATGGGCTTTTTAAATGGGCCGAGAGAGTTTGAA
U3_alyu53         TATTACCCCCAGCCCCCAGGACGGGACATGGGCTTTTTAAATGGGCCGAGAGAGTTTGAA
U3_alyu50         TATTACCCCCAGCCCCCAGGACGGGACATGGGCTTTTTAAATGGGCCGAGAGAGTTTGAA
U3_alyu47         TATTACCCCCAGCCCCCAGGACGGGACATGGGCTTTTTAAATGGGCCGAGAGAGTTTGAA
U3_alyu49         TATTACCCCCAGCCCCCAGGACGGGACATGGGCTTTTTAAATGGGCCGAGAGAGTTTGAA
U3_alyu51         TATTACCCCCAGCCCCCAGGACGGGACATGGGCTTTTTAAATGGGCCGAGAGAGTTTGAA
U3_alyu48_v2      TATTACCCCCAGCCCCCAGGACGGGACATGGGCTTTTTAAATGGGCCGAGAGAGTTTGAA
U3_alyu48_v1      TATTACCCCCAGCCCCCAGGACGGGACATGGGCTTTTTAAATGGGCCGAGAGAGTTTGAA
U3_alyu42_43      TATTACCCCCAGCCCCCAGGACGGGACATGGGCTTTTTAAATGGGCCGAGAGAGTTTGAA
U3_alyu43_v2      TATTACCCCCAGCCCCCAGGACGGGACATGGGCTTTTTAAATGGGCCGAGAGAGTTTGAA
U3_alyu44_45      TATTACCCCCAGCCCCCAGGACGGGACATGGGCTTTTTAAATGGGCCGAGAGAGTTTGAA
U3_alyu55         TATTACCCCCAGCCCCCAGGACGGGACATGGGCTTTTTAAATGGGCCGAGAGAGTTTGAA
U3_alyu26         TATTACCCCCAGCCCCCAGGACGGGACATGGGCTTTTTAAATGGGCCGAGAGAGTTTGAA
U3_alyu54_v1      TATTACCCCCAGCCCCCAGGACGGGACATGGGCTTTTTAAATGGGCCGAGAGAGTTTGAA
U3_alyu54_v1      TATTACCCCCAGCCCCCAGGACGGGACATGGGCTTTTTAAATGGGCCGAGAGAGTTTGAA
U3_alyu35         TATTACCCCCAGCCCCCAGGACGGGACATGGGCTTTTTAAATGGGCCGAGAGAGTTTGAA
U3_alyu34_v2      TATTACCCCCAGCCCCCAGGACGGGACATGGGCTTTTTAAATGGGCCGAGAGAGTTTGAA
U3_alyu56         TATTACCCCCAGCCCCCAGGACGGGACATGGGCTTTTTAAATGGGCCGAGAGAGTTTGAA
U3_alyu25         TATTACCCCCAGCCCCCAGGACGGGACATGGGCTTTTTAAATGGGCCGAGAGAGTTTGAA
U3_alyu36         TATTACCCCCAGCCCCCAGGACGGGACATGGGCTTTTTAAATGGGCCGAGAGAGTTTGAA
U3_alyu34_v1      TATTACCCCCAGCCCCCAGGACGGGACATGGGCTTTTTAAATGGGCCGAGAGAGTTTGAA
U3_alyu39         TATTACCCCCAGCCCCCAGGACGGGACATGGGCTTTTTAAATGGGCCGAGAGAGTTTGAA
U3_alyu37         TATTACCCCCAGCCCCCAGGACGGGACATGGGCTTTTTAAATGGGCCGAGAGAGTTTGAA
U3_alyu33         TATTACCCCCAGCCCCCAGGACGGGACATGGGCTTTTTAAATGGGCCGAGAGAGTTTGAA
U3_alyu40         TATTACCCCCAGCCCCCAGGACGGGACATGGGCTTTTTAAATGGGCCGAGAGAGTTTGAA
U3_alyu29         TATTACCCCCAGCCCCCAGGACGGGACATGGGCTTTTTAAATGGGCCGAGAGAGTTTGAA
U3_alyu32         TATTACCCCCAGCCCCCAGGACGGGACATGGGCTTTTTAAATGGGCCGAGAGAGTTTGAA
                  ********** *  * * *****************************  ***********
                                                           TATA-box
U3_alyu14         CAGTTCAGTATCTTCGTTATTGGGCCAACTCGGCCCAATAATTAAGAGAACGTGTTCAAA
U3_alyu15         CAGTTCAGTATCTTCGTTATTGGGCCAACTCGGCCCAATAATTAAGAGAACGTGTTCAAA
U3_alyu19         CAGTTCAGTATCTTCGTTATTGGGCCAACTCGGCCCAATAATTAAGAGAACGTGTTCAAA
U3_jgf1_v1        CAGTTCAGTATCTTCGTTATTGGGCCAACTCGGCCCAATAATTAAGAGAACGTGTTCAAA
U3_jgf1_v2        CAGTTCAGTATCTTCGTTATTGGGCCAACTCGGCCCAATAATTAAGAGAACGTGTTCAAA
U3_alyu17_v1      CAGTTCAGTATCTTCGTTATTGGGCCAACCCGGCCCAATAATTAAGAGAACGTGTTCAAA
U3_alyu17_v2      CAGTTCAGTATCTTCGTTATTGGGCCAACCCGGCCCAATAATTAAGAGAACGTGTTCAAA
U3_alyu21_v1      CAGTTCAGTATCTTCGTTATTGGGCCAACTCGGCCCAATAATTAAGAGAACGTGTTCAAA
U3_alyu21_v2      CAGTTCAGTATCTTCGTTATTGGGCCAACTCGGCCCAATAATTAAGAGAACGTGTTCAAA
U3_alyu18         CAGTTCAGTATCTTCGTTATTGGGCCAACTCGGCCCAATAATTAAGAGAACGTGTTCAAA
U3_alyu24         CAGTTCAGTATCTTCGTTATTGGGCCAACTCGGCCCAATAATTAAGAGAACGTGTTCAAA
U3_alyu16         CAGTTCAGTATCTTCGTTATTGGGCCAACTCGGCCCAATAATTAAGAGAACGTGTTCAAA
U3_alyu22         CAGTTCAGTATCTTCGTTATTGGGCCAACTCGGCCCAATAATTAAGAGAACGTGTTCAAA
U3_alyu41         CAGTTCAGTATCTTCGTTATTGGGCCATCT-GGCCCAATAATTAAGAGAACGTGTTCAAA
U3_alyu53         CAGTTCAGTATCTTCGTTATTGGGCCATCT-GGCCCAATAATTAAGAGAACGTGTTCAAA
U3_alyu50         CAGTTCAGTATCTTCGTTATTGGGCCATCT-GGCCCAATAATTAAGAGAACGTGTTCAAA
U3_alyu47         CAGTTCAGTATCTTCGTTATTGGGCCATCT-GGCCCAATAATTAAGAGAACGTGTTCAAA
```

```
U3_alyu49        CAGTTCAGTATCTTCGTTATTGGGCCATCT-GGCCCAATAATTAAGAGAACGTGTTCAAA
U3_alyu51        CAGTTCAGTATCTTCGTTATTGGGCCATCT-GGCCCAATAATTAAGAGAACGTGTTCAAA
U3_alyu48_v2     CAGTTCAGTATCTTCGTTATTGGGCCATCT-GGCCCAATAATTAAGAGAACGTGTTCAAA
U3_alyu48_v1     CAGTTCAGTATCTTCGTTATTGGGCCATCT-GGCCCAATAATTAAGAGAACGTGTTCAAA
U3_alyu42_43     CAGTTCAGTATCTTCGTTATTGGGCCATCT-GGCCCAATAATTAAGAGAACGTGTTCAAA
U3_alyu43_v2     CAGTTCAGTATCTTCGTTATTGGGCCATCT-GGCCCAATAATTAAGAGAACGTGTTCAAA
U3_alyu44_45     CAGTTCAGTATCTTCGTTATTGGGCCATCT-GGCCCAATAATTAAGAGAACGTGTTCAAA
U3_alyu55        CAGTTCAGTATCTTCGTTATTGGGCCATCT-GGCCCAATAATTAAGAGAACGTGTTCAAA
U3_alyu26        CAGTTCAGTATCTTCGTTATTGGGCCATCT-GGCCCAATAATTAAGAGAACGTGTTCAAA
U3_alyu54_v1     CAGTTCAGTATCTTCGTTATTGGGCCATCT-GGCCCAATAATTAAGAGAACGTGTTCAAA
U3_alyu54_v1     CAGTTCAGTATCTTCGTTATTGGGCCATCT-GGCCCAATAATTAAGAGAACGTGTTCAAA
U3_alyu35        CAGTTCAGTATCTTCGTTATTGGGCCATCT-GGCCCAATAATTAAGAGAACGTGTTCAAA
U3_alyu34_v2     CAGTTCAGTATCTTCGTTATTGGGCCATCT-GGCCCAATAATTAAGAGAACGTGTTCAAA
U3_alyu56        CAGTTCAGTATCTTCGTTATTGGGCCATCT-GGCCCAATAATTAAGAGAACGTGTTCAAA
U3_alyu25        CAGTTCAGTATCTTCGTTATTGGGCCATCT-GGCCCAATAATTAAGAGAACGTGTTCAAA
U3_alyu36        CAGTTCAGTATCTTCGTTATTGGGCCATCT-GGCCCAATAATTAAGAGAACGTGTTCAAA
U3_alyu34_v1     CAGTTCAGTATCTTCGTTATTGGGCCATCT-GGCCCAATAATTAAGAGAACGTGTTCAAA
U3_alyu39        CAGTTCAGTATCTTCGTTATTGGGCCATCT-GGCCCAATAATTAAGAGAACGTGTTCAAA
U3_alyu37        CAGTTCAGTATCTTCGTTATTGGGCCATCT-GGCCCAATAATTAAGAGAACGTGTTCAAA
U3_alyu33        CAGTTCAGTATCTTCGTTATTGGGCCATCT-GGCCCAATAATTAAGAGAACGTGTTCAAA
U3_alyu40        CAGTTCAGTATCTTCGTTATTGGGCCATCT-GGCCCAATAATTAAGAGAACGTGTTCAAA
U3_alyu29        CAGTTCAGTATCTTCGTTATTGGGCCATCT-GGCCCAATAATTAAGAGAACGTGTTCAAA
U3_alyu32        CAGTTCAGTATCTTCGTTATTGGGCCATCT-GGCCCAATAATTAAGAGAACGTGTTCAAA
                 ************************** *  *****************************

                         Start codon PIO   Stop codon SEA
U3_alyu14        TTCGTG-GTATGACCGAAGGTCAAGGTAACCGGTCAACATTATTCTGGCTTGCGCAGCAA
U3_alyu15        TTCGTG-GTATGACCGAAGGTCAAGGTAACCGGTCAACATTATTCTGGCTTGCGCAGCAA
U3_alyu19        TTCGTG-GTATGACCGAAGGTCAAGGTAACCGGTCAACATTATTCTGGCTTGCGCAGCAA
U3_jgf1_v1       TTCGTG-GTATGACCGAAGGTCAAGGTAACCGGTCAACATTATTCTGGCTTGCGCAGCAA
U3_jgf1_v2       TTCGTG-GTATGACCGAAGGTCAAGGTAACCGGTCAACATTATTCTGGCTTGCGCAGCAA
U3_alyu17_v1     TTCGTG-GTATGACCGAAGGTCAAGGTAACCGGTCAACATTATTCTGGCTTGCGCAGCAA
U3_alyu17_v2     TTCGTG-GTATGACCGAAGGTCAAGGTAACCGGTCAACATTATTCTGGCTTGCGCAGCAA
U3_alyu21_v1     TTCGTG-GTATGACCGAAGGTCAAGGTAACCGGTCAACATTATTCTGGCTTGCGCAGCAA
U3_alyu21_v2     TTCGTG-GTATGACCGAAGGTCAAGGTAACCGGTCAACATTATTCTGGCTTGCGCAGCAA
U3_alyu18        TTCGTG-GTATGACCGAAGGTCAAGGTAACCGGTCAACATTATTCTGGCTTGCGCAGCAA
U3_alyu24        TTCGTG-GTATGACCGAAGGTCAAGGTAACCGGTCAACATTATTCTGGCTTGCGCAGCAA
U3_alyu16        TTCGTG-GTATGACCGAAGGTCAAGGTAACCGGTCAACATTATTCTGGCTTGCGCAGCAA
U3_alyu22        TTCGTG-GTATGACCGAAGGTCAAGGTAACCGGTCAACATTATTCTGGCTTGCGCAGCAA
U3_alyu41        ATCTGG-GTTTGACCGAAGGTCAAGGTAGACGGTCAACAATATTCTGGCTTGCGGAGCAA
U3_alyu53        ATCTGG-GTTTGACCGAAGGTCAAGGTAGACGGTCAACAATATTCTGGCTTGCGGAGCAA
U3_alyu50        ATCTGG-GTTTGACCGAAGGTCAAGGTAGACGGTCAACAATATTCTGGCTTGCGGAGCAA
U3_alyu47        ATCTGG-GTTTGACCGAAGGTCAAGGTAGACGGTCAACAATATTCTGGCTTGCGGAGCAA
U3_alyu49        ATCTGG-GTTTGACCGAAGGTCAAGGTAGACGGTCAACAATATTCTGGCTTGCGGAGCAA
U3_alyu51        ATCTGG-GTTTGACCGAAGGTCAAGGTAGACGGTCAACAATATTCTGGCTTGCGGAGCAA
U3_alyu48_v2     ATCTGG-GTTTGACCGAAGGTCAAGGTAGACGGTCAACAATATTCTGGCTTGCGGAGCAA
U3_alyu48_v1     ATCTGG-GTTTGACCGAAGGTCAAGGTAGACGGTCAACAATATTCTGGCTTGCGGAGCAA
U3_alyu42_43     ATCGGG-GTTTGACCGAAGGTCAAGGTAGACGGTCAACAATATTCTGGCTTGCGGAGCAA
U3_alyu43_v2     ATCGGG-GTTTGACCGAAGGTCAAGGTAGACGGTCAACAATATTCTGGCTTGCGGAGCAA
U3_alyu44_45     ATCGGG-GTTTGACCGAAGGTCAAGGTAGACGGTCAACAATATTCTGGCTTGCGGAGCAA
U3_alyu55        ATCTGG-GTTTGACCGAAGGTCAAGGTAGACGGTCAACAATATTCTGGCTTGCGGAGCAA
U3_alyu26        ATCTGG-GTTTGACCGAAGGTCAAGGTAGACGGTCAACAATATTCTGGCTTGCGGAGCAA
U3_alyu54_v1     ATCTGG-GTTTGACCGAAGGTCAAGGTAGACGGTCAACAATATTCTGGCTTGCGGAGCAA
U3_alyu54_v1     ATCTGG-GTTTGACCGAAGGTCAAGGTAGACGGTCAACAATATTCTGGCTTGCGGAGCAA
U3_alyu35        ATCGGG-GTTTGACCGAAGGTCAAGGTAGACGGTCAACAATATTCTGGCTTGCGGAGCAA
U3_alyu34_v2     ATCGGG-GTTTGACCGAAGGTCAAGGTAGACGGTCAACAATATTCTGGCTTGCGGAGCAA
U3_alyu56        ATCGGG-GTTTGACCGAAGGTCAAGGTAGACGGTCAACATTATTCTGGCTTGCGGAGCAA
U3_alyu25        ATCGGG-GTTTGACCGAAGGTCAAGGTAGACGGTCAACAATATTCTGGCTTGCGGAGCAA
U3_alyu36        ATCGGG-GTTTGACCGAAGGTCAAGGTAGACGGTCAACAATATTCTGGCTTGCGGAGCAA
U3_alyu34_v1     ATCGGG-GTTTGACCGAAGGTCAAGGTAGACGGTCAACAATATTCTGGCTTGCGGAGCAA
U3_alyu39        ATCGGG-GTTTGACCGAAGGTCAAGGTAGACGGTCAACAATATTCTGGCTTGCGGAGCAA
U3_alyu37        ATCGGG-GTTTGACCGAAGGTCAAGGTAGACGGTCAACAATATTCTGGCTTGCGAAGCAA
U3_alyu33        ATCGGG-GTTTGACCGAAGGTCAAGGTAGACGGTCAACAATATTCTGGCTTGCGGAGCAA
U3_alyu40        ATCGGGAGTTTGACCGAAGGTCAAGGTAGACGGTCAACAATATTCTGGCTTGCGGAGCAA
U3_alyu29        ATCTGG-GTGTGACCGAAGGTCAAGGTAGACGGTCAACAATATTCTGGCTTGCGGAGCAA
U3_alyu32        ATCTGG-GTTTGACCGAAGGTCAAGGTAGACGGTCAACAATATTCTGGCTTGCGGAGCAA
                  **  * ** ******************  ********* ************** *****

U3_alyu14        GCTACACGAATTAAATTATTAATTCGTAGGACACGTGGACGGACCGAAATACTCCTGCAT
U3_alyu15        GATACACGAATTAAATTATTAATTCGTAGGACACGTGGACGGACCGAAATACTCCTGCAT
U3_alyu19        GATACACGAATTAAATTATTAATTCGTAGGACACGTGGACGGACCGAAATACTCCTGCAT
U3_jgf1_v1       GATACACGAATTAAATTATTAATTCGTAGGACACGTGGACGGACCGAAATACTCCTGCAT
U3_jgf1_v2       GATACACGAATTAAATTATTAATTCGTAGGACACGTGGACGGACCGAAATACTCCTGCAT
U3_alyu17_v1     GATACACGAATTAAATAATTAATTCGTAGGACACGTGGACGGACCGAAATACTCCTGCAT
U3_alyu17_v2     GATACACGAATTAAATTATTAATTCGTAGGACACGTGGACGGACCGAAATACTCCTGCAT
U3_alyu21_v1     GATACACGAATTAAATTATTAATTCGTAGGACACGTGGACGGACCGAAATACTCCTGCAT
U3_alyu21_v2     GATACACGAATTAAATTATTAATTCGTAGGACACGTGGACGGACCGAAATACTCCTGCAT
U3_alyu18        GATACACGAATTAAATTATTAATTCGTAGGACACGTGGACGGACCGAAATACTCCTGCAT
U3_alyu24        GATACACGAATTAAATTATTAATTCGTAGGACACGTGGACGGACCGAAATACTCCTGCAT
U3_alyu16        GATACACGAATTAAATTATTAATTCGTAGGACACGTGGACGGACCGAAATACTCCTGCAT
U3_alyu22        GATACACGAATTAAATTATTAATTCGTAGGACACGTGGACGGACCGAAATACTCCTGCAT
U3_alyu41        GCAACAAGAATTAAATATTTAATTCGTATGACACGTGGACGGACCGAAATACTCCTGTAT
U3_alyu53        GCAACAAGAATTAAATATTTAATTCGTATGACACGTGGACGGACCGAAATACTCCTGTAT
U3_alyu50        GCAACAAGAATTAAATATTTAATTCGTATGACACGTGGACGGACCGAAATACTCCTGTAT
U3_alyu47        GCAACAAGAATTAAATATTTAATTCGTATGACACGTGGACGGACCGAAATACTCCTGTAT
U3_alyu49        GCAACAAGAATTAAATATTTAATTCGTATGACACGTGGACGGACCGAAATACTCCTGTAT
U3_alyu51        GCAACAAGAATTAAATATTTAATTCGTATGACACGTGGACGGACCGAAATACTCCTGTAT
U3_alyu48_v2     GCAACAAGAATTAAATATTTAATTCGTATGACACGTGGACGGACCGAAATACTCCTGTAT
U3_alyu48_v1     GCAACAAGAATTAAATATTTAATTCGTATGACACGTGGACGGACCGAAATACTCCTGTAT
U3_alyu42_43     GCAACACGAATTAAATATTTAATTCGTATGACACGTGGACGGACCGAAATACTCCTGTAA
U3_alyu43_v2     GCAACACGAATTAAATATTTAATTCGTATGACACGTGGACGGACCGAAATACTCCTGTAA
U3_alyu44_45     GCAACACGAATTAAATATTTAATTCGTATGACACGTGGACGGACCGAAATACTCCTGTAA
U3_alyu55        GCAACAAGAATTAAATATTTAATTCTTAAGACACGTGGACGGACCGAAATACTCCTGTAT
U3_alyu26        GCAACAAGAATTAAATATTTAATTCGTATGACACGTGGACGGACCGAAATACTCCTGCAT
U3_alyu54_v1     GCAACAAGAATTAAATATTTAATTCGTATGACACGTGGACGGACCGAAATACTCCTGCAT
U3_alyu54_v1     GCAACAAGAATTAAATATTTAATTCGTATGACACGTGGACGGACCGAAATACTCCTGCAT
```

```
U3_alyu35         GCAACACGAATTAAATATTTAATTCGTATGACACGTGGACGGACCGAAATACTCCTGTAT
U3_alyu34_v2      GCAACAAGAATTAAATATTTAATTCTTATGACACGTGGACGGACCGAAATACTCCTGTAT
U3_alyu56         GCAACACGAATTAAATATTTAATTCGTATGACACGTGGACGGACCGAAATACTCCTGTAT
U3_alyu25         GCAACACGAATTAAATATTTAATTCGTAAGACACGTGGACGGACCGAAATACTCCTGTAT
U3_alyu36         GCAACACGAATTAAATATTTAATTCGTAAGACACGTGGACGGACCGAAATACTCCTGAAT
U3_alyu34_v1      GCAACACGAATTAAATATTTAATTCGTATGACACGTGGACGGACCGAAATACTCCTGTAT
U3_alyu39         GCAACACGAATTAAATATTTAATTCGTATGACACGTGGACGGACCGAAATACTCCTGTAA
U3_alyu37         GCAACACGAATTAAATATTTAATTCGTATGACACGTGGACGGACCGAAATACTCCTGTAT
U3_alyu33         GCAACACGAATTAAATATTTAATTCGTA-GACACGTGGACGGACCGAAATACTCCTGTAT
U3_alyu40         GCAACACGAATTAAATATTTAATTCGTATGACACGTGGACGGACCGAAATACTCCTGTAC
U3_alyu29         GCAACACGAATTAAATATCTAATTCGTAAGACACGTGGACGGACCGAAATACTCCTGTAT
U3_alyu32         GCAACACGAATTAAATATTTAATTCGTATGACACGTGGACGGACCGAAATACTCCTGCAT
                  *  *** *********   ****** ** *************************** *
                    TATA-box                        miR156 family target site
U3_alyu14         CTCTATAAATACCCTAGTCTTGTGAAGGATAATTGCTCTCTCTCTTCTGTCAAGGTGGTT
U3_alyu15         CTCTATAAATACCCTAGTCTTGTGAAGGATAATTGCTCTCTCTCTTCTGTCAAGGTGGTT
U3_alyu19         CTCTATAAATACCCTAGTCCTGTGAAGGATAATTGCTCTCTCTCTTCTGTCAAGGTGGTT
U3_jgf1_v1        CTCTATAAATACCCTAGTCCTGTGAAGGATAATTGCTCTCTCTCTTCTGTCAAGGTGGTT
U3_jgf1_v2        CTCTATAAATACCCTAGTCCTGTGAAGGATAATTGCTCTCTCTCTTCTGTCAAGGTGGTT
U3_alyu17_v1      CTCTATAAATACCCTAGTCCTGTGAAGGATAATTGCTCTCTCTCTTCTGTCAAGGTGGTT
U3_alyu17_v2      CTCTATAAATACCCTAGTCCTGTGAAGGATAATTGCTCTCTCTCTTCTGTCAAGGTGGTT
U3_alyu21_v1      CTCTATAAATACCCTAGTCATGTGAAGGATAATTGCTCTCTCTCTTCTGTCAAGGTGGTT
U3_alyu21_v2      CTCTATAAATACCCTAGTCATGTGAAGGATAATTGCTCTCTCTCTTCTGTCGAGGTGGTT
U3_alyu18         CTCTATAAATACCCTAGTCCTGTGAAGGATAATTGCTCTCTCTCTTCTGTCAAGGTTGTT
U3_alyu24         CTCTATAAATACCCTAGTCCTGTGAAGGATAATTGCTCTCTCTCTTCTGTCAAGGTGGTT
U3_alyu16         CTCTATAAATACCCTAGTCCTGTGAAGGATAATTGCTCTCTCTCTTCTGTCAAGGTGGTT
U3_alyu22         CTCTATAAATACCCTAGTCCTGTGAAGGATAATTGCTCTCTCTCTTCTGTCAAGGTGGTT
U3_alyu41         CTCTATAAATAGCCTAAATCTGGCATGGATAATTGCTCTCGCTCTTCTGTCAAAGCTGTT
U3_alyu53         CTCTATAAATAGCCTAAATCTGCTATGGATAATTGCTCTCGCTCTTCTGTCAAAGGTGTT
U3_alyu50         CTCTATAAATAGCCTAAATCTGGCATGGATAATTGCTCTCGCTCTTCTGTCAAAGCTGTT
U3_alyu47         CTCTATAAATAGCCTAAATCTGGCATGGATAATTGCTCTCGCTCTTCTGTCAAAGCTGTT
U3_alyu49         CTCTATAAATAGCCTAAATCTGGCATGGATAATTGCTCTCGCTCTTCTGTCAAAGCTGTT
U3_alyu51         CTCTATAAATAGCCTAAATCTGGCATGGATAATTGCTCTCGCTCTTCTGTCAAAGCTGTT
U3_alyu48_v2      CTCTATAAATAGCCTAAATCTGGCATGGATAATTGCTCTCGCTCTTCTGTCAAAGCTGTT
U3_alyu48_v1      CTCTATAAATAGCCTAAATCTGGCATGGATAATTGCTCTCGCTCTTCTGTCAAAGCTGTT
U3_alyu42_43      ATCTATAAATAGCCTAAATCTGGCCTGGATAATTGCTCTCGCTCTTCTGTCAAAGCTGTT
U3_alyu43_v2      ATCTATAAATAGCTTAAATCTGGCTTGGATAATTGCTCTCGCTCTTCTGTCAAAGGTGTT
U3_alyu44_45      CTCTATAAATAGCTTAAATCTGGCTTGGATAATTGCTCTCGCTCTTCTGTCAAAGGTGTT
U3_alyu55         CTCTATAAATAGCCTAAATCTGGCCTGGATAATTGCTCTCGCTCTTCTGTCAAAGCTGTT
U3_alyu26         CTCTATAAATAGCCTAAAACTGGCATGGATAATTGCTCTCGCTCTTCTGTCAAAGCTATT
U3_alyu54_v1      CTCTATAAATAGCCTAAATCTGGCATGGATAATTGCTCTCGCTCTTCTGTCAAAGGTTTT
U3_alyu54_v1      CTCTATAAATAGCCTAAATCTGGCATGGATAATTGCTCTCGCTCTTCTGTCAAAGGTTGT
U3_alyu35         CTCTATAAATAGCTTAAATCTGGCTTGGATAATTGCTCTCGCTCTTCTGTCAAAGCTGTT
U3_alyu34_v2      CTCTATAAATAGCTTAAATCTGGCTTGGATAATTGCTCTCGCTCTTCTGTCAAAGGTGTT
U3_alyu56         CTCTATAAATAGCCTAAATCTGGCTTGGATAATTGCTCTCGCTCTTCTGTCAAAGCTGCT
U3_alyu25         CTCTATAAATAGCTTAAATCTGGCTTGGATAATTGCTCTCGCTCTTCTGTCAAAGGTGTT
U3_alyu36         CTCTATAAATAGCTTAAATCTGGCTTGGATAATTGCTCTCGCTCTTCTGTCAAAGCTGTT
U3_alyu34_v1      CTCTATAAATAGCCTAAATCTGGCTTGGATAATTGCTCTCGCTCTTCTGTCAAAGCTGTT
U3_alyu39         CTCTATAAATAGCTTAAATCTGGCTTGGATAATTGCTCTCGCTCTTCTGTCAAAGCTGTT
U3_alyu37         CTCTATAAATAGCTTAAATCTGGCTTGGATAATTGCTCTCGCTCTTCTGTCAAAGCTGTT
U3_alyu33         CTCTATAAATAGCTTAAATCTGGCTTGGATATTTGCTCTCGCTCTTCTGTCAAAGCTGTT
U3_alyu40         CTCTATAAATAGCTTAAAACTGGCTTGGATAATTGCTCTCGCTCTTCTGTCAAAGCTGTT
U3_alyu29         CTCTATAAATAGCTTAAATCTGGCTTGGATAATTACTCTCGCTCTTCTGTCAAAGCTGTT
U3_alyu32         CTCTATAAATAGCTTAAATCTGGCTTGGATAATTGCTCTCGCTCTTCTGTCAAAGATGTT
                   ********** * **    **    ***** ** ***** ********** * *    *

U3_alyu14         GTGCGGAGGCGGAAGATCGCCAGCGGCGATCGTCGGAACGATGTGCATCTAGAGAGGCGG
U3_alyu15         GTGCGGAGGCGGAAGATCGCCAGCGGCGATCGTCGGAACGATGTGCATCTAGAGAGGCGG
U3_alyu19         GTGCTGAGGCGGAAGATCGCCAGCGGCGATCGTCGGAACGATGTGCATCTAGAGAGGCGG
U3_jgf1_v1        GTGCTGAGGCGGAAGATCGCCAGCGGCGATCGTCGGAACGATGTGCATCTAGAGAGGCGG
U3_jgf1_v2        GTGCTGAGGCGGAAGATCGCCAGCGGCGATCGTCGGAACGATGTGCATCTAGAGAGGCGG
U3_alyu17_v1      GTGCTGAGGCGGAAGATCGCCAGCGGCGATCGTCGGAACGATGTGCATCTAGAGAGGCGG
U3_alyu17_v2      GTGCTGAGGCGGAAGATCGCCAGCGGCGATCGTCGGAACGATGTGCATCTAGAGAGGCGG
U3_alyu21_v1      GTGCTGAGGCGGAAGATCGCCAGCGGCGATCGTCGGAACGATGTGCATCTAGAGAGGCGG
U3_alyu21_v2      GTGCTGAGGCGGAAGATCGCCAGCGGCGATCGTCGGAACGATGTGCATCTAGAGAGGCGG
U3_alyu18         GTGCTGAGGCGGAAGATCGCCAGCGGCGATCGTCGGAACGATGTGCATCTAGAGAGGCGG
U3_alyu24         GTGCTGAGGCGGAAGATCGCCAGCGGCGATCGTCGGAACGATGTGCATCTAGAGAGGCGG
U3_alyu16         GTGCTGAGGCGGAAGATCGCCAGCGGCGATCGTCGGAACGATGTGCATCTAGAGAGGCGG
U3_alyu22         GTGCTGAGGCGGAAGATCGCCAGCGGCGATCGTCGGAACGATGTGCATCTAGAGAGGCGG
U3_alyu41         GTGTTGAGGCGGAAGATCGCCATCGGCGATCATCAG-ACGAAAAGCTGCAAGAGAGACGG
U3_alyu53         GTGTTGAGGCGGAAGATCGCCATCGGCGATCATCAG-ACGAAAAGCTGCAAGAGAGACGG
U3_alyu50         GTGTTGAGGCGGAAGATCGCCATCGGCGATCATCAG-ACGAAAAGCTGCAAGAGAGACGG
U3_alyu47         GTGTTGAGGCGGAAGATCGCCATCGGCGATCATCAG-ACGAAAAGCTGCAAGAGAGACGG
U3_alyu49         GTGTTGAGGCGGAAGATCGCCATCGGCGATCATCAG-ACGAAAAGCTGCAAGAGAGACGG
U3_alyu51         GTGTTGAGGCGGAAGATCGCCATCGGCGATCATCAG-ACGAAAAGCTGCAAGAGAGACGG
U3_alyu48_v2      GTGTTGAGGCGGAAGATCGCCATCGGCGATCATCAG-ACGAAAAGCTGCAAGAGAGACGG
U3_alyu48_v1      GTGTTGAGGCGGAAGATCGCCATCGGCGATCATCAG-ACGAAAAGCTGCAAGAGAGACGG
U3_alyu42_43      GTGTTGAGGCGGAAGATCGCCATCGGCGATCATCAG-ACGAAAAGCTGCAAGAGAGACGG
U3_alyu43_v2      GTGTTGAGGCGGAAGATCGCCATCGGCGATCATCAG-ACGAAAAGCTGCAAGAGAGACGG
U3_alyu44_45      GTGTTGAGGCGGAAGATCGCCATCGGCGATCATCAG-ACGAAAAGCTGCAAGAGAGACGG
U3_alyu55         GTGTTGAGGCGGAAGATCGCCACCGGCGATCATCGG-ACGAAAAGCTGCAAGAGAGACGG
U3_alyu26         GTGTTGAGGCGGAAGATCGCCATCGGCGATCATCGG-ACGAAAAGCTGCAAGAGAGACGG
U3_alyu54_v1      GTGTTGAGGCGGAAGATCGCCATCGGCGATCATCGG-ACGAAAAGCTGCAAGAGAGACGG
U3_alyu54_v1      GTGTTGAGGCGGAAGATCGCCATCGGCGATCATCGG-ACGAAAAGCTGCAAGAGAGACGG
U3_alyu35         GTGTTGAGGCGGAAGATCGCCATCGGCGATCATCGG-ACGAAAAGCTGCAAGAGAGACGG
U3_alyu34_v2      GTGTTGAGGCGGAAGATCGCCATCGGCGATCATCGG-ACGAAAAGCTGCAAGAGAGACGG
U3_alyu56         GTGTTGAGGCGGAAGATCGCCATCGGCGATCATCGG-ACGAAAAGCTGCAAGAGAGACGG
U3_alyu25         GTGTTGAGGCGGAAGATCGCCATCGGCGATCATCGG-ACGAAAAGCTGCAAGAGAGACGG
U3_alyu36         GTGTTGAGGCGGAAGATCGCCATCGGCGATCATCGG-ACGAAAAGCTGCAAGAGAGACGG
U3_alyu34_v1      GTGTTGAGGCGGAAGATCGCCATCGGCGATCATCGG-ACGAAAAGCTGCAAGAGAGACGG
U3_alyu39         GTGTTGAGGCGGAAGATCGCCATCGGCGATCATCGG-ACGAAAAGCTGCAAGAGAGACGG
U3_alyu37         GTGTTGAGGCGGAAGATCGCCATCGGCGATCATCGG-ACGAAAAGCTGCAAGAGAGACGG
U3_alyu33         GTGTTGAGGCGGAAGATCGCCATCGGCGATCATCAG-ACGAAAAGCTGCAAGAGAGACGG
U3_alyu40         GTGTTGAGGCGGAAGATCGCCATCGGCGATCATCAG-ACGAAAAGCTGCAAGAGAGACGG
U3_alyu29         GTGTTGAGGCGGAAGATCGCCATCGGCGATCATCAG-ACGAAAAGCTGCAAGAGAGACGG
```

```
U3_alyu32         GTGTTGAGGCGGAAGATCGCCATCGGCGATCATCAG-ACGAAAAGCTGCAAGAGAGACGG
                  ***  ***************** ******** ** * ****   **  * ****** ***
                  Stop codon PIO
U3_alyu14         TGAAGGAAACTACGAAGCATATATCGGGTATTTATAGACTTATAGCGCAGCTAGAAGTAT
U3_alyu15         TGAAGGAAACTACGAAGCATATATCGGGTATTTATAGACTTATAGCGCAGCTAGAAGTAT
U3_alyu19         TGAAGCAAACTACGAAGCATATATCGGGTATTTATAGACTTATAGCGCAGCTAGAAGTAT
U3_jgf1_v1        TGAAGCAAACTACGAAGCGTATATCGGGTATTTATAGACTTATAGCGCAGCTAGAAGTAT
U3_jgf1_v2        TGAAGCAAACTACGAAGCGTATATCGGGTATTTATAGACTTATAGCGCAGCTAGAAGTAT
U3_alyu17_v1      TGAAGGAAACTACGAAGCATATATCGGGTATTTATAGACTTATAGCGCAGCTAGAAGTAT
U3_alyu17_v2      TGAAGCAAACTACGAAGCATATATCGGGTATTTATAGACTTATAGCGCAGCTAGAAGTAT
U3_alyu21_v1      TGAAGCAAACTACGAAGCATATATCGGGTATTTATAGACTTATAGCGCAGCTAGAAGTAT
U3_alyu21_v2      TGAAGCAAACTACGAAGCATATATCGGGTATTTATAGACTTATAGCGCAGCTAGAAGTAT
U3_alyu18         TGAAGCAAACTACGAAGCATATATCGGGTATTTATAGACTTATAGCGCAGCTAGAAGTAT
U3_alyu24         TGAAGCAAACTACGAAGCATATATCGGGTATTTATAGACTTATAGCGCAGCTAGAAGTAT
U3_alyu16         TGAAGCAAACTACGAAGCATATATCGGGTATTTATAGACTTATAGCGCAGCTAGAAGTAT
U3_alyu22         TGAAGCAAACTACGAAGCATATATCGGGTATTTATAGACTTATAGCGCAGCTAGAAGTAT
U3_alyu41         AGAACCATGCTGCGAAGCGTATATCGGGTATTTATAGACTTCTAGCGCAGCTAGAAGTTT
U3_alyu53         AGAACCATGCTGCGAAGCGTATATCGGGTATTTATAGACTTATAGCGCAGCTAGAAGTAT
U3_alyu50         AGAACCATGCTGCGAAGCGTATATCGGGTATTTATAGACTTCTAGCGCAGCTAGAAGTTT
U3_alyu47         AGAACCATGCTGCGAAGCGTATATCGGGTATTTATAGACTTCTAGCGCAGCTAGAAGTTT
U3_alyu49         AGAACCATGCTGCGAAGCGTATATCGGGTATTTATAGACTTCTAGCGCAGCTAGAAGTTT
U3_alyu51         AGAACCATGCTGCGAAGCGTATATCGGGTATTTATAGACTTCTAGCGCAGCTAGAAGTTT
U3_alyu48_v2      AGAACCATGCTGCGAAGCGTATATCGGGTATTTATAGACTTCTAGCGCAGCTAGAAGTTT
U3_alyu48_v1      AGAACCATGCTGCGAAGCGTATATCGGGTATTTATAGACTTCTAGCGCAGCTAGAAGTTT
U3_alyu42_43      AGAAACATGCTGCGAAGCGTATATCGGGTATTTATAGACTTCTAGCGCAGCTAGAAGTTT
U3_alyu43_v2      AGAAACATGCTGCGAAGCGTATATCGGGTATTTATAGACTTCTAGCGCAGCTAGAAGTTT
U3_alyu44_45      AGAAACATGCTGCGAAGCGTATATCGGGTATTTATAGACTTCTAGCGCAGCTAGAAGTTT
U3_alyu55         AGAACCATGCTACGAAGCGTATATCGGGTATTTATAGACTTCTAGCGCAGCTAGAAGTTT
U3_alyu26         ATAACCATGCTGCGAAGCGTATATCGGGTATTTATAGACTTCTAGCGCAGCTAGAAGTTT
U3_alyu54_v1      AGAACCATGCTGCGAAGCGTATAACGGGTATTTATAGACTTCTAGCGCAGCTAGAAGTAT
U3_alyu54_v1      AGAACCATGCTGCGAAGCGTATAACGGGTATTTATAGACTTCTAGCGCAGCTAGAAGTAT
U3_alyu35         AGAACCATGCCACGAAGCGTATATCGGGTATTTATAGACTTCTAGCGCAGCTAGAAGTTT
U3_alyu34_v2      AGAACCATGCTGCGAAGCGTATATCGGGTATTTATAGACTTCTAGCGCAGCTAGAAGTTT
U3_alyu56         AGAACCATGCTACGAAGCGTATATCGGGTATTTATAGACTTCTAGCGCAGCTAGAAGTTT
U3_alyu25         AGAACCATGCTACGAAGCGTATATCGGGTATTTATAGACTTCTAGCGCAGCTAGAAGTAT
U3_alyu36         AGAACCATGCTACGAAGCGTATATCGGGTATTTATAGACTTCTAGCGCAGCTAGAAGTTT
U3_alyu34_v1      AGAACCATGCTACGAAGCGTATATCGGGTATTTATAGACTTCTAGCGCAGCTAGAAGTTT
U3_alyu39         AGAACCATGCTACGAAGCGTATATCGGGTATTTATAGACTTCTAGCGCAGCTAGAAGTTT
U3_alyu37         AGAACCATGCTACGAAGCGTATATCGGGTATTTATAGACTTCTAGCGCAGCTTGAAGTTT
U3_alyu33         AGAACCATGCTGCGAAGCGTATATCCGGTATTTATAGACTTCTAGCGCAGCTAGAAGTTT
U3_alyu40         AGAACCATGCTGCGAAGCGTATATCGGGTATTTATAGACTTCTAGCGCAGCTAGAAGTTT
U3_alyu29         AGAACCATGCTGCGAAGCGTATATCGGGTATTTATAGACTTCTAGCGCAGCTAGAAGTTT
U3_alyu32         AGAACCATGCTGCGAAGCGTATATCGGGTATTTATAGACTTCTAGCGCAGCTAGAAGTTT
                    **  *  *  ****** **** * *************** ********** ***** *

U3_alyu14         ACACTGTAC-AGATTTTGTATTTTGTA--AATATACGAAGAATTTCGTATA-TGATATTA
U3_alyu15         ACACTGTAC-AGATTTTGTATTTTGTA--AATATACGAAGAATTTCGTATA-TGATATTA
U3_alyu19         ATACTGTAC-AGATTTTGTATTTTGTA--AAT-TACGAAGAATATCGTATA-TGATATTA
U3_jgf1_v1        ACACTGTAC-AGATTATGTATTTTGTA--AAT-TACGAAGAATTTCGTATA-TGATATTA
U3_jgf1_v2        ACACTGTAC-AGATTATGTATTTTGTA--AAT-TACGAAGAATTTCGTATA-TGATATTA
U3_alyu17_v1      ACACTGTAC-AGATTTTGTATTTTGTA--AAT-TACGAAGAATATCGTATA-TGATATTA
U3_alyu17_v2      ACACTGTAC-AGATTATGTATTTTGTA--AAT-TACGAAGAATATCGTATA-TGATATTA
U3_alyu21_v1      ACACTGTAC-AGATTTTGTATTTTGTA--AAT-TACGAAGAATTTCGTATA-TGATATTA
U3_alyu21_v2      ACACTGTAC-AGATTTTGTATTTTGTA--AAT-TACGAAGAATTTCGTATA-TGATATTA
U3_alyu18         ACACTGTAC-AGATATTGTATTTTGTATCATT-TACGAAGAATTTCGTATA-TGATATTA
U3_alyu24         ACACTGTAC-AGATTTTGTATTTTGTA--CAT-TACGAAGAATTTCGTATA-TGATATTA
U3_alyu16         ACACTGTAC-AGATTTTGTATTTTGTA--AAT-TACGAAGAATTTCGTATA-TGATATTA
U3_alyu22         ACACTGTAC-AGATTTTGTATTTTGTA--AAT-TACGAAGAATTTCGTATA-TGATATTA
U3_alyu41         CC-TTGTACTTGTATTTGTATTTTGTA--AAT-TACGAAGAAATTCGTACATTGATAATA
U3_alyu53         AC-TTGTACTTGATATTGTATTTTGTA--AAT-TACGAAGAAATTCGTAT--TGATAATA
U3_alyu50         CC-TTGTACTTGTATTTGTATTTTGTA--AAT-TACGAAGAAATTCGTACATTGATAATA
U3_alyu47         CC-TTGTACTTGTATTTGTATTTTGTA--AAT-TACGAAGAAATTCGTACATTGATAATA
U3_alyu49         CC-TTGTACTTGTATTTGTATTTTGTA--AAT-TACGAAGAAATTCGTACATTGATAATA
U3_alyu51         CC-TTGTACTTGTATTTGTATTTTGTA--AAT-TACGAAGAAATTCGTACATTGATAATA
U3_alyu48_v2      CC-TTGTACTTGTATTTGTATTTTGTA--AAT-TACGAAGAAATTCGTACATTGATAATA
U3_alyu48_v1      CC-TTGTACTTGTATTTGTATTTTGTA--AAT-TACGAAGAAATTCGTACATTGATAATA
U3_alyu42_43      CG-TTGTATTTGATATTGTATTTTGTA--AAT-TACGAAGAA-TTCGTACATTGATAATA
U3_alyu43_v2      CT-TTGTACTTGATATTGTATTTTGTA--AAT-TACGAAGAAA-TCGTACATTGATAATA
U3_alyu44_45      CG-TTGTACTTGATATTGTATTTTGTA--AAT-TACGAAGAAA-TCGTACATTGATAATA
U3_alyu55         CC-TTGTACTTGTTATTGTATTTTGTA--AAG-TACGAAGAAATTCGTATATTGATAATA
U3_alyu26         CC-TTGTATTTGATATTGTATTTTGTA--AAT-TACGAAGAAATTCGTACATTGATAATA
U3_alyu54_v1      TC-TTGTACTTGATATTGTATTTTGTA--AAT-TACGAAGAAATTCGTACATTGATAATA
U3_alyu54_v1      TC-TTGTACTTGATATTGTATTTTGTA--AAT-TACGAAGAAATTCGTACATTGATAATA
U3_alyu35         CC-TTGTACTTGATATTGTATTTTGTA--AAT-TACGAAGAAATTCGTATATTGATAATA
U3_alyu34_v2      CC-TTGTACTTGATATTGTATTTTGTA--AAT-TACGAAGAAATTCGTATATTGATAATA
U3_alyu56         CC-TTGTACTTGATATTGTATTTTGTA--AAT-TACGAAGAAATTCGTATATTGATAATA
U3_alyu25         CC-TTGTACTTGATATTGTATTTTGTA--AAT-TACGAAGAAATTCGTATATTGATAATA
U3_alyu36         CC-TTGTACTTGATATTGTATTTTGTA--AAT-TACGAAGAAATTCGTATATTGATAATA
U3_alyu34_v1      CC-TTGTACTTGTTATTGTATTTTGTA--AAT-TACGAAGAAATTCGTATATTGATAATA
U3_alyu39         CC-TTGTACTTGATATTGTATTTTGTA--AAT-TACGAAGAAATTCGTATATTGATAATA
U3_alyu37         CC-TTGTACTTGATATTGTATTTTGTA--AAT-TACGAAGAAATTCGTATATTGATAATA
U3_alyu33         CC-CTGTACTTGATATTGTATTTTGTA--AAT-TACGAAGAAATTCGTACATTGATAATA
U3_alyu40         CC-TTGTACTTGATATTGTATTTTGTA--AAT-TACGAAGAAATTCGTAGATTGATAATA
U3_alyu29         CC-TTGTACTTGATATTGTATTTTGTA--AAT-CACGAAGAAATTCGTACATTGATAATA
U3_alyu32         CC-TTGTACTTGATTTTGTATTTTGTA--AAT-TACGAAGAAATTCGTATATTGATAATA
                      ****   *    ***********        ********  *****   ***** **
                 Poly(A) signal PIO (mapped by Bentham et al. 1999 and Guyot et al. 2022)
U3_alyu14         ATAAAACACCTGGGTGTTTTAATGTTTACATTAAATAGTATCCTGAATGTACACAATAA
U3_alyu15         ATAAAACACCTGGGT-GTTTTAATGTTTACATTAAATAGTATCCTGAATGTACACAATAA
U3_alyu19         ATAAAACACCTGGGT-GTGTTAATGTTTACATTAAATAGTATCCTGAATGTACACAATAA
U3_jgf1_v1        ATAAAACACCTGGGT-GTGTTAATGTTTACATTAAATAGTATCCTGAATGTACACAATAA
U3_jgf1_v2        ATAAAACACCTGGGT-GTGTTAATGTTTACATTAAATAGTATCCTGAATGTACACAATAA
U3_alyu17_v1      ATAAAACACCTGGGT-GTGTTAATGTTTACATTAAATAGTATCCTGAATGTACACAATAA
U3_alyu17_v2      ATAAAACACCTGGGT-GTGTTAATGTTTACATTAAATAGTATCCTGAATGTACACAATAA
U3_alyu21_v1      ATAAAACACCTGGGT-GTGTTAATGTTTACATTAAATTGTATCCTGAATGTACACAATAA
```

```
U3_alyu21_v2      ATAAAACACCTGGGT-GTGTTAATGTTTACATTAAATTGTATCCTGAATGTACACAATAA
U3_alyu18         ATAAAACACCTGGGT-GTTTTAATGTTTACATTAAATAGTATCCTGAATGTACACAATAA
U3_alyu24         ATAAAACACCTGGGT-GTGTTAATGTTTACATTAAATAGTATCCTGAATGTACACAATAA
U3_alyu16         ATAGAACACCTGGGT-GTGTTAATGTTTACATTAAATAGTATCCTGAATGTACACAATAA
U3_alyu22         ATAAAACACCTGGGT-GTGTTAATGTTTACATTAAATAGTATCCTGAATGTACACAATAA
U3_alyu41         ATAAAACATCTGGGA-TTGTTAATGTTTACATTAACTAGTATTAATAATGTACAATAACA
U3_alyu53         ATAAAACATCTGGGA-TTGTTAATGTTTACATTAACTAGTATTAATAATGTACAATAACA
U3_alyu50         ATAAAACATCTGGGA-TTGTTAATGTTTACATTAACTAGTATTAATAATGTACAATAACA
U3_alyu47         ATAAAACATCTGGGA-TTGTTAATGTTTACATTAACTAGTATTAATAATGTACAATAACA
U3_alyu49         ATAAAACATCTGGGA-TTGTTAATGTTTACATTAACTAGTATTAATAATGTACAATAACA
U3_alyu51         ATAAAACATCTGGGA-TTGTTAATGTTTACATTAACTAGTATTAATAATGTACAATAACA
U3_alyu48_v2      ATAAAACATCTGGGA-TTGTTAATGTTTACATTAACTAGTATTAATAATGTACAATAACA
U3_alyu48_v1      ATAAAACATCTGGGA-TTGTTAATGTTTACATTAACTAGTATTAATAATGTACAATAACA
U3_alyu42_43      ATAAAACATCTGGGA-TTGTTAATGTTTACATTAACCAGTATTAATAATGTACAATAAAA
U3_alyu43_v2      ATAAAACATCTGGGA-TTGTTAATGTTTACATTAACCAGTATTAATAATGTACAATAAAA
U3_alyu44_45      ATAAAACATCTGGGA-TTGTTAATGTTTACATTAACCAGTATTAATAATGTACAATAAAA
U3_alyu55         ATAAAACATCTGGGT-TTGTTAATGTTTACATTAACCAGTATTAATAATGTACAATAACA
U3_alyu26         ATAAAACATCTGGGA-TTGTTAATGTTTACATTAACCAGTATTAATAATGTACAATAACA
U3_alyu54_v1      ATAAAACATCTGGGA-TTGTTAATTTTTACATTAACCAGTATTAATAATGTACAATAACA
U3_alyu54_v1      ATAAAACATCTGGGA-TTGTTAATTTTTACATTAACCAGTATTAATAATGTACAATAACA
U3_alyu35         ATAAAACATCTGGGA-TTGTTAATGTTTACATTAACCAGTATTAATAATGTACAATAACA
U3_alyu34_v2      ATAAAACATCTGGGA-TTGTTAATGTTTACATTAACTAGTATTAATAATGTACAATAACA
U3_alyu56         ATAAAACATCTGGGA-TTGTTAATGTTTACATTAACCAGTATTAATAATGTACAATAACA
U3_alyu25         ATAAAACATCTGGGA-CTGTTAATGTTTACATTAACCAGTATTAATAATGTATAATAACA
U3_alyu36         ATAAAACATCTGGGA-CTGTTAATGTTTACATTAACCAGTATTAATAATGTATAATAACA
U3_alyu34_v1      ATAAAACATCTGGGA-TTGTTAATGTTTACATTAACCAGTATTAATAATGTACAATAACA
U3_alyu39         ATAAAACATCTGGGA-TTGTTAATGTTTACATTAACTAGTATTAATAATGTACAATAACA
U3_alyu37         ATAAAACATCTGGGA-TTGTTAATGTTTACATTAACCAGTATTAATAATGTACAATAACA
U3_alyu33         ATAAAACATCTGGGT-TTGTTAATGTTTACATTAACCAGTATCAATAATGTACAATAACA
U3_alyu40         ATAAAACATCTGGGT-TTGTTAATGTTTACATTAACCAGTATTAATAATGTACAATAACA
U3_alyu29         ATAAAACATCTGGGA-TTGTTAATGTTTACATTAACCAGTATTAAAAATGTACAATAACA
U3_alyu32         ATAAAACATCTGGGA-TTGTTAATGTTTACATTAACCAGTATTAATAATGTACAATAACA
                  *** **** *****   * ***** **********   ****     ****** *  *  *

U3_alyu14         AATACAGTATACGGAACGTATACTGA-CAAAGTAATAAATGATAGGCGAAGCATGATTAA
U3_alyu15         AATACAGTATACGGAACGTATACTGA-CAAAGTAATAAATGATAGGCGAAGCATGATTAA
U3_alyu19         AATACAGTATACGGAACGTATACTGA-CAAAGTAATAAATGATAGGCGAAGCATGATTAA
U3_jgf1_v1        AATACAGTATACGGAACGTATACTGA-CAAAGTAATAAATGATAGGCGAAGCATGATTAA
U3_jgf1_v2        AATACAGTATACGGAACGTATACTGA-CAAAGTAATAAATGATAGGCGAAGCATGATTAA
U3_alyu17_v1      AATACAGTATACGGAACGTATACTGACAAAAGTAATACATGATAGGCGAAGCATGATTAA
U3_alyu17_v2      AATACAGTATACGGAACGTATACTGACAAAAGTAATACATGATAGGCGAAGCATGATTAA
U3_alyu21_v1      AATACAGTATACGGAACGTATACTGA-CAAAGTAATAAATGATAGGCGAAGCATGATTAA
U3_alyu21_v2      AATACAGTATACGGAACGTATACTGA-CAAAGTAATAAATGATAGGCGAAGCATGATTAA
U3_alyu18         AATACAGTATACGGAACGTATACTGA-CAAAGTAATAAATGATAGGCGAAGCATGATTAA
U3_alyu24         AATACAGTATACGGAACGTATACTGA-CAAAGTAATAACTGATAGGCGAAGCATGATTAA
U3_alyu16         AATACAGTATACGGAACGTATACTGA-CAAAGTAATAAATGATAGGCGAAGCATGATTAA
U3_alyu22         AATACAGTATACGGAACGTATACTGA-CAAAGTAATAACTGATAGGCGAAGCATGATTAA
U3_alyu41         AACATTGTATGAGGAACGAATACAAG-AATATTAAAATATGAGGAGCGTAGCGTGATAAA
U3_alyu53         AAAATTGTATGAGGAACGAATACAAG-AATATTAAAATAAGAGGAGCGTAGCGTGATAAA
U3_alyu50         AACATTGTATGAGGAACGAATACAAG-AATATTAAAATATGAGGAGCGTAGCGTGATAAA
U3_alyu47         AACATTGTATGAGGAACGAATACAAG-AATATTAAAATATGAGGAGCGTAGCGTGATAAA
U3_alyu49         AACATTGTATGAGGAACGAATACAAG-AATATTAAAATATGAGGAGCGTAGCGTGATAAA
U3_alyu51         AACATTGTATGAGGAACGAATACAAG-AATATTAAAATATGAGGAGCGTAGCGTGATAAA
U3_alyu48_v2      AACATTGTATGAGGAACGAATACAAG-AATATTAAAATATGAGGAGCGTAGCGTGATAAA
U3_alyu48_v1      AACATTGTATGAGGAACGAATACAAG-AATATTAAAATATGAGGAGCGTAGCGTGATAAA
U3_alyu42_43      AAAATTGTATGAGGATCGAATACAAT-AATATTAAAATATGAGGAGCGTAGCATGATAAA
U3_alyu43_v2      AATATTGTATGAGGAACGAATACAAT-AATATTAAAATATGAGGAGCGTAGCATGATAAA
U3_alyu44_45      AATATTGTATGAGGAACGAATACAAT-AATATTAAAATATGAGGAGCGTAGCATGATAAA
U3_alyu55         AACATTGTATGAGGAACGAATACAAG-AATATTAAAAAATGAGGAGCGTAGCGTGATAAA
U3_alyu26         AATATTGTATGAGGAACGAATACAAT-AATATTAAAATATGAGGAGCGTAGCGTGATAAA
U3_alyu54_v1      AATATTGTATGAGGAACGAATACAAG-AATATTAAAATATGAGGAGCGTAGCGTGATAAA
U3_alyu54_v1      AATATTGTATGAGGAACGAATACAAG-AATATTAAAATATGAGGAGCGTAGCGTGATAAA
U3_alyu35         AATATTGTATGAGGAACGAATACAAA-AATATTAAAATATGAGGAGCGTAGCATGATAAA
U3_alyu34_v2      AATATTGTATGAGGAACGAATACAAG-AATATTAAAA-ATGAGGAGCGTAGCGTGATAAA
U3_alyu56         AATATTGTATGAGGAACGAATACAAG-ATTATTAAAATATGAGGAGCGTAGCGTGATAAA
U3_alyu25         AATATTGTATGAGGAACGAATACAAT-AATATTAAAATATGTGGAGCGTAGCGTGATAAA
U3_alyu36         AATATTGTATGAGGAACGAATACAAG-AATATTAAAATATGAGGAGCGTAGCGTGATAAA
U3_alyu34_v1      AATATTGTATGAGGAACGAATACAAG-AATATTAAAATATGAGGAGCGTAGCGTGATAAA
U3_alyu39         AATATTGTATGAGGAACGAATACAAG-AATAATAAAATATGAGGAGCGTAGCGTGATAAA
U3_alyu37         AATATTGTATGAGGAACGAATACAAG-AATATTAAAATATGAGGAGCGTAGCGTGATAAA
U3_alyu33         AATATTGTATGAGGAACGAATACAAT-AATATTAAAATATGAGGAGCGTAGCGTGATAAA
U3_alyu40         AAAATTGTATGAGGAACGAATACAAT-AATATTAAAATATGAGGAGCGTAGCGTGATAAA
U3_alyu29         AATATTGTATGAGGAACGAATACAAT-AATATTAAAATATGAGGAGCGTAGCGTGATAAA
U3_alyu32         AATATTGTATGAGGAACGAATACAAT-AATATTAAAATATGAGGAGCGTAGCGTGATAAA
                  ** *  ****  *** ** ****       * *** *    *     *** *** **** **

U3_alyu14         CAGGTGTTT-AGGTATAATTAACATAATTATGA-GAAGTAATTATAATACGGAAAATGAA
U3_alyu15         CAGGTGTTT-AGGTATAATTAACATAATTATGA-GAAGTAATTATAATACGGAAAATGAA
U3_alyu19         CAGGTGTTT-AGGTATAATTAACATAATTATGA-GAAGTAATAATAATACGGAAAATGAA
U3_jgf1_v1        CAGGTGTTT-AGGTATAATTAACATAATTATGA-GAAGTAATAATAATACGGAAAATGAA
U3_jgf1_v2        CAGGTGTTT-AGGTATAATTAACATAATTATGA-GAAGTAATAATAATACGGAAAATGAA
U3_alyu17_v1      CAGGTGTTT-AGGTATAATTAACATAATTATGA-GAAGTAATAATAATACGGAAAATGAA
U3_alyu17_v2      CAGGTGTTT-AGGTATAATTAACATAATTATGA-GAAGTAATAATAATACGGAAAATGAA
U3_alyu21_v1      CAGGTGTTT-AGGTATAATTAACATAATTATGA-GAAGTAATAATAATACGGAAAATGAA
U3_alyu21_v2      CAGGTGTTT-AGGTATAATTAACATAATTATGA-GAAGTAATAATAATACGGAAAATGAA
U3_alyu18         CAGGTGTTT-AGGTATAATTAACATAATTATGA-GAAGTAATAATAATACGGAAAATGAA
U3_alyu24         CAGGTGTTT-AGGTATAATTAACATAATTATGA-GAAGTAATAATAATACGGAAAATGAA
U3_alyu16         CAGGTGTTT-AGGTATAATTAACATAATTATGA-GAAGTAATAATAATACGGAAAATGAA
U3_alyu22         CAGGTGTTT-AGGTATAATTAACATAATTATGA-GAAGTAATAATAATACGGAAAATGAA
U3_alyu41         CAGGTGTTTAAGGAATAATTAAAATAATTATGC-AATGTTATGATAATACGGTATAAGTT
U3_alyu53         CAGGTGTTTAAGGTATAATTAAAATAATTATGCAAATGTCATGATAATACGGTATAAGTT
U3_alyu50         CAGGTGTTTAAGGAATAATTAAAATAATTATGC-AATGTTATGATAATACGGTATAAGTT
U3_alyu47         CAGGTGTTTAAGGAATAATTAAAATAATTATGC-AATGTTATGATAATACGGTATAAGTT
U3_alyu49         CAGGTGTTTAAGGAATAATTAAAATAATTATGC-AATGTTATGATAATACGGTATAAGTT
U3_alyu51         CAGGTGTTTAAGGAATAATTAAAATAATTATGC-AATGTTATGATAATACGGTATAAGTT
```

```
U3_alyu48_v2      CAGGTGTTTAAGGAATAATTAAAATAATTATGC-AATGTTATGATAATACGGTATAAGTT
U3_alyu48_v1      CAGGTGTTTAAGGAATAATTAAAATAATTATGC-AATGTTATGATAATACGGTATAAGTT
U3_alyu42_43      CAGGTGTTTAAGGTATAATTAAAAAAATTATGC-AATGTTATGATAATACGGTATAAGTT
U3_alyu43_v2      CAGGTGTTTAAGGTATAATTAAAAAAATTATGC-AATGTTATGATAATACGGTATAAGTT
U3_alyu44_45      CAGGTGTTTAAGGTATAATTAAAAAAATTATGT-AATGTTATGATAATACGGTATAAGTT
U3_alyu55         CAGGTGTTTAAGGTATAATTACAATAATTATGC-AAAGTCATGATAATACGGTATAAGTT
U3_alyu26         CAGGTGTTTAAGGTATAATTAAATTAATTATGC-AATGTAATGATAATACGGTATAAGTT
U3_alyu54_v1      CAGGTGTTTAAGGTATAATTAAATTAATTATGC-AATGTCATGATAATACGGTATAAGTT
U3_alyu54_v1      CAGGTGTTTAAGGTATAATTAAATTAATTATGC-AATGTCATGATAATACGGTATAAGTT
U3_alyu35         CAGGTGTTTAAGGTATAATTAAATTAATTATGC-AATGTCATGATAATACGGTATAAGTT
U3_alyu34_v2      CAGGTGTTTAAGGTATAATTAAATTAATTATGC-AATGTCATGATAATACGGTATAAGTT
U3_alyu56         CAGGTGTTTAAGGTATAATTAAATTAATTATGT-AATGTCATTATAATACGGTATAAGTT
U3_alyu25         CAGGTGTTTAAGGTATAATTAAATTAATTATGC-AATGTCATGATAATACGGTATAAGTT
U3_alyu36         CAGGTGTTTAAGGTATAATTAAATTAATTATGC-AATGTCATGATAATACGGTATAAGTT
U3_alyu34_v1      CAGGTGTTTAAGGTATAATTAAAATAATTATGC-AAAGTAATGATAATACGGTATAAGTT
U3_alyu39         CAGTTGTTTAAGGTATAATTAAATTAATTATGC-AATGTCCTGATAATACGGTATAAGTT
U3_alyu37         CAGGTGTTTAAGGTATAATTAAATTAATTATGC-AATGTCATGATAATACGGTATAAGTT
U3_alyu33         CAGGTGTTTAAGGTATAATTAAATTAATTATGC-AATGTCATGATAATACGGTATAAGTT
U3_alyu40         CAGGTGTTTAAGGTATAATTAAATTAATTATGC-AAAGTCATGATAATACGGTATAAGTT
U3_alyu29         CAGGTGTTTAAGGTATAATTAAATTAATTATGC-AATGTCATGATAATACGGTATAAGTT
U3_alyu32         CAGGTGTTTAAGGTATAATTAAATTAATTATGC-AATGTCATGATAATACGGTATAAGTT
                  *** ***** *** *******    *******   * **  * ********* * * *

U3_alyu14         TAAGTATGAGGTGAAAGAGGAGATATTAGAATATTTAAAAACCCAATTATATTATTTTGG
U3_alyu15         TAAGTATGAGGTGAAAGAGGAGATATTAGAATATTTAAAAACCCAATTATATTATTTTGG
U3_alyu19         TAAGTATGAGGTGAAAGAGGAGATATTAGAATATTTAAAAACCCAATTATATTATTTTGG
U3_jgf1_v1        TAAGTATGAGGTGAAAGAGGAGATATTAGAATATTTAAAAACCCAATTATATTATTTTGG
U3_jgf1_v2        TAAGTATGAGGTGAAAGAGGAGATATTAGAATATTTAAAAACCCAATTATATTATTTTGG
U3_alyu17_v1      TAAGTATGAGGTGAAAGAGGAGATATTAGAATATTTAAAAACCCAATTATATTATTTTGG
U3_alyu17_v2      TAAGTATGAGGTGAAAGAGGAGATATTAGAATATTTAAAAACCCAATTATATTATTTTGG
U3_alyu21_v1      TAAGTATGAGGTGAAAGAGGAGATATTAGAATATTTAAAAACCCAATTATATTATTTTGG
U3_alyu21_v2      TAAGTATGAGGTGAAAGAGGAGATATTAGAATATTTAAAAACCCAATTATATTATTTTGG
U3_alyu18         TAAGTATGAGGTGAAAGAGGAGATTTTAGAATATTTAAAAACCCAATTATATTATTTTGG
U3_alyu24         TAAGTATGAGGTGAAAGAGGAGATATTAGAATATTTAAAAACCCATTTATATTATTTTGG
U3_alyu16         TAAGTATGAGGTGAAAGAGGAGATATTAGAATATTTAAAAACCCAATTATATTATTTTGG
U3_alyu22         TAAGTATGAGGTGAAAGAGGAGATATTAGAATATTTAAAAACCCAATTATATTATTTTGG
U3_alyu41         GAAGTATGAGGTGAAAGAGGAGATATTAGAATATT-AAAAACCCAATTATATTATTT--A
U3_alyu53         GAAGTATGAGGTGAAAGAGGAGATATTAGAATATT-AAAAACCCAATTATATTATTT--A
U3_alyu50         GAAGTATGAGGTGAAAGAGGAGATATTAGAATATT-AAAAACCCAATTATATTATTT--A
U3_alyu47         GAAGTATGAGGTGAAAGAGGAGATATTAGAATATT-AAAAACCCAATTATATTATTT--A
U3_alyu49         GAAGTATGAGGTGAAAGAGGAGATATTAGAATATT-AAAAACCCAATTATATTATTT--A
U3_alyu51         GAAGTATGAGGTGAAAGAGGAGATATTAGAATATT-AAAAACCCAATTATATTATTT--A
U3_alyu48_v2      GAAGTATGAGGTGAAAGAGGAGATATTAGAATATT-AAAAACCCAATTATATTATTT--A
U3_alyu48_v1      GAAGTATGAGGTGAAAGAGGAGATATTAGAATATT-AAAAACCCAATTATATTATTT--A
U3_alyu42_43      GAAGTATGAGGTGAAAGAGGAGATATTAGAATATT-AAAAACCCAATTATATTATTT--A
U3_alyu43_v2      GAAGTATGAGGTGAAAGAGGAGATATTAGAATATT-AAAAACCCAATTATATTATTT--A
U3_alyu44_45      GAAGTATGAGGTGAAAGAGGAGATATTAGAATATT-AAAAACCCAATTATATTATTT--A
U3_alyu55         GAAGTATGAGGTGAAAGAGGAGGTATTAGAATATT-AAAAACCCAATTATATTATTT--A
U3_alyu26         GAAGTATGAGGTGAAAGAGGAGATATTAGAATATT-AAAAACCCAATTATATTATTT--A
U3_alyu54_v1      GAAGTATGAGGTGAAAGAGGAGATATTAGAATATT-AAAAACCCAATTATATTATTT--A
U3_alyu54_v1      GAAGTATGAGGTGAAAGAGGAGATATTAGAATATT-AAAAACCCAATTATATTATTT--A
U3_alyu35         GAAGTATGAGGTGAAAGAGGAGATATAAGAATATT-AAAAACCCAATTATATTATTT--A
U3_alyu34_v2      GAAGTATGAGGTGAAAGAGGAGATATTAGAATATT-AAAAACCCAATTATATTATTT--A
U3_alyu56         GAAGTATGAGGTGAAAGAGGAGATATTAGAATATT-AAAAACCCAATTATATTATTT--A
U3_alyu25         GAAGTATGAGGTGAAAGAGGAGATATTAGAATATT-AAAAACCCAATTATATTATTT--A
U3_alyu36         GAAGTATGAGGTGAAAGAGGAGATATTAGAATATT-AAAAACCCAATTATATTATTT--A
U3_alyu34_v1      GAAGTATGAGGTGAAAGAGGAGATATTAGAATATT-AAAAACCCAATTATATTATTT--A
U3_alyu39         GAAGTATGAGGTGAAAGAGGAGATATTAGAATATT-AAAAACCCAATTATATTATTT--A
U3_alyu37         GAAGTATGAGGTGAAAGAGGAGATATTAGAATATT-AAAAACCCAATTATATTATTT--A
U3_alyu33         GAAGTATGAGGTGAAAGAGGAGATATTAGAATATTAAAAAACCCAATTATATTATTT--A
U3_alyu40         GAAGTATGAGGTGAAAGAGGAGATATTAGAATATT-AAAAACCCAATTATATTATTT--A
U3_alyu29         GAAGTATGAGGTGAAAGAGGAGATATTAGAATATT-AAAAACCCAATTATATTATTT--A
U3_alyu32         GAAGTATGAGGTGAAAGAGGAGATATTAGAATATT-AAAAACCCAATTATATTATTT--A
                   ********************* * * ******** ********* ***********

U3_alyu14         AACGAAATACAACACGCTATGAAATACAAGACGCTATGACAAAT--ACGGGTATCTGATT
U3_alyu15         AACGAAATACAACACGGTATGAAATACAAGACGCTATGACAAAT--ACGGGTATCTGATT
U3_alyu19         AACGAAATACAACACGCTATGAAATACAAGACGCTATGACAAATGTACGGGTATCTGATT
U3_jgf1_v1        AACGAAATACAACACGCTATGAAATACAAGACGCTATGACAAATGTACGGGTATCTGATT
U3_jgf1_v2        AACGAAATACAACACGCTATGAAATACAAGACGCTATGACAAATGTACGGGTATTTGATT
U3_alyu17_v1      AACGAAATACAACACACTATGAAATACAAGACGCTATGACAAATGTACGGGTATCTGATT
U3_alyu17_v2      AACGAAATACAACACACTATGAAATACAAGACGCTATGACAAATGTACGGGTATCTGATT
U3_alyu21_v1      AACGAAATACAACACACTATGAAATACAAGGCGCTATGACAAATGTACGGGTATTTGATT
U3_alyu21_v2      AACGAAATACAACACACTATGAAATACAAGGCGCTATGACAAATGTACGGGTATTTGATT
U3_alyu18         AACGAAATACAACACGCTATGAAATACAAGACGCTATGACAAATGTACGGGTATCTGATT
U3_alyu24         AACGAAATACAAGACGCTATGAAATACAAGACGCTATGACAAATGTACGGGTATCTGATT
U3_alyu16         AACGAAATACAACACGCTATGAAATACAAGACGCTATGACAAATGTACGGGTATCTGATT
U3_alyu22         AACGAAATACAACACGCTATGAAATACAAGACGCTATGACAAATGTACGGGTATCTGATT
U3_alyu41         TAATACATACAACACGCTATGACAAAAGGGGAAAAAAGAAAAAT---CGGGGGT-TGATT
U3_alyu53         TAATACATACAACACGCTATGACAAAAGGGGAAAAAATGAAGAAT---CGGGGGT-TGATT
U3_alyu50         TAATACATACAACACGCTATGACAAAAGGGGAAAAAAGAAAAAT---CGGGGGT-TGATT
U3_alyu47         TAATACATACAACACGCTATGACAAAAGGGGAAAAAAGAAAAAT---CGGGGGT-TGATT
U3_alyu49         TAATACATACAACACGCTATGACAAAAGGGGAAAAAAGAAAAAT---CGGGGGT-TGATT
U3_alyu51         TAATACATACAACACGCTATGACAAAAGGGGAAAAAAGAAAAAT---CGGGGGT-TGATT
U3_alyu48_v2      TAATACATACAACACGCTATGACAAAAGGGGAAAAAAGAAAAAT---CGGGGGT-TGATT
U3_alyu48_v1      TAATACATACAACACGCTATGACAAAAGGGGAAAAAAGAAAAAT---CGGGGGT-TGATT
U3_alyu42_43      TAATACATACAACACGCTATGACAAAAGGGGAAAAATGAAGAAT---CGGGGGT-TGATT
U3_alyu43_v2      TAATACATACAACACGCTATGACAAAAGGGGAAAAATGAAGAAT---CGGGGGT-TGATT
U3_alyu44_45      TAATACATACAACACGCTATGACAAAAGGGGAAAAATGAAGAAT---CGGGGGT-TGATT
U3_alyu55         TAATACATACAACACGCTATGACAAACGGGGAAAAAAGAAAAAT---CGGGGGT-TGATT
U3_alyu26         TAATACATACAACACGCTATGACAAACGGGGAAAAAAGAAAAAT---CGGGGGT-TGATT
U3_alyu54_v1      TAATACATACAACACGCTATGACAAACGGGGAAAAAAGAAAAAT---CGGGGGT-TGATT
U3_alyu54_v1      TAATACATACAACACGCTATGACAAACGGGGAAAAAAGAAAAAT---CGGGGGT-TGATT
U3_alyu35         TAGGACATACAACACGCTATGACAAAAGGGGAAAAAAGAAAAAT---CGGGGGT-TGATT
U3_alyu34_v2      TAATACATACAACACGCTATGACAAACGGGGAAAAAAGAAAAAT---CGGGGGT-TGATT
```

```
U3_alyu56         TAATACATACAACACGCTATGACAAACGGGGAAAAAAGAAAAAT---CGGGGGT-TGATT
U3_alyu25         TAATACATACAACACGCTATGACAAACGGGGAAAAAAGAAAAAT---CGGGGGT-TGATT
U3_alyu36         TAATACATACAACACGCTATGACAAACGGGGAAAAAAGAAAAAT---CGGGGGT-TGATT
U3_alyu34_v1      TAATACATACAACACGCTATGACAAACGGGGAAAAAAGAAAAAT---CGGGGGT-TGATT
U3_alyu39         TAATACATACAACACGCTATGACAAACGGGGAAAAAAGAAAAAT---CGGGGGT-TGATT
U3_alyu37         TAATACATACAACACGCTATGACAAACGGGGAAAAAAGAAAAAT---CGGGGGT-TGATT
U3_alyu33         TAATACATACAACACGCTATGACAAAAGGGGAAAAATGAAGAAT---CGGGGGT-TGATT
U3_alyu40         TAATACATACAACACGCTATGACAAAAGGGGAAAAAAGAAAAAT---CGGGGGT-TGATT
U3_alyu29         TAATACATACAACACGCTATGACAAAAGGGGAAAAAAGAAAAAT---CGGGGGT-TGATT
U3_alyu32         TAATACATACAACACGCTATGACAAAAGGGGAAAAAAGAAAAAT---CGGGGGT-TGATT
                   *  * ****** **  ***** * *   *     * **  ***   ****  * *****

U3_alyu14         GTT-TATCTTAACGCTTAAGGGCCGCAGGCCCGTCAAGTTGAA-----------------
U3_alyu15         GTT-TATCTTAACGCTTAAGGGCCGCAGGCCCGTCAAGTTGAA-----------------
U3_alyu19         GGT-TATCTTAACGATCTAGGGCCGTAGGCCCGT---GAGCAA-----------------
U3_jgf1_v1        GTA-TATCTTAACGATCTAGGGCCGTAGGCCCGT---GAGCAA-----------------
U3_jgf1_v2        GTA-TATCTTAACGATCTAGGGCCGTAGGCCCGT---GAGCAA-----------------
U3_alyu17_v1      GTG-TATCTTAACGATCTAGGGCCGTAGGCCCGT---GAGCAA-----------------
U3_alyu17_v2      GTG-TATCTTAACGATCTAGGGCCGTAGGCCCGT---GAGCAA-----------------
U3_alyu21_v1      GTG-TATCTTAACGCTTAAGGGCCGCAGGCCCGTCAAGTTGAA-----------------
U3_alyu21_v2      GTG-TATCTTAACGCTTAAGGGCCGCAGGCCCGTCAAGTTGAA-----------------
U3_alyu18         GTG-TATCTGAACGCTTAAGGGCCGCAGGCCCGTCAAGATGAA-----------------
U3_alyu24         GGGTTATCTTAACGATCTAGGGCCGTAGGCCCGT---GAGCAA-----------------
U3_alyu16         GTG-TATCTGAACGCTTAAGGGCCGCAGGCCCGTCAAGTTGAA-----------------
U3_alyu22         GTG-TATCTTAACGCTTAAGGGCCGCAGGCCCGTCAAGTTTGA-----------------
U3_alyu41         GGTCTATCCTAACGATTAAGGGCCGCAGGCCCGTCAAGATGGATCCAATAACCCGTTAAG
U3_alyu53         GGTCTATCCTAACGATTAAGGGCCGCAGGCCCGTCAAGATGGA-----------------
U3_alyu50         GGTCTATCCTAACGATTAAGGGCCGCAGGCCCGTCAAGATGGA-----------------
U3_alyu47         GGTCTATCCTAACGATTAAGGGCCGCAGGCCCGTCAAGATGGA-----------------
U3_alyu49         GGTCTATCCTAACGATTAAGGGCCGCAGGCCCGTCAAGATGGA-----------------
U3_alyu51         GGTCTATCCTAACGATTAAGGGCCGCAGGCCCGTCAAGATGGA-----------------
U3_alyu48_v2      GGTCTATCCTAACGATTAAGGGCCGCAGGCCCGTCAAGATGGA-----------------
U3_alyu48_v1      GGTCTATCCTAACGATTAAGGGCCGCAGGCCCGTCAAGATGGA-----------------
U3_alyu42_43      GGTCTATCGTATCGCTTAAGGGCCGCAGGCCCGTCAAGATGGA-----------------
U3_alyu43_v2      GGTCTATCGTATCGCTTAAGGGCCGCAGGCCCGTCAAGATGGA-----------------
U3_alyu44_45      GGTCTATCGTATCGCTTAAGGGCCGCAGGCCCGTCAAGATGGA-----------------
U3_alyu55         GGTCTATCCTAACGATTAAGGGCCGCAGGCCCGTCAAGATGGA-----------------
U3_alyu26         GGTCTATCCTAACGATTAAGGGCCGCAGGCCCGTCAAGATGGA-----------------
U3_alyu54_v1      GGTCTATCCTAACGATTAAGGGCCGCAGGCCCGTCAAGATGGA-----------------
U3_alyu54_v1      GGTCTATCCTAACGATTAAGGGCCGCAGGCCCGTCAAGATGGA-----------------
U3_alyu35         GGTCTATCCTAACGATTAAGGGCCGCAGGCCCGTCAAGATGGA-----------------
U3_alyu34_v2      GGTCTATCCTAACGATTAAGGGCCGCAGGCCCGTCAAGATGGA-----------------
U3_alyu56         GGTCTATCCTAACGATTAAGGGCCGCAGGCCCGTCAAGATGGA-----------------
U3_alyu25         GGTCTATCCTAACGATTAAGGGCCGCAGGCCCGTCAAGATGGA-----------------
U3_alyu36         GGTCTATCCTAACGATTAAGGGCCGCAGGCCCGTCAAGATGGA-----------------
U3_alyu34_v1      GGTCTATCCTAACGATTAAGGGCCGCAGGCCCGTCAAGATGGA-----------------
U3_alyu39         GGTCTATCCTAACGATTAAGGGCCGCAGGCCCGTCAAGATGGA-----------------
U3_alyu37         GGTCTATCCTAACGATTAAGGGCCGCAGGCCCGTCAAGATGGA-----------------
U3_alyu33         GGTCTATCCTAACGATTAAGGGCCGCAGGCCCGTCAAGATGGA-----------------
U3_alyu40         GGTCTATCCTAACGATTAAGGGCCGCAGGCCCGTCAAGATGGA-----------------
U3_alyu29         GGTCTATCCTAACGATTAAGGGCCGCAGGCCCGTCAAGATGGA-----------------
U3_alyu32         GGTCTATCGTATCGCTTAAGGGCCGCAGGCCCGTCAAGATGGA-----------------
                  *   ****  * ** *  ******* ********   *    *

U3_alyu14         ----TGAAC-GGTCCAGATTAATTCCTTAGCGACGAAGAAAGGAATCTTAATGGGGATAA
U3_alyu15         ----TGAAC-GGTCCAGATTAATTCCTTAGCGACGAAGAAAGGAATCTTGATGGGGATCA
U3_alyu19         ----TGAAC-GGTCCAGATTAATTCCTTAGCGACGAAGAAAGGAATCTTAATAGGGACCA
U3_jgf1_v1        ----TGAAC-GGTCCAGATTAATTCCTTAGCGACGAAGAAAGGAATCTTAATAGGGACCA
U3_jgf1_v2        ----TGAAC-GGTATAGATTAATTCCTTAGCGACGAAGAAAGGAATCTTAATAGGGACCA
U3_alyu17_v1      ----TGAAC-GGTCCAGATTAATTCCTTAGCGACGAAGAAAGGAATCTTAG-TGGGACCA
U3_alyu17_v2      ----TGAAC-GGTCCAGATTAATTCCTTAGCGACGAAGAAAGGAATCTTAG-TGGGACCA
U3_alyu21_v1      ----TGAAC-GGTCTAGATTAATTCCTTAGCGACGAAGAAAGGAATCTTAGTGGGGACCA
U3_alyu21_v2      ----TGAAC-GGTCTAGATTAATTCCTTAGCGACGAAGAAAGGAATCTTAGTGGGGACCA
U3_alyu18         ----TGAAC-GGTCCAGATTAATTCCTTAGCGACGAAGAAAAGAATCTTAATGGGGATCA
U3_alyu24         ----TGAAC-GGTCCAGATTAATTCCTTAGCGACGAAGAAAGGAATCTTAGTGGGGACCA
U3_alyu16         ----TGAAC-GGTCCAGATTAATTCCTTAGCGACGAAGAAAGGAATCTTAATGGGGACCA
U3_alyu22         ----TGAAC-GGTCCAGATTAATTCCTTAGCGACGAAGAAAGGAATCTTAGTGGGGACCA
U3_alyu41         AAGGTAAACGGGTCTAAAACGATTTCTTCGCCCGCAAGCAA--CACCTTTA---------
U3_alyu53         ----TGAAC-GGTTAGATTTGATTGCTTAGCCACGAAGAAAGGAATCTTTT-TGGGACCA
U3_alyu50         ----TGAAC-GGTTAGATTTGATTGCTTAGACACGAAGAAAGGAATCTTTT-TGGGACCA
U3_alyu47         ----TGAAC-GGTTAGATTTGATTGCTTAGACACGAAGAAAGGAATCTTTT-TGGGACCA
U3_alyu49         ----TGAAC-GGTTAGATTTGATTGCTTAGACACGAAGAAAGGAATCTTTT-TGGGACCA
U3_alyu51         ----TGAAC-GGTTAGATTTGATTGCTTAGACACGAAGAAAGGAATCTTTT-TGGGACCA
U3_alyu48_v2      ----TGAAC-GGTTAGATTTGATTGCTTAGACACGAAGAAAGGAATCTTTT-TAGGACCA
U3_alyu48_v1      ----TGAAC-GGTTAGATTTGATTGCTTAGACACGAAGAAAGGAATCTTTT-TAGGACCA
U3_alyu42_43      ----TGAAC-GGTCAGATTTGATTGCTTAGCCACGAAGAAAGGAATCTTTT-TGGGACCA
U3_alyu43_v2      ----TGAAC-GGTCAGATTTGATTGCTTAGCCACGAAGAAAGGAATCTTTT-TGGGACCA
U3_alyu44_45      ----TGAAC-GGCCAGATTTGATTGCTTAGCCACGAAGAAAGGAATCTTTT-TGGGACCA
U3_alyu55         ----TGAAC-GGTTAGATTTGATTGCTTAGCCACGAAGAAAGGAATCGTTT-TGGGACCA
U3_alyu26         ----TGAAC-GGTTAGATTTGATTGCTTAGCGACGAAGAAAGGAATCGTTT-TGGGACCA
U3_alyu54_v1      ----TGAAC-GGTTAGATTTGATTGCTTAGCCACGAAGAAAGGAATCGTTT-TGGGACCA
U3_alyu54_v1      ----TGAAC-GGTTAGATTTGATTGCTTAGCCACGAAGAAAGGAATCGTTT-TGGGACCA
U3_alyu35         ----TGAAC-GGTTAGATTTGATTGCTTAGCTACGAAGAAAGGAATCCTTT-TGGGACCA
U3_alyu34_v2      ----TGAAC-GGTTAGATTTGATTGCTTAGCCACGAAGAAAGGTATCGTTT-TGGGACCA
U3_alyu56         ----TGAAC-GGTTAGATTTGATTGCTTAGCTACGAAGAAAGGAATCGTTT-TGGGACCA
U3_alyu25         ----TGAAC-GGTTAGATTTGATTGCTTAGCCACGAAGAAAGGAATCGTTT-TGGGACCA
U3_alyu36         ----TGAAC-GGTTAGATTTGATTGCTTAGCCACGAAGAAAGGAATCGTTT-TGGGACCA
U3_alyu34_v1      ----TGAAC-GGTTAGATTTGATTGCTTAGCCACGAAGAAAGGAATCGTTT-TGGGACCA
U3_alyu39         ----TGAAC-GGTTAGATTTGATTGCTTAGCCACGAAGAAAGGAATCGTTT-TGGGACCA
U3_alyu37         ----TGAAC-GGTTAGATTTGATTGCTTAGCCACGAAGAAAGGAATCGTTT-TGGGACCA
U3_alyu33         ----TGAAC-GGTTAGATTTGATTGCTTAGCCAGAAGAAAGGAATCTTTT-TGGGACCA
U3_alyu40         ----TGAAC-GGTCAGATTTTATTGCTTAGCCACGAAGAAAGGAATCTTTT-TGGGACCA
U3_alyu29         ----TGAAC-GGTTAGATTTGATTGCTTAGCCACGAAGAAAGGAATCTTTTGTGGGACCA
U3_alyu32         ----TGAAC-GGTTAGATTTGATTGCTTAGCCACGAAGAAAGGAATCTTTT-TGGGACCA
                      * *** **         *** *** *     *** **   * * *
```

```
U3_alyu14         CATTAAAGACAGCTGTCATTGATTAA-ATAAATAATATAATATACAAAAGACTTATGTAC
U3_alyu15         CATTAAAGACAGCTGTCATTGATTAA-ATAAATAATATAATATACAAAAGACTTATGTAC
U3_alyu19         CATTAAAGACAGCTGTCATTGATTAA-ATAAATAATATAATAACCAAAAGACTTTTGTAC
U3_jgf1_v1        CATTAAAGACAGCTGTCATTGATTAA-ATAAATAATATAATATACAAAAGACTTTTGTAC
U3_jgf1_v2        CATTAAAGACAGCTGTCATTGATTAA-ATAAATAATATAATATACAAAAGACTTTTGTAC
U3_alyu17_v1      TATTAAAGACAGCTGTTATTGATTAA-ATAAATAATATAATATCCAAAAGACTTTTGTAC
U3_alyu17_v2      TATTAAAGACAGCTGTTATTGATTAA-ATAAATAATATAATATCCAAAAGACTTTTGTAC
U3_alyu21_v1      TATTAAAGACAGCTGTCATTGATTAA-ATAAATAATATAATATACAAAAGACTATTGTAC
U3_alyu21_v2      TATTAAAGACAGCTGTCATTGATTAA-ATAAATAATATAATATTCAAAAGACTATTGTAC
U3_alyu18         CATTAAAGACAGCTGTCATTGATTAA-ATAAATAATATAATATACAAAAGACTTTTGTAC
U3_alyu24         TATTAAAGACAACTGTTATTGATTAA-ATAAATAATATAATATCTAAAAGACTTTTGTAC
U3_alyu16         TATTAAAGACAGCTGTTATTGATTAA-ATAAATAATATAATATCCAAAAGACTTATGTAC
U3_alyu22         TATTAAAGACAACTGTCATTGATTAA-ATAAATAATATAATATCCAAAAGACTTTTGTAC
U3_alyu41         ----------ACCTCTGCGCACCTAT-ATATATAATATAATAACCAAATGACCATAGTGC
U3_alyu53         CAGATAAGACAGCTGTCACTACCTTT-AAAAATAATATAATAACCAATTGACGATAGTAC
U3_alyu50         CAGATAAGACAGCTGTCACTACTTTTAAAAAATAATATAATAACCAAATGACCATATTGC
U3_alyu47         CAGATAAGACAGCTGTCACTACTTTTAAAAAATAATATAATAACCAAATGACCATAGTGC
U3_alyu49         CAGATAAGACAGCTGTCACTACTTTTAAAAAATAATATAATAACCAAATGACCATAGTGC
U3_alyu51         CAGATAAGACAGCTGTCACTACTTTTAAAAAATAATATAATAACCAAATGACCATAGTGC
U3_alyu48_v2      CAGATAAGACAGCTGTCACTACTTTTAAAAAATAATATAATAACCAAATGACCATAGTGC
U3_alyu48_v1      CAGATAAGACAGCTGTCACTACTTTTAAAAAATAATATAATAACCAAATGACCATAGTGC
U3_alyu42_43      CAGATAAGACAGCTGTCATTACCTTT-TAAAATAATATAATAACCAATTGACGATAGTAC
U3_alyu43_v2      CAGATAAGACAGCTGTCATTACCTTT-TAAAATAATATAATAACCAATTGACGATAGTAC
U3_alyu44_45      CAGATAAGACAGCTGTCATTACCTTT-TAAAATAATATAATAACCAATTGACGATAGTAC
U3_alyu55         CAGACAAGACAGCTGTCACTACCTTTAAAAAATAATATAATAACCAATTGACGATAGTAC
U3_alyu26         CAGACAAGACAGCTGTCACTACTTTTAAAAAATAATATAATAACCAATTGACGATAGTAC
U3_alyu54_v1      CAGACAAGACAGCTGTCACTACTTTTAAAAAATAATATAATAACCAAATGACGATAGTAC
U3_alyu54_v1      CAGACAAGACAGCTGTCACTACTTTTAAAAAATAATATAATAACCAAATGACGATAGTAC
U3_alyu35         CAGACAAGACAGCTGTCACTACTTTTAAAAAATAATATAATAACCAAATGACGATAGTAC
U3_alyu34_v2      CAGACAAGACAGCTGTCACTACTTTTAAAAAATAATATAATAACCAAATGACGATAGTAC
U3_alyu56         CAGACAAGACAGCTGTCACTACTTTTAAAAAATAATATAATAACCAATTGACGATAGTAC
U3_alyu25         CTGACAAGACAGCTGTCACTACTTTTAAAAAATAATATAATAACTAATTGACGATAGTAC
U3_alyu36         CTGACAAGACAGCTGTCACTACTTTTAAAAAATAATATAATAACCAATTGACGATAGTAC
U3_alyu34_v1      CAGACAAGACAGCTGTCACTACTTTTAAAAAATAATATAATAACCAATTGACGATAGTAC
U3_alyu39         CAGACAAGACAGCTGTCACTACTTTTAAAAAATAATATAATAACCAATTGACGATAGTAC
U3_alyu37         CAGACAAGACAGCTGTCATTACTTTTAAAAAATAATATAATAACCAATTGACGATAGTAC
U3_alyu33         CAGATAAGACAGCTGTTACTACCTTT-AAAAATAATATAATATCCAATTGACGATAGTAC
U3_alyu40         CAGATAAGACAGCTGTCACTACTTTT-TAAAATAATATAATAACCAATTGACGATAGTAC
U3_alyu29         CAGACAAGACAGCTGTCACTACTTTT-TAAAATAATATAATAACCAATGGACGATACTAC
U3_alyu32         CAGATAAGACAGCTGTCATTACTTTT-TAAAATAATATAATAACCAAAGGACGATAGTAC
                            * ** *       *     * ***********   **  ***     * *

U3_alyu14         CCCTGATAATGATGACGTGTAGGGGTGTCCCGATGTAATATAGCATAGCTATGTGAAGAG
U3_alyu15         CCCTGATAATGATGACGTGTAGGGGTGTCCCGATGTAATATAGCATAGCTATGTGAAGAG
U3_alyu19         CCCTGATAATGATGACGTGTAGGGGTGTCCCGATGTAATATAGCATAGCTATGTGAAGAG
U3_jgf1_v1        CCCTGATAATGATGACGTGTAGGGGTGTCCCGATGTAATATAGCATAGCTATGTGAAGAG
U3_jgf1_v2        CCCTGATAATGATGACGTGTAGGGGTGTCCCGATGTAATATAGCATAGCTATGTGAAGAG
U3_alyu17_v1      CCCTGATAATGATGACGTGTAGGGGTGTCCCGATGTAATATAGCATAGCTATGTGAAGAG
U3_alyu17_v2      CCCTGATAATGATGACGTGTAGGGGTGTCCCGATGTAATATAGCATAGCTATGTGAAGAG
U3_alyu21_v1      CCCTGATAATGATGACGTGTAGGGGTGTCCCGATGTAATATAGCATAGCTATGTGAAGAG
U3_alyu21_v2      CCCTGATAATGATGACGTGTAGGGGTGTCCCGATGTAATATAGCATAGCTATGTGAAGAG
U3_alyu18         CCCTGATAATGATGACGTGTAGGGGTGTCCCGATGTAATATAGCATAGCTATGTGAAGAG
U3_alyu24         CCCTGAGAATGATGACGTGTAGGGGTGTCCCGATGTAATATAGCATAGCTATGCGAAGAG
U3_alyu16         CCCTGATAATGTTGACGTGTAGGGGTGTCCCGATGTAATATAGCATAGCTATGTGAAGAG
U3_alyu22         CCCTGAGAATGATGACGTGTAGGGGTGTCCCGATGTAATATAGCATAGCTATGCGAAGAG
U3_alyu41         CCCTCCCAAAGATGACGTATAGGGGTGTCCCGAGGTAATTTAACATAGCTCTGATAAGAG
U3_alyu53         CCCTCCCAAAGATGACGTATAGGGGTGTCCCGAGGTAATTTAACATAGCTCTGACAAGAG
U3_alyu50         CCCTCCCAAAGATGACGTATAGGGGTGTCCCGAGGTAATTTAACATAGCTCTGACAAGAG
U3_alyu47         CCCTCCCAAAGATGACGTATAGGGGTGTCCCGAGGTAATTTAACATAGCTCTGACAAGAG
U3_alyu49         CCCTCCCAAAGATGACGTATAGGGGTGTCCCGAGGTAATTTAACATAGCTCTGACAAGAG
U3_alyu51         CCCTCCCAAAGATGACGTATAGGGGTGTCCCGAGGTAATTTAACATAGCTCTGATAAGAG
U3_alyu48_v2      CCCTCCCAAAGATGACGTATAGGGGTGTCCCGAGGTAATTTAACATAGCTCTGACAAGAG
U3_alyu48_v1      CCCTCCCAAAGATGACGTATAGGGGTGTCCCGAGGTAATTTAACATAGCTCTGACAAGAG
U3_alyu42_43      CCCTCCCAAAGATGACGTATAGGGGTGTCCCGAGGTAATTTAACATAGCTCTGACAAGAG
U3_alyu43_v2      CCCTCCCAAAGATGACGTATAGGGGTGTCCCGAGGTAATTTAACATAGCTCTGACAAGAG
U3_alyu44_45      CCCTCCCAAAGATGACGTATAGGGGTGTCCCGAGGTAATTTAACATAGCTCTGACAAGAG
U3_alyu55         CCCTCCCAAAGATGACGTATAGGGGTGTCCCGAGGTAATTTAACATAGCTCTGACAAGAG
U3_alyu26         CCCTCCCAAAGATGACGTATAGGGGTGTCCCGAGGTAATTTAACATAGCTCTGACAAGAG
U3_alyu54_v1      CCCTCCCAAAGATGACGTATAGGGGTGTCCCGAGGTAATTTAACATAGCTCTGACAAGAG
U3_alyu54_v1      CCCTCCCAAAGATGACGTATAGGGGTGTCCCGAGGTAATTTAACATAGCTCTGACAAGAG
U3_alyu35         CCCTCCCAAAGATGACGTATAGGGGTGTCCCGAGGTAATTTAACATAGCTCTGACAAGAG
U3_alyu34_v2      CCCTCCCAAAGATGACGTATAGGGGTGTCCCGAGGTAATTTAACATAGCTCTGACAAGAG
U3_alyu56         CCCTACCAAAGATGACGTATAGGGGTGTCCCGAGGTAATTTAACATAGCTCTGACAAGAG
U3_alyu25         CCCTCCCAAAGATGACGTATAGGGGTGTCCCGAGGTAATTTAACATAGCTCTGACAAGAG
U3_alyu36         CCCTCCCAAAGATGACGTATAGGGGTGTCCCGAGGTAATTTAACATAGCTCTGACAAGAG
U3_alyu34_v1      CCCTCCCAAAGATGACGTATAGGGGTGTCCCGAGGTAATTTAACATAGCTCTGACAAGAG
U3_alyu39         CCCTCCCAAAGATGACGTATAGGGGTGTCCCGAGGTAATTTAACATAGCTCTGACAAGAG
U3_alyu37         CCCTCCCAAAGATGACGTATAGGGGTGTCCCGAGGTAATTTAACATAGCTCTGACAAGAG
U3_alyu33         CCCTCCCAAAGATGACGTATAGGGGTGTCCCGAGGTAATTTAACATAGCTCTGACAAGAG
U3_alyu40         CCCTCCCAAAGATGACGTATAGGGGTGTCCCGAGGTAATTTAACATAGCTCTGACAAGAG
U3_alyu29         CCCTCCCAAAGATGACGTATAGGGGTGTCCCGAGGTAATTTAACATAGCTCTGACAAGAG
U3_alyu32         CCCTCCCAAAGATGACGTATAGGGGTGTCCCGAGGTAATTTAACATAGCTCTGACAAGAG
                  ****   ** * ****** ************** ***** ** ******* **  *****

U3_alyu14         ATAAGCATGTGGCCGACAACTTTAGTTGTGGGCCAGATGTCCCGAGTTAGTGCGCC----
U3_alyu15         ATAAGCATGTGGCCGACAACTTTAGTTGTGGGCCAGATGTCCCGAGTTAGTGCGCC----
U3_alyu19         ATAAGCATTTGGCCGACAACTTTAGTGGTGGGCCAGATGTCCCGAGTTAGTGCGCC----
U3_jgf1_v1        ATAAGCATGTGGCCGACAACTTTAGTGGTGGGCCAGATGTCCCGAGTTAGTGCGCC----
U3_jgf1_v2        ATAAGCATGTGGCCGACAACTTTAGTGGTGGGCCAGATGTCCCGAGTTAGTGCGCC----
U3_alyu17_v1      ATAAGCATGTGGCCGACAACTTTAGTGGTGGGCCAGATGTCCCGAGTTAGTGCGCC----
U3_alyu17_v2      ATAAGCATGTGGCCGACAACTTTAGTAGTGGGCCAGATGTCCCGAGTTAGTGCGCC----
U3_alyu21_v1      ATAAGCATGTGGCCGACAACTTTAGTGGTGGGCCAGATGTCCCGAGTTAGTGCGCC----
U3_alyu21_v2      ATAAGCATGTGGCCG----------TGGTGGGCCAGATGTCCCGAGTTAGTGCGCC----
U3_alyu18         ATAAGCATGTGGCCGACAACTTTAGTGGTGGGCCAAATGTCCCGAGTTAGTGCGCC----
```

```
U3_alyu24        ATAAGCATGTGGCCGACAACTTTAGTGGTGGGCCAGATGTCCCGAGTTAGTGCGCC----
U3_alyu16        ATAAGCATGTGGCCGACAACTTTAGTGGTGGGCCAGATGTCCCGAGTTAGTGCGCC----
U3_alyu22        ATAAGCATGTGGCCGACAACTTTAGTGGTGGGCCAGATGTCCCGAGTTAGTGCGCC----
U3_alyu41        ATA-----GTGGACGTTGGATTCCACGATCGGACGATCGT---GGTTTGATGAACTTATG
U3_alyu53        ATA-----GTGGACGTTGGATGCCACGATCGGACGATCGT---GGTTTGATGAACTTATG
U3_alyu50        ATA-----GTGGACGTTGGATTCCACGATCGGACGATCGT---GGTTTGATGAACTTATG
U3_alyu47        ATA-----GTGGACGTTGGATTCCACGATCGGACGATCGT---GGTTTGATGAACTTATG
U3_alyu49        ATA-----GTGGACGTTGGATTCCACGATCGGACGATCGT---GGTTTGATGAACTTATG
U3_alyu51        ATA-----GTGGACGTTGGATTCCACGATCGGACGATCGT---GGTTTGATGAACTTATG
U3_alyu48_v2     ATA-----GTGGACGTTGGATTCCACGATCGGACGATCGT---GGTTTGATGAACTTATG
U3_alyu48_v1     ATA-----GTGGACGTTGGATTCCACGATCGGACGATCGT---GGTTTGATGAACTTATG
U3_alyu42_43     ATA-----GTGGACGTTGGATGCCACGATCGGACGATCGT---GGTTTGATGAACTTATG
U3_alyu43_v2     ATA-----GTGGACGTTGGATGCCACGATCGGACGATCGT---GGTTTGATGAACTTATG
U3_alyu44_45     ATA-----GTGGACGTTGGATGCCACGATCGGACGATCGT---GGTTTGATGAACCTATG
U3_alyu55        ATA-----GTGGACGTTGGATGCCACGATCGGACGATCGT---GGTTTGATGAACTTATG
U3_alyu26        ATA-----GTGGACGTTGGATGCCACGATCGGACGATCGT---GGTTTGATGAACCAATG
U3_alyu54_v1     ATA-----GTGGACGTTGGATGCCACGATCGGACGATCGT---GGTTTGACGAACCAATG
U3_alyu54_v1     ATA-----GTGGACGTTGGATGCCACGATCGGACGATCGT---GGTTTGACGAACCAATG
U3_alyu35        ATA-----GTGGACGTTGGATGCCACGATCGGACGATCGT---GGTTTGATGAACTAATG
U3_alyu34_v2     ATA-----GTGGACGTTGGATCCCACGATCGGACGATCGT---GGTTTGATGAACTAATG
U3_alyu56        ATA-----GTGGACGTTGGATGCCACGATCGGACGATCGT---GGTTTGATGAACCAATG
U3_alyu25        ATA-----GTGGACGTTGGATGCCACGATCGGACGATCGT---GGTTTGATGAACTAATG
U3_alyu36        ATA-----GTGGACGTTGGATGCCACGATCGGACGATCGT---GGTTTGATGAACTAATG
U3_alyu34_v1     ATA-----GTGGACGTTGGATGCCACGATCGGACGATCGT---GGTTTGATGAACCAATG
U3_alyu39        ATA-----GTGGACGTTGGATGCCACGATCGGACGATCGT---GGTTTGATGAACCAATG
U3_alyu37        ATA-----GTGGACGTTGGATGGCACGATCGGACGATCGT---GGTTTGATGAACCAATG
U3_alyu33        ATA-----GTGGACGTTGGATGCCACGATCGGACGATCGT---GGTTTGATGAACTAATG
U3_alyu40        ATA-----GTGGACGTTGGATGCCACGATCGGACGATCGT---GGTTTGATGAACTAATG
U3_alyu29        ATA-----GTGGACGTTGGATGCCACGATCGGACGATCGT---GGTTTGATGAACTAATG
U3_alyu32        ATA-----GTGGACGTTGGATGCCACGATCGGACGATCGT---GGTTTGATGAACCAATG
                 ***      *** **             * ** *    **   *  **   *  *

U3_alyu14        ---ACGTAAGCACGGGGGACTAT
U3_alyu15        ---ACGTAAGCACGGGGGACTAT
U3_alyu19        ---ACGTAAGCGCTGGGGCTTAT
U3_jgf1_v1       ---ACGTAAGCACGGGGGACTAT
U3_jgf1_v2       ---ACGTAAGCACGGGGGACTAT
U3_alyu17_v1     ---ACGTAAGCGCTGGGGCTTAT
U3_alyu17_v2     ---ACGTAAGCACGGGGGACTAT
U3_alyu21_v1     ---ACGTAAGCGCTGGGGACTAT
U3_alyu21_v2     ---ACGTAAGCACGGGGGACTAT
U3_alyu18        ---ACGTAAGCACGGGGGACTAT
U3_alyu24        ---ACGTAAGCACGGGGGACTAT
U3_alyu16        ---ACGTAAGCACGGGGGACTAT
U3_alyu22        ---ACGTAAGCACGGGGGACTAT
U3_alyu41        ATGACGTAGGGGCTGGGGCTTAT
U3_alyu53        ATGACGTAGGGGCTGGGGCTTAT
U3_alyu50        ATGACGTAGGGGCTGGGGCTTAT
U3_alyu47        ATGACGTAGGGGCTGGGGCTTAT
U3_alyu49        ATGACGTAGGGGCTGGGGCTTAT
U3_alyu51        ATGACGTAGGGGCTGGGGCTTAT
U3_alyu48_v2     CTGACGTAGGGGCTGGGGCTTAT
U3_alyu48_v1     ATGACGTAGGGGCTGGGGCTTAT
U3_alyu42_43     ATGACGTAGGGGCTGGGGCTTAT
U3_alyu43_v2     ATGACGTAGGGGCTGGGGCTTAT
U3_alyu44_45     ATGACGTAGGGGCTGGGGCTTAT
U3_alyu55        ATGACGTAGGGGCTGGGGCTTAT
U3_alyu26        ATGACGTAGGGGCTGGGGCTTAT
U3_alyu54_v1     ATGACGTAGGGGCTGGGGCTTAT
U3_alyu54_v1     ATGACGTAGGGGCTGGGGCTTAT
U3_alyu35        ATGACGTAGGGGCTGGGGCTTAT
U3_alyu34_v2     ATGACGTAGGGGCTGGGGCTTAT
U3_alyu56        ATGACGTAGGGGCTGGGGCTTAT
U3_alyu25        ATGACGTAGGGGCTGGGGCTTAT
U3_alyu36        ATGACGTAGGGGCTGGGGCTTAT
U3_alyu34_v1     ATGACGTAGGGGCTGGGGCTTAT
U3_alyu39        ATGACGTAGGGGCTGGGGCTTAT
U3_alyu37        ATGACGTAGGGGCTGGGGCTTAT
U3_alyu33        ATGACGTAGGGGCTGGGGCTTAT
U3_alyu40        ATGACGTAGGGGCTGGGGCTTAT
U3_alyu29        ATGACGTAGGGGCTGGGGCTTAT
U3_alyu32        ATGACGTAGGGGCTGGGGCTTAT
                    ***** *  * ****  ***
```
