# Supplementary material for: Evidence for Dicot Plants as Alternative Hosts of Banana Bunchy Top Virus and Its Alphasatellites in South-East Asia
Source: Pathogens. 2023 Oct 28;12(11):1289. doi: 10.3390/pathogens12111289 (PMC10675457; doi:10.3390/pathogens12111289)
Supplement: Supplementary file 1 [file pathogens-12-01289-s001.zip › Supplementary Figure S1.pdf]

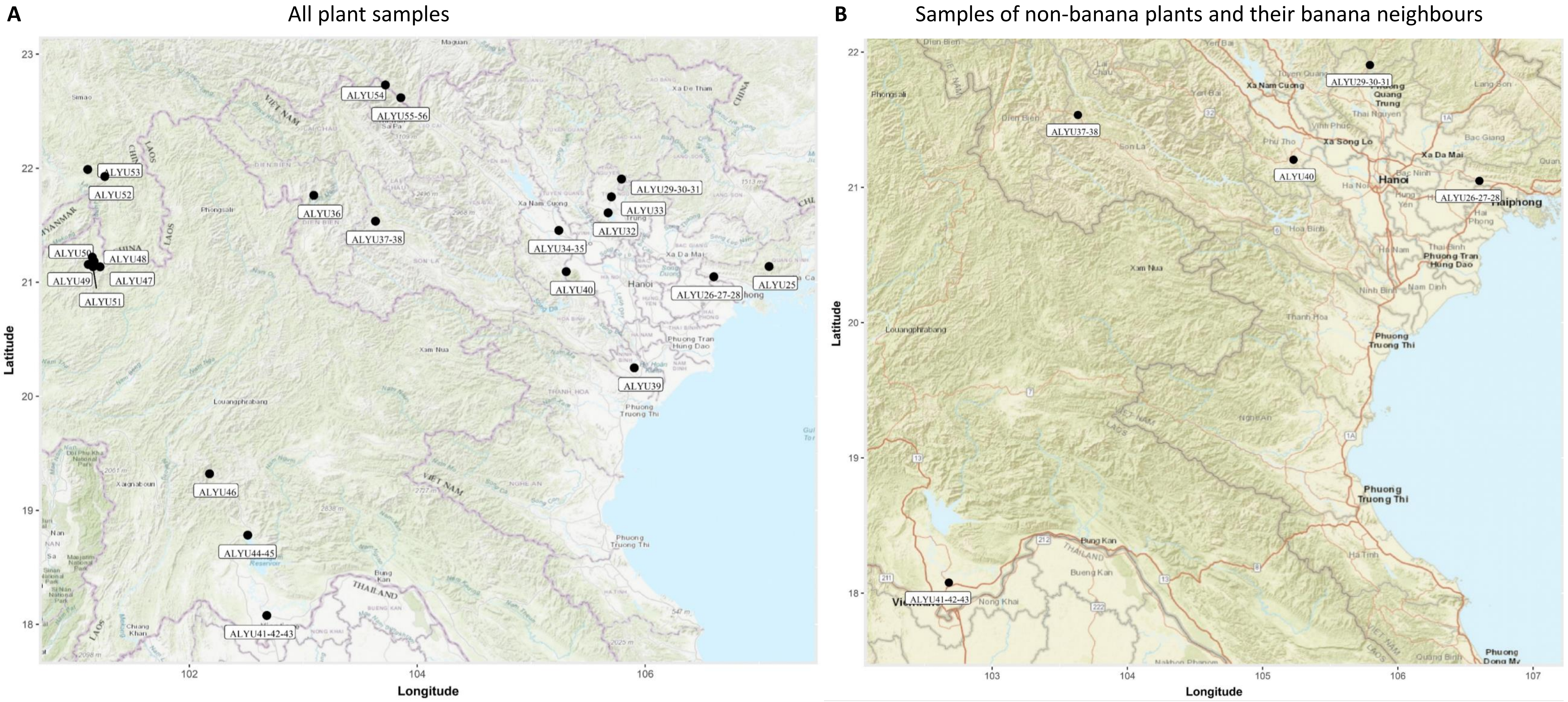

Figure S1. Locations of banana and non-banana plant leaf sampling places in Vietnam, Laos and China in 2018 and 2019. (A) All plant samples. **(B)** Samples of non-banana plants and their banana neighbors. Sample names (ALYU-24 to ALYU-56) are indicated at each location. The map was created based on the GPS coordinates using R studio and package OpenStreepMap.
