# Supplementary material for: Evidence for Dicot Plants as Alternative Hosts of Banana Bunchy Top Virus and Its Alphasatellites in South-East Asia
Source: Pathogens. 2023 Oct 28;12(11):1289. doi: 10.3390/pathogens12111289 (PMC10675457; doi:10.3390/pathogens12111289)
Supplement: Supplementary file 1 [file pathogens-12-01289-s001.zip › Supplementary Figure S2.pdf]

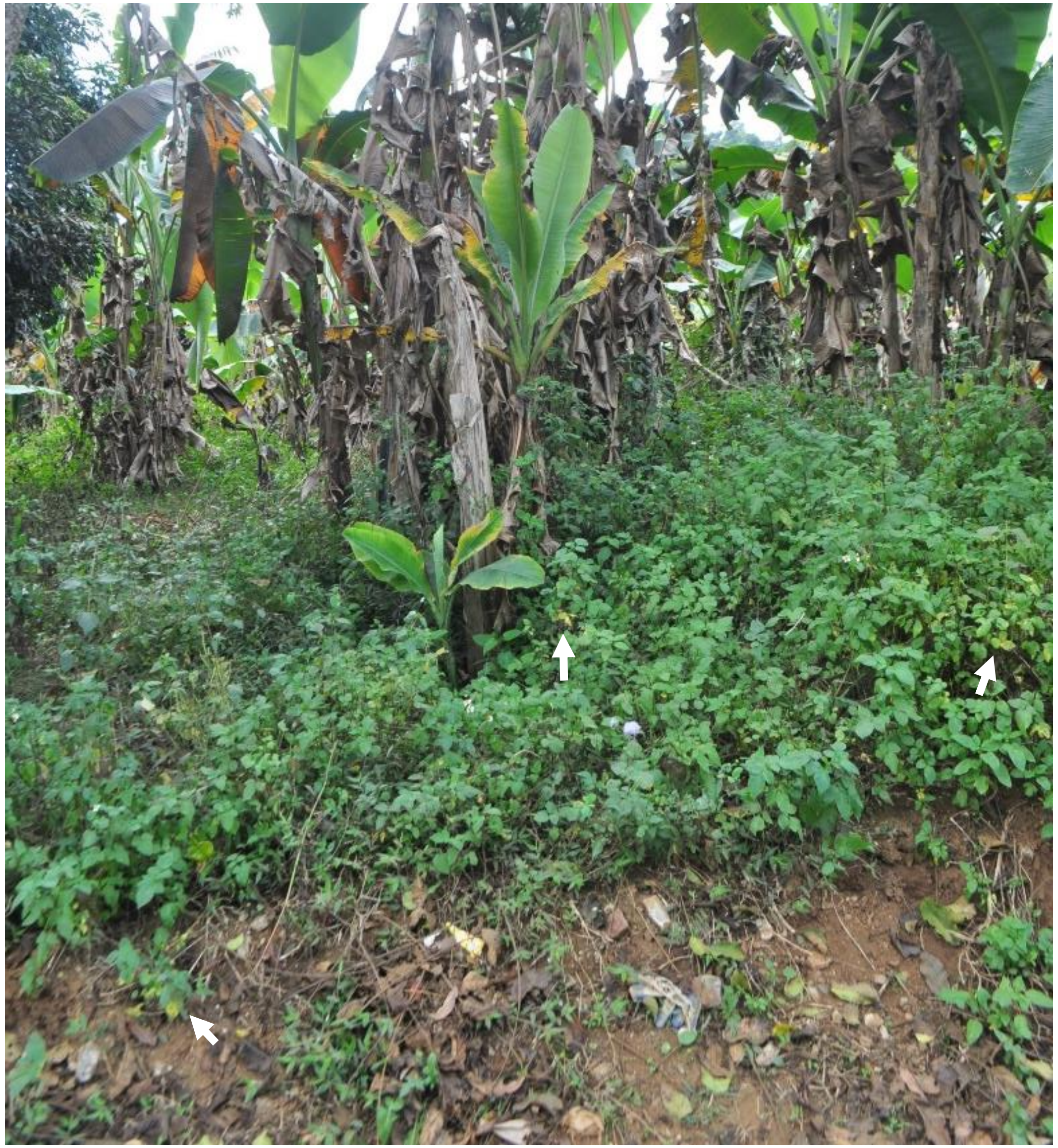

Figure S2. *Bidens pilosa* and its neighbor banana plants. White arrows point at virus-like chlorotic symptoms on leaves of *B. pilosa* plants.
