# Supplementary material for: Evidence for Dicot Plants as Alternative Hosts of Banana Bunchy Top Virus and Its Alphasatellites in South-East Asia
Source: Pathogens. 2023 Oct 28;12(11):1289. doi: 10.3390/pathogens12111289 (PMC10675457; doi:10.3390/pathogens12111289)
Supplement: Supplementary file 1 [file pathogens-12-01289-s001.zip › Supplementary Figure S4.pdf]

Plant	Samples	BBTV reads	BBTA+alpha reads	Total number reads	% of viral reads
Musa	ALYU-26	2,343,872	7,190,098	10,455,772	0.6376
Ipomoea	ALYU-28	2,170	3,376	12,488,618	0.0003
Musa	ALYU-29	1,088,396	8,063,084	11,642,444	0.6926
Phyllanthus	ALYU-30	6,022	7,842	13,503,218	0.0006
Arachis	ALYU-31	1,042	1,452	10,347,832	0.0001
Musa	ALYU-46	259	467	18,154,862	0.0000
Musa	ALYU-52	1,685	1,885	10,763,616	0.0002

Plant	Samples	BBTV reads	BBTA+alpha reads	Total number reads	% of viral reads
Musa	ALYU-26	2,343,872	7,190,098	10,455,772	0.6376
Commelina	ALYU-27	14,932	53,512	11,731,770	0.0043
Musa	ALYU-37	6,742,128	9,793,147	10,828,680	0.9044
Bidens	ALYU-38	2,435,471	2,808,035	11,763,264	0.2387
Chromolaena	ALYU-41	6,999	17,142	11,534,294	0.0015
Musa	ALYU-42	1,132,366	1,747,470	13,002,052	0.1344
Musa	ALYU-43	1,278,482	2,905,982	13,238,836	0.2195

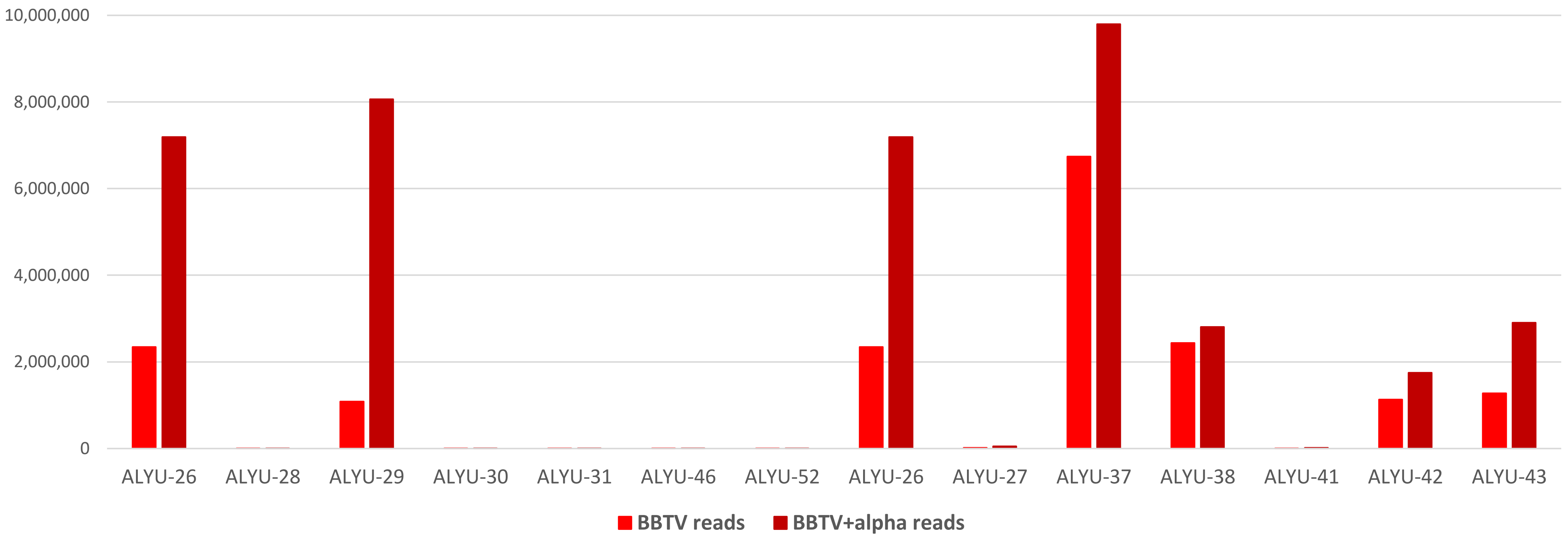

Figure S4. Counts of Illumina reads representing BBTV and alphasatellite DNA from banana and non-banana neighbor plants. Total DNA from banana (ALYU-26, ALYU-29, ALYU-37, ALYU-42-43, ALYU-46, ALYU-52) and non-banana (ALYU-27-28, AYLU-30-31, ALYU-38, ALYU-41) plants was treated by RCA, RCA products were Illumina sequenced and the sequencing reads were used for *de novo* reconstruction of BBTV and alphasatellite genomes. The Illumina reads were then mapped onto the reconstructed BBTV and alphasatellite genomes present in each sample (or in the case of Ipomoea, Phyllantus, Arachis and bananas ALYU-46 and ALYU-52 onto the BBTV and alphasatellite genomes present in infected banana neighbors) and counted. Counts are given in tables and plotted as a bar-graph.
